# Supplementary material for: Existing guidance on reporting of consensus methodology: a systematic review to inform ACCORD guideline development
Source: BMJ Open. 2022 Sep 8;12(9):e065154. doi: 10.1136/bmjopen-2022-065154 (PMC9462098; doi:10.1136/bmjopen-2022-065154)
Supplement: Supplementary data [file bmjopen-2022-065154supp001.pdf]

**ACCORD - January 7th, 2022**

Regular references:

Total: 2599 references, sourced from:

- Web of Science - core collection: 1775
- MEDLINE (Web of Science): 1501 - 202 unique
- PubMed: 375 - 219 unique
- MEDLINE (OVID): 641 - 174 unique
- Embase (OVID): 331 - 66 unique
- Cochrane Library: 131 - 77 unique
- Emcare (OVID): 179 - 29 unique
- Academic Search Premier: 280 - 23 unique
- PsycINFO: 173 - 34 unique

Meeting abstract references:

Total: 137 references, sourced from:

- Web of Science: 14
- Embase (OVID): 99 - 90 unique
- Cochrane Library: 36 - 33 unique

Known references:

- PubMed: 27841062 26796090 25587865 26395179 24581294
- MEDLINE (Web of Science): PMID=(27841062 OR 26796090 OR 25587865 OR 26395179 OR 24581294)
- Web of Science Core Collection: UT=(000393885800003 OR 000375153500022 OR 000376181900007 OR 000361506400001 OR 000334256400007 OR 000309802600012 OR 000321232400002 OR 000309802600012 OR 000465105500070)

Databases:

**Web of Science Core Collection and MEDLINE (Web of Science)**
http://isiknowledge.com/wos

((TI=("Delphi Technique" OR "Delphi Technique" OR "Delphi techniques" OR "Delphi method" OR "Delphi methods" OR "Delphi study" OR "Delphi studies" OR "Delphi survey" OR "Delphi surveys" OR "Delphi consensus" OR "Delphi based consensus" OR "Delphi questionnaire" OR "Delphi questionnaires" OR "Delphi research" OR "Delphi review" OR "Delphi reviews" OR "Delphi process" OR "Delphi processes" OR "Delphi based" OR "Delphi procedure" OR "Delphi procedures" OR "Delphi assessment" OR "Delphi assessments" OR "Delphi approach" OR "Delphi approaches" OR "Delphi panel" OR "Delphi panels" OR "Delphi round" OR "Delphi rounds" OR "Delphi analysis" OR

"Delphi expert" OR "Delphi experts" OR "Delphi consultation" OR "Delphi methodology" OR "nominal group technique" OR "nominal group techniques" OR "nominal group" OR "nominal groups" OR "nominal grouping" OR "consensus recommendation" OR "consensus recommendations" OR "consensus development" OR "consensus activity" OR "consensus activities" OR "Consensus Development Conference" OR "Consensus Development" OR "Consensus methodology" OR "consensus method*" OR "RAND" OR (("Guidelines" OR "guideline") NEAR/2 ("consensus" OR "delphi"))) OR AB=("Delphi Technique" OR "Delphi Technique" OR "Delphi techniques" OR "Delphi method" OR "Delphi methods" OR "Delphi study" OR "Delphi studies" OR "Delphi survey" OR "Delphi surveys" OR "Delphi consensus" OR "Delphi based consensus" OR "Delphi questionnaire" OR "Delphi questionnaires" OR "Delphi research" OR "Delphi review" OR "Delphi reviews" OR "Delphi process" OR "Delphi processes" OR "Delphi based" OR "Delphi procedure" OR "Delphi procedures" OR "Delphi assessment" OR "Delphi assessments" OR "Delphi approach" OR "Delphi approaches" OR "Delphi panel" OR "Delphi panels" OR "Delphi round" OR "Delphi rounds" OR "Delphi analysis" OR "Delphi expert" OR "Delphi experts" OR "Delphi consultation" OR "Delphi methodology" OR "nominal group technique" OR "nominal group techniques" OR "nominal group" OR "nominal groups" OR "nominal grouping" OR "consensus recommendation" OR "consensus recommendations" OR "consensus development" OR "consensus activity" OR "consensus activities" OR "Consensus Development Conference" OR "Consensus Development" OR "Consensus methodology" OR "consensus method*" OR "RAND") OR AK=("Delphi Technique" OR "Delphi Technique" OR "Delphi techniques" OR "Delphi method" OR "Delphi methods" OR "Delphi study" OR "Delphi studies" OR "Delphi survey" OR "Delphi surveys" OR "Delphi consensus" OR "Delphi based consensus" OR "Delphi questionnaire" OR "Delphi questionnaires" OR "Delphi research" OR "Delphi review" OR "Delphi reviews" OR "Delphi process" OR "Delphi processes" OR "Delphi based" OR "Delphi procedure" OR "Delphi procedures" OR "Delphi assessment" OR "Delphi assessments" OR "Delphi approach" OR "Delphi approaches" OR "Delphi panel" OR "Delphi panels" OR "Delphi round" OR "Delphi rounds" OR "Delphi analysis" OR "Delphi expert" OR "Delphi experts" OR "Delphi consultation" OR "Delphi methodology" OR "nominal group technique" OR "nominal group techniques" OR "nominal group" OR "nominal groups" OR "nominal grouping" OR "consensus recommendation" OR "consensus recommendations" OR "consensus development" OR "consensus activity" OR "consensus activities" OR "Consensus Development Conference" OR "Consensus Development" OR "Consensus methodology" OR "consensus method*" OR "RAND" OR (("Guidelines" OR "guideline") NEAR/2 ("consensus" OR "delphi")))) AND (TI=("quality of reporting" OR "reporting quality" OR "reporting qualities" OR "selective reporting" OR "poor reporting" OR "poor reported" OR "reporting guideline" OR "reporting" OR ("reporting" AND ("quality" OR "selective" OR "poor" OR "weak" OR "manner" OR "rigor" OR "improv*")) OR "Data Accuracy" OR "quality assessment" OR "strengths" OR "strength" OR "weaknesses" OR "weakness" OR "research method" OR "research methods" OR "research method*") OR AK=("quality of reporting" OR "reporting quality" OR "reporting qualities" OR "selective reporting" OR "poor reporting" OR "poor reported" OR ("reporting" NEAR/5 ("quality" OR "selective" OR "poor" OR "weak" OR "manner" OR "rigor" OR "improv*")) OR "Data Accuracy" OR

"Research Report standards" OR "quality assessment" OR "strengths" OR "strength" OR "weaknesses" OR "weakness" OR "research method" OR "research methods" OR "research method*") OR AB=("quality of reporting" OR "reporting quality" OR "reporting qualities" OR "selective reporting" OR "poor reporting" OR "poor reported" OR ("reporting" NEAR/5 ("quality" OR "selective" OR "poor" OR "weak" OR "manner" OR "rigor" OR "improv*")) OR "Data Accuracy" OR "Research Report standards" OR "quality assessment" OR "strengths" OR "strength" OR "weaknesses" OR "weakness")))

**PubMed**
http://www.ncbi.nlm.nih.gov/pubmed?otool=leiden

(("Delphi Technique"[majr] OR "Delphi Technique"[ti] OR "Delphi techniques"[ti] OR "Delphi method"[ti] OR "Delphi methods"[ti] OR "Delphi study"[ti] OR "Delphi studies"[ti] OR "Delphi survey"[ti] OR "Delphi surveys"[ti] OR "Delphi consensus"[ti] OR "Delphi based consensus"[ti] OR "Delphi questionnaire"[ti] OR "Delphi questionnaires"[ti] OR "Delphi research"[ti] OR "Delphi review"[ti] OR "Delphi reviews"[ti] OR "Delphi process"[ti] OR "Delphi processes"[ti] OR "Delphi based"[ti] OR "Delphi procedure"[ti] OR "Delphi procedures"[ti] OR "Delphi assessment"[ti] OR "Delphi assessments"[ti] OR "Delphi approach"[ti] OR "Delphi approaches"[ti] OR "Delphi panel"[ti] OR "Delphi panels"[ti] OR "Delphi round"[ti] OR "Delphi rounds"[ti] OR "Delphi analysis"[ti] OR "Delphi expert"[ti] OR "Delphi experts"[ti] OR "Delphi consultation"[ti] OR "Delphi methodology"[ti] OR "nominal group technique"[ti] OR "nominal group techniques"[ti] OR "nominal group"[ti] OR "nominal groups"[ti] OR "nominal grouping"[ti] OR "consensus recommendation"[ti] OR "consensus recommendations"[ti] OR "consensus development"[ti] OR "consensus activity"[ti] OR "consensus activities"[ti] OR "consensus methodology"[ti] OR "consensus method*"[ti] OR "Consensus Development Conferences as Topic"[majr] OR "RAND"[ti] OR ("Guidelines as Topic"[majr:noexp] AND ("consensus"[tw] OR "delphi"[tw]))) AND ("reporting"[ti] OR "quality of reporting"[tw] OR "reporting quality"[tw] OR "reporting qualities"[tw] OR "selective reporting"[tw] OR "poor reporting"[tw] OR "poor reported"[tw] OR "poorly reported"[tw] OR "Research Report/standards"[majr] OR "Research Design/standards"[mesh] OR "Research Design"[majr:noexp] OR "Writing/standards"[mesh] OR "Writing"[majr] OR "research method"[ti] OR "research methods"[ti] OR "research method*"[ti]))

**MEDLINE via OVID**
http://gateway.ovid.com/ovidweb.cgi?T=JS&MODE=ovid&NEWS=n&PAGE=main&D=medall

((exp *"Delphi Technique"/ OR "Delphi Technique".ti OR "Delphi techniques".ti OR "Delphi method".ti OR "Delphi methods".ti OR "Delphi study".ti OR "Delphi studies".ti OR "Delphi survey".ti OR "Delphi surveys".ti OR "Delphi consensus".ti OR "Delphi based consensus".ti OR "Delphi questionnaire".ti OR "Delphi questionnaires".ti OR "Delphi research".ti OR "Delphi review".ti OR "Delphi reviews".ti OR "Delphi

process".ti OR "Delphi processes".ti OR "Delphi based".ti OR "Delphi procedure".ti OR "Delphi procedures".ti OR "Delphi assessment".ti OR "Delphi assessments".ti OR "Delphi approach".ti OR "Delphi approaches".ti OR "Delphi panel".ti OR "Delphi panels".ti OR "Delphi round".ti OR "Delphi rounds".ti OR "Delphi analysis".ti OR "Delphi expert".ti OR "Delphi experts".ti OR "Delphi consultation".ti OR "Delphi methodology".ti OR "nominal group technique".ti OR "nominal group techniques".ti OR "nominal group".ti OR "nominal groups".ti OR "nominal grouping".ti OR "consensus recommendation".ti OR "consensus recommendations".ti OR "consensus development".ti OR "consensus activity".ti OR "consensus activities".ti OR "consensus methodology".ti OR "consensus method*".ti OR exp *"Consensus Development Conferences as Topic"/ OR "RAND".ti OR ("Guidelines as Topic"/ AND ("consensus".mp OR "delphi".mp)) OR (("Guidelines".mp OR "guideline".mp) ADJ2 ("consensus".mp OR "delphi".mp))) AND ("reporting".ti OR "quality of reporting".mp OR "reporting quality".mp OR "reporting qualities".mp OR "selective reporting".mp OR "poor reporting".mp OR "poor reported".mp OR "poorly reported".mp OR "Research Report/standards"/ OR exp "Research Design"/st OR *"Research Design"/ OR exp "Writing"/st OR exp *"Writing"/ OR "research method".ti OR "research methods".ti OR "research method*".ti OR ("reporting" ADJ8 ("quality" OR "selective" OR "poor" OR "weak" OR "manner" OR "rigor" OR "improv*")).mp))

**Embase**
http://ovidsp.ovid.com/ovidweb.cgi?T=JS&PAGE=main&MODE=ovid&D=oemezd

((exp *"Delphi Study"/ OR "Delphi Technique".ti OR "Delphi techniques".ti OR "Delphi method".ti OR "Delphi methods".ti OR "Delphi study".ti OR "Delphi studies".ti OR "Delphi survey".ti OR "Delphi surveys".ti OR "Delphi consensus".ti OR "Delphi based consensus".ti OR "Delphi questionnaire".ti OR "Delphi questionnaires".ti OR "Delphi research".ti OR "Delphi review".ti OR "Delphi reviews".ti OR "Delphi process".ti OR "Delphi processes".ti OR "Delphi based".ti OR "Delphi procedure".ti OR "Delphi procedures".ti OR "Delphi assessment".ti OR "Delphi assessments".ti OR "Delphi approach".ti OR "Delphi approaches".ti OR "Delphi panel".ti OR "Delphi panels".ti OR "Delphi round".ti OR "Delphi rounds".ti OR "Delphi analysis".ti OR "Delphi expert".ti OR "Delphi experts".ti OR "Delphi consultation".ti OR "Delphi methodology".ti OR "nominal group technique".ti OR "nominal group techniques".ti OR "nominal group".ti OR "nominal groups".ti OR "nominal grouping".ti OR "consensus recommendation".ti OR "consensus recommendations".ti OR "consensus development".ti OR "consensus activity".ti OR "consensus activities".ti OR "consensus methodology".ti OR "consensus method*".ti OR exp *"Consensus Development"/ OR "RAND".ti OR (("Guidelines".ti,ab OR "guideline".ti,ab) ADJ2 ("consensus".ti,ab OR "delphi".ti,ab))) AND ("reporting".ti OR "quality of reporting".ti,ab OR "reporting quality".ti,ab OR "reporting qualities".ti,ab OR "selective reporting".ti,ab OR "poor reporting".ti,ab OR "poor reported".ti,ab OR "poorly reported".ti,ab OR *"Methodology"/ OR *"Writing"/ OR "research method".ti OR "research methods".ti OR "research method*".ti OR ("reporting" ADJ8 ("quality" OR "selective" OR "poor" OR "weak" OR "manner" OR "rigor" OR "improv*")).ti,ab))

**Cochrane**
https://www.cochranelibrary.com/advanced-search/search-manager

(("Delphi Technique" OR "Delphi Technique" OR "Delphi techniques" OR "Delphi method" OR "Delphi methods" OR "Delphi study" OR "Delphi studies" OR "Delphi survey" OR "Delphi surveys" OR "Delphi consensus" OR "Delphi based consensus" OR "Delphi questionnaire" OR "Delphi questionnaires" OR "Delphi research" OR "Delphi review" OR "Delphi reviews" OR "Delphi process" OR "Delphi processes" OR "Delphi based" OR "Delphi procedure" OR "Delphi procedures" OR "Delphi assessment" OR "Delphi assessments" OR "Delphi approach" OR "Delphi approaches" OR "Delphi panel" OR "Delphi panels" OR "Delphi round" OR "Delphi rounds" OR "Delphi analysis" OR "Delphi expert" OR "Delphi experts" OR "Delphi consultation" OR "Delphi methodology" OR "nominal group technique" OR "nominal group techniques" OR "nominal group" OR "nominal groups" OR "nominal grouping" OR "consensus recommendation" OR "consensus recommendations" OR "consensus development" OR "consensus activity" OR "consensus activities" OR "Consensus Development Conference" OR "Consensus Development" OR "Consensus methodology" OR "consensus method*" OR "RAND" OR (("Guidelines" OR "guideline") NEAR/2 ("consensus" OR "delphi"))) AND ("quality of reporting" OR "reporting quality" OR "reporting qualities" OR "selective reporting" OR "poor reporting" OR "poor reported" OR ("reporting" NEAR/5 ("quality" OR "selective" OR "poor" OR "weak" OR "manner" OR "rigor" OR "improv*")) OR "Data Accuracy" OR "Research Report standards" OR "quality assessment" OR "strengths" OR "strength" OR "weaknesses" OR "weakness" OR "research method" OR "research methods" OR "research method*")):ti,ab,kw

**Emcare**
http://ovidsp.ovid.com/ovidweb.cgi?T=JS&NEWS=n&CSC=Y&PAGE=main&D=emcr

((exp *"Delphi Study"/ OR "Delphi Technique".ti OR "Delphi techniques".ti OR "Delphi method".ti OR "Delphi methods".ti OR "Delphi study".ti OR "Delphi studies".ti OR "Delphi survey".ti OR "Delphi surveys".ti OR "Delphi consensus".ti OR "Delphi based consensus".ti OR "Delphi questionnaire".ti OR "Delphi questionnaires".ti OR "Delphi research".ti OR "Delphi review".ti OR "Delphi reviews".ti OR "Delphi process".ti OR "Delphi processes".ti OR "Delphi based".ti OR "Delphi procedure".ti OR "Delphi procedures".ti OR "Delphi assessment".ti OR "Delphi assessments".ti OR "Delphi approach".ti OR "Delphi approaches".ti OR "Delphi panel".ti OR "Delphi panels".ti OR "Delphi round".ti OR "Delphi rounds".ti OR "Delphi analysis".ti OR "Delphi expert".ti OR "Delphi experts".ti OR "Delphi consultation".ti OR "Delphi methodology".ti OR "nominal group technique".ti OR "nominal group techniques".ti OR "nominal group".ti OR "nominal groups".ti OR "nominal grouping".ti OR "consensus recommendation".ti OR "consensus recommendations".ti OR "consensus development".ti OR "consensus activity".ti OR "consensus activities".ti OR "consensus methodology".ti OR "consensus method*".ti OR exp *"Consensus Development"/ OR "RAND".ti OR (("Guidelines".ti,ab OR "guideline".ti,ab) ADJ2 ("consensus".ti,ab OR "delphi".ti,ab))) AND ("reporting".ti

OR "quality of reporting".ti,ab OR "reporting quality".ti,ab OR "reporting qualities".ti,ab OR "selective reporting".ti,ab OR "poor reporting".ti,ab OR "poor reported".ti,ab OR "poorly reported".ti,ab OR *"Methodology"/ OR *"Writing"/ OR "research method".ti OR "research methods".ti OR "research method*".ti OR ("reporting" ADJ8 ("quality" OR "selective" OR "poor" OR "weak" OR "manner" OR "rigor" OR "improv*")).ti,ab))

**Academic Search Premier**
http://search.ebscohost.com/login.aspx?authtype=ip,uid&profile=lumc&defaultdb=aph

((TI("Delphi Technique" OR "Delphi Technique" OR "Delphi techniques" OR "Delphi method" OR "Delphi methods" OR "Delphi study" OR "Delphi studies" OR "Delphi survey" OR "Delphi surveys" OR "Delphi consensus" OR "Delphi based consensus" OR "Delphi questionnaire" OR "Delphi questionnaires" OR "Delphi research" OR "Delphi review" OR "Delphi reviews" OR "Delphi process" OR "Delphi processes" OR "Delphi based" OR "Delphi procedure" OR "Delphi procedures" OR "Delphi assessment" OR "Delphi assessments" OR "Delphi approach" OR "Delphi approaches" OR "Delphi panel" OR "Delphi panels" OR "Delphi round" OR "Delphi rounds" OR "Delphi analysis" OR "Delphi expert" OR "Delphi experts" OR "Delphi consultation" OR "Delphi methodology" OR "nominal group technique" OR "nominal group techniques" OR "nominal group" OR "nominal groups" OR "nominal grouping" OR "consensus recommendation" OR "consensus recommendations" OR "consensus development" OR "consensus activity" OR "consensus activities" OR "Consensus Development Conference" OR "Consensus Development" OR "Consensus methodology" OR "consensus method*" OR "RAND" OR (("Guidelines" OR "guideline") N2 ("consensus" OR "delphi"))) OR KW("Delphi Technique" OR "Delphi Technique" OR "Delphi techniques" OR "Delphi method" OR "Delphi methods" OR "Delphi study" OR "Delphi studies" OR "Delphi survey" OR "Delphi surveys" OR "Delphi consensus" OR "Delphi based consensus" OR "Delphi questionnaire" OR "Delphi questionnaires" OR "Delphi research" OR "Delphi review" OR "Delphi reviews" OR "Delphi process" OR "Delphi processes" OR "Delphi based" OR "Delphi procedure" OR "Delphi procedures" OR "Delphi assessment" OR "Delphi assessments" OR "Delphi approach" OR "Delphi approaches" OR "Delphi panel" OR "Delphi panels" OR "Delphi round" OR "Delphi rounds" OR "Delphi analysis" OR "Delphi expert" OR "Delphi experts" OR "Delphi consultation" OR "Delphi methodology" OR "nominal group technique" OR "nominal group techniques" OR "nominal group" OR "nominal groups" OR "nominal grouping" OR "consensus recommendation" OR "consensus recommendations" OR "consensus development" OR "consensus activity" OR "consensus activities" OR "Consensus Development Conference" OR "Consensus Development" OR "Consensus methodology" OR "consensus method*" OR "RAND" OR (("Guidelines" OR "guideline") N2 ("consensus" OR "delphi")))) AND (TI("quality of reporting" OR "reporting quality" OR "reporting qualities" OR "selective reporting" OR "poor reporting" OR "poor reported" OR "reporting guideline" OR "reporting" OR ("reporting" AND ("quality" OR "selective" OR "poor" OR "weak" OR "manner" OR "rigor" OR "improv*")) OR "Data Accuracy" OR "quality assessment" OR "strengths" OR "strength" OR "weaknesses" OR "weakness" OR "research method" OR "research methods" OR "research method*") OR

KW("quality of reporting" OR "reporting quality" OR "reporting qualities" OR "selective reporting" OR "poor reporting" OR "poor reported" OR ("reporting" N5 ("quality" OR "selective" OR "poor" OR "weak" OR "manner" OR "rigor" OR "improv*")) OR "Data Accuracy" OR "Research Report standards" OR "quality assessment" OR "strengths" OR "strength" OR "weaknesses" OR "weakness" OR "research method" OR "research methods" OR "research method*") OR AB("quality of reporting" OR "reporting quality" OR "reporting qualities" OR "selective reporting" OR "poor reporting" OR "poor reported" OR ("reporting" N5 ("quality" OR "selective" OR "poor" OR "weak" OR "manner" OR "rigor" OR "improv*")) OR "Data Accuracy" OR "Research Report standards" OR "quality assessment" OR "strengths" OR "strength" OR "weaknesses" OR "weakness")))

**PsycINFO**
http://search.ebscohost.com/login.aspx?authtype=ip,uid&profile=lumc&defaultdb=psyh

((TI("Delphi Technique" OR "Delphi Technique" OR "Delphi techniques" OR "Delphi method" OR "Delphi methods" OR "Delphi study" OR "Delphi studies" OR "Delphi survey" OR "Delphi surveys" OR "Delphi consensus" OR "Delphi based consensus" OR "Delphi questionnaire" OR "Delphi questionnaires" OR "Delphi research" OR "Delphi review" OR "Delphi reviews" OR "Delphi process" OR "Delphi processes" OR "Delphi based" OR "Delphi procedure" OR "Delphi procedures" OR "Delphi assessment" OR "Delphi assessments" OR "Delphi approach" OR "Delphi approaches" OR "Delphi panel" OR "Delphi panels" OR "Delphi round" OR "Delphi rounds" OR "Delphi analysis" OR "Delphi expert" OR "Delphi experts" OR "Delphi consultation" OR "Delphi methodology" OR "nominal group technique" OR "nominal group techniques" OR "nominal group" OR "nominal groups" OR "nominal grouping" OR "consensus recommendation" OR "consensus recommendations" OR "consensus development" OR "consensus activity" OR "consensus activities" OR "Consensus Development Conference" OR "Consensus Development" OR "Consensus methodology" OR "consensus method*" OR "RAND" OR (("Guidelines" OR "guideline") N2 ("consensus" OR "delphi"))) OR AB("Delphi Technique" OR "Delphi Technique" OR "Delphi techniques" OR "Delphi method" OR "Delphi methods" OR "Delphi study" OR "Delphi studies" OR "Delphi survey" OR "Delphi surveys" OR "Delphi consensus" OR "Delphi based consensus" OR "Delphi questionnaire" OR "Delphi questionnaires" OR "Delphi research" OR "Delphi review" OR "Delphi reviews" OR "Delphi process" OR "Delphi processes" OR "Delphi based" OR "Delphi procedure" OR "Delphi procedures" OR "Delphi assessment" OR "Delphi assessments" OR "Delphi approach" OR "Delphi approaches" OR "Delphi panel" OR "Delphi panels" OR "Delphi round" OR "Delphi rounds" OR "Delphi analysis" OR "Delphi expert" OR "Delphi experts" OR "Delphi consultation" OR "Delphi methodology" OR "nominal group technique" OR "nominal group techniques" OR "nominal group" OR "nominal groups" OR "nominal grouping" OR "consensus recommendation" OR "consensus recommendations" OR "consensus development" OR "consensus activity" OR "consensus activities" OR "Consensus Development Conference" OR "Consensus Development" OR "Consensus methodology" OR "consensus method*" OR "RAND") OR KW("Delphi Technique" OR "Delphi

Technique" OR "Delphi techniques" OR "Delphi method" OR "Delphi methods" OR "Delphi study" OR "Delphi studies" OR "Delphi survey" OR "Delphi surveys" OR "Delphi consensus" OR "Delphi based consensus" OR "Delphi questionnaire" OR "Delphi questionnaires" OR "Delphi research" OR "Delphi review" OR "Delphi reviews" OR "Delphi process" OR "Delphi processes" OR "Delphi based" OR "Delphi procedure" OR "Delphi procedures" OR "Delphi assessment" OR "Delphi assessments" OR "Delphi approach" OR "Delphi approaches" OR "Delphi panel" OR "Delphi panels" OR "Delphi round" OR "Delphi rounds" OR "Delphi analysis" OR "Delphi expert" OR "Delphi experts" OR "Delphi consultation" OR "Delphi methodology" OR "nominal group technique" OR "nominal group techniques" OR "nominal group" OR "nominal groups" OR "nominal grouping" OR "consensus recommendation" OR "consensus recommendations" OR "consensus development" OR "consensus activity" OR "consensus activities" OR "Consensus Development Conference" OR "Consensus Development" OR "Consensus methodology" OR "consensus method*" OR "RAND" OR (("Guidelines" OR "guideline") N2 ("consensus" OR "delphi")))) AND (TI("quality of reporting" OR "reporting quality" OR "reporting qualities" OR "selective reporting" OR "poor reporting" OR "poor reported" OR "reporting guideline" OR "reporting" OR ("reporting" AND ("quality" OR "selective" OR "poor" OR "weak" OR "manner" OR "rigor" OR "improv*")) OR "Data Accuracy" OR "quality assessment" OR "strengths" OR "strength" OR "weaknesses" OR "weakness" OR "research method" OR "research methods" OR "research method*") OR KW("quality of reporting" OR "reporting quality" OR "reporting qualities" OR "selective reporting" OR "poor reporting" OR "poor reported" OR ("reporting" N5 ("quality" OR "selective" OR "poor" OR "weak" OR "manner" OR "rigor" OR "improv*")) OR "Data Accuracy" OR "Research Report standards" OR "quality assessment" OR "strengths" OR "strength" OR "weaknesses" OR "weakness" OR "research method" OR "research methods" OR "research method*") OR AB("quality of reporting" OR "reporting quality" OR "reporting qualities" OR "selective reporting" OR "poor reporting" OR "poor reported" OR ("reporting" N5 ("quality" OR "selective" OR "poor" OR "weak" OR "manner" OR "rigor" OR "improv*")) OR "Data Accuracy" OR "Research Report standards" OR "quality assessment" OR "strengths" OR "strength" OR "weaknesses" OR "weakness")))
