# Supplementary material for: Existing guidance on reporting of consensus methodology: a systematic review to inform ACCORD guideline development
Source: BMJ Open. 2022 Sep 8;12(9):e065154. doi: 10.1136/bmjopen-2022-065154 (PMC9462098; doi:10.1136/bmjopen-2022-065154)
Supplement: Supplementary data [file bmjopen-2022-065154supp002.pdf]

**Author, year**	
**Assessor**	

**Background**<br>1.1 Does the study suggest anything about how or if consensus papers should report the context or rationale for choosing a consensus method over other methods?	
**Background**<br>1.2 Does the study suggest anything about how/what or if consensus papers should report the objectives of the consensus exercise?	

**Methods**<br>2.1 Does the study suggest anything about how/what or if consensus papers should report regarding:<br>A literature search/strategy?	
**Methods**<br>2.2 Does the study the suggest anything about how/what or if consensus papers should report regarding:<br>Inclusion and exclusion criteria for the literature search?	
**Methods**<br>2.3 Does the study suggest anything of what or if consensus report should report on panel composition, n of participants, expertise, origin? Prespecified?	
**Methods**<br>2.4 Does the study suggest anything of how or if PPI (public patient involvement) activity should be reported?	
**Methods**<br>2.5 Does the study suggest anything about what or if consensus papers should report regarding panel recruitment strategies, invitations? Any level of detail specified?	
**Methods**<br>2.6 Does the study suggest how or if consensus papers should report the consensus criteria/threshold (or the level of agreement considered to reach consensus)?	
**Methods**<br>2.7 Does the study suggest how or if consensus papers should report how decision of approval of an item will be made?	
**Methods**	

2.8 Does the study suggest anything about what level of detail should be reported regarding the number of Delphi rounds or if this should be reported?	
**Methods**<br>2.9 Does the study suggest anything about what level of detail should be reported regarding the criteria used for defining the number of rounds? (why 2-3 or more e.g.) or if this should be reported?	
**Methods**<br>2.10 Does the study suggest anything about the details that should be reported regarding the time between rounds, if it should be prespecified or if this should be reported?	
**Methods**<br>2.11 Does the study suggest anything about details that should be reported of the names of the techniques of non-Delphi methods used to gather participants' inputs and reach consensus?	
**Methods**<br>2.12 Does the study suggest anything of what or in which detail should be reported regarding tool or electronic system used for Delphi? (If Delphi was used)? Or if this should be reported?	
**Methods**<br>2.13 Does the study suggest anything about how or in what level of detail the anonymity of participants (in Delphi or other methods) has to be reported? Or if this should be reported?	
**Methods**<br>2.14 Does the study suggest anything about how to report, and in what level of detail, the feedback for panellists (in Delphi rounds or other methods) process? Or if this should be reported?	
**Methods**<br>2.15 Does the study suggest anything about how or if data synthesis/analysis should be reported (from any consensus method used and how this was calculated statistically) and in what level of detail?	
**Methods**<br>2.16 Does the study suggest anything about how or if piloting should be reported and in what level of detail (e.g. understanding of consensus items, platforms used, tools used)?	
**Methods**	

2.17 Does the study suggest anything about how or if the role of Steering Committee members should be reported?	
**Methods**<br>2.18 Does the study suggest anything on what or if should be described regarding COI or funding?	
**Methods**<br>2.19 Does the study suggest anything on what should be described of how is dealt with COI of panellist (not allowed to vote when there is COI)? Or if this should be described	

**Results**<br>3.1 Does the study suggest anything on how to report the initial evidence search (presentation of results of the literature review)?	
**Results**<br>3.2 Does the study suggest anything on how to report n of studies found?	
**Results**<br>3.3 Does the study recommend which detail should be used when reporting panellists drop-outs (numbers and reasons)? Or if this should be reported?	
**Results**<br>3.4 Does the study suggest how or if approval rates per item shared with respondents for each round should be reported in the Results section?	
**Results**<br>3.5 Does the study suggest anything about in which detail the items that have been dropped should be reported? (reasons e.g.) Or if this should be reported?	
**Results**<br>3.6 Does the study make any recommendation on how to report the collection, synthesis and use of comments from panellists? Or if this should be reported?	
**Results**<br>3.7 Does the study suggest regarding how the final list of items (for clinical guideline or reporting guideline) should be reported? Or if this should be reported?	

**Discussion**<br>4.1 Does the paper suggest anything about reporting the limitations and strengths of the study and how? Or if this should be reported?	
**Discussion**	

4.2 Does the paper suggest anything about what or in which detail the applicability generalisability, and reproducibility of the study should be reported? Or if this should be reported?	

**5.1 Any other item proposed by the paper that is not captured in other columns?**	
**5.2 Any other item not proposed by the paper, but you think that could be added (not fitting the categories above)?**	

**Examples of text with well reported methods/results (for E&E document) - write NA if none was cited or found by you**	
**Additional comments from assessor**	
