# Supplementary material for: Existing guidance on reporting of consensus methodology: a systematic review to inform ACCORD guideline development
Source: BMJ Open. 2022 Sep 8;12(9):e065154. doi: 10.1136/bmjopen-2022-065154 (PMC9462098; doi:10.1136/bmjopen-2022-065154)
Supplement: Supplementary data [file bmjopen-2022-065154supp003.pdf]

Data on reporting quality (*recommendations in italics)*

| Study | What is stated regarding reporting quality? |
|---|---|
| Banno 2019[32] | • "The reporting quality of the Delphi technique in reporting guidelines is unknown even though the use of the Delphi technique was recommended in the guidance for reporting guidelines." (Note: This is a protocol for the systematic review of 2020.)<br>*4 quality score items are summarised of Delphi methods used in reporting guidelines*. |
| Banno 2020[16] | • "Reproducible criteria of participants, number of rounds, criteria for dropping items, and stopping criteria other than rounds were found for 87%, 97%, 69%, and 13%, respectively of reporting guidelines developed with the Delphi method. The total score of reporting quality was 2 or more in 94% of reporting guidelines using the Delphi method."<br>*4 quality score items are summarised of Delphi methods used in reporting guidelines*. |
| Boulkedid 2011[17] | • "Study reports did not consistently provide details that are important for interpreting the results. For example, only 39% of studies reported that individual feedback was given between rounds and the method used to define a consensus was specified in only 77% studies. Moreover, response rates for all rounds were reported in only 31% of studies. Information on both points is needed to evaluate the validity and credibility of the results. If the Delphi method is incompletely described this may affect the overall quality of the final consensus and the selected indicators are unlikely to gain the level of credibility needed for adoption I clinical practice."<br>• "The Delphi procedure is valuable for achieving a consensus about issues where none existed previously. However, our findings indicate a need for improving the use and reporting of this technique."<br>*Table 5 provides recommendations for reporting the Delphi procedure.* |
| Chan 2019[20] | • "This lack of clear definition has led to considerable confusion and substantial variation in the quality of reporting of Delphi studies"<br>• "One-third of medical education Delphi studies failed to report that a literature review on the topic of interest had been conducted , and over half failed to report key aspects such as what background information was provided to participants; the response rate for each round; what formal feedback of group rating was shared between rounds; a statement that anonymity was maintained; and a clear definition of consensus."<br>• "Lack of clarity in the report in the reporting of procedures and methodological choices associated with the modified Delphi studies can prevent readers from effectively appraising and interpreting findings."<br>• "Methodological rigor and transparent reporting are essential to assure readers that the consensus results are applicable to their environment, and to translate expert opinion into practice."<br>*Box 1 provides recommendations to improve reporting.* |
| Diamond 2014[18] | • "Definitions of consensus vary widely and are poorly reported. Improved criteria for reporting of methods of Delphi studies are required."<br>• "Methodologic criteria are proposed for the reporting of Delphi studies."<br>• "Despite the fact that the most Delphi studies in our cohort had consensus as their aim, in only a minority of the Delphi studies reviewed was consensus defined with a specific criterion. Furthermore, this criterion was the reason for termination of the Delphi process, usually on the basis of an *a priori* definition."<br>• "We believe that there is a need to improve the reporting of Delphi studies, along the lines of a CONSORT-like guideline, as is used for randomized controlled trials."<br>*Methodologic criteria are proposed for the reporting of Delphi studies.* |



van Zuuren EJ, *et al. BMJ Open* 2022; 12:e065154. doi: 10.1136/bmjopen-2022-065154

| | |
|---|---|
| Gattrell 2019[29] | "At present there are a lack of standard, validated reporting guidelines for publications reporting the results of Delphi panel studies."<br><br>Quality assessment: Methodological quality<br>• The type of Delphi technique used, or the modifications to the method, was not outlined in all publications (included in 62/90 publications; 68.9%).<br>• Just over half of all publications stated that there was some diversity amongst participants and clearly outlined the methods for the selection of panellists.<br>• Agreement and consensus thresholds should be defined prior to study commencement, but in 40% of publications it was unclear, or not stated whether these thresholds were predefined.<br>• Anonymised responses are typically conveyed back to the group after each round, but this was clearly reported in less than half (38.9%) of publications.<br><br>Quality assessment: Reporting quality and transparency (Figure 3b).<br>• The funding source was not clearly disclosed in over a third of publications, and almost twice as many publications did not clearly disclose the funder's role.<br>• Conflicts of interest were clearly described in most publications (included in 79/90 publications; 87.8%).<br>• Clear disclosure of external support was not evident in the majority of the publications. |
| Grant 2018[24] | • "Specifying the analysis procedure for consensus is therefore a critical consideration when designing consensus-oriented Delphi processes in health research."<br>• "Without prespecifying their analysis procedures in a study registry, health researchers conducting consensus-oriented Delphi processes can mine for and selectively report the most desirable set of items reaching consensus and even present the reported analysis as the only one conducted. Undisclosed flexibility in data collection, analysis, and reporting is a growing concern in empirical research."<br>• "Without preregistering and reporting all of the attempted analysis procedures and when they were attempted, the extent and impact of researchers trying different analysis procedures is nearly impossible for peer reviewers, editors, and consumers of Delphi research to assess."<br>• "To be completely registered, the preanalysis plan should precisely describe the essential elements of the analysis procedure for determining consensus (see Box 2)."<br>• "Researchers should use existing guidance on reporting completed Delphi processes to provide sufficient information for comparing the final article to the registered preanalysis plan [1,12,42], with particular attention in the final article to any changes from the preanalysis plan in the items, rating criteria, analytic procedure (measure and threshold), and data and participants included in the analysis."<br>*Box 2 provides a minimum set of items to include in prospectively registered preanalysis plans for consensus-oriented Delphi processes.* |
| Hasson 2017[27] | • "Figure 1 Areas for reporting on the Delphi survey technique."<br>• "In Delphi surveys there exists no consistent method for reporting findings (Schmidt 1997) and a review of the literature showed that a number of approaches have been used."<br>• "The following diagram attempts to outline those sections that researchers should report upon when using the Delphi. This will help readers to judge the reliability of the method and the results obtained."<br>*Followed by a checklist of issues, which could be used by researchers.* |

| | |
|---|---|
| Humphrey-Murto 2017[21] | • "The authors set out to describe the use of consensus methods in medical education research and to assess the reporting quality of these methods and results."<br>• "Improved criteria for reporting are needed."<br>• "Our findings suggest that the reporting quality and standardization of consensus methods in medical education research varies greatly. The following areas appeared particularly problematic and were often left out or poorly described in the articles we reviewed: conducting a literature review to inform the consensus method; providing background information to participants; reporting the number of participants after each round; describing the level of anonymity used in the study; providing participants with feedback of group ratings; and articulating the definition of consensus used in the study."<br>*Recommendations for improvements in these areas are provided in Discussion.* |
| Humphrey-Murto 2017[28] | • "Consensus group methods are widely used in research to identify and measure areas where incomplete evidence exists for decision-making. Despite their widespread use, these methods are often inconsistently used and reported."<br>• "This paper and associated Guide aim to describe these methods and to highlight common weaknesses in methodology and reporting."<br>• "The AMEE Guide describes these methods to provide a "how to" approach, highlight common weaknesses in methodology and reporting, and outline recommendations for reporting future consensus based studies."<br>• "Four recent reviews using the Delphi in health care and policy-related research have systematically explored deficiencies in the use and reporting of consensus group methods. Collectively, these studies have noted deficiencies regarding: information provided to the participants at the start of Delphi, reporting response rates, feedback to participants, level of anonymity, outcomes after each round and the definition of consensus."<br>*This guide provides recommendations for improvement of reporting.* |
| Humphrey-Murto 2019[25] | • "Studies using the Delphi for selecting performance indicators for healthcare, for medical and nursing education, or for determining outcomes to measure in clinical trials, often fail to adequately report sufficient methodological detail. Examples include poor reporting of background information provided to participants, response rates for all rounds, level of anonymity, formal feedback between rounds, and the definition of consensus."<br>*OMERACT Delphi consensus checklist is provided in Figure 1.* |
| Jünger 2017[12] | • "Substantial variation was found concerning the quality of the study conduct and the transparency of reporting of Delphi studies used for the development of best practice guidance in palliative care. Since credibility of the resulting recommendations depends on the rigorous use of the Delphi technique, there is a need for consistency and quality both in the conduct and reporting of studies. To allow a critical appraisal of the methodology and the resulting guidance, a reporting standard for Conducting and Reporting of DElphi Studies (CREDES) is proposed."<br>Study adds in Box 3*"Recommendations for the Conducting and REporting of DElphi Studies (CREDES)."* |
| Ng 2018[30] | • "Given the variance in the use of Delphi method, reporting guidelines could help improve reporting of this research, and thereby allow readers to be aware of the accuracy of data and conclusions."<br>• "We anticipate the implementation of this will promote transparent and accurate reporting of research using Delphi method for obtaining quantitative data."<br>*A set of reporting guidelines is proposed.* |
| Niederberger 2020[26] | • "Significant weaknesses exist in the quality of the reporting." |

| | |
|---|---|
| | • "Criteria for evaluating the quality of their execution and reporting also appear to be necessary."<br>• "A specific definition of the underlying Delphi technique was found in 61% (ID11) and 88.2% (ID4) of the Delphi articles investigated."<br>• "Most of the Delphi studies analyzed in the reviews reported on the number of participating experts. The rates for the initial round were between 84% (ID6) and 100% (ID12). Four of the reviews investigated whether the number of experts was stated for each round (ID4, ID7, ID11, ID12). In one review based on 10 Delphi studies from health sciences (ID7), the authors discovered that the number of experts per round was stated in all articles. A review of 48 studies in a medical context indicated that the number of invited experts was stated less frequently with each round (ID6). Seven of the 12 reviews investigated whether the backgrounds of the experts had been reported, what kind of expertise they possessed, and the criteria according to which they were selected (ID1, ID3, ID4, ID6, ID9, ID11, ID12). One review of Delphi techniques in a health context determined that the criteria for selecting the experts was reproduced in 65 of 100 articles (65%) (ID3) included in that particular review. In other reviews with a more specific focus, such as on health care, palliative medicine, or health promotion, the rates were higher at 69% (ID11), 70% (ID9) and 79% (ID1), respectively. Based on the results of the reviews, the criteria by which the experts were selected and approached was not always clear. In one review of 100 studies from the care sector, the proportion of articles with unclear selection criteria was 11.2% (ID4), while the proportion was 93.3% in a review of 15 studies from the clinical sector (ID12)."<br>• "Seven of the 12 reviews determined whether and when consensus was defined in the Delphi studies (ID1, ID3, ID4, ID6, ID9, ID11, ID12). The number of studies in which consensus was defined in the article was between 73.5% (ID3) and 83.3% (ID9) in the reviews."<br>• "The authors of seven reviews investigated whether the number of Delphi rounds was published (ID1, ID3, ID4, ID6, ID9, ID11, ID12). The number of Delphi rounds was stated in most of the Delphi studies (e.g., ID1 82.5%, ID4 91%, ID6 100%, ID9 49.3%, ID12 93.3%). Six of the reviews included a report of the generation of the questionnaire (ID1, ID4, ID6, ID9, ID11, ID12). They demonstrated that up to 96.3% of the investigated articles reported on how the items for the questionnaire were developed (ID1). In contrast, this rate stood at 33.3% in the review of palliative care articles (ID9). The authors of two reviews investigated the question of how the items were changed during the Delphi process based on the judgments submitted by the experts (ID3, ID12). In one of the reviews, the authors indicated that 59% of the analyzed articles had defined criteria for dropping items (ID3). In another review, the authors stated that all of the investigated Delphi studies included a report of "what was asked in each round" (ID12, p. 2). The authors of the reviews reported about the feedback in most of the Delphi studies (ID11 67.9%, ID12 93.3%). The information provided about the response rate per Delphi round was less (ID1 and ID4 39%). According to the results of the reviews, around half of the studies did not provide information about the feedback design between the Delphi rounds (ID1 40%, ID4 55.1%, ID6 37.7% ID12 40%). According to the authors of the review on health promotion, the process—from formulating the issue being investigated through to the development of the questionnaire—was in general similar to a "black box," and the methodological quality of the survey instrument was almost impossible to evaluate using the published information (ID11, p. 318)."<br>• "Our results also indicate deficits both in carrying out and also reporting Delphi techniques." |

| | |
|---|---|
| | • "The findings in the reviews we analyzed indicated that there is no uniform process for carrying out and reporting Delphi techniques." |
| Paré 2013[22] | • "Thirty-one percent of the articles in our sample provided a detailed description of the expert recruitment and selection process, 43% provided only limited details, and 26% did not provide any details."<br>• "All of the articles in our database (n = 42) specified the criteria that were used to select the panel of experts. Position is by far the most used criteria (71%), followed by relevant professional experience (57%), geographic location (7%), and education level (5%)."<br>• "38% of the studies provided detailed information about the participating experts [e.g., 44], 40% provided minimal information [e.g., 2], and 22% did not provide any description".<br>• "The anonymity of the experts was reported in virtually all of the studies (95%) in our sample."<br>• "Only 29% of all of the studies reported the response rate to the initial request for participation."<br>• "35 studies (83%)reported the size of the panels. The majority of the studies (n = 21) reported a panel size between 7 and 30, only one study reported a size of 6 or less, and 13 studies reported panel sizes above 30. Nine studies (19%) examined multiple panels of experts."<br>• "Only 17% of these Delphi studies reported that a pretest of the instruments had been conducted."<br>• "24 studies out of 27 (89%) reported the brainstorming instructions that were sent to the experts."<br>• "Only 8 studies (30%) reported the use of this recommendation. (i.e. Have the experts comment and validate the consolidated list)."<br>• "The vast majority of the studies (85%) reported the final number of items at the end of phase 1."<br>• "Among the 25 studies that did not include this phase (i.e. narrowing down phase), 68% explicitly justified this choice (e.g., the number of items at the end of phase 1 are equal or less than 20 as suggested by Schmidt."<br>• "All 17 studies clearly described the narrowing down instructions that were given to the experts."<br>• "65% of the studies clearly specified their item selection rule."<br>• "Most of the studies (82%) reported the final number of items at the end of the second phase."<br>• "All 42 articles described clearly the ranking instructions that were provided to the experts."<br>• "Almost all of the studies (95%) in our sample reported the statistics that were used for data analysis."<br>• "31% of the studies in our database specified a clear stopping rule."<br>• "Only 15 studies (36%) reported the final consensus rate."<br>• "29 of the 42 studies had multiple rounds of ranking. Of these, the feedback that was provided to the experts in between the rounds included the mean ranks of items (69% of studies), an interpretation of the Kendall's W coefficient (3%), the expert's prior responses (59%), and the comments made by the other experts (38%)."<br>*Recommendations regarding what to report are provided throughout the Results section as well as in the Discussion.* |
| Resemann 2018[31] | • "Reporting of the Delphi method was critiqued against the AGREE Reporting Checklist."<br>• "All studies reported consensus results. The majority (8/11 [73%]) used a two-stage modified Delphi method, while the remainder used a classic three-stage process. Literature searches guided the development of statements for Delphi panel review in the majority of studies, but only 2/11 (18%) conducted |

| | |
|---|---|
| | systematic literature reviews and merely 6/11 (55%) of studies reported the number of statements assessed. Furthermore, 7/11 (64%) did not report collecting panellist feedback to inform subsequent Delphi stages, 5/11 (45%) of studies did not describe the rating scales used, and 2/11 (18%) omitted reporting the level of consensus reached"<br>• "There is a need for improved reporting of Delphi methods". |
| Waggoner 2016[23] | • "Despite the widespread utility of consensus methods and the variety of approaches available, there is a lack of guidelines for conducting such studies. This lack of stringency in guidelines for conducting consensus studies has led to variability not only in reporting results but in conducting the studies themselves."<br>• "Many studies describe their methods for collecting data and that they did have a benchmark that would point to a consensus, but a lack of a description of the analytical techniques is apparent in many studies."<br>• "In addition to the lack of descriptive techniques in these articles, there is a wide range of criteria that points to consensus. How these particular benchmarks are determined is also not a topic in many of the studies. Given the lack of current research, we believe that the methodology used I subsequent studies should be described more thoroughly in the manuscript."<br>• "We set out to determine best practices for conducting such research as well as reporting on results in the hopes that future studies are more reliable and valid."<br>*This article provides guidance for reporting of various consensus methods.* |
| Wang 2015[19] | • "Adoption of reporting guidelines is associated with improved reporting quality of research."<br>• "For example, 28 % of the included guidelines reported no information about consensus, and 57 % were silent about how the feedback after consensus was dealt with."<br>• "In addition to the methodology, only 31 % reported formal consensus method."<br>• "Among guidelines developed through consensus, 30 (50 %) reported group member identification and 31 (52 %) reported member recruitment. Of those who identified members, 27 (45 %) reported specialties of experts, 20 (32 %) described information of members, such as names and institutions, and four (7 %) gave the selection criteria. For those who recruited members, even (12 %) described the recruit methods, for instance, through e-mail, study co-chairs, or group decision. In guidelines developed by a working group, 22 (37 %) reported the number of experts participating in guideline development (median 32, range 3–115). Eleven (18 %) guidelines reported the endpoint of consensus process, which were all terminated after a fixed number of rounds (Table 2). In addition, the inclusion criteria of items were given in eight (13 %) guidelines. For example, items meeting the median score of eight or higher in the final round were included."<br>• "11 (18 %) described the pilot methods, seven (12 %) described the feedback information requirement and five (8 %) gave the methods for feedback collection."<br>• "More than 30 % of the reporting guidelines did not report consensus. For those who did, details of consensus methods were poorly reported."<br>• "Consensus methods should be supported by developers, and the reporting of the methods should be improved."<br>*Recommendations for Consensus methods are provided, but more about improvement of applying and reporting using all other reporting guidelines, but some items are applicable for consensus methodology as well (e.g. reporting COI and funding.* |
