# Supplementary material for: Existing guidance on reporting of consensus methodology: a systematic review to inform ACCORD guideline development
Source: BMJ Open. 2022 Sep 8;12(9):e065154. doi: 10.1136/bmjopen-2022-065154 (PMC9462098; doi:10.1136/bmjopen-2022-065154)
Supplement: Supplementary data [file bmjopen-2022-065154supp004.pdf]

**Background**<br>1.1 Does the study suggest anything about how or if consensus papers should report the context or rationale for choosing a consensus method over other methods?	1) Research problem clearly defined and topic and method justification should be reported [Hasson 2000, Figure 1 and page 1013]<br><br>2) Selection of one consensus method over another should be evident if the purpose is clearly stated. [Humphrey-Murto 2017 Med Teach page 16]<br><br>3) What is the rationale for selecting the Delphi procedure? [Humphrey-Murto 2019, Figure 1]<br><br>4) The choice of the Delphi technique as a method of systematically collating expert consultation and building consensus needs to be well justified. A rationale for the choice of the Delphi technique as the most suitable method needs to be provided [Jünger 2017, Box 3, items 1 and 8]
**Background**<br>1.2 Does the study suggest anything about how/what or if consensus papers should report the objectives of the consensus exercise?	1) Define the study objective [Boulkedid 2011, Table 5 page 7]<br><br>2) Define the purpose of the study [Chan 2019, Box 1]<br><br>3) Is the objective of the Delphi study to present results (eg, a list or statement) reflecting the consensus of the group, or does the study aim to merely quantify the level of agreement? [Diamond 2014, Table 6 and page 403] If the aim of the Delphi study is to elicit consensus, then a clear definition for what constitutes consensus should be provided a priori together with threshold values that specify when consensus is reached. If the investigators plan to only quantify the degree of consensus, but not have consensus as a criterion to stop the Delphi study, this should also be explicitly stated [Diamond 2014, page 406]<br><br>4) Research problem clearly defined and topic and method justification should be reported [Hasson 2020, Figure 1 and page 1013]<br><br>5) Authors must provide a clear purpose for their study or line of inquiry [Humphrey-Murto 2017 Med Teach, page 16]<br><br>6) The purpose of the study should be clearly defined and demonstrate the appropriateness of the use of the Delphi technique as a method to achieve the research aim. A rationale for the choice of the Delphi technique as the most suitable method needs to be provided [Jünger 2017, item 8]

	The Delphi technique is a flexible method and can be adjusted to the respective research aims and purposes. Any modifications should be justified by a rationale and be applied systematically and rigorously" [Jünger 2017, item 2]

**Methods**<br>2.1 Does the study the suggest anything about how/what or if consensus papers should report regarding:<br>A literature search/strategy?	1) Describe the selection and preparation of the scientific evidence for the participants [Chan 2019, Box 1]<br><br>2) A literature review should be reported [Hasson 2000, Figure 1]<br><br>3) "We suggest that this important step must be described", but they don't say how. [Humphrey-Murto 2017 AMA, page 1493 and 1496 Partially]<br><br>4) Describe the selection and preparation of the scientific evidence for the participants [Humphrey-Murto 2017 Med Teach, page 16]<br><br>5) Only implying it should happen and be reported [Resemann 2018]
**Methods**<br>2.2 Does study the suggest anything about how/what or if consensus papers should report regarding:<br>Inclusion and exclusion criteria for the literature search?	1) Clear definition of the selection criteria and/or the definition used in the Delphi questionnaire; criteria for selection should be reported [Boulkedid 2011, Table 5, Appendix S1 item 2]<br><br>2) Describe how items were selected for inclusion in questionnaire, in sufficient detail [Chan 2019, Box 1]<br><br>3) Clear selection criteria should be prespecified [Paré 2013 page 210]
**Methods**<br>2.3 Does the study suggest anything of what or if consensus report should report on panel composition, n of participants, expertise, origin? Prespecified?	1) The method used to select participants is stated. Number and type of participant subgroups (eg, patients, generalists and experts) are needed [Banno 2019, page 2 item 1]<br><br>2) The method to include and exclude participants was described. The number and type of participant subgroups (e.g., patients, generalists, and experts) were essential to record [Banno 2020, page 52 item 1]<br><br>3) How the experts were chosen (e.g., willingness to participate, expertise, or membership in an organization); Composition and characteristics of the panel, number of participants (diagram of participant flow); number invited, how they were chosen, whether they were described (age, sex, specialty), years of experience, single or from multiple

	specialties, inclusion of multiple stakeholders, types of stakeholders [Boulkedid 2011, page 2, Table 5, Appendix S1 item 9-15]<br><br>4) Describe how participants were selected and their qualifications. Include description of facilitator credentials [Chan 2019, Box 1]<br><br>5) Were criteria for participants reproducible? How will participants be selected or excluded? [Diamond 2014, Table 5 and 6]<br><br>6) Was there heterogeneity in panel membership and is the method for selection of experts clearly defined [Gattrell 2019, Table 1]<br><br>7) Expert selection process and characteristics should be reported in detail [Hasson 2000, page 1009, 1013]<br><br>8) How many participants were involved? We noted that the type of expertise required of participants was usually not clearly described [Humphrey-Murto 2017 AMA, page 1493 and 1494]<br><br>9) Describe how the participants were selected and their qualifications: if the NGT or RAND/UCLA is used, describe facilitator's credentials. Whatever the makeup of the expert panel, the authors must provide a rationale and justify their choices [Humphrey-Murto 2017 Med Teach]<br><br>10) How many stakeholder/participant groups will be involved in each step? Provide a rationale for inclusion or exclusion and define the stakeholder groups [Humphrey-Murto 2019, Fig 4]<br><br>11) Criteria for the selection of experts and transparent information on recruitment of the expert panel, sociodemographic details including information on expertise regarding the topic in question, (non)response and response rates over the ongoing iterations should be reported [Jünger 2017, Box 3 9]<br><br>12) Describing expert panel selection with eligibility criteria and including conflicts of interest [Ng 2018]<br><br>13) The number of experts in each round should be stated. The backgrounds of the experts should be reported, what kind of expertise they possessed, and the criteria according to which they were selected [Niederberger 2020, page 4]

	14) Explicit procedures for expert selection; Clear selection criteria; Clear selection criteria should be prespecified and may include the candidates' years of related experience, or tenure in a position that is relevant to the subject under study Report the response rate to the initial call for participation; provide detailed information about the participating experts (profile) to better allow judgments about their credibility [Paré 2013, page 210, Table 3]<br><br>15) Explain how groups were chosen. Consensus Development Panels: Panel composition: the panel should be made up of experts in the field; the publication should report on how they were chosen and why; [Waggoner 2016, page 665, 667]<br><br>16) Implied by mentioning that detailed information on participants was lacking in some reporting guidelines. Page 5 Report specialties of experts, names and institutions, the selection criteria [Wang 2015]
**Methods**<br>2.4 Does the study suggest anything of how or if PPI (public patient involvement) activity should be reported	No data
**Methods**<br>2.5 Does the study suggest anything about what or if consensus papers should report regarding panel recruitment strategies, invitations? Any level of detail specified?	1) The use of specific methods to encourage the experts to respond (e.g., stamped addressed envelope for returning the questionnaire and financial compensation) [page 2] and recommendation to report whether special techniques were used to invite participants [Boulkedid 2011, Appendix S1 item 21]<br><br>2) Criteria for the selection of experts and transparent information on recruitment of the expert panel, socio- demographic details including information on expertise regarding the topic in question, (non)response and response rates over the ongoing iterations should be reported" [Jünger 2017, Box 3, 9]<br><br>3) provide a detailed description of the expert recruitment and selection process [Paré 2013, page 215 first bullet on the right]<br><br>4) method of obtaining participants should be described [Waggoner 2016, page 667]
**Methods**	1) The method used to define a consensus among panel members; , whether the percentage of agreement was determined; Whether a cut-off (e.g., median value) was used to select indicators [page 2] Consensus definition at each

2.6 Does the study suggest how or if consensus papers should report the consensus criteria/threshold (or the level of agreement considered to reach consensus)?	round [page 7, Appendix item 28] how was consensus obtained [page 7, Appendix item 28] definition of consensus should be reported [Boulkedid 2011, table 5]<br><br>2) Clearly describe how consensus was defined [Chan 2019, Box 1]<br><br>3) Need to define criteria for consensus and to document the degree of agreement together with the results of the Delphi process. Should be defined a priori. [Diamond 2014, page 404 and table 6]<br><br>4) Was the agreement/consensus threshold predefined? [Gattrell 2019, table 1]<br><br>5) Box 2 Specific threshold for the chosen measure (e.g., median of at least 7 on a nine-point scale and an interquartile range of less than 2) [Grant 2018, p 97]<br><br>6) Determine the criteria and the meaning of `consensus' in relation to the studies [Hasson 2020, page 1013]<br><br>7) No. They do state that "articulating the definition of consensus used" was identified as "particularly problematic and were often left out or poorly described", and that "the most concerning issue we identified was that consensus was often not defined a priori. Only 43.2% of the articles we reviewed reported their definition of consensus at the start of the study." But they do not suggest how to report. [Humphrey-Murto 2017 AMA]<br><br>8) Clearly describe how consensus was defined [Humphrey-Murto 2017 Med Teach, page 18]<br><br>9) suggests definition of consensus should be reported [Humphrey-Murto 2019, table 1, also fig 1 and page 1044]<br><br>10) Definition of consensus. Unless not reasonable due to the explorative nature of the study, an a priori criterion for consensus should be defined. This includes a clear and transparent guide for action on (a) how to proceed with certain items or topics in the next survey round, (b) the required threshold to terminate the Delphi process and (c) procedures to be followed when consensus is (not) reached after one or more iterations". Definition and attainment of consensus. It needs to be comprehensible to the reader how consensus was achieved throughout the process, including strategies to deal with non-consensus". "If an a priori definition of consensus is not realistic due to the explorative nature of the study, it should be identified and established by the research team in the course of the process." [Jünger 2017, item 12]

	11) How was consensus defined and measured? What role did the stability of the answers play? [Niederberger 2020, Table 2] Whether and when consensus was defined in the Delphi studies. Was consensus defined a priori in advance of development of the questionnaire. [Niederberger 2020, Table 5] How was consensus measured, e.g. percentage agreement, units of central tendency (especially median) or a combination of percent agreement within a certain range and for a certain threshold. [Niederberger 2020, page 6]<br><br>12) NGT explain criteria used to determine how and when a consensus was met Consensus Development Panels: Explain what constituted consensus and how this was assessed. [Waggoner 2016, page 665] Delphi Explain what constituted consensus and how this was assessed. [Waggoner 2016, page 667]<br><br>13) The endpoint of consensus [Wang 2015, page 5]
**Methods**<br>2.7 Does the study suggest how or if consensus papers should report how decision of approval of an item will be made?	1) Whether the percentage of agreement was determined [page 2] We recorded the method used to define a consensus among panel members, whether the percentage of agreement was determined, and whether a cut-off (e.g., median value) was used to select [Boulkedid 2011, Appendix S1 item 16 (technique method)]<br><br>2) Reporting on each round separately illustrates clearly the array of themes generated in round one and gives an indication of the strength of support for each round. The presentations of findings are important and findings from subsequent rounds should be reported in a summarized format to indicate the relative standing of each of the opinions. [Hasson 2020, page 1013]<br><br>3) (Non)response and response rates over the ongoing iterations should be reported [Jünger 2017, item 9]
**Methods**<br>2.8 Does the study suggest anything about what level of detail should be reported regarding the number of Delphi rounds or if this should be reported?	1) Was the number of rounds to be performed stated (not how it should be reported, but implies it should be) [Banno 2019, page 2 under item 2]<br><br>2) Was the number of rounds to be performed stated? [Banno 2020, 3.4, table 3]<br><br>3) Describe the number of rounds planned [Chan 2019, Box 1]

	4) Specify a maximum number of rounds [page 404] what was the reason to stop the delphi [Diamond 2014, table 3] What criteria will be used to determine to stop the Delphi process or will the Delphi be run for a specific number of rounds only [Diamond 2014, table 6, table 1 item 2]<br><br>5) number and outline per round should be reported also page 1013 [Hasson 2020, fig 1]<br><br>6) Describe the number of rounds planned and/or criteria for terminating the process [Humphrey-Murto 2017 Med Teach, page 17]<br><br>7) Only implying that x number of rounds are necessary [Humphrey-Murto 2017 AMA]<br><br>8) The methods employed need to be comprehensible; information about the number and design of survey rounds, [Jünger 2017, Box 3 item 10]<br><br>9) Not specifically under item 4 in table 2 report of the specific process used? How many rounds were used in the Delphi technique [Niederberger 2020]<br><br>10) If a study goes beyond the agreed number of rounds (review suggests 2 rounds are required), this should be explained [Waggoner 2016, page 667]
**Methods**<br>2.9 Does the study suggest anything about what level of detail should be reported regarding the criteria used for defining the number of rounds? (why 2-3 or more e.g.) or if this should be reported?	1) Implied in Banno 2020 The prespecified criteria for stopping the Delphi process, other than a statement of the number of rounds, were clarified [Banno 2020]<br><br>2) Describe the number of rounds planned and criteria for terminating the process [Chan 2019, Box 1]<br><br>3) Describe the number of rounds planned and/or criteria for terminating the process [Humphrey-Murto 2017 Med Teach, page 17]<br><br>4) They, imply that the number of rounds is an important thing to report -- but they do not state this as a suggestion.[Humphrey-Murto 2017 AMA]<br><br>5) Will the number of rounds be decided a priori? If not determined a priori, what are the criteria for terminating the process? [Humphrey-Murto 2019, Fig 1]

	6) What was the rationale for the number of rounds; when was the number of rounds defined [Niederberger 2020, page 6]<br>7) Table 3 Report the stopping [Paré 2013]<br>8) For delphi: if a study goes beyond two rounds, explain reason for doing so; [Waggoner 2016, page 667]
**Methods**<br>2.10 Does the study suggest anything about the details that should be reported regarding the time between rounds, if this should be prespecified in advance, or if this should be reported?	1) The time taken to complete the Delphi procedure was recorded [Boulkedid 2011, page 2]
**Methods**<br>2.11 Does the study suggest anything about details that should be reported of the names of the techniques of non-Delphi methods used to gather participants' inputs and reach consensus ?	1) Whether the meeting was held before, after, or between Delphi rounds and what the participants did during the meeting [Boulkedid 2011, page 2]
**Methods**<br>2.12 Does the study suggest anything of what or in which detail	1) What software will be used to administer the Delphi? [Humphrey-Murto 2019, fig 1]

should be reported regarding tool or electronic system used for Delphi? (If Delphi was used)? Or if this should be reported?	
**Methods**<br>2.13 Does the study suggest anything about how or in what level of detail the anonymity of participants (in Delphi or other methods) has to be reported? Or if this should be reported?	1) No, only that it is a limitation of this study that the quality score did not include that. So actually they feel it should be reported how anonymity was maintained [Banno 2020]<br><br>2) Describe how anonymity was defined [Chan 2019, Box 1]<br><br>3) Were responses anonymized [Gattrell 2019, table 1]<br><br>4) It suggests that conducting anonymous iterative mail or e-mail questionnaire rounds is one of the steps [p 1491]. While the authors may have assumed that readers would understand that anonymity was part of their study design, we suggest that they state this, given the variability in approaches that have been labelled as modified consensus methods. [Humphrey-Murto 2017 AMA, page 1497]<br><br>5) Describe how anonymity was maintained. Authors must clearly state how this was accomplished. It is achieved through the use of mail outs in Delphi and RAND/UCLA and private ranking in NGT. [Humphrey-Murto 2017 Med Teach, page 18]<br><br>6) How will anonymity be maintained? [Humphrey-Murto 2019, fig 1]<br><br>7) Ensure the anonymity of the participants. The anonymity of the experts was reported in virtually all of the studies [Paré 2013]
**Methods**<br>2.14 Does the study suggest anything about how to report, and in what level of detail, the feedback for panellists (in	1) Whether the experts were informed of both the response of the group and their own individual response (individual feedback) to each item. The type of feedback, which was defined as qualitative when a summary of the panel's comments was sent to each participant and quantitative when simple statistical summaries illustrating the collective opinion (e.g., central tendency and variance) were sent to each participant [page 2] After each round, each participant should be given the panel results (median, lowest, and highest ratings), the participant's response, and a summary of all comments received. These data inform each participant of his or her position relative to the rest of the group, thus assisting in decisions about replies during future Delphi rounds. [Boulkedid 2011, page 8] It has been recommended that

Delphi rounds or other methods) process? Or if this should be reported?	feedback should include qualitative comments and statistical measures [citation 51, Murphy 1998]. More specifically, we determined whether the experts were informed of both the response of the group and their own individual response (individual feedback) to each item [Boulkedid 2011]<br><br>2) Describe the type of feedback provided after each round [Chan 2019, Box 1]<br><br>3) Were participants' responses in each round reported back to the group, and were responses anonymized? [Gattrell 2019, Table 1]<br><br>4) Give attention to issues which guide data collection: the discovery of opinions, the process of determining the most important issues referring to the design of the initial round, and the management of opinions [Hasson 2020, page 1013]<br><br>5) Was formal feedback provided? If so, was the feedback described? [page 1493], areas that need to be improved with reporting providing participants with feedback of group ratings [Humphrey-Murto 2017 AMA, page 1494]<br><br>6) Describe the type of feedback provided after each round [page 18]. Feedback to participants can include quantitative and/or qualitative data. It also involves two types of agreement: the extent to which individual participants agree with an issue, and the extent to which participants agree with one another. Quantitative feedback may include summary statistics such as the participants' score, participants' medians, range of scores and the proportion of participants selecting each point on a scale. Participants are provided an opportunity to change their ranking, but it should be made clear that they do not need to conform. Researchers may ask the participants who are outliers to provide written justification for their choices (qualitative data) [Humphrey-Murto 2017 Med Teach]<br><br>7) What type of feedback will participants received after each round? [2019] indicates feedback between rounds should include individuals' scores for each item and the distribution of votes by participant group. Some, however, preferred to view aggregated feedback as well as feedback to individual participants [Humphrey-Murto 2019 Yes page 1042, table 1]<br><br>8) How was the feedback designed? [Niederberger 2020, table 2]<br><br>9) Citation [Schmidt, 54] recommends three relevant pieces of feedback that can be provided to experts in phase 3 in addition to mean ranks, namely, the interpretation of Kendall's W from the previous round, the percentage of experts

	placing each item in the top half of their list and the relevant comments that were made by the other panellists [Paré 2013, page 213]<br><br>10) They imply that it should be reported that panellist feedback was collected to inform subsequent Delphi rounds [Resemann 2018]<br><br>11) not about reporting but they state "57 % were silent about how the feedback after consensus was dealt with." suggesting that they felt it needs to be reported. [page 2] only that some reporting guidelines described the feedback information requirement, or gave the methods for feedback collection [Wang 2015, page 6]
**Methods**<br>2.15 Does the study suggest anything about how or if data synthesis/analysis should be reported (from any consensus method used and how this was calculated statistically) and in what level of detail?	1) It is important that standards and norms for prospectively defining analysis plans are needed to improve the credibility of Delphi processes for informing health research, practice, and policy [Grant 2018, page 97]<br><br>2) The methods employed need to be comprehensible; information about methods of data analysis, processing and synthesis of experts' responses to inform the subsequent survey round [Box 3] {Jünger 2017]<br>Reporting of results for each round separately is highly advisable in order to make the evolving of consensus over the rounds transparent. This includes figures showing the average group response, changes between rounds, as well as any modifications of the survey instrument such as deletion, addition or modification of survey items based on previous rounds." [Jünger 2017, item 13]<br><br>3) Detailing statistical analyses and interpretation in arriving at final agreed values [Ng 2018, item 7]<br><br>4) The statistical analyses should be reported [Paré 2013, page 211]<br><br>5) Consensus Development Panels: Statistical analysis: must be reasonable for the research question, and should be as rigorous as possible [Waggoner 2016, page 665]
**Methods**<br>2.16 Does the study suggest anything about how or if piloting should be reported and in what	1) Pilot testing with a small group of individuals is suggested before implementation [Humphrey-Murto 2017 Med Teach, page 16]<br><br>2) All material provided to the expert panel at the outset of the project and throughout the Delphi process should be carefully reviewed and piloted in advance in order to examine the effect on experts' judgements and to prevent bias. [Box 3] The methods employed need to be comprehensible; this includes information on preparatory steps (How was

level of detail (e.g. understanding of consensus items, platforms used, tools used)?	available evidence on the topic in question synthesised?), piloting of material and survey instruments, design of the survey instrument(s), the number and design of survey rounds, methods of data analysis, processing and synthesis of experts' responses to inform the subsequent survey round and methodological decisions taken by the research team throughout the process [Jünger 2017]<br><br>3) Pre-test task instructions and questionnaire instruments [Paré 2013]
**Methods**<br>2.17 Does the study suggest anything about how or if the role of Steering Committee members should be reported?	No data
**Methods**<br>2.18 Does the study suggest anything on what or if should be described regarding COI or funding?	1) 'Sources of funding (industry, non-industry)'as items associated with reporting quality [Banno 2019, page 2]<br><br>2) Is the funding source clearly disclosed? [table 1] Is the role of the funder clearly disclosed? [table 1] Is the funding of any external support (e.g. with the Delphi panel meeting/questionnaires, or medical writing support for the final manuscript) clearly disclosed? [Gattrell 2019]<br><br>3) "Prevention of bias. Researchers need to take measures to avoid directly or indirectly influencing the experts' judgements. If one or more members of the research team have a conflict of interest, entrusting an independent researcher with the main coordination of the Delphi study is advisable" [Jünger 2017]<br><br>4) Describing expert panel selection with eligibility criteria and including conflicts of interest [Ng 2018]
**Methods**<br>2.19 Does the study suggest anything on what should be described of how is dealt with COI of panellist (not allowed	1) No. It only deals with COI as a planning/methodological procedure, not reporting. "5. Prevention of bias. Researchers need to take measures to avoid directly or indirectly influencing the experts' judgements. If one or more members of the research team have a conflict of interest, entrusting an independent researcher with the main coordination of the Delphi study is advisable"[Jünger 2017]

to vote when there is COI)? Or if this should be described	

**Results**<br>3.1 Does the study suggest anything on how to report the initial evidence search (presentation of results of the literature review)?	1) No, but they suggest it should be reported [Jünger 2017]
**Results**<br>3.2 Does the study suggest anything on how to report n of studies found?	No data
**Results**<br>3.3 Does the study recommend which detail should be used when reporting panellists drop-outs (numbers and reasons)? Or if this should be reported?	1) No but it states that number the response rate for the first round dropped to 170 (66.1%). [page 1494]; areas that need improvement in reporting the number of participants after each round [page 1496] Other analyses of consensus methods research found similar poor reporting of this feature, with 7% to 39% of studies reporting response rates for all rounds of data collection [Humphrey-Murto 2017 AMA]<br><br>2) Fig 1 step 7 How will non-responders be managed, i.e. will they be excluded in subsequent rounds What response rate will be acceptable for each stakeholder group in each round? [Humphrey-Murto 2019]<br><br>3) Reporting of results for each round separately is highly advisable in order to make the evolving of consensus over the rounds transparent. This includes figures showing the average group response, changes between rounds, as well as any modifications of the survey instrument such as deletion, addition or modification of survey items based on previous rounds [Jünger 2017, Box 3]<br><br>4) Outlining participation and attrition rates for each round [Ng 2018]

	5) report the response rate to the initial request for participation, the size of the panel and the retention rate; [Paré 2013, page 215 3rd bullet]
**Results**<br>3.4 Does the study suggest how or if approval rates per item shared with respondents for each round should be reported in the Results section?	1) Response rate for each round [Boulkedid 2011, Table 5 on page 7]<br><br>2) Yes Box 1 report response rates and results after each round [Chan 2019]<br><br>3) Response rates for each round should be reported, presentation of total of issues generated in round 1, and presentation of results in round 2 indicating strength of support [Hasson 2000, figure 1 and page 1013]<br><br>4) Report response rates and results after each round [Humphrey-Murto 2017 Med Teach, page 18]<br><br>5) it should report response rates for all rounds [Humphrey-Murto 2019, page 1042]<br><br>6) Reporting of results for each round separately is highly advisable in order to make the evolving of consensus over the rounds transparent. This includes figures showing the average group response, changes between rounds, as well as any modifications of the survey instrument such as deletion, addition or modification of survey items based on previous rounds." [Jünger 2017, item 13]Criteria for the selection of experts and transparent information on recruitment of the expert panel, socio- demographic details including information on expertise regarding the topic in question, (non)response and response rates over the ongoing iterations should be reported". [Jünger 2017]<br><br>7) Reporting both quantitative results and textual comments for each round of analysis [Ng 2018]<br><br>8) How high was the response rate from the experts both when initially approached and also for the individual rounds [Niederberger 2020, Table 2]<br><br>9) Level of consensus should be reported [Resemann 2018]
**Results**<br>3.5 Does the study suggest anything about in which detail the items that have been dropped should	1) Were the criteria for dropping clear; are stopping criteria, other than rounds, reported [Banno 2019, item 3 and 4]<br><br>2) Were the criteria for dropping items clear? (yes, no, or not applicable) [Banno 2020, 2.6 item 3]<br><br>3) Clear criteria for dropping or combining items should also be specified based on the level of agreement or disagreement with individual items. One of the limitations of a priori specification is that certain items may fall just below the

be reported? (reasons e.g.) Or if this should be reported?	threshold for what is fundamentally an arbitrary cut off. In the event that items, believed to be important fell just below the threshold for inclusion in the study, the authors could consider including these items as posteriori considerations provided that sufficient justification was provided. [page 405] Suggested quality criteria: Were criteria for dropping items clear; Stopping criteria other than rounds specified? [Table 5] Were items dropped? What criteria will be used to determine which items to drop? [Diamond 2014, Table 6]<br><br>4) No, but they state Interpretation and processing of results. Consensus does not necessarily imply the correct answer or judgement; (non)consensus and stable disagreement provide informative insights and highlight differences in perspectives concerning the topic in question and Definition and attainment of consensus. It needs to be comprehensible to the reader how consensus was achieved throughout the process, including strategies to deal with non-consensus [Jünger 2017 in Box 3]<br><br>5) Were criteria defined for dropping items [Niederberger 2020, page 6]
**Results**<br>3.6 Does the study make any recommendation on how to report the collection, synthesis and use of comments from panellists? Or if this should be reported?	1) It has been recommended that feedback should include qualitative comments and statistical measures [Murphy 1998, 51]. After each round, each participant should be given the panel results (median, lowest, and highest ratings), the participant’s response, and a summary of all comments received [Boulkedid 2011]<br><br>2) Describe the type of feedback provided after each round. Quantitative feedback may include summary statistics such as the participants’ score, participants’ medians, range of scores and the proportion of participants selecting each point on a scale. Participants are provided an opportunity to change their ranking, but it should be made clear that they do not need to conform [Humphrey-Murto 2017 Med Teach]<br><br>3) Reporting of results for each round separately is highly advisable in order to make the evolving of consensus over the rounds transparent. This includes figures showing the average group response, changes between rounds, as well as any modifications of the survey instrument such as deletion, addition or modification of survey items based on previous rounds [Jünger 2017, item 13]<br><br>4) Ask experts to justify their rankings. Have experts comment and validate consolidated list [page 210 Table 3]. Did experts consolidate the list of items; Did experts comment on and validate the list of items; Was the final number of items reported.<br>Report whether panel members had the opportunity to justify or clarify their own reasoning and to comment on the responses of the other experts as well as on the progress of the panel as a whole. [Paré 2013, page 213].

	Were panellists able to revise previous statements [Paré 2013]<br><br>5) No, but implied that it should be: did not report collecting panellist feedback to inform subsequent Delphi stages [Resemann 2018]
**Results**<br>3.7 Does the study suggest regarding how the final list of items (for clinical guideline or reporting guideline) should be reported? Or if this should be reported?	1) Partially. It says it should be detailed and disseminated, but it does not suggest how (in what format) it should be reported [Jünger 2017]<br><br>2) Suggests "detailing statistical analyses and interpretation in arriving at final agreed values" [Ng 2018]<br><br>3) Report final number of items [Paré 2013, page 210 Table 3]<br><br>4) No but again imply "reported the number of statements assessed." [Resemann 2018]

**Discussion**<br>4.1 Does the paper suggest anything about reporting the limitations and strengths of the study and how? Or if this should be reported?	1) Address potential methodological issues (e.g lack of consensus) or limitations in the discussion (e.g. low response rate) [Chan 2019, Box 1]<br><br>2) Interpretation of consensus gained/not gained [Hasson 2020, page 1009]<br><br>3) In the discussion the authors should address issues that may have impacted the results such as poor response rates between rounds, lack of participation from a select group or geographic region, or lack of consensus. [Humphrey-Murto 2017 Med Teach, page 18]<br><br>4) Methodological issues should be reported [Humphrey-Murto 2019, figure 1]<br><br>5) Reporting should include a critical reflection of potential limitations and their impact of the resulting guidance". [Jünger 2017]
**Discussion**<br>4.2 Does the paper suggest anything about what or in	1) Page 5: is considered a good measure if it meets criteria including reliability, sensitivity, specificity, and feasibility (or applicability) [20,31]. The common use of these characteristics can facilitate acceptance and implementation of indicators developed [Boulkedid 2011]

which detail the applicability generalisability, and reproducibility of the study should be reported? Or if this should be reported?	2) The conclusions should adequately reflect the outcomes of the Delphi study with a view to the scope and applicability of the resulting practice guidance. [Jünger 2017, item 15]<br><br>3) It is also necessary to discuss the critical and rationalistic criteria for the validity and reliability of the studies and the more constructivist characteristics of credibility, transparency, and transferability. [Niederberger 2020, page 8]

**5.1 Any other item proposed by the paper that is not captured in other columns?**	1) Were criteria for dropping items clear? Are stopping criteria, other than rounds, specified [Banno 2019]<br><br>2) Differences between the protocol and the article [Banno 2020, 2.9]<br><br>3) Geographic scope of the survey [page 2]. Main methods used to send the questionnaires (e.g., mail, E-mail, or fax). [Boulkedid 2011, page 7]<br>The formulation of the questionnaire items (e.g., open questions, rating of quality indicators, or both). [Boulkedid 2011]<br>Whether the quality indicators were rated (in which case, we recorded the minimum and maximum values on the rating scale). [Boulkedid 2011]<br>A flow chart of quality indicators (figure showing the output and input indicators at each round) and/or for a written description of indicator flow. [Boulkedid 2011, page 3]<br>Quality indicators used in the first round versus the end of the last round. [Boulkedid 2011, page 3]<br>Availability of the questionnaires in the article itself or in an appendix [Boulkedid 2011, page 3]<br>Whether selection criteria changed between rounds [Boulkedid 2011, page 5]<br>Whether panelists were able to make comments. [Boulkedid 2011, page 6]<br>Whether there was a meeting; at what stage it took place and how people participated [Boulkedid 2011]<br>Response rate for each round [Boulkedid 2011, page 7]<br>preparation in advance of starting Delphi (outcome indicators, structure indicators, process indicators) [Boulkedid 2011, In appendix S1, item 1]<br>METHODS<br>We evaluated the relationship between the response rate and the use of specific methods to encourage the experts to respond (e.g., stamped addressed envelope for returning the questionnaire and financial compensation). Also on maybe we should add item regarding encouragement of participants [Boulkedid 2011, page 2, page 5 right column]<br>Geographic scope of Delphi consensus procedure [Boulkedid 2011,item 20 of appendix and table 5]<br>Question format (open questions, rating scale?) Also in table 5 how were questions formulated? [Boulkedid 2011, item 24

	appendix]<br>Rating scale [Boulkedid 2011, item 25]<br>Methods used to send questionnaire (email fax, mail) [Boulkedid 2011, table 5]<br>Time to complete questionnaire reporting of differences in response rate in rounds [Boulkedid 2011]<br>Number of rounds necessary to reach consensus [Boulkedid 2011]<br>Duration of the procedure [Boulkedid 2011]<br>Is questionnaire added as appendix? [Boulkedid 2011]<br>For Discussion: Validity [Boulkedid 2011]<br><br>4) Outline each step of the process. If modifications were made, provide a rationale for your choices. [Chan 2019]<br>Describe the selection and preparation of the scientific evidence for the participants. [Chan 2019]<br>Include a description of the facilitator's credentials. [Chan 2019]<br>What background material was provided to participants. [Chan 2019]<br>What formal feedback of group rating was shared between rounds [Chan 2019]<br><br>5) Specify stopping criteria in the absence of consensus [Diamond 2014]<br><br>6) Were the questions formulated or validated by an expert panellist [Gattrell 2019]<br><br>7) Researchers conducting consensus-oriented Delphi processes should prospectively and completely register the intended procedure for identifying which items reach consensus. [Grant 2018]<br>The analysis procedure for determining consensus for Delphi processes should be chosen a priori ideally before starting the first round but at the very latest before completing data collection to improve the validity of findings. [Grant 2018]<br>Health researchers conducting consensus-oriented Delphi processes should commit themselves in advance to an analytic procedure for determining which items reach consensus before they see the actual data (or, ideally, before they even collect the data). [Grant 2018]<br>Registrations should be in a publicly available and independently controlled platform that time-stamps entries [Grant 2018]<br><br>8) "Copy of each round questionnaire illustrated" [Hasson 2020]<br>statistical interpretation for the reader [Hasson 2020]<br>appendices to include the questionnaires [Hasson 2020]<br>For Discussion interpretations of consensus gained/not gained reliability and validity [Hasson 2020]

	9) *Page 1493(2) Was background information provided to the participants? pg 1496 areas appeared particularly problematic and were often left out or poorly described: providing background information to participants<br>AND so a clear description of what information was provided and in what format is important<br>* (3) Was the consensus method used for item generation, ranking, or both?<br>* (11) Was consensus forced?<br>Was mail/e-mail polling or face-to-face questioning used? [Humphrey-Murto 2017 AMA]<br><br>10) Outline each step of the process: if modifications were made, provide a rationale for the choices made. Providing justification for the choices made will also add credibility. [Humphrey-Murto 2017 Med Teach]<br><br>11) Background provided to participants, what is level of detail provided [Humphrey-Murto 2019]<br>Figure 1 clear outline of the overall process involved and where Delphi fits [Humphrey-Murto 2019, figure 1]<br>How sample size is determined of participants [Humphrey-Murto 2019, figure 1]<br><br>12) Any modifications should be justified by a rationale and be applied systematically and rigorously [Jünger 2017, Box 3]<br>All material provided to the expert panel at the outset of the project and throughout the Delphi process should be carefully reviewed and piloted in advance in order to examine the effect on experts' judgements and to prevent bias [Jünger 2017]<br>It is recommended to have the final draft of the resulting guidance on best practice in palliative care reviewed and approved by an external board or authority before publication and dissemination [Jünger 2017, Box 3]<br>information about methodological decisions taken by the research team throughout the process Jünger 2017, Box 3]<br>Flow chart to illustrate the stages of the Delphi process, including a preparatory phase, the actual Delphi rounds, interim steps of data processing and analysis, and concluding steps [Jünger 2017, Box 3]<br>Publication and dissemination [Jünger 2017, Box 3]<br><br>13) Item 2-4 and 9 appending revised questionnaires [Ng 2018]<br><br>14) Specific definition of underlying Delphi technique (or as I thought it is important to define exactly what method is used, especially if a modified method is used this needs to be very clear [Niederberger 2020]<br>What role did the stability of the answers play? [Niederberger 2020, table 2]<br>Questionnaire and scale development How were the questionnaires and the specific items for a Delphi technique

	developed? [Niederberger 2020]<br>Nevertheless, it is important to precisely describe, justify, and methodologically reflect on any modifications [Niederberger 2020]<br>How were the questionnaires and the specific items for a Delphi technique developed? [Niederberger 2020, Table 2]<br>Were items identified from empirical analyses such as qualitative interviews or focus groups that were completed in advance or were taken from existing guidelines. [Niederberger 2020, Complementary AND page 6<br>Was the first (qualitative) round of questions in the Delphi process used to generate the items for a standardized questionnaire. [Niederberger 2020, Complementary AND page 6]<br><br>15) Was the final number of items reported [Paré 2013, Table 3] Were items randomly ordered [Paré 2013, Table 3]<br><br>16) Describe the rating scales used [Resemann 2018] the number of statements assessed should be reported [Resemann 2018]<br><br>17) For nominal group process, the research question used to prompt the panel must be clear and concise to obtain valid suggestions from panel members. [Waggoner 2016, page 665] The heterogeneity should be reported [Waggoner 2016, page 665] Evaluation of reliability [Waggoner 2016, page 665]<br><br>18) Meeting attendance; format (e.g. face-to-face); agenda preparation; materials sent to participants prior to meeting; duration of meeting [Wang 2015, page 5] Flow diagram [Wang 2015, page 3] Should we add something regarding other consensus methods including an item regarding face to face meetings? [Wang 2015, page 5]
**5.2 Any other item not proposed by the paper, but you think that could be added (not fitting the categories above)?**	1) Are stopping criteria, other than rounds, specified? [Banno 2019, page 2]<br><br>2) Information letter explaining the method and the reasons their participation to the whole process would be necessary, as well as a form for collecting their consent to complete the entire Delphi process. [Boulkedid 2011]<br><br>3) "Round 1: presentation of total number of issues generated" [Hasson 2020]<br><br>4) This paper was "pointing fingers", showing what was wrong, without suggesting solutions. However, we can be inspired by the critics to build the following list of items: 1) Purpose of the consensus study<br>Whether a literature review was done to support the selection of items [Humphrey-Murto 2017 AMA]<br><br>5) Length of the background provided [Humphrey-Murto 2019]

	Purpose of study: outcome/diagnosis/intervention? [Humphrey-Murto 2019]

**Examples of text with well reported methods/results (for E&E document) - write NA if none was cited or found by you**	1) Page 7 Table 5 [Boulkedid 2011]<br>2) Box 1 [Chan 2019]<br>3) Might have a look at table 6 [Diamond 2014]<br>4) Table 1 [Gattrell 2019]<br>5) Parts of Fig 1 and checklist page 1013 [Hasson 2020]<br>6) Table 1 lists "exemplary publications" for nominal group process, consensus development panel and Delphi technique Page 667 references studies that were "Very descriptive" of the statistical techniques used. [Waggoner 2016]
**Additional comments from assessor**	1) Limited value; protocol for Banno 2020 [Banno 2019]<br>2) Of limited use. The authors developed a 4-point quality score that they applied to Delphi publications [Banno 2020]<br>3) Excellent resource [Boulkedid 2011]<br>4) Focusses on defining consensus [Diamond 2014]<br>5) Congress poster only [Gattrell 2019]<br>6) Study used RAND's ExpertLens as the Delphi platform [Grant 2018]<br>7) 1497: The lack of consensus on consensus methods makes it imperative that researchers provide clear and detailed reporting of the methods they used and that they justify these choices. [Humphrey-Murto 2017]

	8) Page 1044 A suggestion to improv uniformity is to use a software program that provides structure and help with reporting all relevant outcomes (e.g. DelphiManager, http://comet-initiative.org/delphimanager/) [Humphrey-Murto 2019]<br><br>9) Very informative [Jünger 2017]<br><br>10) The study focusses on information systems. Arguably, this is not within the inclusion criteria for the search [Paré 2013]<br><br>11) Review covers nominal group process, consensus development panel and Delphi technique [Waggoner 2016]<br><br>12) Study looked at the reporting quality of reporting guidelines [Wang 2015]
