# Supplementary material for: Existing guidance on reporting of consensus methodology: a systematic review to inform ACCORD guideline development
Source: BMJ Open. 2022 Sep 8;12(9):e065154. doi: 10.1136/bmjopen-2022-065154 (PMC9462098; doi:10.1136/bmjopen-2022-065154)
Supplement: Supplementary data [file bmjopen-2022-065154supp005.pdf]

ACcurate COnsensus Reporting Document (ACCORD): Summary of extracted data from literature search

Table of Contents

1. Background ***2***

2. Methods ***3***

3. Results ***9***

4. Discussion ***11***

5. Additional topics ***12***

References ***14***

van Zuuren EJ, *et al. BMJ Open* 2022; 12:e065154. doi: 10.1136/bmjopen-2022-065154

Section: Background

1. Background

Data extraction question	Articles	Checklist item(s) with brief explanation
1.1. Does the study suggest anything about how or if consensus papers should report the context or rationale for choosing a consensus method over other methods?	Hasson F, *et al. J Adv Nurs* 2000[1]<br>Humphrey-Murto S, *et al. Med Teach* 2017[2]<br>Humphrey-Murto S, *et al. J Rheumatol* 2019[3]<br>Jünger S, *et al. Palliat Med* 2017[4]	State the rationale for use of consensus method over other options.<br>*Should consider other consensus methods as well as other methodology types.*
1.2. Does the study suggest anything about how/what or if consensus papers should report the objectives of the consensus exercise?	Hasson F, *et al. J Adv Nurs* 2000[1]<br>Humphrey-Murto S, *et al. Med Teach* 2017[2]<br>Jünger S, *et al. Palliat Med* 2017[4]<br>Boulkedid R, *et al. PLoS One* 2011[5]<br>Chan TM, *et al. CJEM* 2019[6]<br>Diamond IR, *et al. J Clin Epidemiol* 2014[7]	Clearly define study objectives.<br>*Could include presentation of group consensus, or just to quantify the level of agreement.*

Section: Methods

2. Methods

Data extraction question	Articles	Checklist item(s) with brief explanation
2.1. Does the study suggest anything about how/what or if consensus papers should report regarding:<br><br>A literature search/strategy?	Hasson F, *et al. J Adv Nurs* 2000[1]<br>Humphrey-Murto S, *et al. Med Teach* 2017[2]<br>Chan TM, *et al. CJEM* 2019[6]<br>Humphrey-Murto S, *et al. Acad Med* 2017[8]<br>Resemann HK, *et al. Curr Med Res Opin* 2018[9]	A) Describe the strategy for reviewing the existing scientific evidence that informed the study.<br>*If no existing literature is available, the extent of the search should be described.*<br>B) Describe how existing scientific evidence will be provided to the participants.<br>*If different participant groups are involved, it should be stated which information will be provided to which group.*
2.2. Does the study suggest anything about how/what or if consensus papers should report regarding:<br><br>Inclusion and exclusion criteria for the literature search?	Boulkedid R, *et al. PLoS One* 2011[5]<br>Chan TM, *et al. CJEM* 2019[6]<br>Paré G, *et al. Inf Manag* 2013[10]	Describe the process of the literature search.<br>*Should include inclusion and exclusion criteria, and state whether these were prespecified.*
2.3. Does the study suggest anything of what or if consensus report should report on panel composition, n of participants, expertise, origin? Prespecified?	Hasson F, *et al. J Adv Nurs* 2000[1]<br>Humphrey-Murto S, *et al. Med Teach* 2017[2]<br>Humphrey-Murto S, *et al. J Rheumatol* 2019[3]<br>Jünger S, *et al. Palliat Med* 2017[4]<br>Boulkedid R, *et al. PLoS One* 2011[5]<br>Chan TM, *et al. CJEM* 2019[6]<br>Diamond IR, *et al. J Clin Epidemiol* 2014[7]<br>Humphrey-Murto S, *et al. Acad Med* 2017[8]<br>Paré G, *et al. Inf Manag* 2013[10]<br>Banno M, *et al. J Clin Epidemiol* 2019[11]<br>Banno M, *et al. J Clin Epidemiol* 2020[12]<br>Gattrell WT, *et al. Curr Med Res Opin* 2019[13]	A) Describe the structure of the study's participants.<br>*Should describe inclusion of a Chair/Co-chairs, steering committee, and subgroups, if applicable.*<br>B) Explain how panel participants were selected.<br>*Should state who was responsible for panellist selection, the selection criteria applied, the justification for choosing panellist numbers and selection criteria, and whether criteria were prespecified.*

van Zuuren EJ, *et al. BMJ Open* 2022; 12:e065154. doi: 10.1136/bmjopen-2022-065154

Section: Methods

Data extraction question	Articles	Checklist item(s) with brief explanation
	Ng J. *Value Health* 2018[14]<br>Niederberger M, *et al. Front Public Health* 2020[15]<br>Waggoner J, *et al. Acad Med* 2016[16]<br>Wang X, *et al. BMC Med Res Methodol* 2015[17]	*C)* Describe the composition of the panel. *Should include number of participants at all stages of the process, sociodemographics (e.g. age, sex, specialty, type and duration of relevant experience). Should also describe panel subgroups, if relevant.*<br>D) Describe the expertise of the panel. *Should include the definition of "expert" and description of any public or patients involved.*<br>E) Describe the facilitator(s), if used. *Should include type and duration of relevant experience, and the role played in the process.*
2.4. Does the study suggest anything of how or if PPI (public patient involvement) activity should be reported	No data	Describe the role and involvement of any public or patients.<br>*Should detail the stage(s) at which they were involved, and their roles and contributions.*
2.5. Does the study suggest anything about what or if consensus papers should report regarding panel recruitment strategies, invitations? Any level of detail specified?	Jünger S, *et al. Palliat Med* 2017[4]<br>Boulkedid R, *et al. PLoS One* 2011[5]<br>Paré G, *et al. Inf Manag* 2013[10]<br>Waggoner J, *et al. Acad Med* 2016[16]	Describe how the panel members were recruited.<br>*Could include communication/advertisement method(s) and locations.*
2.6. Does the study suggest how or if consensus papers should report the consensus criteria/threshold (or the level of agreement considered to reach consensus)?	Hasson F, *et al. J Adv Nurs* 2000[1]<br>Humphrey-Murto S, *et al. Med Teach* 2017[2]<br>Humphrey-Murto S, *et al. J Rheumatol* 2019[3]<br>Jünger S, *et al. Palliat Med* 2017[4]<br>Boulkedid R, *et al. PLoS One* 2011[5]<br>Chan TM, *et al. CJEM* 2019[6]<br>Diamond IR, *et al. J Clin Epidemiol* 2014[7]<br>Humphrey-Murto S, *et al. Acad Med* 2017[8]<br>Gattrell WT, *et al. Curr Med Res Opin* 2019[13]	A) Define the consensus measure to be used. *Could include percentage agreement, units of central tendency (e.g. median), a categorical rating (e.g. Agree/Strongly agree) or a combination of percent agreement within a certain range.*<br>B) State the threshold for the group achieving consensus. *Should include whether the threshold was*

Section: Methods

**Data extraction question**	**Articles**	**Checklist item(s) with brief explanation**
	Niederberger M, *et al. Front Public Health* 2020[15]<br>Waggoner J, *et al. Acad Med* 2016[16]<br>Wang X, *et al. BMC Med Res Methodol* 2015[17]<br>Grant S, *et al. J Clin Epidemiol* 2018[18]	*pre-defined and highlight any threshold variation between rounds, with explanation for the change. If the intention is to quantify the degree of consensus but not to use consensus as a stop criterion for the study, this should be stated.*
2.7. Does the study suggest how or if consensus papers should report how decision of approval of an item will be made?	Hasson F, *et al. J Adv Nurs* 2000[1]<br>Jünger S, *et al. Palliat Med* 2017[4]<br>Boulkedid R, *et al. PLoS One* 2011[5]	Explain how final consensus was reached.<br>*Should describe the evolution of themes between voting rounds, if applicable.*
2.8. Does the study suggest anything about what level of detail should be reported regarding the number of Delphi rounds or if this should be reported?	Hasson F, *et al. J Adv Nurs* 2000[1]<br>Humphrey-Murto S, *et al. Med Teach* 2017[2]<br>Jünger S, *et al. Palliat Med* 2017[4]<br>Chan TM, *et al. CJEM* 2019[6]<br>Diamond IR, *et al. J Clin Epidemiol* 2014[7]<br>Humphrey-Murto S, *et al. Acad Med* 2017[8]<br>Banno M, *et al. J Clin Epidemiol* 2019[11]<br>Banno M, *et al. J Clin Epidemiol* 2020[12]<br>Niederberger M, *et al. Front Public Health* 2020[15]<br>Waggoner J, *et al. Acad Med* 2016[16]	State how many voting rounds were conducted.<br>*Should include whether the number of rounds was prespecified, and whether this was an absolute or a maximum. If the maximum was exceeded, should explain the reasoning for doing so.*
2.9. Does the study suggest anything about what level of detail should be reported regarding the criteria used for defining the number of rounds? (why 2-3 or more e.g.) or if this should be reported?	Humphrey-Murto S, *et al. Med Teach* 2017[2]<br>Humphrey-Murto S, *et al. J Rheumatol* 2019[3]<br>Chan TM, *et al. CJEM* 2019[6]<br>Humphrey-Murto S, *et al. Acad Med* 2017[8]<br>Paré G, *et al. Inf Manag* 2013[10]<br>Banno M, *et al. J Clin Epidemiol* 2020[12]<br>Niederberger M, *et al. Front Public Health* 2020[15]<br>Waggoner J, *et al. Acad Med* 2016[16]	Explain the rationale for choosing the number of voting rounds.<br>*Should also describe the stop criteria, if used, and whether these were prespecified.*
2.10. Does the study suggest anything about the details that should be reported regarding the time between rounds, if this should be	Boulkedid R, *et al. PLoS One* 2011[5]	Describe the time period between voting rounds.<br>*Should include whether the period was*

Section: Methods

Data extraction question	Articles	Checklist item(s) with brief explanation
prespecified in advance, or if this should be reported?		*prespecified and highlight differences between inter-round periods, if applicable.*
2.11. Does the study suggest anything about details that should be reported of the names of the techniques of non-Delphi methods used to gather participants' inputs and reach consensus?	Boulkedid R, *et al. PLoS One* 2011[5]<br>Waggoner J, *et al. Acad Med* 2016[16]	Describe any additional methods used alongside the consensus process.<br>*Should include all that were used, e.g. a self-administered questionnaire combined with a group meeting. Should also explain how the consensus process fitted into the overall study methodology.*
2.12. Does the study suggest anything of what or in which detail should be reported regarding tool or electronic system used for Delphi? (If Delphi was used)? Or if this should be reported?	Humphrey-Murto S, *et al. J Rheumatol* 2019[3]	Describe any tools used to administer the voting.<br>*Could detail electronic platforms, if used.*
2.13. Does the study suggest anything about how or in what level of detail the anonymity of participants (in Delphi or other methods) has to be reported? Or if this should be reported?	Humphrey-Murto S, *et al. Med Teach* 2017[2]<br>Humphrey-Murto S, *et al. J Rheumatol* 2019[3]<br>Chan TM, *et al. CJEM* 2019[6]<br>Humphrey-Murto S, *et al. Acad Med* 2017[8]<br>Paré G, *et al. Inf Manag* 2013[10]<br>Banno M, *et al. J Clin Epidemiol* 2020[12]<br>Gattrell WT, *et al. Curr Med Res Opin* 2019[13]	Detail how anonymity of voters was maintained.<br>*Could involve use of mail-outs in a standard Delphi procedure, blinding on an electronic platform, or private ranking in the NGT.*
2.14. Does the study suggest anything about how to report, and in what level of detail, the feedback for panellists (in Delphi rounds or other methods) process? Or if this should be reported?	Hasson F, *et al. J Adv Nurs* 2000[1]<br>Humphrey-Murto S, *et al. Med Teach* 2017[2]<br>Humphrey-Murto S, *et al. J Rheumatol* 2019[3]<br>Boulkedid R, *et al. PLoS One* 2011[5]<br>Chan TM, *et al. CJEM* 2019[6]<br>Humphrey-Murto S, *et al. Acad Med* 2017[8]<br>Resemann HK, *et al. Curr Med Res Opin* 2018[9]<br>Paré G, *et al. Inf Manag* 2013[10]<br>Gattrell WT, *et al. Curr Med Res Opin* 2019[13]<br>Niederberger M, *et al. Front Public Health* 2020[15]	Explain how voting feedback was provided to panellists at the end of each round.<br>*Could include summaries of group voting and/or their own individual responses. Should state whether feedback will be quantitative and/or qualitative, and whether it will be anonymised. If no feedback was provided, this should be stated.*

Section: Methods

Data extraction question	Articles	Checklist item(s) with brief explanation
	Wang X, *et al. BMC Med Res Methodol* 2015[17]	
2.15. Does the study suggest anything about how or if data synthesis/analysis should be reported (from any consensus method used and how this was calculated statistically) and in what level of detail?	Jünger S, *et al*. *Palliat Med* 2017[4]<br>Paré G, *et al. Inf Manag* 2013[10]<br>Ng J. *Value Health* 2018[14]<br>Waggoner J, *et al*. *Acad Med* 2016[16]<br>Grant S, *et al*. *J Clin Epidemiol* 2018[18]	Detail methods used to process responses after each voting round.<br>*Could include statistical analysis methods, if used.*
2.16. Does the study suggest anything about how or if piloting should be reported and in what level of detail (e.g. understanding of consensus items, platforms used, tools used)?	Humphrey-Murto S, *et al*. *Med Teach* 2017[2]<br>Jünger S, *et al*. *Palliat Med* 2017[4]<br>Paré G, *et al. Inf Manag* 2013[10]	Describe any piloting of the study materials and/or survey instruments.<br>*Should include the number of individuals in the pilot group and the rationale for their selection. Should also explain any changes made as a result of the pilot. If no pilot was conducted, this should be stated.*
2.17. Does the study suggest anything about how or if the role of Steering Committee members should be reported?	No data	Describe the role(s) of the Steering Committee in the process.<br>*Should also detail the involvement of the Chair/Co-chairs, subgroups, or individual members at relevant stages of the process, if different from the group as a whole.*
2.18. Does the study suggest anything on what or if should be described regarding COI or funding?	Jünger S, *et al*. *Palliat Med* 2017[4]<br>Banno M, *et al*. *J Clin Epidemiol* 2019[11]<br>Gattrell WT, *et al*. *Curr Med Res Opin* 2019[13]<br>Ng J. *Value Health* 2018[14]	A) Disclose any COI of the panellists<br>*Should specify COI of each participant in the panel.*<br>B) Disclose any funding received and the role of the funder.<br>*Should specify the role of the funding source(s), e.g. involvement in the study concept/design, participation of the Steering Committee, for conducting the consensus process, medical writing support for its reporting.*

Section: Methods

Data extraction question	Articles	Checklist item(s) with brief explanation
2.19. Does the study suggest anything on what should be described of how is dealt with COI of panellist (not allowed to vote when there is COI)? Or if this should be described	Jünger S, *et al*. *Palliat Med* 2017[4]	Describe measures taken to avoid influence by any conflicts of interest (COI).<br>*Should include disclosure of COI and how this was accounted for in the methodology, e.g. by limiting voting in case of a specific COI, adjudication by an independent researcher.*

Section: Results

3. Results

Data extraction question	Articles	Checklist item(s) with brief explanation
3.1. Does the study suggest anything on how to report the initial evidence search (presentation of results of the literature review)?	Jünger S, *et al. Palliat Med* 2017[4]	Describe how existing scientific evidence was provided to the participants.<br>*Should include relevant specifics of the literature search, e.g. n of studies reported, to provide relevant context for the results. If different participant groups were involved, it should be stated which information was provided to which group.*
3.2. Does the study suggest anything on how to report n of studies found?	No data	Describe the results of the search and number of included studies.
3.3. Does the study recommend which detail should be used when reporting panellists drop-outs (numbers and reasons)? Or if this should be reported?	Humphrey-Murto S, *et al. J Rheumatol* 2019[3]<br>Jünger S, *et al. Palliat Med* 2017[4]<br>Humphrey-Murto S, *et al. Acad Med* 2017[8]<br>Paré G, *et al. Inf Manag* 2013[10]<br>Ng J. *Value Health* 2018[14]	A) State the response rates for each voting round.<br>*Should specify n as well as percent, or otherwise indicate attrition/retention rates.*<br>B) State the reasons cited for voter drop-outs at each stage of the process.<br>*Could be provided as an aggregated summary or as individual responses. If this information was not collected, this should be stated.*<br>C) Describe measures undertaken to maintain acceptable response rates.<br>*If threshold rates differ between stakeholder groups, these should be described with explanation.*

van Zuuren EJ, *et al. BMJ Open* 2022; 12:e065154. doi: 10.1136/bmjopen-2022-065154

Section: Results

Data extraction question	Articles	Checklist item(s) with brief explanation
3.4. Does the study suggest how or if approval rates per item shared with respondents for each round should be reported in the Results section?	Hasson F, *et al. J Adv Nurs* 2000[1]<br>Humphrey-Murto S, *et al. Med Teach* 2017[2]<br>Humphrey-Murto S, *et al. J Rheumatol* 2019[3]<br>Jünger S, *et al. Palliat Med* 2017[4]<br>Boulkedid R, *et al. PLoS One* 2011[5]<br>Chan TM, *et al. CJEM* 2019[6]<br>Resemann HK, *et al. Curr Med Res Opin* 2018[9]<br>Ng J. *Value Health* 2018[14]<br>Niederberger M, *et al. Front Public Health* 2020[15]	Describe which results that were shared with respondents after each voting round were reported in the final manuscript.<br>*Could include response rates, the type of information presented, summaries of group voting and/or individual responses. If this information is not provided, this should be stated together with the rationale.*
3.5. Does the study suggest anything about in which detail the items that have been dropped should be reported? (reasons e.g.) Or if this should be reported?	Jünger S, *et al. Palliat Med* 2017[4]<br>Diamond IR, *et al. J Clin Epidemiol* 2014[7]<br>Banno M, *et al. J Clin Epidemiol* 2019[11]<br>Banno M, *et al. J Clin Epidemiol* 2020[12]<br>Niederberger M, *et al. Front Public Health* 2020[15]	A) List any voting items that were dropped.<br>B) Explain the rationale for dropping any voting items.<br>*Should state whether the criteria for dropping any items were prespecified.*
3.6. Does the study make any recommendation on how to report the collection, synthesis and use of comments from panellists? Or if this should be reported?	Humphrey-Murto S, *et al. Med Teach* 2017[2]<br>Jünger S, *et al. Palliat Med* 2017[4]<br>Boulkedid R, *et al. PLoS One* 2011[5]<br>Resemann HK, *et al. Curr Med Res Opin* 2018[9]<br>Paré G, *et al. Inf Manag* 2013[10]	Describe how responses were processed prior to reporting.<br>*Should describe methods by which responses were analysed, aggregated or summarised, include whether any statements were revised between voting rounds, and state by whom the information was processed.*
3.7. Does the study suggest regarding how the final list of items (for clinical guideline or reporting guideline) should be reported? Or if this should be reported?	Jünger S, *et al. Palliat Med* 2017[4]<br>Resemann HK, *et al. Curr Med Res Opin* 2018[9]<br>Paré G, *et al. Inf Manag* 2013[10]<br>Ng J. *Value Health* 2018[14]	Report the final outcomes.<br>*Could be quantitative (e.g. summary statistics, score means, medians and/or ranges) and/or qualitative (e.g. aggregated themes from comments). Should be clear, accurately represent the consensus methodology used, and relevant to the field.*

Section: Discussion

4. Discussion

Data extraction question	Articles	Checklist item(s) with brief explanation
4.1. Does the paper suggest anything about reporting the limitations and strengths of the study and how? Or if this should be reported?	Hasson F, *et al. J Adv Nurs* 2000[1]<br>Humphrey-Murto S, *et al. Med Teach* 2017[2]<br>Humphrey-Murto S, *et al. J Rheumatol* 2019[3]<br>Jünger S, *et al. Palliat Med* 2017[4]<br>Chan TM, *et al. CJEM* 2019[6]	Discuss the study's methodological strengths and limitations.<br>*Should address issues that may impact results, e.g. response rates or representation.*
4.2. Does the paper suggest anything about what or in which detail the applicability generalisability, and reproducibility of the study should be reported? Or if this should be reported?	Jünger S, *et al. Palliat Med* 2017[4]<br>Boulkedid R, *et al. PLoS One* 2011[5]<br>Niederberger M, *et al. Front Public Health* 2020[15]	A) Discuss the reliability of the study.<br>B) Discuss the sensitivity of the study.<br>C) Discuss the specificity of the study.<br>D) Discuss the applicability of the study.<br>E) Discuss the validity of the study.

Section: Additional topics

5. Additional topics

Data extraction question: Any other item proposed by the paper that is not captured in previous sections?

Articles	Checklist item(s) with brief explanation
Humphrey-Murto S, *et al. Med Teach* 2017[3]<br>Jünger S, *et al. Palliat Med* 2017[4]<br>Boulkedid R, *et al. PLoS One* 2011[5]<br>Paré G, *et al. Inf Manag* 2013[10]<br>Banno M, *et al. J Clin Epidemiol* 2020[12]	Explain any deviations from the planned protocol.<br>*Should include any affected stages, including but not limited to change in panel number or composition, number of voting rounds, stopping criteria, statistical plan, reporting of outcomes.*
Boulkedid R, *et al. PLoS One* 2011[5]<br>Resemann HK, *et al. Curr Med Res Opin* 2018[9]	Describe the formulation of questions.<br>*Should include the type of questions, e.g. open questions, numerical rating, level of agreement rating. If rating questions were used, the scale range should be stated, and whether respondents were able to leave additional comments after rating items.*
Boulkedid R, *et al. PLoS One* 2011[5]<br>Wang X, *et al. BMC Med Res Methodol* 2015[17]	Describe any group meetings that were held.<br>*Should state at what stage the meeting took place, objectives/purpose, format (e.g. face-to-face or virtual), pre-read materials shared, attendance, location, duration, and how individuals participated.*
Hasson F, *et al. J Adv Nurs* 2000[1]<br>Boulkedid R, *et al. PLoS One* 2011[5]<br>Ng J. *Value Health* 2018[14]	List any items included in the appendix accompanying the main report.<br>*Could include e.g. full voting questions from each round with response rates, or information provided to the panel as pre-reads or to summarise voting rounds.*
Boulkedid R, *et al. PLoS One* 2011[5]	State how the survey was presented to participants.<br>*For example, as hard copy or via digital platform; could include description of email or mailing process. Should describe any randomisation procedures for questions, if used. If questions were not randomised, this should be stated.*
Boulkedid R, *et al. PLoS One* 2011[5]	Describe incentives for encouraging responses.<br>*Should list any specific methods, e.g. paid return postage for the questionnaire or financial compensation.*
Boulkedid R, *et al. PLoS One* 2011[5]	State the period in which the process was conducted.
Grant S, *et al. J Clin Epidemiol* 2018[18]	Describe any prospective registrations for the consensus process.

Section: Additional topics

Articles	Checklist item(s) with brief explanation
	Should include the platform on which it was registered and a link, if applicable. If the process was not registered, this should be stated.
Jünger S, *et al*. *Palliat Med* 2017[4]	Describe any external peer review prior to publication.<br>*Should name the authority, state the rationale for their review, and describe any modifications made as a result of their review.*
Humphrey-Murto S, *et al*. *Med Teach* 2017[3]<br>Jünger S, *et al*. *Palliat Med* 2017[4]	Describe the overall process using a flow chart or diagram.
Paré G, *et al. Inf Manag* 2013[10]<br>Niederberger M, *et al. Front Public Health* 2020[15]	Explain how the initial voting items in the consensus were developed.<br>*Could describe e.g. development from empirical analyses, qualitative interviews, advance focus groups, brainstorming, or existing guidelines. Should state who consolidated the information and developed the voting items.*
Boulkedid R, *et al. PLoS One* 2011[5]	Describe the procedure for collecting participants' consent to complete the full consensus process.<br>*Could briefly describe any forms used and how the data were collected and stored.*

Section: References

References

1. Hasson F, Keeney S, McKenna H. Research guidelines for the Delphi survey technique. *J Adv Nurs* 2000;32:1008-15. doi: 10.1046/j.1365-2648.2000.t01-1-01567.x.
2. Humphrey-Murto S, Varpio L, Gonsalves C, et al. Using consensus group methods such as Delphi and Nominal Group in medical education research. *Med Teach* 2017;39:14-9. doi: 10.1080/0142159X.2017.1245856.
3. Humphrey-Murto S, Crew R, Shea B, et al. Consensus Building in OMERACT: Recommendations for Use of the Delphi for Core Outcome Set Development. *J Rheumatol* 2019;46:1041-6. doi: 10.3899/jrheum.181094.
4. Jünger S, Payne SA, Brine J, et al. Guidance on Conducting and REporting DElphi Studies (CREDES) in palliative care: Recommendations based on a methodological systematic review. *Palliat Med* 2017;31(8):684-706. doi: 10.1177/0269216317690685.
5. Boulkedid R, Abdoul H, Loustau M, et al. Using and Reporting the Delphi Method for Selecting Healthcare Quality Indicators: A Systematic Review. *PLoS One* 2011;6:e20476. doi: 10.1371/journal.pone.0020476.
6. Chan TM, Yarris LM, Humphrey-Murto S. Delving into Delphis. *CJEM* 2019;21:167-9. doi: 10.1017/cem.2019.3.
7. Diamond IR, Grant RC, Feldman BM, et al. Defining consensus: A systematic review recommends methodologic criteria for reporting of Delphi studies. *J Clin Epidemiol* 2014;67:401-9. doi: 10.1016/j.jclinepi.2013.12.002.
8. Humphrey-Murto S, Varpio L, Wood TJ, et al. The Use of the Delphi and Other Consensus Group Methods in Medical Education Research: A Review. *Acad Med* 2017;92:1491-8. doi: 10.1097/ACM.0000000000001812.
9. Resemann HK, Clements S, Griffiths A, et al. Reporting of Delphi methods to achieve consensus on guidelines in rare diseases. *Curr Med Res Opin* 2018;34 (Suppl 1):37.
10. Paré G, Cameron AF, Poba-Nzaou P, et al. A systematic assessment of rigor in information systems ranking-type Delphi studies. *Inf Manag* 2013;50:207-17 doi: 10.1016/j.im.2013.03.003.
11. Banno M, Tsujimoto Y, Kataoka Y. Reporting quality of the Delphi technique in reporting guidelines: a protocol for a systematic analysis of the EQUATOR Network Library. *J Clin Epidemiol* 2019;9:e024942.
12. Banno M, Tsujimoto Y, Kataoka Y. The majority of reporting guidelines are not developed with the Delphi method: a systematic review of reporting guidelines. *J Clin Epidemiol* 2020;124;50-7. doi: 10.1016/j.jclinepi.2020.04.010.
13. Gattrell WT, Clements SJ, Sheard D. Quality assessment of guidelines/recommendations developed using Delphi methodology. *Curr Med Res Opin* 2019;35 (Suppl 2):40. doi: 10.1080/03007995.2019.1583496.
14. Ng J. Delphi method: A qualitative approach for quantitative results. *Value Health* 2018;21:S54. Available at https://www.valueinhealthjournal.com/article/S1098-3015(18)30747-2/pdf.
15. Niederberger M, Spranger J. Delphi Technique in Health Sciences: A Map. *Front Public Health* 2020;8:457. doi: 10.3389/fpubh.2020.00457.

Section: References

16. Waggoner J, Carline JD, Durning SJ. Is There a Consensus on Consensus Methodology? Descriptions and Recommendations for Future Consensus Research. *Acad Med* 2016;91:663-8. doi: 10.1097/ACM.0000000000001092.
17. Wang X, Chen Y, Yang N, et al. Methodology and reporting quality of reporting guidelines: systematic review. *BMC Med Res Methodol* 2015;15:74. doi: 10.1186/s12874-015-0069-z.
18. Grant S, Booth M, Khodyakov D. Lack of preregistered analysis plans allows unacceptable data mining for and selective reporting of consensus in Delphi studies. *J Clin Epidemiol* 2018;99:96-105. doi: 10.1016/j.jclinepi.2018.03.007.
