# Supplementary material for: Existing guidance on reporting of consensus methodology: a systematic review to inform ACCORD guideline development
Source: BMJ Open. 2022 Sep 8;12(9):e065154. doi: 10.1136/bmjopen-2022-065154 (PMC9462098; doi:10.1136/bmjopen-2022-065154)
Supplement: Author's manuscript [file bmjopen-2022-065154.draft_revisions.pdf]

BMJ Open is committed to open peer review. As part of this commitment we make the peer review history of every article we publish publicly available.

When an article is published we post the peer reviewers' comments and the authors' responses online. We also post the versions of the paper that were used during peer review. These are the versions that the peer review comments apply to.

The versions of the paper that follow are the versions that were submitted during the peer review process. They are not the versions of record or the final published versions. They should not be cited or distributed as the published version of this manuscript.

BMJ Open is an open access journal and the full, final, typeset and author-corrected version of record of the manuscript is available on our site with no access controls, subscription charges or pay-per-view fees (http://bmjopen.bmj.com).

If you have any questions on BMJ Open's open peer review process please email
info.bmjopen@bmj.com

BMJ Open

Existing guidance on reporting of consensus methodology: a systematic review to inform ACCORD guideline development

Journal:	*BMJ Open*
Manuscript ID	bmjopen-2022-065154
Article Type:	Original research
Date Submitted by the Author:	27-May-2022
Complete List of Authors:	van Zuuren, Esther; Leiden University Medical Center, Department of Dermatology<br>Fedorowicz, Zbys; Veritas Health Sciences Consultancy<br>Price, Amy; Stanford University School of Medicine<br>Logullo, Patricia; University of Oxford, CSM (Centre for Statistics in Medicine)<br>Hughes, Ellen L; Sciwright Limited<br>Gattrell, William T; Ipsen
Keywords:	Protocols & guidelines < HEALTH SERVICES ADMINISTRATION & MANAGEMENT, PUBLIC HEALTH, STATISTICS & RESEARCH METHODS

SCHOLARONE™
Manuscripts

I, the Submitting Author has the right to grant and does grant on behalf of all authors of the Work (as defined in the below author licence), an exclusive licence and/or a non-exclusive licence for contributions from authors who are: i) UK Crown employees; ii) where BMJ has agreed a CC-BY licence shall apply, and/or iii) in accordance with the terms applicable for US Federal Government officers or employees acting as part of their official duties; on a worldwide, perpetual, irrevocable, royalty-free basis to BMJ Publishing Group Ltd ("BMJ") its licensees and where the relevant Journal is co-owned by BMJ to the co-owners of the Journal, to publish the Work in this journal and any other BMJ products and to exploit all rights, as set out in our licence.

The Submitting Author accepts and understands that any supply made under these terms is made by BMJ to the Submitting Author unless you are acting as an employee on behalf of your employer or a postgraduate student of an affiliated institution which is paying any applicable article publishing charge ("APC") for Open Access articles. Where the Submitting Author wishes to make the Work available on an Open Access basis (and intends to pay the relevant APC), the terms of reuse of such Open Access shall be governed by a Creative Commons licence – details of these licences and which Creative Commons licence will apply to this Work are set out in our licence referred to above.

Other than as permitted in any relevant BMJ Author's Self Archiving Policies, I confirm this Work has not been accepted for publication elsewhere, is not being considered for publication elsewhere and does not duplicate material already published. I confirm all authors consent to publication of this Work and authorise the granting of this licence.

For peer review only

Existing guidance on reporting of consensus methodology: a systematic review to inform ACCORD guideline development

Esther J van Zuuren[1], Zbys Fedorowicz[2], Amy Price[3], Patricia Logullo[4], Ellen L Hughes[5], William T Gattrell[6]

[1]Department of Dermatology, Leiden University Medical Centre, Leiden, The Netherlands

[2]Veritas Health Sciences Consultancy, Huntingdon, United Kingdom

[3]Stanford University School of Medicine, Stanford, CA, USA

[4]Centre for Statistics in Medicine (CSM), University of Oxford, and EQUATOR Network UK Centre, Oxford, UK

[5]Sciwright Limited, Somerset, UK

[6]Global Medical Affairs, Ipsen, Abingdon, UK

Corresponding author: Esther J van Zuuren,
Department of Dermatology B1-Q
Leiden University Medical Centre
Albinusdreef 2
2333ZA Leiden
The Netherlands
email: e.j.van_zuuren@lumc.nl

Wordcount: 2081

Keywords: systematic review, consensus, Delphi, reporting guideline

For peer review only

ABSTRACT

Objective: To identify evidence on the quality of reporting of consensus methodology, and to select potential items for a checklist for the ACCORD (ACcurate COnsensus Reporting Document) project to develop a consensus reporting guideline.

Design: Systematic review.

Eligibility criteria for selecting studies: Studies, reviews and published guidance addressing the quality of reporting of consensus methodology that aim to improve health outcomes in biomedicine or clinical practice. Reports of studies using or describing consensus methods but not commenting on their reporting quality were excluded.

Data sources: Embase, MEDLINE, Web of Science, PubMed, Cochrane Library, Emcare, Academic Search Premier and PsycINFO from inception until 7 January 2022.

Data extraction: Screening and data extraction of eligible studies were carried out independently by two authors.

Results: Eighteen studies were included: 5 systematic reviews, 4 narrative reviews, 3 research papers, 3 conference abstracts, 2 research guidance papers and 1 protocol. The majority of studies indicated that the quality of reporting of consensus methodology could be improved. The most commonly addressed items were: the composition of the consensus panel; definition of consensus; and the threshold for achieving that consensus. Items least addressed were: public patient involvement (PPI); the role of the steering committee, chair, co-chair; conflict of interest of panellists; and funding. Data extracted from included studies revealed additional items that were not captured in the data extraction form such as justification of deviation from the protocol or incentives to encourage panellists for responding.

Conclusion: The results of this systematic review confirmed the need for a reporting checklist for consensus methodology and provided a range of potential checklist items that should be reported. The next step in the ACCORD project builds on this systematic review and focuses on reaching consensus on these items to develop the reporting guideline.

Protocol registration: The protocol is registered at https://osf.io/2rzm9.

For peer review only

For peer review only - http://bmjopen.bmj.com/site/about/guidelines.xhtml

STRENGTHS AND LIMITATIONS OF THIS STUDY

- This systematic review utilised a comprehensive search of multiple databases without language restriction
- Included studies ranged from conference abstracts and protocols to guidelines and systematic reviews
- This systematic review highlights the need for a reporting checklist to guide consensus methods
- For full transparency and to promote discussion, all data retrieved are reported as supplemental material
- Conclusions are limited by the paucity of studies that provided substantial useful guidance

For peer review only

For peer review only - http://bmjopen.bmj.com/site/about/guidelines.xhtml

INTRODUCTION

Healthcare providers face continuing challenges in making treatment decisions, particularly where available information on a clinical topic is limited, contradictory, or non-existent. In such situations, alternative and complementary approaches underpinned by collective judgement and based on expert consensus may be used.[1-3]

A variety of approaches with differing methodological rigour can be used to achieve consensus-based decisions. These range from informal "expert consensus meetings" to structured or systematic approaches such as the Delphi method and the Nominal Group Technique (NGT). These methods can be used for generating ideas or determining priorities and aim to achieve consensus through voting on a series of multiple-choice questions.[4-7] The voting process varies according to the method and may take place anonymously (as in Delphi) and/or face to face (in NGT and consensus conferences).[8-10] Key elements in the process include the use of valid and reliable methods to reach consensus and subsequently their transparent reporting; however, these aspects are seldom clearly and explicitly reported.[3, 11]

Reporting guidelines have been developed and are in use for the majority of study designs, e.g. PRISMA, CONSORT and STROBE (for all existing reporting guidelines see: https://www.equator-network.org/). However, no research reporting guideline exists for studies involving consensus methodology other than best practice guidance for Delphi studies in palliative care.[12] Guidelines should include "a checklist, flow diagram, or explicit text to guide authors in reporting a specific type of research, developed using explicit methodology".[3]

Deficiencies in the reporting of consensus methods have been well documented in the literature and are referred to in the protocol for the ACCORD (ACcurate COnsensus Reporting Document) project, which aims to develop a reporting guideline for methods used to reach consensus.[13] In accordance with the EQUATOR Network guidance in the toolkit for the development of reporting guidelines, the

For peer review only - http://bmjopen.bmj.com/site/about/guidelines.xhtml

next step for the ACCORD project was a review of the relevant literature, which would ultimately inform the voting process.[3]

Our objective was to undertake a thorough and comprehensive systematic review that seeks to identify evidence on the quality of reporting of consensus methodology, for subsequent development into a draft checklist of items for the ACCORD guideline. This ACCORD reporting guideline will assist the biomedical research and clinical practice community to describe the methods used to reach consensus in a complete, transparent, and consistent manner.

METHODS

This systematic review conforms to the Preferred Reporting Items for Systematic Reviews and Meta-Analyses (PRISMA) statement,[14] and followed a prespecified protocol (Supplementary Material 1).[13] The protocol was initially registered on 12 October 2021 at the Open Science Foundation (OSF).[15]

Inclusion criteria

Eligible studies consisted of reviews and published guidance which addressed the quality of reporting of consensus methodology and aimed to improve health outcomes in biomedicine or clinical practice.

Exclusion criteria

Excluded were publications using consensus methods or describing consensus methods, but which did not comment on their reporting quality. Examples include guidelines developed through the use of consensus methodology such as reporting guidelines, clinical practice guidelines or core outcome set development studies. Editorials, letters about individual publications, and commentaries on consensus methods outside the scope of biomedical research (for example in the social sciences, economy, politics or marketing) were also considered ineligible.

Literature search strategy

For peer review only

A systematic literature search was conducted on 7 January 2022 by a biomedical information specialist. The following bibliographical databases were searched: MEDLINE (OVID version), Embase (OVID version), PubMed, Web of Science, MEDLINE (Web of Science), Cochrane Library, Emcare (OVID version), PsycINFO (EbscoHOST version) and Academic Search Premier. The full search strategy is presented in Supplementary Material 2. No language restrictions were applied.

We (EJvZ, ZF, PL and WTG) piloted four initial search strategies provided by the information specialist (JWS see Acknowledgements). The initial search strategy was sensitive and precise, producing the highest number of retrieved references (N = 7951). After several rounds of checking through known relevant references and controlling for the effect of the performance of certain search terms, modifications were made, including the use of the most explicit terms in the most specific search fields. The performance of search terms was investigated from two vantage points: homonymy (same search term, but different meaning), and, particularly, loss-of-context (right meaning of the word, but not in the correct context). This extended search strategy provided extra 'signal', but also reduced the level of 'noise'. We chose to keep the search terms broad (in not using the singular terms "delphi" and "consensus" but always in phrases or with other contextual words). In this way, the refined, broad search strategy was better aligned with our inclusion criteria and the objectives of the systematic review.

The final search results were uploaded to Rayyan (https://rayyan.ai) in the blind mode for independent screening by four review authors (EJvZ, ZF, PL, WTG) based on titles and abstracts. Records that were deemed eligible or when there was insufficient detail to make a clear judgement were retrieved as full-text articles (EJvZ). The same four reviewers independently reassessed the eligibility of these full-text papers and any discrepancies were resolved through discussion. The references of the included studies were also checked for additional potentially eligible studies (EJvZ).

Data extraction

For peer review only - http://bmjopen.bmj.com/site/about/guidelines.xhtml

Study details and outcome data from the included studies were collected independently within Covidence (https://www.covidence.org/) by two authors using a piloted data extraction form (EJvZ, WTG). Disagreements were discussed and reconciled by consultation with a third and fourth author (ZF and AP).

The following details were extracted: bibliographic details and potential checklist items. Checklist items were divided into the component parts of background, methods, results and discussion, each addressing key aspects of consensus methodology. We also included a section for additional items retrieved from the studies that were not captured in our data extraction form. The complete data extraction form can be found as Supplementary Material 3.

Patient and public involvement

We involved patients, advocates, and members of the lay public in the initial phases of this protocol [13, 15], as collaborators to develop this project and to co-produce the systematic review and co-author the manuscript. They are cooperating by offering their experience with the use of consensus methods to develop guidelines and also systematic reviews. These contributors will be invited to work with us to disseminate the results.

RESULTS

Our searches across the databases identified 2599 articles and 137 further references to abstracts totaling 2736 references (after removal of duplicates) (see figure 1). A total of 2682 records were excluded after examination of titles and abstracts. Full-text copies of 54 studies were obtained for further assessment of eligibility and finally just 18 were included. Checking of the references of these full-text publications did not yield any additional eligible articles.

Characteristics of included studies

Eighteen studies matched our prespecified eligibility criteria and were finally included. These studies comprised five systematic reviews,[12, 16-19] four reviews,[20-23] three research papers,[24-26] two research guidelines/guidance,[27, 28] three conference abstracts,[29-31] and one protocol.[32]

For peer review only - http://bmjopen.bmj.com/site/about/guidelines.xhtml

Characteristics of excluded studies

A total of 36 studies were excluded.[33- 68] The main reasons for their exclusion were: that they discussed (modified) Delphi methodology but did not include aspects of reporting;[33-54] that they covered reporting but not on consensus methodology;[55-58] that various consensus methodologies were discussed but not their reporting;[59-67] and that the concept of experts in consensus methodology was discussed.[68]

Data extraction

The majority of studies indicated that reporting of consensus methods could be improved and summarised current limitations in reporting or proposed suggestions for improvement.

In Table 1, we have summarised the results of the data extraction, which correlate the specific studies with the corresponding aspects of consensus reporting ("items") they address, and are presented in the format used in the data extraction form (see Supplementary Material 3).

For peer review only

For peer review only - http://bmjopen.bmj.com/site/about/guidelines.xhtml

Table 1. Potential checklist items retrieved from the included studies

Reporting Items	Studies that provide guidance	
Background	Number	References
1.1 Rationale for choosing a consensus method over other methods	4	[12, 25, 27, 28]
1.2 Clearly defined objective	6	[12, 17, 18, 20, 27, 28]
Methods		
2.1 Review of existing evidence informing consensus study	5	[20, 21, 27, 28, 31]
2.2 Inclusion and exclusion criteria of the literature search	3	[17, 20, 22]
2.3 Composition of the panel	16	[12, 16-23, 25-30, 32]
2.4 Public patient involvement (PPI)	0	
2.5 Panel recruitment	4	[12, 17, 22, 23]
2.6 Defining consensus and the threshold for achieving consensus	13	[12, 17-21, 23-29]
2.7 Decision of item approval	3	[12, 17, 27]
2.8 Number of voting rounds	10	[12, 16, 18, 20, 21, 23, 26-28, 32]
2.9 Rationale for number of voting rounds	8	[16, 20, 21-23, 25, 26, 28]
2.10 Time between voting rounds	1	[17]
2.11 Additional methods used alongside consensus	2	[17, 23]
2.12 Software or tools used for voting	1	[25]
2.13 Anonymity of panellists and how this was maintained	7	[16, 20-22, 25, 28, 29]
2.14 Feedback to panellists at the end of each round	11	[17, 19-22, 25-29, 31]
2.15 Synthesis/analysis of responses after voting rounds	5	[12, 22-24, 30]
2.16 Pilot testing of study material/instruments	3	[12, 22, 28]
2.17 Role of the steering committee/chair/co-chair/facilitator	0	
2.18 Conflict of interest or funding received	4	[12, 29, 30, 32]
2.19 Measures to avoid influence by conflict of interest	1	[12]
Results		
3.1 Results of the literature search	1	[12]
3.2 Number of studies found as supporting evidence	0	
3.3 Response rates per voting round	5	[12, 21, 22, 25, 30]
3.4 Results shared with respondents	9	[12, 17, 20, 25-28, 30, 31]
3.5 Dropped items	5	[12, 16, 18, 26, 32]
3.6 Collection, synthesis and comments from panellists	5	[12, 17, 22, 28, 31]
3.7 Final list of items (e.g. for guideline or reporting guideline)	4	[12, 22, 30, 31]
Discussion		
4.1 Limitations and strengths of the study	5	[12, 20, 25, 27, 28]
4.2 Applicability, generalizability, reproducibility	3	[12, 17, 26]

The most frequently addressed item in the included studies was the composition of and the criteria for selecting the panellists, including their demographics; specifically age, gender, specialty, years of experience, and sociodemographic background. Equally addressed were the aspects of clarity in, and the importance of, defining consensus and the corresponding thresholds to reach that consensus.

The prespecified number of voting rounds and provision of feedback to the panellists at the end of each round was addressed in half of the studies. None of the included studies reported or made reference to public patient involvement (PPI). The roles of the steering committee/chair/co-chair

For peer review only - http://bmjopen.bmj.com/site/about/guidelines.xhtml

were not defined in any of the included studies. Reporting of the time interval between voting rounds, panel member's conflicts of interest (COI) and funding as well as the measures used to avoid the influence of COI on voting and decision-making were minimally addressed. Conversely, three studies addressed between 12 and 19 of the items in the data extraction form,[12, 19, 28] whereas two studies covered only two or three items.[19, 24] We identified a considerable number of other aspects of reporting that were proposed in the included studies, but which were not captured in our data extraction form. These included: 'justifications for deviating from the protocol', 'incentives for encouraging panellists to respond', and 'suggestions to add a flow chart of the process'. All extracted data can be found in Supplementary Material 4 and 5.

DISCUSSION

Although consensus methodology is widely used in healthcare and researchers have raised poor reporting as an issue, we were only able to identify 18 studies that provided suggestions to improve the quality of reporting of consensus methodology. However, a few studies were particularly informative. The first was a systematic review on the use and reporting of the Delphi method for selecting healthcare indicators.[17] Specifically, this review not only provided guidance for planning and using the Delphi procedure, but additionally formulated recommendations for reporting. The second study was a guidance report on consensus methods such as Delphi and NGT, which were used in medical education research.[28] The authors reported that there is a lack of "standardization in definitions, methodology and reporting" and proposed items for researchers to consider when using consensus methods to improve methodological rigour as well as the reporting quality. The third study we would like to highlight is the Guidance on Conducting and Reporting DElphi Studies (CREDES) in palliative care, which was based on a methodological systematic review.[12] This study focused on the development of guidance in palliative care and may not be suitable for extrapolation to other biomedical areas. Furthermore, this study, only considered Delphi methodology, whereas we included studies covering consensus processes involving non-Delphi based methods or "modified Delphi". However, many of the suggestions made regarding the design and conduct of Delphi studies

For peer review only - http://bmjopen.bmj.com/site/about/guidelines.xhtml

in addition to recommendations for reporting are equally applicable to our ACCORD project. These items will be used and integrated into the next step of the project, which is the development of a reporting checklist on consensus methods.

Two additional studies proved to be of value.[21, 25] One provided a preliminary Delphi checklist to be used for Outcome Measures in Rheumatology (OMERACT).[25] The other concluded in a scoping review that consensus methods are "poorly standardized and inconsistently used" and exposed reporting flaws in consensus reports.[21]

Both composition and characteristics of the panel, and defining consensus and threshold for achieving assessment received were consistently addressed and appeared to be critical items that should be reported in sufficient detail. Feedback to the panel might be considered an important aspect of ensuring ongoing engagement with the panels; thus it was somewhat surprising to see slightly more than half of the studies consider this an element of consensus methodology worth reporting.

Some items were not addressed in any of the studies, specifically PPI, which is currently considered a key element in the shared decision-making process and is a component of guideline development.[69] Just four studies made reference to the COI of panel members and project funding. COI of panellists, as well as of chair, co-chair and steering committee, can directly impact and influence decision-making during the various steps of consensus methodology. As such, COI remains underreported and is often inconsistently described.[70] This also raises concerns about the measures that can be taken to mitigate the potential influence of COI and to ensure that those panellists who do have relevant interests are, for example, not able to vote on pertinent items.

CONCLUSION

The principal objectives of this systematic review were to conduct a comprehensive search and to identify the existing evidence on the quality of reporting of consensus methodology. As such we

For peer review only - http://bmjopen.bmj.com/site/about/guidelines.xhtml

have been able to gather together a comparatively small number of relevant studies, to summarise the existing research, and to highlight key gaps in the current evidence base. This systematic review will ultimately inform the generation of a draft checklist of items for the ACCORD reporting guideline.

ACKNOWLEDGMENTS

We would like to thank Jan W. Schoones of the Directorate of Research Policy (formerly: Walaeus Library), Leiden University Medical Centre, Leiden, The Netherlands, for his assistance in the development of the search strategy. Furthermore, we would like to thank the other members of the ACCORD project Steering Committee for their contributions to the protocol: Amrit Pali Hungin, Christopher C Winchester, David Tovey, Keith Goldman, Rob Matheis and Niall Harrison.

COMPETING INTERESTS

PL is a member of the UK EQUATOR Centre, an organization that promotes the use of reporting guidelines, many of which are developed using consensus methods, and she is personally involved in the development of other reporting guidelines. ELH has worked with Ogilvy Health UK on consensus projects. WG is a former employee of Ipsen and is now employed by Bristol Myers Squib. EJvZ, ZF and AP have no conflict of interest.

FUNDING

Apart from PL and WG, who contributed their time with the agreement of their employers, this study was conducted without external funding for the development of the study design; for the collection, analysis and interpretation of the data; or for the writing of the report. As ZF (Veritas Health Sciences Consultancy) acted as an independent consultant for this work, his support for data extraction and tabulation was funded by Oxford PharmaGenesis. Support for Open Access was also provided by Oxford PharmaGenesis. The decision to submit the paper for publication was made solely by the authors.

CONTRIBUTORS

For peer review only - http://bmjopen.bmj.com/site/about/guidelines.xhtml

EJvZ, ZF, PL, and WTG contributed to the screening and agreed on the inclusion of studies. EJvZ and WTG extracted data from the included studies. ZF, AP and ELH contributed to the discussion of extracted data and interpretation. EJvZ was the major contributor in the review of studies, data extraction, interpretation of findings as well as writing the manuscript. All authors read the final manuscript, provided feedback and approved the final manuscript. The author EJvZ is the guarantor.

PATIENT CONSENT FOR PUBLICATION

No patient data were used in this study and no patient consent for publication was required.

ETHICS APPROVAL

No patient-level data were used in this study and no ethical approval was sought.

PROVENANCE AND PEER REVIEW

Not commissioned; externally peer reviewed.

DATA AVAILABILITY STATEMENT

All key data for this study are included in this article or uploaded as online supplementary information. The ACCORD protocol has been listed on the EQUATOR website (Reporting guidelines under development for other study designs | The EQUATOR Network (equator-network.org)) and registered with the Open Science Framework (https://osf.io/2rzm9).

Open access

This is an open access article distributed in accordance with the terms of the Creative Commons Attribution (CC BY 4.0) license, which permits others to distribute, remix, adapt and build upon this work, for commercial use, provided the original work is properly cited. See: http://creativecommons.org/licenses/by/4.0.

For peer review only - http://bmjopen.bmj.com/site/about/guidelines.xhtml

For peer review only

ORCID iDs

EJvZ: https://orcid.org/0000-0002-4780-0182

ZF: https://orcid.org/0000-0003-3952-965X

AP: https://orcid.org/0000-0002-5200-7322

PL: https://orcid.org/0000-0001-8708-7003

ELH: https://orcid.org/0000-0003-2012-0037

WG: https://orcid.org/0000-0002-9733-6330

REFERENCES

1. Raine R, Sanderson C, Black N. Developing clinical guidelines: a challenge to current methods. *BMJ* 2005;331:631-3. doi: 10.1136/bmj.331.7517.631.
2. Djulbegovic B, Guyatt G. Evidence vs Consensus in Clinical Practice Guidelines. *JAMA* 2019;322:725-6.doi: 10.1001/jama.2019.9751.
3. Moher D, Schulz KF, Simera I, Altman DG. Guidance for developers of health research reporting guidelines. *PLoS Med* 2010;7:e1000217. doi:10.1371/journal.pmed.1000217.
4. Dalkey N, Helmer O. An Experimental Application of the DELPHI Method to the Use of Experts. Manage Sci. 1963;9:458-467. https://doi.org/10.1287/mnsc.9.3.458.
5. Murphy MK, Black NA, Lamping DL, et al. Consensus development methods, and their use in clinical guideline development. *Health Technol Assess* 1998;2:i-iv, 1-88. doi: 10.3310/hta2030.
6. Foth T, Efstathiou N, Vanderspank-Wright B, et al. The use of Delphi and Nominal Group Technique in nursing education: A review. *Int J Nurs Stud* 2016;60:112-20. doi: 10.1016/j.ijnurstu.2016.04.015.
7. Nair R, Aggarwal R, Khanna D. Methods of formal consensus in classification/diagnostic criteria and guideline development. *Semin Arthritis Rheum* 2011;41:95-105. doi: 10.1016/j.semarthrit.2010.12.001.
8. McMillan SS, King M, Tully MP. How to use the nominal group and Delphi techniques. *Int J Clin Pharm* 2016;38:655-62. doi: 10.1007/s11096-016-0257-x.
9. Grant S, Armstrong C, Khodyakov D. Online Modified-Delphi: a Potential Method for Continuous Patient Engagement Across Stages of Clinical Practice Guideline Development. *J Gen Intern Med* 2021;36:1746-50. doi: 10.1007/s11606-020-06514-6.
10. Helmer-Hirschberg O. Analysis of the future: The Delphi Method. Santa Monica, CA: RAND Corporation, 1967. Available at https://www.rand.org/pubs/papers/P3558.htm.
11. Arakawa N, Bader LR Consensus development methods: Considerations for national and global frameworks and policy development. *Res Social Adm Pharm* 2022;18:2222-9. doi: 10.1016/j.sapharm.2021.06.024.

For peer review only - http://bmjopen.bmj.com/site/about/guidelines.xhtml

12. Jünger S, Payne SA, Brine J, et al. Guidance on Conducting and REporting DElphi Studies (CREDES) in palliative care: Recommendations based on a methodological systematic review. *Palliat Med* 2017;31(8):684-706. doi: 10.1177/0269216317690685.
13. Gattrell WT, Hungin AP, Price A, et al. ACCORD guideline for reporting consensus-based methods in biomedical research and clinical practice: a study protocol. *Res Integr Peer Rev* May 09, 2022. https://doi.org/10.1186/s41073-022-00122-0.
14. Page MJ, McKenzie JE, Bossuyt PM, et al. The PRISMA 2020 statement: an updated guideline for reporting systematic reviews. *BMJ* 2021;372:n71. doi: 10.1136/bmj.n71.
15. Gattrell W, Hungin AP, Arents BWMA, et al. Consensus-based methods in biomedical research and clinical practice: The ACCORD study protocol for establishing a reporting guideline. https://osf.io/e5w9n/ (accessed 19 April 2022).
16. Banno M, Tsujimoto Y, Kataoka Y. The majority of reporting guidelines are not developed with the Delphi method: a systematic review of reporting guidelines. *J Clin Epidemiol* 2020;124;50-7. doi: 10.1016/j.jclinepi.2020.04.010.
17. Boulkedid R, Abdoul H, Loustau M, et al. Using and Reporting the Delphi Method for Selecting Healthcare Quality Indicators: A Systematic Review. *PLoS One* 2011;6:e20476. doi: 10.1371/journal.pone.0020476.
18. Diamond IR, Grant RC, Feldman BM, et al. Defining consensus: A systematic review recommends methodologic criteria for reporting of Delphi studies. *J Clin Epidemiol* 2014;67:401-9. doi: 10.1016/j.jclinepi.2013.12.002.
19. Wang X, Chen Y, Yang N, et al. Methodology and reporting quality of reporting guidelines: systematic review. *BMC Med Res Methodol* 2015;15:74. doi: 10.1186/s12874-015-0069-z
20. Chan TM, Yarris LM, Humphrey-Murto S. Delving into Delphis. *CJEM* 2019;21:167-9. doi: 10.1017/cem.2019.3.
21. Humphrey-Murto S, Varpio L, Wood TJ, et al. The Use of the Delphi and Other Consensus Group Methods in Medical Education Research: A Review. *Acad Med* 2017;92:1491-8. doi: 10.1097/ACM.0000000000001812.
22. Paré G, Cameron AF, Poba-Nzaou P, et al. A systematic assessment of rigor in information systems ranking-type Delphi studies. *Inf Manag* 2013;50:207-17 doi: 10.1016/j.im.2013.03.003.
23. Waggoner J, Carline JD, Durning SJ. Is There a Consensus on Consensus Methodology? Descriptions and Recommendations for Future Consensus Research. *Acad Med* 2016;91:663-8. doi: 10.1097/ACM.0000000000001092.
24. Grant S, Booth M, Khodyakov D. Lack of preregistered analysis plans allows unacceptable data mining for and selective reporting of consensus in Delphi studies. *J Clin Epidemiol* 2018;99:96-105. doi: 10.1016/j.jclinepi.2018.03.007.
25. Humphrey-Murto S, Crew R, Shea B, et al. Consensus Building in OMERACT: Recommendations for Use of the Delphi for Core Outcome Set Development. *J Rheumatol* 2019;46:1041-6. doi: 10.3899/jrheum.181094.
26. Niederberger M, Spranger J. Delphi Technique in Health Sciences: A Map. *Front Public Health* 2020;8:457. doi: 10.3389/fpubh.2020.00457.
27. Hasson F, Keeney S, McKenna H. Research guidelines for the Delphi survey technique. *J Adv Nurs* 2000;32:1008-15. doi: 10.1046/j.1365-2648.2000.t01-1-01567.x.

For peer review only - http://bmjopen.bmj.com/site/about/guidelines.xhtml

28. Humphrey-Murto S, Varpio L, Gonsalves C, et al. Using consensus group methods such as Delphi and Nominal Group in medical education research. *Med Teach* 2017;39:14-9. doi: 10.1080/0142159X.2017.1245856.
29. Gattrell WT, Clements SJ, Sheard D. Quality assessment of guidelines/recommendations developed using Delphi methodology. *Curr Med Res Opin* 2019;35 (Suppl 2):40. doi: 10.1080/03007995.2019.1583496.
30. Ng J. Delphi method: A qualitative approach for quantitative results. *Value Health* 2018;21:S54. Available at https://www.valueinhealthjournal.com/article/S1098-3015(18)30747-2/pdf.
31. Resemann HK, Clements S, Griffiths A, et al. Reporting of Delphi methods to achieve consensus on guidelines in rare diseases. *Curr Med Res Opin* 2018;34 (Suppl 1):37.
32. Banno M, Tsujimoto Y, Kataoka Y. Reporting quality of the Delphi technique in reporting guidelines: a protocol for a systematic analysis of the EQUATOR Network Library. *J Clin Epidemiol* 2019;9:e024942.
33. Bellali T, Karamitri I. The Delphi research methodology and its applications in the healthcare sciences. *Arch Hellen Med* 2011;28:839-48. Available at http://www.mednet.gr/archives/2011-6/pdf/839.pdf.
34. Bijl R. Delphi in a future scenario study on mental health and mental health care. *Futures* 1992;24:232-50. doi: 10.1016/0016-3287(92)90033-C.
35. Boel A, Navarro-Compán V, Landewé R, et al. Two different invitation approaches for consecutive rounds of a Delphi survey led to comparable final outcome. *J Clin Epidemiol* 2021;129:31-9. doi: 10.1016/j.jclinepi.2020.09.034.
36. Bowles N. The Delphi technique. *Nurs Stand* 1999;13:21-6. doi: 10.7748/ns1999.07.13.45.32.c2650.
37. Cramer CK, Klasser GD, Epstein JB, et al. The Delphi process in dental research. *J Evid Based Dent Pract* 2008;8:211-20. doi: 10.1016/j.jebdp.2008.09.002.
38. de Loë R, Melnychuk N, Murray D, et al. Advancing the State of Policy Delphi Practice: A Systematic Review Evaluating Methodological Evolution, Innovation, and Opportunities. *Technol Forecast Soc Change* 2016;104:78-88. doi: 10.1016/j.techfore.2015.12.009.
39. Drumm S, Bradley C, Moriarty F. 'More of an art than a science'? The development, design and mechanics of the Delphi Technique. *Res Social Adm Pharm* 2022;18:2230-6. doi: 10.1016/j.sapharm.2021.06.027.
40. Fink-Hafner D, Dagan T, Doušak M, et al. Delphi Method: Strengths and Weaknesses. *Metodoloski Zv* 2019;16:1-19. doi: 10.51936/fcfm6982.
41. Franklin KK, Hart JK. Idea Generation and Exploration: Benefits and Limitations of the Police Delphi Research Method. *Innov High Educ* 2006;31:237-46. 10.1007/s10755-006-9022-8.
42. Guzys D, Dickson-Swift V, Kenny A, et al. Gadamerian philosophical hermeneutics as a useful methodological framework for the Delphi technique. *Int J Qual Stud Health Well-being* 2015;10:26291. doi: 10.3402/qhw.v10.26291.
43. Hallowell MR, Gambatese JA. Qualitative Research: Application of the Delphi Method to CEM Research. *J Constr Eng Manag* 2010;136:99-107. doi: 0.1061/(ASCE)CO.1943-7862.0000137.
44. Holloway K. Doing the E-Delphi: using online survey tools. *Comput Inform Nurs* 2012;30:347-50. doi: 10.1097/NXN.0b013e31825e8923.

For peer review only - http://bmjopen.bmj.com/site/about/guidelines.xhtml

45. Hung HL, Altschuld JW, Lee YF. Methodological and conceptual issues confronting a cross-country Delphi study of educational program evaluation. *Eval Program Plann* 2008;31:191-8. doi: 10.1016/j.evalprogplan.2008.02.005.
46. Ibiyemi AO, Adnan YM, Daud MN. The validity of the classical Delphi applications for assessing the industrial sustainability-correction factor: an example study. *Foresight* 2016;18:603-24. doi: 10.1108/FS-04-2016-0016.
47. Keeney S, Hasson F, McKenna HP. Consulting the oracle: ten lessons from using the Delphi technique in nursing research. *J Adv Nurs* 2006;53:205-12. doi: 10.1111/j.1365-2648.2006.03716.x.
48. Keeney S, Hasson F, McKenna HP. A critical review of the Delphi technique as a research methodology for nursing. *Int J Nurs Stud* 2001;38:195-200. doi: 10.1016/S0020-7489(00)00044-4.
49. Kennedy HR. Enhancing Delphi research: methods and results. *J Adv Nurs* 2004;45:504-11. doi: 10.1046/j.1365-2648.2003.02933.x.
50. Khodyakov D, Grant S, Denger B, et al. Practical Considerations in Using Online Modified-Delphi Approaches to Engage Patients and Other Stakeholders in Clinical Practice Guideline Development. *Patient* 2020;13:11-21. doi: 10.1007/s40271-019-00389-4.
51. Murry J, Hammons J. Delphi: A versatile methodology for conducting qualitative research. *Rev High Educ* 2017;18:423-36. doi: 10.1353/rhe.1995.0008.
52. Olsen AA, Wolcott MD, Haines ST, et al. How to use the Delphi method to aid in decision making and build consensus in pharmacy education. *Curr Pharm Teach Learn* 2021;13:1376-85. doi: 10.1016/j.cptl.2021.07.018.
53. Vernon W. The Delphi technique: A review. *Int J Ther Rehabil* 2009;16:69-76. doi: 10.12968/ijtr.2009.16.2.38892.
54. Von der Gracht HA. Consensus measurement in Delphi studies Review and implications for future quality assurance. Technol Forecast Soc Change 2012;79:1525-36. doi: 10.1016/j.techfore.2012.04.013.
55. Bennett C, Khangura S, Brehaut JC, et al. Reporting guidelines for survey research: an analysis of published guidance and reporting practices. *PloS Med* 2010;8:e1001069. doi: 10.1371/journal.pmed.1001069.
56. Han SJ, Oh MC, Sughrue ME, et al. Reporting standard compliance in publications of vestibular schwannoma patients treated with microsurgery. *Otol Neurotol* 2015;2012:33:648-50. doi: 10.1097/MAO.0b013e31824f998b.
57. Moher D, Weeks L, Ocampo M, et al. Describing reporting guidelines for health research: a systematic review. *J Clin Epidemiol* 2011;64:718-42. doi: 10.1016/j.jclinepi.2010.09.013.
58. Murray A, Junge A, Robinson PG, et al. International consensus statement: methods for recording and reporting of epidemiological data on injuries and illnesses in golf. Br J Sports Med 2020;54:1136-41. doi: 10.1136/bjsports-2020-102380.
59. Campbell SM, Cantrill JA. Consensus methods in prescribing research. *J Clin Pharm Ther* 2001;26:5-14. doi: 10.1046/j.1365-2710.2001.00331.x.
60. Durkin J, Usher K, Jackson D. Using consensus from experts to inform a shared understanding of subjective terms. *Nurse Res* 2019;27:46-9. doi: 10.7748/nr.2019.e1622.
61. Fink A, Kosecoff J, Chassin M, et al. Consensus methods: characteristics and guidelines for use. *Am J Public Health* 1984;74:979-83. doi: 0.2105/ajph.74.9.979.

For peer review only - http://bmjopen.bmj.com/site/about/guidelines.xhtml

62. Halcomb E, Davidson P, Hardaker L. Using the consensus development conference method in healthcare research. *Nurs Res* 2008;16:56-71. doi: 10.7748/nr2008.10.16.1.56.c6753.
63. James D, Warren-Forward H. Research methods for formal consensus development. *Nurs Res* 2015;22:35-40. doi: 10.7748/nr.22.3.35.e1297.
64. McMillan SS, King M, Tully MP. How to use the nominal group and Delphi techniques. *Int J Clin Pharm* 2016;38:655-62. doi: 10.1007/s11096-016-0257-x.
65. Nair R, Aggarwal R, Khanna D. Methods of Formal Consensus in Classification/Diagnostic Criteria and Guideline Development. *Semin Arthritis Rheum* 2011;41:95-105. doi: 10.1016/j.semarthrit.2010.12.001.
66. Rotondi AJ, Kvetan V, Carlet J, et al. Consensus conferences in critical care medicine - Methodologies and impact. *Crit Care Clin* 1997;13:417-39. doi: 10.1016/s0749-0704(05)70319-5.
67. van Teijlingen E, Pitchforth E, Bishop C, et al. Delphi method and nominal group technique in family planning and reproductive health research. *J Fam Plann Reprod Health Care* 2006;32:249-52. doi: 10.1783/147118906778586598.
68. Baker J, Lovell K, Harris N. How expert are the experts? An exploration of the concept of 'expert' within Delphi panel techniques. *Nurs Res* 2006;14:59-70. doi: 10.7748/nr2006.10.14.1.59.c6010.
69. National Institute for Health and Care Excellence. NICE Guideline [NG197]: Shared decision making. Available at: https://www.nice.org.uk/guidance/ng197.
70. Dunn AG, Coiera E, Mandl KD, et al. Conflict of interest disclosure in biomedical research: A review of current practices, biases, and the role of public registries in improving transparency. *Res Integr Peer Rev* 2016;1:1. doi: 10.1186/s41073-016-0006-7.

Figure Legends

Figure 1
Caption: PRISMA 2020 flow diagram for new systematic reviews which included searches of databases, registers and other sources

For peer review only

For peer review only - http://bmjopen.bmj.com/site/about/guidelines.xhtml

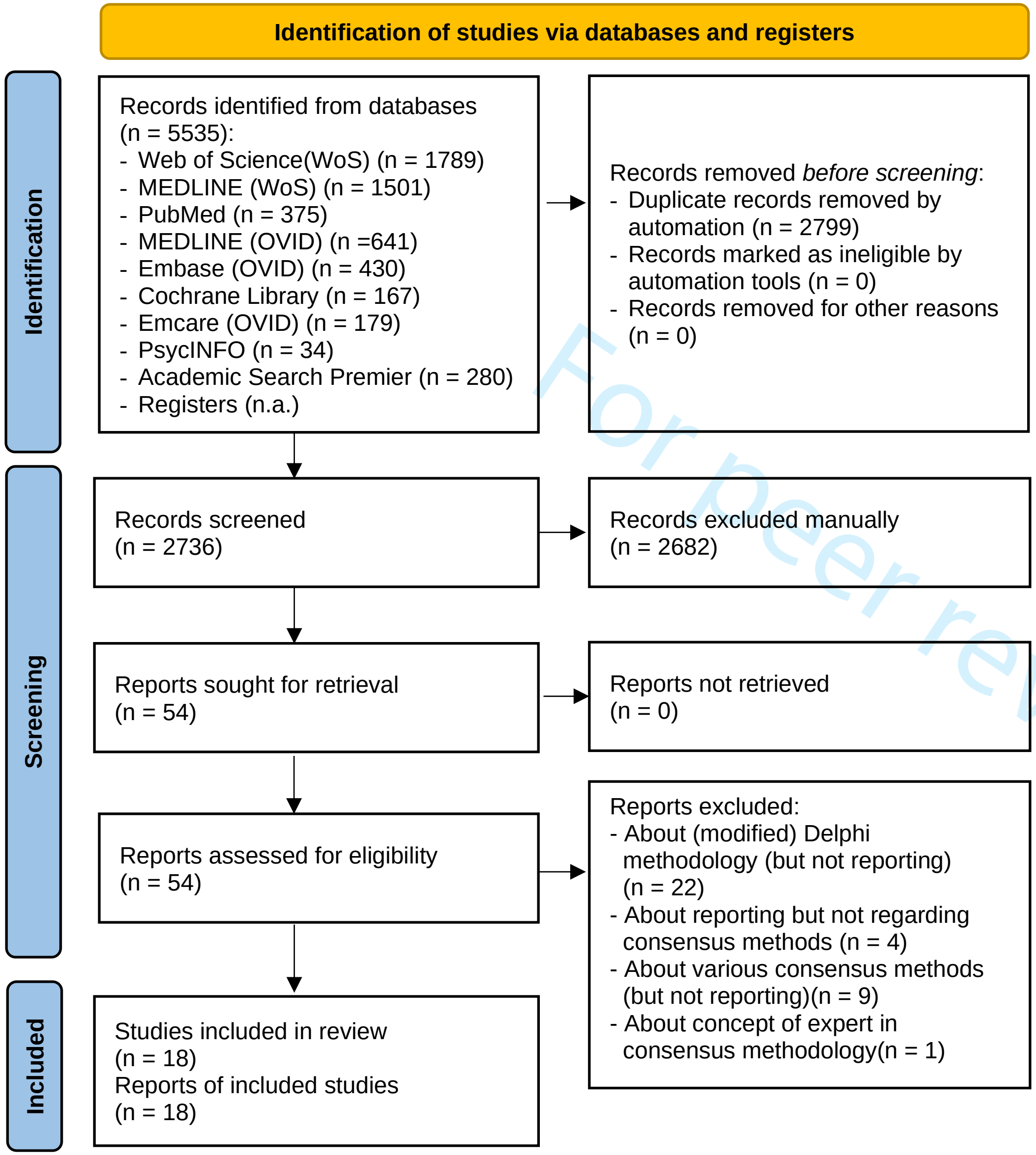

For peer review only

ACCORD guideline for reporting consensus-based methods in biomedical research and clinical practice: a study protocol

William T. Gattrell,[1] Amrit Pali Hungin,[2] Amy Price,[3] Christopher C. Winchester,[4] David Tovey,[5] Ellen L. Hughes,[6] Esther J van Zuuren,[7] Keith Goldman,[8] Patricia Logullo,[9] Rob Matheis,[10] Niall Harrison,[11]

Affiliations: 1. Global Medical Affairs, Ipsen, Abingdon, UK. 2. Faculty of Medical Sciences, Newcastle University, Newcastle, UK. 3. Stanford Anesthesia, Informatics and Media Lab, Stanford University School of Medicine, Stanford, CA, USA. 4. Oxford PharmaGenesis, Tubney, Oxford, UK. 5. Journal of Clinical Epidemiology, London, UK. 6. Sciwright Limited, Somerset, UK. 7. Leiden University Medical Centre, Leiden, The Netherlands. 8. Global Medical Affairs, AbbVie, North Chicago, IL, USA. 9. Centre for Statistics in Medicine (CSM), University of Oxford, and EQUATOR Network UK Centre, Oxford, UK. 10. ISMPP, USA. 11. Ogilvy Health, London, UK.

Corresponding author: William Gattrell

Email address: wgattrell@yahoo.co.uk

ORCID IDs:

CCW https://orcid.org/0000-0003-3267-3990

PL https://orcid.org/0000-0001-8708-7003

KG: https://orcid.org/0000-0002-5497-8413

DT: https://orcid.org/0000-0002-8889-9246

For peer review only

EJvZ: https://orcid.org/0000-0002-4780-0182

WG: https://orcid.org/0000-0002-9733-6330

[AP] https://orcid.org/0000-0002-5200-7322

Current word count: 3196

For peer review only

ABSTRACT [345 words, max 350 words]

Background: Structured, systematic methods to formulate consensus recommendations, such as the Delphi process or Nominal Group Technique, among others, provide the opportunity to harness the knowledge of experts to support clinical decision making in areas of uncertainty. They are widely used in biomedical research, in particular where disease characteristics or resource limitations mean that high-quality evidence generation is difficult. However, poor reporting of methods used to reach a consensus – for example, not clearly explaining the definition of consensus, or not stating how consensus group panellists were selected – can potentially undermine confidence in this type of research and hinder reproducibility. Our objective is therefore to systematically develop a reporting guideline to help the biomedical research and clinical practice community describe the methods or techniques used to reach consensus in a complete, transparent, and consistent manner.

Methods: The ACCORD (ACcurate COnsensus Reporting Document) project will take place in five phases and follow the EQUATOR Network guidance for the development of reporting guidelines. In Stage 1, a multidisciplinary Steering Committee has been established to lead and coordinate the guideline development process. In Stage 2, a systematic literature review will identify evidence on the quality of the reporting of consensus methodology, to obtain potential items for a reporting checklist. In Stage 3, Delphi methodology will be used to reach consensus regarding the checklist items, first among the Steering Committee, and then among a broader Delphi panel comprising participants with a range of expertise, including patient representatives. In Stage 4, the reporting guideline will be finalised in a consensus meeting, along with the production of an Explanation and Elaboration (E&E) document. In Stage 5, we plan to publish the reporting guideline and E&E document in open-access journals, supported by presentations at

For peer review only - http://bmjopen.bmj.com/site/about/guidelines.xhtml

appropriate events. Dissemination of the reporting guideline, including a website linked to social media channels, is crucial for the document to be implemented in practice.

Discussion: The ACCORD reporting guideline will provide a set of minimum items that should be reported about methods used to achieve consensus, including approaches ranging from simple unstructured opinion gatherings to highly structured processes.

Author Keywords:

Methodology, Guidelines, Reporting quality, Reporting completeness, Checklist, Delphi technique, Consensus, Nominal Group Technique

MeSH Terms:

Methods; Consensus; Consensus development conference; Delphi technique

For peer review only

BACKGROUND

Evidence-based medicine relies on three factors: current best evidence based on clinical and real-world studies, individual clinical expertise, and the desires of the patient [1]. Clinical data gathered from systematic reviews, high-quality randomised clinical trials, and observational studies have complementary roles in generating robust evidence [2, 3]. However, healthcare providers face difficult treatment decisions if the available information on a subject is inadequate, contradictory, limited, or does not exist.

The COVID-19 pandemic has brought this situation of lack of evidence into stark relief, as crucial decisions have to be made during any rapidly emerging public health crisis [4]. However, there are areas of medicine for which high-quality evidence generation can be difficult. This is due to disease characteristics such as rare occurrence and clinical heterogeneity among patients with the same condition, which can mean either that trials are difficult to interpret or that they may only be directly applicable to a subset of patients [5, 6]. A lack of resources and/or infrastructure can also be limiting [6, 7]. Moreover, even when evidence does exist, in medical situations with multiple considerations or confounding factors, there is the need to prioritise the use of available evidence to optimise outcomes [8].

Therefore, when no robust evidence is available, when divergent guidance exists, or when there is a need for collective judgement to increase reliability and validity, guidelines for clinical decision making or methodological or reporting approaches may be formulated based on expert consensus only [9-11]. Consensus methods provide opportunities to harness the knowledge of experts to support clinical decision making in areas of uncertainty [12]. As with all studies, appropriate methods and transparent reporting are key; however, the method used to reach consensus is not always clearly reported [11, 13].

For peer review only - http://bmjopen.bmj.com/site/about/guidelines.xhtml

Multiple methods are used to develop consensus-based publications. These range in methodological rigour from informal "expert consensus meetings" to structured or systematic approaches such as the Delphi method and the Nominal Group Technique (NGT). Both Delphi and NGT are used for generating ideas or determining priorities, aiming to achieve general convergence, usually through voting on a series of multiple-choice questions [14-17]. In Delphi, and more recently eDelphi, individuals vote anonymously, while NGT is usually face-to-face [8, 18, 19]. The techniques and methodological steps used to reach consensus can vary (**Table 1**).

In group decisions, a wider range of knowledge may be drawn upon, the interaction between group members can stimulate and challenge received ideas, and idiosyncrasies may be filtered out through the group prioritisation process [19-22]. The use of structured, systematic approaches to reach consensus is supported by the observation that, in an unstructured group meeting, there is the risk of a single individual dominating the discussion and decisions may be portrayed as unanimous when, in reality, there is dissent within the group [20]. Even within structured consensus meetings, depending on their roles, a few panel members can dominate the discussion [23]. Furthermore, individuals may be unwilling to retract long-held views in open discussion. For these reasons, structured approaches including a step where responses are anonymised are generally held to be superior to unstructured methods to achieve consensus [24, 25].

Developing consensus-based publications using robust methods is vital, but poor execution or reporting can render the techniques used for gathering opinion susceptible to criticism [26-29]. To take one of the most widely-used and most rigorous consensus methodologies, the Delphi method has been used extensively in a wide range of sectors including military, education, social science and healthcare since its conception in the 1950s at the RAND Corporation [30]. This is because it has the potential to mitigate many of the aforementioned pitfalls in group decisions, such as the

For peer review only - http://bmjopen.bmj.com/site/about/guidelines.xhtml

risk of peer pressure in techniques such as the NGT [27, 31]. Due to its versatility, the Delphi method can be modified to meet individual study needs. However, the reporting of such "modified Delphi" methods may lack clarity on the details of the process involved or the rationale for the modification [27, 31].

Definitions of the thresholds for consensus (i.e., approval rates), for example, can vary or be poorly described in studies using consensus [32]. Other reporting or methodological problems identified are that analytical methods may not be predefined [26, 32], the recruitment process used to identify the experts may not be explicit [33], or the funding source not clearly disclosed [34]. In fact, critics suggest the term "Delphi research" be phased out in academic publications to force authors to more precisely describe the methodology used [35].

The lack of appropriate and transparent description in publications of the consensus methods used suggests that a reporting guideline is needed. A reporting guideline comprises "a checklist, flow diagram, or explicit text to guide authors in reporting a specific type of research, developed using explicit methodology" [11]. Consensus methods themselves play an important role in the development of reporting guidelines in various fields of health. As part of an ongoing audit of the EQUATOR database [36], it has been observed that, of the 226 reporting guidelines added between database inception and October 2018, only one third (77/226) explicitly mentioned the use of Delphi methodology (**Figure 1**), while in another third (75), the information was not reported. A systematic review of the EQUATOR database indicated a similar result and added that among the reporting guidelines that mentioned the Delphi method, the description of details of the participants, number of rounds, criteria for dropping items or stopping the rounds was not always reproducible [37].

For peer review only

For peer review only - http://bmjopen.bmj.com/site/about/guidelines.xhtml

A range of methods can be used to reach consensus for clinical guidance, nomenclature, and other approaches in healthcare and public health [38]. However, to the best of our knowledge, the only reporting guidance in healthcare using consensus research is the CREDES (guidance on Conducting and REporting DElphi Studies) Statement, which provides valuable recommendations for the reporting of Delphi consensus in palliative care [27]. Nevertheless, CREDES is specific to palliative care and is limited to the Delphi method [27], which leaves a gap for a reporting guideline that can be applied to other biomedical areas and consensus processes involving non-Delphi based methods or "modified Delphi" — an issue that CREDES acknowledges. Moreover, CREDES does not provide a detailed checklist to guide the incorporation of essential steps to be reported.

Detail-oriented reporting can help readers of publications to understand the key elements of the process – the methodology used, the participants involved, and how the study was conducted including the criteria for statement approval. Our objective is therefore to systematically develop a reporting guideline to help the biomedical research and clinical practice community describe the methods used to reach consensus in a complete, transparent, and consistent manner. Our aim is that the reporting guideline is appropriate to describe all types of consensus methodology. The reporting guideline for consensus-based biomedical publications will include a general statement with a checklist and an explanation and elaboration (E&E) document, including examples of good reporting. It will be identified under the acronym ACCORD (ACcurate COnsensus Reporting Document).

For peer review only - http://bmjopen.bmj.com/site/about/guidelines.xhtml

METHODS/DESIGN

We have adopted the general method proposed by the EQUATOR Network for developing reporting guidelines [11]. The process for ACCORD development is outlined in **Figure 2**.

Stage 1: Establishment of a Steering Committee

With the endorsement of the International Society of Medical Publication Professionals (ISMPP), we assembled a Steering Committee to develop a reporting guideline for research using consensus. The Steering Committee (the authors, AH, AP, CW, DT, EH, EvZ, KG, NH, PL, RM, and WG) will lead and co-ordinate the guideline development process. Specifically, the Steering Committee will be responsible for: establishing the goals and timelines for the work, including registering and publishing the protocol; generating the initial list of checklist items from the literature review; conducting a consensus process to enrich and refine the initial list of minimum items that should be reported; implementing each stage of the process including developing questionnaires and analysing voting outcomes and other data; reporting the findings of the process in a statement document with the main checklist and guidance; developing an E&E document where all the items are individually explained and examples of approach and reporting are given; disseminating the reporting guidelines via publication, presentation at congresses and other events, and online presence including a website linked to social media channels.

For peer review only

The Steering Committee is a multidisciplinary group (11 people) that includes clinician practitioners, methodologists, publication professionals, patients, journal editors and publishers and the pharmaceutical industry. Prior to initiating Stage 2, we listed the project in the EQUATOR Network registry for reporting guidelines under development [39] and registered the protocol with the Open Science Framework [40].

For peer review only - http://bmjopen.bmj.com/site/about/guidelines.xhtml

Stage 2: Literature review and generation of draft checklist items

The aim of this step is to seek evidence on the quality of reporting of the process undertaken in health studies using consensus methodology. This research will provide insight into possible checklist items for evaluation by the Delphi Panel (further information on the Delphi Panel is provided in 'Stage 3' below). The CREDES guidelines, specific to palliative care, will also be reviewed for elements that can be generalised to other biomedical fields [27].

Search strategy

The process for conducting the systematic review will be informed by and reported according to the Preferred Reporting Items for Systematic reviews and Meta-Analyses (PRISMA) 2020 and PRISMA-Search extension guidelines [11, 41]. Eligible studies will include studies, reviews and published guidance addressing the quality of reporting of consensus methodology that aim to improve health outcomes in biomedicine or clinical practice. Reports of studies using consensus methods but not commenting their reporting quality will be excluded, for example, studies to reach clinical recommendations of core outcome sets or reporting guidelines using consensus methods. Ineligible publications include editorials, letters about individual publications, and comments on methodology of consensus outside the scope of biomedical research.

Searches of EMBASE (OVID), MEDLINE (OVID), Web of Science - Core Collection, MEDLINE (Web of Science), PubMed, Cochrane Library, and Emcare (OVID), Academic Search Premier and PsycINFO databases will be run with no limits by year or language of publication at the search stage. Four initial search strategies were developed and sequentially piloted by members of the Steering Committee (WG, EvZ and PL) with the assistance of an information (JS) and

For peer review only - http://bmjopen.bmj.com/site/about/guidelines.xhtml

systematic review specialist (ZF). The piloting allowed the adjustment of the initial search strategy by the information specialist to provide results that better aligned with the inclusion criteria and objective of this study. The refined, broad search strategy (Supplementary File) will be used to identify and generate the final list of studies focusing on the quality and accuracy of reporting of Delphi and other consensus processes, methods, techniques or recommendations. The search may also be augmented with relevant articles highlighted by the Steering Committee as appropriate based on the individuals' prior work and expertise in the area (via a manual search).

Data extraction

EvZ, PL, WG, and ZF will independently screen the titles and abstracts retrieved from the search for potential inclusion using the Rayyan tool in blind mode [42]. Any discrepancies will be resolved by discussion. Full-text articles will then be retrieved and assessed independently for eligibility, with reconciliation of any differences through discussion. Data will be extracted using a draft extraction form which will be piloted on three studies before use. Based on the information gathered on the literature review, a list of preliminary items for the checklist will be generated to be refined in a Delphi exercise in Stage 3.

Stage 3: Reaching consensus on checklist items

We will use Delphi methodology, as described below, to reach a consensus regarding the checklist items to include in the reporting guideline. This will take place in two steps, with the first involving the Steering Committee and the second involving a full Delphi Panel (the ACCORD Delphi Panel;

For peer review only - http://bmjopen.bmj.com/site/about/guidelines.xhtml

Figure 3). We plan to report the consensus methodology in accordance with our own guidelines under development.

First step: Steering Committee Survey

The Steering Committee will review the data extracted from literature search. This initial list is likely to contain duplicated items or items that require rewording. The aim is to eliminate repetitions and inadequately or ambiguously written items to reach a list of unique items. Using a survey, the Steering Committee members involved in the literature review will independently suggest items for the initial checklist; NH and WG will consolidate the initial checklist items.

There will then be anonymous voting to confirm the initial checklist that will be put to the full ACCORD consensus panel. Steering Committee members (excluding NH and WG) will vote (anonymised and blinded) on whether they 'Strongly Agree', 'Agree', 'Disagree', 'Strongly Disagree', or feel 'Abstained/Unable to answer' for all proposed items. There will also be the opportunity to provide comments. Any items that do not receive support will be discussed by the Steering Committee, and either included as 'possible additional items' or discarded completely. The eliminated items and the reasons for their elimination will be reported. The candidate items will be presented in sequence as a draft checklist, and in the same order to all people voting, so that the overall checklist structure, considering the manuscript sections (like Introduction, Methods, Results, Discussion) can be evaluated. Within each section, there will be 'proposed items' and 'possible additional items.'

Second step: ACCORD Delphi Panel

The preliminary list of checklist items agreed on by the Steering Committee will subsequently be put to the ACCORD Delphi Panel for validation using a blinded electronic voting platform (e-

For peer review only - http://bmjopen.bmj.com/site/about/guidelines.xhtml

survey). In addition, the ACCORD Delphi Panel will be provided with the list of items excluded by the Steering Committee for information, as a confirmatory step.

The order of the candidate items within each manuscript section will be randomised so that it is different for each person voting and all items are evaluated fully independently from each other. Five voting options will be offered: ‘Strongly Agree’, ‘Agree’, ‘Disagree’, ‘Strongly Disagree’, and ‘Abstained/Unable to answer’. Votes of ‘Abstained/Unable to answer’ will be included in the denominator. Panellists will be able to provide free text comments and will have the opportunity to propose additional items. There will be three rounds of voting; with feedback and descriptive statistics incorporated for the next round by NH and WG. The approval rate and the reasons for elimination of items will be reported.

The consensus threshold is defined in this step as at least 20 respondents (approximately 50% of the target panel size), and at least 80% of responding ACCORD Delphi panellists who are able to answer voting ‘Agree’ or ‘Strongly Agree’, with two rounds of statement revision and re-voting. The steering committee will review items that do not achieve consensus in rounds 1 or 2 and these will be revised or eliminated taking into account any free-text comments. If consensus is not achieved by the ACCORD Delphi Panel, or there are insufficient respondents, the Steering Committee may decide that the item will be included as an optional item or a discussion point on the E&E document or checklist, alongside core items on which consensus was achieved. Simple descriptive statistics (response rates, level of agreement for each statement, median levels of agreement and interquartile ranges) will be used to describe approval rates between rounds. The same measures will be used to evaluate consensus stability across rounds [43].

There are no generally agreed standards for the panel size for Delphi studies, and a wide range of panel sizes has been reported; panels of 20–30 participants are common [44, 45]. However, it is

For peer review only - http://bmjopen.bmj.com/site/about/guidelines.xhtml

recognised that the size and diversity of a Delphi panel can impact the quality of the final recommendations [46]. The ACCORD Delphi Panel will comprise approximately 40 members, so that it allows for representation from clinicians, methodologists, patient advocates, lay public representatives, health technologists, journal editors and publishers, regulatory specialists, and publications professionals, and to ensure an acceptable number of responses (20, or at least 50% of the group) in the event of drop-outs or partial completion of review. The ACCORD project will be advertised to potential Delphi Panellists via relevant societies, organisations, and networks; in addition, authors of recently published consensus studies in high-profile journals will be invited directly.

When registering, panellists will be asked to complete a preliminary survey to capture basic information on experience, geographical, and demographic representation. Although no formal targets will be established, the Steering Committee will endeavour to ensure a broad spread of representation across these categories. Members of the Delphi Panel will be recognised as contributors in the acknowledgements section of the guideline (with their permission) but participation in ACCORD Delphi panel will not qualify a panellist for authorship.

Software or a voting platform that is appropriate for Delphi exercises will be used to implement the voting process, administered by NH and WG. Alternatives available on the market are being evaluated and tested at the time of this protocol publication, and the platform and version used will be reported. Initial requirements are that the software used follows security regulations, ethical standards and allows, besides voting, the inclusion of free text responses in the e-surveys to supplement discussion in the E&E document.

For peer review only - http://bmjopen.bmj.com/site/about/guidelines.xhtml

Stage 4: Creation of the reporting guideline and E&E document

On completion of the Delphi consensus process, the checklist will be finalised by WG and NH for approval by the Steering Committee, and the reporting guideline will be developed. A separate E&E document will be created to provide a detailed rationale for the items included in the checklist. In each case, an example will be included of good reporting from a published paper. The E&E document can also be informed by perspectives collected from researchers involved in consensus-based studies outside the biomedical field.

Stage 5: Dissemination

We intend to publish the reporting guideline and E&E document in open access format via a CC-BY licence. Future publications from the ACCORD project will be reported according to the best available reporting guidelines for each type of manuscript. To aid dissemination, we plan to present the findings at congresses including ISMPP European and Annual Meetings, the World Conference on Research Integrity and Peer Review, and the UK Research Integrity Office Annual Conference. Progress will be updated on a dedicated website for the ACCORD project, the EQUATOR website and newsletter, and social media channels, and communicated in appropriate professional forums and events. This dissemination of the reporting guideline is crucial for the document to be implemented in practice.

For peer review only - http://bmjopen.bmj.com/site/about/guidelines.xhtml

DISCUSSION

The ACCORD reporting guideline will provide a set of minimum items that should be reported about methods used to achieve consensus in biomedical research and guidance, including processes ranging from simple unstructured opinion gatherings to highly structured processes. The objective is to systematically develop a reporting guideline to help the biomedical research and clinical practice community describe the methods or techniques used to reach consensus in a complete, transparent, and consistent manner.

Extensions of the ACCORD reporting guideline and checklist could potentially be developed in the future to cover consensus studies in the non-biomedical sectors, with appropriate input from experts in those sectors to account for characteristics specific to each field. Our objective is to increase the completeness, transparency and consistency of the reporting of consensus methodology and, as a result, to improve the trustworthiness of recommendations developed using consensus methods. The Steering Committee welcomes enquiries from individuals interested in participating in the ACCORD Delphi Panel.

For peer review only - http://bmjopen.bmj.com/site/about/guidelines.xhtml

DECLARATIONS

Ethics approval and consent to participate

Not applicable

Consent for publication

Not applicable

Availability of data and materials

Anonymised aggregated data will be deposited in the Open Science Framework, where the study protocol has already been registered (https://osf.io/2rzm9). Individual responses to Delphi rounds will be deidentified at the source level by the platform used. These individual responses and approval rates can be requested to the corresponding author. The ACCORD protocol has been listed on the EQUATOR website (link) and pre-registered with the Open Science Framework (link).

Competing interests

PL is a member of the UK EQUATOR Centre, an organisation that promotes the use of reporting guidelines, many of which are developed using consensus methods, and she is personally involved in the development of other reporting guidelines. WG is a former employee of Ipsen and is now employed by Bristol Myers Squib. KG is an employee of AbbVie. APH, in the last five years, worked with Reckitt Benckiser for the development of the definitions and management of gastro-oesophageal reflux disease. CCW is an employee, Director, and shareholder of Oxford PharmaGenesis Ltd, a Director of Oxford Health Policy Forum CIC, a Trustee of the Friends of the National Library of Medicine, and an Associate Fellow of Green Templeton College. NH is an

For peer review only - http://bmjopen.bmj.com/site/about/guidelines.xhtml

employee of Ogilvy Health UK. EH has worked with Ogilvy Health UK on consensus projects. AP, DT, RM and EJvZ have no conflict of interest.

Funding

Ipsen provided access to full-text articles that were not otherwise freely available to support the development of this protocol and provided the open access article processing charge. Ogilvy Health provided medical writing support.

Authors' contributions

NH and WG recruited the ACCORD Steering Committee and coordinated the development, drafting, and review of this protocol. All authors contributed to the development of the protocol, and reviewed and commented, and approved the draft manuscript. The authors, except for the NH and WG, are listed in alphabetical order.

Acknowledgements

We thank Bernd Arents, who joined the Steering Committee and provided valuable input into the project but then had to step down because of other commitments. Medical writing support was provided by Sreerekha Pillai and Luke Worley of Ogilvy Health. Wendy Hazard of ISMPP provided administrative support. Jan Schoones (Leiden University Medical Center) assisted in development of the search strategy and Zbys Fedorowicz provided support for screening of hits from the systematic searches.

For peer review only

References

1. Szajewska H. Evidence-Based Medicine and Clinical Research: Both Are Needed, Neither Is Perfect. Ann Nutr Metab. 2018;72(Suppl 3):13-23. https://doi.org/10.1159/000487375.

2. Barton S. Which clinical studies provide the best evidence? The best RCT still trumps the best observational study. BMJ. 2000;321(7256):255-256. https://doi.org/10.1136/bmj.321.7256.255.

3. Gopalakrishnan S, Ganeshkumar P. Systematic Reviews and Meta-analysis: Understanding the Best Evidence in Primary Healthcare. J Family Med Prim Care. 2013;2(1):9-14. https://doi.org/10.4103/2249-4863.109934.

4. Greenhalgh T. Will COVID-19 be evidence-based medicine's nemesis? PLoS Med. 2020;17(6):e1003266. https://doi.org/10.1371/journal.pmed.1003266.

5. Kempf L, Goldsmith JC, Temple R. Challenges of developing and conducting clinical trials in rare disorders. Am J Med Genet A. 2018;176(4):773-783. https://doi.org/10.1002/ajmg.a.38413.

6. Frieden TR. Evidence for Health Decision Making - Beyond Randomized, Controlled Trials. N Engl J Med. 2017;377(5):465-475. https://doi.org/10.1056/NEJMra1614394.

7. Haw WY, Al-Janabi A, Arents BWM, Asfour L, Exton LS, Grindlay D, et al. Global Guidelines in Dermatology Mapping Project (GUIDEMAP): a scoping review of dermatology clinical practice guidelines. Br J Dermatol. 2021;185(4):467-475. https://doi.org/10.1111/bjd.20428.

8. McMillan SS, King M, Tully MP. How to use the nominal group and Delphi techniques. Int J Clin Pharm. 2016;38(3):655-662. https://doi.org/10.1007/s11096-016-0257-x.

9. Raine R, Sanderson C, Black N. Developing clinical guidelines: a challenge to current methods. BMJ. 2005;331(7517):631-633. https://doi.org/10.1136/bmj.331.7517.631.

10. Djulbegovic B, Guyatt G. Evidence vs Consensus in Clinical Practice Guidelines. JAMA. 2019;322(8):725-726. https://doi.org/10.1001/jama.2019.9751.

11. Moher D, Schulz KF, Simera I, Altman DG. Guidance for developers of health research reporting guidelines. PLoS Med. 2010;7(2):e1000217. https://doi.org/10.1371/journal.pmed.1000217.

For peer review only - http://bmjopen.bmj.com/site/about/guidelines.xhtml

12. Fink-Hafner D, Dagen T, Dousak M, Novak M, Hafner-Fink M. Delphi Method: Strengths and Weaknesses. Metodoloski zvezki. 2019;16(2):1-19. http://www.dlib.si/?URN=URN:NBN:SI:DOC-4UR1RO95.

13. Arakawa N, Bader LR. Consensus development methods: Considerations for national and global frameworks and policy development. Res Social Adm Pharm. 2021;18(1):2222-2229. https://doi.org/10.1016/j.sapharm.2021.06.024.

14. Dalkey N, Helmer O. An Experimental Application of the DELPHI Method to the Use of Experts. Management Science. 1963;9(3):458-467. https://doi.org/10.1287/mnsc.9.3.458.

15. Murphy MK, Black NA, Lamping DL, McKee CM, Sanderson CFB, Askham J, et al. Consensus development methods, and their use in clinical guideline development. Health Technol Assess. 1998;2(3):1-88. https://doi.org/10.3310/hta2030.

16. Foth T, Efstathiou N, Vanderspank-Wright B, Ufholz LA, Dutthorn N, Zimansky M, et al. The use of Delphi and Nominal Group Technique in nursing education: A review. Int J Nurs Stud. 2016;60:112-120. https://doi.org/10.1016/j.ijnurstu.2016.04.015.

17. Nair R, Aggarwal R, Khanna D. Methods of formal consensus in classification/diagnostic criteria and guideline development. Semin Arthritis Rheum. 2011;41(2):95-105. https://doi.org/10.1016/j.semarthrit.2010.12.001.

18. Grant S, Armstrong C, Khodyakov D. Online Modified-Delphi: a Potential Method for Continuous Patient Engagement Across Stages of Clinical Practice Guideline Development. J Gen Intern Med. 2021;36(6):1746-1750. https://doi.org/10.1007/s11606-020-06514-6.

19. Helmer-Hirshberg O. Analysis of the future: The Delphi Method. The RAND Corporation, Santa Monica, CA 1967. https://www.rand.org/pubs/papers/P3558.html.

20. Hsu CC, Sandford BA. The Delphi Technique: Making Sense of Consensus. Practical Assessment, Research, and Evaluation. 2007;12(10):1-8. https://doi.org/10.7275/pdz9-th90.

21. Hsieh CJ, Fific M, Yang CT. A new measure of group decision-making efficiency. Cogn Res Princ Implic. 2020;5(1):45. https://doi.org/10.1186/s41235-020-00244-3.

22. Beiderbeck D, Frevel N, von der Gracht HA, Schmidt SL, Schweitzer VM. Preparing, conducting, and analyzing Delphi surveys: Cross-disciplinary practices, new directions, and advancements. MethodsX. 2021;8:101401. https://doi.org/10.1016/j.mex.2021.101401.

23. Li S-A, Yousefi-Nooraie R, Guyatt G, Talwar G, Wang Q, Zhu Y, et al. A few panel members dominated guideline development meeting discussions: A social network

For peer review only

For peer review only - http://bmjopen.bmj.com/site/about/guidelines.xhtml

analysis. J Clin Epidemiol. 2022;141(1-10. https://doi.org/https://doi.org/10.1016/j.jclinepi.2021.09.023.

24. Hohmann E, Brand JC, Rossi MJ, Lubowitz JH. Expert Opinion Is Necessary: Delphi Panel Methodology Facilitates a Scientific Approach to Consensus. Arthroscopy. 2018;34(2):349-351. https://doi.org/10.1016/j.arthro.2017.11.022.

25. Humphrey-Murto S, Varpio L, Gonsalves C, Wood TJ. Using consensus group methods such as Delphi and Nominal Group in medical education research. Med Teach. 2017;39(1):14-19. https://doi.org/10.1080/0142159X.2017.1245856.

26. Grant S, Booth M, Khodyakov D. Lack of preregistered analysis plans allows unacceptable data mining for and selective reporting of consensus in Delphi studies. J Clin Epidemiol. 2018;99:96-105. https://doi.org/10.1016/j.jclinepi.2018.03.007.

27. Junger S, Payne SA, Brine J, Radbruch L, Brearley SG. Guidance on Conducting and REporting DElphi Studies (CREDES) in palliative care: Recommendations based on a methodological systematic review. Palliat Med. 2017;31(8):684-706. https://doi.org/10.1177/0269216317690685.

28. Hohmann E, Cote MP, Brand JC. Research Pearls: Expert Consensus Based Evidence Using the Delphi Method. Arthroscopy. 2018;34(12):3278-3282. https://doi.org/10.1016/j.arthro.2018.10.004.

29. Humphrey-Murto S, Varpio L, Wood TJ, Gonsalves C, Ufholz LA, Mascioli K, et al. The Use of the Delphi and Other Consensus Group Methods in Medical Education Research: A Review. Acad Med. 2017;92(10):1491-1498. https://doi.org/10.1097/ACM.0000000000001812.

30. Gupta UG, Clarke RE. Theory and applications of the Delphi technique: A bibliography (1975–1994). Technological Forecasting and Social Change. 1996;53(2):185-211. https://doi.org/https://doi.org/10.1016/S0040-1625(96)00094-7.

31. Veugelers R, Gaakeer MI, Patka P, Huijsman R. Improving design choices in Delphi studies in medicine: the case of an exemplary physician multi-round panel study with 100% response. BMC Med Res Methodol. 2020;20(1):156. https://doi.org/10.1186/s12874-020-01029-4.

32. Diamond IR, Grant RC, Feldman BM, Pencharz PB, Ling SC, Moore AM, et al. Defining consensus: a systematic review recommends methodologic criteria for reporting of Delphi studies. J Clin Epidemiol. 2014;67(4):401-409. https://doi.org/10.1016/j.jclinepi.2013.12.002.

33. Paré G, Cameron A-F, Poba-Nzaou P, Templier M. A systematic assessment of rigor in information systems ranking-type Delphi studies. Information & Management. 2013;50(5):207-217. https://doi.org/10.1016/j.im.2013.03.003.

For peer review only - http://bmjopen.bmj.com/site/about/guidelines.xhtml

34. Gattrell WT, Clements SJ, Sheard D. Quality assessment of guidelines/recommendations developed using Delphi methodology. Curr Med Res Opin. 2019;35(Suppl 2):40. https://doi.org/10.1080/03007995.2019.1583496.

35. Thompson M. Considering the implication of variations within Delphi research. Fam Pract. 2009;26(5):420-424. https://doi.org/10.1093/fampra/cmp051.

36. Schlussel M, Collins G, de Beyer JA, Kirtley S. EQUATOR Library Audit. [Available from:https://osf.io/fuv3c] Last accessed 21 January 2022.

37. Banno M, Tsujimoto Y, Kataoka Y. The majority of reporting guidelines are not developed with the Delphi method: a systematic review of reporting guidelines. J Clin Epidemiol. 2020;124:50-57. https://doi.org/10.1016/j.jclinepi.2020.04.010.

38. Brouwers MC, Kho ME, Browman GP, Burgers JS, Cluzeau F, Feder G, et al. AGREE II: advancing guideline development, reporting and evaluation in health care. CMAJ. 2010;182(18):E839-842. https://doi.org/10.1503/cmaj.090449.

39. EQUATOR Network. [Available from:equator-network.org] Last accessed 17 September 2021.

40. Open Science Framework Registries. [Available from:https://osf.io/2rzm9] Last accessed 07 March 2022.

41. Rethlefsen ML, Kirtley S, Waffenschmidt S, Ayala AP, Moher D, Page MJ, et al. PRISMA-S: an extension to the PRISMA Statement for Reporting Literature Searches in Systematic Reviews. Syst Rev. 2021;10(1):39. https://doi.org/10.1186/s13643-020-01542-z.

42. Ouzzani M, Hammady H, Fedorowicz Z, Elmagarmid A. Rayyan—a web and mobile app for systematic reviews. Systematic Reviews. 2016;5(1):210. https://doi.org/10.1186/s13643-016-0384-4.

43. Holey EA, Feeley JL, Dixon J, Whittaker VJ. An exploration of the use of simple statistics to measure consensus and stability in Delphi studies. BMC Medical Research Methodology. 2007;7(1):52. https://doi.org/10.1186/1471-2288-7-52.

44. Akins RB, Tolson H, Cole BR. Stability of response characteristics of a Delphi panel: application of bootstrap data expansion. BMC Med Res Methodol. 2005;5(37. https://doi.org/10.1186/1471-2288-5-37.

45. Chalmers J, Armour M. The Delphi Technique. In Handbook of Research Methods in Health Social Sciences. Singapore:Springer;2019. p. 715-735. https://doi.org/10.1007/978-981-10-5251-4_99

46. Birko S, Dove ES, Ozdemir V. Evaluation of Nine Consensus Indices in Delphi Foresight Research and Their Dependency on Delphi Survey Characteristics: A

For peer review only - http://bmjopen.bmj.com/site/about/guidelines.xhtml

Simulation Study and Debate on Delphi Design and Interpretation. PLoS One. 2015;10(8):e0135162. https://doi.org/10.1371/journal.pone.0135162.

47. Fink A, Kosecoff J, Chassin M, Brook RH. Consensus methods: characteristics and guidelines for use. Am J Public Health. 1984;74(9):979-983. https://doi.org/10.2105/ajph.74.9.979.

48. Halcomb E, Davidson P, Hardaker L. Using the consensus development conference method in healthcare research. Nurse Res. 2008;16(1):56-71. https://doi.org/10.7748/nr2008.10.16.1.56.c6753.

49. James D, Warren-Forward H. Research methods for formal consensus development. Nurse Res. 2015;22(3):35-40. https://doi.org/10.7748/nr.22.3.35.e1297.

50. Campbell SM, Cantrill JA. Consensus methods in prescribing research. J Clin Pharm Ther. 2001;26(1):5-14. https://doi.org/10.1046/j.1365-2710.2001.00331.x.

51. Drumm S, Bradley C, Moriarty F. 'More of an art than a science'? The development, design and mechanics of the Delphi Technique. Res Social Adm Pharm. 2022;18(1):2230-2236. https://doi.org/10.1016/j.sapharm.2021.06.027.

52. Durkin J, Usher K, Jackson D. Using consensus from experts to inform a shared understanding of subjective terms. Nurse Res. 2019;27(2):46-49. https://doi.org/10.7748/nr.2019.e1622.

53. Franklin KK. Idea Generation and Exploration: Benefits and Limitations of the Policy Delphi Research Method. Innov High Educ. 2006;31(4):237-246.

54. Guzys D, Dickson-Swift V, Kenny A, Threlkeld G. Gadamerian philosophical hermeneutics as a useful methodological framework for the Delphi technique. Int J Qual Stud Health Well-being. 2015;10:26291. https://doi.org/10.3402/qhw.v10.26291.

55. Keeney S, Hasson F, McKenna H. Consulting the oracle: ten lessons from using the Delphi technique in nursing research. J Adv Nurs. 2006;53(2):205-212. https://doi.org/10.1111/j.1365-2648.2006.03716.x.

56. Khodyakov D, Grant S, Denger B, Kinnett K, Martin A, Peay H, et al. Practical Considerations in Using Online Modified-Delphi Approaches to Engage Patients and Other Stakeholders in Clinical Practice Guideline Development. Patient. 2020;13(1):11-21. https://doi.org/10.1007/s40271-019-00389-4.

57. Hallowell MR. Qualitative Research: Application of the Delphi Method to CEM Research. J Constr Eng Manag. 2010;136(1):99-107. https://doi.org/10.1061/(ASCE)CO.1943-7862.0000137.

For peer review only

For peer review only - http://bmjopen.bmj.com/site/about/guidelines.xhtml

Figures and Tables

Figure 1. Methodology declared by authors in developing a reporting guideline added to the EQUATOR database from inception to October 2018 (n = 226).

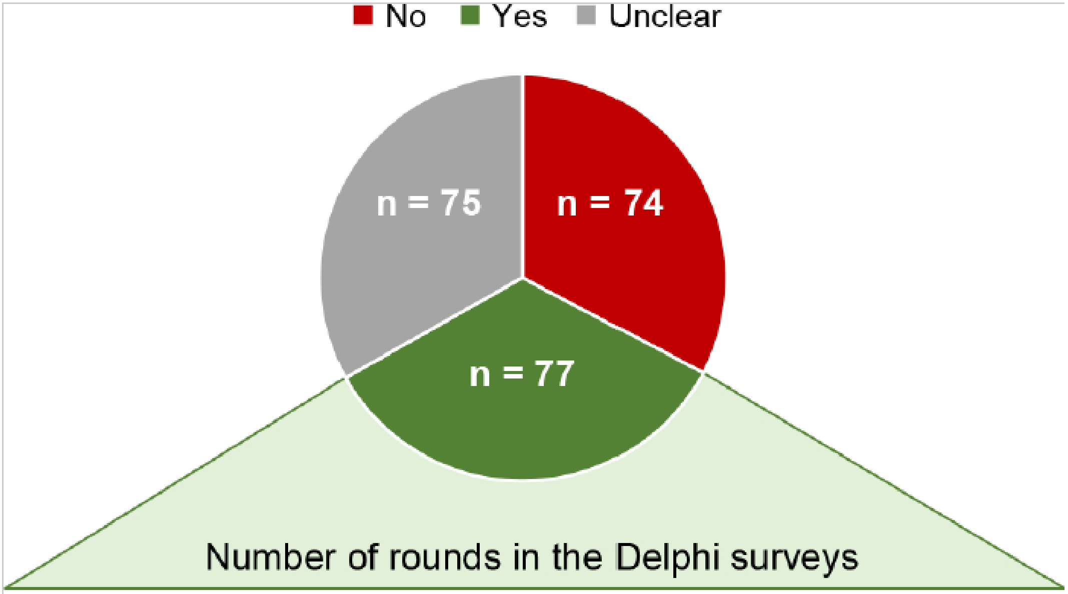

Rounds	n	%
1	8	3.54
2	32	14.16
3	24	10.62
4	6	2.65
5	1	0.44
Unclear	6	2.65
N/A	149	65.94

For peer review only - http://bmjopen.bmj.com/site/about/guidelines.xhtml

537 Figure 2. Project overview for creating ACCORD, a reporting guideline for studies developed
538 using consensus methods.

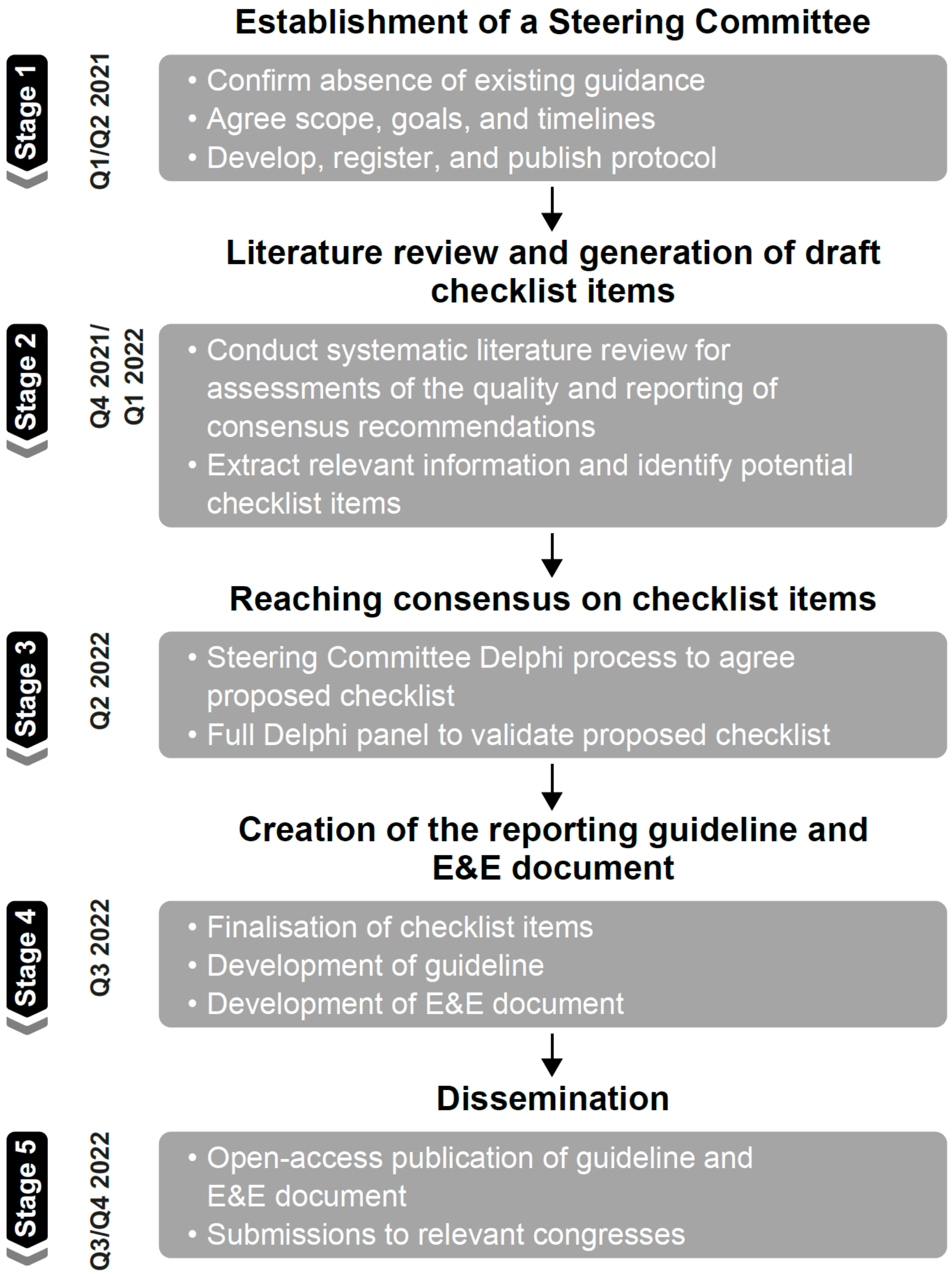

539

540

For peer review only - http://bmjopen.bmj.com/site/about/guidelines.xhtml

Figure 3. Methodology used by the ACCORD Steering Committee and ACCORD Delphi Panel to achieve consensus on core checklist items for a consensus reporting guideline.

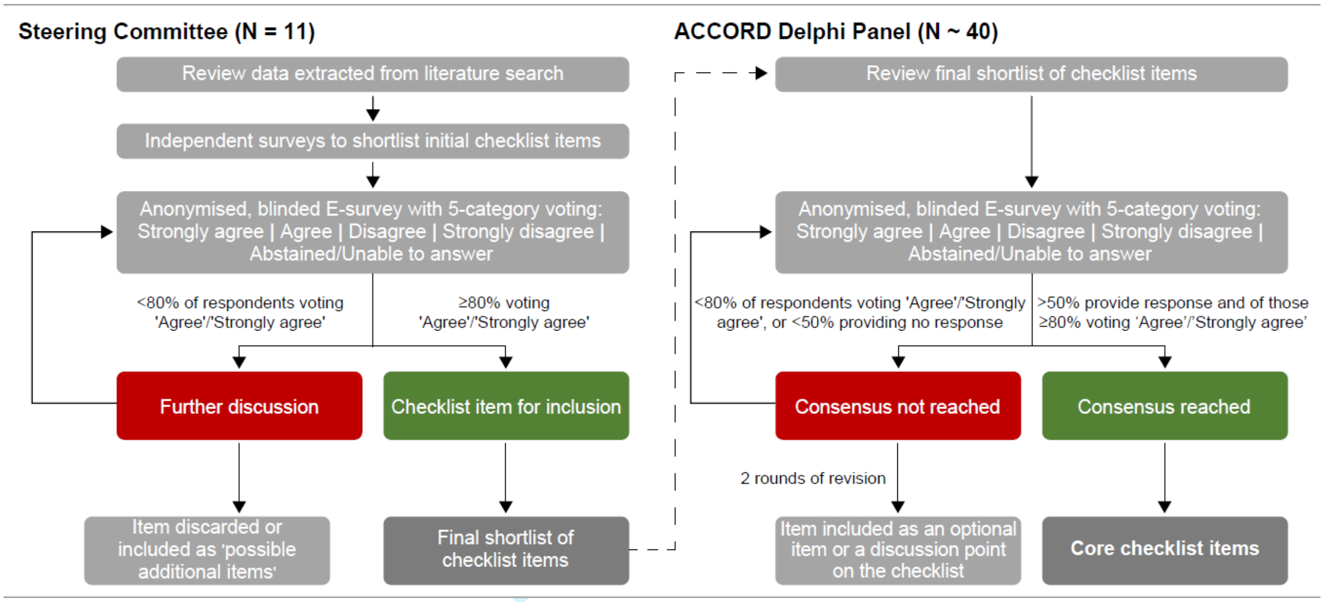

review only

For peer review only - http://bmjopen.bmj.com/site/about/guidelines.xhtml

Table 1. Possible types of consensus methods and characteristics that can be mixed or used separately in different stages of studies to reach consensus

Method	Characteristics	Data analysis
Consensus conference or meeting [47-49]	Face-to-face meetings where a group of participants, usually experts in one field of knowledge, discuss one or more topics, prompted by facilitators, and have to either create ideas/statements or decide/vote on pre-set topics/statements. The discussion is frequently prompted by evidence from the literature — or the lack of it.	Qualitative or quantitative, or mixed
Nominal group technique (NGT) [47, 49, 50]	As in conference meetings, in NGT, face-to-face meetings are held, but several sessions are organised with iterative stages. In the first step, suggestions are collected from the groups into questionnaires or lists of topics circulated again in the second step. In the second stage, participants need to vote or rate, usually using scales (like Likert scales). The group then discusses the aggregated summary of the voting or rating. The group is not anonymous and may include experts and non-experts. A facilitator makes sure every participant is given the opportunity to speak and vote.	Qualitative initially and then quantitative when responses are aggregated and summarised
Delphi [12, 47, 49-57]	The three principles of the Delphi technique are: 1) anonymity during voting/selecting/rating (participants do not meet); 2) multiple rounds (at least 2) and 3) feedback to participants to inform them about each last voting/rating before they start the next round. Delphi was traditionally organised by postal mail in the past, and now electronic specialised survey platforms facilitate the process.	Quantitative for voting/rating, qualitative when extra comments/suggestions are allowed
Other mixed methods [47, 49]	A consensus study can begin with simple focus groups to collect ideas, stories, experiences, and general opinions to start a more structured NGT or Delphi exercise. Frequently, two or more methods are used. For example, a Delphi activity can be used initially with the list of statements approved to be discussed in consensus conferences where final decisions are made, sometimes referred to as a "modified Delphi".	Qualitative methods are used when perceptions, stories, and experiences are collected. Several quantitative statistics can be used to summarise voting and ratings

For peer review only

For peer review only - http://bmjopen.bmj.com/site/about/guidelines.xhtml

Web of Science Core Collection and MEDLINE (Web of Science)
http://isiknowledge.com/wos

((TI=("Delphi Technique" OR "Delphi Technique" OR "Delphi techniques" OR "Delphi method" OR "Delphi methods" OR "Delphi study" OR "Delphi studies" OR "Delphi survey" OR "Delphi surveys" OR "Delphi consensus" OR "Delphi based consensus" OR "Delphi questionnaire" OR "Delphi questionnaires" OR "Delphi research" OR "Delphi review" OR "Delphi reviews" OR "Delphi process" OR "Delphi processes" OR "Delphi based" OR "Delphi procedure" OR "Delphi procedures" OR "Delphi assessment" OR "Delphi assessments" OR "Delphi approach" OR "Delphi approaches" OR "Delphi panel" OR "Delphi panels" OR "Delphi round" OR "Delphi rounds" OR "Delphi analysis" OR "Delphi expert" OR "Delphi experts" OR "Delphi consultation" OR "Delphi methodology" OR "nominal group technique" OR "nominal group techniques" OR "nominal group" OR "nominal groups" OR "nominal grouping" OR "consensus recommendation" OR "consensus recommendations" OR "consensus development" OR "consensus activity" OR "consensus activities" OR "Consensus Development Conference" OR "Consensus Development" OR "Consensus methodology" OR "consensus method*" OR "RAND" OR (("Guidelines" OR "guideline") NEAR/2 ("consensus" OR "delphi"))) OR AB=("Delphi Technique" OR "Delphi Technique" OR "Delphi techniques" OR "Delphi method" OR "Delphi methods" OR "Delphi study" OR "Delphi studies" OR "Delphi survey" OR "Delphi surveys" OR "Delphi consensus" OR "Delphi based consensus" OR "Delphi questionnaire" OR "Delphi questionnaires" OR "Delphi research" OR "Delphi review" OR "Delphi reviews" OR "Delphi process" OR "Delphi processes" OR "Delphi based" OR "Delphi procedure" OR "Delphi procedures" OR "Delphi assessment" OR "Delphi assessments" OR "Delphi approach" OR "Delphi approaches" OR "Delphi panel" OR "Delphi panels" OR "Delphi round" OR "Delphi rounds" OR "Delphi analysis" OR "Delphi expert" OR "Delphi experts" OR "Delphi consultation" OR "Delphi methodology" OR "nominal group technique" OR "nominal group techniques" OR "nominal group" OR "nominal groups" OR "nominal grouping" OR "consensus recommendation" OR "consensus recommendations" OR "consensus development" OR "consensus activity" OR "consensus activities" OR "Consensus Development Conference" OR "Consensus Development" OR "Consensus methodology" OR "consensus method*" OR "RAND") OR AK=("Delphi Technique" OR "Delphi Technique" OR "Delphi techniques" OR "Delphi method" OR "Delphi methods" OR "Delphi study" OR "Delphi studies" OR "Delphi survey" OR "Delphi surveys" OR "Delphi consensus" OR "Delphi based consensus" OR "Delphi questionnaire" OR "Delphi questionnaires" OR "Delphi research" OR "Delphi review" OR "Delphi reviews" OR "Delphi process" OR "Delphi processes" OR "Delphi based" OR "Delphi procedure" OR "Delphi procedures" OR "Delphi assessment" OR "Delphi assessments" OR "Delphi approach" OR "Delphi approaches" OR "Delphi panel" OR "Delphi panels" OR "Delphi round" OR "Delphi rounds" OR "Delphi analysis" OR "Delphi expert" OR "Delphi experts" OR "Delphi consultation" OR "Delphi methodology" OR "nominal group technique" OR "nominal group techniques" OR "nominal group" OR "nominal groups" OR "nominal grouping" OR "consensus recommendation" OR "consensus recommendations" OR "consensus development" OR "consensus activity" OR "consensus activities" OR "Consensus Development Conference" OR "Consensus

For peer review only

Development" OR "Consensus methodology" OR "consensus method*" OR "RAND" OR (("Guidelines" OR "guideline") NEAR/2 ("consensus" OR "delphi")))) AND (TI=("quality of reporting" OR "reporting quality" OR "reporting qualities" OR "selective reporting" OR "poor reporting" OR "poor reported" OR "reporting guideline" OR "reporting" OR ("reporting" AND ("quality" OR "selective" OR "poor" OR "weak" OR "manner" OR "rigor" OR "improv*")) OR "Data Accuracy" OR "quality assessment" OR "strengths" OR "strength" OR "weaknesses" OR "weakness" OR "research method" OR "research methods" OR "research method*") OR AK=("quality of reporting" OR "reporting quality" OR "reporting qualities" OR "selective reporting" OR "poor reporting" OR "poor reported" OR ("reporting" NEAR/5 ("quality" OR "selective" OR "poor" OR "weak" OR "manner" OR "rigor" OR "improv*")) OR "Data Accuracy" OR "Research Report standards" OR "quality assessment" OR "strengths" OR "strength" OR "weaknesses" OR "weakness" OR "research method" OR "research methods" OR "research method*") OR AB=("quality of reporting" OR "reporting quality" OR "reporting qualities" OR "selective reporting" OR "poor reporting" OR "poor reported" OR ("reporting" NEAR/5 ("quality" OR "selective" OR "poor" OR "weak" OR "manner" OR "rigor" OR "improv*")) OR "Data Accuracy" OR "Research Report standards" OR "quality assessment" OR "strengths" OR "strength" OR "weaknesses" OR "weakness")))

PubMed
http://www.ncbi.nlm.nih.gov/pubmed?otool=leiden

(("Delphi Technique"[majr] OR "Delphi Technique"[ti] OR "Delphi techniques"[ti] OR "Delphi method"[ti] OR "Delphi methods"[ti] OR "Delphi study"[ti] OR "Delphi studies"[ti] OR "Delphi survey"[ti] OR "Delphi surveys"[ti] OR "Delphi consensus"[ti] OR "Delphi based consensus"[ti] OR "Delphi questionnaire"[ti] OR "Delphi questionnaires"[ti] OR "Delphi research"[ti] OR "Delphi review"[ti] OR "Delphi reviews"[ti] OR "Delphi process"[ti] OR "Delphi processes"[ti] OR "Delphi based"[ti] OR "Delphi procedure"[ti] OR "Delphi procedures"[ti] OR "Delphi assessment"[ti] OR "Delphi assessments"[ti] OR "Delphi approach"[ti] OR "Delphi approaches"[ti] OR "Delphi panel"[ti] OR "Delphi panels"[ti] OR "Delphi round"[ti] OR "Delphi rounds"[ti] OR "Delphi analysis"[ti] OR "Delphi expert"[ti] OR "Delphi experts"[ti] OR "Delphi consultation"[ti] OR "Delphi methodology"[ti] OR "nominal group technique"[ti] OR "nominal group techniques"[ti] OR "nominal group"[ti] OR "nominal groups"[ti] OR "nominal grouping"[ti] OR "consensus recommendation"[ti] OR "consensus recommendations"[ti] OR "consensus development"[ti] OR "consensus activity"[ti] OR "consensus activities"[ti] OR "consensus methodology"[ti] OR "consensus method*"[ti] OR "Consensus Development Conferences as Topic"[majr] OR "RAND"[ti] OR ("Guidelines as Topic"[majr:noexp] AND ("consensus"[tw] OR "delphi"[tw]))) AND ("reporting"[ti] OR "quality of reporting"[tw] OR "reporting quality"[tw] OR "reporting qualities"[tw] OR "selective reporting"[tw] OR "poor reporting"[tw] OR "poor reported"[tw] OR "poorly reported"[tw] OR "Research Report/standards"[majr] OR "Research Design/standards"[mesh] OR "Research Design"[majr:noexp] OR "Writing/standards"[mesh] OR "Writing"[majr] OR "research method"[ti] OR "research methods"[ti] OR "research method*"[ti]))

For peer review only - http://bmjopen.bmj.com/site/about/guidelines.xhtml

MEDLINE via OVID
http://gateway.ovid.com/ovidweb.cgi?T=JS&MODE=ovid&NEWS=n&PAGE=main&D=medall

Gewijzigde veldcode

((exp *"Delphi Technique"/ OR "Delphi Technique".ti OR "Delphi techniques".ti OR "Delphi method".ti OR "Delphi methods".ti OR "Delphi study".ti OR "Delphi studies".ti OR "Delphi survey".ti OR "Delphi surveys".ti OR "Delphi consensus".ti OR "Delphi based consensus".ti OR "Delphi questionnaire".ti OR "Delphi questionnaires".ti OR "Delphi research".ti OR "Delphi review".ti OR "Delphi reviews".ti OR "Delphi process".ti OR "Delphi processes".ti OR "Delphi based".ti OR "Delphi procedure".ti OR "Delphi procedures".ti OR "Delphi assessment".ti OR "Delphi assessments".ti OR "Delphi approach".ti OR "Delphi approaches".ti OR "Delphi panel".ti OR "Delphi panels".ti OR "Delphi round".ti OR "Delphi rounds".ti OR "Delphi analysis".ti OR "Delphi expert".ti OR "Delphi experts".ti OR "Delphi consultation".ti OR "Delphi methodology".ti OR "nominal group technique".ti OR "nominal group techniques".ti OR "nominal group".ti OR "nominal groups".ti OR "nominal grouping".ti OR "consensus recommendation".ti OR "consensus recommendations".ti OR "consensus development".ti OR "consensus activity".ti OR "consensus activities".ti OR "consensus methodology".ti OR "consensus method*".ti OR exp *"Consensus Development Conferences as Topic"/ OR "RAND".ti OR ("Guidelines as Topic"/ AND ("consensus".mp OR "delphi".mp)) OR (("Guidelines".mp OR "guideline".mp) ADJ2 ("consensus".mp OR "delphi".mp))) AND ("reporting".ti OR "quality of reporting".mp OR "reporting quality".mp OR "reporting qualities".mp OR "selective reporting".mp OR "poor reporting".mp OR "poor reported".mp OR "poorly reported".mp OR "Research Report/standards"/ OR exp "Research Design"/st OR *"Research Design"/ OR exp "Writing"/st OR exp *"Writing"/ OR "research method".ti OR "research methods".ti OR "research method*".ti OR ("reporting" ADJ8 ("quality" OR "selective" OR "poor" OR "weak" OR "manner" OR "rigor" OR "improv*")).mp))

Embase
http://ovidsp.ovid.com/ovidweb.cgi?T=JS&PAGE=main&MODE=ovid&D=oemezd

Gewijzigde veldcode

((exp *"Delphi Study"/ OR "Delphi Technique".ti OR "Delphi techniques".ti OR "Delphi method".ti OR "Delphi methods".ti OR "Delphi study".ti OR "Delphi studies".ti OR "Delphi survey".ti OR "Delphi surveys".ti OR "Delphi consensus".ti OR "Delphi based consensus".ti OR "Delphi questionnaire".ti OR "Delphi questionnaires".ti OR "Delphi research".ti OR "Delphi review".ti OR "Delphi reviews".ti OR "Delphi process".ti OR "Delphi processes".ti OR "Delphi based".ti OR "Delphi procedure".ti OR "Delphi procedures".ti OR "Delphi assessment".ti OR "Delphi assessments".ti OR "Delphi approach".ti OR "Delphi approaches".ti OR "Delphi panel".ti OR "Delphi panels".ti OR "Delphi round".ti OR "Delphi rounds".ti OR "Delphi analysis".ti OR "Delphi expert".ti OR "Delphi experts".ti OR "Delphi consultation".ti OR "Delphi methodology".ti OR "nominal group technique".ti OR "nominal group techniques".ti OR "nominal group".ti OR "nominal groups".ti OR "nominal grouping".ti OR "consensus recommendation".ti OR "consensus recommendations".ti OR "consensus development".ti OR "consensus

activity".ti OR "consensus activities".ti OR "consensus methodology".ti OR "consensus method*".ti OR exp *"Consensus Development"/ OR "RAND".ti OR (("Guidelines".ti,ab OR "guideline".ti,ab) ADJ2 ("consensus".ti,ab OR "delphi".ti,ab))) AND ("reporting".ti OR "quality of reporting".ti,ab OR "reporting quality".ti,ab OR "reporting qualities".ti,ab OR "selective reporting".ti,ab OR "poor reporting".ti,ab OR "poor reported".ti,ab OR "poorly reported".ti,ab OR *"Methodology"/ OR *"Writing"/ OR "research method".ti OR "research methods".ti OR "research method*".ti OR ("reporting" ADJ8 ("quality" OR "selective" OR "poor" OR "weak" OR "manner" OR "rigor" OR "improv*")).ti,ab))

Cochrane
https://www.cochranelibrary.com/advanced-search/search-manager

(("Delphi Technique" OR "Delphi Technique" OR "Delphi techniques" OR "Delphi method" OR "Delphi methods" OR "Delphi study" OR "Delphi studies" OR "Delphi survey" OR "Delphi surveys" OR "Delphi consensus" OR "Delphi based consensus" OR "Delphi questionnaire" OR "Delphi questionnaires" OR "Delphi research" OR "Delphi review" OR "Delphi reviews" OR "Delphi process" OR "Delphi processes" OR "Delphi based" OR "Delphi procedure" OR "Delphi procedures" OR "Delphi assessment" OR "Delphi assessments" OR "Delphi approach" OR "Delphi approaches" OR "Delphi panel" OR "Delphi panels" OR "Delphi round" OR "Delphi rounds" OR "Delphi analysis" OR "Delphi expert" OR "Delphi experts" OR "Delphi consultation" OR "Delphi methodology" OR "nominal group technique" OR "nominal group techniques" OR "nominal group" OR "nominal groups" OR "nominal grouping" OR "consensus recommendation" OR "consensus recommendations" OR "consensus development" OR "consensus activity" OR "consensus activities" OR "Consensus Development Conference" OR "Consensus Development" OR "Consensus methodology" OR "consensus method*" OR "RAND" OR (("Guidelines" OR "guideline") NEAR/2 ("consensus" OR "delphi"))) AND ("quality of reporting" OR "reporting quality" OR "reporting qualities" OR "selective reporting" OR "poor reporting" OR "poor reported" OR ("reporting" NEAR/5 ("quality" OR "selective" OR "poor" OR "weak" OR "manner" OR "rigor" OR "improv*")) OR "Data Accuracy" OR "Research Report standards" OR "quality assessment" OR "strengths" OR "strength" OR "weaknesses" OR "weakness" OR "research method" OR "research methods" OR "research method*")):ti,ab,kw

Emcare
http://ovidsp.ovid.com/ovidweb.cgi?T=JS&NEWS=n&CSC=Y&PAGE=main&D=emcr

((exp *"Delphi Study"/ OR "Delphi Technique".ti OR "Delphi techniques".ti OR "Delphi method".ti OR "Delphi methods".ti OR "Delphi study".ti OR "Delphi studies".ti OR "Delphi survey".ti OR "Delphi surveys".ti OR "Delphi consensus".ti OR "Delphi based consensus".ti OR "Delphi questionnaire".ti OR "Delphi questionnaires".ti OR "Delphi research".ti OR "Delphi review".ti OR "Delphi reviews".ti OR "Delphi process".ti OR "Delphi processes".ti OR "Delphi based".ti OR "Delphi procedure".ti OR "Delphi procedures".ti OR "Delphi assessment".ti OR "Delphi assessments".ti OR "Delphi

For peer review only

approach".ti OR "Delphi approaches".ti OR "Delphi panel".ti OR "Delphi panels".ti OR "Delphi round".ti OR "Delphi rounds".ti OR "Delphi analysis".ti OR "Delphi expert".ti OR "Delphi experts".ti OR "Delphi consultation".ti OR "Delphi methodology".ti OR "nominal group technique".ti OR "nominal group techniques".ti OR "nominal group".ti OR "nominal groups".ti OR "nominal grouping".ti OR "consensus recommendation".ti OR "consensus recommendations".ti OR "consensus development".ti OR "consensus activity".ti OR "consensus activities".ti OR "consensus methodology".ti OR "consensus method*".ti OR exp *"Consensus Development"/ OR "RAND".ti OR (("Guidelines".ti,ab OR "guideline".ti,ab) ADJ2 ("consensus".ti,ab OR "delphi".ti,ab))) AND ("reporting".ti OR "quality of reporting".ti,ab OR "reporting quality".ti,ab OR "reporting qualities".ti,ab OR "selective reporting".ti,ab OR "poor reporting".ti,ab OR "poor reported".ti,ab OR "poorly reported".ti,ab OR *"Methodology"/ OR *"Writing"/ OR "research method".ti OR "research methods".ti OR "research method*".ti OR ("reporting" ADJ8 ("quality" OR "selective" OR "poor" OR "weak" OR "manner" OR "rigor" OR "improv*")).ti,ab))

Academic Search Premier
http://search.ebscohost.com/login.aspx?authtype=ip,uid&profile=lumc&defaultdb=aph

((TI("Delphi Technique" OR "Delphi Technique" OR "Delphi techniques" OR "Delphi method" OR "Delphi methods" OR "Delphi study" OR "Delphi studies" OR "Delphi survey" OR "Delphi surveys" OR "Delphi consensus" OR "Delphi based consensus" OR "Delphi questionnaire" OR "Delphi questionnaires" OR "Delphi research" OR "Delphi review" OR "Delphi reviews" OR "Delphi process" OR "Delphi processes" OR "Delphi based" OR "Delphi procedure" OR "Delphi procedures" OR "Delphi assessment" OR "Delphi assessments" OR "Delphi approach" OR "Delphi approaches" OR "Delphi panel" OR "Delphi panels" OR "Delphi round" OR "Delphi rounds" OR "Delphi analysis" OR "Delphi expert" OR "Delphi experts" OR "Delphi consultation" OR "Delphi methodology" OR "nominal group technique" OR "nominal group techniques" OR "nominal group" OR "nominal groups" OR "nominal grouping" OR "consensus recommendation" OR "consensus recommendations" OR "consensus development" OR "consensus activity" OR "consensus activities" OR "Consensus Development Conference" OR "Consensus Development" OR "Consensus methodology" OR "consensus method*" OR "RAND" OR (("Guidelines" OR "guideline") N2 ("consensus" OR "delphi"))) OR KW("Delphi Technique" OR "Delphi Technique" OR "Delphi techniques" OR "Delphi method" OR "Delphi methods" OR "Delphi study" OR "Delphi studies" OR "Delphi survey" OR "Delphi surveys" OR "Delphi consensus" OR "Delphi based consensus" OR "Delphi questionnaire" OR "Delphi questionnaires" OR "Delphi research" OR "Delphi review" OR "Delphi reviews" OR "Delphi process" OR "Delphi processes" OR "Delphi based" OR "Delphi procedure" OR "Delphi procedures" OR "Delphi assessment" OR "Delphi assessments" OR "Delphi approach" OR "Delphi approaches" OR "Delphi panel" OR "Delphi panels" OR "Delphi round" OR "Delphi rounds" OR "Delphi analysis" OR "Delphi expert" OR "Delphi experts" OR "Delphi consultation" OR "Delphi methodology" OR "nominal group technique" OR "nominal group techniques" OR "nominal group" OR "nominal groups" OR "nominal grouping" OR "consensus recommendation" OR "consensus recommendations" OR "consensus

development" OR "consensus activity" OR "consensus activities" OR "Consensus Development Conference" OR "Consensus Development" OR "Consensus methodology" OR "consensus method*" OR "RAND" OR (("Guidelines" OR "guideline") N2 ("consensus" OR "delphi")))) AND (TI("quality of reporting" OR "reporting quality" OR "reporting qualities" OR "selective reporting" OR "poor reporting" OR "poor reported" OR "reporting guideline" OR "reporting" OR ("reporting" AND ("quality" OR "selective" OR "poor" OR "weak" OR "manner" OR "rigor" OR "improv*")) OR "Data Accuracy" OR "quality assessment" OR "strengths" OR "strength" OR "weaknesses" OR "weakness" OR "research method" OR "research methods" OR "research method*") OR KW("quality of reporting" OR "reporting quality" OR "reporting qualities" OR "selective reporting" OR "poor reporting" OR "poor reported" OR ("reporting" N5 ("quality" OR "selective" OR "poor" OR "weak" OR "manner" OR "rigor" OR "improv*")) OR "Data Accuracy" OR "Research Report standards" OR "quality assessment" OR "strengths" OR "strength" OR "weaknesses" OR "weakness" OR "research method" OR "research methods" OR "research method*") OR AB("quality of reporting" OR "reporting quality" OR "reporting qualities" OR "selective reporting" OR "poor reporting" OR "poor reported" OR ("reporting" N5 ("quality" OR "selective" OR "poor" OR "weak" OR "manner" OR "rigor" OR "improv*")) OR "Data Accuracy" OR "Research Report standards" OR "quality assessment" OR "strengths" OR "strength" OR "weaknesses" OR "weakness")))

PsycINFO
http://search.ebscohost.com/login.aspx?authtype=ip,uid&profile=lumc&defaultdb=psyh

((TI("Delphi Technique" OR "Delphi Technique" OR "Delphi techniques" OR "Delphi method" OR "Delphi methods" OR "Delphi study" OR "Delphi studies" OR "Delphi survey" OR "Delphi surveys" OR "Delphi consensus" OR "Delphi based consensus" OR "Delphi questionnaire" OR "Delphi questionnaires" OR "Delphi research" OR "Delphi review" OR "Delphi reviews" OR "Delphi process" OR "Delphi processes" OR "Delphi based" OR "Delphi procedure" OR "Delphi procedures" OR "Delphi assessment" OR "Delphi assessments" OR "Delphi approach" OR "Delphi approaches" OR "Delphi panel" OR "Delphi panels" OR "Delphi round" OR "Delphi rounds" OR "Delphi analysis" OR "Delphi expert" OR "Delphi experts" OR "Delphi consultation" OR "Delphi methodology" OR "nominal group technique" OR "nominal group techniques" OR "nominal group" OR "nominal groups" OR "nominal grouping" OR "consensus recommendation" OR "consensus recommendations" OR "consensus development" OR "consensus activity" OR "consensus activities" OR "Consensus Development Conference" OR "Consensus Development" OR "Consensus methodology" OR "consensus method*" OR "RAND" OR (("Guidelines" OR "guideline") N2 ("consensus" OR "delphi"))) OR AB("Delphi Technique" OR "Delphi Technique" OR "Delphi techniques" OR "Delphi method" OR "Delphi methods" OR "Delphi study" OR "Delphi studies" OR "Delphi survey" OR "Delphi surveys" OR "Delphi consensus" OR "Delphi based consensus" OR "Delphi questionnaire" OR "Delphi questionnaires" OR "Delphi research" OR "Delphi review" OR "Delphi reviews" OR "Delphi process" OR "Delphi processes" OR "Delphi based" OR "Delphi procedure" OR "Delphi procedures" OR

"Delphi assessment" OR "Delphi assessments" OR "Delphi approach" OR "Delphi approaches" OR "Delphi panel" OR "Delphi panels" OR "Delphi round" OR "Delphi rounds" OR "Delphi analysis" OR "Delphi expert" OR "Delphi experts" OR "Delphi consultation" OR "Delphi methodology" OR "nominal group technique" OR "nominal group techniques" OR "nominal group" OR "nominal groups" OR "nominal grouping" OR "consensus recommendation" OR "consensus recommendations" OR "consensus development" OR "consensus activity" OR "consensus activities" OR "Consensus Development Conference" OR "Consensus Development" OR "Consensus methodology" OR "consensus method*" OR "RAND") OR KW("Delphi Technique" OR "Delphi Technique" OR "Delphi techniques" OR "Delphi method" OR "Delphi methods" OR "Delphi study" OR "Delphi studies" OR "Delphi survey" OR "Delphi surveys" OR "Delphi consensus" OR "Delphi based consensus" OR "Delphi questionnaire" OR "Delphi questionnaires" OR "Delphi research" OR "Delphi review" OR "Delphi reviews" OR "Delphi process" OR "Delphi processes" OR "Delphi based" OR "Delphi procedure" OR "Delphi procedures" OR "Delphi assessment" OR "Delphi assessments" OR "Delphi approach" OR "Delphi approaches" OR "Delphi panel" OR "Delphi panels" OR "Delphi round" OR "Delphi rounds" OR "Delphi analysis" OR "Delphi expert" OR "Delphi experts" OR "Delphi consultation" OR "Delphi methodology" OR "nominal group technique" OR "nominal group techniques" OR "nominal group" OR "nominal groups" OR "nominal grouping" OR "consensus recommendation" OR "consensus recommendations" OR "consensus development" OR "consensus activity" OR "consensus activities" OR "Consensus Development Conference" OR "Consensus Development" OR "Consensus methodology" OR "consensus method*" OR "RAND" OR (("Guidelines" OR "guideline") N2 ("consensus" OR "delphi")))) AND (TI("quality of reporting" OR "reporting quality" OR "reporting qualities" OR "selective reporting" OR "poor reporting" OR "poor reported" OR "reporting guideline" OR "reporting" OR ("reporting" AND ("quality" OR "selective" OR "poor" OR "weak" OR "manner" OR "rigor" OR "improv*")) OR "Data Accuracy" OR "quality assessment" OR "strengths" OR "strength" OR "weaknesses" OR "weakness" OR "research method" OR "research methods" OR "research method*") OR KW("quality of reporting" OR "reporting quality" OR "reporting qualities" OR "selective reporting" OR "poor reporting" OR "poor reported" OR ("reporting" N5 ("quality" OR "selective" OR "poor" OR "weak" OR "manner" OR "rigor" OR "improv*")) OR "Data Accuracy" OR "Research Report standards" OR "quality assessment" OR "strengths" OR "strength" OR "weaknesses" OR "weakness" OR "research method" OR "research methods" OR "research method*") OR AB("quality of reporting" OR "reporting quality" OR "reporting qualities" OR "selective reporting" OR "poor reporting" OR "poor reported" OR ("reporting" N5 ("quality" OR "selective" OR "poor" OR "weak" OR "manner" OR "rigor" OR "improv*")) OR "Data Accuracy" OR "Research Report standards" OR "quality assessment" OR "strengths" OR "strength" OR "weaknesses" OR "weakness")))

Author, year	
**Assessor**	

**Background**<br>1.1 Does the study suggest anything about how or if consensus papers should report the context or rationale for choosing a consensus method over other methods?	
**Background**<br>1.2 Does the study suggest anything about how/what or if consensus papers should report the objectives of the consensus exercise?	

**Methods**<br>2.1 Does the study suggest anything about how/what or if consensus papers should report regarding:<br>A literature search/strategy?	
**Methods**<br>2.2 Does the study the suggest anything about how/what or if consensus papers should report regarding:<br>Inclusion and exclusion criteria for the literature search?	
**Methods**<br>2.3 Does the study suggest anything of what or if consensus report should report on panel composition, n of participants, expertise, origin? Prespecified?	
**Methods**<br>2.4 Does the study suggest anything of how or if PPI (public patient involvement) activity should be reported?	
**Methods**<br>2.5 Does the study suggest anything about what or if consensus papers should report regarding panel recruitment strategies, invitations? Any level of detail specified?	
**Methods**<br>2.6 Does the study suggest how or if consensus papers should report the consensus criteria/threshold (or the level of agreement considered to reach consensus)?	
**Methods**<br>2.7 Does the study suggest how or if consensus papers should report how decision of approval of an item will be made?	
**Methods**	

For peer review only

2.8 Does the study suggest anything about what level of detail should be reported regarding the number of Delphi rounds or if this should be reported?	
**Methods**<br>2.9 Does the study suggest anything about what level of detail should be reported regarding the criteria used for defining the number of rounds? (why 2-3 or more e.g.) or if this should be reported?	
**Methods**<br>2.10 Does the study suggest anything about the details that should be reported regarding the time between rounds, if it should be prespecified or if this should be reported?	
**Methods**<br>2.11 Does the study suggest anything about details that should be reported of the names of the techniques of non-Delphi methods used to gather participants' inputs and reach consensus?	
**Methods**<br>2.12 Does the study suggest anything of what or in which detail should be reported regarding tool or electronic system used for Delphi? (If Delphi was used)? Or if this should be reported?	
**Methods**<br>2.13 Does the study suggest anything about how or in what level of detail the anonymity of participants (in Delphi or other methods) has to be reported? Or if this should be reported?	
**Methods**<br>2.14 Does the study suggest anything about how to report, and in what level of detail, the feedback for panellists (in Delphi rounds or other methods) process? Or if this should be reported?	
**Methods**<br>2.15 Does the study suggest anything about how or if data synthesis/analysis should be reported (from any consensus method used and how this was calculated statistically) and in what level of detail?	
**Methods**<br>2.16 Does the study suggest anything about how or if piloting should be reported and in what level of detail (e.g. understanding of consensus items, platforms used, tools used)?	
**Methods**	

For peer review only

2.17 Does the study suggest anything about how or if the role of Steering Committee members should be reported?	
**Methods**<br>2.18 Does the study suggest anything on what or if should be described regarding COI or funding?	
**Methods**<br>2.19 Does the study suggest anything on what should be described of how is dealt with COI of panellist (not allowed to vote when there is COI)? Or if this should be described	

**Results**<br>3.1 Does the study suggest anything on how to report the initial evidence search (presentation of results of the literature review)?	
**Results**<br>3.2 Does the study suggest anything on how to report n of studies found?	
**Results**<br>3.3 Does the study recommend which detail should be used when reporting panellists drop-outs (numbers and reasons)? Or if this should be reported?	
**Results**<br>3.4 Does the study suggest how or if approval rates per item shared with respondents for each round should be reported in the Results section?	
**Results**<br>3.5 Does the study suggest anything about in which detail the items that have been dropped should be reported? (reasons e.g.) Or if this should be reported?	
**Results**<br>3.6 Does the study make any recommendation on how to report the collection, synthesis and use of comments from panellists? Or if this should be reported?	
**Results**<br>3.7 Does the study suggest regarding how the final list of items (for clinical guideline or reporting guideline) should be reported? Or if this should be reported?	

**Discussion**<br>4.1 Does the paper suggest anything about reporting the limitations and strengths of the study and how? Or if this should be reported?	
**Discussion**	

For peer review only

4.2 Does the paper suggest anything about what or in which detail the applicability generalisability, and reproducibility of the study should be reported? Or if this should be reported?	

**5.1 Any other item proposed by the paper that is not captured in other columns?**	
**5.2 Any other item not proposed by the paper, but you think that could be added (not fitting the categories above)?**	

**Examples of text with well reported methods/results (for E&E document) - write NA if none was cited or found by you**	
**Additional comments from assessor**	

For peer review only

**Background**<br>1.1 Does the study suggest anything about how or if consensus papers should report the context or rationale for choosing a consensus method over other methods?	1) Research problem clearly defined and topic and method justification should be reported [Hasson 2000, Figure 1 and page 1013]<br><br>2) Selection of one consensus method over another should be evident if the purpose is clearly stated. [Humphrey-Murto 2017 Med Teach page 16]<br><br>3) What is the rationale for selecting the Delphi procedure? [Humphrey-Murto 2019, Figure 1]<br><br>4) The choice of the Delphi technique as a method of systematically collating expert consultation and building consensus needs to be well justified. A rationale for the choice of the Delphi technique as the most suitable method needs to be provided [Jünger 2017, Box 3, items 1 and 8]
**Background**<br>1.2 Does the study suggest anything about how/what or if consensus papers should report the objectives of the consensus exercise?	1) Define the study objective [Boulkedid 2011, Table 5 page 7]<br><br>2) Define the purpose of the study [Chan 2019, Box 1]<br><br>3) Is the objective of the Delphi study to present results (eg, a list or statement) reflecting the consensus of the group, or does the study aim to merely quantify the level of agreement? [Diamond 2014, Table 6 and page 403] If the aim of the Delphi study is to elicit consensus, then a clear definition for what constitutes consensus should be provided a priori together with threshold values that specify when consensus is reached. If the investigators plan to only quantify the degree of consensus, but not have consensus as a criterion to stop the Delphi study, this should also be explicitly stated [Diamond 2014, page 406]<br><br>4) Research problem clearly defined and topic and method justification should be reported [Hasson 2020, Figure 1 and page 1013]<br><br>5) Authors must provide a clear purpose for their study or line of inquiry [Humphrey-Murto 2017 Med Teach, page 16]<br><br>6) The purpose of the study should be clearly defined and demonstrate the appropriateness of the use of the Delphi technique as a method to achieve the research aim. A rationale for the choice of the Delphi technique as the most suitable method needs to be provided [Jünger 2017, item 8]

For peer review only

	The Delphi technique is a flexible method and can be adjusted to the respective research aims and purposes. Any modifications should be justified by a rationale and be applied systematically and rigorously" [Jünger 2017, item 2]

**Methods**<br>2.1 Does the study the suggest anything about how/what or if consensus papers should report regarding:<br>A literature search/strategy?	1) Describe the selection and preparation of the scientific evidence for the participants [Chan 2019, Box 1]<br><br>2) A literature review should be reported [Hasson 2000, Figure 1]<br><br>3) "We suggest that this important step must be described", but they don't say how. [Humphrey-Murto 2017 AMA, page 1493 and 1496 Partially]<br><br>4) Describe the selection and preparation of the scientific evidence for the participants [Humphrey-Murto 2017 Med Teach, page 16]<br><br>5) Only implying it should happen and be reported [Resemann 2018]
**Methods**<br>2.2 Does study the suggest anything about how/what or if consensus papers should report regarding:<br>Inclusion and exclusion criteria for the literature search?	1) Clear definition of the selection criteria and/or the definition used in the Delphi questionnaire; criteria for selection should be reported [Boulkedid 2011, Table 5, Appendix S1 item 2]<br><br>2) Describe how items were selected for inclusion in questionnaire, in sufficient detail [Chan 2019, Box 1]<br><br>3) Clear selection criteria should be prespecified [Paré 2013 page 210]
**Methods**<br>2.3 Does the study suggest anything of what or if consensus report should report on panel composition, n of participants, expertise, origin? Prespecified?	1) The method used to select participants is stated. Number and type of participant subgroups (eg, patients, generalists and experts) are needed [Banno 2019, page 2 item 1]<br><br>2) The method to include and exclude participants was described. The number and type of participant subgroups (e.g., patients, generalists, and experts) were essential to record [Banno 2020, page 52 item 1]<br><br>3) How the experts were chosen (e.g., willingness to participate, expertise, or membership in an organization); Composition and characteristics of the panel, number of participants (diagram of participant flow); number invited, how they were chosen, whether they were described (age, sex, specialty), years of experience, single or from multiple

For peer review only

	specialties, inclusion of multiple stakeholders, types of stakeholders [Boulkedid 2011, page 2, Table 5, Appendix S1 item 9-15]<br><br>4) Describe how participants were selected and their qualifications. Include description of facilitator credentials [Chan 2019, Box 1]<br><br>5) Were criteria for participants reproducible? How will participants be selected or excluded? [Diamond 2014, Table 5 and 6]<br><br>6) Was there heterogeneity in panel membership and is the method for selection of experts clearly defined [Gattrell 2019, Table 1]<br><br>7) Expert selection process and characteristics should be reported in detail [Hasson 2000, page 1009, 1013]<br><br>8) How many participants were involved? We noted that the type of expertise required of participants was usually not clearly described [Humphrey-Murto 2017 AMA, page 1493 and 1494]<br><br>9) Describe how the participants were selected and their qualifications: if the NGT or RAND/UCLA is used, describe facilitator's credentials. Whatever the makeup of the expert panel, the authors must provide a rationale and justify their choices [Humphrey-Murto 2017 Med Teach]<br><br>10) How many stakeholder/participant groups will be involved in each step? Provide a rationale for inclusion or exclusion and define the stakeholder groups [Humphrey-Murto 2019, Fig 4]<br><br>11) Criteria for the selection of experts and transparent information on recruitment of the expert panel, sociodemographic details including information on expertise regarding the topic in question, (non)response and response rates over the ongoing iterations should be reported [Jünger 2017, Box 3 9]<br><br>12) Describing expert panel selection with eligibility criteria and including conflicts of interest [Ng 2018]<br><br>13) The number of experts in each round should be stated. The backgrounds of the experts should be reported, what kind of expertise they possessed, and the criteria according to which they were selected [Niederberger 2020, page 4]

For peer review only

	14) Explicit procedures for expert selection; Clear selection criteria; Clear selection criteria should be prespecified and may include the candidates' years of related experience, or tenure in a position that is relevant to the subject under study Report the response rate to the initial call for participation; provide detailed information about the participating experts (profile) to better allow judgments about their credibility [Paré 2013, page 210, Table 3]<br><br>15) Explain how groups were chosen. Consensus Development Panels: Panel composition: the panel should be made up of experts in the field; the publication should report on how they were chosen and why; [Waggoner 2016, page 665, 667]<br><br>16) Implied by mentioning that detailed information on participants was lacking in some reporting guidelines. Page 5 Report specialties of experts, names and institutions, the selection criteria [Wang 2015]
**Methods**<br>2.4 Does the study suggest anything of how or if PPI (public patient involvement) activity should be reported	No data
**Methods**<br>2.5 Does the study suggest anything about what or if consensus papers should report regarding panel recruitment strategies, invitations? Any level of detail specified?	1) The use of specific methods to encourage the experts to respond (e.g., stamped addressed envelope for returning the questionnaire and financial compensation) [page 2] and recommendation to report whether special techniques were used to invite participants [Boulkedid 2011, Appendix S1 item 21]<br><br>2) Criteria for the selection of experts and transparent information on recruitment of the expert panel, socio- demographic details including information on expertise regarding the topic in question, (non)response and response rates over the ongoing iterations should be reported" [Jünger 2017, Box 3, 9]<br><br>3) provide a detailed description of the expert recruitment and selection process [Paré 2013, page 215 first bullet on the right]<br><br>4) method of obtaining participants should be described [Waggoner 2016, page 667]
**Methods**	1) The method used to define a consensus among panel members; , whether the percentage of agreement was determined; Whether a cut-off (e.g., median value) was used to select indicators [page 2] Consensus definition at each

For peer review only

2.6 Does the study suggest how or if consensus papers should report the consensus criteria/threshold (or the level of agreement considered to reach consensus)?	round [page 7, Appendix item 28] how was consensus obtained [page 7, Appendix item 28] definition of consensus should be reported [Boulkedid 2011, table 5]<br><br>2) Clearly describe how consensus was defined [Chan 2019, Box 1]<br><br>3) Need to define criteria for consensus and to document the degree of agreement together with the results of the Delphi process. Should be defined a priori. [Diamond 2014, page 404 and table 6]<br><br>4) Was the agreement/consensus threshold predefined? [Gattrell 2019, table 1]<br><br>5) Box 2 Specific threshold for the chosen measure (e.g., median of at least 7 on a nine-point scale and an interquartile range of less than 2) [Grant 2018, p 97]<br><br>6) Determine the criteria and the meaning of `consensus' in relation to the studies [Hasson 2020, page 1013]<br><br>7) No. They do state that "articulating the definition of consensus used" was identified as "particularly problematic and were often left out or poorly described", and that "the most concerning issue we identified was that consensus was often not defined a priori. Only 43.2% of the articles we reviewed reported their definition of consensus at the start of the study." But they do not suggest how to report. [Humphrey-Murto 2017 AMA]<br><br>8) Clearly describe how consensus was defined [Humphrey-Murto 2017 Med Teach, page 18]<br><br>9) suggests definition of consensus should be reported [Humphrey-Murto 2019, table 1, also fig 1 and page 1044]<br><br>10) Definition of consensus. Unless not reasonable due to the explorative nature of the study, an a priori criterion for consensus should be defined. This includes a clear and transparent guide for action on (a) how to proceed with certain items or topics in the next survey round, (b) the required threshold to terminate the Delphi process and (c) procedures to be followed when consensus is (not) reached after one or more iterations". Definition and attainment of consensus. It needs to be comprehensible to the reader how consensus was achieved throughout the process, including strategies to deal with non-consensus". "If an a priori definition of consensus is not realistic due to the explorative nature of the study, it should be identified and established by the research team in the course of the process." [Jünger 2017, item 12]

For peer review only

	11) How was consensus defined and measured? What role did the stability of the answers play? [Niederberger 2020, Table 2] Whether and when consensus was defined in the Delphi studies. Was consensus defined a priori in advance of development of the questionnaire. [Niederberger 2020, Table 5] How was consensus measured, e.g. percentage agreement, units of central tendency (especially median) or a combination of percent agreement within a certain range and for a certain threshold. [Niederberger 2020, page 6]<br><br>12) NGT explain criteria used to determine how and when a consensus was met Consensus Development Panels: Explain what constituted consensus and how this was assessed. [Waggoner 2016, page 665] Delphi Explain what constituted consensus and how this was assessed. [Waggoner 2016, page 667]<br><br>13) The endpoint of consensus [Wang 2015, page 5]
**Methods**<br>2.7 Does the study suggest how or if consensus papers should report how decision of approval of an item will be made?	1) Whether the percentage of agreement was determined [page 2] We recorded the method used to define a consensus among panel members, whether the percentage of agreement was determined, and whether a cut-off (e.g., median value) was used to select [Boulkedid 2011, Appendix S1 item 16 (technique method)]<br><br>2) Reporting on each round separately illustrates clearly the array of themes generated in round one and gives an indication of the strength of support for each round. The presentations of findings are important and findings from subsequent rounds should be reported in a summarized format to indicate the relative standing of each of the opinions. [Hasson 2020, page 1013]<br><br>3) (Non)response and response rates over the ongoing iterations should be reported [Jünger 2017, item 9]
**Methods**<br>2.8 Does the study suggest anything about what level of detail should be reported regarding the number of Delphi rounds or if this should be reported?	1) Was the number of rounds to be performed stated (not how it should be reported, but implies it should be) [Banno 2019, page 2 under item 2]<br><br>2) Was the number of rounds to be performed stated? [Banno 2020, 3.4, table 3]<br><br>3) Describe the number of rounds planned [Chan 2019, Box 1]

For peer review only - http://bmjopen.bmj.com/site/about/guidelines.xhtml

	4) Specify a maximum number of rounds [page 404] what was the reason to stop the delphi [Diamond 2014, table 3] What criteria will be used to determine to stop the Delphi process or will the Delphi be run for a specific number of rounds only [Diamond 2014, table 6, table 1 item 2]<br><br>5) number and outline per round should be reported also page 1013 [Hasson 2020, fig 1]<br><br>6) Describe the number of rounds planned and/or criteria for terminating the process [Humphrey-Murto 2017 Med Teach, page 17]<br><br>7) Only implying that x number of rounds are necessary [Humphrey-Murto 2017 AMA]<br><br>8) The methods employed need to be comprehensible; information about the number and design of survey rounds, [Jünger 2017, Box 3 item 10]<br><br>9) Not specifically under item 4 in table 2 report of the specific process used? How many rounds were used in the Delphi technique [Niederberger 2020]<br><br>10) If a study goes beyond the agreed number of rounds (review suggests 2 rounds are required), this should be explained [Waggoner 2016, page 667]
**Methods**<br>2.9 Does the study suggest anything about what level of detail should be reported regarding the criteria used for defining the number of rounds? (why 2-3 or more e.g.) or if this should be reported?	1) Implied in Banno 2020 The prespecified criteria for stopping the Delphi process, other than a statement of the number of rounds, were clarified [Banno 2020]<br><br>2) Describe the number of rounds planned and criteria for terminating the process [Chan 2019, Box 1]<br><br>3) Describe the number of rounds planned and/or criteria for terminating the process [Humphrey-Murto 2017 Med Teach, page 17]<br><br>4) They, imply that the number of rounds is an important thing to report -- but they do not state this as a suggestion.[Humphrey-Murto 2017 AMA]<br><br>5) Will the number of rounds be decided a priori? If not determined a priori, what are the criteria for terminating the process? [Humphrey-Murto 2019, Fig 1]

For peer review only

	6) What was the rationale for the number of rounds; when was the number of rounds defined [Niederberger 2020, page 6]<br>7) Table 3 Report the stopping [Paré 2013]<br>8) For delphi: if a study goes beyond two rounds, explain reason for doing so; [Waggoner 2016, page 667]
**Methods**<br>2.10 Does the study suggest anything about the details that should be reported regarding the time between rounds, if this should be prespecified in advance, or if this should be reported?	1) The time taken to complete the Delphi procedure was recorded [Boulkedid 2011, page 2]
**Methods**<br>2.11 Does the study suggest anything about details that should be reported of the names of the techniques of non-Delphi methods used to gather participants' inputs and reach consensus ?	1) Whether the meeting was held before, after, or between Delphi rounds and what the participants did during the meeting [Boulkedid 2011, page 2]
**Methods**<br>2.12 Does the study suggest anything of what or in which detail	1) What software will be used to administer the Delphi? [Humphrey-Murto 2019, fig 1]

For peer review only

should be reported regarding tool or electronic system used for Delphi? (If Delphi was used)? Or if this should be reported?	
**Methods**<br>2.13 Does the study suggest anything about how or in what level of detail the anonymity of participants (in Delphi or other methods) has to be reported? Or if this should be reported?	1) No, only that it is a limitation of this study that the quality score did not include that. So actually they feel it should be reported how anonymity was maintained [Banno 2020]<br><br>2) Describe how anonymity was defined [Chan 2019, Box 1]<br><br>3) Were responses anonymized [Gattrell 2019, table 1]<br><br>4) It suggests that conducting anonymous iterative mail or e-mail questionnaire rounds is one of the steps [p 1491]. While the authors may have assumed that readers would understand that anonymity was part of their study design, we suggest that they state this, given the variability in approaches that have been labelled as modified consensus methods. [Humphrey-Murto 2017 AMA, page 1497]<br><br>5) Describe how anonymity was maintained. Authors must clearly state how this was accomplished. It is achieved through the use of mail outs in Delphi and RAND/UCLA and private ranking in NGT. [Humphrey-Murto 2017 Med Teach, page 18]<br><br>6) How will anonymity be maintained? [Humphrey-Murto 2019, fig 1]<br><br>7) Ensure the anonymity of the participants. The anonymity of the experts was reported in virtually all of the studies [Paré 2013]
**Methods**<br>2.14 Does the study suggest anything about how to report, and in what level of detail, the feedback for panellists (in	1) Whether the experts were informed of both the response of the group and their own individual response (individual feedback) to each item. The type of feedback, which was defined as qualitative when a summary of the panel's comments was sent to each participant and quantitative when simple statistical summaries illustrating the collective opinion (e.g., central tendency and variance) were sent to each participant [page 2] After each round, each participant should be given the panel results (median, lowest, and highest ratings), the participant's response, and a summary of all comments received. These data inform each participant of his or her position relative to the rest of the group, thus assisting in decisions about replies during future Delphi rounds. [Boulkedid 2011, page 8] It has been recommended that

For peer review only

Delphi rounds or other methods) process? Or if this should be reported?	feedback should include qualitative comments and statistical measures [citation 51, Murphy 1998]. More specifically, we determined whether the experts were informed of both the response of the group and their own individual response (individual feedback) to each item [Boulkedid 2011]<br><br>2) Describe the type of feedback provided after each round [Chan 2019, Box 1]<br><br>3) Were participants' responses in each round reported back to the group, and were responses anonymized? [Gattrell 2019, Table 1]<br><br>4) Give attention to issues which guide data collection: the discovery of opinions, the process of determining the most important issues referring to the design of the initial round, and the management of opinions [Hasson 2020, page 1013]<br><br>5) Was formal feedback provided? If so, was the feedback described? [page 1493], areas that need to be improved with reporting providing participants with feedback of group ratings [Humphrey-Murto 2017 AMA, page 1494]<br><br>6) Describe the type of feedback provided after each round [page 18]. Feedback to participants can include quantitative and/or qualitative data. It also involves two types of agreement: the extent to which individual participants agree with an issue, and the extent to which participants agree with one another. Quantitative feedback may include summary statistics such as the participants' score, participants' medians, range of scores and the proportion of participants selecting each point on a scale. Participants are provided an opportunity to change their ranking, but it should be made clear that they do not need to conform. Researchers may ask the participants who are outliers to provide written justification for their choices (qualitative data) [Humphrey-Murto 2017 Med Teach]<br><br>7) What type of feedback will participants received after each round? [2019] indicates feedback between rounds should include individuals' scores for each item and the distribution of votes by participant group. Some, however, preferred to view aggregated feedback as well as feedback to individual participants [Humphrey-Murto 2019 Yes page 1042, table 1]<br><br>8) How was the feedback designed? [Niederberger 2020, table 2]<br><br>9) Citation [Schmidt, 54] recommends three relevant pieces of feedback that can be provided to experts in phase 3 in addition to mean ranks, namely, the interpretation of Kendall's W from the previous round, the percentage of experts

For peer review only

	placing each item in the top half of their list and the relevant comments that were made by the other panellists [Paré 2013, page 213]<br><br>10) They imply that it should be reported that panellist feedback was collected to inform subsequent Delphi rounds [Resemann 2018]<br><br>11) not about reporting but they state "57 % were silent about how the feedback after consensus was dealt with." suggesting that they felt it needs to be reported. [page 2] only that some reporting guidelines described the feedback information requirement, or gave the methods for feedback collection [Wang 2015, page 6]
**Methods**<br>2.15 Does the study suggest anything about how or if data synthesis/analysis should be reported (from any consensus method used and how this was calculated statistically) and in what level of detail?	1) It is important that standards and norms for prospectively defining analysis plans are needed to improve the credibility of Delphi processes for informing health research, practice, and policy [Grant 2018, page 97]<br><br>2) The methods employed need to be comprehensible; information about methods of data analysis, processing and synthesis of experts' responses to inform the subsequent survey round [Box 3] {Jünger 2017]<br>Reporting of results for each round separately is highly advisable in order to make the evolving of consensus over the rounds transparent. This includes figures showing the average group response, changes between rounds, as well as any modifications of the survey instrument such as deletion, addition or modification of survey items based on previous rounds." [Jünger 2017, item 13]<br><br>3) Detailing statistical analyses and interpretation in arriving at final agreed values [Ng 2018, item 7]<br><br>4) The statistical analyses should be reported [Paré 2013, page 211]<br><br>5) Consensus Development Panels: Statistical analysis: must be reasonable for the research question, and should be as rigorous as possible [Waggoner 2016, page 665]
**Methods**<br>2.16 Does the study suggest anything about how or if piloting should be reported and in what	1) Pilot testing with a small group of individuals is suggested before implementation [Humphrey-Murto 2017 Med Teach, page 16]<br><br>2) All material provided to the expert panel at the outset of the project and throughout the Delphi process should be carefully reviewed and piloted in advance in order to examine the effect on experts' judgements and to prevent bias. [Box 3] The methods employed need to be comprehensible; this includes information on preparatory steps (How was

For peer review only

level of detail (e.g. understanding of consensus items, platforms used, tools used)?	available evidence on the topic in question synthesised?), piloting of material and survey instruments, design of the survey instrument(s), the number and design of survey rounds, methods of data analysis, processing and synthesis of experts' responses to inform the subsequent survey round and methodological decisions taken by the research team throughout the process [Jünger 2017]<br><br>3) Pre-test task instructions and questionnaire instruments [Paré 2013]
**Methods**<br>2.17 Does the study suggest anything about how or if the role of Steering Committee members should be reported?	No data
**Methods**<br>2.18 Does the study suggest anything on what or if should be described regarding COI or funding?	1) 'Sources of funding (industry, non-industry)'as items associated with reporting quality [Banno 2019, page 2]<br><br>2) Is the funding source clearly disclosed? [table 1] Is the role of the funder clearly disclosed? [table 1] Is the funding of any external support (e.g. with the Delphi panel meeting/questionnaires, or medical writing support for the final manuscript) clearly disclosed? [Gattrell 2019]<br><br>3) "Prevention of bias. Researchers need to take measures to avoid directly or indirectly influencing the experts' judgements. If one or more members of the research team have a conflict of interest, entrusting an independent researcher with the main coordination of the Delphi study is advisable" [Jünger 2017]<br><br>4) Describing expert panel selection with eligibility criteria and including conflicts of interest [Ng 2018]
**Methods**<br>2.19 Does the study suggest anything on what should be described of how is dealt with COI of panellist (not allowed	1) No. It only deals with COI as a planning/methodological procedure, not reporting. "5. Prevention of bias. Researchers need to take measures to avoid directly or indirectly influencing the experts' judgements. If one or more members of the research team have a conflict of interest, entrusting an independent researcher with the main coordination of the Delphi study is advisable"[Jünger 2017]

For peer review only

to vote when there is COI)? Or if this should be described	

**Results**<br>3.1 Does the study suggest anything on how to report the initial evidence search (presentation of results of the literature review)?	1) No, but they suggest it should be reported [Jünger 2017]
**Results**<br>3.2 Does the study suggest anything on how to report n of studies found?	No data
**Results**<br>3.3 Does the study recommend which detail should be used when reporting panellists drop-outs (numbers and reasons)? Or if this should be reported?	1) No but it states that number the response rate for the first round dropped to 170 (66.1%). [page 1494]; areas that need improvement in reporting the number of participants after each round [page 1496] Other analyses of consensus methods research found similar poor reporting of this feature, with 7% to 39% of studies reporting response rates for all rounds of data collection [Humphrey-Murto 2017 AMA]<br><br>2) Fig 1 step 7 How will non-responders be managed, i.e. will they be excluded in subsequent rounds What response rate will be acceptable for each stakeholder group in each round? [Humphrey-Murto 2019]<br><br>3) Reporting of results for each round separately is highly advisable in order to make the evolving of consensus over the rounds transparent. This includes figures showing the average group response, changes between rounds, as well as any modifications of the survey instrument such as deletion, addition or modification of survey items based on previous rounds [Jünger 2017, Box 3]<br><br>4) Outlining participation and attrition rates for each round [Ng 2018]

For peer review only

	5) report the response rate to the initial request for participation, the size of the panel and the retention rate; [Paré 2013, page 215 3rd bullet]
**Results**<br>3.4 Does the study suggest how or if approval rates per item shared with respondents for each round should be reported in the Results section?	1) Response rate for each round [Boulkedid 2011, Table 5 on page 7]<br><br>2) Yes Box 1 report response rates and results after each round [Chan 2019]<br><br>3) Response rates for each round should be reported, presentation of total of issues generated in round 1, and presentation of results in round 2 indicating strength of support [Hasson 2000, figure 1 and page 1013]<br><br>4) Report response rates and results after each round [Humphrey-Murto 2017 Med Teach, page 18]<br><br>5) it should report response rates for all rounds [Humphrey-Murto 2019, page 1042]<br><br>6) Reporting of results for each round separately is highly advisable in order to make the evolving of consensus over the rounds transparent. This includes figures showing the average group response, changes between rounds, as well as any modifications of the survey instrument such as deletion, addition or modification of survey items based on previous rounds." [Jünger 2017, item 13]Criteria for the selection of experts and transparent information on recruitment of the expert panel, socio- demographic details including information on expertise regarding the topic in question, (non)response and response rates over the ongoing iterations should be reported". [Jünger 2017]<br><br>7) Reporting both quantitative results and textual comments for each round of analysis [Ng 2018]<br><br>8) How high was the response rate from the experts both when initially approached and also for the individual rounds [Niederberger 2020, Table 2]<br><br>9) Level of consensus should be reported [Resemann 2018]
**Results**<br>3.5 Does the study suggest anything about in which detail the items that have been dropped should	1) Were the criteria for dropping clear; are stopping criteria, other than rounds, reported [Banno 2019, item 3 and 4]<br><br>2) Were the criteria for dropping items clear? (yes, no, or not applicable) [Banno 2020, 2.6 item 3]<br><br>3) Clear criteria for dropping or combining items should also be specified based on the level of agreement or disagreement with individual items. One of the limitations of a priori specification is that certain items may fall just below the

For peer review only

be reported? (reasons e.g.) Or if this should be reported?	threshold for what is fundamentally an arbitrary cut off. In the event that items, believed to be important fell just below the threshold for inclusion in the study, the authors could consider including these items as posteriori considerations provided that sufficient justification was provided. [page 405] Suggested quality criteria: Were criteria for dropping items clear; Stopping criteria other than rounds specified? [Table 5] Were items dropped? What criteria will be used to determine which items to drop? [Diamond 2014, Table 6]<br><br>4) No, but they state Interpretation and processing of results. Consensus does not necessarily imply the correct answer or judgement; (non)consensus and stable disagreement provide informative insights and highlight differences in perspectives concerning the topic in question and Definition and attainment of consensus. It needs to be comprehensible to the reader how consensus was achieved throughout the process, including strategies to deal with non-consensus [Jünger 2017 in Box 3]<br><br>5) Were criteria defined for dropping items [Niederberger 2020, page 6]
**Results**<br>3.6 Does the study make any recommendation on how to report the collection, synthesis and use of comments from panellists? Or if this should be reported?	1) It has been recommended that feedback should include qualitative comments and statistical measures [Murphy 1998, 51]. After each round, each participant should be given the panel results (median, lowest, and highest ratings), the participant's response, and a summary of all comments received [Boulkedid 2011]<br><br>2) Describe the type of feedback provided after each round. Quantitative feedback may include summary statistics such as the participants' score, participants' medians, range of scores and the proportion of participants selecting each point on a scale. Participants are provided an opportunity to change their ranking, but it should be made clear that they do not need to conform [Humphrey-Murto 2017 Med Teach]<br><br>3) Reporting of results for each round separately is highly advisable in order to make the evolving of consensus over the rounds transparent. This includes figures showing the average group response, changes between rounds, as well as any modifications of the survey instrument such as deletion, addition or modification of survey items based on previous rounds [Jünger 2017, item 13]<br><br>4) Ask experts to justify their rankings. Have experts comment and validate consolidated list [page 210 Table 3]. Did experts consolidate the list of items; Did experts comment on and validate the list of items; Was the final number of items reported.<br>Report whether panel members had the opportunity to justify or clarify their own reasoning and to comment on the responses of the other experts as well as on the progress of the panel as a whole. [Paré 2013, page 213].

For peer review only

	Were panellists able to revise previous statements [Paré 2013]<br><br>5) No, but implied that it should be: did not report collecting panellist feedback to inform subsequent Delphi stages [Resemann 2018]
**Results**<br>3.7 Does the study suggest regarding how the final list of items (for clinical guideline or reporting guideline) should be reported? Or if this should be reported?	1) Partially. It says it should be detailed and disseminated, but it does not suggest how (in what format) it should be reported [Jünger 2017]<br><br>2) Suggests "detailing statistical analyses and interpretation in arriving at final agreed values" [Ng 2018]<br><br>3) Report final number of items [Paré 2013, page 210 Table 3]<br><br>4) No but again imply "reported the number of statements assessed." [Resemann 2018]

**Discussion**<br>4.1 Does the paper suggest anything about reporting the limitations and strengths of the study and how? Or if this should be reported?	1) Address potential methodological issues (e.g lack of consensus) or limitations in the discussion (e.g. low response rate) [Chan 2019, Box 1]<br><br>2) Interpretation of consensus gained/not gained [Hasson 2020, page 1009]<br><br>3) In the discussion the authors should address issues that may have impacted the results such as poor response rates between rounds, lack of participation from a select group or geographic region, or lack of consensus. [Humphrey-Murto 2017 Med Teach, page 18]<br><br>4) Methodological issues should be reported [Humphrey-Murto 2019, figure 1]<br><br>5) Reporting should include a critical reflection of potential limitations and their impact of the resulting guidance". [Jünger 2017]
**Discussion**<br>4.2 Does the paper suggest anything about what or in	1) Page 5: is considered a good measure if it meets criteria including reliability, sensitivity, specificity, and feasibility (or applicability) [20,31]. The common use of these characteristics can facilitate acceptance and implementation of indicators developed [Boulkedid 2011]

For peer review only

which detail the applicability generalisability, and reproducibility of the study should be reported? Or if this should be reported?	2) The conclusions should adequately reflect the outcomes of the Delphi study with a view to the scope and applicability of the resulting practice guidance. [Jünger 2017, item 15]<br><br>3) It is also necessary to discuss the critical and rationalistic criteria for the validity and reliability of the studies and the more constructivist characteristics of credibility, transparency, and transferability. [Niederberger 2020, page 8]

**5.1 Any other item proposed by the paper that is not captured in other columns?**	1) Were criteria for dropping items clear? Are stopping criteria, other than rounds, specified [Banno 2019]<br><br>2) Differences between the protocol and the article [Banno 2020, 2.9]<br><br>3) Geographic scope of the survey [page 2]. Main methods used to send the questionnaires (e.g., mail, E-mail, or fax). [Boulkedid 2011, page 7]<br>The formulation of the questionnaire items (e.g., open questions, rating of quality indicators, or both). [Boulkedid 2011]<br>Whether the quality indicators were rated (in which case, we recorded the minimum and maximum values on the rating scale). [Boulkedid 2011]<br>A flow chart of quality indicators (figure showing the output and input indicators at each round) and/or for a written description of indicator flow. [Boulkedid 2011, page 3]<br>Quality indicators used in the first round versus the end of the last round. [Boulkedid 2011, page 3]<br>Availability of the questionnaires in the article itself or in an appendix [Boulkedid 2011, page 3]<br>Whether selection criteria changed between rounds [Boulkedid 2011, page 5]<br>Whether panelists were able to make comments. [Boulkedid 2011, page 6]<br>Whether there was a meeting; at what stage it took place and how people participated [Boulkedid 2011]<br>Response rate for each round [Boulkedid 2011, page 7]<br>preparation in advance of starting Delphi (outcome indicators, structure indicators, process indicators) [Boulkedid 2011, In appendix S1, item 1]<br>METHODS<br>We evaluated the relationship between the response rate and the use of specific methods to encourage the experts to respond (e.g., stamped addressed envelope for returning the questionnaire and financial compensation). Also on maybe we should add item regarding encouragement of participants [Boulkedid 2011, page 2, page 5 right column]<br>Geographic scope of Delphi consensus procedure [Boulkedid 2011,item 20 of appendix and table 5]<br>Question format (open questions, rating scale?) Also in table 5 how were questions formulated? [Boulkedid 2011, item 24

For peer review only

	appendix]<br>Rating scale [Boulkedid 2011, item 25]<br>Methods used to send questionnaire (email fax, mail) [Boulkedid 2011, table 5]<br>Time to complete questionnaire reporting of differences in response rate in rounds [Boulkedid 2011]<br>Number of rounds necessary to reach consensus [Boulkedid 2011]<br>Duration of the procedure [Boulkedid 2011]<br>Is questionnaire added as appendix? [Boulkedid 2011]<br>For Discussion: Validity [Boulkedid 2011]<br><br>4) Outline each step of the process. If modifications were made, provide a rationale for your choices. [Chan 2019]<br>Describe the selection and preparation of the scientific evidence for the participants. [Chan 2019]<br>Include a description of the facilitator's credentials. [Chan 2019]<br>What background material was provided to participants. [Chan 2019]<br>What formal feedback of group rating was shared between rounds [Chan 2019]<br><br>5) Specify stopping criteria in the absence of consensus [Diamond 2014]<br><br>6) Were the questions formulated or validated by an expert panellist [Gattrell 2019]<br><br>7) Researchers conducting consensus-oriented Delphi processes should prospectively and completely register the intended procedure for identifying which items reach consensus. [Grant 2018]<br>The analysis procedure for determining consensus for Delphi processes should be chosen a priori ideally before starting the first round but at the very latest before completing data collection to improve the validity of findings. [Grant 2018]<br>Health researchers conducting consensus-oriented Delphi processes should commit themselves in advance to an analytic procedure for determining which items reach consensus before they see the actual data (or, ideally, before they even collect the data). [Grant 2018]<br>Registrations should be in a publicly available and independently controlled platform that time-stamps entries [Grant 2018]<br><br>8) "Copy of each round questionnaire illustrated" [Hasson 2020]<br>statistical interpretation for the reader [Hasson 2020]<br>appendices to include the questionnaires [Hasson 2020]<br>For Discussion interpretations of consensus gained/not gained reliability and validity [Hasson 2020]

For peer review only

	9) *Page 1493(2) Was background information provided to the participants? pg 1496 areas appeared particularly problematic and were often left out or poorly described: providing background information to participants<br>AND so a clear description of what information was provided and in what format is important<br>* (3) Was the consensus method used for item generation, ranking, or both?<br>* (11) Was consensus forced?<br>Was mail/e-mail polling or face-to-face questioning used? [Humphrey-Murto 2017 AMA]<br><br>10) Outline each step of the process: if modifications were made, provide a rationale for the choices made. Providing justification for the choices made will also add credibility. [Humphrey-Murto 2017 Med Teach]<br><br>11) Background provided to participants, what is level of detail provided [Humphrey-Murto 2019]<br>Figure 1 clear outline of the overall process involved and where Delphi fits [Humphrey-Murto 2019, figure 1]<br>How sample size is determined of participants [Humphrey-Murto 2019, figure 1]<br><br>12) Any modifications should be justified by a rationale and be applied systematically and rigorously [Jünger 2017, Box 3]<br>All material provided to the expert panel at the outset of the project and throughout the Delphi process should be carefully reviewed and piloted in advance in order to examine the effect on experts' judgements and to prevent bias [Jünger 2017]<br>It is recommended to have the final draft of the resulting guidance on best practice in palliative care reviewed and approved by an external board or authority before publication and dissemination [Jünger 2017, Box 3]<br>information about methodological decisions taken by the research team throughout the process Jünger 2017, Box 3]<br>Flow chart to illustrate the stages of the Delphi process, including a preparatory phase, the actual Delphi rounds, interim steps of data processing and analysis, and concluding steps [Jünger 2017, Box 3]<br>Publication and dissemination [Jünger 2017, Box 3]<br><br>13) Item 2-4 and 9 appending revised questionnaires [Ng 2018]<br><br>14) Specific definition of underlying Delphi technique (or as I thought it is important to define exactly what method is used, especially if a modified method is used this needs to be very clear [Niederberger 2020]<br>What role did the stability of the answers play? [Niederberger 2020, table 2]<br>Questionnaire and scale development How were the questionnaires and the specific items for a Delphi technique

For peer review only

	developed? [Niederberger 2020]<br>Nevertheless, it is important to precisely describe, justify, and methodologically reflect on any modifications [Niederberger 2020]<br>How were the questionnaires and the specific items for a Delphi technique developed? [Niederberger 2020, Table 2]<br>Were items identified from empirical analyses such as qualitative interviews or focus groups that were completed in advance or were taken from existing guidelines. [Niederberger 2020, Complementary AND page 6<br>Was the first (qualitative) round of questions in the Delphi process used to generate the items for a standardized questionnaire. [Niederberger 2020, Complementary AND page 6]<br><br>15) Was the final number of items reported [Paré 2013, Table 3] Were items randomly ordered [Paré 2013, Table 3]<br><br>16) Describe the rating scales used [Resemann 2018] the number of statements assessed should be reported [Resemann 2018]<br><br>17) For nominal group process, the research question used to prompt the panel must be clear and concise to obtain valid suggestions from panel members. [Waggoner 2016, page 665] The heterogeneity should be reported [Waggoner 2016, page 665] Evaluation of reliability [Waggoner 2016, page 665]<br><br>18) Meeting attendance; format (e.g. face-to-face); agenda preparation; materials sent to participants prior to meeting; duration of meeting [Wang 2015, page 5] Flow diagram [Wang 2015, page 3] Should we add something regarding other consensus methods including an item regarding face to face meetings? [Wang 2015, page 5]
**5.2 Any other item not proposed by the paper, but you think that could be added (not fitting the categories above)?**	1) Are stopping criteria, other than rounds, specified? [Banno 2019, page 2]<br><br>2) Information letter explaining the method and the reasons their participation to the whole process would be necessary, as well as a form for collecting their consent to complete the entire Delphi process. [Boulkedid 2011]<br><br>3) "Round 1: presentation of total number of issues generated" [Hasson 2020]<br><br>4) This paper was "pointing fingers", showing what was wrong, without suggesting solutions. However, we can be inspired by the critics to build the following list of items: 1) Purpose of the consensus study<br>Whether a literature review was done to support the selection of items [Humphrey-Murto 2017 AMA]<br><br>5) Length of the background provided [Humphrey-Murto 2019]

For peer review only

	Purpose of study: outcome/diagnosis/intervention? [Humphrey-Murto 2019]

**Examples of text with well reported methods/results (for E&E document) - write NA if none was cited or found by you**	1) Page 7 Table 5 [Boulkedid 2011]<br>2) Box 1 [Chan 2019]<br>3) Might have a look at table 6 [Diamond 2014]<br>4) Table 1 [Gattrell 2019]<br>5) Parts of Fig 1 and checklist page 1013 [Hasson 2020]<br>6) Table 1 lists "exemplary publications" for nominal group process, consensus development panel and Delphi technique Page 667 references studies that were "Very descriptive" of the statistical techniques used. [Waggoner 2016]
**Additional comments from assessor**	1) Limited value; protocol for Banno 2020 [Banno 2019]<br>2) Of limited use. The authors developed a 4-point quality score that they applied to Delphi publications [Banno 2020]<br>3) Excellent resource [Boulkedid 2011]<br>4) Focusses on defining consensus [Diamond 2014]<br>5) Congress poster only [Gattrell 2019]<br>6) Study used RAND's ExpertLens as the Delphi platform [Grant 2018]<br>7) 1497: The lack of consensus on consensus methods makes it imperative that researchers provide clear and detailed reporting of the methods they used and that they justify these choices. [Humphrey-Murto 2017]

For peer review only

	8) Page 1044 A suggestion to improv uniformity is to use a software program that provides structure and help with reporting all relevant outcomes (e.g. DelphiManager, http://comet-initiative.org/delphimanager/) [Humphrey-Murto 2019]<br><br>9) Very informative [Jünger 2017]<br><br>10) The study focusses on information systems. Arguably, this is not within the inclusion criteria for the search [Paré 2013]<br><br>11) Review covers nominal group process, consensus development panel and Delphi technique [Waggoner 2016]<br><br>12) Study looked at the reporting quality of reporting guidelines [Wang 2015]

For peer review only

ACcurate COnsensus Reporting Document (ACCORD): Summary of extracted data from literature search

Table of Contents

1. ***Background*** ***2***
2. ***Methods*** ***3***
3. ***Results*** ***9***
4. ***Discussion*** ***11***
5. ***Additional topics*** ***12***

References ***14***

For peer review only

Section: Background

1. Background

Data extraction question	Articles	Checklist item(s) with brief explanation
1.1. Does the study suggest anything about how or if consensus papers should report the context or rationale for choosing a consensus method over other methods?	Hasson F, *et al. J Adv Nurs* 2000[1]<br>Humphrey-Murto S, *et al. Med Teach* 2017[2]<br>Humphrey-Murto S, *et al. J Rheumatol* 2019[3]<br>Jünger S, *et al. Palliat Med* 2017[4]	State the rationale for use of consensus method over other options.<br>*Should consider other consensus methods as well as other methodology types.*
1.2. Does the study suggest anything about how/what or if consensus papers should report the objectives of the consensus exercise?	Hasson F, *et al. J Adv Nurs* 2000[1]<br>Humphrey-Murto S, *et al. Med Teach* 2017[2]<br>Jünger S, *et al. Palliat Med* 2017[4]<br>Boulkedid R, *et al. PLoS One* 2011[5]<br>Chan TM, *et al. CJEM* 2019[6]<br>Diamond IR, *et al. J Clin Epidemiol* 2014[7]	Clearly define study objectives.<br>*Could include presentation of group consensus, or just to quantify the level of agreement.*

For peer review only

Section: Methods

2. Methods

Data extraction question	Articles	Checklist item(s) with brief explanation
2.1. Does the study suggest anything about how/what or if consensus papers should report regarding:<br><br>A literature search/strategy?	Hasson F, *et al. J Adv Nurs* 2000[1]<br>Humphrey-Murto S, *et al. Med Teach* 2017[2]<br>Chan TM, *et al. CJEM* 2019[6]<br>Humphrey-Murto S, *et al. Acad Med* 2017[8]<br>Resemann HK, *et al. Curr Med Res Opin* 2018[9]	A) Describe the strategy for reviewing the existing scientific evidence that informed the study.<br>*If no existing literature is available, the extent of the search should be described.*<br>B) Describe how existing scientific evidence will be provided to the participants.<br>*If different participant groups are involved, it should be stated which information will be provided to which group.*
2.2. Does the study suggest anything about how/what or if consensus papers should report regarding:<br><br>Inclusion and exclusion criteria for the literature search?	Boulkedid R, *et al. PLoS One* 2011[5]<br>Chan TM, *et al. CJEM* 2019[6]<br>Paré G, *et al. Inf Manag* 2013[10]	Describe the process of the literature search.<br>*Should include inclusion and exclusion criteria, and state whether these were prespecified.*
2.3. Does the study suggest anything of what or if consensus report should report on panel composition, n of participants, expertise, origin? Prespecified?	Hasson F, *et al. J Adv Nurs* 2000[1]<br>Humphrey-Murto S, *et al. Med Teach* 2017[2]<br>Humphrey-Murto S, *et al. J Rheumatol* 2019[3]<br>Jünger S, *et al. Palliat Med* 2017[4]<br>Boulkedid R, *et al. PLoS One* 2011[5]<br>Chan TM, *et al. CJEM* 2019[6]<br>Diamond IR, *et al. J Clin Epidemiol* 2014[7]<br>Humphrey-Murto S, *et al. Acad Med* 2017[8]<br>Paré G, *et al. Inf Manag* 2013[10]<br>Banno M, *et al. J Clin Epidemiol* 2019[11]<br>Banno M, *et al. J Clin Epidemiol* 2020[12]<br>Gattrell WT, *et al. Curr Med Res Opin* 2019[13]	A) Describe the structure of the study's participants.<br>*Should describe inclusion of a Chair/Co-chairs, steering committee, and subgroups, if applicable.*<br>B) Explain how panel participants were selected.<br>*Should state who was responsible for panellist selection, the selection criteria applied, the justification for choosing panellist numbers and selection criteria, and whether criteria were prespecified.*

For peer review only

Section: Methods

Data extraction question	Articles	Checklist item(s) with brief explanation
	Ng J. *Value Health* 2018[14]<br>Niederberger M, *et al. Front Public Health* 2020[15]<br>Waggoner J, *et al. Acad Med* 2016[16]<br>Wang X, *et al. BMC Med Res Methodol* 2015[17]	*C)* Describe the composition of the panel. *Should include number of participants at all stages of the process, sociodemographics (e.g. age, sex, specialty, type and duration of relevant experience). Should also describe panel subgroups, if relevant.*<br>D) Describe the expertise of the panel. *Should include the definition of "expert" and description of any public or patients involved.*<br>E) Describe the facilitator(s), if used. *Should include type and duration of relevant experience, and the role played in the process.*
2.4. Does the study suggest anything of how or if PPI (public patient involvement) activity should be reported	No data	Describe the role and involvement of any public or patients.<br>*Should detail the stage(s) at which they were involved, and their roles and contributions.*
2.5. Does the study suggest anything about what or if consensus papers should report regarding panel recruitment strategies, invitations? Any level of detail specified?	Jünger S, *et al. Palliat Med* 2017[4]<br>Boulkedid R, *et al. PLoS One* 2011[5]<br>Paré G, *et al. Inf Manag* 2013[10]<br>Waggoner J, *et al. Acad Med* 2016[16]	Describe how the panel members were recruited.<br>*Could include communication/advertisement method(s) and locations.*
2.6. Does the study suggest how or if consensus papers should report the consensus criteria/threshold (or the level of agreement considered to reach consensus)?	Hasson F, *et al. J Adv Nurs* 2000[1]<br>Humphrey-Murto S, *et al. Med Teach* 2017[2]<br>Humphrey-Murto S, *et al. J Rheumatol* 2019[3]<br>Jünger S, *et al. Palliat Med* 2017[4]<br>Boulkedid R, *et al. PLoS One* 2011[5]<br>Chan TM, *et al. CJEM* 2019[6]<br>Diamond IR, *et al. J Clin Epidemiol* 2014[7]<br>Humphrey-Murto S, *et al. Acad Med* 2017[8]<br>Gattrell WT, *et al. Curr Med Res Opin* 2019[13]	A) Define the consensus measure to be used. *Could include percentage agreement, units of central tendency (e.g. median), a categorical rating (e.g. Agree/Strongly agree) or a combination of percent agreement within a certain range.*<br>B) State the threshold for the group achieving consensus. *Should include whether the threshold was*

For peer review only - http://bmjopen.bmj.com/site/about/guidelines.xhtml

Section: Methods

Data extraction question	Articles	Checklist item(s) with brief explanation
	Niederberger M, *et al. Front Public Health* 2020[15]<br>Waggoner J, *et al. Acad Med* 2016[16]<br>Wang X, *et al. BMC Med Res Methodol* 2015[17]<br>Grant S, *et al. J Clin Epidemiol* 2018[18]	*pre-defined and highlight any threshold variation between rounds, with explanation for the change. If the intention is to quantify the degree of consensus but not to use consensus as a stop criterion for the study, this should be stated.*
2.7. Does the study suggest how or if consensus papers should report how decision of approval of an item will be made?	Hasson F, *et al. J Adv Nurs* 2000[1]<br>Jünger S, *et al. Palliat Med* 2017[4]<br>Boulkedid R, *et al. PLoS One* 2011[5]	Explain how final consensus was reached.<br>*Should describe the evolution of themes between voting rounds, if applicable.*
2.8. Does the study suggest anything about what level of detail should be reported regarding the number of Delphi rounds or if this should be reported?	Hasson F, *et al. J Adv Nurs* 2000[1]<br>Humphrey-Murto S, *et al. Med Teach* 2017[2]<br>Jünger S, *et al. Palliat Med* 2017[4]<br>Chan TM, *et al. CJEM* 2019[6]<br>Diamond IR, *et al. J Clin Epidemiol* 2014[7]<br>Humphrey-Murto S, *et al. Acad Med* 2017[8]<br>Banno M, *et al. J Clin Epidemiol* 2019[11]<br>Banno M, *et al. J Clin Epidemiol* 2020[12]<br>Niederberger M, *et al. Front Public Health* 2020[15]<br>Waggoner J, *et al. Acad Med* 2016[16]	State how many voting rounds were conducted.<br>*Should include whether the number of rounds was prespecified, and whether this was an absolute or a maximum. If the maximum was exceeded, should explain the reasoning for doing so.*
2.9. Does the study suggest anything about what level of detail should be reported regarding the criteria used for defining the number of rounds? (why 2-3 or more e.g.) or if this should be reported?	Humphrey-Murto S, *et al. Med Teach* 2017[2]<br>Humphrey-Murto S, *et al. J Rheumatol* 2019[3]<br>Chan TM, *et al. CJEM* 2019[6]<br>Humphrey-Murto S, *et al. Acad Med* 2017[8]<br>Paré G, *et al. Inf Manag* 2013[10]<br>Banno M, *et al. J Clin Epidemiol* 2020[12]<br>Niederberger M, *et al. Front Public Health* 2020[15]<br>Waggoner J, *et al. Acad Med* 2016[16]	Explain the rationale for choosing the number of voting rounds.<br>*Should also describe the stop criteria, if used, and whether these were prespecified.*
2.10. Does the study suggest anything about the details that should be reported regarding the time between rounds, if this should be	Boulkedid R, *et al. PLoS One* 2011[5]	Describe the time period between voting rounds.<br>*Should include whether the period was*

For peer review only

Section: Methods

Data extraction question	Articles	Checklist item(s) with brief explanation
prespecified in advance, or if this should be reported?		*prespecified and highlight differences between inter-round periods, if applicable.*
2.11. Does the study suggest anything about details that should be reported of the names of the techniques of non-Delphi methods used to gather participants' inputs and reach consensus?	Boulkedid R, *et al. PLoS One* 2011[5]<br>Waggoner J, *et al. Acad Med* 2016[16]	Describe any additional methods used alongside the consensus process.<br>*Should include all that were used, e.g. a self-administered questionnaire combined with a group meeting. Should also explain how the consensus process fitted into the overall study methodology.*
2.12. Does the study suggest anything of what or in which detail should be reported regarding tool or electronic system used for Delphi? (If Delphi was used)? Or if this should be reported?	Humphrey-Murto S, *et al. J Rheumatol* 2019[3]	Describe any tools used to administer the voting.<br>*Could detail electronic platforms, if used.*
2.13. Does the study suggest anything about how or in what level of detail the anonymity of participants (in Delphi or other methods) has to be reported? Or if this should be reported?	Humphrey-Murto S, *et al. Med Teach* 2017[2]<br>Humphrey-Murto S, *et al. J Rheumatol* 2019[3]<br>Chan TM, *et al. CJEM* 2019[6]<br>Humphrey-Murto S, *et al. Acad Med* 2017[8]<br>Paré G, *et al. Inf Manag* 2013[10]<br>Banno M, *et al. J Clin Epidemiol* 2020[12]<br>Gattrell WT, *et al. Curr Med Res Opin* 2019[13]	Detail how anonymity of voters was maintained.<br>*Could involve use of mail-outs in a standard Delphi procedure, blinding on an electronic platform, or private ranking in the NGT.*
2.14. Does the study suggest anything about how to report, and in what level of detail, the feedback for panellists (in Delphi rounds or other methods) process? Or if this should be reported?	Hasson F, *et al. J Adv Nurs* 2000[1]<br>Humphrey-Murto S, *et al. Med Teach* 2017[2]<br>Humphrey-Murto S, *et al. J Rheumatol* 2019[3]<br>Boulkedid R, *et al. PLoS One* 2011[5]<br>Chan TM, *et al. CJEM* 2019[6]<br>Humphrey-Murto S, *et al. Acad Med* 2017[8]<br>Resemann HK, *et al. Curr Med Res Opin* 2018[9]<br>Paré G, *et al. Inf Manag* 2013[10]<br>Gattrell WT, *et al. Curr Med Res Opin* 2019[13]<br>Niederberger M, *et al. Front Public Health* 2020[15]	Explain how voting feedback was provided to panellists at the end of each round.<br>*Could include summaries of group voting and/or their own individual responses. Should state whether feedback will be quantitative and/or qualitative, and whether it will be anonymised. If no feedback was provided, this should be stated.*

For peer review only

Section: Methods

Data extraction question	Articles	Checklist item(s) with brief explanation
	Wang X, *et al. BMC Med Res Methodol* 2015[17]	
2.15. Does the study suggest anything about how or if data synthesis/analysis should be reported (from any consensus method used and how this was calculated statistically) and in what level of detail?	Jünger S, *et al. Palliat Med* 2017[4]<br>Paré G, *et al. Inf Manag* 2013[10]<br>Ng J. *Value Health* 2018[14]<br>Waggoner J, *et al. Acad Med* 2016[16]<br>Grant S, *et al. J Clin Epidemiol* 2018[18]	Detail methods used to process responses after each voting round.<br>*Could include statistical analysis methods, if used.*
2.16. Does the study suggest anything about how or if piloting should be reported and in what level of detail (e.g. understanding of consensus items, platforms used, tools used)?	Humphrey-Murto S, *et al. Med Teach* 2017[2]<br>Jünger S, *et al. Palliat Med* 2017[4]<br>Paré G, *et al. Inf Manag* 2013[10]	Describe any piloting of the study materials and/or survey instruments.<br>*Should include the number of individuals in the pilot group and the rationale for their selection. Should also explain any changes made as a result of the pilot. If no pilot was conducted, this should be stated.*
2.17. Does the study suggest anything about how or if the role of Steering Committee members should be reported?	No data	Describe the role(s) of the Steering Committee in the process.<br>*Should also detail the involvement of the Chair/Co-chairs, subgroups, or individual members at relevant stages of the process, if different from the group as a whole.*
2.18. Does the study suggest anything on what or if should be described regarding COI or funding?	Jünger S, *et al. Palliat Med* 2017[4]<br>Banno M, *et al. J Clin Epidemiol* 2019[11]<br>Gattrell WT, *et al. Curr Med Res Opin* 2019[13]<br>Ng J. *Value Health* 2018[14]	A) Disclose any COI of the panellists<br>*Should specify COI of each participant in the panel.*<br>B) Disclose any funding received and the role of the funder.<br>*Should specify the role of the funding source(s), e.g. involvement in the study concept/design, participation of the Steering Committee, for conducting the consensus process, medical writing support for its reporting.*

For peer review only

Section: Methods

Data extraction question	Articles	Checklist item(s) with brief explanation
2.19. Does the study suggest anything on what should be described of how is dealt with COI of panellist (not allowed to vote when there is COI)? Or if this should be described	Jünger S, *et al*. *Palliat Med* 2017[4]	Describe measures taken to avoid influence by any conflicts of interest (COI). *Should include disclosure of COI and how this was accounted for in the methodology, e.g. by limiting voting in case of a specific COI, adjudication by an independent researcher.*

For peer review only

Section: Results

3. Results

Data extraction question	Articles	Checklist item(s) with brief explanation
3.1. Does the study suggest anything on how to report the initial evidence search (presentation of results of the literature review)?	Jünger S, *et al*. *Palliat Med* 2017[4]	Describe how existing scientific evidence was provided to the participants.<br>*Should include relevant specifics of the literature search, e.g. n of studies reported, to provide relevant context for the results. If different participant groups were involved, it should be stated which information was provided to which group.*
3.2. Does the study suggest anything on how to report n of studies found?	No data	Describe the results of the search and number of included studies.
3.3. Does the study recommend which detail should be used when reporting panellists drop-outs (numbers and reasons)? Or if this should be reported?	Humphrey-Murto S, *et al. J Rheumatol* 2019[3]<br>Jünger S, *et al*. *Palliat Med* 2017[4]<br>Humphrey-Murto S, *et al. Acad Med* 2017[8]<br>Paré G, *et al. Inf Manag* 2013[10]<br>Ng J. *Value Health* 2018[14]	A) State the response rates for each voting round.<br>*Should specify n as well as percent, or otherwise indicate attrition/retention rates.*<br>B) State the reasons cited for voter drop-outs at each stage of the process.<br>*Could be provided as an aggregated summary or as individual responses. If this information was not collected, this should be stated.*<br>C) Describe measures undertaken to maintain acceptable response rates.<br>*If threshold rates differ between stakeholder groups, these should be described with explanation.*

For peer review only - http://bmjopen.bmj.com/site/about/guidelines.xhtml

Section: Results

Data extraction question	Articles	Checklist item(s) with brief explanation
3.4. Does the study suggest how or if approval rates per item shared with respondents for each round should be reported in the Results section?	Hasson F, *et al. J Adv Nurs* 2000[1]<br>Humphrey-Murto S, *et al. Med Teach* 2017[2]<br>Humphrey-Murto S, *et al. J Rheumatol* 2019[3]<br>Jünger S, *et al. Palliat Med* 2017[4]<br>Boulkedid R, *et al. PLoS One* 2011[5]<br>Chan TM, *et al. CJEM* 2019[6]<br>Resemann HK, *et al. Curr Med Res Opin* 2018[9]<br>Ng J. *Value Health* 2018[14]<br>Niederberger M, *et al. Front Public Health* 2020[15]	Describe which results that were shared with respondents after each voting round were reported in the final manuscript.<br>*Could include response rates, the type of information presented, summaries of group voting and/or individual responses. If this information is not provided, this should be stated together with the rationale.*
3.5. Does the study suggest anything about in which detail the items that have been dropped should be reported? (reasons e.g.) Or if this should be reported?	Jünger S, *et al. Palliat Med* 2017[4]<br>Diamond IR, *et al. J Clin Epidemiol* 2014[7]<br>Banno M, *et al. J Clin Epidemiol* 2019[11]<br>Banno M, *et al. J Clin Epidemiol* 2020[12]<br>Niederberger M, *et al. Front Public Health* 2020[15]	A) List any voting items that were dropped.<br>B) Explain the rationale for dropping any voting items.<br>*Should state whether the criteria for dropping any items were prespecified.*
3.6. Does the study make any recommendation on how to report the collection, synthesis and use of comments from panellists? Or if this should be reported?	Humphrey-Murto S, *et al. Med Teach* 2017[2]<br>Jünger S, *et al. Palliat Med* 2017[4]<br>Boulkedid R, *et al. PLoS One* 2011[5]<br>Resemann HK, *et al. Curr Med Res Opin* 2018[9]<br>Paré G, *et al. Inf Manag* 2013[10]	Describe how responses were processed prior to reporting.<br>*Should describe methods by which responses were analysed, aggregated or summarised, include whether any statements were revised between voting rounds, and state by whom the information was processed.*
3.7. Does the study suggest regarding how the final list of items (for clinical guideline or reporting guideline) should be reported? Or if this should be reported?	Jünger S, *et al. Palliat Med* 2017[4]<br>Resemann HK, *et al. Curr Med Res Opin* 2018[9]<br>Paré G, *et al. Inf Manag* 2013[10]<br>Ng J. *Value Health* 2018[14]	Report the final outcomes.<br>*Could be quantitative (e.g. summary statistics, score means, medians and/or ranges) and/or qualitative (e.g. aggregated themes from comments). Should be clear, accurately represent the consensus methodology used, and relevant to the field.*

For peer review only - http://bmjopen.bmj.com/site/about/guidelines.xhtml

Section: Discussion

4. Discussion

Data extraction question	Articles	Checklist item(s) with brief explanation
4.1. Does the paper suggest anything about reporting the limitations and strengths of the study and how? Or if this should be reported?	Hasson F, *et al. J Adv Nurs* 2000[1]<br>Humphrey-Murto S, *et al. Med Teach* 2017[2]<br>Humphrey-Murto S, *et al. J Rheumatol* 2019[3]<br>Jünger S, *et al. Palliat Med* 2017[4]<br>Chan TM, *et al. CJEM* 2019[6]	Discuss the study's methodological strengths and limitations.<br>*Should address issues that may impact results, e.g. response rates or representation.*
4.2. Does the paper suggest anything about what or in which detail the applicability generalisability, and reproducibility of the study should be reported? Or if this should be reported?	Jünger S, *et al. Palliat Med* 2017[4]<br>Boulkedid R, *et al. PLoS One* 2011[5]<br>Niederberger M, *et al. Front Public Health* 2020[15]	A) Discuss the reliability of the study.<br>B) Discuss the sensitivity of the study.<br>C) Discuss the specificity of the study.<br>D) Discuss the applicability of the study.<br>E) Discuss the validity of the study.

For peer review only

Section: Additional topics

5. Additional topics

Data extraction question: Any other item proposed by the paper that is not captured in previous sections?

Articles	Checklist item(s) with brief explanation
Humphrey-Murto S, *et al. Med Teach* 2017[3]<br>Jünger S, *et al. Palliat Med* 2017[4]<br>Boulkedid R, *et al. PLoS One* 2011[5]<br>Paré G, *et al. Inf Manag* 2013[10]<br>Banno M, *et al. J Clin Epidemiol* 2020[12]	Explain any deviations from the planned protocol.<br>*Should include any affected stages, including but not limited to change in panel number or composition, number of voting rounds, stopping criteria, statistical plan, reporting of outcomes.*
Boulkedid R, *et al. PLoS One* 2011[5]<br>Resemann HK, *et al. Curr Med Res Opin* 2018[9]	Describe the formulation of questions.<br>*Should include the type of questions, e.g. open questions, numerical rating, level of agreement rating. If rating questions were used, the scale range should be stated, and whether respondents were able to leave additional comments after rating items.*
Boulkedid R, *et al. PLoS One* 2011[5]<br>Wang X, *et al. BMC Med Res Methodol* 2015[17]	Describe any group meetings that were held.<br>*Should state at what stage the meeting took place, objectives/purpose, format (e.g. face-to-face or virtual), pre-read materials shared, attendance, location, duration, and how individuals participated.*
Hasson F, *et al. J Adv Nurs* 2000[1]<br>Boulkedid R, *et al. PLoS One* 2011[5]<br>Ng J. *Value Health* 2018[14]	List any items included in the appendix accompanying the main report.<br>*Could include e.g. full voting questions from each round with response rates, or information provided to the panel as pre-reads or to summarise voting rounds.*
Boulkedid R, *et al. PLoS One* 2011[5]	State how the survey was presented to participants.<br>*For example, as hard copy or via digital platform; could include description of email or mailing process. Should describe any randomisation procedures for questions, if used. If questions were not randomised, this should be stated.*
Boulkedid R, *et al. PLoS One* 2011[5]	Describe incentives for encouraging responses.<br>*Should list any specific methods, e.g. paid return postage for the questionnaire or financial compensation.*
Boulkedid R, *et al. PLoS One* 2011[5]	State the period in which the process was conducted.
Grant S, *et al. J Clin Epidemiol* 2018[18]	Describe any prospective registrations for the consensus process.

For peer review only - http://bmjopen.bmj.com/site/about/guidelines.xhtml

Section: Additional topics

Articles	Checklist item(s) with brief explanation
	Should include the platform on which it was registered and a link, if applicable. If the process was not registered, this should be stated.
Jünger S, *et al*. *Palliat Med* 2017[4]	Describe any external peer review prior to publication. *Should name the authority, state the rationale for their review, and describe any modifications made as a result of their review.*
Humphrey-Murto S, *et al. Med Teach* 2017[3]<br>Jünger S, *et al*. *Palliat Med* 2017[4]	Describe the overall process using a flow chart or diagram.
Paré G, *et al. Inf Manag* 2013[10]<br>Niederberger M, *et al. Front Public Health* 2020[15]	Explain how the initial voting items in the consensus were developed. *Could describe e.g. development from empirical analyses, qualitative interviews, advance focus groups, brainstorming, or existing guidelines. Should state who consolidated the information and developed the voting items.*
Boulkedid R, *et al. PLoS One* 2011[5]	Describe the procedure for collecting participants' consent to complete the full consensus process. *Could briefly describe any forms used and how the data were collected and stored.*

For peer review only

Section: References

References

1. Hasson F, Keeney S, McKenna H. Research guidelines for the Delphi survey technique. *J Adv Nurs* 2000;32:1008-15. doi: 10.1046/j.1365-2648.2000.t01-1-01567.x.
2. Humphrey-Murto S, Varpio L, Gonsalves C, et al. Using consensus group methods such as Delphi and Nominal Group in medical education research. *Med Teach* 2017;39:14-9. doi: 10.1080/0142159X.2017.1245856.
3. Humphrey-Murto S, Crew R, Shea B, et al. Consensus Building in OMERACT: Recommendations for Use of the Delphi for Core Outcome Set Development. *J Rheumatol* 2019;46:1041-6. doi: 10.3899/jrheum.181094.
4. Jünger S, Payne SA, Brine J, et al. Guidance on Conducting and REporting DElphi Studies (CREDES) in palliative care: Recommendations based on a methodological systematic review. *Palliat Med* 2017;31(8):684-706. doi: 10.1177/0269216317690685.
5. Boulkedid R, Abdoul H, Loustau M, et al. Using and Reporting the Delphi Method for Selecting Healthcare Quality Indicators: A Systematic Review. *PLoS One* 2011;6:e20476. doi: 10.1371/journal.pone.0020476.
6. Chan TM, Yarris LM, Humphrey-Murto S. Delving into Delphis. *CJEM* 2019;21:167-9. doi: 10.1017/cem.2019.3.
7. Diamond IR, Grant RC, Feldman BM, et al. Defining consensus: A systematic review recommends methodologic criteria for reporting of Delphi studies. *J Clin Epidemiol* 2014;67:401-9. doi: 10.1016/j.jclinepi.2013.12.002.
8. Humphrey-Murto S, Varpio L, Wood TJ, et al. The Use of the Delphi and Other Consensus Group Methods in Medical Education Research: A Review. *Acad Med* 2017;92:1491-8. doi: 10.1097/ACM.0000000000001812.
9. Resemann HK, Clements S, Griffiths A, et al. Reporting of Delphi methods to achieve consensus on guidelines in rare diseases. *Curr Med Res Opin* 2018;34 (Suppl 1):37.
10. Paré G, Cameron AF, Poba-Nzaou P, et al. A systematic assessment of rigor in information systems ranking-type Delphi studies. *Inf Manag* 2013;50:207-17 doi: 10.1016/j.im.2013.03.003.
11. Banno M, Tsujimoto Y, Kataoka Y. Reporting quality of the Delphi technique in reporting guidelines: a protocol for a systematic analysis of the EQUATOR Network Library. *J Clin Epidemiol* 2019;9:e024942.
12. Banno M, Tsujimoto Y, Kataoka Y. The majority of reporting guidelines are not developed with the Delphi method: a systematic review of reporting guidelines. *J Clin Epidemiol* 2020;124;50-7. doi: 10.1016/j.jclinepi.2020.04.010.
13. Gattrell WT, Clements SJ, Sheard D. Quality assessment of guidelines/recommendations developed using Delphi methodology. *Curr Med Res Opin* 2019;35 (Suppl 2):40. doi: 10.1080/03007995.2019.1583496.
14. Ng J. Delphi method: A qualitative approach for quantitative results. *Value Health* 2018;21:S54. Available at https://www.valueinhealthjournal.com/article/S1098-3015(18)30747-2/pdf.
15. Niederberger M, Spranger J. Delphi Technique in Health Sciences: A Map. *Front Public Health* 2020;8:457. doi: 10.3389/fpubh.2020.00457.

For peer review only - http://bmjopen.bmj.com/site/about/guidelines.xhtml

Section: References

16. Waggoner J, Carline JD, Durning SJ. Is There a Consensus on Consensus Methodology? Descriptions and Recommendations for Future Consensus Research. *Acad Med* 2016;91:663-8. doi: 10.1097/ACM.0000000000001092.
17. Wang X, Chen Y, Yang N, et al. Methodology and reporting quality of reporting guidelines: systematic review. *BMC Med Res Methodol* 2015;15:74. doi: 10.1186/s12874-015-0069-z.
18. Grant S, Booth M, Khodyakov D. Lack of preregistered analysis plans allows unacceptable data mining for and selective reporting of consensus in Delphi studies. *J Clin Epidemiol* 2018;99:96-105. doi: 10.1016/j.jclinepi.2018.03.007.

For peer review only

PRISMA 2020 Checklist

Section and Topic	Item #	Checklist item	Location where item is reported
**TITLE**			
Title	1	Identify the report as a systematic review.	Page 1, 2
**ABSTRACT**			
Abstract	2	See the PRISMA 2020 for Abstracts checklist.	Page 2
**INTRODUCTION**			
Rationale	3	Describe the rationale for the review in the context of existing knowledge.	Page 4, 5
Objectives	4	Provide an explicit statement of the objective(s) or question(s) the review addresses.	Page 5
**METHODS**			
Eligibility criteria	5	Specify the inclusion and exclusion criteria for the review and how studies were grouped for the syntheses.	Page 5
Information sources	6	Specify all databases, registers, websites, organisations, reference lists and other sources searched or consulted to identify studies. Specify the date when each source was last searched or consulted.	Page 6
Search strategy	7	Present the full search strategies for all databases, registers and websites, including any filters and limits used.	Online supplemental material 2
Selection process	8	Specify the methods used to decide whether a study met the inclusion criteria of the review, including how many reviewers screened each record and each report retrieved, whether they worked independently, and if applicable, details of automation tools used in the process.	Page 6
Data collection process	9	Specify the methods used to collect data from reports, including how many reviewers collected data from each report, whether they worked independently, any processes for obtaining or confirming data from study investigators, and if applicable, details of automation tools used in the process.	Page 6, 7
Data items	10a	List and define all outcomes for which data were sought. Specify whether all results that were compatible with each outcome domain in each study were sought (e.g. for all measures, time points, analyses), and if not, the methods used to decide which results to collect.	Online supplemental material 3
	10b	List and define all other variables for which data were sought (e.g. participant and intervention characteristics, funding sources). Describe any assumptions made about any missing or unclear information.	Online supplemental material 3
Study risk of bias assessment	11	Specify the methods used to assess risk of bias in the included studies, including details of the tool(s) used, how many reviewers assessed each study and whether they worked independently, and if applicable, details of automation tools used in the process.	Not applicable
Effect measures	12	Specify for each outcome the effect measure(s) (e.g. risk ratio, mean difference) used in the synthesis or presentation of results.	Not applicable
Synthesis methods	13a	Describe the processes used to decide which studies were eligible for each synthesis (e.g. tabulating the study intervention characteristics and comparing against the planned groups for each synthesis (item #5)).	Not applicable
	13b	Describe any methods required to prepare the data for presentation or synthesis, such as handling of missing summary statistics, or data conversions.	Not applicable
	13c	Describe any methods used to tabulate or visually display results of individual studies and syntheses.	Not applicable
	13d	Describe any methods used to synthesize results and provide a rationale for the choice(s). If meta-analysis was performed, describe the model(s), method(s) to identify the presence and extent of statistical heterogeneity, and software package(s) used.	Not applicable

For peer review only - http://bmjopen.bmj.com/site/about/guidelines.xhtml

PRISMA 2020 Checklist

Section and Topic	Item #	Checklist item	Location where item is reported
	13e	Describe any methods used to explore possible causes of heterogeneity among study results (e.g. subgroup analysis, meta-regression).	Not applicable
	13f	Describe any sensitivity analyses conducted to assess robustness of the synthesized results.	Not applicable
Reporting bias assessment	14	Describe any methods used to assess risk of bias due to missing results in a synthesis (arising from reporting biases).	Not applicable
Certainty assessment	15	Describe any methods used to assess certainty (or confidence) in the body of evidence for an outcome.	Not applicable
**RESULTS**			
Study selection	16a	Describe the results of the search and selection process, from the number of records identified in the search to the number of studies included in the review, ideally using a flow diagram.	Page 7 Fig 1
	16b	Cite studies that might appear to meet the inclusion criteria, but which were excluded, and explain why they were excluded.	Page 8, Fig 1
Study characteristics	17	Cite each included study and present its characteristics.	Page 7
Risk of bias in studies	18	Present assessments of risk of bias for each included study.	Not applicable
Results of individual studies	19	For all outcomes, present, for each study: (a) summary statistics for each group (where appropriate) and (b) an effect estimate and its precision (e.g. confidence/credible interval), ideally using structured tables or plots.	Table 1 Online supplemental material 4 and 5
Results of syntheses	20a	For each synthesis, briefly summarise the characteristics and risk of bias among contributing studies.	Not applicable
	20b	Present results of all statistical syntheses conducted. If meta-analysis was done, present for each the summary estimate and its precision (e.g. confidence/credible interval) and measures of statistical heterogeneity. If comparing groups, describe the direction of the effect.	Not applicable
	20c	Present results of all investigations of possible causes of heterogeneity among study results.	Not applicable
	20d	Present results of all sensitivity analyses conducted to assess the robustness of the synthesized results.	Not applicable
Reporting biases	21	Present assessments of risk of bias due to missing results (arising from reporting biases) for each synthesis assessed.	Not applicable
Certainty of evidence	22	Present assessments of certainty (or confidence) in the body of evidence for each outcome assessed.	Not applicable
**DISCUSSION**			
Discussion	23a	Provide a general interpretation of the results in the context of other evidence.	Page 10, 11
	23b	Discuss any limitations of the evidence included in the review.	Page 3, 11, 12
	23c	Discuss any limitations of the review processes used.	Page 3, 11,

For peer review only

PRISMA 2020 Checklist

Section and Topic	Item #	Checklist item	Location where item is reported
			12
	23d	Discuss implications of the results for practice, policy, and future research.	Page12
**OTHER INFORMATION**			
Registration and protocol	24a	Provide registration information for the review, including register name and registration number, or state that the review was not registered.	Page 1, 5
	24b	Indicate where the review protocol can be accessed, or state that a protocol was not prepared.	Page 5 Online supplemental material 1 ref 13 and 15
	24c	Describe and explain any amendments to information provided at registration or in the protocol.	Online supplemental material 1
Support	25	Describe sources of financial or non-financial support for the review, and the role of the funders or sponsors in the review.	Page 12
Competing interests	26	Declare any competing interests of review authors.	Page 12
Availability of data, code and other materials	27	Report which of the following are publicly available and where they can be found: template data collection forms; data extracted from included studies; data used for all analyses; analytic code; any other materials used in the review.	Online supplemental material 1-5

From: Page MJ, McKenzie JE, Bossuyt PM, Boutron I, Hoffmann TC, Mulrow CD, et al. The PRISMA 2020 statement: an updated guideline for reporting systematic reviews. BMJ 2021;372:n71. doi: 10.1136/bmj.n71

For more information, visit: http://www.prisma-statement.org/

For peer review only

PRISMA 2020 for Abstracts Checklist

Section and Topic	Item #	Checklist item	Reported (Yes/No)
**TITLE**			
Title	1	Identify the report as a systematic review.	Yes
**BACKGROUND**			
Objectives	2	Provide an explicit statement of the main objective(s) or question(s) the review addresses.	Yes
**METHODS**			
Eligibility criteria	3	Specify the inclusion and exclusion criteria for the review.	Yes
Information sources	4	Specify the information sources (e.g. databases, registers) used to identify studies and the date when each was last searched.	Yes
Risk of bias	5	Specify the methods used to assess risk of bias in the included studies.	Not applicable
Synthesis of results	6	Specify the methods used to present and synthesise results.	Not applicable
**RESULTS**			
Included studies	7	Give the total number of included studies and participants and summarise relevant characteristics of studies.	Yes
Synthesis of results	8	Present results for main outcomes, preferably indicating the number of included studies and participants for each. If meta-analysis was done, report the summary estimate and confidence/credible interval. If comparing groups, indicate the direction of the effect (i.e. which group is favoured).	Yes
**DISCUSSION**			
Limitations of evidence	9	Provide a brief summary of the limitations of the evidence included in the review (e.g. study risk of bias, inconsistency and imprecision).	Not applicable
Interpretation	10	Provide a general interpretation of the results and important implications.	Yes
**OTHER**			
Funding	11	Specify the primary source of funding for the review.	Not in abstract, in main document
Registration	12	Provide the register name and registration number.	Yes

From: Page MJ, McKenzie JE, Bossuyt PM, Boutron I, Hoffmann TC, Mulrow CD, et al. The PRISMA 2020 statement: an updated guideline for reporting systematic reviews. BMJ 2021;372:n71. doi: 10.1136/bmj.n71

For peer review only

PRISMA 2020 for Abstracts Checklist

For more information, visit: http://www.prisma-statement.org/

For peer review only

Existing guidance on reporting of consensus methodology: a systematic review to inform ACCORD guideline development

Journal:	*BMJ Open*
Manuscript ID	bmjopen-2022-065154.R1
Article Type:	Original research
Date Submitted by the Author:	20-Jul-2022
Complete List of Authors:	van Zuuren, Esther; Leiden University Medical Center, Department of Dermatology<br>Logullo, Patricia; University of Oxford, CSM (Centre for Statistics in Medicine)<br>Price, Amy; Stanford University School of Medicine<br>Fedorowicz, Zbys; Veritas Health Sciences Consultancy<br>Hughes, Ellen L; Sciwright Limited<br>Gattrell, William T; Ipsen
**Primary Subject Heading**:	Research methods
Secondary Subject Heading:	Health policy
Keywords:	Protocols & guidelines < HEALTH SERVICES ADMINISTRATION & MANAGEMENT, STATISTICS & RESEARCH METHODS, Health policy < HEALTH SERVICES ADMINISTRATION & MANAGEMENT

I, the Submitting Author has the right to grant and does grant on behalf of all authors of the Work (as defined in the below author licence), an exclusive licence and/or a non-exclusive licence for contributions from authors who are: i) UK Crown employees; ii) where BMJ has agreed a CC-BY licence shall apply, and/or iii) in accordance with the terms applicable for US Federal Government officers or employees acting as part of their official duties; on a worldwide, perpetual, irrevocable, royalty-free basis to BMJ Publishing Group Ltd ("BMJ") its licensees and where the relevant Journal is co-owned by BMJ to the co-owners of the Journal, to publish the Work in this journal and any other BMJ products and to exploit all rights, as set out in our licence.

The Submitting Author accepts and understands that any supply made under these terms is made by BMJ to the Submitting Author unless you are acting as an employee on behalf of your employer or a postgraduate student of an affiliated institution which is paying any applicable article publishing charge ("APC") for Open Access articles. Where the Submitting Author wishes to make the Work available on an Open Access basis (and intends to pay the relevant APC), the terms of reuse of such Open Access shall be governed by a Creative Commons licence – details of these licences and which Creative Commons licence will apply to this Work are set out in our licence referred to above.

Other than as permitted in any relevant BMJ Author's Self Archiving Policies, I confirm this Work has not been accepted for publication elsewhere, is not being considered for publication elsewhere and does not duplicate material already published. I confirm all authors consent to publication of this Work and authorise the granting of this licence.

For peer review only

Existing guidance on reporting of consensus methodology: a systematic review to inform ACCORD guideline development

Esther J van Zuuren[1], Patricia Logullo[2], Amy Price[3], Zbys Fedorowicz[4], Ellen L Hughes[5], William T Gattrell[6]

[1]Department of Dermatology, Leiden University Medical Centre, Leiden, The Netherlands

[2]Centre for Statistics in Medicine (CSM), University of Oxford, and EQUATOR Network UK Centre, Oxford, UK

[3]Stanford University School of Medicine, Stanford, CA, USA

[4]Veritas Health Sciences Consultancy, Huntingdon, United Kingdom

[5]Sciwright Limited, Somerset, UK

[6]Global Medical Affairs, Ipsen, Abingdon, UK

Corresponding author: Esther J van Zuuren,
Department of Dermatology B1-Q
Leiden University Medical Centre
Albinusdreef 2
2333ZA Leiden
The Netherlands
email: e.j.van_zuuren@lumc.nl

Word count: 2492

Keywords: systematic review, consensus, Delphi, reporting guideline

For peer review only

ABSTRACT

Objective: To identify evidence on the reporting quality of consensus methodology, and to select potential checklist items for the ACCORD (ACcurate COnsensus Reporting Document) project to develop a consensus reporting guideline.

Design: Systematic review.

Data sources: Embase, MEDLINE, Web of Science, PubMed, Cochrane Library, Emcare, Academic Search Premier and PsycINFO from inception until 7 January 2022.

Eligibility criteria: Studies, reviews and published guidance addressing the reporting quality of consensus methodology for improvement of health outcomes in biomedicine or clinical practice. Reports of studies using or describing consensus methods but not commenting on their reporting quality were excluded. No language restrictions were applied.

Data extraction and synthesis: Screening and data extraction of eligible studies were carried out independently by two authors. Reporting quality items addressed by the studies were synthesized narratively.

Results: Eighteen studies were included: 5 systematic reviews, 4 narrative reviews, 3 research papers, 3 conference abstracts, 2 research guidance papers and 1 protocol. The majority of studies indicated that the quality of reporting of consensus methodology could be improved. Commonly addressed items were: consensus panel composition; definition of consensus; and the threshold for achieving consensus. Items least addressed were: public patient involvement (PPI); the role of the steering committee, chair, co-chair; conflict of interest of panellists; and funding. Data extracted from included studies revealed additional items that were not captured in the data extraction form such as justification of deviation from the protocol or incentives to encourage panellist response.

Conclusion: The results of this systematic review confirmed the need for a reporting checklist for consensus methodology and provided a range of potential checklist items to report. The next step in the ACCORD project builds on this systematic review and focuses on reaching consensus on these items to develop the reporting guideline.

Protocol registration: The protocol is registered at https://osf.io/2rzm9.

For peer review only

STRENGTHS AND LIMITATIONS OF THIS STUDY

- This systematic review utilised a comprehensive search of multiple databases without language restriction
- The included studies ranged from conference abstracts and protocols to guidelines and systematic reviews
- For full transparency and to promote discussion, all data retrieved are reported
- Conclusions are limited by the paucity of studies that provided substantial useful guidance

For peer review only

For peer review only - http://bmjopen.bmj.com/site/about/guidelines.xhtml

INTRODUCTION

Healthcare providers face continuing challenges in making treatment decisions, particularly where available information on a clinical topic is limited, contradictory, or non-existent. In such situations, alternative and complementary approaches underpinned by collective judgement and based on expert consensus may be used.[1-3]

A variety of approaches with differing methodological rigour can be used to achieve consensus-based decisions. These range from informal “expert consensus meetings” to structured or systematic approaches such as the Delphi method and the Nominal Group Technique (NGT). These methods can be used for generating ideas or determining priorities and aim to achieve consensus through voting on a series of multiple-choice questions.[4-7] The voting process varies according to the method and may take place anonymously (as in Delphi) and/or face to face (in NGT and consensus conferences).[8-10] Key elements in the process include the use of valid and reliable methods to reach consensus and subsequently their transparent reporting; however, these aspects are seldom clearly and explicitly reported.[3, 11]

Reporting guidelines have been developed and are in use for the majority of study designs, e.g. PRISMA, CONSORT and STROBE (for all existing reporting guidelines see: https://www.equator-network.org/). However, no research reporting guideline exists for studies involving consensus methodology other than best practice guidance for Delphi studies in palliative care.[12] Guidelines should include “a checklist, flow diagram, or explicit text to guide authors in reporting a specific type of research, developed using explicit methodology”.[3]

Deficiencies in the reporting of consensus methods have been well documented in the literature and are referred to in the protocol for the ACCORD (ACcurate COnsensus Reporting Document) project, which aims to develop a reporting guideline for methods used to reach consensus.[13] In accordance with the EQUATOR Network guidance in the toolkit for the development of reporting guidelines, the

For peer review only - http://bmjopen.bmj.com/site/about/guidelines.xhtml

next step for the ACCORD project was a review of the relevant literature, which would ultimately inform the voting process.[3]

Our objective was to undertake a thorough and comprehensive systematic review that seeks to identify evidence on the quality of reporting of consensus methodology, for subsequent development into a draft checklist of items for the ACCORD guideline. This ACCORD reporting guideline will assist the biomedical research and clinical practice community to describe the methods used to reach consensus in a complete, transparent, and consistent manner.

METHODS

This manuscript conforms to the Preferred Reporting Items for Systematic Reviews and Meta-Analyses (PRISMA) statement,[14] and follows a prespecified protocol (Supplementary Material 1).[13] The protocol was registered on 12 October 2021 at the Open Science Framework (OSF).[15]

Inclusion criteria

Eligible studies consisted of reviews and published guidance which addressed the reporting quality of consensus methodology and aimed to improve health outcomes in biomedicine or clinical practice.

Exclusion criteria

Excluded were publications using consensus methods or describing consensus methods, or discussing the advantages or disadvantages of frameworks, procedures, or techniques to reach consensus, without specifically addressing reporting quality. Examples include guidelines developed through the use of consensus methodologies, such as reporting guidelines, clinical practice guidelines or core outcome set development studies. Editorials (usually brief opinion-based comments), letters about individual publications, and commentaries on consensus methods outside the scope of biomedical research (for example, in the social sciences, economy, politics or marketing) were also excluded for this systematic review.

Literature search strategy

For peer review only - http://bmjopen.bmj.com/site/about/guidelines.xhtml

A systematic literature search was conducted on 7 January 2022 by a biomedical information specialist. The following bibliographical databases were searched: MEDLINE (OVID version), Embase (OVID version), PubMed, Web of Science, MEDLINE (Web of Science), Cochrane Library, Emcare (OVID version), PsycINFO (EbscoHOST version) and Academic Search Premier. The full search strategy is presented in Supplementary Material 2.

We (EJvZ, ZF, PL and WTG) piloted four initial search strategies provided by the information specialist (JWS, see Acknowledgements). The initial search strategy was sensitive and precise, producing the highest number of retrieved references (N = 7951). After several rounds of checking through known relevant references and controlling for the effect of the performance of certain search terms, modifications were made, including the use of the most explicit terms in the most specific search fields. The performance of search terms was investigated from two vantage points: homonymy (same search term, but different meaning), and, particularly, loss-of-context (right meaning of the word, but not in the correct context). This extended search strategy provided extra 'signal', but also reduced the level of 'noise'. We chose to use specific rather than broad terms (for example, not using the singular terms "delphi" and "consensus" instead we included these words with relevant phrases or with other contextual words). In this way, the refined search strategy was better aligned with our inclusion criteria and the objectives of the systematic review.

The final search results were uploaded to Rayyan (https://rayyan.ai) in the blind mode for independent screening by four review authors (EJvZ, ZF, PL, WTG) based on titles and abstracts. No language restrictions were applied. Records deemed eligible or without sufficient detail to make a clear judgement, we retrieved as full-text articles (EJvZ). The same four reviewers independently reassessed the eligibility of these full-text papers and any discrepancies were resolved through discussion. The references of the included studies were also checked for additional potentially eligible studies (EJvZ).

Data extraction

For peer review only - http://bmjopen.bmj.com/site/about/guidelines.xhtml

Study details and outcome data from the included studies were collected independently within Covidence (https://www.covidence.org/) by two authors using a piloted data extraction form (EJvZ, WTG). Disagreements were discussed and reconciled by consultation with a third and fourth author (ZF and AP).

The following details were extracted: bibliographic details and reporting items including any suggestions and comments regarding reporting items. Reporting items were divided into the component parts of background, methods, results and discussion, each addressing key aspects of consensus methodology. We also included a section for additional items retrieved from the studies and not captured in the data extraction form. The complete data extraction form can be found as Supplementary Material 3.

Patient and public involvement

We involved patients, advocates, and members of the lay public in the initial phases of this protocol [13, 15], as collaborators to develop this project and to co-produce the systematic review and co-author the manuscript. They are collaborating with us by offering their experience with the use of consensus methods to develop guidelines and also systematic reviews. These contributors will work with us to disseminate the results.

RESULTS

Our searches across the databases identified 2599 articles and 137 further references to abstracts totalling 2736 references (after removal of duplicates) (see Figure 1). A total of 2682 records were excluded after examination of titles and abstracts. Full-text copies of 54 studies were obtained for further assessment of eligibility, and finally, just 18 eligible studies were included. Checking of the references of these full-text publications did not yield any additional eligible articles.

Characteristics of included studies

Eighteen studies matched our prespecified eligibility criteria and were finally included in this review. These studies comprised five systematic reviews,[12, 16-19] four reviews,[20-23] three research

For peer review only - http://bmjopen.bmj.com/site/about/guidelines.xhtml

papers,[24-26] two research guidelines/guidance,[27, 28] three conference abstracts,[29-31] and one protocol.[32] Of the 18 included studies, 4 used Delphi plus other consensus methods [19, 21, 23 and 28] and the remaining 14 were primarily focused on only the Delphi method.[12, 16-19, 20, 22, 24-27, 29, 30]

Characteristics of excluded studies

A total of 36 studies were excluded.[33- 68] The main reasons for their exclusion were: that they discussed (modified) Delphi methodology but did not include aspects of reporting;[33-54] that they covered reporting but not on consensus methodology;[55-58] that various other consensus methodologies were discussed but not their reporting;[59-67] and that only the concept of experts in consensus methodology was discussed.[68]

Data extraction

The majority of studies indicated that reporting of consensus methods could be improved overall. The authors of these studies summarised some current limitations in reporting or proposed suggestions for improvement. Often there were common generic comments that noted reporting of consensus methodologies is inconsistent or lacks transparency. The studies provided few examples of areas that could be reported in more detail such as: selection criteria for the participants and information about the participants; background information for panellists; definition of consensus; response rates after each round; description of level of anonymity or how anonymity was maintained; and feedback between rounds (see Table 1).

For peer review only - http://bmjopen.bmj.com/site/about/guidelines.xhtml

Table 1 Data on reporting quality of consensus methodologies

**Items that are not or not adequately reported in sufficient detail**	
Selection criteria for participants/information about the participants [16, 19, 23, 26, 32]	Statement that anonymity was maintained or level of anonymity [[20, 21, 25, 28, 29, 32]
Literature review [20, 21, 31]	Type of consensus method used [29]
Background information for participants [20, 21, 25, 28]	Threshold of consensus [29]
Recruitment strategies [19, 22]	How questionnaire was developed [26]
Criteria for number of rounds [16, 26]	Pretesting of instruments [19, 32]
Stopping criteria [16, 32]	Analysis procedure [24, 32]
Feedback after rounds [17, 20, 21, 23, 25, 26, 28, 31, 32]	Changes to registered pre-analysis plan [24]
Rating scales used [31]	Reporting final number of list of items [32]
Criteria for dropping items [26]	Conflict of interest of panellists [29]
Response rates for each round [17 20, 21, 25, 26, 28, 32]	Funding source [29]
Definition of consensus [17-19, 21, 23, 25, 26, 28]	External support [29]
Level of consensus reached [19, 31]	Generic comments that reporting needs improvement [12, 17, 26, 30]

The studies we reviewed did not provide a systematic or standardised evaluation of the quality of reporting, but they did evaluate the literature critically and offered insights into the gaps of information about consensus. Fifteen papers made recommendations sometimes in the form of short lists —based solely on the authors' opinion, rather than using a systematic approach to reporting guidance development.[12, 16-25, 27, 28, 30, 32] Detailed statements regarding quality of reporting are reproduced in Supplementary Material 4.

In Table 2, we summarise the results of the data extraction, which correlates the corresponding aspects of consensus reporting ("items") to the studies that address them. The items in the table are presented in the format used in the data extraction form (see Supplementary Material 3).

For peer review only - http://bmjopen.bmj.com/site/about/guidelines.xhtml

Table 2. Studies providing guidance for reporting items in the extraction form of this systematic review

**Reporting Items**	**Studies that provide guidance**	
Background	Number	References
1.1 Rationale for choosing a consensus method over other methods	4	[12, 25, 27, 28]
1.2 Clearly defined objective	6	[12, 17, 18, 20, 27, 28]
Methods		
2.1 Review of existing evidence informing consensus study	5	[20, 21, 27, 28, 31]
2.2 Inclusion and exclusion criteria of the literature search	3	[17, 20, 22]
2.3 Composition of the panel	16	[12, 16-23, 25-30, 32]
2.4 Public patient involvement (PPI)	0	
2.5 Panel recruitment	4	[12, 17, 22, 23]
2.6 Defining consensus and the threshold for achieving consensus	13	[12, 17-21, 23-29]
2.7 Decision of item approval	3	[12, 17, 27]
2.8 Number of voting rounds	10	[12, 16, 18, 20, 21, 23, 26-28, 32]
2.9 Rationale for number of voting rounds	8	[16, 20, 21-23, 25, 26, 28]
2.10 Time between voting rounds	1	[17]
2.11 Additional methods used alongside consensus	2	[17, 23]
2.12 Software or tools used for voting	1	[25]
2.13 Anonymity of panellists and how this was maintained	7	[16, 20-22, 25, 28, 29]
2.14 Feedback to panellists at the end of each round	11	[17, 19-22, 25-29, 31]
2.15 Synthesis/analysis of responses after voting rounds	5	[12, 22-24, 30]
2.16 Pilot testing of study material/instruments	3	[12, 22, 28]
2.17 Role of the steering committee/chair/co-chair/facilitator	0	
2.18 Conflict of interest or funding received	4	[12, 29, 30, 32]
2.19 Measures to avoid influence by conflict of interest	1	[12]
Results		
3.1 Results of the literature search	1	[12]
3.2 Number of studies found as supporting evidence	0	
3.3 Response rates per voting round	5	[12, 21, 22, 25, 30]
3.4 Results shared with respondents	9	[12, 17, 20, 25-28, 30, 31]
3.5 Dropped items	5	[12, 16, 18, 26, 32]
3.6 Collection, synthesis and comments from panellists	5	[12, 17, 22, 28, 31]
3.7 Final list of items (e.g. for guideline or reporting guideline)	4	[12, 22, 30, 31]
Discussion		
4.1 Limitations and strengths of the study	5	[12, 20, 25, 27, 28]
4.2 Applicability, generalizability, reproducibility	3	[12, 17, 26]

For peer review only

The most frequently addressed item in the included studies (16 times) was the composition of and the criteria for selecting the panellists, including their demographics; specifically, age, gender, specialty, years of experience, and sociodemographic background. The aspects of clarity in, and the importance of, defining consensus and the corresponding thresholds to reach that consensus were addressed in 13 studies. The prespecified number of voting rounds and provision of feedback to the panellists at the end of each round were addressed in 10 and 11 of the studies, respectively.

For peer review only - http://bmjopen.bmj.com/site/about/guidelines.xhtml

None of the included studies reported or made reference to public patient involvement (PPI). The roles of the steering committee/chair/co-chair were not defined in any of the included studies. Reporting of the time interval between voting rounds, panel members' conflicts of interest (COI) and funding sources, as well as the measures used to avoid the influence of COI on voting and decision-making, were minimally addressed.

Conversely, three studies addressed between 12 and 19 reporting items of the 30 items present in the data extraction form of this review,[12, 19, 28] whereas two studies covered only two or three items.[19, 24] We identified a considerable number of other aspects of reporting that were proposed in the included studies, but which were not captured in our data extraction form. These included: 'justifications for deviating from the protocol', 'incentives for encouraging panellists to respond', and 'suggestions to add a flow chart of the consensus process'. All extracted data can be found in Supplementary Material 5 and 6.

DISCUSSION

Although consensus methodology is widely used in healthcare and researchers do raise poor reporting as an issue, we were able to identify only 18 studies that commented on reporting quality and/or provided suggestions to improve the quality of reporting of consensus methodology. These included studies ranged from conference abstracts and protocols to guidelines and systematic reviews. Only four studies covered methods other than the Delphi method and thus providing very limited guidance on other consensus methodologies. As we carried out a comprehensive search of multiple databases without language restriction, it is unlikely that we have missed eligible studies within the period. Comments regarding deficient reporting varied from generic statements such as "reporting could be improved" to rather specific comments of which aspects of consensus methods were inadequately or not reported. Far more detailed data were provided regarding guidance to improve reporting quality or suggestions for items that require reporting. Both composition and characteristics of the panel, and defining consensus and threshold for achieving assessment

For peer review only - http://bmjopen.bmj.com/site/about/guidelines.xhtml

received, were consistently addressed and appeared to be critical items that should be reported in sufficient detail. Feedback to the panel might be considered an important aspect of ensuring ongoing engagement with the panellists, transparency and replicability of methods; thus, it was somewhat surprising to see just 11 of the 18 studies consider this an element of consensus methodology worth reporting.

Some items were not addressed in any of the studies, specifically PPI, which is currently considered a key element in the shared decision-making process and is a component of guideline development.[69] Just four studies made reference to the COI of panel members and project funding. COI of panellists, as well as of chair, co-chair and steering committee, can directly or indirectly impact and influence decision-making during the various steps of consensus methodology. As such, COI remains underreported and is often inconsistently described.[70] This also raises concerns about the measures that can be taken to mitigate the potential influence of COI and to ensure that those panellists who do have relevant interests are, for example, not able to vote on pertinent items. For full transparency and to promote discussion, all data retrieved are reported as supplementary material (Supplementary Material 4–6).

Although conclusions are limited by the paucity of studies, a few were particularly informative. The first was a systematic review on the use and reporting of the Delphi method for selecting healthcare indicators.[17] Specifically, this review not only provided guidance for planning and using the Delphi procedure but additionally formulated general recommendations for reporting. The second study was a guidance report on consensus methods such as Delphi and NGT, which were used in medical education research.[28] The authors reported that there is a lack of “standardization in definitions, methodology and reporting” and proposed items for researchers to consider when using consensus methods to improve methodological rigour as well as the reporting quality. However, it is worth noting that none of these studies followed the EQUATOR Network guidance for the development of a reporting guideline.[3]

For peer review only - http://bmjopen.bmj.com/site/about/guidelines.xhtml

The third study we would like to highlight is the Guidance on Conducting and REporting DElphi Studies (CREDES) in palliative care, which was based on a methodological systematic review.[12] This study focused on the development of guidance in palliative care, although it may not be suitable for extrapolation to other biomedical areas. Furthermore, this study only considered the Delphi methodology, whereas we included studies covering consensus processes involving non-Delphi based methods or “modified Delphi” in our review (and in the ACCORD project overall). However, many of the suggestions made regarding the design and conduct of Delphi studies in addition to recommendations for reporting are equally applicable to our ACCORD project. These items will be used and integrated into the next step of the project, which is the development of a reporting checklist on consensus methods.

Two additional studies proved to be of particular value.[21, 25] One provided a preliminary Delphi checklist to be used for Outcome Measures in Rheumatology (OMERACT).[25] The other concluded, in a scoping review that consensus methods are “poorly standardized and inconsistently used” and exposed reporting flaws in consensus reports.[21]

CONCLUSION

The principal objectives of this systematic review were to conduct a comprehensive search and to identify the existing evidence on the quality of reporting of consensus methodology. As such, we have been able to gather together all relevant studies, summarise the existing research, and highlight key gaps in the current evidence base on consensus methods. This systematic review will ultimately inform the generation of a draft checklist of items for the development steps of the ACCORD reporting guideline.

ACKNOWLEDGMENTS

We would like to thank Jan W. Schoones of the Directorate of Research Policy (formerly: Walaeus Library), Leiden University Medical Centre, Leiden, The Netherlands, for his assistance in the development of the search strategy. Furthermore, we would like to thank the other members of the

For peer review only

For peer review only - http://bmjopen.bmj.com/site/about/guidelines.xhtml

ACCORD project Steering Committee for their contributions to the protocol: Amrit Pali Hungin, Christopher C Winchester, David Tovey, Keith Goldman, Rob Matheis and Niall Harrison. We are grateful for the editorial guidance and reviewer insights from the BMJ Open team. We agree their observations improved the manuscript.

COMPETING INTERESTS

PL is a member of the UK EQUATOR Centre, an organization that promotes the use of reporting guidelines, many of which are developed using consensus methods, and she is personally involved in the development of other reporting guidelines. ELH has worked with Ogilvy Health UK on consensus projects. WTG is a former employee of Ipsen and is now employed by Bristol Myers Squib. AP is an editor at the BMJ, EJvZ and ZF have no conflict of interest.

FUNDING

PL contributed to this study using her time funded by CRUK. WTG contributed his time with the agreement of his employers. Apart from these authors, this study was conducted without external funding for the development of the study design; for the collection, analysis and interpretation of the data; or for the writing of the report. As ZF (Veritas Health Sciences Consultancy) acted as an independent consultant for this work, his support for data extraction and tabulation was funded by Oxford PharmaGenesis. Support for Open Access was also provided by Oxford PharmaGenesis. The decision to submit the paper for publication was made solely by the authors.

CONTRIBUTORS

EJvZ, PL, ZF and WTG contributed to the screening and agreed on the inclusion of studies. EJvZ and WTG extracted data from the included studies. AP, ZF and ELH contributed to the discussion of extracted data and interpretation. EJvZ was the major contributor in the review of studies, data extraction, interpretation of findings as well as writing of the manuscript. All authors read the final manuscript, provided feedback and approved the final manuscript. The author EJvZ is the guarantor.

PATIENT CONSENT FOR PUBLICATION

For peer review only - http://bmjopen.bmj.com/site/about/guidelines.xhtml

For peer review only

No patient data were used in this study and no patient consent for publication was required.

ETHICS APPROVAL

No patient-level data were used in this study and no ethical approval was sought.

PROVENANCE AND PEER REVIEW

Not commissioned; externally peer reviewed.

DISSEMINATION

A link to this research will be made available on the EQUATOR website and the ACCORD research page, shared via social media, shared with future Delphi respondents and stored in local online institutional repositories.

DATA AVAILABILITY STATEMENT

All key data for this study are included in this article or uploaded as online supplementary information. The ACCORD protocol has been listed on the EQUATOR website (Reporting guidelines under development for other study designs | The EQUATOR Network (equator-network.org)) and registered with the Open Science Framework (https://osf.io/2rzm9).

OPEN ACCESS

This is an open access article distributed in accordance with the terms of the Creative Commons Attribution (CC BY 4.0) license, which permits others to distribute, remix, adapt and build upon this work, for commercial use, provided the original work is properly cited. See: http://creativecommons.org/licenses/by/4.0.

ORCID iDs

EJvZ: https://orcid.org/0000-0002-4780-0182

PL: https://orcid.org/0000-0001-8708-7003

For peer review only - http://bmjopen.bmj.com/site/about/guidelines.xhtml

AP: https://orcid.org/0000-0002-5200-7322

ZF: https://orcid.org/0000-0003-3952-965X

ELH: https://orcid.org/0000-0003-2012-0037

WTG: https://orcid.org/0000-0002-9733-6330

REFERENCES

1. Raine R, Sanderson C, Black N. Developing clinical guidelines: a challenge to current methods. *BMJ* 2005;331:631-3. doi: 10.1136/bmj.331.7517.631.
2. Djulbegovic B, Guyatt G. Evidence vs Consensus in Clinical Practice Guidelines. *JAMA* 2019;322:725-6.doi: 10.1001/jama.2019.9751.
3. Moher D, Schulz KF, Simera I, Altman DG. Guidance for developers of health research reporting guidelines. *PLoS Med* 2010;7:e1000217. doi:10.1371/journal.pmed.1000217.
4. Dalkey N, Helmer O. An Experimental Application of the DELPHI Method to the Use of Experts. Manage Sci. 1963;9:458-467. https://doi.org/10.1287/mnsc.9.3.458.
5. Murphy MK, Black NA, Lamping DL, et al. Consensus development methods, and their use in clinical guideline development. *Health Technol Assess* 1998;2:i-iv, 1-88. doi: 10.3310/hta2030.
6. Foth T, Efstathiou N, Vanderspank-Wright B, et al. The use of Delphi and Nominal Group Technique in nursing education: A review. *Int J Nurs Stud* 2016;60:112-20. doi: 10.1016/j.ijnurstu.2016.04.015.
7. Nair R, Aggarwal R, Khanna D. Methods of formal consensus in classification/diagnostic criteria and guideline development. *Semin Arthritis Rheum* 2011;41:95-105. doi: 10.1016/j.semarthrit.2010.12.001.
8. McMillan SS, King M, Tully MP. How to use the nominal group and Delphi techniques. *Int J Clin Pharm* 2016;38:655-62. doi: 10.1007/s11096-016-0257-x.
9. Grant S, Armstrong C, Khodyakov D. Online Modified-Delphi: a Potential Method for Continuous Patient Engagement Across Stages of Clinical Practice Guideline Development. *J Gen Intern Med* 2021;36:1746-50. doi: 10.1007/s11606-020-06514-6.
10. Helmer-Hirschberg O. Analysis of the future: The Delphi Method. Santa Monica, CA: RAND Corporation, 1967. Available at https://www.rand.org/pubs/papers/P3558.htm.
11. Arakawa N, Bader LR Consensus development methods: Considerations for national and global frameworks and policy development. *Res Social Adm Pharm* 2022;18:2222-9. doi: 10.1016/j.sapharm.2021.06.024.
12. Jünger S, Payne SA, Brine J, et al. Guidance on Conducting and REporting DElphi Studies (CREDES) in palliative care: Recommendations based on a methodological systematic review. *Palliat Med* 2017;31(8):684-706. doi: 10.1177/0269216317690685.
13. Gattrell WT, Hungin AP, Price A, et al. ACCORD guideline for reporting consensus-based methods in biomedical research and clinical practice: a study protocol. *Res Integr Peer Rev* May 09, 2022. https://doi.org/10.1186/s41073-022-00122-0.

For peer review only - http://bmjopen.bmj.com/site/about/guidelines.xhtml

14. Page MJ, McKenzie JE, Bossuyt PM, et al. The PRISMA 2020 statement: an updated guideline for reporting systematic reviews. *BMJ* 2021;372:n71. doi: 10.1136/bmj.n71.
15. Gattrell W, Hungin AP, Arents BWMA, et al. Consensus-based methods in biomedical research and clinical practice: The ACCORD study protocol for establishing a reporting guideline. https://osf.io/e5w9n/ (accessed 19 April 2022).
16. Banno M, Tsujimoto Y, Kataoka Y. The majority of reporting guidelines are not developed with the Delphi method: a systematic review of reporting guidelines. *J Clin Epidemiol* 2020;124;50-7. doi: 10.1016/j.jclinepi.2020.04.010.
17. Boulkedid R, Abdoul H, Loustau M, et al. Using and Reporting the Delphi Method for Selecting Healthcare Quality Indicators: A Systematic Review. *PLoS One* 2011;6:e20476. doi: 10.1371/journal.pone.0020476.
18. Diamond IR, Grant RC, Feldman BM, et al. Defining consensus: A systematic review recommends methodologic criteria for reporting of Delphi studies. *J Clin Epidemiol* 2014;67:401-9. doi: 10.1016/j.jclinepi.2013.12.002.
19. Wang X, Chen Y, Yang N, et al. Methodology and reporting quality of reporting guidelines: systematic review. *BMC Med Res Methodol* 2015;15:74. doi: 10.1186/s12874-015-0069-z
20. Chan TM, Yarris LM, Humphrey-Murto S. Delving into Delphis. *CJEM* 2019;21:167-9. doi: 10.1017/cem.2019.3.
21. Humphrey-Murto S, Varpio L, Wood TJ, et al. The Use of the Delphi and Other Consensus Group Methods in Medical Education Research: A Review. *Acad Med* 2017;92:1491-8. doi: 10.1097/ACM.0000000000001812.
22. Paré G, Cameron AF, Poba-Nzaou P, et al. A systematic assessment of rigor in information systems ranking-type Delphi studies. *Inf Manag* 2013;50:207-17 doi: 10.1016/j.im.2013.03.003.
23. Waggoner J, Carline JD, Durning SJ. Is There a Consensus on Consensus Methodology? Descriptions and Recommendations for Future Consensus Research. *Acad Med* 2016;91:663-8. doi: 10.1097/ACM.0000000000001092.
24. Grant S, Booth M, Khodyakov D. Lack of preregistered analysis plans allows unacceptable data mining for and selective reporting of consensus in Delphi studies. *J Clin Epidemiol* 2018;99:96-105. doi: 10.1016/j.jclinepi.2018.03.007.
25. Humphrey-Murto S, Crew R, Shea B, et al. Consensus Building in OMERACT: Recommendations for Use of the Delphi for Core Outcome Set Development. *J Rheumatol* 2019;46:1041-6. doi: 10.3899/jrheum.181094.
26. Niederberger M, Spranger J. Delphi Technique in Health Sciences: A Map. *Front Public Health* 2020;8:457. doi: 10.3389/fpubh.2020.00457.
27. Hasson F, Keeney S, McKenna H. Research guidelines for the Delphi survey technique. *J Adv Nurs* 2000;32:1008-15. doi: 10.1046/j.1365-2648.2000.t01-1-01567.x.
28. Humphrey-Murto S, Varpio L, Gonsalves C, et al. Using consensus group methods such as Delphi and Nominal Group in medical education research. *Med Teach* 2017;39:14-9. doi: 10.1080/0142159X.2017.1245856.
29. Gattrell WT, Clements SJ, Sheard D. Quality assessment of guidelines/recommendations developed using Delphi methodology. *Curr Med Res Opin* 2019;35 (Suppl 2):40. doi: 10.1080/03007995.2019.1583496.

For peer review only

30. Ng J. Delphi method: A qualitative approach for quantitative results. *Value Health* 2018;21:S54. Available at https://www.valueinhealthjournal.com/article/S1098-3015(18)30747-2/pdf.
31. Resemann HK, Clements S, Griffiths A, et al. Reporting of Delphi methods to achieve consensus on guidelines in rare diseases. *Curr Med Res Opin* 2018;34 (Suppl 1):37.
32. Banno M, Tsujimoto Y, Kataoka Y. Reporting quality of the Delphi technique in reporting guidelines: a protocol for a systematic analysis of the EQUATOR Network Library. *J Clin Epidemiol* 2019;9:e024942.
33. Bellali T, Karamitri I. The Delphi research methodology and its applications in the healthcare sciences. *Arch Hellen Med* 2011;28:839-48. Available at http://www.mednet.gr/archives/2011-6/pdf/839.pdf.
34. Bijl R. Delphi in a future scenario study on mental health and mental health care. *Futures* 1992;24:232-50. doi: 10.1016/0016-3287(92)90033-C.
35. Boel A, Navarro-Compán V, Landewé R, et al. Two different invitation approaches for consecutive rounds of a Delphi survey led to comparable final outcome. *J Clin Epidemiol* 2021;129:31-9. doi: 10.1016/j.jclinepi.2020.09.034.
36. Bowles N. The Delphi technique. *Nurs Stand* 1999;13:21-6. doi: 10.7748/ns1999.07.13.45.32.c2650.
37. Cramer CK, Klasser GD, Epstein JB, et al. The Delphi process in dental research. *J Evid Based Dent Pract* 2008;8:211-20. doi: 10.1016/j.jebdp.2008.09.002.
38. de Loë R, Melnychuk N, Murray D, et al. Advancing the State of Policy Delphi Practice: A Systematic Review Evaluating Methodological Evolution, Innovation, and Opportunities. *Technol Forecast Soc Change* 2016;104:78-88. doi: 10.1016/j.techfore.2015.12.009.
39. Drumm S, Bradley C, Moriarty F. 'More of an art than a science'? The development, design and mechanics of the Delphi Technique. *Res Social Adm Pharm* 2022;18:2230-6. doi: 10.1016/j.sapharm.2021.06.027.
40. Fink-Hafner D, Dagan T, Doušak M, et al. Delphi Method: Strengths and Weaknesses. *Metodoloski Zv* 2019;16:1-19. doi: 10.51936/fcfm6982.
41. Franklin KK, Hart JK. Idea Generation and Exploration: Benefits and Limitations of the Police Delphi Research Method. *Innov High Educ* 2006;31:237-46. 10.1007/s10755-006-9022-8.
42. Guzys D, Dickson-Swift V, Kenny A, et al. Gadamerian philosophical hermeneutics as a useful methodological framework for the Delphi technique. *Int J Qual Stud Health Well-being* 2015;10:26291. doi: 10.3402/qhw.v10.26291.
43. Hallowell MR, Gambatese JA. Qualitative Research: Application of the Delphi Method to CEM Research. *J Constr Eng Manag* 2010;136:99-107. doi: 0.1061/(ASCE)CO.1943-7862.0000137.
44. Holloway K. Doing the E-Delphi: using online survey tools. *Comput Inform Nurs* 2012;30:347-50. doi: 10.1097/NXN.0b013e31825e8923.
45. Hung HL, Altschuld JW, Lee YF. Methodological and conceptual issues confronting a cross-country Delphi study of educational program evaluation. *Eval Program Plann* 2008;31:191-8. doi: 10.1016/j.evalprogplan.2008.02.005.
46. Ibiyemi AO, Adnan YM, Daud MN. The validity of the classical Delphi applications for assessing the industrial sustainability-correction factor: an example study. *Foresight* 2016;18:603-24. doi: 10.1108/FS-04-2016-0016.

For peer review only - http://bmjopen.bmj.com/site/about/guidelines.xhtml

47. Keeney S, Hasson F, McKenna HP. Consulting the oracle: ten lessons from using the Delphi technique in nursing research. *J Adv Nurs* 2006;53:205-12. doi: 10.1111/j.1365-2648.2006.03716.x.
48. Keeney S, Hasson F, McKenna HP. A critical review of the Delphi technique as a research methodology for nursing. *Int J Nurs Stud* 2001;38:195-200. doi: 10.1016/S0020-7489(00)00044-4.
49. Kennedy HR. Enhancing Delphi research: methods and results. *J Adv Nurs* 2004;45:504-11. doi: 10.1046/j.1365-2648.2003.02933.x.
50. Khodyakov D, Grant S, Denger B, et al. Practical Considerations in Using Online Modified-Delphi Approaches to Engage Patients and Other Stakeholders in Clinical Practice Guideline Development. *Patient* 2020;13:11-21. doi: 10.1007/s40271-019-00389-4.
51. Murry J, Hammons J. Delphi: A versatile methodology for conducting qualitative research. *Rev High Educ* 2017;18:423-36. doi: 10.1353/rhe.1995.0008.
52. Olsen AA, Wolcott MD, Haines ST, et al. How to use the Delphi method to aid in decision making and build consensus in pharmacy education. *Curr Pharm Teach Learn* 2021;13:1376-85. doi: 10.1016/j.cptl.2021.07.018.
53. Vernon W. The Delphi technique: A review. *Int J Ther Rehabil* 2009;16:69-76. doi: 10.12968/ijtr.2009.16.2.38892.
54. Von der Gracht HA. Consensus measurement in Delphi studies Review and implications for future quality assurance. Technol Forecast Soc Change 2012;79:1525-36. doi: 10.1016/j.techfore.2012.04.013.
55. Bennett C, Khangura S, Brehaut JC, et al. Reporting guidelines for survey research: an analysis of published guidance and reporting practices. *PloS Med* 2010;8:e1001069. doi: 10.1371/journal.pmed.1001069.
56. Han SJ, Oh MC, Sughrue ME, et al. Reporting standard compliance in publications of vestibular schwannoma patients treated with microsurgery. *Otol Neurotol* 2015;2012:33:648-50. doi: 10.1097/MAO.0b013e31824f998b.
57. Moher D, Weeks L, Ocampo M, et al. Describing reporting guidelines for health research: a systematic review. *J Clin Epidemiol* 2011;64:718-42. doi: 10.1016/j.jclinepi.2010.09.013.
58. Murray A, Junge A, Robinson PG, et al. International consensus statement: methods for recording and reporting of epidemiological data on injuries and illnesses in golf. Br J Sports Med 2020;54:1136-41. doi: 10.1136/bjsports-2020-102380.
59. Campbell SM, Cantrill JA. Consensus methods in prescribing research. *J Clin Pharm Ther* 2001;26:5-14. doi: 10.1046/j.1365-2710.2001.00331.x.
60. Durkin J, Usher K, Jackson D. Using consensus from experts to inform a shared understanding of subjective terms. *Nurse Res* 2019;27:46-9. doi: 10.7748/nr.2019.e1622.
61. Fink A, Kosecoff J, Chassin M, et al. Consensus methods: characteristics and guidelines for use. *Am J Public Health* 1984;74:979-83. doi: 0.2105/ajph.74.9.979.
62. Halcomb E, Davidson P, Hardaker L. Using the consensus development conference method in healthcare research. *Nurs Res* 2008;16:56-71. doi: 10.7748/nr2008.10.16.1.56.c6753.
63. James D, Warren-Forward H. Research methods for formal consensus development. *Nurs Res* 2015;22:35-40. doi: 10.7748/nr.22.3.35.e1297.
64. McMillan SS, King M, Tully MP. How to use the nominal group and Delphi techniques. *Int J Clin Pharm* 2016;38:655-62. doi: 10.1007/s11096-016-0257-x.

For peer review only - http://bmjopen.bmj.com/site/about/guidelines.xhtml

65. Nair R, Aggarwal R, Khanna D. Methods of Formal Consensus in Classification/Diagnostic Criteria and Guideline Development. *Semin Arthritis Rheum* 2011;41:95-105. doi: 10.1016/j.semarthrit.2010.12.001.
66. Rotondi AJ, Kvetan V, Carlet J, et al. Consensus conferences in critical care medicine - Methodologies and impact. *Crit Care Clin* 1997;13:417-39. doi: 10.1016/s0749-0704(05)70319-5.
67. van Teijlingen E, Pitchforth E, Bishop C, et al. Delphi method and nominal group technique in family planning and reproductive health research. *J Fam Plann Reprod Health Care* 2006;32:249-52. doi: 10.1783/147118906778586598.
68. Baker J, Lovell K, Harris N. How expert are the experts? An exploration of the concept of 'expert' within Delphi panel techniques. *Nurs Res* 2006;14:59-70. doi: 10.7748/nr2006.10.14.1.59.c6010.
69. National Institute for Health and Care Excellence. NICE Guideline [NG197]: Shared decision making. Available at: https://www.nice.org.uk/guidance/ng197.
70. Dunn AG, Coiera E, Mandl KD, et al. Conflict of interest disclosure in biomedical research: A review of current practices, biases, and the role of public registries in improving transparency. *Res Integr Peer Rev* 2016;1:1. doi: 10.1186/s41073-016-0006-7.

Figure Legends

Figure 1
Caption: PRISMA 2020 flow diagram for new systematic reviews which included searches of databases, registers and other sources

For peer review only

For peer review only - http://bmjopen.bmj.com/site/about/guidelines.xhtml

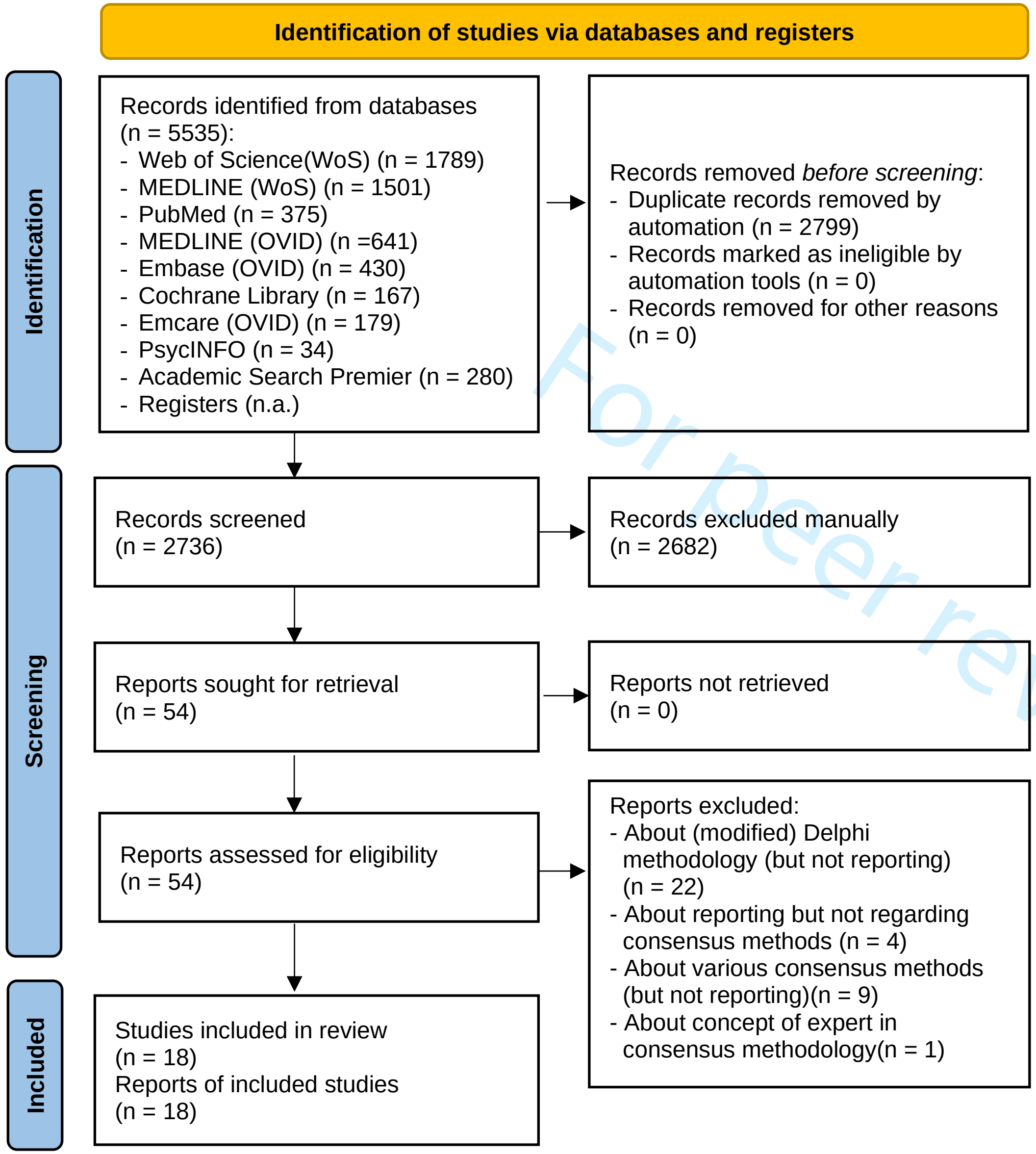

For peer review only

Gattrell *et al.*
Research Integrity and Peer Review (2022) 7:3
https://doi.org/10.1186/s41073-022-00122-0

Research Integrity and Peer Review

STUDY PROTOCOL **Open Access**

ACCORD guideline for reporting consensus-based methods in biomedical research and clinical practice: a study protocol

William T. Gattrell[1*], Amrit Pali Hungin[2], Amy Price[3], Christopher C. Winchester[4], David Tovey[5], Ellen L. Hughes[6], Esther J. van Zuuren[7], Keith Goldman[8], Patricia Logullo[9], Robert Matheis[10] and Niall Harrison[11]

Abstract

Background: Structured, systematic methods to formulate consensus recommendations, such as the Delphi process or nominal group technique, among others, provide the opportunity to harness the knowledge of experts to support clinical decision making in areas of uncertainty. They are widely used in biomedical research, in particular where disease characteristics or resource limitations mean that high-quality evidence generation is difficult. However, poor reporting of methods used to reach a consensus – for example, not clearly explaining the definition of consensus, or not stating how consensus group panellists were selected – can potentially undermine confidence in this type of research and hinder reproducibility. Our objective is therefore to systematically develop a reporting guideline to help the biomedical research and clinical practice community describe the methods or techniques used to reach consensus in a complete, transparent, and consistent manner.

Methods: The ACCORD (ACcurate COnsensus Reporting Document) project will take place in five stages and follow the EQUATOR Network guidance for the development of reporting guidelines. In Stage 1, a multidisciplinary Steering Committee has been established to lead and coordinate the guideline development process. In Stage 2, a systematic literature review will identify evidence on the quality of the reporting of consensus methodology, to obtain potential items for a reporting checklist. In Stage 3, Delphi methodology will be used to reach consensus regarding the checklist items, first among the Steering Committee, and then among a broader Delphi panel comprising participants with a range of expertise, including patient representatives. In Stage 4, the reporting guideline will be finalised in a consensus meeting, along with the production of an Explanation and Elaboration (E&E) document. In Stage 5, we plan to publish the reporting guideline and E&E document in open-access journals, supported by presentations at appropriate events. Dissemination of the reporting guideline, including a website linked to social media channels, is crucial for the document to be implemented in practice.

Discussion: The ACCORD reporting guideline will provide a set of minimum items that should be reported about methods used to achieve consensus, including approaches ranging from simple unstructured opinion gatherings to highly structured processes.

*Correspondence: wgattrell@yahoo.co.uk

[1] Global Medical Affairs, Ipsen, Abingdon, UK
Full list of author information is available at the end of the article

BMC

© The Author(s) 2022. **Open Access** This article is licensed under a Creative Commons Attribution 4.0 International License, which permits use, sharing, adaptation, distribution and reproduction in any medium or format, as long as you give appropriate credit to the original author(s) and the source, provide a link to the Creative Commons licence, and indicate if changes were made. The images or other third party material in this article are included in the article's Creative Commons licence, unless indicated otherwise in a credit line to the material. If material is not included in the article's Creative Commons licence and your intended use is not permitted by statutory regulation or exceeds the permitted use, you will need to obtain permission directly from the copyright holder. To view a copy of this licence, visit http://creativecommons.org/licenses/by/4.0/. The Creative Commons Public Domain Dedication waiver (http://creativecommons.org/publicdomain/zero/1.0/) applies to the data made available in this article, unless otherwise stated in a credit line to the data.

For peer review only - http://bmjopen.bmj.com/site/about/guidelines.xhtml

Keywords: Methodology, Guidelines, Reporting quality, Reporting completeness, Checklist, Delphi technique, Consensus, Nominal group technique, Consensus development conference

Background

Evidence-based medicine relies on three factors: current best evidence based on clinical and real-world studies, individual clinical expertise, and the desires of the patient [1]. Clinical data gathered from systematic reviews, high-quality randomised clinical trials, and observational studies have complementary roles in generating robust evidence [2, 3]. However, healthcare providers face difficult treatment decisions if the available information on a subject is inadequate, contradictory, limited, or does not exist.

The COVID-19 pandemic has brought this situation of lack of evidence into stark relief, as crucial decisions have to be made during any rapidly emerging public health crisis [4]. However, there are areas of medicine for which high-quality evidence generation can be difficult. This is due to disease characteristics such as rare occurrence and clinical heterogeneity among patients with the same condition, which can mean either that trials are difficult to interpret or that they may only be directly applicable to a subset of patients [5, 6]. A lack of resources and/or infrastructure can also be limiting [6, 7]. Moreover, even when evidence does exist, in medical situations with multiple considerations or confounding factors, there is the need to prioritise the use of available evidence to optimise outcomes [8].

Therefore, when no robust evidence is available, when divergent guidance exists, or when there is a need for collective judgement to increase reliability and validity, guidelines for clinical decision making or methodological or reporting approaches may be formulated based on expert consensus only [9–11]. Consensus methods provide opportunities to harness the knowledge of experts to support clinical decision making in areas of uncertainty [12]. As with all studies, appropriate methods and transparent reporting are key; however, the method used to reach consensus is not always clearly reported [11, 13].

Multiple methods are used to develop consensus-based publications. These range in methodological rigour from informal "expert consensus meetings" to structured or systematic approaches such as the Delphi method and the nominal group technique (NGT). Both Delphi and NGT are used for generating ideas or determining priorities, aiming to achieve general convergence, usually through voting on a series of multiple-choice questions [14–17]. In Delphi, and more recently electronic Delphi (eDelphi), individuals vote anonymously, while NGT is usually face-to-face [8, 18, 19]. The techniques and methodological steps used to reach consensus can vary (Table 1).

In group decisions, a wider range of knowledge may be drawn upon, the interaction between group members can stimulate and challenge received ideas, and idiosyncrasies may be filtered out through the group prioritisation process [19, 31–33]. The use of structured, systematic approaches to reach consensus is supported by the observation that, in an unstructured group meeting, there is the risk of a single individual dominating the discussion and decisions may be portrayed as unanimous when, in reality, there is dissent within the group [31]. Even within structured consensus meetings, depending on their roles, a few panel members can dominate the discussion [34]. Furthermore, individuals may be unwilling to retract long-held views in open discussion. For these reasons, structured approaches including a step where responses are anonymised are generally held to be superior to unstructured methods to achieve consensus [35, 36].

Developing consensus-based publications using robust methods is vital, but poor execution or reporting can render the techniques used for gathering opinion susceptible to criticism [37–40]. To take one of the most widely-used and most rigorous consensus methodologies, the Delphi method has been used extensively in a wide range of sectors including military, education, social science and healthcare since its conception in the 1950s at the RAND Corporation [41]. This is because it has the potential to mitigate many of the aforementioned pitfalls in group decisions, such as the risk of peer pressure in techniques such as the NGT [38, 42]. Due to its versatility, the Delphi method can be modified to meet individual study needs. However, the reporting of such "modified Delphi" methods may lack clarity on the details of the process involved or the rationale for the modification [38, 42].

Definitions of the thresholds for consensus (i.e., approval rates), for example, can vary or be poorly described in studies using consensus [43]. Other reporting or methodological problems identified are that analytical methods may not be predefined [37, 43], the recruitment process used to identify the experts may not be explicit [44], or the funding source not clearly disclosed [45]. In fact, critics suggest the term "Delphi research" be phased out in academic publications to force authors to more precisely describe the methodology used [46].

For peer review only - http://bmjopen.bmj.com/site/about/guidelines.xhtml

Table 1 Possible types of consensus methods and characteristics that can be mixed or used separately in different stages of studies to reach consensus

Method	Characteristics	Data analysis
Consensus conference or meeting [20–22]	Face-to-face meetings where a group of participants, usually experts in one field of knowledge, discuss one or more topics, prompted by facilitators, and have to either create ideas/statements or decide/vote on pre-set topics/statements. The discussion is frequently prompted by evidence from the literature — or the lack of it.	Qualitative or quantitative, or mixed.
Nominal group technique (NGT) [20, 22, 23]	As in conference meetings, in NGT, face-to-face meetings are held, but several sessions are organised with iterative stages. In the first step, suggestions are collected from the groups into questionnaires or lists of topics circulated again in the second step. In the second stage, participants need to vote or rate, usually using scales (like Likert scales). The group then discusses the aggregated summary of the voting or rating. The group is not anonymous and may include experts and non-experts. A facilitator makes sure every participant is given the opportunity to speak and vote.	Qualitative initially and then quantitative when responses are aggregated and summarised.
Delphi [12, 20, 22–30]	The three principles of the Delphi technique are: 1) anonymity during voting/selecting/rating (participants do not meet); 2) multiple rounds (at least 2) and 3) feedback to participants to inform them about each last voting/rating before they start the next round. Delphi was traditionally organised by postal mail in the past, and now electronic specialised survey platforms facilitate the process.	Quantitative for voting/rating, qualitative when extra comments/suggestions are allowed.
Other mixed methods [20, 22]	A consensus study can begin with simple focus groups to collect ideas, stories, experiences, and general opinions to start a more structured NGT or Delphi exercise. Frequently, two or more methods are used. For example, a Delphi activity can be used initially with the list of statements approved to be discussed in consensus conferences where final decisions are made, sometimes referred to as a "modified Delphi".	Qualitative methods are used when perceptions, stories, and experiences are collected. Several quantitative statistics can be used to summarise voting and ratings.

The lack of appropriate and transparent description in publications of the consensus methods used suggests that a reporting guideline is needed. A reporting guideline comprises "a checklist, flow diagram, or explicit text to guide authors in reporting a specific type of research, developed using explicit methodology" [11]. Consensus methods themselves play an important role in the development of reporting guidelines in various fields of health. As part of an ongoing audit of the EQUATOR database [47], it has been observed that, of the 226 reporting guidelines added between database inception and October 2018, only one third (77/226) explicitly mentioned the use of Delphi methodology (Fig. 1), while in another third (75/226), the information was not reported. A systematic review of the EQUATOR database indicated a similar result and added that among the reporting guidelines that mentioned the Delphi method, the description of details of the participants, number of rounds, criteria for dropping items or stopping the rounds was not always reproducible [48].

A range of methods can be used to reach consensus for clinical guidance, nomenclature, and other approaches in healthcare and public health [49]. However, to the best of our knowledge, the only reporting guidance in healthcare using consensus research is the CREDES (guidance on Conducting and REporting DElphi Studies) Statement, which provides valuable recommendations for the reporting of Delphi consensus in palliative care [38]. Nevertheless, CREDES is specific to palliative care and is limited to the Delphi method [38], which leaves a gap for a reporting guideline that can be applied to other biomedical areas and consensus processes involving non-Delphi based methods or "modified Delphi" — an issue that CREDES acknowledges. Moreover, CREDES does not provide a detailed checklist to guide the incorporation of essential steps to be reported.

For peer review only - http://bmjopen.bmj.com/site/about/guidelines.xhtml

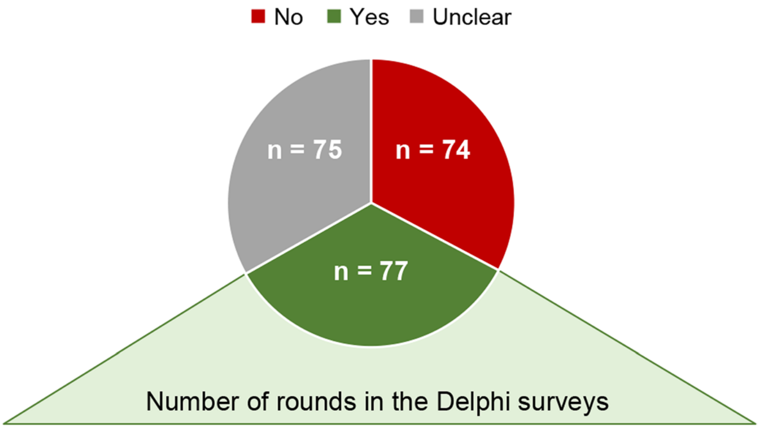

Rounds	n	%
1	8	3.54
2	32	14.16
3	24	10.62
4	6	2.65
5	1	0.44
Unclear	6	2.65
N/A	149	65.94

Fig. 1 Methodology declared by authors in developing a reporting guideline added to the EQUATOR database from inception to October 2018 (N = 226)

Detail-oriented reporting can help readers of publications to understand the key elements of the process – the methodology used, the participants involved, and how the study was conducted including the criteria for statement approval. Our objective is therefore to systematically develop a reporting guideline to help the biomedical research and clinical practice community describe the methods used to reach consensus in a complete, transparent, and consistent manner. Our aim is that the reporting guideline is appropriate to describe all types of consensus methodology. The reporting guideline for consensus-based biomedical publications will include a general statement with a checklist and an explanation and elaboration (E&E) document, including examples of good reporting. It will be identified under the acronym ACCORD (ACcurate COnsensus Reporting Document).

Methods/design

We have adopted the general method proposed by the EQUATOR Network for developing reporting guidelines [11]. The process for ACCORD development is outlined in Fig. 2.

Stage 1: establishment of a Steering Committee

With the endorsement of the International Society of Medical Publication Professionals (ISMPP), we assembled a Steering Committee to develop a reporting guideline for research using consensus. The Steering Committee (the authors, AH, AP, CW, DT, EH, EvZ, KG, NH, PL, RM, and WG) will lead and co-ordinate the guideline development process. Specifically, the Steering Committee will be responsible for: establishing the goals and timelines for the work, including registering and publishing the protocol; generating the initial list of checklist items from the literature review; conducting a consensus process to enrich and refine the initial list of minimum items that should be reported; implementing each stage of the process including developing questionnaires and analysing voting outcomes and other data; reporting the findings of the process in a statement document with the main checklist and guidance; developing an E&E document where all the items are individually explained and examples of approach and reporting are given; disseminating the reporting guidelines via publication, presentation at congresses and other events, and online presence including a website linked to social media channels.

The Steering Committee is a multidisciplinary group (11 people) that includes clinician practitioners, methodologists, publication professionals, patients, journal editors and publishers and the pharmaceutical industry. Prior to initiating Stage 2, we listed the project in the EQUATOR Network registry for reporting guidelines under development [50] and registered the protocol with the Open Science Framework [51].

For peer review only - http://bmjopen.bmj.com/site/about/guidelines.xhtml

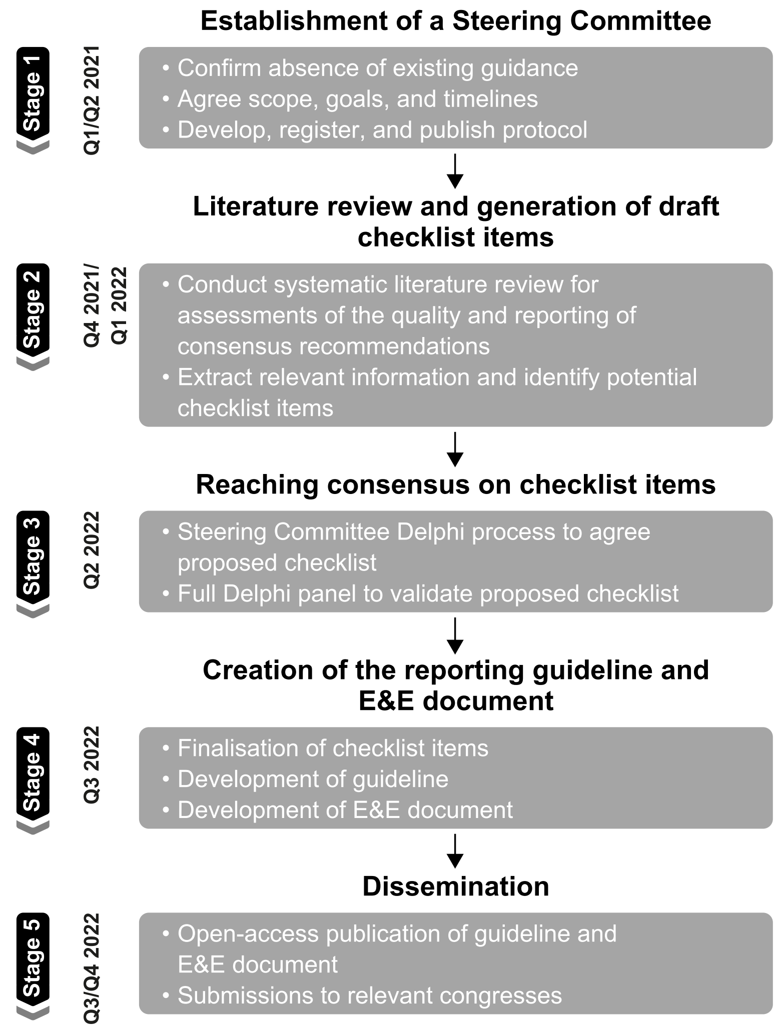

Fig. 2 Project overview for creating ACCORD, a reporting guideline for studies developed using consensus methods

Stage 2: literature review and generation of draft checklist items

The aim of this step is to seek evidence on the quality of reporting of the process undertaken in health studies using consensus methodology. This research will provide insight into possible checklist items for evaluation by the Delphi Panel (further information on the Delphi Panel is provided in 'Stage 3' below). The CREDES guidelines, specific to palliative care, will also be reviewed for elements that can be generalised to other biomedical fields [38].

Search strategy

The process for conducting the systematic review will be informed by and reported according to the Preferred Reporting Items for Systematic reviews and Meta-Analyses (PRISMA) 2020 and PRISMA-Search extension guidelines [11, 52]. Eligible studies will include studies, reviews and published guidance addressing the quality of reporting of consensus methodology that aim to improve health outcomes in biomedicine or clinical practice. Reports of studies using consensus methods but not commenting on their reporting quality will be excluded, for example, studies to reach clinical recommendations of core outcome sets or reporting guidelines using consensus methods. Ineligible publications include editorials, letters about individual publications, and comments on methodology of consensus outside the scope of biomedical research.

Searches of EMBASE (OVID), MEDLINE (OVID), Web of Science - Core Collection, MEDLINE (Web of Science), PubMed, Cochrane Library, and Emcare (OVID), Academic Search Premier and PsycINFO databases will be run with no limits by year or language of publication at the search stage. Four initial search strategies were developed and sequentially

For peer review only - http://bmjopen.bmj.com/site/about/guidelines.xhtml

piloted by members of the Steering Committee (WG, EvZ and PL) with the assistance of an information (JS) and systematic review specialist (ZF). The piloting allowed the adjustment of the initial search strategy by the information specialist to provide results that better aligned with the inclusion criteria and objective of this study. The refined, broad search strategy (Supplementary File) will be used to identify and generate the final list of studies focusing on the quality and accuracy of reporting of Delphi and other consensus processes, methods, techniques or recommendations. The search may also be augmented with relevant articles highlighted by the Steering Committee as appropriate based on the individuals' prior work and expertise in the area (via a manual search).

Data extraction

EvZ, PL, WG, and ZF will independently screen the titles and abstracts retrieved from the search for potential inclusion using the Rayyan tool in blind mode [53]. Any discrepancies will be resolved by discussion. Full-text articles will then be retrieved and assessed independently for eligibility, with reconciliation of any differences through discussion. Data will be extracted using a draft extraction form, which will be piloted on three studies before use. Based on the information gathered on the literature review, a list of preliminary items for the checklist will be generated to be refined in a Delphi exercise in Stage 3.

Stage 3: reaching consensus on checklist items

We will use Delphi methodology, as described below, to reach a consensus regarding the checklist items to include in the reporting guideline. This will take place in two steps, with the first involving the Steering Committee and the second involving a full Delphi Panel (the ACCORD Delphi Panel; Fig. 3). We plan to report the consensus methodology in accordance with our own guidelines under development.

First step: steering committee survey

The Steering Committee will review the data extracted from literature search. This initial list is likely to contain duplicated items or items that require rewording. The aim is to eliminate repetitions and inadequately or ambiguously written items to reach a list of unique items. Using a survey, the Steering Committee members involved in the literature review will independently suggest items for the initial checklist; NH and WG will consolidate the initial checklist items.

There will then be anonymous voting to confirm the initial checklist that will be put to the full ACCORD consensus panel. Steering Committee members (excluding NH and WG) will vote (anonymised and blinded) on whether they 'Strongly Agree', 'Agree', 'Disagree', 'Strongly Disagree', or feel 'Abstained/Unable to answer' for all proposed items. There will also be the opportunity to provide comments. Any items that do not receive support will be discussed by the Steering Committee, and either included as 'possible additional items' or discarded completely. The eliminated items and the reasons for their

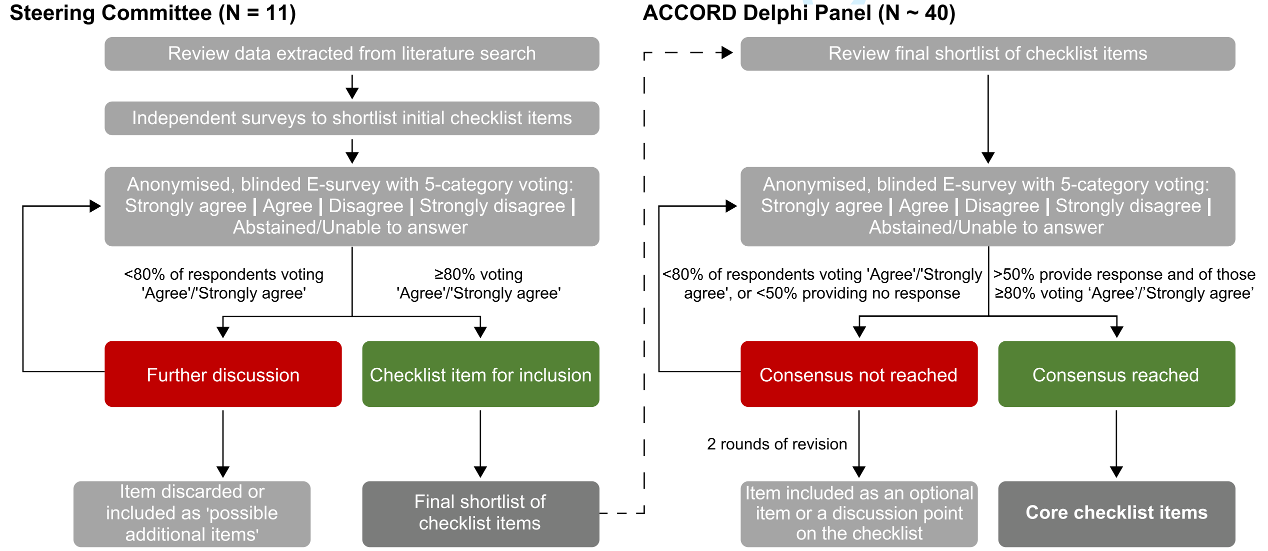

Fig. 3 Methodology used by the ACCORD Steering Committee and ACCORD Delphi Panel to achieve consensus on core checklist items for a consensus reporting guideline

elimination will be reported. The candidate items will be presented in sequence as a draft checklist, and in the same order to all people voting, so that the overall checklist structure, considering the manuscript sections (such as Introduction, Methods, Results, Discussion) can be evaluated. Within each section, there will be 'proposed items' and 'possible additional items'.

Second step: ACCORD Delphi panel

The preliminary list of checklist items agreed on by the Steering Committee will subsequently be put to the ACCORD Delphi Panel for validation using a blinded electronic voting platform (e-survey). In addition, the ACCORD Delphi Panel will be provided with the list of items excluded by the Steering Committee for information, as a confirmatory step.

The order of the candidate items within each manuscript section will be randomised so that it is different for each person voting and all items are evaluated fully independently from each other. Five voting options will be offered: 'Strongly Agree', 'Agree', 'Disagree', 'Strongly Disagree', and 'Abstained/Unable to answer'. Votes of 'Abstained/Unable to answer' will be included in the denominator. Panellists will be able to provide free-text comments and will have the opportunity to propose additional items. There will be three rounds of voting; with feedback and descriptive statistics incorporated for the next round by NH and WG. The approval rate and the reasons for elimination of items will be reported.

The consensus threshold is defined in this step as at least 20 respondents (approximately 50% of the target panel size), and at least 80% of responding ACCORD Delphi panellists who are able to answer voting 'Agree' or 'Strongly Agree', with two rounds of statement revision and re-voting. The Steering Committee will review items that do not achieve consensus in rounds 1 or 2 and these will be revised or eliminated taking into account any free-text comments. If consensus is not achieved by the ACCORD Delphi Panel, or there are insufficient respondents, the Steering Committee may decide that the item will be included as an optional item or a discussion point on the E&E document or checklist, alongside core items on which consensus was achieved. Simple descriptive statistics (response rates, level of agreement for each statement, median levels of agreement and interquartile ranges) will be used to describe approval rates between rounds. The same measures will be used to evaluate consensus stability across rounds [54].

There are no generally agreed standards for the panel size for Delphi studies, and a wide range of panel sizes has been reported; panels of 20–30 participants are common [55, 56]. However, it is recognised that the size and diversity of a Delphi panel can impact the quality of the final recommendations [57]. The ACCORD Delphi Panel will comprise approximately 40 members, so that it allows for representation from clinicians, methodologists, patient advocates, lay public representatives, health technologists, journal editors and publishers, regulatory specialists, and publications professionals, and to ensure an acceptable number of responses (20, or at least 50% of the group) in the event of drop-outs or partial completion of review. The ACCORD project will be advertised to potential Delphi Panellists via relevant societies, organisations, and networks; in addition, authors of recently published consensus studies in high-profile journals will be invited directly.

When registering, panellists will be asked to complete a preliminary survey to capture basic information on experience, geographical, and demographic representation. Although no formal targets will be established, the Steering Committee will endeavour to ensure a broad spread of representation across these categories. Members of the Delphi Panel will be recognised as contributors in the acknowledgements section of the guideline (with their permission) but participation in ACCORD Delphi panel will not qualify a panellist for authorship.

Software or a voting platform that is appropriate for Delphi exercises will be used to implement the voting process, administered by NH and WG. Alternatives available on the market are being evaluated and tested at the time of this protocol publication, and the platform and version used will be reported. Initial requirements are that the software used follows security regulations, ethical standards and allows, besides voting, the inclusion of free text responses in the e-surveys to supplement discussion in the E&E document.

Stage 4: creation of the reporting guideline and E&E document

On completion of the Delphi consensus process, the checklist will be finalised by WG and NH for approval by the Steering Committee, and the reporting guideline will be developed. A separate E&E document will be created to provide a detailed rationale for the items included in the checklist. In each case, an example will be included of good reporting from a published paper. The E&E document can also be informed by perspectives collected from researchers involved in consensus-based studies outside the biomedical field.

Stage 5: dissemination

We intend to publish the reporting guideline and E&E document in open access format via a CC-BY copyright licence. Future publications from the ACCORD project will be reported according to the best available reporting guidelines for each type of manuscript.

For peer review only - http://bmjopen.bmj.com/site/about/guidelines.xhtml

To aid dissemination, we plan to present the findings at congresses including ISMPP European and Annual Meetings, the World Conference on Research Integrity and Peer Review, and the UK Research Integrity Office Annual Conference. Progress will be updated on a dedicated website for the ACCORD project, the EQUATOR website and newsletter, and social media channels, and communicated in appropriate professional forums and events. This dissemination of the reporting guideline is crucial for the document to be implemented in practice.

Discussion

The ACCORD reporting guideline will provide a set of minimum items that should be reported about methods used to achieve consensus in biomedical research and guidance, including processes ranging from simple unstructured opinion gatherings to highly structured processes. The objective is to systematically develop a reporting guideline to help the biomedical research and clinical practice community describe the methods or techniques used to reach consensus in a complete, transparent, and consistent manner.

Extensions of the ACCORD reporting guideline and checklist could potentially be developed in the future to cover consensus studies in the non-biomedical sectors, with appropriate input from experts in those sectors to account for characteristics specific to each field. Our objective is to increase the completeness, transparency and consistency of the reporting of consensus methodology and, as a result, to improve the trustworthiness of recommendations developed using consensus methods. The Steering Committee welcomes enquiries from individuals interested in participating in the ACCORD Delphi Panel.

Supplementary Information

The online version contains supplementary material available at https://doi.org/10.1186/s41073-022-00122-0.

Additional file 1.

Acknowledgements
We thank Bernd Arents, who joined the Steering Committee and provided valuable input into the project but then had to step down because of other commitments. Medical writing support was provided by Sreerekha Pillai and Luke Worley of Ogilvy Health. Wendy Hazard of ISMPP provided administrative support. Jan Schoones (Leiden University Medical Center) assisted in development of the search strategy and Zbys Fedorowicz provided support for screening of hits from the systematic searches.

Authors' contributions
NH and WG recruited the ACCORD Steering Committee and coordinated the development, drafting, and review of this protocol. All authors contributed to the development of the protocol, reviewed, commented and approved the draft manuscript. The authors, except for the NH and WG, are listed in alphabetical order.

Funding
Ipsen provided access to full-text articles that were not otherwise freely available to support the development of this protocol and provided the open access processing charge. Ogilvy Health provided medical writing support.

Availability of data and materials
Anonymised aggregated data will be deposited in the Open Science Framework, where the study protocol has already been registered (https://osf.io/2rzm9). Individual responses to Delphi rounds will be deidentified at the source level by the platform used. These individual responses and approval rates can be requested to the corresponding author. The ACCORD protocol has been listed on the EQUATOR website and pre-registered with the Open Science Framework.

Declarations

Ethics approval and consent to participate
Not applicable.

Consent for publication
Not applicable.

Competing interests
PL is a member of the UK EQUATOR Centre, an organisation that promotes the use of reporting guidelines, many of which are developed using consensus methods, and she is personally involved in the development of other reporting guidelines. WG is a former employee of Ipsen and is now employed by Bristol Myers Squib. KG is an employee of AbbVie. APH, in the last five years, worked with Reckitt Benckiser for the development of the definitions and management of gastro-oesophageal reflux disease. CCW is an employee, Director, and shareholder of Oxford PharmaGenesis Ltd., a Director of Oxford Health Policy Forum CIC, a Trustee of the Friends of the National Library of Medicine, and an Associate Fellow of Green Templeton College. NH is an employee of Ogilvy Health UK. EH has worked with Ogilvy Health UK on consensus projects. AP, DT, RM and EJvZ have no conflict of interest.

Author details
[1]Global Medical Affairs, Ipsen, Abingdon, UK. [2]Faculty of Medical Sciences, Newcastle University, Newcastle, UK. [3]Stanford Anesthesia, Informatics and Media Lab, Stanford University School of Medicine, Stanford, CA, USA. [4]Oxford PharmaGenesis, Tubney, Oxford, UK. [5]Journal of Clinical Epidemiology, Sussex, UK. [6]Sciwright Limited, Somerset, UK. [7]Leiden University Medical Centre, Leiden, The Netherlands. [8]Global Medical Affairs, AbbVie, North Chicago, IL, USA. [9]Centre for Statistics in Medicine (CSM), University of Oxford, and EQUATOR Network UK Centre, Oxford, UK. [10]International Society for Medical Publication Professionals, New York, NY, USA. [11]Ogilvy Health, London, UK.

Received: 15 October 2021 Accepted: 9 May 2022
Published online: 07 June 2022

References
1. Szajewska H. Evidence-based medicine and clinical research: both are needed, Neither Is Perfect. Ann Nutr Metab. 2018;72(Suppl 3):13–23. https://doi.org/10.1159/000487375.
2. Barton S. Which clinical studies provide the best evidence? The best RCT still trumps the best observational study. BMJ. 2000;321(7256):255–6. https://doi.org/10.1136/bmj.321.7256.255.
3. Gopalakrishnan S, Ganeshkumar P. Systematic reviews and Meta-analysis: understanding the best evidence in primary healthcare. J Family Med Prim Care. 2013;2(1):9–14. https://doi.org/10.4103/2249-4863.109934.
4. Greenhalgh T. Will COVID-19 be evidence-based medicine's nemesis? PLoS Med. 2020;17(6):e1003266. https://doi.org/10.1371/journal.pmed.1003266.
5. Kempf L, Goldsmith JC, Temple R. Challenges of developing and conducting clinical trials in rare disorders. Am J Med Genet A. 2018;176(4):773–83. https://doi.org/10.1002/ajmg.a.38413.

For peer review only

6. Frieden TR. Evidence for health decision making - beyond randomized, controlled trials. N Engl J Med. 2017;377(5):465–75. https://doi.org/10.1056/NEJMra1614394.
7. Haw WY, Al-Janabi A, Arents BWM, Asfour L, Exton LS, Grindlay D, et al. Global guidelines in dermatology mapping project (GUIDEMAP): a scoping review of dermatology clinical practice guidelines. Br J Dermatol. 2021;185(4):467–75. https://doi.org/10.1111/bjd.20428.
8. McMillan SS, King M, Tully MP. How to use the nominal group and Delphi techniques. Int J Clin Pharm. 2016;38(3):655–62. https://doi.org/10.1007/s11096-016-0257-x.
9. Raine R, Sanderson C, Black N. Developing clinical guidelines: a challenge to current methods. BMJ. 2005;331(7517):631–3. https://doi.org/10.1136/bmj.331.7517.631.
10. Djulbegovic B, Guyatt G. Evidence vs consensus in clinical practice guidelines. JAMA. 2019;322(8):725–6. https://doi.org/10.1001/jama.2019.9751.
11. Moher D, Schulz KF, Simera I, Altman DG. Guidance for developers of health research reporting guidelines. PLoS Med. 2010;7(2):e1000217. https://doi.org/10.1371/journal.pmed.1000217.
12. Fink-Hafner D, Dagen T, Dousak M, Novak M, Hafner-Fink M. Delphi method: strengths and weaknesses. Metodoloski Zvezki. 2019;16(2):1–19 http://www.dlib.si/?URN=URN:NBN:SI:DOC-4UR1RO95.
13. Arakawa N, Bader LR. Consensus development methods: considerations for national and global frameworks and policy development. Res Soc Adm Pharm. 2021;18(1):2222–9. https://doi.org/10.1016/j.sapharm.2021.06.024.
14. Dalkey N, Helmer O. An experimental application of the DELPHI method to the use of experts. Manag Sci. 1963;9(3):458–67. https://doi.org/10.1287/mnsc.9.3.458.
15. Murphy MK, Black NA, Lamping DL, McKee CM, Sanderson CFB, Askham J, et al. Consensus development methods, and their use in clinical guideline development. Health Technol Assess. 1998;2(3):1–88. https://doi.org/10.3310/hta2030.
16. Foth T, Efstathiou N, Vanderspank-Wright B, Ufholz LA, Dutthorn N, Zimansky M, et al. The use of Delphi and nominal group technique in nursing education: a review. Int J Nurs Stud. 2016;60:112–20. https://doi.org/10.1016/j.ijnurstu.2016.04.015.
17. Nair R, Aggarwal R, Khanna D. Methods of formal consensus in classification/diagnostic criteria and guideline development. Semin Arthritis Rheum. 2011;41(2):95–105. https://doi.org/10.1016/j.semarthrit.2010.12.001.
18. Grant S, Armstrong C, Khodyakov D. Online modified-Delphi: a potential method for continuous patient engagement across stages of clinical practice guideline development. J Gen Intern Med. 2021;36(6):1746–50. https://doi.org/10.1007/s11606-020-06514-6.
19. Helmer-Hirshberg O. Analysis of the future: the Delphi method. Santa Monica: The RAND Corporation; 1967. https://www.rand.org/pubs/papers/P3558.html
20. Fink A, Kosecoff J, Chassin M, Brook RH. Consensus methods: characteristics and guidelines for use. Am J Public Health. 1984;74(9):979–83. https://doi.org/10.2105/ajph.74.9.979.
21. Halcomb E, Davidson P, Hardaker L. Using the consensus development conference method in healthcare research. Nurs Res. 2008;16(1):56–71. https://doi.org/10.7748/nr2008.10.16.1.56.c6753.
22. James D, Warren-Forward H. Research methods for formal consensus development. Nurs Res. 2015;22(3):35–40. https://doi.org/10.7748/nr.22.3.35.e1297.
23. Campbell SM, Cantrill JA. Consensus methods in prescribing research. J Clin Pharm Ther. 2001;26(1):5–14. https://doi.org/10.1046/j.1365-2710.2001.00331.x.
24. Drumm S, Bradley C, Moriarty F. 'More of an art than a science'? The development, design and mechanics of the Delphi technique. Res Soc Adm Pharm. 2022;18(1):2230–6. https://doi.org/10.1016/j.sapharm.2021.06.027.
25. Durkin J, Usher K, Jackson D. Using consensus from experts to inform a shared understanding of subjective terms. Nurs Res. 2019;27(2):46–9. https://doi.org/10.7748/nr.2019.e1622.
26. Franklin KK, Hart JK. Idea generation and exploration: benefits and limitations of the policy Delphi research method. Innov High Educ. 2007;31:237–46. https://doi.org/10.1007/s10755-006-9022-8
27. Guzys D, Dickson-Swift V, Kenny A, Threlkeld G. Gadamerian philosophical hermeneutics as a useful methodological framework for the Delphi technique. Int J Qual Stud Health Well-being. 2015;10:26291. https://doi.org/10.3402/qhw.v10.26291.
28. Keeney S, Hasson F, McKenna H. Consulting the oracle: ten lessons from using the Delphi technique in nursing research. J Adv Nurs. 2006;53(2):205–12. https://doi.org/10.1111/j.1365-2648.2006.03716.x.
29. Khodyakov D, Grant S, Denger B, Kinnett K, Martin A, Peay H, et al. Practical considerations in using online modified-Delphi approaches to engage patients and other stakeholders in clinical practice guideline development. Patient. 2020;13(1):11–21. https://doi.org/10.1007/s40271-019-00389-4.
30. Hallowell MR. Qualitative research: application of the Delphi method to CEM research. J Constr Eng Manag. 2010;136(1):99–107. https://doi.org/10.1061/(ASCE)CO.1943-7862.0000137.
31. Hsu CC, Sandford BA. The Delphi technique: making sense of consensus. Pract Assess Res Eval. 2007;12(10):1–8. https://doi.org/10.7275/pdz9-th90.
32. Hsieh CJ, Fific M, Yang CT. A new measure of group decision-making efficiency. Cogn Res Princ Implic. 2020;5(1):45. https://doi.org/10.1186/s41235-020-00244-3.
33. Beiderbeck D, Frevel N, von der Gracht HA, Schmidt SL, Schweitzer VM. Preparing, conducting, and analyzing Delphi surveys: cross-disciplinary practices, new directions, and advancements. MethodsX. 2021;8:101401. https://doi.org/10.1016/j.mex.2021.101401.
34. Li S-A, Yousefi-Nooraie R, Guyatt G, Talwar G, Wang Q, Zhu Y, et al. A few panel members dominated guideline development meeting discussions: a social network analysis. J Clin Epidemiol. 2022;141:1–10. https://doi.org/10.1016/j.jclinepi.2021.09.023.
35. Hohmann E, Brand JC, Rossi MJ, Lubowitz JH. Expert opinion is necessary: Delphi panel methodology facilitates a scientific approach to consensus. Arthroscopy. 2018;34(2):349–51. https://doi.org/10.1016/j.arthro.2017.11.022.
36. Humphrey-Murto S, Varpio L, Gonsalves C, Wood TJ. Using consensus group methods such as Delphi and nominal group in medical education research. Med Teach. 2017;39(1):14–9. https://doi.org/10.1080/0142159X.2017.1245856.
37. Grant S, Booth M, Khodyakov D. Lack of preregistered analysis plans allows unacceptable data mining for and selective reporting of consensus in Delphi studies. J Clin Epidemiol. 2018;99:96–105. https://doi.org/10.1016/j.jclinepi.2018.03.007.
38. Jünger S, Payne SA, Brine J, Radbruch L, Brearley SG. Guidance on conducting and REporting DElphi studies (CREDES) in palliative care: recommendations based on a methodological systematic review. Palliat Med. 2017;31(8):684–706. https://doi.org/10.1177/0269216317690685.
39. Hohmann E, Cote MP, Brand JC. Research pearls: expert consensus based evidence using the Delphi method. Arthroscopy. 2018;34(12):3278–82. https://doi.org/10.1016/j.arthro.2018.10.004.
40. Humphrey-Murto S, Varpio L, Wood TJ, Gonsalves C, Ufholz LA, Mascioli K, et al. The use of the Delphi and other consensus group methods in medical education research: a review. Acad Med. 2017;92(10):1491–8. https://doi.org/10.1097/ACM.0000000000001812.
41. Gupta UG, Clarke RE. Theory and applications of the Delphi technique: a bibliography (1975–1994). Technol Forecast Soc Change. 1996;53(2):185–211. https://doi.org/10.1016/S0040-1625(96)00094-7.
42. Veugelers R, Gaakeer MI, Patka P, Huijsman R. Improving design choices in Delphi studies in medicine: the case of an exemplary physician multi-round panel study with 100% response. BMC Med Res Methodol. 2020;20(1):156. https://doi.org/10.1186/s12874-020-01029-4.
43. Diamond IR, Grant RC, Feldman BM, Pencharz PB, Ling SC, Moore AM, et al. Defining consensus: a systematic review recommends methodologic criteria for reporting of Delphi studies. J Clin Epidemiol. 2014;67(4):401–9. https://doi.org/10.1016/j.jclinepi.2013.12.002.
44. Paré G, Cameron A-F, Poba-Nzaou P, Templier M. A systematic assessment of rigor in information systems ranking-type Delphi studies. Inf Manag. 2013;50(5):207–17. https://doi.org/10.1016/j.im.2013.03.003.
45. Gattrell WT, Clements SJ, Sheard D. Quality assessment of guidelines/recommendations developed using Delphi methodology. Curr Med Res Opin. 2019;35(Suppl 2):40. https://doi.org/10.1080/03007995.2019.1583496.
46. Thompson M. Considering the implication of variations within Delphi research. Fam Pract. 2009;26(5):420–4. https://doi.org/10.1093/fampra/cmp051.

For peer review only

47. Schlussel M, Collins G, de Beyer JA, Kirtley S. EQUATOR Library Audit. Available from: https://osf.io/fuv3c. Accessed 21 Jan 2022.
48. Banno M, Tsujimoto Y, Kataoka Y. The majority of reporting guidelines are not developed with the Delphi method: a systematic review of reporting guidelines. J Clin Epidemiol. 2020;124:50–7. https://doi.org/10.1016/j.jclinepi.2020.04.010.
49. Brouwers MC, Kho ME, Browman GP, Burgers JS, Cluzeau F, Feder G, et al. AGREE II: advancing guideline development, reporting and evaluation in health care. CMAJ. 2010;182(18):E839–42. https://doi.org/10.1503/cmaj.090449.
50. EQUATOR Network. Available from: equator-network.org. Accessed 17 Sept 2021.
51. Open Science Framework Registries. Available from: https://osf.io/2rzm9. Accessed 07 Mar 2022.
52. Rethlefsen ML, Kirtley S, Waffenschmidt S, Ayala AP, Moher D, Page MJ, et al. PRISMA-S: an extension to the PRISMA statement for reporting literature searches in systematic reviews. Syst Rev. 2021;10(1):39. https://doi.org/10.1186/s13643-020-01542-z.
53. Ouzzani M, Hammady H, Fedorowicz Z, Elmagarmid A. Rayyan—a web and mobile app for systematic reviews. Syst Rev. 2016;5(1):210. https://doi.org/10.1186/s13643-016-0384-4.
54. Holey EA, Feeley JL, Dixon J, Whittaker VJ. An exploration of the use of simple statistics to measure consensus and stability in Delphi studies. BMC Med Res Methodol. 2007;7(1):52. https://doi.org/10.1186/1471-2288-7-52.
55. Akins RB, Tolson H, Cole BR. Stability of response characteristics of a Delphi panel: application of bootstrap data expansion. BMC Med Res Methodol. 2005;5:37. https://doi.org/10.1186/1471-2288-5-37.
56. Chalmers J, Armour M. The Delphi technique. In handbook of research methods in health social sciences. Singapore: Springer; 2019. p. 715–35. https://doi.org/10.1007/978-981-10-5251-4_99.
57. Birko S, Dove ES, Ozdemir V. Evaluation of nine consensus indices in Delphi foresight research and their dependency on Delphi survey characteristics: a simulation study and debate on Delphi design and interpretation. PLoS One. 2015;10(8):e0135162. https://doi.org/10.1371/journal.pone.0135162.

Publisher's Note

Springer Nature remains neutral with regard to jurisdictional claims in published maps and institutional affiliations.

Ready to submit your research? Choose BMC and benefit from:

- fast, convenient online submission
- thorough peer review by experienced researchers in your field
- rapid publication on acceptance
- support for research data, including large and complex data types
- gold Open Access which fosters wider collaboration and increased citations
- maximum visibility for your research: over 100M website views per year

At BMC, research is always in progress.

Learn more biomedcentral.com/submissions

For peer review only

ACCORD - January 7th, 2022

Regular references:

Total: 2599 references, sourced from:

- Web of Science - core collection: 1775
- MEDLINE (Web of Science): 1501 - 202 unique
- PubMed: 375 - 219 unique
- MEDLINE (OVID): 641 - 174 unique
- Embase (OVID): 331 - 66 unique
- Cochrane Library: 131 - 77 unique
- Emcare (OVID): 179 - 29 unique
- Academic Search Premier: 280 - 23 unique
- PsycINFO: 173 - 34 unique

Meeting abstract references:

Total: 137 references, sourced from:

- Web of Science: 14
- Embase (OVID): 99 - 90 unique
- Cochrane Library: 36 - 33 unique

Known references:

- PubMed: 27841062 26796090 25587865 26395179 24581294
- MEDLINE (Web of Science): PMID=(27841062 OR 26796090 OR 25587865 OR 26395179 OR 24581294)
- Web of Science Core Collection: UT=(000393885800003 OR 000375153500022 OR 000376181900007 OR 000361506400001 OR 000334256400007 OR 000309802600012 OR 000321232400002 OR 000309802600012 OR 000465105500070)

Databases:

Web of Science Core Collection and MEDLINE (Web of Science)
http://isiknowledge.com/wos

((TI=("Delphi Technique" OR "Delphi Technique" OR "Delphi techniques" OR "Delphi method" OR "Delphi methods" OR "Delphi study" OR "Delphi studies" OR "Delphi survey" OR "Delphi surveys" OR "Delphi consensus" OR "Delphi based consensus" OR "Delphi questionnaire" OR "Delphi questionnaires" OR "Delphi research" OR "Delphi review" OR "Delphi reviews" OR "Delphi process" OR "Delphi processes" OR "Delphi based" OR "Delphi procedure" OR "Delphi procedures" OR "Delphi assessment" OR "Delphi assessments" OR "Delphi approach" OR "Delphi approaches" OR "Delphi panel" OR "Delphi panels" OR "Delphi round" OR "Delphi rounds" OR "Delphi analysis" OR

"Delphi expert" OR "Delphi experts" OR "Delphi consultation" OR "Delphi methodology" OR "nominal group technique" OR "nominal group techniques" OR "nominal group" OR "nominal groups" OR "nominal grouping" OR "consensus recommendation" OR "consensus recommendations" OR "consensus development" OR "consensus activity" OR "consensus activities" OR "Consensus Development Conference" OR "Consensus Development" OR "Consensus methodology" OR "consensus method*" OR "RAND" OR (("Guidelines" OR "guideline") NEAR/2 ("consensus" OR "delphi"))) OR AB=("Delphi Technique" OR "Delphi Technique" OR "Delphi techniques" OR "Delphi method" OR "Delphi methods" OR "Delphi study" OR "Delphi studies" OR "Delphi survey" OR "Delphi surveys" OR "Delphi consensus" OR "Delphi based consensus" OR "Delphi questionnaire" OR "Delphi questionnaires" OR "Delphi research" OR "Delphi review" OR "Delphi reviews" OR "Delphi process" OR "Delphi processes" OR "Delphi based" OR "Delphi procedure" OR "Delphi procedures" OR "Delphi assessment" OR "Delphi assessments" OR "Delphi approach" OR "Delphi approaches" OR "Delphi panel" OR "Delphi panels" OR "Delphi round" OR "Delphi rounds" OR "Delphi analysis" OR "Delphi expert" OR "Delphi experts" OR "Delphi consultation" OR "Delphi methodology" OR "nominal group technique" OR "nominal group techniques" OR "nominal group" OR "nominal groups" OR "nominal grouping" OR "consensus recommendation" OR "consensus recommendations" OR "consensus development" OR "consensus activity" OR "consensus activities" OR "Consensus Development Conference" OR "Consensus Development" OR "Consensus methodology" OR "consensus method*" OR "RAND") OR AK=("Delphi Technique" OR "Delphi Technique" OR "Delphi techniques" OR "Delphi method" OR "Delphi methods" OR "Delphi study" OR "Delphi studies" OR "Delphi survey" OR "Delphi surveys" OR "Delphi consensus" OR "Delphi based consensus" OR "Delphi questionnaire" OR "Delphi questionnaires" OR "Delphi research" OR "Delphi review" OR "Delphi reviews" OR "Delphi process" OR "Delphi processes" OR "Delphi based" OR "Delphi procedure" OR "Delphi procedures" OR "Delphi assessment" OR "Delphi assessments" OR "Delphi approach" OR "Delphi approaches" OR "Delphi panel" OR "Delphi panels" OR "Delphi round" OR "Delphi rounds" OR "Delphi analysis" OR "Delphi expert" OR "Delphi experts" OR "Delphi consultation" OR "Delphi methodology" OR "nominal group technique" OR "nominal group techniques" OR "nominal group" OR "nominal groups" OR "nominal grouping" OR "consensus recommendation" OR "consensus recommendations" OR "consensus development" OR "consensus activity" OR "consensus activities" OR "Consensus Development Conference" OR "Consensus Development" OR "Consensus methodology" OR "consensus method*" OR "RAND" OR (("Guidelines" OR "guideline") NEAR/2 ("consensus" OR "delphi")))) AND (TI=("quality of reporting" OR "reporting quality" OR "reporting qualities" OR "selective reporting" OR "poor reporting" OR "poor reported" OR "reporting guideline" OR "reporting" OR ("reporting" AND ("quality" OR "selective" OR "poor" OR "weak" OR "manner" OR "rigor" OR "improv*")) OR "Data Accuracy" OR "quality assessment" OR "strengths" OR "strength" OR "weaknesses" OR "weakness" OR "research method" OR "research methods" OR "research method*") OR AK=("quality of reporting" OR "reporting quality" OR "reporting qualities" OR "selective reporting" OR "poor reporting" OR "poor reported" OR ("reporting" NEAR/5 ("quality" OR "selective" OR "poor" OR "weak" OR "manner" OR "rigor" OR "improv*")) OR "Data Accuracy" OR

For peer review only

"Research Report standards" OR "quality assessment" OR "strengths" OR "strength" OR "weaknesses" OR "weakness" OR "research method" OR "research methods" OR "research method*") OR AB=("quality of reporting" OR "reporting quality" OR "reporting qualities" OR "selective reporting" OR "poor reporting" OR "poor reported" OR ("reporting" NEAR/5 ("quality" OR "selective" OR "poor" OR "weak" OR "manner" OR "rigor" OR "improv*")) OR "Data Accuracy" OR "Research Report standards" OR "quality assessment" OR "strengths" OR "strength" OR "weaknesses" OR "weakness")))

PubMed
http://www.ncbi.nlm.nih.gov/pubmed?otool=leiden

(("Delphi Technique"[majr] OR "Delphi Technique"[ti] OR "Delphi techniques"[ti] OR "Delphi method"[ti] OR "Delphi methods"[ti] OR "Delphi study"[ti] OR "Delphi studies"[ti] OR "Delphi survey"[ti] OR "Delphi surveys"[ti] OR "Delphi consensus"[ti] OR "Delphi based consensus"[ti] OR "Delphi questionnaire"[ti] OR "Delphi questionnaires"[ti] OR "Delphi research"[ti] OR "Delphi review"[ti] OR "Delphi reviews"[ti] OR "Delphi process"[ti] OR "Delphi processes"[ti] OR "Delphi based"[ti] OR "Delphi procedure"[ti] OR "Delphi procedures"[ti] OR "Delphi assessment"[ti] OR "Delphi assessments"[ti] OR "Delphi approach"[ti] OR "Delphi approaches"[ti] OR "Delphi panel"[ti] OR "Delphi panels"[ti] OR "Delphi round"[ti] OR "Delphi rounds"[ti] OR "Delphi analysis"[ti] OR "Delphi expert"[ti] OR "Delphi experts"[ti] OR "Delphi consultation"[ti] OR "Delphi methodology"[ti] OR "nominal group technique"[ti] OR "nominal group techniques"[ti] OR "nominal group"[ti] OR "nominal groups"[ti] OR "nominal grouping"[ti] OR "consensus recommendation"[ti] OR "consensus recommendations"[ti] OR "consensus development"[ti] OR "consensus activity"[ti] OR "consensus activities"[ti] OR "consensus methodology"[ti] OR "consensus method*"[ti] OR "Consensus Development Conferences as Topic"[majr] OR "RAND"[ti] OR ("Guidelines as Topic"[majr:noexp] AND ("consensus"[tw] OR "delphi"[tw]))) AND ("reporting"[ti] OR "quality of reporting"[tw] OR "reporting quality"[tw] OR "reporting qualities"[tw] OR "selective reporting"[tw] OR "poor reporting"[tw] OR "poor reported"[tw] OR "poorly reported"[tw] OR "Research Report/standards"[majr] OR "Research Design/standards"[mesh] OR "Research Design"[majr:noexp] OR "Writing/standards"[mesh] OR "Writing"[majr] OR "research method"[ti] OR "research methods"[ti] OR "research method*"[ti]))

MEDLINE via OVID
http://gateway.ovid.com/ovidweb.cgi?T=JS&MODE=ovid&NEWS=n&PAGE=main&D=medall

((exp *"Delphi Technique"/ OR "Delphi Technique".ti OR "Delphi techniques".ti OR "Delphi method".ti OR "Delphi methods".ti OR "Delphi study".ti OR "Delphi studies".ti OR "Delphi survey".ti OR "Delphi surveys".ti OR "Delphi consensus".ti OR "Delphi based consensus".ti OR "Delphi questionnaire".ti OR "Delphi questionnaires".ti OR "Delphi research".ti OR "Delphi review".ti OR "Delphi reviews".ti OR "Delphi

For peer review only

process".ti OR "Delphi processes".ti OR "Delphi based".ti OR "Delphi procedure".ti OR "Delphi procedures".ti OR "Delphi assessment".ti OR "Delphi assessments".ti OR "Delphi approach".ti OR "Delphi approaches".ti OR "Delphi panel".ti OR "Delphi panels".ti OR "Delphi round".ti OR "Delphi rounds".ti OR "Delphi analysis".ti OR "Delphi expert".ti OR "Delphi experts".ti OR "Delphi consultation".ti OR "Delphi methodology".ti OR "nominal group technique".ti OR "nominal group techniques".ti OR "nominal group".ti OR "nominal groups".ti OR "nominal grouping".ti OR "consensus recommendation".ti OR "consensus recommendations".ti OR "consensus development".ti OR "consensus activity".ti OR "consensus activities".ti OR "consensus methodology".ti OR "consensus method*".ti OR exp *"Consensus Development Conferences as Topic"/ OR "RAND".ti OR ("Guidelines as Topic"/ AND ("consensus".mp OR "delphi".mp)) OR (("Guidelines".mp OR "guideline".mp) ADJ2 ("consensus".mp OR "delphi".mp))) AND ("reporting".ti OR "quality of reporting".mp OR "reporting quality".mp OR "reporting qualities".mp OR "selective reporting".mp OR "poor reporting".mp OR "poor reported".mp OR "poorly reported".mp OR "Research Report/standards"/ OR exp "Research Design"/st OR *"Research Design"/ OR exp "Writing"/st OR exp *"Writing"/ OR "research method".ti OR "research methods".ti OR "research method*".ti OR ("reporting" ADJ8 ("quality" OR "selective" OR "poor" OR "weak" OR "manner" OR "rigor" OR "improv*")).mp))

Embase
http://ovidsp.ovid.com/ovidweb.cgi?T=JS&PAGE=main&MODE=ovid&D=oemezd

((exp *"Delphi Study"/ OR "Delphi Technique".ti OR "Delphi techniques".ti OR "Delphi method".ti OR "Delphi methods".ti OR "Delphi study".ti OR "Delphi studies".ti OR "Delphi survey".ti OR "Delphi surveys".ti OR "Delphi consensus".ti OR "Delphi based consensus".ti OR "Delphi questionnaire".ti OR "Delphi questionnaires".ti OR "Delphi research".ti OR "Delphi review".ti OR "Delphi reviews".ti OR "Delphi process".ti OR "Delphi processes".ti OR "Delphi based".ti OR "Delphi procedure".ti OR "Delphi procedures".ti OR "Delphi assessment".ti OR "Delphi assessments".ti OR "Delphi approach".ti OR "Delphi approaches".ti OR "Delphi panel".ti OR "Delphi panels".ti OR "Delphi round".ti OR "Delphi rounds".ti OR "Delphi analysis".ti OR "Delphi expert".ti OR "Delphi experts".ti OR "Delphi consultation".ti OR "Delphi methodology".ti OR "nominal group technique".ti OR "nominal group techniques".ti OR "nominal group".ti OR "nominal groups".ti OR "nominal grouping".ti OR "consensus recommendation".ti OR "consensus recommendations".ti OR "consensus development".ti OR "consensus activity".ti OR "consensus activities".ti OR "consensus methodology".ti OR "consensus method*".ti OR exp *"Consensus Development"/ OR "RAND".ti OR (("Guidelines".ti,ab OR "guideline".ti,ab) ADJ2 ("consensus".ti,ab OR "delphi".ti,ab))) AND ("reporting".ti OR "quality of reporting".ti,ab OR "reporting quality".ti,ab OR "reporting qualities".ti,ab OR "selective reporting".ti,ab OR "poor reporting".ti,ab OR "poor reported".ti,ab OR "poorly reported".ti,ab OR *"Methodology"/ OR *"Writing"/ OR "research method".ti OR "research methods".ti OR "research method*".ti OR ("reporting" ADJ8 ("quality" OR "selective" OR "poor" OR "weak" OR "manner" OR "rigor" OR "improv*")).ti,ab))

Cochrane
https://www.cochranelibrary.com/advanced-search/search-manager

(("Delphi Technique" OR "Delphi Technique" OR "Delphi techniques" OR "Delphi method" OR "Delphi methods" OR "Delphi study" OR "Delphi studies" OR "Delphi survey" OR "Delphi surveys" OR "Delphi consensus" OR "Delphi based consensus" OR "Delphi questionnaire" OR "Delphi questionnaires" OR "Delphi research" OR "Delphi review" OR "Delphi reviews" OR "Delphi process" OR "Delphi processes" OR "Delphi based" OR "Delphi procedure" OR "Delphi procedures" OR "Delphi assessment" OR "Delphi assessments" OR "Delphi approach" OR "Delphi approaches" OR "Delphi panel" OR "Delphi panels" OR "Delphi round" OR "Delphi rounds" OR "Delphi analysis" OR "Delphi expert" OR "Delphi experts" OR "Delphi consultation" OR "Delphi methodology" OR "nominal group technique" OR "nominal group techniques" OR "nominal group" OR "nominal groups" OR "nominal grouping" OR "consensus recommendation" OR "consensus recommendations" OR "consensus development" OR "consensus activity" OR "consensus activities" OR "Consensus Development Conference" OR "Consensus Development" OR "Consensus methodology" OR "consensus method*" OR "RAND" OR (("Guidelines" OR "guideline") NEAR/2 ("consensus" OR "delphi"))) AND ("quality of reporting" OR "reporting quality" OR "reporting qualities" OR "selective reporting" OR "poor reporting" OR "poor reported" OR ("reporting" NEAR/5 ("quality" OR "selective" OR "poor" OR "weak" OR "manner" OR "rigor" OR "improv*")) OR "Data Accuracy" OR "Research Report standards" OR "quality assessment" OR "strengths" OR "strength" OR "weaknesses" OR "weakness" OR "research method" OR "research methods" OR "research method*")):ti,ab,kw

Emcare
http://ovidsp.ovid.com/ovidweb.cgi?T=JS&NEWS=n&CSC=Y&PAGE=main&D=emcr

((exp *"Delphi Study"/ OR "Delphi Technique".ti OR "Delphi techniques".ti OR "Delphi method".ti OR "Delphi methods".ti OR "Delphi study".ti OR "Delphi studies".ti OR "Delphi survey".ti OR "Delphi surveys".ti OR "Delphi consensus".ti OR "Delphi based consensus".ti OR "Delphi questionnaire".ti OR "Delphi questionnaires".ti OR "Delphi research".ti OR "Delphi review".ti OR "Delphi reviews".ti OR "Delphi process".ti OR "Delphi processes".ti OR "Delphi based".ti OR "Delphi procedure".ti OR "Delphi procedures".ti OR "Delphi assessment".ti OR "Delphi assessments".ti OR "Delphi approach".ti OR "Delphi approaches".ti OR "Delphi panel".ti OR "Delphi panels".ti OR "Delphi round".ti OR "Delphi rounds".ti OR "Delphi analysis".ti OR "Delphi expert".ti OR "Delphi experts".ti OR "Delphi consultation".ti OR "Delphi methodology".ti OR "nominal group technique".ti OR "nominal group techniques".ti OR "nominal group".ti OR "nominal groups".ti OR "nominal grouping".ti OR "consensus recommendation".ti OR "consensus recommendations".ti OR "consensus development".ti OR "consensus activity".ti OR "consensus activities".ti OR "consensus methodology".ti OR "consensus method*".ti OR exp *"Consensus Development"/ OR "RAND".ti OR (("Guidelines".ti,ab OR "guideline".ti,ab) ADJ2 ("consensus".ti,ab OR "delphi".ti,ab))) AND ("reporting".ti

For peer review only

OR "quality of reporting".ti,ab OR "reporting quality".ti,ab OR "reporting qualities".ti,ab OR "selective reporting".ti,ab OR "poor reporting".ti,ab OR "poor reported".ti,ab OR "poorly reported".ti,ab OR *"Methodology"/ OR *"Writing"/ OR "research method".ti OR "research methods".ti OR "research method*".ti OR ("reporting" ADJ8 ("quality" OR "selective" OR "poor" OR "weak" OR "manner" OR "rigor" OR "improv*")).ti,ab))

Academic Search Premier
http://search.ebscohost.com/login.aspx?authtype=ip,uid&profile=lumc&defaultdb=aph

((TI("Delphi Technique" OR "Delphi Technique" OR "Delphi techniques" OR "Delphi method" OR "Delphi methods" OR "Delphi study" OR "Delphi studies" OR "Delphi survey" OR "Delphi surveys" OR "Delphi consensus" OR "Delphi based consensus" OR "Delphi questionnaire" OR "Delphi questionnaires" OR "Delphi research" OR "Delphi review" OR "Delphi reviews" OR "Delphi process" OR "Delphi processes" OR "Delphi based" OR "Delphi procedure" OR "Delphi procedures" OR "Delphi assessment" OR "Delphi assessments" OR "Delphi approach" OR "Delphi approaches" OR "Delphi panel" OR "Delphi panels" OR "Delphi round" OR "Delphi rounds" OR "Delphi analysis" OR "Delphi expert" OR "Delphi experts" OR "Delphi consultation" OR "Delphi methodology" OR "nominal group technique" OR "nominal group techniques" OR "nominal group" OR "nominal groups" OR "nominal grouping" OR "consensus recommendation" OR "consensus recommendations" OR "consensus development" OR "consensus activity" OR "consensus activities" OR "Consensus Development Conference" OR "Consensus Development" OR "Consensus methodology" OR "consensus method*" OR "RAND" OR (("Guidelines" OR "guideline") N2 ("consensus" OR "delphi"))) OR KW("Delphi Technique" OR "Delphi Technique" OR "Delphi techniques" OR "Delphi method" OR "Delphi methods" OR "Delphi study" OR "Delphi studies" OR "Delphi survey" OR "Delphi surveys" OR "Delphi consensus" OR "Delphi based consensus" OR "Delphi questionnaire" OR "Delphi questionnaires" OR "Delphi research" OR "Delphi review" OR "Delphi reviews" OR "Delphi process" OR "Delphi processes" OR "Delphi based" OR "Delphi procedure" OR "Delphi procedures" OR "Delphi assessment" OR "Delphi assessments" OR "Delphi approach" OR "Delphi approaches" OR "Delphi panel" OR "Delphi panels" OR "Delphi round" OR "Delphi rounds" OR "Delphi analysis" OR "Delphi expert" OR "Delphi experts" OR "Delphi consultation" OR "Delphi methodology" OR "nominal group technique" OR "nominal group techniques" OR "nominal group" OR "nominal groups" OR "nominal grouping" OR "consensus recommendation" OR "consensus recommendations" OR "consensus development" OR "consensus activity" OR "consensus activities" OR "Consensus Development Conference" OR "Consensus Development" OR "Consensus methodology" OR "consensus method*" OR "RAND" OR (("Guidelines" OR "guideline") N2 ("consensus" OR "delphi")))) AND (TI("quality of reporting" OR "reporting quality" OR "reporting qualities" OR "selective reporting" OR "poor reporting" OR "poor reported" OR "reporting guideline" OR "reporting" OR ("reporting" AND ("quality" OR "selective" OR "poor" OR "weak" OR "manner" OR "rigor" OR "improv*")) OR "Data Accuracy" OR "quality assessment" OR "strengths" OR "strength" OR "weaknesses" OR "weakness" OR "research method" OR "research methods" OR "research method*") OR

For peer review only

KW("quality of reporting" OR "reporting quality" OR "reporting qualities" OR "selective reporting" OR "poor reporting" OR "poor reported" OR ("reporting" N5 ("quality" OR "selective" OR "poor" OR "weak" OR "manner" OR "rigor" OR "improv*")) OR "Data Accuracy" OR "Research Report standards" OR "quality assessment" OR "strengths" OR "strength" OR "weaknesses" OR "weakness" OR "research method" OR "research methods" OR "research method*") OR AB("quality of reporting" OR "reporting quality" OR "reporting qualities" OR "selective reporting" OR "poor reporting" OR "poor reported" OR ("reporting" N5 ("quality" OR "selective" OR "poor" OR "weak" OR "manner" OR "rigor" OR "improv*")) OR "Data Accuracy" OR "Research Report standards" OR "quality assessment" OR "strengths" OR "strength" OR "weaknesses" OR "weakness")))

PsycINFO
http://search.ebscohost.com/login.aspx?authtype=ip,uid&profile=lumc&defaultdb=psyh

((TI("Delphi Technique" OR "Delphi Technique" OR "Delphi techniques" OR "Delphi method" OR "Delphi methods" OR "Delphi study" OR "Delphi studies" OR "Delphi survey" OR "Delphi surveys" OR "Delphi consensus" OR "Delphi based consensus" OR "Delphi questionnaire" OR "Delphi questionnaires" OR "Delphi research" OR "Delphi review" OR "Delphi reviews" OR "Delphi process" OR "Delphi processes" OR "Delphi based" OR "Delphi procedure" OR "Delphi procedures" OR "Delphi assessment" OR "Delphi assessments" OR "Delphi approach" OR "Delphi approaches" OR "Delphi panel" OR "Delphi panels" OR "Delphi round" OR "Delphi rounds" OR "Delphi analysis" OR "Delphi expert" OR "Delphi experts" OR "Delphi consultation" OR "Delphi methodology" OR "nominal group technique" OR "nominal group techniques" OR "nominal group" OR "nominal groups" OR "nominal grouping" OR "consensus recommendation" OR "consensus recommendations" OR "consensus development" OR "consensus activity" OR "consensus activities" OR "Consensus Development Conference" OR "Consensus Development" OR "Consensus methodology" OR "consensus method*" OR "RAND" OR (("Guidelines" OR "guideline") N2 ("consensus" OR "delphi"))) OR AB("Delphi Technique" OR "Delphi Technique" OR "Delphi techniques" OR "Delphi method" OR "Delphi methods" OR "Delphi study" OR "Delphi studies" OR "Delphi survey" OR "Delphi surveys" OR "Delphi consensus" OR "Delphi based consensus" OR "Delphi questionnaire" OR "Delphi questionnaires" OR "Delphi research" OR "Delphi review" OR "Delphi reviews" OR "Delphi process" OR "Delphi processes" OR "Delphi based" OR "Delphi procedure" OR "Delphi procedures" OR "Delphi assessment" OR "Delphi assessments" OR "Delphi approach" OR "Delphi approaches" OR "Delphi panel" OR "Delphi panels" OR "Delphi round" OR "Delphi rounds" OR "Delphi analysis" OR "Delphi expert" OR "Delphi experts" OR "Delphi consultation" OR "Delphi methodology" OR "nominal group technique" OR "nominal group techniques" OR "nominal group" OR "nominal groups" OR "nominal grouping" OR "consensus recommendation" OR "consensus recommendations" OR "consensus development" OR "consensus activity" OR "consensus activities" OR "Consensus Development Conference" OR "Consensus Development" OR "Consensus methodology" OR "consensus method*" OR "RAND") OR KW("Delphi Technique" OR "Delphi

For peer review only

Technique" OR "Delphi techniques" OR "Delphi method" OR "Delphi methods" OR "Delphi study" OR "Delphi studies" OR "Delphi survey" OR "Delphi surveys" OR "Delphi consensus" OR "Delphi based consensus" OR "Delphi questionnaire" OR "Delphi questionnaires" OR "Delphi research" OR "Delphi review" OR "Delphi reviews" OR "Delphi process" OR "Delphi processes" OR "Delphi based" OR "Delphi procedure" OR "Delphi procedures" OR "Delphi assessment" OR "Delphi assessments" OR "Delphi approach" OR "Delphi approaches" OR "Delphi panel" OR "Delphi panels" OR "Delphi round" OR "Delphi rounds" OR "Delphi analysis" OR "Delphi expert" OR "Delphi experts" OR "Delphi consultation" OR "Delphi methodology" OR "nominal group technique" OR "nominal group techniques" OR "nominal group" OR "nominal groups" OR "nominal grouping" OR "consensus recommendation" OR "consensus recommendations" OR "consensus development" OR "consensus activity" OR "consensus activities" OR "Consensus Development Conference" OR "Consensus Development" OR "Consensus methodology" OR "consensus method*" OR "RAND" OR (("Guidelines" OR "guideline") N2 ("consensus" OR "delphi")))) AND (TI("quality of reporting" OR "reporting quality" OR "reporting qualities" OR "selective reporting" OR "poor reporting" OR "poor reported" OR "reporting guideline" OR "reporting" OR ("reporting" AND ("quality" OR "selective" OR "poor" OR "weak" OR "manner" OR "rigor" OR "improv*")) OR "Data Accuracy" OR "quality assessment" OR "strengths" OR "strength" OR "weaknesses" OR "weakness" OR "research method" OR "research methods" OR "research method*") OR KW("quality of reporting" OR "reporting quality" OR "reporting qualities" OR "selective reporting" OR "poor reporting" OR "poor reported" OR ("reporting" N5 ("quality" OR "selective" OR "poor" OR "weak" OR "manner" OR "rigor" OR "improv*")) OR "Data Accuracy" OR "Research Report standards" OR "quality assessment" OR "strengths" OR "strength" OR "weaknesses" OR "weakness" OR "research method" OR "research methods" OR "research method*") OR AB("quality of reporting" OR "reporting quality" OR "reporting qualities" OR "selective reporting" OR "poor reporting" OR "poor reported" OR ("reporting" N5 ("quality" OR "selective" OR "poor" OR "weak" OR "manner" OR "rigor" OR "improv*")) OR "Data Accuracy" OR "Research Report standards" OR "quality assessment" OR "strengths" OR "strength" OR "weaknesses" OR "weakness")))

For peer review only

**Author, year**	
**Assessor**	

**Background**<br>1.1 Does the study suggest anything about how or if consensus papers should report the context or rationale for choosing a consensus method over other methods?	
**Background**<br>1.2 Does the study suggest anything about how/what or if consensus papers should report the objectives of the consensus exercise?	

**Methods**<br>2.1 Does the study suggest anything about how/what or if consensus papers should report regarding:<br>A literature search/strategy?	
**Methods**<br>2.2 Does the study the suggest anything about how/what or if consensus papers should report regarding:<br>Inclusion and exclusion criteria for the literature search?	
**Methods**<br>2.3 Does the study suggest anything of what or if consensus report should report on panel composition, n of participants, expertise, origin? Prespecified?	
**Methods**<br>2.4 Does the study suggest anything of how or if PPI (public patient involvement) activity should be reported?	
**Methods**<br>2.5 Does the study suggest anything about what or if consensus papers should report regarding panel recruitment strategies, invitations? Any level of detail specified?	
**Methods**<br>2.6 Does the study suggest how or if consensus papers should report the consensus criteria/threshold (or the level of agreement considered to reach consensus)?	
**Methods**<br>2.7 Does the study suggest how or if consensus papers should report how decision of approval of an item will be made?	
**Methods**	

2.8 Does the study suggest anything about what level of detail should be reported regarding the number of Delphi rounds or if this should be reported?	
**Methods**<br>2.9 Does the study suggest anything about what level of detail should be reported regarding the criteria used for defining the number of rounds? (why 2-3 or more e.g.) or if this should be reported?	
**Methods**<br>2.10 Does the study suggest anything about the details that should be reported regarding the time between rounds, if it should be prespecified or if this should be reported?	
**Methods**<br>2.11 Does the study suggest anything about details that should be reported of the names of the techniques of non-Delphi methods used to gather participants' inputs and reach consensus?	
**Methods**<br>2.12 Does the study suggest anything of what or in which detail should be reported regarding tool or electronic system used for Delphi? (If Delphi was used)? Or if this should be reported?	
**Methods**<br>2.13 Does the study suggest anything about how or in what level of detail the anonymity of participants (in Delphi or other methods) has to be reported? Or if this should be reported?	
**Methods**<br>2.14 Does the study suggest anything about how to report, and in what level of detail, the feedback for panellists (in Delphi rounds or other methods) process? Or if this should be reported?	
**Methods**<br>2.15 Does the study suggest anything about how or if data synthesis/analysis should be reported (from any consensus method used and how this was calculated statistically) and in what level of detail?	
**Methods**<br>2.16 Does the study suggest anything about how or if piloting should be reported and in what level of detail (e.g. understanding of consensus items, platforms used, tools used)?	
**Methods**	

For peer review only

2.17 Does the study suggest anything about how or if the role of Steering Committee members should be reported?	
**Methods**<br>2.18 Does the study suggest anything on what or if should be described regarding COI or funding?	
**Methods**<br>2.19 Does the study suggest anything on what should be described of how is dealt with COI of panellist (not allowed to vote when there is COI)? Or if this should be described	

**Results**<br>3.1 Does the study suggest anything on how to report the initial evidence search (presentation of results of the literature review)?	
**Results**<br>3.2 Does the study suggest anything on how to report n of studies found?	
**Results**<br>3.3 Does the study recommend which detail should be used when reporting panellists drop-outs (numbers and reasons)? Or if this should be reported?	
**Results**<br>3.4 Does the study suggest how or if approval rates per item shared with respondents for each round should be reported in the Results section?	
**Results**<br>3.5 Does the study suggest anything about in which detail the items that have been dropped should be reported? (reasons e.g.) Or if this should be reported?	
**Results**<br>3.6 Does the study make any recommendation on how to report the collection, synthesis and use of comments from panellists? Or if this should be reported?	
**Results**<br>3.7 Does the study suggest regarding how the final list of items (for clinical guideline or reporting guideline) should be reported? Or if this should be reported?	

**Discussion**<br>4.1 Does the paper suggest anything about reporting the limitations and strengths of the study and how? Or if this should be reported?	
**Discussion**	

For peer review only

4.2 Does the paper suggest anything about what or in which detail the applicability generalisability, and reproducibility of the study should be reported? Or if this should be reported?	

**5.1 Any other item proposed by the paper that is not captured in other columns?**	
**5.2 Any other item not proposed by the paper, but you think that could be added (not fitting the categories above)?**	

**Examples of text with well reported methods/results (for E&E document) - write NA if none was cited or found by you**	
**Additional comments from assessor**	

For peer review only

Data on reporting quality (*recommendations in italics)*

Study	What is stated regarding reporting quality?
Banno 2019[32]	• "The reporting quality of the Delphi technique in reporting guidelines is unknown even though the use of the Delphi technique was recommended in the guidance for reporting guidelines." (Note: This is a protocol for the systematic review of 2020.)<br>*4 quality score items are summarised of Delphi methods used in reporting guidelines*.
Banno 2020[16]	• "Reproducible criteria of participants, number of rounds, criteria for dropping items, and stopping criteria other than rounds were found for 87%, 97%, 69%, and 13%, respectively of reporting guidelines developed with the Delphi method. The total score of reporting quality was 2 or more in 94% of reporting guidelines using the Delphi method."<br>*4 quality score items are summarised of Delphi methods used in reporting guidelines*.
Boulkedid 2011[17]	• "Study reports did not consistently provide details that are important for interpreting the results. For example, only 39% of studies reported that individual feedback was given between rounds and the method used to define a consensus was specified in only 77% studies. Moreover, response rates for all rounds were reported in only 31% of studies. Information on both points is needed to evaluate the validity and credibility of the results. If the Delphi method is incompletely described this may affect the overall quality of the final consensus and the selected indicators are unlikely to gain the level of credibility needed for adoption I clinical practice."<br>• "The Delphi procedure is valuable for achieving a consensus about issues where none existed previously. However, our findings indicate a need for improving the use and reporting of this technique."<br>*Table 5 provides recommendations for reporting the Delphi procedure.*
Chan 2019[20]	• "This lack of clear definition has led to considerable confusion and substantial variation in the quality of reporting of Delphi studies"<br>• "One-third of medical education Delphi studies failed to report that a literature review on the topic of interest had been conducted , and over half failed to report key aspects such as what background information was provided to participants; the response rate for each round; what formal feedback of group rating was shared between rounds; a statement that anonymity was maintained; and a clear definition of consensus."<br>• "Lack of clarity in the report in the reporting of procedures and methodological choices associated with the modified Delphi studies can prevent readers from effectively appraising and interpreting findings."<br>• "Methodological rigor and transparent reporting are essential to assure readers that the consensus results are applicable to their environment, and to translate expert opinion into practice."<br>*Box 1 provides recommendations to improve reporting.*
Diamond 2014[18]	• "Definitions of consensus vary widely and are poorly reported. Improved criteria for reporting of methods of Delphi studies are required."<br>• "Methodologic criteria are proposed for the reporting of Delphi studies."<br>• "Despite the fact that the most Delphi studies in our cohort had consensus as their aim, in only a minority of the Delphi studies reviewed was consensus defined with a specific criterion. Furthermore, this criterion was the reason for termination of the Delphi process, usually on the basis of an *a priori* definition."<br>• "We believe that there is a need to improve the reporting of Delphi studies, along the lines of a CONSORT-like guideline, as is used for randomized controlled trials."<br>*Methodologic criteria are proposed for the reporting of Delphi studies.*

For peer review only - http://bmjopen.bmj.com/site/about/guidelines.xhtml

Gattrell 2019[29]	"At present there are a lack of standard, validated reporting guidelines for publications reporting the results of Delphi panel studies."<br><br>Quality assessment: Methodological quality<br>• The type of Delphi technique used, or the modifications to the method, was not outlined in all publications (included in 62/90 publications; 68.9%).<br>• Just over half of all publications stated that there was some diversity amongst participants and clearly outlined the methods for the selection of panellists.<br>• Agreement and consensus thresholds should be defined prior to study commencement, but in 40% of publications it was unclear, or not stated whether these thresholds were predefined.<br>• Anonymised responses are typically conveyed back to the group after each round, but this was clearly reported in less than half (38.9%) of publications.<br><br>Quality assessment: Reporting quality and transparency (Figure 3b).<br>• The funding source was not clearly disclosed in over a third of publications, and almost twice as many publications did not clearly disclose the funder's role.<br>• Conflicts of interest were clearly described in most publications (included in 79/90 publications; 87.8%).<br>• Clear disclosure of external support was not evident in the majority of the publications.
Grant 2018[24]	• "Specifying the analysis procedure for consensus is therefore a critical consideration when designing consensus-oriented Delphi processes in health research."<br>• "Without prespecifying their analysis procedures in a study registry, health researchers conducting consensus-oriented Delphi processes can mine for and selectively report the most desirable set of items reaching consensus and even present the reported analysis as the only one conducted. Undisclosed flexibility in data collection, analysis, and reporting is a growing concern in empirical research."<br>• "Without preregistering and reporting all of the attempted analysis procedures and when they were attempted, the extent and impact of researchers trying different analysis procedures is nearly impossible for peer reviewers, editors, and consumers of Delphi research to assess."<br>• "To be completely registered, the preanalysis plan should precisely describe the essential elements of the analysis procedure for determining consensus (see Box 2)."<br>• "Researchers should use existing guidance on reporting completed Delphi processes to provide sufficient information for comparing the final article to the registered preanalysis plan [1,12,42], with particular attention in the final article to any changes from the preanalysis plan in the items, rating criteria, analytic procedure (measure and threshold), and data and participants included in the analysis."<br>*Box 2 provides a minimum set of items to include in prospectively registered preanalysis plans for consensus-oriented Delphi processes.*
Hasson 2017[27]	• "Figure 1 Areas for reporting on the Delphi survey technique."<br>• "In Delphi surveys there exists no consistent method for reporting findings (Schmidt 1997) and a review of the literature showed that a number of approaches have been used."<br>• "The following diagram attempts to outline those sections that researchers should report upon when using the Delphi. This will help readers to judge the reliability of the method and the results obtained."<br>*Followed by a checklist of issues, which could be used by researchers.*

For peer review only

Humphrey-Murto 2017[21]	• "The authors set out to describe the use of consensus methods in medical education research and to assess the reporting quality of these methods and results."<br>• "Improved criteria for reporting are needed."<br>• "Our findings suggest that the reporting quality and standardization of consensus methods in medical education research varies greatly. The following areas appeared particularly problematic and were often left out or poorly described in the articles we reviewed: conducting a literature review to inform the consensus method; providing background information to participants; reporting the number of participants after each round; describing the level of anonymity used in the study; providing participants with feedback of group ratings; and articulating the definition of consensus used in the study."<br>*Recommendations for improvements in these areas are provided in Discussion.*
Humphrey-Murto 2017[28]	• "Consensus group methods are widely used in research to identify and measure areas where incomplete evidence exists for decision-making. Despite their widespread use, these methods are often inconsistently used and reported."<br>• "This paper and associated Guide aim to describe these methods and to highlight common weaknesses in methodology and reporting."<br>• "The AMEE Guide describes these methods to provide a "how to" approach, highlight common weaknesses in methodology and reporting, and outline recommendations for reporting future consensus based studies."<br>• "Four recent reviews using the Delphi in health care and policy-related research have systematically explored deficiencies in the use and reporting of consensus group methods. Collectively, these studies have noted deficiencies regarding: information provided to the participants at the start of Delphi, reporting response rates, feedback to participants, level of anonymity, outcomes after each round and the definition of consensus."<br>*This guide provides recommendations for improvement of reporting.*
Humphrey-Murto 2019[25]	• "Studies using the Delphi for selecting performance indicators for healthcare, for medical and nursing education, or for determining outcomes to measure in clinical trials, often fail to adequately report sufficient methodological detail. Examples include poor reporting of background information provided to participants, response rates for all rounds, level of anonymity, formal feedback between rounds, and the definition of consensus."<br>*OMERACT Delphi consensus checklist is provided in Figure 1.*
Jünger 2017[12]	• "Substantial variation was found concerning the quality of the study conduct and the transparency of reporting of Delphi studies used for the development of best practice guidance in palliative care. Since credibility of the resulting recommendations depends on the rigorous use of the Delphi technique, there is a need for consistency and quality both in the conduct and reporting of studies. To allow a critical appraisal of the methodology and the resulting guidance, a reporting standard for Conducting and Reporting of DElphi Studies (CREDES) is proposed."<br>Study adds in Box 3*"Recommendations for the Conducting and REporting of DElphi Studies (CREDES)."*
Ng 2018[30]	• "Given the variance in the use of Delphi method, reporting guidelines could help improve reporting of this research, and thereby allow readers to be aware of the accuracy of data and conclusions."<br>• "We anticipate the implementation of this will promote transparent and accurate reporting of research using Delphi method for obtaining quantitative data."<br>*A set of reporting guidelines is proposed.*
Niederberger 2020[26]	• "Significant weaknesses exist in the quality of the reporting."

For peer review only - http://bmjopen.bmj.com/site/about/guidelines.xhtml

	• "Criteria for evaluating the quality of their execution and reporting also appear to be necessary."<br>• "A specific definition of the underlying Delphi technique was found in 61% (ID11) and 88.2% (ID4) of the Delphi articles investigated."<br>• "Most of the Delphi studies analyzed in the reviews reported on the number of participating experts. The rates for the initial round were between 84% (ID6) and 100% (ID12). Four of the reviews investigated whether the number of experts was stated for each round (ID4, ID7, ID11, ID12). In one review based on 10 Delphi studies from health sciences (ID7), the authors discovered that the number of experts per round was stated in all articles. A review of 48 studies in a medical context indicated that the number of invited experts was stated less frequently with each round (ID6). Seven of the 12 reviews investigated whether the backgrounds of the experts had been reported, what kind of expertise they possessed, and the criteria according to which they were selected (ID1, ID3, ID4, ID6, ID9, ID11, ID12). One review of Delphi techniques in a health context determined that the criteria for selecting the experts was reproduced in 65 of 100 articles (65%) (ID3) included in that particular review. In other reviews with a more specific focus, such as on health care, palliative medicine, or health promotion, the rates were higher at 69% (ID11), 70% (ID9) and 79% (ID1), respectively. Based on the results of the reviews, the criteria by which the experts were selected and approached was not always clear. In one review of 100 studies from the care sector, the proportion of articles with unclear selection criteria was 11.2% (ID4), while the proportion was 93.3% in a review of 15 studies from the clinical sector (ID12)."<br>• "Seven of the 12 reviews determined whether and when consensus was defined in the Delphi studies (ID1, ID3, ID4, ID6, ID9, ID11, ID12). The number of studies in which consensus was defined in the article was between 73.5% (ID3) and 83.3% (ID9) in the reviews."<br>• "The authors of seven reviews investigated whether the number of Delphi rounds was published (ID1, ID3, ID4, ID6, ID9, ID11, ID12). The number of Delphi rounds was stated in most of the Delphi studies (e.g., ID1 82.5%, ID4 91%, ID6 100%, ID9 49.3%, ID12 93.3%). Six of the reviews included a report of the generation of the questionnaire (ID1, ID4, ID6, ID9, ID11, ID12). They demonstrated that up to 96.3% of the investigated articles reported on how the items for the questionnaire were developed (ID1). In contrast, this rate stood at 33.3% in the review of palliative care articles (ID9). The authors of two reviews investigated the question of how the items were changed during the Delphi process based on the judgments submitted by the experts (ID3, ID12). In one of the reviews, the authors indicated that 59% of the analyzed articles had defined criteria for dropping items (ID3). In another review, the authors stated that all of the investigated Delphi studies included a report of "what was asked in each round" (ID12, p. 2). The authors of the reviews reported about the feedback in most of the Delphi studies (ID11 67.9%, ID12 93.3%). The information provided about the response rate per Delphi round was less (ID1 and ID4 39%). According to the results of the reviews, around half of the studies did not provide information about the feedback design between the Delphi rounds (ID1 40%, ID4 55.1%, ID6 37.7% ID12 40%). According to the authors of the review on health promotion, the process—from formulating the issue being investigated through to the development of the questionnaire—was in general similar to a "black box," and the methodological quality of the survey instrument was almost impossible to evaluate using the published information (ID11, p. 318)."<br>• "Our results also indicate deficits both in carrying out and also reporting Delphi techniques."

For peer review only

	• "The findings in the reviews we analyzed indicated that there is no uniform process for carrying out and reporting Delphi techniques."
Paré 2013[22]	• "Thirty-one percent of the articles in our sample provided a detailed description of the expert recruitment and selection process, 43% provided only limited details, and 26% did not provide any details."<br>• "All of the articles in our database (n = 42) specified the criteria that were used to select the panel of experts. Position is by far the most used criteria (71%), followed by relevant professional experience (57%), geographic location (7%), and education level (5%)."<br>• "38% of the studies provided detailed information about the participating experts [e.g., 44], 40% provided minimal information [e.g., 2], and 22% did not provide any description".<br>• "The anonymity of the experts was reported in virtually all of the studies (95%) in our sample."<br>• "Only 29% of all of the studies reported the response rate to the initial request for participation."<br>• "35 studies (83%)reported the size of the panels. The majority of the studies (n = 21) reported a panel size between 7 and 30, only one study reported a size of 6 or less, and 13 studies reported panel sizes above 30. Nine studies (19%) examined multiple panels of experts."<br>• "Only 17% of these Delphi studies reported that a pretest of the instruments had been conducted."<br>• "24 studies out of 27 (89%) reported the brainstorming instructions that were sent to the experts."<br>• "Only 8 studies (30%) reported the use of this recommendation. (i.e. Have the experts comment and validate the consolidated list)."<br>• "The vast majority of the studies (85%) reported the final number of items at the end of phase 1."<br>• "Among the 25 studies that did not include this phase (i.e. narrowing down phase), 68% explicitly justified this choice (e.g., the number of items at the end of phase 1 are equal or less than 20 as suggested by Schmidt."<br>• "All 17 studies clearly described the narrowing down instructions that were given to the experts."<br>• "65% of the studies clearly specified their item selection rule."<br>• "Most of the studies (82%) reported the final number of items at the end of the second phase."<br>• "All 42 articles described clearly the ranking instructions that were provided to the experts."<br>• "Almost all of the studies (95%) in our sample reported the statistics that were used for data analysis."<br>• "31% of the studies in our database specified a clear stopping rule."<br>• "Only 15 studies (36%) reported the final consensus rate."<br>• "29 of the 42 studies had multiple rounds of ranking. Of these, the feedback that was provided to the experts in between the rounds included the mean ranks of items (69% of studies), an interpretation of the Kendall's W coefficient (3%), the expert's prior responses (59%), and the comments made by the other experts (38%)."<br>*Recommendations regarding what to report are provided throughout the Results section as well as in the Discussion.*
Resemann 2018[31]	• "Reporting of the Delphi method was critiqued against the AGREE Reporting Checklist."<br>• "All studies reported consensus results. The majority (8/11 [73%]) used a two-stage modified Delphi method, while the remainder used a classic three-stage process. Literature searches guided the development of statements for Delphi panel review in the majority of studies, but only 2/11 (18%) conducted

For peer review only

	systematic literature reviews and merely 6/11 (55%) of studies reported the number of statements assessed. Furthermore, 7/11 (64%) did not report collecting panellist feedback to inform subsequent Delphi stages, 5/11 (45%) of studies did not describe the rating scales used, and 2/11 (18%) omitted reporting the level of consensus reached"<br>• "There is a need for improved reporting of Delphi methods".
Waggoner 2016[23]	• "Despite the widespread utility of consensus methods and the variety of approaches available, there is a lack of guidelines for conducting such studies. This lack of stringency in guidelines for conducting consensus studies has led to variability not only in reporting results but in conducting the studies themselves."<br>• "Many studies describe their methods for collecting data and that they did have a benchmark that would point to a consensus, but a lack of a description of the analytical techniques is apparent in many studies."<br>• "In addition to the lack of descriptive techniques in these articles, there is a wide range of criteria that points to consensus. How these particular benchmarks are determined is also not a topic in many of the studies. Given the lack of current research, we believe that the methodology used I subsequent studies should be described more thoroughly in the manuscript."<br>• "We set out to determine best practices for conducting such research as well as reporting on results in the hopes that future studies are more reliable and valid."<br>*This article provides guidance for reporting of various consensus methods.*
Wang 2015[19]	• "Adoption of reporting guidelines is associated with improved reporting quality of research."<br>• "For example, 28 % of the included guidelines reported no information about consensus, and 57 % were silent about how the feedback after consensus was dealt with."<br>• "In addition to the methodology, only 31 % reported formal consensus method."<br>• "Among guidelines developed through consensus, 30 (50 %) reported group member identification and 31 (52 %) reported member recruitment. Of those who identified members, 27 (45 %) reported specialties of experts, 20 (32 %) described information of members, such as names and institutions, and four (7 %) gave the selection criteria. For those who recruited members, even (12 %) described the recruit methods, for instance, through e-mail, study co-chairs, or group decision. In guidelines developed by a working group, 22 (37 %) reported the number of experts participating in guideline development (median 32, range 3–115). Eleven (18 %) guidelines reported the endpoint of consensus process, which were all terminated after a fixed number of rounds (Table 2). In addition, the inclusion criteria of items were given in eight (13 %) guidelines. For example, items meeting the median score of eight or higher in the final round were included."<br>• "11 (18 %) described the pilot methods, seven (12 %) described the feedback information requirement and five (8 %) gave the methods for feedback collection."<br>• "More than 30 % of the reporting guidelines did not report consensus. For those who did, details of consensus methods were poorly reported."<br>• "Consensus methods should be supported by developers, and the reporting of the methods should be improved."<br>*Recommendations for Consensus methods are provided, but more about improvement of applying and reporting using all other reporting guidelines, but some items are applicable for consensus methodology as well (e.g. reporting COI and funding.*

For peer review only

**Background**<br>1.1 Does the study suggest anything about how or if consensus papers should report the context or rationale for choosing a consensus method over other methods?	1) Research problem clearly defined and topic and method justification should be reported [Hasson 2000, Figure 1 and page 1013]<br><br>2) Selection of one consensus method over another should be evident if the purpose is clearly stated. [Humphrey-Murto 2017 Med Teach page 16]<br><br>3) What is the rationale for selecting the Delphi procedure? [Humphrey-Murto 2019, Figure 1]<br><br>4) The choice of the Delphi technique as a method of systematically collating expert consultation and building consensus needs to be well justified. A rationale for the choice of the Delphi technique as the most suitable method needs to be provided [Jünger 2017, Box 3, items 1 and 8]
**Background**<br>1.2 Does the study suggest anything about how/what or if consensus papers should report the objectives of the consensus exercise?	1) Define the study objective [Boulkedid 2011, Table 5 page 7]<br><br>2) Define the purpose of the study [Chan 2019, Box 1]<br><br>3) Is the objective of the Delphi study to present results (eg, a list or statement) reflecting the consensus of the group, or does the study aim to merely quantify the level of agreement? [Diamond 2014, Table 6 and page 403] If the aim of the Delphi study is to elicit consensus, then a clear definition for what constitutes consensus should be provided a priori together with threshold values that specify when consensus is reached. If the investigators plan to only quantify the degree of consensus, but not have consensus as a criterion to stop the Delphi study, this should also be explicitly stated [Diamond 2014, page 406]<br><br>4) Research problem clearly defined and topic and method justification should be reported [Hasson 2020, Figure 1 and page 1013]<br><br>5) Authors must provide a clear purpose for their study or line of inquiry [Humphrey-Murto 2017 Med Teach, page 16]<br><br>6) The purpose of the study should be clearly defined and demonstrate the appropriateness of the use of the Delphi technique as a method to achieve the research aim. A rationale for the choice of the Delphi technique as the most suitable method needs to be provided [Jünger 2017, item 8]

For peer review only

	The Delphi technique is a flexible method and can be adjusted to the respective research aims and purposes. Any modifications should be justified by a rationale and be applied systematically and rigorously" [Jünger 2017, item 2]

**Methods**<br>2.1 Does the study the suggest anything about how/what or if consensus papers should report regarding:<br>A literature search/strategy?	1) Describe the selection and preparation of the scientific evidence for the participants [Chan 2019, Box 1]<br><br>2) A literature review should be reported [Hasson 2000, Figure 1]<br><br>3) "We suggest that this important step must be described", but they don't say how. [Humphrey-Murto 2017 AMA, page 1493 and 1496 Partially]<br><br>4) Describe the selection and preparation of the scientific evidence for the participants [Humphrey-Murto 2017 Med Teach, page 16]<br><br>5) Only implying it should happen and be reported [Resemann 2018]
**Methods**<br>2.2 Does study the suggest anything about how/what or if consensus papers should report regarding:<br>Inclusion and exclusion criteria for the literature search?	1) Clear definition of the selection criteria and/or the definition used in the Delphi questionnaire; criteria for selection should be reported [Boulkedid 2011, Table 5, Appendix S1 item 2]<br><br>2) Describe how items were selected for inclusion in questionnaire, in sufficient detail [Chan 2019, Box 1]<br><br>3) Clear selection criteria should be prespecified [Paré 2013 page 210]
**Methods**<br>2.3 Does the study suggest anything of what or if consensus report should report on panel composition, n of participants, expertise, origin? Prespecified?	1) The method used to select participants is stated. Number and type of participant subgroups (eg, patients, generalists and experts) are needed [Banno 2019, page 2 item 1]<br><br>2) The method to include and exclude participants was described. The number and type of participant subgroups (e.g., patients, generalists, and experts) were essential to record [Banno 2020, page 52 item 1]<br><br>3) How the experts were chosen (e.g., willingness to participate, expertise, or membership in an organization); Composition and characteristics of the panel, number of participants (diagram of participant flow); number invited, how they were chosen, whether they were described (age, sex, specialty), years of experience, single or from multiple

For peer review only

	specialties, inclusion of multiple stakeholders, types of stakeholders [Boulkedid 2011, page 2, Table 5, Appendix S1 item 9-15]<br><br>4) Describe how participants were selected and their qualifications. Include description of facilitator credentials [Chan 2019, Box 1]<br><br>5) Were criteria for participants reproducible? How will participants be selected or excluded? [Diamond 2014, Table 5 and 6]<br><br>6) Was there heterogeneity in panel membership and is the method for selection of experts clearly defined [Gattrell 2019, Table 1]<br><br>7) Expert selection process and characteristics should be reported in detail [Hasson 2000, page 1009, 1013]<br><br>8) How many participants were involved? We noted that the type of expertise required of participants was usually not clearly described [Humphrey-Murto 2017 AMA, page 1493 and 1494]<br><br>9) Describe how the participants were selected and their qualifications: if the NGT or RAND/UCLA is used, describe facilitator's credentials. Whatever the makeup of the expert panel, the authors must provide a rationale and justify their choices [Humphrey-Murto 2017 Med Teach]<br><br>10) How many stakeholder/participant groups will be involved in each step? Provide a rationale for inclusion or exclusion and define the stakeholder groups [Humphrey-Murto 2019, Fig 4]<br><br>11) Criteria for the selection of experts and transparent information on recruitment of the expert panel, sociodemographic details including information on expertise regarding the topic in question, (non)response and response rates over the ongoing iterations should be reported [Jünger 2017, Box 3 9]<br><br>12) Describing expert panel selection with eligibility criteria and including conflicts of interest [Ng 2018]<br><br>13) The number of experts in each round should be stated. The backgrounds of the experts should be reported, what kind of expertise they possessed, and the criteria according to which they were selected [Niederberger 2020, page 4]

For peer review only

	14) Explicit procedures for expert selection; Clear selection criteria; Clear selection criteria should be prespecified and may include the candidates' years of related experience, or tenure in a position that is relevant to the subject under study Report the response rate to the initial call for participation; provide detailed information about the participating experts (profile) to better allow judgments about their credibility [Paré 2013, page 210, Table 3]<br><br>15) Explain how groups were chosen. Consensus Development Panels: Panel composition: the panel should be made up of experts in the field; the publication should report on how they were chosen and why; [Waggoner 2016, page 665, 667]<br><br>16) Implied by mentioning that detailed information on participants was lacking in some reporting guidelines. Page 5 Report specialties of experts, names and institutions, the selection criteria [Wang 2015]
**Methods**<br>2.4 Does the study suggest anything of how or if PPI (public patient involvement) activity should be reported	No data
**Methods**<br>2.5 Does the study suggest anything about what or if consensus papers should report regarding panel recruitment strategies, invitations? Any level of detail specified?	1) The use of specific methods to encourage the experts to respond (e.g., stamped addressed envelope for returning the questionnaire and financial compensation) [page 2] and recommendation to report whether special techniques were used to invite participants [Boulkedid 2011, Appendix S1 item 21]<br><br>2) Criteria for the selection of experts and transparent information on recruitment of the expert panel, socio- demographic details including information on expertise regarding the topic in question, (non)response and response rates over the ongoing iterations should be reported" [Jünger 2017, Box 3, 9]<br><br>3) provide a detailed description of the expert recruitment and selection process [Paré 2013, page 215 first bullet on the right]<br><br>4) method of obtaining participants should be described [Waggoner 2016, page 667]
**Methods**	1) The method used to define a consensus among panel members; , whether the percentage of agreement was determined; Whether a cut-off (e.g., median value) was used to select indicators [page 2] Consensus definition at each

For peer review only

2.6 Does the study suggest how or if consensus papers should report the consensus criteria/threshold (or the level of agreement considered to reach consensus)?	round [page 7, Appendix item 28] how was consensus obtained [page 7, Appendix item 28] definition of consensus should be reported [Boulkedid 2011, table 5]<br><br>2) Clearly describe how consensus was defined [Chan 2019, Box 1]<br><br>3) Need to define criteria for consensus and to document the degree of agreement together with the results of the Delphi process. Should be defined a priori. [Diamond 2014, page 404 and table 6]<br><br>4) Was the agreement/consensus threshold predefined? [Gattrell 2019, table 1]<br><br>5) Box 2 Specific threshold for the chosen measure (e.g., median of at least 7 on a nine-point scale and an interquartile range of less than 2) [Grant 2018, p 97]<br><br>6) Determine the criteria and the meaning of `consensus' in relation to the studies [Hasson 2020, page 1013]<br><br>7) No. They do state that "articulating the definition of consensus used" was identified as "particularly problematic and were often left out or poorly described", and that "the most concerning issue we identified was that consensus was often not defined a priori. Only 43.2% of the articles we reviewed reported their definition of consensus at the start of the study." But they do not suggest how to report. [Humphrey-Murto 2017 AMA]<br><br>8) Clearly describe how consensus was defined [Humphrey-Murto 2017 Med Teach, page 18]<br><br>9) suggests definition of consensus should be reported [Humphrey-Murto 2019, table 1, also fig 1 and page 1044]<br><br>10) Definition of consensus. Unless not reasonable due to the explorative nature of the study, an a priori criterion for consensus should be defined. This includes a clear and transparent guide for action on (a) how to proceed with certain items or topics in the next survey round, (b) the required threshold to terminate the Delphi process and (c) procedures to be followed when consensus is (not) reached after one or more iterations". Definition and attainment of consensus. It needs to be comprehensible to the reader how consensus was achieved throughout the process, including strategies to deal with non-consensus". "If an a priori definition of consensus is not realistic due to the explorative nature of the study, it should be identified and established by the research team in the course of the process." [Jünger 2017, item 12]

For peer review only

	11) How was consensus defined and measured? What role did the stability of the answers play? [Niederberger 2020, Table 2] Whether and when consensus was defined in the Delphi studies. Was consensus defined a priori in advance of development of the questionnaire. [Niederberger 2020, Table 5] How was consensus measured, e.g. percentage agreement, units of central tendency (especially median) or a combination of percent agreement within a certain range and for a certain threshold. [Niederberger 2020, page 6]<br><br>12) NGT explain criteria used to determine how and when a consensus was met Consensus Development Panels: Explain what constituted consensus and how this was assessed. [Waggoner 2016, page 665] Delphi Explain what constituted consensus and how this was assessed. [Waggoner 2016, page 667]<br><br>13) The endpoint of consensus [Wang 2015, page 5]
**Methods**<br>2.7 Does the study suggest how or if consensus papers should report how decision of approval of an item will be made?	1) Whether the percentage of agreement was determined [page 2] We recorded the method used to define a consensus among panel members, whether the percentage of agreement was determined, and whether a cut-off (e.g., median value) was used to select [Boulkedid 2011, Appendix S1 item 16 (technique method)]<br><br>2) Reporting on each round separately illustrates clearly the array of themes generated in round one and gives an indication of the strength of support for each round. The presentations of findings are important and findings from subsequent rounds should be reported in a summarized format to indicate the relative standing of each of the opinions. [Hasson 2020, page 1013]<br><br>3) (Non)response and response rates over the ongoing iterations should be reported [Jünger 2017, item 9]
**Methods**<br>2.8 Does the study suggest anything about what level of detail should be reported regarding the number of Delphi rounds or if this should be reported?	1) Was the number of rounds to be performed stated (not how it should be reported, but implies it should be) [Banno 2019, page 2 under item 2]<br><br>2) Was the number of rounds to be performed stated? [Banno 2020, 3.4, table 3]<br><br>3) Describe the number of rounds planned [Chan 2019, Box 1]

	4) Specify a maximum number of rounds [page 404] what was the reason to stop the delphi [Diamond 2014, table 3] What criteria will be used to determine to stop the Delphi process or will the Delphi be run for a specific number of rounds only [Diamond 2014, table 6, table 1 item 2]<br><br>5) number and outline per round should be reported also page 1013 [Hasson 2020, fig 1]<br><br>6) Describe the number of rounds planned and/or criteria for terminating the process [Humphrey-Murto 2017 Med Teach, page 17]<br><br>7) Only implying that x number of rounds are necessary [Humphrey-Murto 2017 AMA]<br><br>8) The methods employed need to be comprehensible; information about the number and design of survey rounds, [Jünger 2017, Box 3 item 10]<br><br>9) Not specifically under item 4 in table 2 report of the specific process used? How many rounds were used in the Delphi technique [Niederberger 2020]<br><br>10) If a study goes beyond the agreed number of rounds (review suggests 2 rounds are required), this should be explained [Waggoner 2016, page 667]
**Methods**<br>2.9 Does the study suggest anything about what level of detail should be reported regarding the criteria used for defining the number of rounds? (why 2-3 or more e.g.) or if this should be reported?	1) Implied in Banno 2020 The prespecified criteria for stopping the Delphi process, other than a statement of the number of rounds, were clarified [Banno 2020]<br><br>2) Describe the number of rounds planned and criteria for terminating the process [Chan 2019, Box 1]<br><br>3) Describe the number of rounds planned and/or criteria for terminating the process [Humphrey-Murto 2017 Med Teach, page 17]<br><br>4) They, imply that the number of rounds is an important thing to report -- but they do not state this as a suggestion.[Humphrey-Murto 2017 AMA]<br><br>5) Will the number of rounds be decided a priori? If not determined a priori, what are the criteria for terminating the process? [Humphrey-Murto 2019, Fig 1]

For peer review only

	6) What was the rationale for the number of rounds; when was the number of rounds defined [Niederberger 2020, page 6]<br><br>7) Table 3 Report the stopping [Paré 2013]<br><br>8) For delphi: if a study goes beyond two rounds, explain reason for doing so; [Waggoner 2016, page 667]
**Methods**<br>2.10 Does the study suggest anything about the details that should be reported regarding the time between rounds, if this should be prespecified in advance, or if this should be reported?	1) The time taken to complete the Delphi procedure was recorded [Boulkedid 2011, page 2]
**Methods**<br>2.11 Does the study suggest anything about details that should be reported of the names of the techniques of non-Delphi methods used to gather participants' inputs and reach consensus ?	1) Whether the meeting was held before, after, or between Delphi rounds and what the participants did during the meeting [Boulkedid 2011, page 2]
**Methods**<br>2.12 Does the study suggest anything of what or in which detail	1) What software will be used to administer the Delphi? [Humphrey-Murto 2019, fig 1]

For peer review only

should be reported regarding tool or electronic system used for Delphi? (If Delphi was used)? Or if this should be reported?	
**Methods**<br>2.13 Does the study suggest anything about how or in what level of detail the anonymity of participants (in Delphi or other methods) has to be reported? Or if this should be reported?	1) No, only that it is a limitation of this study that the quality score did not include that. So actually they feel it should be reported how anonymity was maintained [Banno 2020]<br><br>2) Describe how anonymity was defined [Chan 2019, Box 1]<br><br>3) Were responses anonymized [Gattrell 2019, table 1]<br><br>4) It suggests that conducting anonymous iterative mail or e-mail questionnaire rounds is one of the steps [p 1491]. While the authors may have assumed that readers would understand that anonymity was part of their study design, we suggest that they state this, given the variability in approaches that have been labelled as modified consensus methods. [Humphrey-Murto 2017 AMA, page 1497]<br><br>5) Describe how anonymity was maintained. Authors must clearly state how this was accomplished. It is achieved through the use of mail outs in Delphi and RAND/UCLA and private ranking in NGT. [Humphrey-Murto 2017 Med Teach, page 18]<br><br>6) How will anonymity be maintained? [Humphrey-Murto 2019, fig 1]<br><br>7) Ensure the anonymity of the participants. The anonymity of the experts was reported in virtually all of the studies [Paré 2013]
**Methods**<br>2.14 Does the study suggest anything about how to report, and in what level of detail, the feedback for panellists (in	1) Whether the experts were informed of both the response of the group and their own individual response (individual feedback) to each item. The type of feedback, which was defined as qualitative when a summary of the panel's comments was sent to each participant and quantitative when simple statistical summaries illustrating the collective opinion (e.g., central tendency and variance) were sent to each participant [page 2] After each round, each participant should be given the panel results (median, lowest, and highest ratings), the participant's response, and a summary of all comments received. These data inform each participant of his or her position relative to the rest of the group, thus assisting in decisions about replies during future Delphi rounds. [Boulkedid 2011, page 8] It has been recommended that

For peer review only

Delphi rounds or other methods) process? Or if this should be reported?	feedback should include qualitative comments and statistical measures [citation 51, Murphy 1998]. More specifically, we determined whether the experts were informed of both the response of the group and their own individual response (individual feedback) to each item [Boulkedid 2011]<br><br>2) Describe the type of feedback provided after each round [Chan 2019, Box 1]<br><br>3) Were participants' responses in each round reported back to the group, and were responses anonymized? [Gattrell 2019, Table 1]<br><br>4) Give attention to issues which guide data collection: the discovery of opinions, the process of determining the most important issues referring to the design of the initial round, and the management of opinions [Hasson 2020, page 1013]<br><br>5) Was formal feedback provided? If so, was the feedback described? [page 1493], areas that need to be improved with reporting providing participants with feedback of group ratings [Humphrey-Murto 2017 AMA, page 1494]<br><br>6) Describe the type of feedback provided after each round [page 18]. Feedback to participants can include quantitative and/or qualitative data. It also involves two types of agreement: the extent to which individual participants agree with an issue, and the extent to which participants agree with one another. Quantitative feedback may include summary statistics such as the participants' score, participants' medians, range of scores and the proportion of participants selecting each point on a scale. Participants are provided an opportunity to change their ranking, but it should be made clear that they do not need to conform. Researchers may ask the participants who are outliers to provide written justification for their choices (qualitative data) [Humphrey-Murto 2017 Med Teach]<br><br>7) What type of feedback will participants received after each round? [2019] indicates feedback between rounds should include individuals' scores for each item and the distribution of votes by participant group. Some, however, preferred to view aggregated feedback as well as feedback to individual participants [Humphrey-Murto 2019 Yes page 1042, table 1]<br><br>8) How was the feedback designed? [Niederberger 2020, table 2]<br><br>9) Citation [Schmidt, 54] recommends three relevant pieces of feedback that can be provided to experts in phase 3 in addition to mean ranks, namely, the interpretation of Kendall's W from the previous round, the percentage of experts

For peer review only

	placing each item in the top half of their list and the relevant comments that were made by the other panellists [Paré 2013, page 213]<br><br>10) They imply that it should be reported that panellist feedback was collected to inform subsequent Delphi rounds [Resemann 2018]<br><br>11) not about reporting but they state "57 % were silent about how the feedback after consensus was dealt with." suggesting that they felt it needs to be reported. [page 2] only that some reporting guidelines described the feedback information requirement, or gave the methods for feedback collection [Wang 2015, page 6]
**Methods**<br>2.15 Does the study suggest anything about how or if data synthesis/analysis should be reported (from any consensus method used and how this was calculated statistically) and in what level of detail?	1) It is important that standards and norms for prospectively defining analysis plans are needed to improve the credibility of Delphi processes for informing health research, practice, and policy [Grant 2018, page 97]<br><br>2) The methods employed need to be comprehensible; information about methods of data analysis, processing and synthesis of experts' responses to inform the subsequent survey round [Box 3] {Jünger 2017]<br>Reporting of results for each round separately is highly advisable in order to make the evolving of consensus over the rounds transparent. This includes figures showing the average group response, changes between rounds, as well as any modifications of the survey instrument such as deletion, addition or modification of survey items based on previous rounds." [Jünger 2017, item 13]<br><br>3) Detailing statistical analyses and interpretation in arriving at final agreed values [Ng 2018, item 7]<br><br>4) The statistical analyses should be reported [Paré 2013, page 211]<br><br>5) Consensus Development Panels: Statistical analysis: must be reasonable for the research question, and should be as rigorous as possible [Waggoner 2016, page 665]
**Methods**<br>2.16 Does the study suggest anything about how or if piloting should be reported and in what	1) Pilot testing with a small group of individuals is suggested before implementation [Humphrey-Murto 2017 Med Teach, page 16]<br><br>2) All material provided to the expert panel at the outset of the project and throughout the Delphi process should be carefully reviewed and piloted in advance in order to examine the effect on experts' judgements and to prevent bias. [Box 3] The methods employed need to be comprehensible; this includes information on preparatory steps (How was

For peer review only

level of detail (e.g. understanding of consensus items, platforms used, tools used)?	available evidence on the topic in question synthesised?), piloting of material and survey instruments, design of the survey instrument(s), the number and design of survey rounds, methods of data analysis, processing and synthesis of experts' responses to inform the subsequent survey round and methodological decisions taken by the research team throughout the process [Jünger 2017]<br><br>3) Pre-test task instructions and questionnaire instruments [Paré 2013]
**Methods**<br>2.17 Does the study suggest anything about how or if the role of Steering Committee members should be reported?	No data
**Methods**<br>2.18 Does the study suggest anything on what or if should be described regarding COI or funding?	1) 'Sources of funding (industry, non-industry)'as items associated with reporting quality [Banno 2019, page 2]<br><br>2) Is the funding source clearly disclosed? [table 1] Is the role of the funder clearly disclosed? [table 1] Is the funding of any external support (e.g. with the Delphi panel meeting/questionnaires, or medical writing support for the final manuscript) clearly disclosed? [Gattrell 2019]<br><br>3) "Prevention of bias. Researchers need to take measures to avoid directly or indirectly influencing the experts' judgements. If one or more members of the research team have a conflict of interest, entrusting an independent researcher with the main coordination of the Delphi study is advisable" [Jünger 2017]<br><br>4) Describing expert panel selection with eligibility criteria and including conflicts of interest [Ng 2018]
**Methods**<br>2.19 Does the study suggest anything on what should be described of how is dealt with COI of panellist (not allowed	1) No. It only deals with COI as a planning/methodological procedure, not reporting. "5. Prevention of bias. Researchers need to take measures to avoid directly or indirectly influencing the experts' judgements. If one or more members of the research team have a conflict of interest, entrusting an independent researcher with the main coordination of the Delphi study is advisable"[Jünger 2017]

For peer review only

to vote when there is COI)? Or if this should be described	

**Results**<br>3.1 Does the study suggest anything on how to report the initial evidence search (presentation of results of the literature review)?	1) No, but they suggest it should be reported [Jünger 2017]
**Results**<br>3.2 Does the study suggest anything on how to report n of studies found?	No data
**Results**<br>3.3 Does the study recommend which detail should be used when reporting panellists drop-outs (numbers and reasons)? Or if this should be reported?	1) No but it states that number the response rate for the first round dropped to 170 (66.1%). [page 1494]; areas that need improvement in reporting the number of participants after each round [page 1496] Other analyses of consensus methods research found similar poor reporting of this feature, with 7% to 39% of studies reporting response rates for all rounds of data collection [Humphrey-Murto 2017 AMA]<br><br>2) Fig 1 step 7 How will non-responders be managed, i.e. will they be excluded in subsequent rounds What response rate will be acceptable for each stakeholder group in each round? [Humphrey-Murto 2019]<br><br>3) Reporting of results for each round separately is highly advisable in order to make the evolving of consensus over the rounds transparent. This includes figures showing the average group response, changes between rounds, as well as any modifications of the survey instrument such as deletion, addition or modification of survey items based on previous rounds [Jünger 2017, Box 3]<br><br>4) Outlining participation and attrition rates for each round [Ng 2018]

For peer review only

	5) report the response rate to the initial request for participation, the size of the panel and the retention rate; [Paré 2013, page 215 3rd bullet]
**Results**<br>3.4 Does the study suggest how or if approval rates per item shared with respondents for each round should be reported in the Results section?	1) Response rate for each round [Boulkedid 2011, Table 5 on page 7]<br><br>2) Yes Box 1 report response rates and results after each round [Chan 2019]<br><br>3) Response rates for each round should be reported, presentation of total of issues generated in round 1, and presentation of results in round 2 indicating strength of support [Hasson 2000, figure 1 and page 1013]<br><br>4) Report response rates and results after each round [Humphrey-Murto 2017 Med Teach, page 18]<br><br>5) it should report response rates for all rounds [Humphrey-Murto 2019, page 1042]<br><br>6) Reporting of results for each round separately is highly advisable in order to make the evolving of consensus over the rounds transparent. This includes figures showing the average group response, changes between rounds, as well as any modifications of the survey instrument such as deletion, addition or modification of survey items based on previous rounds." [Jünger 2017, item 13]Criteria for the selection of experts and transparent information on recruitment of the expert panel, socio- demographic details including information on expertise regarding the topic in question, (non)response and response rates over the ongoing iterations should be reported". [Jünger 2017]<br><br>7) Reporting both quantitative results and textual comments for each round of analysis [Ng 2018]<br><br>8) How high was the response rate from the experts both when initially approached and also for the individual rounds [Niederberger 2020, Table 2]<br><br>9) Level of consensus should be reported [Resemann 2018]
**Results**<br>3.5 Does the study suggest anything about in which detail the items that have been dropped should	1) Were the criteria for dropping clear; are stopping criteria, other than rounds, reported [Banno 2019, item 3 and 4]<br><br>2) Were the criteria for dropping items clear? (yes, no, or not applicable) [Banno 2020, 2.6 item 3]<br><br>3) Clear criteria for dropping or combining items should also be specified based on the level of agreement or disagreement with individual items. One of the limitations of a priori specification is that certain items may fall just below the

For peer review only

be reported? (reasons e.g.) Or if this should be reported?	threshold for what is fundamentally an arbitrary cut off. In the event that items, believed to be important fell just below the threshold for inclusion in the study, the authors could consider including these items as posteriori considerations provided that sufficient justification was provided. [page 405] Suggested quality criteria: Were criteria for dropping items clear; Stopping criteria other than rounds specified? [Table 5] Were items dropped? What criteria will be used to determine which items to drop? [Diamond 2014, Table 6]<br><br>4) No, but they state Interpretation and processing of results. Consensus does not necessarily imply the correct answer or judgement; (non)consensus and stable disagreement provide informative insights and highlight differences in perspectives concerning the topic in question and Definition and attainment of consensus. It needs to be comprehensible to the reader how consensus was achieved throughout the process, including strategies to deal with non-consensus [Jünger 2017 in Box 3]<br><br>5) Were criteria defined for dropping items [Niederberger 2020, page 6]
**Results**<br>3.6 Does the study make any recommendation on how to report the collection, synthesis and use of comments from panellists? Or if this should be reported?	1) It has been recommended that feedback should include qualitative comments and statistical measures [Murphy 1998, 51]. After each round, each participant should be given the panel results (median, lowest, and highest ratings), the participant's response, and a summary of all comments received [Boulkedid 2011]<br><br>2) Describe the type of feedback provided after each round. Quantitative feedback may include summary statistics such as the participants' score, participants' medians, range of scores and the proportion of participants selecting each point on a scale. Participants are provided an opportunity to change their ranking, but it should be made clear that they do not need to conform [Humphrey-Murto 2017 Med Teach]<br><br>3) Reporting of results for each round separately is highly advisable in order to make the evolving of consensus over the rounds transparent. This includes figures showing the average group response, changes between rounds, as well as any modifications of the survey instrument such as deletion, addition or modification of survey items based on previous rounds [Jünger 2017, item 13]<br><br>4) Ask experts to justify their rankings. Have experts comment and validate consolidated list [page 210 Table 3]. Did experts consolidate the list of items; Did experts comment on and validate the list of items; Was the final number of items reported.<br>Report whether panel members had the opportunity to justify or clarify their own reasoning and to comment on the responses of the other experts as well as on the progress of the panel as a whole. [Paré 2013, page 213].

For peer review only

	Were panellists able to revise previous statements [Paré 2013]<br><br>5) No, but implied that it should be: did not report collecting panellist feedback to inform subsequent Delphi stages [Resemann 2018]
**Results**<br>3.7 Does the study suggest regarding how the final list of items (for clinical guideline or reporting guideline) should be reported? Or if this should be reported?	1) Partially. It says it should be detailed and disseminated, but it does not suggest how (in what format) it should be reported [Jünger 2017]<br><br>2) Suggests "detailing statistical analyses and interpretation in arriving at final agreed values" [Ng 2018]<br><br>3) Report final number of items [Paré 2013, page 210 Table 3]<br><br>4) No but again imply "reported the number of statements assessed." [Resemann 2018]

**Discussion**<br>4.1 Does the paper suggest anything about reporting the limitations and strengths of the study and how? Or if this should be reported?	1) Address potential methodological issues (e.g lack of consensus) or limitations in the discussion (e.g. low response rate) [Chan 2019, Box 1]<br><br>2) Interpretation of consensus gained/not gained [Hasson 2020, page 1009]<br><br>3) In the discussion the authors should address issues that may have impacted the results such as poor response rates between rounds, lack of participation from a select group or geographic region, or lack of consensus. [Humphrey-Murto 2017 Med Teach, page 18]<br><br>4) Methodological issues should be reported [Humphrey-Murto 2019, figure 1]<br><br>5) Reporting should include a critical reflection of potential limitations and their impact of the resulting guidance". [Jünger 2017]
**Discussion**<br>4.2 Does the paper suggest anything about what or in	1) Page 5: is considered a good measure if it meets criteria including reliability, sensitivity, specificity, and feasibility (or applicability) [20,31]. The common use of these characteristics can facilitate acceptance and implementation of indicators developed [Boulkedid 2011]

For peer review only

which detail the applicability generalisability, and reproducibility of the study should be reported? Or if this should be reported?	2) The conclusions should adequately reflect the outcomes of the Delphi study with a view to the scope and applicability of the resulting practice guidance. [Jünger 2017, item 15]<br><br>3) It is also necessary to discuss the critical and rationalistic criteria for the validity and reliability of the studies and the more constructivist characteristics of credibility, transparency, and transferability. [Niederberger 2020, page 8]

**5.1 Any other item proposed by the paper that is not captured in other columns?**	1) Were criteria for dropping items clear? Are stopping criteria, other than rounds, specified [Banno 2019]<br><br>2) Differences between the protocol and the article [Banno 2020, 2.9]<br><br>3) Geographic scope of the survey [page 2]. Main methods used to send the questionnaires (e.g., mail, E-mail, or fax). [Boulkedid 2011, page 7]<br>The formulation of the questionnaire items (e.g., open questions, rating of quality indicators, or both). [Boulkedid 2011]<br>Whether the quality indicators were rated (in which case, we recorded the minimum and maximum values on the rating scale). [Boulkedid 2011]<br>A flow chart of quality indicators (figure showing the output and input indicators at each round) and/or for a written description of indicator flow. [Boulkedid 2011, page 3]<br>Quality indicators used in the first round versus the end of the last round. [Boulkedid 2011, page 3]<br>Availability of the questionnaires in the article itself or in an appendix [Boulkedid 2011, page 3]<br>Whether selection criteria changed between rounds [Boulkedid 2011, page 5]<br>Whether panelists were able to make comments. [Boulkedid 2011, page 6]<br>Whether there was a meeting; at what stage it took place and how people participated [Boulkedid 2011]<br>Response rate for each round [Boulkedid 2011, page 7]<br>preparation in advance of starting Delphi (outcome indicators, structure indicators, process indicators) [Boulkedid 2011, In appendix S1, item 1]<br>METHODS<br>We evaluated the relationship between the response rate and the use of specific methods to encourage the experts to respond (e.g., stamped addressed envelope for returning the questionnaire and financial compensation). Also on maybe we should add item regarding encouragement of participants [Boulkedid 2011, page 2, page 5 right column]<br>Geographic scope of Delphi consensus procedure [Boulkedid 2011,item 20 of appendix and table 5]<br>Question format (open questions, rating scale?) Also in table 5 how were questions formulated? [Boulkedid 2011, item 24

For peer review only

	appendix]<br>Rating scale [Boulkedid 2011, item 25]<br>Methods used to send questionnaire (email fax, mail) [Boulkedid 2011, table 5]<br>Time to complete questionnaire reporting of differences in response rate in rounds [Boulkedid 2011]<br>Number of rounds necessary to reach consensus [Boulkedid 2011]<br>Duration of the procedure [Boulkedid 2011]<br>Is questionnaire added as appendix? [Boulkedid 2011]<br>For Discussion: Validity [Boulkedid 2011]<br><br>4) Outline each step of the process. If modifications were made, provide a rationale for your choices. [Chan 2019]<br>Describe the selection and preparation of the scientific evidence for the participants. [Chan 2019]<br>Include a description of the facilitator's credentials. [Chan 2019]<br>What background material was provided to participants. [Chan 2019]<br>What formal feedback of group rating was shared between rounds [Chan 2019]<br><br>5) Specify stopping criteria in the absence of consensus [Diamond 2014]<br><br>6) Were the questions formulated or validated by an expert panellist [Gattrell 2019]<br><br>7) Researchers conducting consensus-oriented Delphi processes should prospectively and completely register the intended procedure for identifying which items reach consensus. [Grant 2018]<br>The analysis procedure for determining consensus for Delphi processes should be chosen a priori ideally before starting the first round but at the very latest before completing data collection to improve the validity of findings. [Grant 2018]<br>Health researchers conducting consensus-oriented Delphi processes should commit themselves in advance to an analytic procedure for determining which items reach consensus before they see the actual data (or, ideally, before they even collect the data). [Grant 2018]<br>Registrations should be in a publicly available and independently controlled platform that time-stamps entries [Grant 2018]<br><br>8) "Copy of each round questionnaire illustrated" [Hasson 2020]<br>statistical interpretation for the reader [Hasson 2020]<br>appendices to include the questionnaires [Hasson 2020]<br>For Discussion interpretations of consensus gained/not gained reliability and validity [Hasson 2020]

For peer review only

	9) *Page 1493(2) Was background information provided to the participants? pg 1496 areas appeared particularly problematic and were often left out or poorly described: providing background information to participants<br>AND so a clear description of what information was provided and in what format is important<br>* (3) Was the consensus method used for item generation, ranking, or both?<br>* (11) Was consensus forced?<br>Was mail/e-mail polling or face-to-face questioning used? [Humphrey-Murto 2017 AMA]<br><br>10) Outline each step of the process: if modifications were made, provide a rationale for the choices made. Providing justification for the choices made will also add credibility. [Humphrey-Murto 2017 Med Teach]<br><br>11) Background provided to participants, what is level of detail provided [Humphrey-Murto 2019]<br>Figure 1 clear outline of the overall process involved and where Delphi fits [Humphrey-Murto 2019, figure 1]<br>How sample size is determined of participants [Humphrey-Murto 2019, figure 1]<br><br>12) Any modifications should be justified by a rationale and be applied systematically and rigorously [Jünger 2017, Box 3]<br>All material provided to the expert panel at the outset of the project and throughout the Delphi process should be carefully reviewed and piloted in advance in order to examine the effect on experts' judgements and to prevent bias [Jünger 2017]<br>It is recommended to have the final draft of the resulting guidance on best practice in palliative care reviewed and approved by an external board or authority before publication and dissemination [Jünger 2017, Box 3]<br>information about methodological decisions taken by the research team throughout the process Jünger 2017, Box 3]<br>Flow chart to illustrate the stages of the Delphi process, including a preparatory phase, the actual Delphi rounds, interim steps of data processing and analysis, and concluding steps [Jünger 2017, Box 3]<br>Publication and dissemination [Jünger 2017, Box 3]<br><br>13) Item 2-4 and 9 appending revised questionnaires [Ng 2018]<br><br>14) Specific definition of underlying Delphi technique (or as I thought it is important to define exactly what method is used, especially if a modified method is used this needs to be very clear [Niederberger 2020]<br>What role did the stability of the answers play? [Niederberger 2020, table 2]<br>Questionnaire and scale development How were the questionnaires and the specific items for a Delphi technique

For peer review only

	developed? [Niederberger 2020]<br>Nevertheless, it is important to precisely describe, justify, and methodologically reflect on any modifications [Niederberger 2020]<br>How were the questionnaires and the specific items for a Delphi technique developed? [Niederberger 2020, Table 2]<br>Were items identified from empirical analyses such as qualitative interviews or focus groups that were completed in advance or were taken from existing guidelines. [Niederberger 2020, Complementary AND page 6<br>Was the first (qualitative) round of questions in the Delphi process used to generate the items for a standardized questionnaire. [Niederberger 2020, Complementary AND page 6]<br><br>15) Was the final number of items reported [Paré 2013, Table 3] Were items randomly ordered [Paré 2013, Table 3]<br><br>16) Describe the rating scales used [Resemann 2018] the number of statements assessed should be reported [Resemann 2018]<br><br>17) For nominal group process, the research question used to prompt the panel must be clear and concise to obtain valid suggestions from panel members. [Waggoner 2016, page 665] The heterogeneity should be reported [Waggoner 2016, page 665] Evaluation of reliability [Waggoner 2016, page 665]<br><br>18) Meeting attendance; format (e.g. face-to-face); agenda preparation; materials sent to participants prior to meeting; duration of meeting [Wang 2015, page 5] Flow diagram [Wang 2015, page 3] Should we add something regarding other consensus methods including an item regarding face to face meetings? [Wang 2015, page 5]
**5.2 Any other item not proposed by the paper, but you think that could be added (not fitting the categories above)?**	1) Are stopping criteria, other than rounds, specified? [Banno 2019, page 2]<br><br>2) Information letter explaining the method and the reasons their participation to the whole process would be necessary, as well as a form for collecting their consent to complete the entire Delphi process. [Boulkedid 2011]<br><br>3) "Round 1: presentation of total number of issues generated" [Hasson 2020]<br><br>4) This paper was "pointing fingers", showing what was wrong, without suggesting solutions. However, we can be inspired by the critics to build the following list of items: 1) Purpose of the consensus study<br>Whether a literature review was done to support the selection of items [Humphrey-Murto 2017 AMA]<br><br>5) Length of the background provided [Humphrey-Murto 2019]

For peer review only

	Purpose of study: outcome/diagnosis/intervention? [Humphrey-Murto 2019]

**Examples of text with well reported methods/results (for E&E document) - write NA if none was cited or found by you**	1) Page 7 Table 5 [Boulkedid 2011]<br>2) Box 1 [Chan 2019]<br>3) Might have a look at table 6 [Diamond 2014]<br>4) Table 1 [Gattrell 2019]<br>5) Parts of Fig 1 and checklist page 1013 [Hasson 2020]<br>6) Table 1 lists "exemplary publications" for nominal group process, consensus development panel and Delphi technique Page 667 references studies that were "Very descriptive" of the statistical techniques used. [Waggoner 2016]
**Additional comments from assessor**	1) Limited value; protocol for Banno 2020 [Banno 2019]<br>2) Of limited use. The authors developed a 4-point quality score that they applied to Delphi publications [Banno 2020]<br>3) Excellent resource [Boulkedid 2011]<br>4) Focusses on defining consensus [Diamond 2014]<br>5) Congress poster only [Gattrell 2019]<br>6) Study used RAND's ExpertLens as the Delphi platform [Grant 2018]<br>7) 1497: The lack of consensus on consensus methods makes it imperative that researchers provide clear and detailed reporting of the methods they used and that they justify these choices. [Humphrey-Murto 2017]

For peer review only

	8) Page 1044 A suggestion to improv uniformity is to use a software program that provides structure and help with reporting all relevant outcomes (e.g. DelphiManager, http://comet-initiative.org/delphimanager/) [Humphrey-Murto 2019]<br><br>9) Very informative [Jünger 2017]<br><br>10) The study focusses on information systems. Arguably, this is not within the inclusion criteria for the search [Paré 2013]<br><br>11) Review covers nominal group process, consensus development panel and Delphi technique [Waggoner 2016]<br><br>12) Study looked at the reporting quality of reporting guidelines [Wang 2015]

For peer review only

ACcurate COnsensus Reporting Document (ACCORD): Summary of extracted data from literature search

Table of Contents

1. Background 2

2. Methods 3

3. Results 9

4. Discussion 11

5. Additional topics 12

References 14

For peer review only

For peer review only - http://bmjopen.bmj.com/site/about/guidelines.xhtml

Section: Background

1. Background

Data extraction question	Articles	Checklist item(s) with brief explanation
1.1. Does the study suggest anything about how or if consensus papers should report the context or rationale for choosing a consensus method over other methods?	Hasson F, *et al. J Adv Nurs* 2000[1]<br>Humphrey-Murto S, *et al. Med Teach* 2017[2]<br>Humphrey-Murto S, *et al. J Rheumatol* 2019[3]<br>Jünger S, *et al. Palliat Med* 2017[4]	State the rationale for use of consensus method over other options.<br>*Should consider other consensus methods as well as other methodology types.*
1.2. Does the study suggest anything about how/what or if consensus papers should report the objectives of the consensus exercise?	Hasson F, *et al. J Adv Nurs* 2000[1]<br>Humphrey-Murto S, *et al. Med Teach* 2017[2]<br>Jünger S, *et al. Palliat Med* 2017[4]<br>Boulkedid R, *et al. PLoS One* 2011[5]<br>Chan TM, *et al. CJEM* 2019[6]<br>Diamond IR, *et al. J Clin Epidemiol* 2014[7]	Clearly define study objectives.<br>*Could include presentation of group consensus, or just to quantify the level of agreement.*

For peer review only

Section: Methods

2. Methods

Data extraction question	Articles	Checklist item(s) with brief explanation
2.1. Does the study suggest anything about how/what or if consensus papers should report regarding:<br><br>A literature search/strategy?	Hasson F, *et al. J Adv Nurs* 2000[1]<br>Humphrey-Murto S, *et al. Med Teach* 2017[2]<br>Chan TM, *et al. CJEM* 2019[6]<br>Humphrey-Murto S, *et al. Acad Med* 2017[8]<br>Resemann HK, *et al. Curr Med Res Opin* 2018[9]	A) Describe the strategy for reviewing the existing scientific evidence that informed the study.<br>*If no existing literature is available, the extent of the search should be described.*<br>B) Describe how existing scientific evidence will be provided to the participants.<br>*If different participant groups are involved, it should be stated which information will be provided to which group.*
2.2. Does the study suggest anything about how/what or if consensus papers should report regarding:<br><br>Inclusion and exclusion criteria for the literature search?	Boulkedid R, *et al. PLoS One* 2011[5]<br>Chan TM, *et al. CJEM* 2019[6]<br>Paré G, *et al. Inf Manag* 2013[10]	Describe the process of the literature search.<br>*Should include inclusion and exclusion criteria, and state whether these were prespecified.*
2.3. Does the study suggest anything of what or if consensus report should report on panel composition, n of participants, expertise, origin? Prespecified?	Hasson F, *et al. J Adv Nurs* 2000[1]<br>Humphrey-Murto S, *et al. Med Teach* 2017[2]<br>Humphrey-Murto S, *et al. J Rheumatol* 2019[3]<br>Jünger S, *et al. Palliat Med* 2017[4]<br>Boulkedid R, *et al. PLoS One* 2011[5]<br>Chan TM, *et al. CJEM* 2019[6]<br>Diamond IR, *et al. J Clin Epidemiol* 2014[7]<br>Humphrey-Murto S, *et al. Acad Med* 2017[8]<br>Paré G, *et al. Inf Manag* 2013[10]<br>Banno M, *et al. J Clin Epidemiol* 2019[11]<br>Banno M, *et al. J Clin Epidemiol* 2020[12]<br>Gattrell WT, *et al. Curr Med Res Opin* 2019[13]	A) Describe the structure of the study's participants.<br>*Should describe inclusion of a Chair/Co-chairs, steering committee, and subgroups, if applicable.*<br>B) Explain how panel participants were selected.<br>*Should state who was responsible for panellist selection, the selection criteria applied, the justification for choosing panellist numbers and selection criteria, and whether criteria were prespecified.*

For peer review only

Section: Methods

Data extraction question	Articles	Checklist item(s) with brief explanation
	Ng J. *Value Health* 2018[14]<br>Niederberger M, *et al. Front Public Health* 2020[15]<br>Waggoner J, *et al. Acad Med* 2016[16]<br>Wang X, *et al. BMC Med Res Methodol* 2015[17]	*C)* Describe the composition of the panel. *Should include number of participants at all stages of the process, sociodemographics (e.g. age, sex, specialty, type and duration of relevant experience). Should also describe panel subgroups, if relevant.*<br>D) Describe the expertise of the panel. *Should include the definition of "expert" and description of any public or patients involved.*<br>E) Describe the facilitator(s), if used. *Should include type and duration of relevant experience, and the role played in the process.*
2.4. Does the study suggest anything of how or if PPI (public patient involvement) activity should be reported	No data	Describe the role and involvement of any public or patients.<br>*Should detail the stage(s) at which they were involved, and their roles and contributions.*
2.5. Does the study suggest anything about what or if consensus papers should report regarding panel recruitment strategies, invitations? Any level of detail specified?	Jünger S, *et al. Palliat Med* 2017[4]<br>Boulkedid R, *et al. PLoS One* 2011[5]<br>Paré G, *et al. Inf Manag* 2013[10]<br>Waggoner J, *et al. Acad Med* 2016[16]	Describe how the panel members were recruited.<br>*Could include communication/advertisement method(s) and locations.*
2.6. Does the study suggest how or if consensus papers should report the consensus criteria/threshold (or the level of agreement considered to reach consensus)?	Hasson F, *et al. J Adv Nurs* 2000[1]<br>Humphrey-Murto S, *et al. Med Teach* 2017[2]<br>Humphrey-Murto S, *et al. J Rheumatol* 2019[3]<br>Jünger S, *et al. Palliat Med* 2017[4]<br>Boulkedid R, *et al. PLoS One* 2011[5]<br>Chan TM, *et al. CJEM* 2019[6]<br>Diamond IR, *et al. J Clin Epidemiol* 2014[7]<br>Humphrey-Murto S, *et al. Acad Med* 2017[8]<br>Gattrell WT, *et al. Curr Med Res Opin* 2019[13]	A) Define the consensus measure to be used. *Could include percentage agreement, units of central tendency (e.g. median), a categorical rating (e.g. Agree/Strongly agree) or a combination of percent agreement within a certain range.*<br>B) State the threshold for the group achieving consensus. *Should include whether the threshold was*

For peer review only - http://bmjopen.bmj.com/site/about/guidelines.xhtml

Section: Methods

Data extraction question	Articles	Checklist item(s) with brief explanation
	Niederberger M, *et al. Front Public Health* 2020[15]<br>Waggoner J, *et al. Acad Med* 2016[16]<br>Wang X, *et al. BMC Med Res Methodol* 2015[17]<br>Grant S, *et al. J Clin Epidemiol* 2018[18]	*pre-defined and highlight any threshold variation between rounds, with explanation for the change. If the intention is to quantify the degree of consensus but not to use consensus as a stop criterion for the study, this should be stated.*
2.7. Does the study suggest how or if consensus papers should report how decision of approval of an item will be made?	Hasson F, *et al. J Adv Nurs* 2000[1]<br>Jünger S, *et al. Palliat Med* 2017[4]<br>Boulkedid R, *et al. PLoS One* 2011[5]	Explain how final consensus was reached.<br>*Should describe the evolution of themes between voting rounds, if applicable.*
2.8. Does the study suggest anything about what level of detail should be reported regarding the number of Delphi rounds or if this should be reported?	Hasson F, *et al. J Adv Nurs* 2000[1]<br>Humphrey-Murto S, *et al. Med Teach* 2017[2]<br>Jünger S, *et al. Palliat Med* 2017[4]<br>Chan TM, *et al. CJEM* 2019[6]<br>Diamond IR, *et al. J Clin Epidemiol* 2014[7]<br>Humphrey-Murto S, *et al. Acad Med* 2017[8]<br>Banno M, *et al. J Clin Epidemiol* 2019[11]<br>Banno M, *et al. J Clin Epidemiol* 2020[12]<br>Niederberger M, *et al. Front Public Health* 2020[15]<br>Waggoner J, *et al. Acad Med* 2016[16]	State how many voting rounds were conducted.<br>*Should include whether the number of rounds was prespecified, and whether this was an absolute or a maximum. If the maximum was exceeded, should explain the reasoning for doing so.*
2.9. Does the study suggest anything about what level of detail should be reported regarding the criteria used for defining the number of rounds? (why 2-3 or more e.g.) or if this should be reported?	Humphrey-Murto S, *et al. Med Teach* 2017[2]<br>Humphrey-Murto S, *et al. J Rheumatol* 2019[3]<br>Chan TM, *et al. CJEM* 2019[6]<br>Humphrey-Murto S, *et al. Acad Med* 2017[8]<br>Paré G, *et al. Inf Manag* 2013[10]<br>Banno M, *et al. J Clin Epidemiol* 2020[12]<br>Niederberger M, *et al. Front Public Health* 2020[15]<br>Waggoner J, *et al. Acad Med* 2016[16]	Explain the rationale for choosing the number of voting rounds.<br>*Should also describe the stop criteria, if used, and whether these were prespecified.*
2.10. Does the study suggest anything about the details that should be reported regarding the time between rounds, if this should be	Boulkedid R, *et al. PLoS One* 2011[5]	Describe the time period between voting rounds.<br>*Should include whether the period was*

Section: Methods

Data extraction question	Articles	Checklist item(s) with brief explanation
prespecified in advance, or if this should be reported?		*prespecified and highlight differences between inter-round periods, if applicable.*
2.11. Does the study suggest anything about details that should be reported of the names of the techniques of non-Delphi methods used to gather participants' inputs and reach consensus?	Boulkedid R, *et al. PLoS One* 2011[5]<br>Waggoner J, *et al. Acad Med* 2016[16]	Describe any additional methods used alongside the consensus process.<br>*Should include all that were used, e.g. a self-administered questionnaire combined with a group meeting. Should also explain how the consensus process fitted into the overall study methodology.*
2.12. Does the study suggest anything of what or in which detail should be reported regarding tool or electronic system used for Delphi? (If Delphi was used)? Or if this should be reported?	Humphrey-Murto S, *et al. J Rheumatol* 2019[3]	Describe any tools used to administer the voting.<br>*Could detail electronic platforms, if used.*
2.13. Does the study suggest anything about how or in what level of detail the anonymity of participants (in Delphi or other methods) has to be reported? Or if this should be reported?	Humphrey-Murto S, *et al. Med Teach* 2017[2]<br>Humphrey-Murto S, *et al. J Rheumatol* 2019[3]<br>Chan TM, *et al. CJEM* 2019[6]<br>Humphrey-Murto S, *et al. Acad Med* 2017[8]<br>Paré G, *et al. Inf Manag* 2013[10]<br>Banno M, *et al. J Clin Epidemiol* 2020[12]<br>Gattrell WT, *et al. Curr Med Res Opin* 2019[13]	Detail how anonymity of voters was maintained.<br>*Could involve use of mail-outs in a standard Delphi procedure, blinding on an electronic platform, or private ranking in the NGT.*
2.14. Does the study suggest anything about how to report, and in what level of detail, the feedback for panellists (in Delphi rounds or other methods) process? Or if this should be reported?	Hasson F, *et al. J Adv Nurs* 2000[1]<br>Humphrey-Murto S, *et al. Med Teach* 2017[2]<br>Humphrey-Murto S, *et al. J Rheumatol* 2019[3]<br>Boulkedid R, *et al. PLoS One* 2011[5]<br>Chan TM, *et al. CJEM* 2019[6]<br>Humphrey-Murto S, *et al. Acad Med* 2017[8]<br>Resemann HK, *et al. Curr Med Res Opin* 2018[9]<br>Paré G, *et al. Inf Manag* 2013[10]<br>Gattrell WT, *et al. Curr Med Res Opin* 2019[13]<br>Niederberger M, *et al. Front Public Health* 2020[15]	Explain how voting feedback was provided to panellists at the end of each round.<br>*Could include summaries of group voting and/or their own individual responses. Should state whether feedback will be quantitative and/or qualitative, and whether it will be anonymised. If no feedback was provided, this should be stated.*

For peer review only - http://bmjopen.bmj.com/site/about/guidelines.xhtml

Section: Methods

Data extraction question	Articles	Checklist item(s) with brief explanation
	Wang X, *et al. BMC Med Res Methodol* 2015[17]	
2.15. Does the study suggest anything about how or if data synthesis/analysis should be reported (from any consensus method used and how this was calculated statistically) and in what level of detail?	Jünger S, *et al. Palliat Med* 2017[4]<br>Paré G, *et al. Inf Manag* 2013[10]<br>Ng J. *Value Health* 2018[14]<br>Waggoner J, *et al. Acad Med* 2016[16]<br>Grant S, *et al. J Clin Epidemiol* 2018[18]	Detail methods used to process responses after each voting round.<br>*Could include statistical analysis methods, if used.*
2.16. Does the study suggest anything about how or if piloting should be reported and in what level of detail (e.g. understanding of consensus items, platforms used, tools used)?	Humphrey-Murto S, *et al. Med Teach* 2017[2]<br>Jünger S, *et al. Palliat Med* 2017[4]<br>Paré G, *et al. Inf Manag* 2013[10]	Describe any piloting of the study materials and/or survey instruments.<br>*Should include the number of individuals in the pilot group and the rationale for their selection. Should also explain any changes made as a result of the pilot. If no pilot was conducted, this should be stated.*
2.17. Does the study suggest anything about how or if the role of Steering Committee members should be reported?	No data	Describe the role(s) of the Steering Committee in the process.<br>*Should also detail the involvement of the Chair/Co-chairs, subgroups, or individual members at relevant stages of the process, if different from the group as a whole.*
2.18. Does the study suggest anything on what or if should be described regarding COI or funding?	Jünger S, *et al. Palliat Med* 2017[4]<br>Banno M, *et al. J Clin Epidemiol* 2019[11]<br>Gattrell WT, *et al. Curr Med Res Opin* 2019[13]<br>Ng J. *Value Health* 2018[14]	A) Disclose any COI of the panellists<br>*Should specify COI of each participant in the panel.*<br>B) Disclose any funding received and the role of the funder.<br>*Should specify the role of the funding source(s), e.g. involvement in the study concept/design, participation of the Steering Committee, for conducting the consensus process, medical writing support for its reporting.*

For peer review only

Section: Methods

Data extraction question	Articles	Checklist item(s) with brief explanation
2.19. Does the study suggest anything on what should be described of how is dealt with COI of panellist (not allowed to vote when there is COI)? Or if this should be described	Jünger S, *et al*. *Palliat Med* 2017[4]	Describe measures taken to avoid influence by any conflicts of interest (COI).<br>*Should include disclosure of COI and how this was accounted for in the methodology, e.g. by limiting voting in case of a specific COI, adjudication by an independent researcher.*

For peer review only

Section: Results

3. Results

Data extraction question	Articles	Checklist item(s) with brief explanation
3.1. Does the study suggest anything on how to report the initial evidence search (presentation of results of the literature review)?	Jünger S, *et al*. *Palliat Med* 2017[4]	Describe how existing scientific evidence was provided to the participants.<br>*Should include relevant specifics of the literature search, e.g. n of studies reported, to provide relevant context for the results. If different participant groups were involved, it should be stated which information was provided to which group.*
3.2. Does the study suggest anything on how to report n of studies found?	No data	Describe the results of the search and number of included studies.
3.3. Does the study recommend which detail should be used when reporting panellists drop-outs (numbers and reasons)? Or if this should be reported?	Humphrey-Murto S, *et al. J Rheumatol* 2019[3]<br>Jünger S, *et al*. *Palliat Med* 2017[4]<br>Humphrey-Murto S, *et al. Acad Med* 2017[8]<br>Paré G, *et al. Inf Manag* 2013[10]<br>Ng J. *Value Health* 2018[14]	A) State the response rates for each voting round.<br>*Should specify n as well as percent, or otherwise indicate attrition/retention rates.*<br>B) State the reasons cited for voter drop-outs at each stage of the process.<br>*Could be provided as an aggregated summary or as individual responses. If this information was not collected, this should be stated.*<br>C) Describe measures undertaken to maintain acceptable response rates.<br>*If threshold rates differ between stakeholder groups, these should be described with explanation.*

For peer review only - http://bmjopen.bmj.com/site/about/guidelines.xhtml

Section: Results

Data extraction question	Articles	Checklist item(s) with brief explanation
3.4. Does the study suggest how or if approval rates per item shared with respondents for each round should be reported in the Results section?	Hasson F, *et al. J Adv Nurs* 2000[1]<br>Humphrey-Murto S, *et al. Med Teach* 2017[2]<br>Humphrey-Murto S, *et al. J Rheumatol* 2019[3]<br>Jünger S, *et al. Palliat Med* 2017[4]<br>Boulkedid R, *et al. PLoS One* 2011[5]<br>Chan TM, *et al. CJEM* 2019[6]<br>Resemann HK, *et al. Curr Med Res Opin* 2018[9]<br>Ng J. *Value Health* 2018[14]<br>Niederberger M, *et al. Front Public Health* 2020[15]	Describe which results that were shared with respondents after each voting round were reported in the final manuscript.<br>*Could include response rates, the type of information presented, summaries of group voting and/or individual responses. If this information is not provided, this should be stated together with the rationale.*
3.5. Does the study suggest anything about in which detail the items that have been dropped should be reported? (reasons e.g.) Or if this should be reported?	Jünger S, *et al. Palliat Med* 2017[4]<br>Diamond IR, *et al. J Clin Epidemiol* 2014[7]<br>Banno M, *et al. J Clin Epidemiol* 2019[11]<br>Banno M, *et al. J Clin Epidemiol* 2020[12]<br>Niederberger M, *et al. Front Public Health* 2020[15]	A) List any voting items that were dropped.<br>B) Explain the rationale for dropping any voting items.<br>*Should state whether the criteria for dropping any items were prespecified.*
3.6. Does the study make any recommendation on how to report the collection, synthesis and use of comments from panellists? Or if this should be reported?	Humphrey-Murto S, *et al. Med Teach* 2017[2]<br>Jünger S, *et al. Palliat Med* 2017[4]<br>Boulkedid R, *et al. PLoS One* 2011[5]<br>Resemann HK, *et al. Curr Med Res Opin* 2018[9]<br>Paré G, *et al. Inf Manag* 2013[10]	Describe how responses were processed prior to reporting.<br>*Should describe methods by which responses were analysed, aggregated or summarised, include whether any statements were revised between voting rounds, and state by whom the information was processed.*
3.7. Does the study suggest regarding how the final list of items (for clinical guideline or reporting guideline) should be reported? Or if this should be reported?	Jünger S, *et al. Palliat Med* 2017[4]<br>Resemann HK, *et al. Curr Med Res Opin* 2018[9]<br>Paré G, *et al. Inf Manag* 2013[10]<br>Ng J. *Value Health* 2018[14]	Report the final outcomes.<br>*Could be quantitative (e.g. summary statistics, score means, medians and/or ranges) and/or qualitative (e.g. aggregated themes from comments). Should be clear, accurately represent the consensus methodology used, and relevant to the field.*

For peer review only

Section: Discussion

4. Discussion

Data extraction question	Articles	Checklist item(s) with brief explanation
4.1. Does the paper suggest anything about reporting the limitations and strengths of the study and how? Or if this should be reported?	Hasson F, *et al. J Adv Nurs* 2000[1]<br>Humphrey-Murto S, *et al. Med Teach* 2017[2]<br>Humphrey-Murto S, *et al. J Rheumatol* 2019[3]<br>Jünger S, *et al. Palliat Med* 2017[4]<br>Chan TM, *et al. CJEM* 2019[6]	Discuss the study's methodological strengths and limitations.<br>*Should address issues that may impact results, e.g. response rates or representation.*
4.2. Does the paper suggest anything about what or in which detail the applicability generalisability, and reproducibility of the study should be reported? Or if this should be reported?	Jünger S, *et al. Palliat Med* 2017[4]<br>Boulkedid R, *et al. PLoS One* 2011[5]<br>Niederberger M, *et al. Front Public Health* 2020[15]	A) Discuss the reliability of the study.<br>B) Discuss the sensitivity of the study.<br>C) Discuss the specificity of the study.<br>D) Discuss the applicability of the study.<br>E) Discuss the validity of the study.

For peer review only - http://bmjopen.bmj.com/site/about/guidelines.xhtml

Section: Additional topics

5. Additional topics

Data extraction question: Any other item proposed by the paper that is not captured in previous sections?

Articles	Checklist item(s) with brief explanation
Humphrey-Murto S, *et al. Med Teach* 2017[3]<br>Jünger S, *et al. Palliat Med* 2017[4]<br>Boulkedid R, *et al. PLoS One* 2011[5]<br>Paré G, *et al. Inf Manag* 2013[10]<br>Banno M, *et al. J Clin Epidemiol* 2020[12]	Explain any deviations from the planned protocol.<br>*Should include any affected stages, including but not limited to change in panel number or composition, number of voting rounds, stopping criteria, statistical plan, reporting of outcomes.*
Boulkedid R, *et al. PLoS One* 2011[5]<br>Resemann HK, *et al. Curr Med Res Opin* 2018[9]	Describe the formulation of questions.<br>*Should include the type of questions, e.g. open questions, numerical rating, level of agreement rating. If rating questions were used, the scale range should be stated, and whether respondents were able to leave additional comments after rating items.*
Boulkedid R, *et al. PLoS One* 2011[5]<br>Wang X, *et al. BMC Med Res Methodol* 2015[17]	Describe any group meetings that were held.<br>*Should state at what stage the meeting took place, objectives/purpose, format (e.g. face-to-face or virtual), pre-read materials shared, attendance, location, duration, and how individuals participated.*
Hasson F, *et al. J Adv Nurs* 2000[1]<br>Boulkedid R, *et al. PLoS One* 2011[5]<br>Ng J. *Value Health* 2018[14]	List any items included in the appendix accompanying the main report.<br>*Could include e.g. full voting questions from each round with response rates, or information provided to the panel as pre-reads or to summarise voting rounds.*
Boulkedid R, *et al. PLoS One* 2011[5]	State how the survey was presented to participants.<br>*For example, as hard copy or via digital platform; could include description of email or mailing process. Should describe any randomisation procedures for questions, if used. If questions were not randomised, this should be stated.*
Boulkedid R, *et al. PLoS One* 2011[5]	Describe incentives for encouraging responses.<br>*Should list any specific methods, e.g. paid return postage for the questionnaire or financial compensation.*
Boulkedid R, *et al. PLoS One* 2011[5]	State the period in which the process was conducted.
Grant S, *et al. J Clin Epidemiol* 2018[18]	Describe any prospective registrations for the consensus process.

For peer review only - http://bmjopen.bmj.com/site/about/guidelines.xhtml

Section: Additional topics

**Articles**	**Checklist item(s) with brief explanation**
	Should include the platform on which it was registered and a link, if applicable. If the process was not registered, this should be stated.
Jünger S, *et al*. *Palliat Med* 2017[4]	Describe any external peer review prior to publication.<br>*Should name the authority, state the rationale for their review, and describe any modifications made as a result of their review.*
Humphrey-Murto S, *et al. Med Teach* 2017[3]<br>Jünger S, *et al*. *Palliat Med* 2017[4]	Describe the overall process using a flow chart or diagram.
Paré G, *et al. Inf Manag* 2013[10]<br>Niederberger M, *et al. Front Public Health* 2020[15]	Explain how the initial voting items in the consensus were developed.<br>*Could describe e.g. development from empirical analyses, qualitative interviews, advance focus groups, brainstorming, or existing guidelines. Should state who consolidated the information and developed the voting items.*
Boulkedid R, *et al. PLoS One* 2011[5]	Describe the procedure for collecting participants' consent to complete the full consensus process.<br>*Could briefly describe any forms used and how the data were collected and stored.*

For peer review only

Section: References

References

1. Hasson F, Keeney S, McKenna H. Research guidelines for the Delphi survey technique. *J Adv Nurs* 2000;32:1008-15. doi: 10.1046/j.1365-2648.2000.t01-1-01567.x.
2. Humphrey-Murto S, Varpio L, Gonsalves C, et al. Using consensus group methods such as Delphi and Nominal Group in medical education research. *Med Teach* 2017;39:14-9. doi: 10.1080/0142159X.2017.1245856.
3. Humphrey-Murto S, Crew R, Shea B, et al. Consensus Building in OMERACT: Recommendations for Use of the Delphi for Core Outcome Set Development. *J Rheumatol* 2019;46:1041-6. doi: 10.3899/jrheum.181094.
4. Jünger S, Payne SA, Brine J, et al. Guidance on Conducting and REporting DElphi Studies (CREDES) in palliative care: Recommendations based on a methodological systematic review. *Palliat Med* 2017;31(8):684-706. doi: 10.1177/0269216317690685.
5. Boulkedid R, Abdoul H, Loustau M, et al. Using and Reporting the Delphi Method for Selecting Healthcare Quality Indicators: A Systematic Review. *PLoS One* 2011;6:e20476. doi: 10.1371/journal.pone.0020476.
6. Chan TM, Yarris LM, Humphrey-Murto S. Delving into Delphis. *CJEM* 2019;21:167-9. doi: 10.1017/cem.2019.3.
7. Diamond IR, Grant RC, Feldman BM, et al. Defining consensus: A systematic review recommends methodologic criteria for reporting of Delphi studies. *J Clin Epidemiol* 2014;67:401-9. doi: 10.1016/j.jclinepi.2013.12.002.
8. Humphrey-Murto S, Varpio L, Wood TJ, et al. The Use of the Delphi and Other Consensus Group Methods in Medical Education Research: A Review. *Acad Med* 2017;92:1491-8. doi: 10.1097/ACM.0000000000001812.
9. Resemann HK, Clements S, Griffiths A, et al. Reporting of Delphi methods to achieve consensus on guidelines in rare diseases. *Curr Med Res Opin* 2018;34 (Suppl 1):37.
10. Paré G, Cameron AF, Poba-Nzaou P, et al. A systematic assessment of rigor in information systems ranking-type Delphi studies. *Inf Manag* 2013;50:207-17 doi: 10.1016/j.im.2013.03.003.
11. Banno M, Tsujimoto Y, Kataoka Y. Reporting quality of the Delphi technique in reporting guidelines: a protocol for a systematic analysis of the EQUATOR Network Library. *J Clin Epidemiol* 2019;9:e024942.
12. Banno M, Tsujimoto Y, Kataoka Y. The majority of reporting guidelines are not developed with the Delphi method: a systematic review of reporting guidelines. *J Clin Epidemiol* 2020;124;50-7. doi: 10.1016/j.jclinepi.2020.04.010.
13. Gattrell WT, Clements SJ, Sheard D. Quality assessment of guidelines/recommendations developed using Delphi methodology. *Curr Med Res Opin* 2019;35 (Suppl 2):40. doi: 10.1080/03007995.2019.1583496.
14. Ng J. Delphi method: A qualitative approach for quantitative results. *Value Health* 2018;21:S54. Available at https://www.valueinhealthjournal.com/article/S1098-3015(18)30747-2/pdf.
15. Niederberger M, Spranger J. Delphi Technique in Health Sciences: A Map. *Front Public Health* 2020;8:457. doi: 10.3389/fpubh.2020.00457.

For peer review only - http://bmjopen.bmj.com/site/about/guidelines.xhtml

Section: References

16. Waggoner J, Carline JD, Durning SJ. Is There a Consensus on Consensus Methodology? Descriptions and Recommendations for Future Consensus Research. *Acad Med* 2016;91:663-8. doi: 10.1097/ACM.0000000000001092.
17. Wang X, Chen Y, Yang N, et al. Methodology and reporting quality of reporting guidelines: systematic review. *BMC Med Res Methodol* 2015;15:74. doi: 10.1186/s12874-015-0069-z.
18. Grant S, Booth M, Khodyakov D. Lack of preregistered analysis plans allows unacceptable data mining for and selective reporting of consensus in Delphi studies. *J Clin Epidemiol* 2018;99:96-105. doi: 10.1016/j.jclinepi.2018.03.007.

For peer review only

PRISMA 2020 for Abstracts Checklist

Section and Topic	Item #	Checklist item	Reported (Yes/No)
**TITLE**			
Title	1	Identify the report as a systematic review.	Yes
**BACKGROUND**			
Objectives	2	Provide an explicit statement of the main objective(s) or question(s) the review addresses.	Yes
**METHODS**			
Eligibility criteria	3	Specify the inclusion and exclusion criteria for the review.	Yes
Information sources	4	Specify the information sources (e.g. databases, registers) used to identify studies and the date when each was last searched.	Yes
Risk of bias	5	Specify the methods used to assess risk of bias in the included studies.	Not applicable
Synthesis of results	6	Specify the methods used to present and synthesise results.	Not applicable
**RESULTS**			
Included studies	7	Give the total number of included studies and participants and summarise relevant characteristics of studies.	Yes
Synthesis of results	8	Present results for main outcomes, preferably indicating the number of included studies and participants for each. If meta-analysis was done, report the summary estimate and confidence/credible interval. If comparing groups, indicate the direction of the effect (i.e. which group is favoured).	Yes
**DISCUSSION**			
Limitations of evidence	9	Provide a brief summary of the limitations of the evidence included in the review (e.g. study risk of bias, inconsistency and imprecision).	Not applicable
Interpretation	10	Provide a general interpretation of the results and important implications.	Yes
**OTHER**			
Funding	11	Specify the primary source of funding for the review.	Not in abstract, in main document
Registration	12	Provide the register name and registration number.	Yes

From: Page MJ, McKenzie JE, Bossuyt PM, Boutron I, Hoffmann TC, Mulrow CD, et al. The PRISMA 2020 statement: an updated guideline for reporting systematic reviews. BMJ 2021;372:n71. doi: 10.1136/bmj.n71

For peer review only

PRISMA 2020 for Abstracts Checklist

For more information, visit: http://www.prisma-statement.org/

For peer review only

For peer review only - http://bmjopen.bmj.com/site/about/guidelines.xhtml

PRISMA 2020 Checklist

Section and Topic	Item #	Checklist item	Location where item is reported
**TITLE**			
Title	1	Identify the report as a systematic review.	Page 1
**ABSTRACT**			
Abstract	2	See the PRISMA 2020 for Abstracts checklist.	Page 2
**INTRODUCTION**			
Rationale	3	Describe the rationale for the review in the context of existing knowledge.	Page 4, 5
Objectives	4	Provide an explicit statement of the objective(s) or question(s) the review addresses.	Page 5
**METHODS**			
Eligibility criteria	5	Specify the inclusion and exclusion criteria for the review and how studies were grouped for the syntheses.	Page 5
Information sources	6	Specify all databases, registers, websites, organisations, reference lists and other sources searched or consulted to identify studies. Specify the date when each source was last searched or consulted.	Page 6
Search strategy	7	Present the full search strategies for all databases, registers and websites, including any filters and limits used.	Online supplemental material 2
Selection process	8	Specify the methods used to decide whether a study met the inclusion criteria of the review, including how many reviewers screened each record and each report retrieved, whether they worked independently, and if applicable, details of automation tools used in the process.	Page 6
Data collection process	9	Specify the methods used to collect data from reports, including how many reviewers collected data from each report, whether they worked independently, any processes for obtaining or confirming data from study investigators, and if applicable, details of automation tools used in the process.	Page 6, 7
Data items	10a	List and define all outcomes for which data were sought. Specify whether all results that were compatible with each outcome domain in each study were sought (e.g. for all measures, time points, analyses), and if not, the methods used to decide which results to collect.	Online supplemental material 3
	10b	List and define all other variables for which data were sought (e.g. participant and intervention characteristics, funding sources). Describe any assumptions made about any missing or unclear information.	Online supplemental material 3
Study risk of bias assessment	11	Specify the methods used to assess risk of bias in the included studies, including details of the tool(s) used, how many reviewers assessed each study and whether they worked independently, and if applicable, details of automation tools used in the process.	Not applicable
Effect measures	12	Specify for each outcome the effect measure(s) (e.g. risk ratio, mean difference) used in the synthesis or presentation of results.	Not applicable
Synthesis methods	13a	Describe the processes used to decide which studies were eligible for each synthesis (e.g. tabulating the study intervention characteristics and comparing against the planned groups for each synthesis (item #5)).	Not applicable
	13b	Describe any methods required to prepare the data for presentation or synthesis, such as handling of missing summary statistics, or data conversions.	Not applicable
	13c	Describe any methods used to tabulate or visually display results of individual studies and syntheses.	Not applicable
	13d	Describe any methods used to synthesize results and provide a rationale for the choice(s). If meta-analysis was performed, describe the model(s), method(s) to identify the presence and extent of statistical heterogeneity, and software package(s) used.	Not applicable

For peer review only - http://bmjopen.bmj.com/site/about/guidelines.xhtml

PRISMA 2020 Checklist

Section and Topic	Item #	Checklist item	Location where item is reported
	13e	Describe any methods used to explore possible causes of heterogeneity among study results (e.g. subgroup analysis, meta-regression).	Not applicable
	13f	Describe any sensitivity analyses conducted to assess robustness of the synthesized results.	Not applicable
Reporting bias assessment	14	Describe any methods used to assess risk of bias due to missing results in a synthesis (arising from reporting biases).	Not applicable
Certainty assessment	15	Describe any methods used to assess certainty (or confidence) in the body of evidence for an outcome.	Not applicable
**RESULTS**			
Study selection	16a	Describe the results of the search and selection process, from the number of records identified in the search to the number of studies included in the review, ideally using a flow diagram.	Page 7 Fig 1
	16b	Cite studies that might appear to meet the inclusion criteria, but which were excluded, and explain why they were excluded.	Page 8, Fig 1
Study characteristics	17	Cite each included study and present its characteristics.	Page 7, 8
Risk of bias in studies	18	Present assessments of risk of bias for each included study.	Not applicable
Results of individual studies	19	For all outcomes, present, for each study: (a) summary statistics for each group (where appropriate) and (b) an effect estimate and its precision (e.g. confidence/credible interval), ideally using structured tables or plots.	Table 1 and 2<br>Online supplemental material 4, 5 and 6
Results of syntheses	20a	For each synthesis, briefly summarise the characteristics and risk of bias among contributing studies.	Not applicable
	20b	Present results of all statistical syntheses conducted. If meta-analysis was done, present for each the summary estimate and its precision (e.g. confidence/credible interval) and measures of statistical heterogeneity. If comparing groups, describe the direction of the effect.	Not applicable
	20c	Present results of all investigations of possible causes of heterogeneity among study results.	Not applicable
	20d	Present results of all sensitivity analyses conducted to assess the robustness of the synthesized results.	Not applicable
Reporting biases	21	Present assessments of risk of bias due to missing results (arising from reporting biases) for each synthesis assessed.	Not applicable
Certainty of evidence	22	Present assessments of certainty (or confidence) in the body of evidence for each outcome assessed.	Not applicable
**DISCUSSION**			
Discussion	23a	Provide a general interpretation of the results in the context of other evidence.	Page 11-13
	23b	Discuss any limitations of the evidence included in the review.	Page 3, 11, 12

For peer review only

PRISMA 2020 Checklist

Section and Topic	Item #	Checklist item	Location where item is reported
	23c	Discuss any limitations of the review processes used.	Page 3, 11-13
	23d	Discuss implications of the results for practice, policy, and future research.	Page 13
**OTHER INFORMATION**			
Registration and protocol	24a	Provide registration information for the review, including register name and registration number, or state that the review was not registered.	Page 1, 5
	24b	Indicate where the review protocol can be accessed, or state that a protocol was not prepared.	Page 5 Online supplemental material 1 ref 13 and 15
	24c	Describe and explain any amendments to information provided at registration or in the protocol.	Online supplemental material 1
Support	25	Describe sources of financial or non-financial support for the review, and the role of the funders or sponsors in the review.	Page 14
Competing interests	26	Declare any competing interests of review authors.	Page 14
Availability of data, code and other materials	27	Report which of the following are publicly available and where they can be found: template data collection forms; data extracted from included studies; data used for all analyses; analytic code; any other materials used in the review.	Online supplemental material 1-6

From: Page MJ, McKenzie JE, Bossuyt PM, Boutron I, Hoffmann TC, Mulrow CD, et al. The PRISMA 2020 statement: an updated guideline for reporting systematic reviews. BMJ 2021;372:n71. doi: 10.1136/bmj.n71

For more information, visit: http://www.prisma-statement.org/

For peer review only

BMJ Open

Existing guidance on reporting of consensus methodology: a systematic review to inform ACCORD guideline development

Journal:	*BMJ Open*
Manuscript ID	bmjopen-2022-065154.R2
Article Type:	Original research
Date Submitted by the Author:	29-Jul-2022
Complete List of Authors:	van Zuuren, Esther; Leiden University Medical Center, Department of Dermatology<br>Logullo, Patricia; University of Oxford, CSM (Centre for Statistics in Medicine)<br>Price, Amy; Stanford University School of Medicine<br>Fedorowicz, Zbys; Veritas Health Sciences Consultancy<br>Hughes, Ellen L; Sciwright Limited<br>Gattrell, William T; Ipsen
**Primary Subject Heading**:	Research methods
Secondary Subject Heading:	Health policy
Keywords:	Protocols & guidelines < HEALTH SERVICES ADMINISTRATION & MANAGEMENT, STATISTICS & RESEARCH METHODS, Health policy < HEALTH SERVICES ADMINISTRATION & MANAGEMENT

SCHOLARONE™
Manuscripts

I, the Submitting Author has the right to grant and does grant on behalf of all authors of the Work (as defined in the below author licence), an exclusive licence and/or a non-exclusive licence for contributions from authors who are: i) UK Crown employees; ii) where BMJ has agreed a CC-BY licence shall apply, and/or iii) in accordance with the terms applicable for US Federal Government officers or employees acting as part of their official duties; on a worldwide, perpetual, irrevocable, royalty-free basis to BMJ Publishing Group Ltd ("BMJ") its licensees and where the relevant Journal is co-owned by BMJ to the co-owners of the Journal, to publish the Work in this journal and any other BMJ products and to exploit all rights, as set out in our licence.

The Submitting Author accepts and understands that any supply made under these terms is made by BMJ to the Submitting Author unless you are acting as an employee on behalf of your employer or a postgraduate student of an affiliated institution which is paying any applicable article publishing charge ("APC") for Open Access articles. Where the Submitting Author wishes to make the Work available on an Open Access basis (and intends to pay the relevant APC), the terms of reuse of such Open Access shall be governed by a Creative Commons licence – details of these licences and which Creative Commons licence will apply to this Work are set out in our licence referred to above.

Other than as permitted in any relevant BMJ Author's Self Archiving Policies, I confirm this Work has not been accepted for publication elsewhere, is not being considered for publication elsewhere and does not duplicate material already published. I confirm all authors consent to publication of this Work and authorise the granting of this licence.

For peer review only

Existing guidance on reporting of consensus methodology: a systematic review to inform ACCORD guideline development

Esther J van Zuuren[1], Patricia Logullo[2], Amy Price[3], Zbys Fedorowicz[4], Ellen L Hughes[5], William T Gattrell[6]

[1]Department of Dermatology, Leiden University Medical Centre, Leiden, The Netherlands

[2]Centre for Statistics in Medicine (CSM), University of Oxford, and EQUATOR Network UK Centre, Oxford, UK

[3]Stanford University School of Medicine, Stanford, CA, USA

[4]Veritas Health Sciences Consultancy, Huntingdon, United Kingdom

[5]Sciwright Limited, Somerset, UK

[6]Global Medical Affairs, Ipsen, Abingdon, UK

Corresponding author: Esther J van Zuuren,
Department of Dermatology B1-Q
Leiden University Medical Centre
Albinusdreef 2
2333ZA Leiden
The Netherlands
email: e.j.van_zuuren@lumc.nl

Word count: 2706

Keywords: systematic review, consensus, Delphi, reporting guideline

For peer review only

For peer review only - http://bmjopen.bmj.com/site/about/guidelines.xhtml

ABSTRACT

Objective: To identify evidence on the reporting quality of consensus methodology, and to select potential checklist items for the ACCORD (ACcurate COnsensus Reporting Document) project to develop a consensus reporting guideline.

Design: Systematic review.

Data sources: Embase, MEDLINE, Web of Science, PubMed, Cochrane Library, Emcare, Academic Search Premier and PsycINFO from inception until 7 January 2022.

Eligibility criteria: Studies, reviews and published guidance addressing the reporting quality of consensus methodology for improvement of health outcomes in biomedicine or clinical practice. Reports of studies using or describing consensus methods but not commenting on their reporting quality were excluded. No language restrictions were applied.

Data extraction and synthesis: Screening and data extraction of eligible studies were carried out independently by two authors. Reporting quality items addressed by the studies were synthesized narratively.

Results: Eighteen studies were included: 5 systematic reviews, 4 narrative reviews, 3 research papers, 3 conference abstracts, 2 research guidance papers and 1 protocol. The majority of studies indicated that the quality of reporting of consensus methodology could be improved. Commonly addressed items were: consensus panel composition; definition of consensus; and the threshold for achieving consensus. Items least addressed were: public patient involvement (PPI); the role of the steering committee, chair, co-chair; conflict of interest of panellists; and funding. Data extracted from included studies revealed additional items that were not captured in the data extraction form such as justification of deviation from the protocol or incentives to encourage panellist response.

Conclusion: The results of this systematic review confirmed the need for a reporting checklist for consensus methodology and provided a range of potential checklist items to report. The next step in the ACCORD project builds on this systematic review and focuses on reaching consensus on these items to develop the reporting guideline.

Protocol registration: The protocol is registered at https://osf.io/2rzm9.

For peer review only - http://bmjopen.bmj.com/site/about/guidelines.xhtml

STRENGTHS AND LIMITATIONS OF THIS STUDY

- This systematic review utilised a comprehensive search of multiple databases without language restriction
- The included studies ranged from conference abstracts and protocols to guidelines and systematic reviews
- For full transparency and to promote discussion, all data retrieved are reported
- The data extraction form used may have missed a few potential reporting topics, but these will be recovered, in the nest stages of the ACCORD project, by additional reviews and the Delphi panel experience
- Conclusions are limited by the paucity of studies that provided substantial useful guidance

For peer review only

INTRODUCTION

Healthcare providers face continuing challenges in making treatment decisions, particularly where available information on a clinical topic is limited, contradictory, or non-existent. In such situations, alternative and complementary approaches underpinned by collective judgement and based on expert consensus may be used.[1-3]

A variety of approaches with differing methodological rigour can be used to achieve consensus-based decisions. These range from informal "expert consensus meetings" to structured or systematic approaches such as the Delphi method and the Nominal Group Technique (NGT). These methods can be used for generating ideas or determining priorities and aim to achieve consensus through voting on a series of multiple-choice questions.[4-7] The voting process varies according to the method and may take place anonymously (as in Delphi) and/or face to face (in NGT and consensus conferences).[8-10] Key elements in the process include the use of valid and reliable methods to reach consensus and subsequently their transparent reporting; however, these aspects are seldom clearly and explicitly reported.[3, 11]

Reporting guidelines have been developed and are in use for the majority of study designs, e.g. PRISMA, CONSORT and STROBE (for all existing reporting guidelines see: https://www.equator-network.org/). However, no research reporting guideline exists for studies involving consensus methodology other than best practice guidance for Delphi studies in palliative care.[12] Guidelines should include "a checklist, flow diagram, or explicit text to guide authors in reporting a specific type of research, developed using explicit methodology".[3]

Deficiencies in the reporting of consensus methods have been well documented in the literature and are referred to in the protocol for the ACCORD (ACcurate COnsensus Reporting Document) project, which aims to develop a reporting guideline for methods used to reach consensus.[13] In accordance with the EQUATOR Network guidance in the toolkit for the development of reporting guidelines, the

For peer review only - http://bmjopen.bmj.com/site/about/guidelines.xhtml

next step for the ACCORD project was a review of the relevant literature, which would ultimately inform the voting process.[3]

Our objective was to undertake a thorough and comprehensive systematic review that seeks to identify evidence on the quality of reporting of consensus methodology, for subsequent development into a draft checklist of items for the ACCORD guideline. This ACCORD reporting guideline will assist the biomedical research and clinical practice community to describe the methods used to reach consensus in a complete, transparent, and consistent manner.

METHODS

This manuscript conforms to the Preferred Reporting Items for Systematic Reviews and Meta-Analyses (PRISMA) statement,[14] and follows a prespecified protocol (Supplementary Material 1).[13] The protocol was registered on 12 October 2021 at the Open Science Framework (OSF).[15]

Inclusion criteria

Eligible studies consisted of reviews and published guidance which addressed the reporting quality of consensus methodology and aimed to improve health outcomes in biomedicine or clinical practice.

Exclusion criteria

Excluded were publications using consensus methods or describing consensus methods, or discussing the advantages or disadvantages of frameworks, procedures, or techniques to reach consensus, without specifically addressing reporting quality. Examples include guidelines developed through the use of consensus methodologies, such as reporting guidelines, clinical practice guidelines or core outcome set development studies. Editorials (usually brief opinion-based comments), letters about individual publications, and commentaries on consensus methods outside the scope of biomedical research (for example, in the social sciences, economy, politics or marketing) were also excluded for this systematic review.

Literature search strategy and data sources

For peer review only - http://bmjopen.bmj.com/site/about/guidelines.xhtml

A systematic literature search was conducted on 7 January 2022 by a biomedical information specialist. The following bibliographical databases were searched: MEDLINE (OVID version), Embase (OVID version), PubMed, Web of Science, MEDLINE (Web of Science), Cochrane Library, Emcare (OVID version), PsycINFO (EbscoHOST version) and Academic Search Premier. The full search strategy is presented in Supplementary Material 2.

We (EJvZ, ZF, PL and WTG) piloted four initial search strategies provided by the information specialist (JWS, see Acknowledgements). The initial search strategy was sensitive and precise, producing the highest number of retrieved references (N = 7951). After several rounds of checking through known relevant references and controlling for the effect of the performance of certain search terms, modifications were made, including the use of the most explicit terms in the most specific search fields. The performance of search terms was investigated from two vantage points: homonymy (same search term, but different meaning), and, particularly, loss-of-context (right meaning of the word, but not in the correct context). This extended search strategy provided extra 'signal', but also reduced the level of 'noise'. We chose to use specific rather than broad terms (for example, not using the singular terms "delphi" and "consensus" instead we included these words with relevant phrases or with other contextual words). In this way, the refined search strategy was better aligned with our inclusion criteria and the objectives of the systematic review.

For peer review only

Selection process

The final search results were uploaded to Rayyan (https://rayyan.ai) in the blind mode for independent screening by four review authors (EJvZ, ZF, PL, WTG) based on titles and abstracts. No language restrictions were applied. Records deemed eligible or without sufficient detail to make a clear judgement, we retrieved as full-text articles (EJvZ). The same four reviewers independently reassessed the eligibility of these full-text papers and any discrepancies were resolved through discussion. The references of the included studies were also checked for additional potentially eligible studies (EJvZ).

For peer review only - http://bmjopen.bmj.com/site/about/guidelines.xhtml

Data extraction, collection of items and synthesis

Study details and outcome data from the included studies were collected independently within Covidence (https://www.covidence.org/) by two authors using a piloted data extraction form (EJvZ, WTG). The data extraction form questions were compiled based on the review authors' own experiences with reporting quality evaluation and literature on consensus methodology. Furthermore, two additional free text fields were created for extractors to present issues addressed by the included studies that were not captured by the other questions, and for others that the extractors felt were not directly addressed by the studies but were rather inferences about topics that could be potential issues in the reporting of consensus methods. Disagreements were discussed and reconciled by consultation with a third and fourth author (ZF and AP).

The following details were extracted: bibliographic details and reporting items including any suggestions and comments regarding reporting items. Reporting items were divided into the component parts of background, methods, results and discussion, each addressing key aspects of consensus methodology. We also included a section for additional items retrieved from the studies and not captured in the data extraction form. The complete data extraction form can be found as Supplementary Material 3.

The topics extracted and the methods used in the studies included are synthetised narratively, in text and tables (and Supplementary Material). No further analyses were carried out but these will follow during the next stage of the ACCORD project as per protocol.[13]

Patient and public involvement

We involved patients, advocates, and members of the lay public in the initial phases of this protocol [13, 15], as collaborators to develop this project and to co-produce the systematic review and co-author the manuscript. They are collaborating with us by offering their experience with the use of consensus methods to develop guidelines and also systematic reviews. These contributors will work with us to disseminate the results.

For peer review only - http://bmjopen.bmj.com/site/about/guidelines.xhtml

RESULTS

Our searches across the databases identified 2599 articles and 137 further references to abstracts totalling 2736 references (after removal of duplicates) (see Figure 1). A total of 2682 records were excluded after examination of titles and abstracts. Full-text copies of 54 studies were obtained for further assessment of eligibility, and finally, just 18 eligible studies were included. Checking of the references of these full-text publications did not yield any additional eligible articles.

Characteristics of included studies

Eighteen studies matched our prespecified eligibility criteria and were finally included in this review. These studies comprised five systematic reviews,[12, 16-19] four reviews,[20-23] three research papers,[24-26] two research guidelines/guidance,[27, 28] three conference abstracts,[29-31] and one protocol.[32] Of the 18 included studies, 4 used Delphi plus other consensus methods [19, 21, 23 and 28] and the remaining 14 were primarily focused on only the Delphi method.[12, 16-19, 20, 22, 24-27, 29, 30]

Characteristics of excluded studies

A total of 36 studies were excluded.[33- 68] The main reasons for their exclusion were: that they discussed (modified) Delphi methodology but did not include aspects of reporting;[33-54] that they covered reporting but not on consensus methodology;[55-58] that various other consensus methodologies were discussed but not their reporting;[59-67] and that only the concept of experts in consensus methodology was discussed.[68]

Data extraction and narrative synthesis

The majority of studies indicated that reporting of consensus methods could be improved overall. The authors of these studies summarised some current limitations in reporting or proposed suggestions for improvement. Often there were common generic comments that noted reporting of consensus methodologies is inconsistent or lacks transparency. The studies provided few examples of areas that could be reported in more detail such as: selection criteria for the participants and information about the participants; background information for panellists; definition of consensus;

For peer review only - http://bmjopen.bmj.com/site/about/guidelines.xhtml

For peer review only

response rates after each round; description of level of anonymity or how anonymity was maintained; and feedback between rounds (see Table 1).

Table 1 Data on reporting quality of consensus methodologies

Items that are not or not adequately reported in sufficient detail	
Selection criteria for participants/information about the participants [16, 19, 23, 26, 32]	Statement that anonymity was maintained or level of anonymity [[20, 21, 25, 28, 29, 32]
Literature review [20, 21, 31]	Type of consensus method used [29]
Background information for participants [20, 21, 25, 28]	Threshold of consensus [29]
Recruitment strategies [19, 22]	How questionnaire was developed [26]
Criteria for number of rounds [16, 26]	Pretesting of instruments [19, 32]
Stopping criteria [16, 32]	Analysis procedure [24, 32]
Feedback after rounds [17, 20, 21, 23, 25, 26, 28, 31, 32]	Changes to registered pre-analysis plan [24]
Rating scales used [31]	Reporting final number of list of items [32]
Criteria for dropping items [26]	Conflict of interest of panellists [29]
Response rates for each round [17 20, 21, 25, 26, 28, 32]	Funding source [29]
Definition of consensus [17-19, 21, 23, 25, 26, 28]	External support [29]
Level of consensus reached [19, 31]	Generic comments that reporting needs improvement [12, 17, 26, 30]

The studies we reviewed did not provide a systematic or standardised evaluation of the quality of reporting, but they did evaluate the literature critically and offered insights into the gaps of information about consensus. Fifteen papers made recommendations sometimes in the form of short lists —based solely on the authors' opinion, rather than using a systematic approach to reporting guidance development.[12, 16-25, 27, 28, 30, 32] Detailed statements regarding quality of reporting are reproduced in Supplementary Material 4.

In Table 2, we summarise the results of the data extraction, which correlates the corresponding aspects of consensus reporting ("items") to the studies that address them. The items in the table are presented in the format used in the data extraction form (see Supplementary Material 3).

For peer review only - http://bmjopen.bmj.com/site/about/guidelines.xhtml

For peer review only

Table 2. Studies providing guidance for reporting items in the extraction form of this systematic review

Reporting Items	Studies that provide guidance	
Background	Number	References
1.1 Rationale for choosing a consensus method over other methods	4	[12, 25, 27, 28]
1.2 Clearly defined objective	6	[12, 17, 18, 20, 27, 28]
Methods		
2.1 Review of existing evidence informing consensus study	5	[20, 21, 27, 28, 31]
2.2 Inclusion and exclusion criteria of the literature search	3	[17, 20, 22]
2.3 Composition of the panel	16	[12, 16-23, 25-30, 32]
2.4 Public patient involvement (PPI)	0	
2.5 Panel recruitment	4	[12, 17, 22, 23]
2.6 Defining consensus and the threshold for achieving consensus	13	[12, 17-21, 23-29]
2.7 Decision of item approval	3	[12, 17, 27]
2.8 Number of voting rounds	10	[12, 16, 18, 20, 21, 23, 26-28, 32]
2.9 Rationale for number of voting rounds	8	[16, 20, 21-23, 25, 26, 28]
2.10 Time between voting rounds	1	[17]
2.11 Additional methods used alongside consensus	2	[17, 23]
2.12 Software or tools used for voting	1	[25]
2.13 Anonymity of panellists and how this was maintained	7	[16, 20-22, 25, 28, 29]
2.14 Feedback to panellists at the end of each round	11	[17, 19-22, 25-29, 31]
2.15 Synthesis/analysis of responses after voting rounds	5	[12, 22-24, 30]
2.16 Pilot testing of study material/instruments	3	[12, 22, 28]
2.17 Role of the steering committee/chair/co-chair/facilitator	0	
2.18 Conflict of interest or funding received	4	[12, 29, 30, 32]
2.19 Measures to avoid influence by conflict of interest	1	[12]
Results		
3.1 Results of the literature search	1	[12]
3.2 Number of studies found as supporting evidence	0	
3.3 Response rates per voting round	5	[12, 21, 22, 25, 30]
3.4 Results shared with respondents	9	[12, 17, 20, 25-28, 30, 31]
3.5 Dropped items	5	[12, 16, 18, 26, 32]
3.6 Collection, synthesis and comments from panellists	5	[12, 17, 22, 28, 31]
3.7 Final list of items (e.g. for guideline or reporting guideline)	4	[12, 22, 30, 31]
Discussion		
4.1 Limitations and strengths of the study	5	[12, 20, 25, 27, 28]
4.2 Applicability, generalizability, reproducibility	3	[12, 17, 26]

The most frequently addressed item in the included studies (16 times) was the composition of and the criteria for selecting the panellists, including their demographics; specifically, age, gender, specialty, years of experience, and sociodemographic background. The aspects of clarity in, and the importance of, defining consensus and the corresponding thresholds to reach that consensus were addressed in 13 studies. The prespecified number of voting rounds and provision of feedback to the panellists at the end of each round were addressed in 10 and 11 of the studies, respectively.

For peer review only - http://bmjopen.bmj.com/site/about/guidelines.xhtml

None of the included studies reported or made reference to public patient involvement (PPI). The roles of the steering committee/chair/co-chair were not defined in any of the included studies. Reporting of the time interval between voting rounds, panel members' conflicts of interest (COI) and funding sources, as well as the measures used to avoid the influence of COI on voting and decision-making, were minimally addressed.

Conversely, three studies addressed between 12 and 19 reporting items of the 30 items present in the data extraction form of this review,[12, 19, 28] whereas two studies covered only two or three items.[19, 24] We identified a considerable number of other aspects of reporting that were proposed in the included studies, but which were not captured in our data extraction form. These included: 'justifications for deviating from the protocol', 'incentives for encouraging panellists to respond', and 'suggestions to add a flow chart of the consensus process'. All extracted data can be found in Supplementary Material 5 and 6.

DISCUSSION

Although consensus methodology is widely used in healthcare and researchers do raise poor reporting as an issue, we were able to identify only 18 studies that commented on reporting quality and/or provided suggestions to improve the quality of reporting of consensus methodology. These included studies ranged from conference abstracts and protocols to guidelines and systematic reviews. Only four studies covered methods other than the Delphi method and thus providing very limited guidance on other consensus methodologies. As we carried out a comprehensive search of multiple databases without language restriction, it is unlikely that we have missed eligible studies within the period. However, the data extraction form may have missed a few potential reporting topics — which will be recovered, in the next stages of the ACCORD project, by additional reviews and the Delphi panel experience. Furthermore, one study was published after our search date, showing that the development of reporting guidelines for consensus methodologies is an active area, with more studies being published on the topic continuously, which could inform future stages

For peer review only - http://bmjopen.bmj.com/site/about/guidelines.xhtml

or updates of ACCORD.[69] Comments regarding deficient reporting from the included studies varied from generic statements such as “reporting could be improved” to rather specific comments of which aspects of consensus methods were inadequately or not reported. Far more detailed data were provided regarding guidance to improve reporting quality or suggestions for items that require reporting. Both composition and characteristics of the panel, and defining consensus and threshold for achieving assessment received, were consistently addressed and appeared to be critical items that should be reported in sufficient detail. Feedback to the panel might be considered an important aspect of ensuring ongoing engagement with the panellists, transparency and replicability of methods; thus, it was somewhat surprising to see just 11 of the 18 studies consider this an element of consensus methodology worth reporting.

Some items were not addressed in any of the studies, specifically PPI, which is currently considered a key element in the shared decision-making process and is a component of guideline development.[70] Just four studies made reference to the COI of panel members and project funding. COI of panellists, as well as of chair, co-chair and steering committee, can directly or indirectly impact and influence decision-making during the various steps of consensus methodology. As such, COI remains underreported and is often inconsistently described.[71] This also raises concerns about the measures that can be taken to mitigate the potential influence of COI and to ensure that those panellists who do have relevant interests are, for example, not able to vote on pertinent items. For full transparency and to promote discussion, all data retrieved are reported as supplementary material (Supplementary Material 4–6).

Although conclusions are limited by the paucity of studies, a few were particularly informative. The first was a systematic review on the use and reporting of the Delphi method for selecting healthcare indicators.[17] Specifically, this review not only provided guidance for planning and using the Delphi procedure but additionally formulated general recommendations for reporting. The second study was a guidance report on consensus methods such as Delphi and NGT, which were used in medical

For peer review only - http://bmjopen.bmj.com/site/about/guidelines.xhtml

education research.[28] The authors reported that there is a lack of “standardization in definitions, methodology and reporting” and proposed items for researchers to consider when using consensus methods to improve methodological rigour as well as the reporting quality. However, it is worth noting that none of these studies followed the EQUATOR Network guidance for the development of a reporting guideline.[3]

The third study we would like to highlight is the Guidance on Conducting and REporting DElphi Studies (CREDES) in palliative care, which was based on a methodological systematic review.[12] This study focused on the development of guidance in palliative care, although it may not be suitable for extrapolation to other biomedical areas. Furthermore, this study only considered the Delphi methodology, whereas we included studies covering consensus processes involving non-Delphi based methods or “modified Delphi” in our review (and in the ACCORD project overall). However, many of the suggestions made regarding the design and conduct of Delphi studies in addition to recommendations for reporting are equally applicable to our ACCORD project. These items will be used and integrated into the next step of the project, which is the development of a reporting checklist on consensus methods.

Two additional studies proved to be of particular value.[21, 25] One provided a preliminary Delphi checklist to be used for Outcome Measures in Rheumatology (OMERACT).[25] The other concluded, in a scoping review that consensus methods are “poorly standardized and inconsistently used” and exposed reporting flaws in consensus reports.[21]

CONCLUSION

The principal objectives of this systematic review were to conduct a comprehensive search and to identify the existing evidence on the quality of reporting of consensus methodology. As such, we have been able to gather together all relevant studies, summarise the existing research, and highlight key gaps in the current evidence base on consensus methods. This systematic review will

For peer review only - http://bmjopen.bmj.com/site/about/guidelines.xhtml

ultimately inform the generation of a draft checklist of items for the development steps of the ACCORD reporting guideline.

ACKNOWLEDGMENTS

We would like to thank Jan W. Schoones of the Directorate of Research Policy (formerly: Walaeus Library), Leiden University Medical Centre, Leiden, The Netherlands, for his assistance in the development of the search strategy. Furthermore, we would like to thank the other members of the ACCORD project Steering Committee for their contributions to the protocol: Amrit Pali Hungin, Christopher C Winchester, David Tovey, Keith Goldman, Rob Matheis and Niall Harrison. We are grateful for the editorial guidance and reviewer insights from the BMJ Open team. We agree their observations improved the manuscript.

COMPETING INTERESTS

PL is a member of the UK EQUATOR Centre, an organization that promotes the use of reporting guidelines, many of which are developed using consensus methods, and she is personally involved in the development of other reporting guidelines. ELH has worked with Ogilvy Health UK on consensus projects. WTG is a former employee of Ipsen and is now employed by Bristol Myers Squib. AP is an editor at the BMJ, EJvZ and ZF have no conflict of interest.

FUNDING

PL contributed to this study using her time funded by CRUK. WTG contributed his time with the agreement of his employers. Apart from these authors, this study was conducted without external funding for the development of the study design; for the collection, analysis and interpretation of the data; or for the writing of the report. As ZF (Veritas Health Sciences Consultancy) acted as an independent consultant for this work, his support for data extraction and tabulation was funded by Oxford PharmaGenesis. Support for Open Access was also provided by Oxford PharmaGenesis. The decision to submit the paper for publication was made solely by the authors.

CONTRIBUTORS

For peer review only - http://bmjopen.bmj.com/site/about/guidelines.xhtml

EJvZ, PL, ZF and WTG contributed to the screening and agreed on the inclusion of studies. EJvZ and WTG extracted data from the included studies. AP, ZF and ELH contributed to the discussion of extracted data and interpretation. EJvZ was the major contributor in the review of studies, data extraction, interpretation of findings as well as writing of the manuscript. All authors read the final manuscript, provided feedback and approved the final manuscript. The author EJvZ is the guarantor.

PATIENT CONSENT FOR PUBLICATION

No patient data were used in this study and no patient consent for publication was required.

ETHICS APPROVAL

No patient-level data were used in this study and no ethical approval was sought.

PROVENANCE AND PEER REVIEW

Not commissioned; externally peer reviewed.

DISSEMINATION

A link to this research will be made available on the EQUATOR website and the ACCORD research page, shared via social media, shared with future Delphi respondents and stored in local online institutional repositories.

DATA AVAILABILITY STATEMENT

All key data for this study are included in this article or uploaded as online supplementary information. The ACCORD protocol has been listed on the EQUATOR website (Reporting guidelines under development for other study designs | The EQUATOR Network (equator-network.org)) and registered with the Open Science Framework (https://osf.io/2rzm9).

OPEN ACCESS

For peer review only - http://bmjopen.bmj.com/site/about/guidelines.xhtml

This is an open access article distributed in accordance with the terms of the Creative Commons Attribution (CC BY 4.0) license, which permits others to distribute, remix, adapt and build upon this work, for commercial use, provided the original work is properly cited. See: http://creativecommons.org/licenses/by/4.0.

ORCID iDs

EJvZ: https://orcid.org/0000-0002-4780-0182

PL: https://orcid.org/0000-0001-8708-7003

AP: https://orcid.org/0000-0002-5200-7322

ZF: https://orcid.org/0000-0003-3952-965X

ELH: https://orcid.org/0000-0003-2012-0037

WTG: https://orcid.org/0000-0002-9733-6330

REFERENCES

1. Raine R, Sanderson C, Black N. Developing clinical guidelines: a challenge to current methods. *BMJ* 2005;331:631-3. doi: 10.1136/bmj.331.7517.631.
2. Djulbegovic B, Guyatt G. Evidence vs Consensus in Clinical Practice Guidelines. *JAMA* 2019;322:725-6.doi: 10.1001/jama.2019.9751.
3. Moher D, Schulz KF, Simera I, Altman DG. Guidance for developers of health research reporting guidelines. *PLoS Med* 2010;7:e1000217. doi:10.1371/journal.pmed.1000217.
4. Dalkey N, Helmer O. An Experimental Application of the DELPHI Method to the Use of Experts. Manage Sci. 1963;9:458-467. https://doi.org/10.1287/mnsc.9.3.458.
5. Murphy MK, Black NA, Lamping DL, et al. Consensus development methods, and their use in clinical guideline development. *Health Technol Assess* 1998;2:i-iv, 1-88. doi: 10.3310/hta2030.
6. Foth T, Efstathiou N, Vanderspank-Wright B, et al. The use of Delphi and Nominal Group Technique in nursing education: A review. *Int J Nurs Stud* 2016;60:112-20. doi: 10.1016/j.ijnurstu.2016.04.015.
7. Nair R, Aggarwal R, Khanna D. Methods of formal consensus in classification/diagnostic criteria and guideline development. *Semin Arthritis Rheum* 2011;41:95-105. doi: 10.1016/j.semarthrit.2010.12.001.
8. McMillan SS, King M, Tully MP. How to use the nominal group and Delphi techniques. *Int J Clin Pharm* 2016;38:655-62. doi: 10.1007/s11096-016-0257-x.

For peer review only - http://bmjopen.bmj.com/site/about/guidelines.xhtml

9. Grant S, Armstrong C, Khodyakov D. Online Modified-Delphi: a Potential Method for Continuous Patient Engagement Across Stages of Clinical Practice Guideline Development. *J Gen Intern Med* 2021;36:1746-50. doi: 10.1007/s11606-020-06514-6.
10. Helmer-Hirschberg O. Analysis of the future: The Delphi Method. Santa Monica, CA: RAND Corporation, 1967. Available at https://www.rand.org/pubs/papers/P3558.htm.
11. Arakawa N, Bader LR Consensus development methods: Considerations for national and global frameworks and policy development. *Res Social Adm Pharm* 2022;18:2222-9. doi: 10.1016/j.sapharm.2021.06.024.
12. Jünger S, Payne SA, Brine J, et al. Guidance on Conducting and REporting DElphi Studies (CREDES) in palliative care: Recommendations based on a methodological systematic review. *Palliat Med* 2017;31(8):684-706. doi: 10.1177/0269216317690685.
13. Gattrell WT, Hungin AP, Price A, et al. ACCORD guideline for reporting consensus-based methods in biomedical research and clinical practice: a study protocol. *Res Integr Peer Rev* 2022;7(1):3. doi: 10.1186/s41073-022-00122-0.
14. Page MJ, McKenzie JE, Bossuyt PM, et al. The PRISMA 2020 statement: an updated guideline for reporting systematic reviews. *BMJ* 2021;372:n71. doi: 10.1136/bmj.n71.
15. Gattrell W, Hungin AP, Arents BWMA, et al. Consensus-based methods in biomedical research and clinical practice: The ACCORD study protocol for establishing a reporting guideline. https://osf.io/e5w9n/ (accessed 19 April 2022).
16. Banno M, Tsujimoto Y, Kataoka Y. The majority of reporting guidelines are not developed with the Delphi method: a systematic review of reporting guidelines. *J Clin Epidemiol* 2020;124;50-7. doi: 10.1016/j.jclinepi.2020.04.010.
17. Boulkedid R, Abdoul H, Loustau M, et al. Using and Reporting the Delphi Method for Selecting Healthcare Quality Indicators: A Systematic Review. *PLoS One* 2011;6:e20476. doi: 10.1371/journal.pone.0020476.
18. Diamond IR, Grant RC, Feldman BM, et al. Defining consensus: A systematic review recommends methodologic criteria for reporting of Delphi studies. *J Clin Epidemiol* 2014;67:401-9. doi: 10.1016/j.jclinepi.2013.12.002.
19. Wang X, Chen Y, Yang N, et al. Methodology and reporting quality of reporting guidelines: systematic review. *BMC Med Res Methodol* 2015;15:74. doi: 10.1186/s12874-015-0069-z
20. Chan TM, Yarris LM, Humphrey-Murto S. Delving into Delphis. *CJEM* 2019;21:167-9. doi: 10.1017/cem.2019.3.
21. Humphrey-Murto S, Varpio L, Wood TJ, et al. The Use of the Delphi and Other Consensus Group Methods in Medical Education Research: A Review. *Acad Med* 2017;92:1491-8. doi: 10.1097/ACM.0000000000001812.
22. Paré G, Cameron AF, Poba-Nzaou P, et al. A systematic assessment of rigor in information systems ranking-type Delphi studies. *Inf Manag* 2013;50:207-17 doi: 10.1016/j.im.2013.03.003.
23. Waggoner J, Carline JD, Durning SJ. Is There a Consensus on Consensus Methodology? Descriptions and Recommendations for Future Consensus Research. *Acad Med* 2016;91:663-8. doi: 10.1097/ACM.0000000000001092.
24. Grant S, Booth M, Khodyakov D. Lack of preregistered analysis plans allows unacceptable data mining for and selective reporting of consensus in Delphi studies. *J Clin Epidemiol* 2018;99:96-105. doi: 10.1016/j.jclinepi.2018.03.007.

For peer review only - http://bmjopen.bmj.com/site/about/guidelines.xhtml

25. Humphrey-Murto S, Crew R, Shea B, et al. Consensus Building in OMERACT: Recommendations for Use of the Delphi for Core Outcome Set Development. *J Rheumatol* 2019;46:1041-6. doi: 10.3899/jrheum.181094.
26. Niederberger M, Spranger J. Delphi Technique in Health Sciences: A Map. *Front Public Health* 2020;8:457. doi: 10.3389/fpubh.2020.00457.
27. Hasson F, Keeney S, McKenna H. Research guidelines for the Delphi survey technique. *J Adv Nurs* 2000;32:1008-15. doi: 10.1046/j.1365-2648.2000.t01-1-01567.x.
28. Humphrey-Murto S, Varpio L, Gonsalves C, et al. Using consensus group methods such as Delphi and Nominal Group in medical education research. *Med Teach* 2017;39:14-9. doi: 10.1080/0142159X.2017.1245856.
29. Gattrell WT, Clements SJ, Sheard D. Quality assessment of guidelines/recommendations developed using Delphi methodology. *Curr Med Res Opin* 2019;35 (Suppl 2):40. doi: 10.1080/03007995.2019.1583496.
30. Ng J. Delphi method: A qualitative approach for quantitative results. *Value Health* 2018;21:S54. Available at https://www.valueinhealthjournal.com/article/S1098-3015(18)30747-2/pdf.
31. Resemann HK, Clements S, Griffiths A, et al. Reporting of Delphi methods to achieve consensus on guidelines in rare diseases. *Curr Med Res Opin* 2018;34 (Suppl 1):37.
32. Banno M, Tsujimoto Y, Kataoka Y. Reporting quality of the Delphi technique in reporting guidelines: a protocol for a systematic analysis of the EQUATOR Network Library. *J Clin Epidemiol* 2019;9:e024942.
33. Bellali T, Karamitri I. The Delphi research methodology and its applications in the healthcare sciences. *Arch Hellen Med* 2011;28:839-48. Available at http://www.mednet.gr/archives/2011-6/pdf/839.pdf.
34. Bijl R. Delphi in a future scenario study on mental health and mental health care. *Futures* 1992;24:232-50. doi: 10.1016/0016-3287(92)90033-C.
35. Boel A, Navarro-Compán V, Landewé R, et al. Two different invitation approaches for consecutive rounds of a Delphi survey led to comparable final outcome. *J Clin Epidemiol* 2021;129:31-9. doi: 10.1016/j.jclinepi.2020.09.034.
36. Bowles N. The Delphi technique. *Nurs Stand* 1999;13:21-6. doi: 10.7748/ns1999.07.13.45.32.c2650.
37. Cramer CK, Klasser GD, Epstein JB, et al. The Delphi process in dental research. *J Evid Based Dent Pract* 2008;8:211-20. doi: 10.1016/j.jebdp.2008.09.002.
38. de Loë R, Melnychuk N, Murray D, et al. Advancing the State of Policy Delphi Practice: A Systematic Review Evaluating Methodological Evolution, Innovation, and Opportunities. *Technol Forecast Soc Change* 2016;104:78-88. doi: 10.1016/j.techfore.2015.12.009.
39. Drumm S, Bradley C, Moriarty F. 'More of an art than a science'? The development, design and mechanics of the Delphi Technique. *Res Social Adm Pharm* 2022;18:2230-6. doi: 10.1016/j.sapharm.2021.06.027.
40. Fink-Hafner D, Dagan T, Doušak M, et al. Delphi Method: Strengths and Weaknesses. *Metodoloski Zv* 2019;16:1-19. doi: 10.51936/fcfm6982.
41. Franklin KK, Hart JK. Idea Generation and Exploration: Benefits and Limitations of the Police Delphi Research Method. *Innov High Educ* 2006;31:237-46. 10.1007/s10755-006-9022-8.

For peer review only - http://bmjopen.bmj.com/site/about/guidelines.xhtml

42. Guzys D, Dickson-Swift V, Kenny A, et al. Gadamerian philosophical hermeneutics as a useful methodological framework for the Delphi technique. *Int J Qual Stud Health Well-being* 2015;10:26291. doi: 10.3402/qhw.v10.26291.
43. Hallowell MR, Gambatese JA. Qualitative Research: Application of the Delphi Method to CEM Research. *J Constr Eng Manag* 2010;136:99-107. doi: 0.1061/(ASCE)CO.1943-7862.0000137.
44. Holloway K. Doing the E-Delphi: using online survey tools. *Comput Inform Nurs* 2012;30:347-50. doi: 10.1097/NXN.0b013e31825e8923.
45. Hung HL, Altschuld JW, Lee YF. Methodological and conceptual issues confronting a cross-country Delphi study of educational program evaluation. *Eval Program Plann* 2008;31:191-8. doi: 10.1016/j.evalprogplan.2008.02.005.
46. Ibiyemi AO, Adnan YM, Daud MN. The validity of the classical Delphi applications for assessing the industrial sustainability-correction factor: an example study. *Foresight* 2016;18:603-24. doi: 10.1108/FS-04-2016-0016.
47. Keeney S, Hasson F, McKenna HP. Consulting the oracle: ten lessons from using the Delphi technique in nursing research. *J Adv Nurs* 2006;53:205-12. doi: 10.1111/j.1365-2648.2006.03716.x.
48. Keeney S, Hasson F, McKenna HP. A critical review of the Delphi technique as a research methodology for nursing. *Int J Nurs Stud* 2001;38:195-200. doi: 10.1016/S0020-7489(00)00044-4.
49. Kennedy HR. Enhancing Delphi research: methods and results. *J Adv Nurs* 2004;45:504-11. doi: 10.1046/j.1365-2648.2003.02933.x.
50. Khodyakov D, Grant S, Denger B, et al. Practical Considerations in Using Online Modified-Delphi Approaches to Engage Patients and Other Stakeholders in Clinical Practice Guideline Development. *Patient* 2020;13:11-21. doi: 10.1007/s40271-019-00389-4.
51. Murry J, Hammons J. Delphi: A versatile methodology for conducting qualitative research. *Rev High Educ* 2017;18:423-36. doi: 10.1353/rhe.1995.0008.
52. Olsen AA, Wolcott MD, Haines ST, et al. How to use the Delphi method to aid in decision making and build consensus in pharmacy education. *Curr Pharm Teach Learn* 2021;13:1376-85. doi: 10.1016/j.cptl.2021.07.018.
53. Vernon W. The Delphi technique: A review. *Int J Ther Rehabil* 2009;16:69-76. doi: 10.12968/ijtr.2009.16.2.38892.
54. Von der Gracht HA. Consensus measurement in Delphi studies Review and implications for future quality assurance. Technol Forecast Soc Change 2012;79:1525-36. doi: 10.1016/j.techfore.2012.04.013.
55. Bennett C, Khangura S, Brehaut JC, et al. Reporting guidelines for survey research: an analysis of published guidance and reporting practices. *PloS Med* 2010;8:e1001069. doi: 10.1371/journal.pmed.1001069.
56. Han SJ, Oh MC, Sughrue ME, et al. Reporting standard compliance in publications of vestibular schwannoma patients treated with microsurgery. *Otol Neurotol* 2015;2012:33:648-50. doi: 10.1097/MAO.0b013e31824f998b.
57. Moher D, Weeks L, Ocampo M, et al. Describing reporting guidelines for health research: a systematic review. *J Clin Epidemiol* 2011;64:718-42. doi: 10.1016/j.jclinepi.2010.09.013.

For peer review only - http://bmjopen.bmj.com/site/about/guidelines.xhtml

58. Murray A, Junge A, Robinson PG, et al. International consensus statement: methods for recording and reporting of epidemiological data on injuries and illnesses in golf. Br J Sports Med 2020;54:1136-41. doi: 10.1136/bjsports-2020-102380.
59. Campbell SM, Cantrill JA. Consensus methods in prescribing research. *J Clin Pharm Ther* 2001;26:5-14. doi: 10.1046/j.1365-2710.2001.00331.x.
60. Durkin J, Usher K, Jackson D. Using consensus from experts to inform a shared understanding of subjective terms. *Nurse Res* 2019;27:46-9. doi: 10.7748/nr.2019.e1622.
61. Fink A, Kosecoff J, Chassin M, et al. Consensus methods: characteristics and guidelines for use. *Am J Public Health* 1984;74:979-83. doi: 0.2105/ajph.74.9.979.
62. Halcomb E, Davidson P, Hardaker L. Using the consensus development conference method in healthcare research. *Nurs Res* 2008;16:56-71. doi: 10.7748/nr2008.10.16.1.56.c6753.
63. James D, Warren-Forward H. Research methods for formal consensus development. *Nurs Res* 2015;22:35-40. doi: 10.7748/nr.22.3.35.e1297.
64. McMillan SS, King M, Tully MP. How to use the nominal group and Delphi techniques. *Int J Clin Pharm* 2016;38:655-62. doi: 10.1007/s11096-016-0257-x.
65. Nair R, Aggarwal R, Khanna D. Methods of Formal Consensus in Classification/Diagnostic Criteria and Guideline Development. *Semin Arthritis Rheum* 2011;41:95-105. doi: 10.1016/j.semarthrit.2010.12.001.
66. Rotondi AJ, Kvetan V, Carlet J, et al. Consensus conferences in critical care medicine - Methodologies and impact. *Crit Care Clin* 1997;13:417-39. doi: 10.1016/s0749-0704(05)70319-5.
67. van Teijlingen E, Pitchforth E, Bishop C, et al. Delphi method and nominal group technique in family planning and reproductive health research. *J Fam Plann Reprod Health Care* 2006;32:249-52. doi: 10.1783/147118906778586598.
68. Baker J, Lovell K, Harris N. How expert are the experts? An exploration of the concept of 'expert' within Delphi panel techniques. *Nurs Res* 2006;14:59-70. doi: 10.7748/nr2006.10.14.1.59.c6010.
69. Spranger J, Homberg A, Sonnberger M, Niederberger M. Reporting guidelines for Delphi techniques in health sciences: A methodological reviewBerichterstattungsleitlinien für Delphi-Verfahren in den Gesundheitswissenschaften: Ein methodologisches Review. *Z Evid Fortbild Qual Gesundh wesen (ZEFQ)* Available online since June 16th 2022. doi: https://doi.org/10.1016/j.zefq.2022.04.025.
70. National Institute for Health and Care Excellence. NICE Guideline [NG197]: Shared decision making. Available at: https://www.nice.org.uk/guidance/ng197.
71. Dunn AG, Coiera E, Mandl KD, et al. Conflict of interest disclosure in biomedical research: A review of current practices, biases, and the role of public registries in improving transparency. *Res Integr Peer Rev* 2016;1:1. doi: 10.1186/s41073-016-0006-7.

Figure Legends

Figure 1
Caption: PRISMA 2020 flow diagram for new systematic reviews which included searches of databases, registers and other sources

For peer review only - http://bmjopen.bmj.com/site/about/guidelines.xhtml

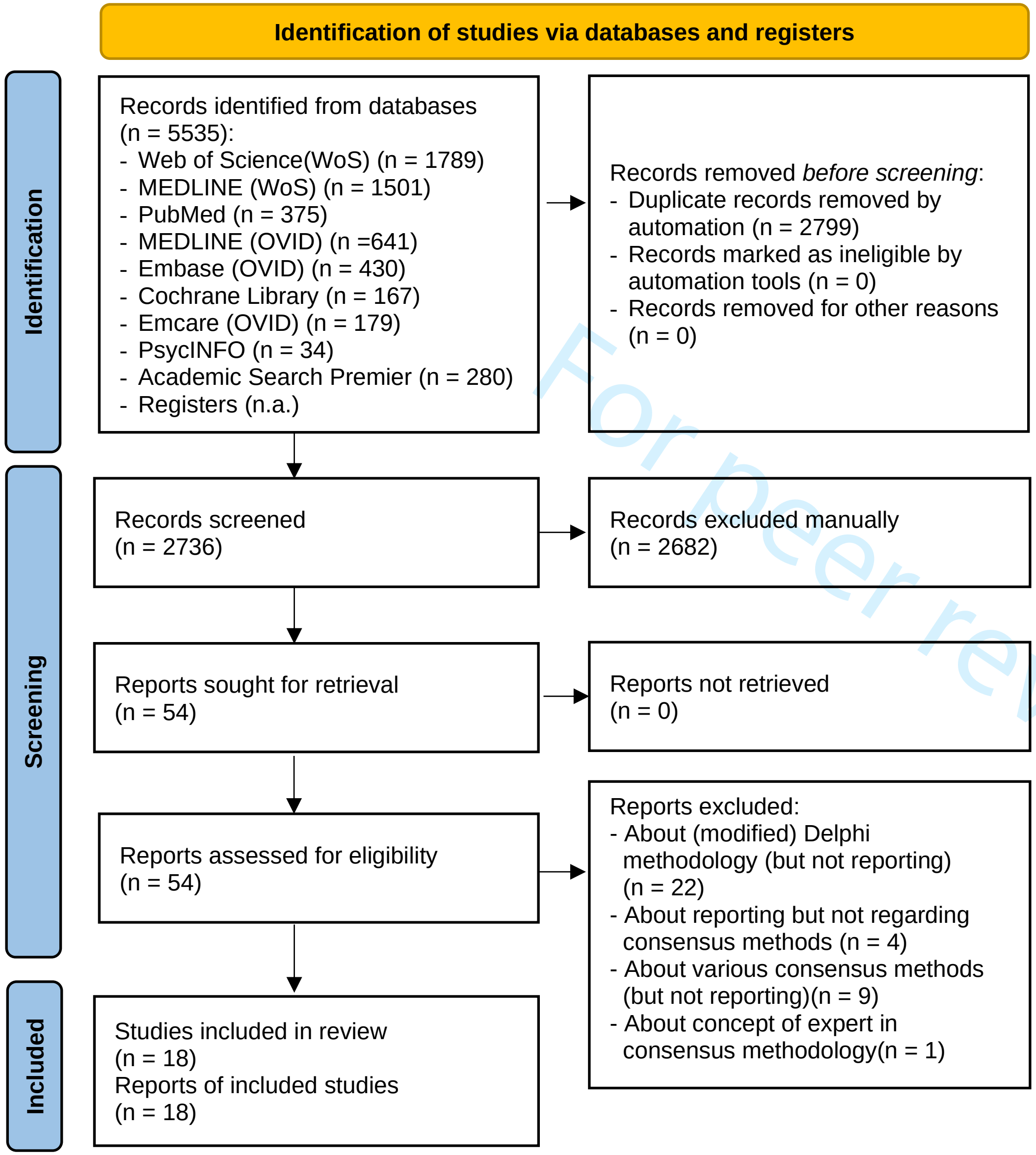

For peer review only

Gattrell *et al.*
Research Integrity and Peer Review (2022) 7:3
https://doi.org/10.1186/s41073-022-00122-0

Research Integrity and Peer Review

STUDY PROTOCOL Open Access

ACCORD guideline for reporting consensus-based methods in biomedical research and clinical practice: a study protocol

William T. Gattrell[1*], Amrit Pali Hungin[2], Amy Price[3], Christopher C. Winchester[4], David Tovey[5], Ellen L. Hughes[6], Esther J. van Zuuren[7], Keith Goldman[8], Patricia Logullo[9], Robert Matheis[10] and Niall Harrison[11]

Abstract

Background: Structured, systematic methods to formulate consensus recommendations, such as the Delphi process or nominal group technique, among others, provide the opportunity to harness the knowledge of experts to support clinical decision making in areas of uncertainty. They are widely used in biomedical research, in particular where disease characteristics or resource limitations mean that high-quality evidence generation is difficult. However, poor reporting of methods used to reach a consensus – for example, not clearly explaining the definition of consensus, or not stating how consensus group panellists were selected – can potentially undermine confidence in this type of research and hinder reproducibility. Our objective is therefore to systematically develop a reporting guideline to help the biomedical research and clinical practice community describe the methods or techniques used to reach consensus in a complete, transparent, and consistent manner.

Methods: The ACCORD (ACcurate COnsensus Reporting Document) project will take place in five stages and follow the EQUATOR Network guidance for the development of reporting guidelines. In Stage 1, a multidisciplinary Steering Committee has been established to lead and coordinate the guideline development process. In Stage 2, a systematic literature review will identify evidence on the quality of the reporting of consensus methodology, to obtain potential items for a reporting checklist. In Stage 3, Delphi methodology will be used to reach consensus regarding the checklist items, first among the Steering Committee, and then among a broader Delphi panel comprising participants with a range of expertise, including patient representatives. In Stage 4, the reporting guideline will be finalised in a consensus meeting, along with the production of an Explanation and Elaboration (E&E) document. In Stage 5, we plan to publish the reporting guideline and E&E document in open-access journals, supported by presentations at appropriate events. Dissemination of the reporting guideline, including a website linked to social media channels, is crucial for the document to be implemented in practice.

Discussion: The ACCORD reporting guideline will provide a set of minimum items that should be reported about methods used to achieve consensus, including approaches ranging from simple unstructured opinion gatherings to highly structured processes.

*Correspondence: wgattrell@yahoo.co.uk

[1] Global Medical Affairs, Ipsen, Abingdon, UK
Full list of author information is available at the end of the article

BMC

© The Author(s) 2022. **Open Access** This article is licensed under a Creative Commons Attribution 4.0 International License, which permits use, sharing, adaptation, distribution and reproduction in any medium or format, as long as you give appropriate credit to the original author(s) and the source, provide a link to the Creative Commons licence, and indicate if changes were made. The images or other third party material in this article are included in the article's Creative Commons licence, unless indicated otherwise in a credit line to the material. If material is not included in the article's Creative Commons licence and your intended use is not permitted by statutory regulation or exceeds the permitted use, you will need to obtain permission directly from the copyright holder. To view a copy of this licence, visit http://creativecommons.org/licenses/by/4.0/. The Creative Commons Public Domain Dedication waiver (http://creativecommons.org/publicdomain/zero/1.0/) applies to the data made available in this article, unless otherwise stated in a credit line to the data.

For peer review only

For peer review only - http://bmjopen.bmj.com/site/about/guidelines.xhtml

Keywords: Methodology, Guidelines, Reporting quality, Reporting completeness, Checklist, Delphi technique, Consensus, Nominal group technique, Consensus development conference

Background

Evidence-based medicine relies on three factors: current best evidence based on clinical and real-world studies, individual clinical expertise, and the desires of the patient [1]. Clinical data gathered from systematic reviews, high-quality randomised clinical trials, and observational studies have complementary roles in generating robust evidence [2, 3]. However, healthcare providers face difficult treatment decisions if the available information on a subject is inadequate, contradictory, limited, or does not exist.

The COVID-19 pandemic has brought this situation of lack of evidence into stark relief, as crucial decisions have to be made during any rapidly emerging public health crisis [4]. However, there are areas of medicine for which high-quality evidence generation can be difficult. This is due to disease characteristics such as rare occurrence and clinical heterogeneity among patients with the same condition, which can mean either that trials are difficult to interpret or that they may only be directly applicable to a subset of patients [5, 6]. A lack of resources and/or infrastructure can also be limiting [6, 7]. Moreover, even when evidence does exist, in medical situations with multiple considerations or confounding factors, there is the need to prioritise the use of available evidence to optimise outcomes [8].

Therefore, when no robust evidence is available, when divergent guidance exists, or when there is a need for collective judgement to increase reliability and validity, guidelines for clinical decision making or methodological or reporting approaches may be formulated based on expert consensus only [9–11]. Consensus methods provide opportunities to harness the knowledge of experts to support clinical decision making in areas of uncertainty [12]. As with all studies, appropriate methods and transparent reporting are key; however, the method used to reach consensus is not always clearly reported [11, 13].

Multiple methods are used to develop consensus-based publications. These range in methodological rigour from informal "expert consensus meetings" to structured or systematic approaches such as the Delphi method and the nominal group technique (NGT). Both Delphi and NGT are used for generating ideas or determining priorities, aiming to achieve general convergence, usually through voting on a series of multiple-choice questions [14–17]. In Delphi, and more recently electronic Delphi (eDelphi), individuals vote anonymously, while NGT is usually face-to-face [8, 18, 19]. The techniques and methodological steps used to reach consensus can vary (Table 1).

In group decisions, a wider range of knowledge may be drawn upon, the interaction between group members can stimulate and challenge received ideas, and idiosyncrasies may be filtered out through the group prioritisation process [19, 31–33]. The use of structured, systematic approaches to reach consensus is supported by the observation that, in an unstructured group meeting, there is the risk of a single individual dominating the discussion and decisions may be portrayed as unanimous when, in reality, there is dissent within the group [31]. Even within structured consensus meetings, depending on their roles, a few panel members can dominate the discussion [34]. Furthermore, individuals may be unwilling to retract long-held views in open discussion. For these reasons, structured approaches including a step where responses are anonymised are generally held to be superior to unstructured methods to achieve consensus [35, 36].

Developing consensus-based publications using robust methods is vital, but poor execution or reporting can render the techniques used for gathering opinion susceptible to criticism [37–40]. To take one of the most widely-used and most rigorous consensus methodologies, the Delphi method has been used extensively in a wide range of sectors including military, education, social science and healthcare since its conception in the 1950s at the RAND Corporation [41]. This is because it has the potential to mitigate many of the aforementioned pitfalls in group decisions, such as the risk of peer pressure in techniques such as the NGT [38, 42]. Due to its versatility, the Delphi method can be modified to meet individual study needs. However, the reporting of such "modified Delphi" methods may lack clarity on the details of the process involved or the rationale for the modification [38, 42].

Definitions of the thresholds for consensus (i.e., approval rates), for example, can vary or be poorly described in studies using consensus [43]. Other reporting or methodological problems identified are that analytical methods may not be predefined [37, 43], the recruitment process used to identify the experts may not be explicit [44], or the funding source not clearly disclosed [45]. In fact, critics suggest the term "Delphi research" be phased out in academic publications to force authors to more precisely describe the methodology used [46].

For peer review only - http://bmjopen.bmj.com/site/about/guidelines.xhtml

Table 1 Possible types of consensus methods and characteristics that can be mixed or used separately in different stages of studies to reach consensus

Method	Characteristics	Data analysis
Consensus conference or meeting [20–22]	Face-to-face meetings where a group of participants, usually experts in one field of knowledge, discuss one or more topics, prompted by facilitators, and have to either create ideas/statements or decide/vote on pre-set topics/statements. The discussion is frequently prompted by evidence from the literature — or the lack of it.	Qualitative or quantitative, or mixed.
Nominal group technique (NGT) [20, 22, 23]	As in conference meetings, in NGT, face-to-face meetings are held, but several sessions are organised with iterative stages. In the first step, suggestions are collected from the groups into questionnaires or lists of topics circulated again in the second step. In the second stage, participants need to vote or rate, usually using scales (like Likert scales). The group then discusses the aggregated summary of the voting or rating. The group is not anonymous and may include experts and non-experts. A facilitator makes sure every participant is given the opportunity to speak and vote.	Qualitative initially and then quantitative when responses are aggregated and summarised.
Delphi [12, 20, 22–30]	The three principles of the Delphi technique are: 1) anonymity during voting/selecting/rating (participants do not meet); 2) multiple rounds (at least 2) and 3) feedback to participants to inform them about each last voting/rating before they start the next round. Delphi was traditionally organised by postal mail in the past, and now electronic specialised survey platforms facilitate the process.	Quantitative for voting/rating, qualitative when extra comments/suggestions are allowed.
Other mixed methods [20, 22]	A consensus study can begin with simple focus groups to collect ideas, stories, experiences, and general opinions to start a more structured NGT or Delphi exercise. Frequently, two or more methods are used. For example, a Delphi activity can be used initially with the list of statements approved to be discussed in consensus conferences where final decisions are made, sometimes referred to as a "modified Delphi".	Qualitative methods are used when perceptions, stories, and experiences are collected. Several quantitative statistics can be used to summarise voting and ratings.

The lack of appropriate and transparent description in publications of the consensus methods used suggests that a reporting guideline is needed. A reporting guideline comprises "a checklist, flow diagram, or explicit text to guide authors in reporting a specific type of research, developed using explicit methodology" [11]. Consensus methods themselves play an important role in the development of reporting guidelines in various fields of health. As part of an ongoing audit of the EQUATOR database [47], it has been observed that, of the 226 reporting guidelines added between database inception and October 2018, only one third (77/226) explicitly mentioned the use of Delphi methodology (Fig. 1), while in another third (75/226), the information was not reported. A systematic review of the EQUATOR database indicated a similar result and added that among the reporting guidelines that mentioned the Delphi method, the description of details of the participants, number of rounds, criteria for dropping items or stopping the rounds was not always reproducible [48].

A range of methods can be used to reach consensus for clinical guidance, nomenclature, and other approaches in healthcare and public health [49]. However, to the best of our knowledge, the only reporting guidance in healthcare using consensus research is the CREDES (guidance on Conducting and REporting DElphi Studies) Statement, which provides valuable recommendations for the reporting of Delphi consensus in palliative care [38]. Nevertheless, CREDES is specific to palliative care and is limited to the Delphi method [38], which leaves a gap for a reporting guideline that can be applied to other biomedical areas and consensus processes involving non-Delphi based methods or "modified Delphi" — an issue that CREDES acknowledges. Moreover, CREDES does not provide a detailed checklist to guide the incorporation of essential steps to be reported.

For peer review only - http://bmjopen.bmj.com/site/about/guidelines.xhtml

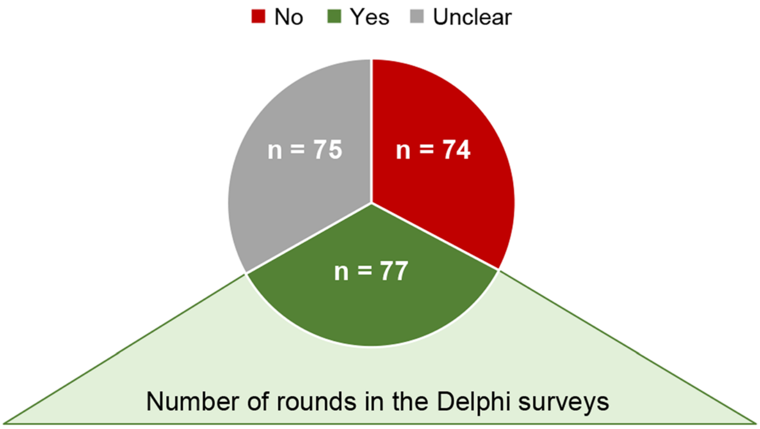

Number of rounds in the Delphi surveys

Rounds	n	%
1	8	3.54
2	32	14.16
3	24	10.62
4	6	2.65
5	1	0.44
Unclear	6	2.65
N/A	149	65.94

Fig. 1 Methodology declared by authors in developing a reporting guideline added to the EQUATOR database from inception to October 2018 (N = 226)

Detail-oriented reporting can help readers of publications to understand the key elements of the process – the methodology used, the participants involved, and how the study was conducted including the criteria for statement approval. Our objective is therefore to systematically develop a reporting guideline to help the biomedical research and clinical practice community describe the methods used to reach consensus in a complete, transparent, and consistent manner. Our aim is that the reporting guideline is appropriate to describe all types of consensus methodology. The reporting guideline for consensus-based biomedical publications will include a general statement with a checklist and an explanation and elaboration (E&E) document, including examples of good reporting. It will be identified under the acronym ACCORD (ACcurate COnsensus Reporting Document).

Methods/design

We have adopted the general method proposed by the EQUATOR Network for developing reporting guidelines [11]. The process for ACCORD development is outlined in Fig. 2.

Stage 1: establishment of a Steering Committee

With the endorsement of the International Society of Medical Publication Professionals (ISMPP), we assembled a Steering Committee to develop a reporting guideline for research using consensus. The Steering Committee (the authors, AH, AP, CW, DT, EH, EvZ, KG, NH, PL, RM, and WG) will lead and co-ordinate the guideline development process. Specifically, the Steering Committee will be responsible for: establishing the goals and timelines for the work, including registering and publishing the protocol; generating the initial list of checklist items from the literature review; conducting a consensus process to enrich and refine the initial list of minimum items that should be reported; implementing each stage of the process including developing questionnaires and analysing voting outcomes and other data; reporting the findings of the process in a statement document with the main checklist and guidance; developing an E&E document where all the items are individually explained and examples of approach and reporting are given; disseminating the reporting guidelines via publication, presentation at congresses and other events, and online presence including a website linked to social media channels.

The Steering Committee is a multidisciplinary group (11 people) that includes clinician practitioners, methodologists, publication professionals, patients, journal editors and publishers and the pharmaceutical industry. Prior to initiating Stage 2, we listed the project in the EQUATOR Network registry for reporting guidelines under development [50] and registered the protocol with the Open Science Framework [51].

For peer review only - http://bmjopen.bmj.com/site/about/guidelines.xhtml

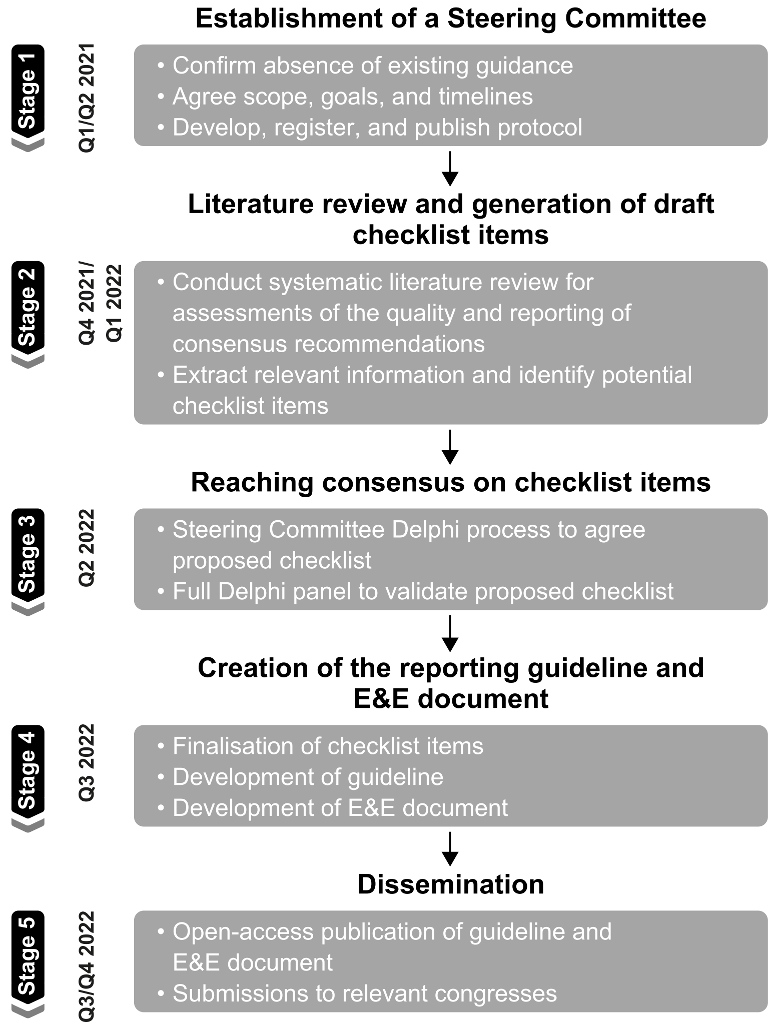

Fig. 2 Project overview for creating ACCORD, a reporting guideline for studies developed using consensus methods

Stage 2: literature review and generation of draft checklist items

The aim of this step is to seek evidence on the quality of reporting of the process undertaken in health studies using consensus methodology. This research will provide insight into possible checklist items for evaluation by the Delphi Panel (further information on the Delphi Panel is provided in 'Stage 3' below). The CREDES guidelines, specific to palliative care, will also be reviewed for elements that can be generalised to other biomedical fields [38].

Search strategy

The process for conducting the systematic review will be informed by and reported according to the Preferred Reporting Items for Systematic reviews and Meta-Analyses (PRISMA) 2020 and PRISMA-Search extension guidelines [11, 52]. Eligible studies will include studies, reviews and published guidance addressing the quality of reporting of consensus methodology that aim to improve health outcomes in biomedicine or clinical practice. Reports of studies using consensus methods but not commenting on their reporting quality will be excluded, for example, studies to reach clinical recommendations of core outcome sets or reporting guidelines using consensus methods. Ineligible publications include editorials, letters about individual publications, and comments on methodology of consensus outside the scope of biomedical research.

Searches of EMBASE (OVID), MEDLINE (OVID), Web of Science - Core Collection, MEDLINE (Web of Science), PubMed, Cochrane Library, and Emcare (OVID), Academic Search Premier and PsycINFO databases will be run with no limits by year or language of publication at the search stage. Four initial search strategies were developed and sequentially

For peer review only - http://bmjopen.bmj.com/site/about/guidelines.xhtml

piloted by members of the Steering Committee (WG, EvZ and PL) with the assistance of an information (JS) and systematic review specialist (ZF). The piloting allowed the adjustment of the initial search strategy by the information specialist to provide results that better aligned with the inclusion criteria and objective of this study. The refined, broad search strategy (Supplementary File) will be used to identify and generate the final list of studies focusing on the quality and accuracy of reporting of Delphi and other consensus processes, methods, techniques or recommendations. The search may also be augmented with relevant articles highlighted by the Steering Committee as appropriate based on the individuals' prior work and expertise in the area (via a manual search).

Data extraction

EvZ, PL, WG, and ZF will independently screen the titles and abstracts retrieved from the search for potential inclusion using the Rayyan tool in blind mode [53]. Any discrepancies will be resolved by discussion. Full-text articles will then be retrieved and assessed independently for eligibility, with reconciliation of any differences through discussion. Data will be extracted using a draft extraction form, which will be piloted on three studies before use. Based on the information gathered on the literature review, a list of preliminary items for the checklist will be generated to be refined in a Delphi exercise in Stage 3.

Stage 3: reaching consensus on checklist items

We will use Delphi methodology, as described below, to reach a consensus regarding the checklist items to include in the reporting guideline. This will take place in two steps, with the first involving the Steering Committee and the second involving a full Delphi Panel (the ACCORD Delphi Panel; Fig. 3). We plan to report the consensus methodology in accordance with our own guidelines under development.

First step: steering committee survey

The Steering Committee will review the data extracted from literature search. This initial list is likely to contain duplicated items or items that require rewording. The aim is to eliminate repetitions and inadequately or ambiguously written items to reach a list of unique items. Using a survey, the Steering Committee members involved in the literature review will independently suggest items for the initial checklist; NH and WG will consolidate the initial checklist items.

There will then be anonymous voting to confirm the initial checklist that will be put to the full ACCORD consensus panel. Steering Committee members (excluding NH and WG) will vote (anonymised and blinded) on whether they 'Strongly Agree', 'Agree', 'Disagree', 'Strongly Disagree', or feel 'Abstained/Unable to answer' for all proposed items. There will also be the opportunity to provide comments. Any items that do not receive support will be discussed by the Steering Committee, and either included as 'possible additional items' or discarded completely. The eliminated items and the reasons for their

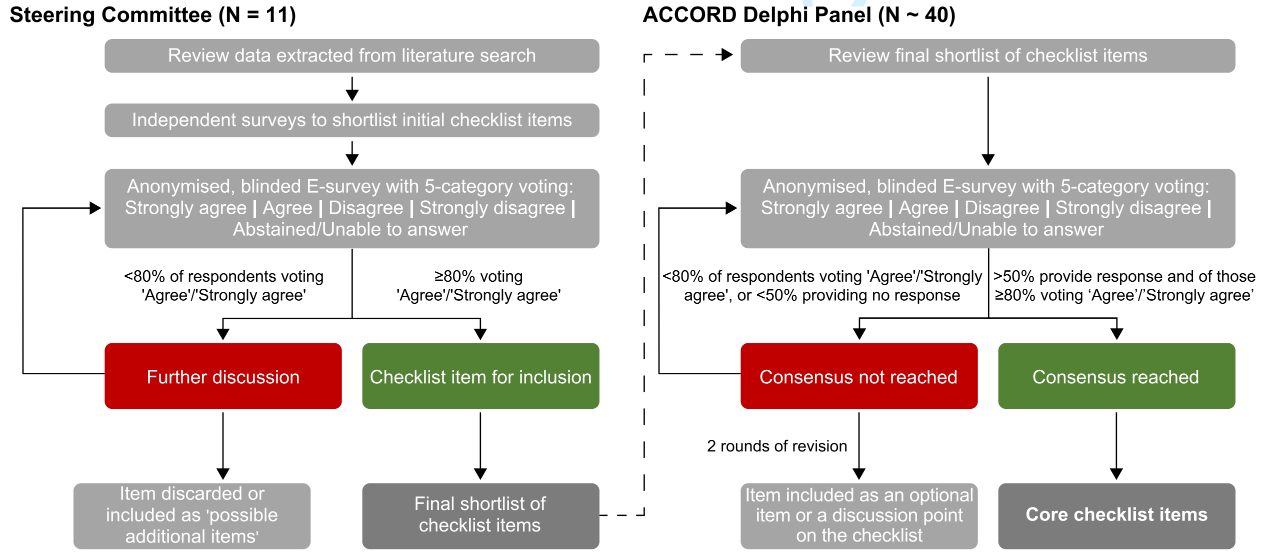

Fig. 3 Methodology used by the ACCORD Steering Committee and ACCORD Delphi Panel to achieve consensus on core checklist items for a consensus reporting guideline

For peer review only - http://bmjopen.bmj.com/site/about/guidelines.xhtml

elimination will be reported. The candidate items will be presented in sequence as a draft checklist, and in the same order to all people voting, so that the overall checklist structure, considering the manuscript sections (such as Introduction, Methods, Results, Discussion) can be evaluated. Within each section, there will be 'proposed items' and 'possible additional items'.

Second step: ACCORD Delphi panel

The preliminary list of checklist items agreed on by the Steering Committee will subsequently be put to the ACCORD Delphi Panel for validation using a blinded electronic voting platform (e-survey). In addition, the ACCORD Delphi Panel will be provided with the list of items excluded by the Steering Committee for information, as a confirmatory step.

The order of the candidate items within each manuscript section will be randomised so that it is different for each person voting and all items are evaluated fully independently from each other. Five voting options will be offered: 'Strongly Agree', 'Agree', 'Disagree', 'Strongly Disagree', and 'Abstained/Unable to answer'. Votes of 'Abstained/Unable to answer' will be included in the denominator. Panellists will be able to provide free-text comments and will have the opportunity to propose additional items. There will be three rounds of voting; with feedback and descriptive statistics incorporated for the next round by NH and WG. The approval rate and the reasons for elimination of items will be reported.

The consensus threshold is defined in this step as at least 20 respondents (approximately 50% of the target panel size), and at least 80% of responding ACCORD Delphi panellists who are able to answer voting 'Agree' or 'Strongly Agree', with two rounds of statement revision and re-voting. The Steering Committee will review items that do not achieve consensus in rounds 1 or 2 and these will be revised or eliminated taking into account any free-text comments. If consensus is not achieved by the ACCORD Delphi Panel, or there are insufficient respondents, the Steering Committee may decide that the item will be included as an optional item or a discussion point on the E&E document or checklist, alongside core items on which consensus was achieved. Simple descriptive statistics (response rates, level of agreement for each statement, median levels of agreement and interquartile ranges) will be used to describe approval rates between rounds. The same measures will be used to evaluate consensus stability across rounds [54].

There are no generally agreed standards for the panel size for Delphi studies, and a wide range of panel sizes has been reported; panels of 20–30 participants are common [55, 56]. However, it is recognised that the size and diversity of a Delphi panel can impact the quality of the final recommendations [57]. The ACCORD Delphi Panel will comprise approximately 40 members, so that it allows for representation from clinicians, methodologists, patient advocates, lay public representatives, health technologists, journal editors and publishers, regulatory specialists, and publications professionals, and to ensure an acceptable number of responses (20, or at least 50% of the group) in the event of drop-outs or partial completion of review. The ACCORD project will be advertised to potential Delphi Panellists via relevant societies, organisations, and networks; in addition, authors of recently published consensus studies in high-profile journals will be invited directly.

When registering, panellists will be asked to complete a preliminary survey to capture basic information on experience, geographical, and demographic representation. Although no formal targets will be established, the Steering Committee will endeavour to ensure a broad spread of representation across these categories. Members of the Delphi Panel will be recognised as contributors in the acknowledgements section of the guideline (with their permission) but participation in ACCORD Delphi panel will not qualify a panellist for authorship.

Software or a voting platform that is appropriate for Delphi exercises will be used to implement the voting process, administered by NH and WG. Alternatives available on the market are being evaluated and tested at the time of this protocol publication, and the platform and version used will be reported. Initial requirements are that the software used follows security regulations, ethical standards and allows, besides voting, the inclusion of free text responses in the e-surveys to supplement discussion in the E&E document.

Stage 4: creation of the reporting guideline and E&E document

On completion of the Delphi consensus process, the checklist will be finalised by WG and NH for approval by the Steering Committee, and the reporting guideline will be developed. A separate E&E document will be created to provide a detailed rationale for the items included in the checklist. In each case, an example will be included of good reporting from a published paper. The E&E document can also be informed by perspectives collected from researchers involved in consensus-based studies outside the biomedical field.

Stage 5: dissemination

We intend to publish the reporting guideline and E&E document in open access format via a CC-BY copyright licence. Future publications from the ACCORD project will be reported according to the best available reporting guidelines for each type of manuscript.

For peer review only - http://bmjopen.bmj.com/site/about/guidelines.xhtml

To aid dissemination, we plan to present the findings at congresses including ISMPP European and Annual Meetings, the World Conference on Research Integrity and Peer Review, and the UK Research Integrity Office Annual Conference. Progress will be updated on a dedicated website for the ACCORD project, the EQUATOR website and newsletter, and social media channels, and communicated in appropriate professional forums and events. This dissemination of the reporting guideline is crucial for the document to be implemented in practice.

Discussion

The ACCORD reporting guideline will provide a set of minimum items that should be reported about methods used to achieve consensus in biomedical research and guidance, including processes ranging from simple unstructured opinion gatherings to highly structured processes. The objective is to systematically develop a reporting guideline to help the biomedical research and clinical practice community describe the methods or techniques used to reach consensus in a complete, transparent, and consistent manner.

Extensions of the ACCORD reporting guideline and checklist could potentially be developed in the future to cover consensus studies in the non-biomedical sectors, with appropriate input from experts in those sectors to account for characteristics specific to each field. Our objective is to increase the completeness, transparency and consistency of the reporting of consensus methodology and, as a result, to improve the trustworthiness of recommendations developed using consensus methods. The Steering Committee welcomes enquiries from individuals interested in participating in the ACCORD Delphi Panel.

Supplementary Information

The online version contains supplementary material available at https://doi.org/10.1186/s41073-022-00122-0.

Additional file 1.

Acknowledgements

We thank Bernd Arents, who joined the Steering Committee and provided valuable input into the project but then had to step down because of other commitments. Medical writing support was provided by Sreerekha Pillai and Luke Worley of Ogilvy Health. Wendy Hazard of ISMPP provided administrative support. Jan Schoones (Leiden University Medical Center) assisted in development of the search strategy and Zbys Fedorowicz provided support for screening of hits from the systematic searches.

Authors' contributions

NH and WG recruited the ACCORD Steering Committee and coordinated the development, drafting, and review of this protocol. All authors contributed to the development of the protocol, reviewed, commented and approved the draft manuscript. The authors, except for the NH and WG, are listed in alphabetical order.

Funding

Ipsen provided access to full-text articles that were not otherwise freely available to support the development of this protocol and provided the open access processing charge. Ogilvy Health provided medical writing support.

Availability of data and materials

Anonymised aggregated data will be deposited in the Open Science Framework, where the study protocol has already been registered (https://osf.io/2rzm9). Individual responses to Delphi rounds will be deidentified at the source level by the platform used. These individual responses and approval rates can be requested to the corresponding author. The ACCORD protocol has been listed on the EQUATOR website and pre-registered with the Open Science Framework.

Declarations

Ethics approval and consent to participate

Not applicable.

Consent for publication

Not applicable.

Competing interests

PL is a member of the UK EQUATOR Centre, an organisation that promotes the use of reporting guidelines, many of which are developed using consensus methods, and she is personally involved in the development of other reporting guidelines. WG is a former employee of Ipsen and is now employed by Bristol Myers Squib. KG is an employee of AbbVie. APH, in the last five years, worked with Reckitt Benckiser for the development of the definitions and management of gastro-oesophageal reflux disease. CCW is an employee, Director, and shareholder of Oxford PharmaGenesis Ltd., a Director of Oxford Health Policy Forum CIC, a Trustee of the Friends of the National Library of Medicine, and an Associate Fellow of Green Templeton College. NH is an employee of Ogilvy Health UK. EH has worked with Ogilvy Health UK on consensus projects. AP, DT, RM and EJvZ have no conflict of interest.

Author details

[1]Global Medical Affairs, Ipsen, Abingdon, UK. [2]Faculty of Medical Sciences, Newcastle University, Newcastle, UK. [3]Stanford Anesthesia, Informatics and Media Lab, Stanford University School of Medicine, Stanford, CA, USA. [4]Oxford PharmaGenesis, Tubney, Oxford, UK. [5]Journal of Clinical Epidemiology, Sussex, UK. [6]Sciwright Limited, Somerset, UK. [7]Leiden University Medical Centre, Leiden, The Netherlands. [8]Global Medical Affairs, AbbVie, North Chicago, IL, USA. [9]Centre for Statistics in Medicine (CSM), University of Oxford, and EQUATOR Network UK Centre, Oxford, UK. [10]International Society for Medical Publication Professionals, New York, NY, USA. [11]Ogilvy Health, London, UK.

Received: 15 October 2021 Accepted: 9 May 2022
Published online: 07 June 2022

References

1. Szajewska H. Evidence-based medicine and clinical research: both are needed, Neither Is Perfect. Ann Nutr Metab. 2018;72(Suppl 3):13–23. https://doi.org/10.1159/000487375.
2. Barton S. Which clinical studies provide the best evidence? The best RCT still trumps the best observational study. BMJ. 2000;321(7256):255–6. https://doi.org/10.1136/bmj.321.7256.255.
3. Gopalakrishnan S, Ganeshkumar P. Systematic reviews and Meta-analysis: understanding the best evidence in primary healthcare. J Family Med Prim Care. 2013;2(1):9–14. https://doi.org/10.4103/2249-4863.109934.
4. Greenhalgh T. Will COVID-19 be evidence-based medicine's nemesis? PLoS Med. 2020;17(6):e1003266. https://doi.org/10.1371/journal.pmed.1003266.
5. Kempf L, Goldsmith JC, Temple R. Challenges of developing and conducting clinical trials in rare disorders. Am J Med Genet A. 2018;176(4):773–83. https://doi.org/10.1002/ajmg.a.38413.

6. Frieden TR. Evidence for health decision making - beyond randomized, controlled trials. N Engl J Med. 2017;377(5):465–75. https://doi.org/10.1056/NEJMra1614394.
7. Haw WY, Al-Janabi A, Arents BWM, Asfour L, Exton LS, Grindlay D, et al. Global guidelines in dermatology mapping project (GUIDEMAP): a scoping review of dermatology clinical practice guidelines. Br J Dermatol. 2021;185(4):467–75. https://doi.org/10.1111/bjd.20428.
8. McMillan SS, King M, Tully MP. How to use the nominal group and Delphi techniques. Int J Clin Pharm. 2016;38(3):655–62. https://doi.org/10.1007/s11096-016-0257-x.
9. Raine R, Sanderson C, Black N. Developing clinical guidelines: a challenge to current methods. BMJ. 2005;331(7517):631–3. https://doi.org/10.1136/bmj.331.7517.631.
10. Djulbegovic B, Guyatt G. Evidence vs consensus in clinical practice guidelines. JAMA. 2019;322(8):725–6. https://doi.org/10.1001/jama.2019.9751.
11. Moher D, Schulz KF, Simera I, Altman DG. Guidance for developers of health research reporting guidelines. PLoS Med. 2010;7(2):e1000217. https://doi.org/10.1371/journal.pmed.1000217.
12. Fink-Hafner D, Dagen T, Dousak M, Novak M, Hafner-Fink M. Delphi method: strengths and weaknesses. Metodoloski Zvezki. 2019;16(2):1–19 http://www.dlib.si/?URN=URN:NBN:SI:DOC-4UR1RO95.
13. Arakawa N, Bader LR. Consensus development methods: considerations for national and global frameworks and policy development. Res Soc Adm Pharm. 2021;18(1):2222–9. https://doi.org/10.1016/j.sapharm.2021.06.024.
14. Dalkey N, Helmer O. An experimental application of the DELPHI method to the use of experts. Manag Sci. 1963;9(3):458–67. https://doi.org/10.1287/mnsc.9.3.458.
15. Murphy MK, Black NA, Lamping DL, McKee CM, Sanderson CFB, Askham J, et al. Consensus development methods, and their use in clinical guideline development. Health Technol Assess. 1998;2(3):1–88. https://doi.org/10.3310/hta2030.
16. Foth T, Efstathiou N, Vanderspank-Wright B, Ufholz LA, Dutthorn N, Zimansky M, et al. The use of Delphi and nominal group technique in nursing education: a review. Int J Nurs Stud. 2016;60:112–20. https://doi.org/10.1016/j.ijnurstu.2016.04.015.
17. Nair R, Aggarwal R, Khanna D. Methods of formal consensus in classification/diagnostic criteria and guideline development. Semin Arthritis Rheum. 2011;41(2):95–105. https://doi.org/10.1016/j.semarthrit.2010.12.001.
18. Grant S, Armstrong C, Khodyakov D. Online modified-Delphi: a potential method for continuous patient engagement across stages of clinical practice guideline development. J Gen Intern Med. 2021;36(6):1746–50. https://doi.org/10.1007/s11606-020-06514-6.
19. Helmer-Hirshberg O. Analysis of the future: the Delphi method. Santa Monica: The RAND Corporation; 1967. https://www.rand.org/pubs/papers/P3558.html
20. Fink A, Kosecoff J, Chassin M, Brook RH. Consensus methods: characteristics and guidelines for use. Am J Public Health. 1984;74(9):979–83. https://doi.org/10.2105/ajph.74.9.979.
21. Halcomb E, Davidson P, Hardaker L. Using the consensus development conference method in healthcare research. Nurs Res. 2008;16(1):56–71. https://doi.org/10.7748/nr2008.10.16.1.56.c6753.
22. James D, Warren-Forward H. Research methods for formal consensus development. Nurs Res. 2015;22(3):35–40. https://doi.org/10.7748/nr.22.3.35.e1297.
23. Campbell SM, Cantrill JA. Consensus methods in prescribing research. J Clin Pharm Ther. 2001;26(1):5–14. https://doi.org/10.1046/j.1365-2710.2001.00331.x.
24. Drumm S, Bradley C, Moriarty F. 'More of an art than a science'? The development, design and mechanics of the Delphi technique. Res Soc Adm Pharm. 2022;18(1):2230–6. https://doi.org/10.1016/j.sapharm.2021.06.027.
25. Durkin J, Usher K, Jackson D. Using consensus from experts to inform a shared understanding of subjective terms. Nurs Res. 2019;27(2):46–9. https://doi.org/10.7748/nr.2019.e1622.
26. Franklin KK, Hart JK. Idea generation and exploration: benefits and limitations of the policy Delphi research method. Innov High Educ. 2007;31:237–46. https://doi.org/10.1007/s10755-006-9022-8
27. Guzys D, Dickson-Swift V, Kenny A, Threlkeld G. Gadamerian philosophical hermeneutics as a useful methodological framework for the Delphi technique. Int J Qual Stud Health Well-being. 2015;10:26291. https://doi.org/10.3402/qhw.v10.26291.
28. Keeney S, Hasson F, McKenna H. Consulting the oracle: ten lessons from using the Delphi technique in nursing research. J Adv Nurs. 2006;53(2):205–12. https://doi.org/10.1111/j.1365-2648.2006.03716.x.
29. Khodyakov D, Grant S, Denger B, Kinnett K, Martin A, Peay H, et al. Practical considerations in using online modified-Delphi approaches to engage patients and other stakeholders in clinical practice guideline development. Patient. 2020;13(1):11–21. https://doi.org/10.1007/s40271-019-00389-4.
30. Hallowell MR. Qualitative research: application of the Delphi method to CEM research. J Constr Eng Manag. 2010;136(1):99–107. https://doi.org/10.1061/(ASCE)CO.1943-7862.0000137.
31. Hsu CC, Sandford BA. The Delphi technique: making sense of consensus. Pract Assess Res Eval. 2007;12(10):1–8. https://doi.org/10.7275/pdz9-th90.
32. Hsieh CJ, Fific M, Yang CT. A new measure of group decision-making efficiency. Cogn Res Princ Implic. 2020;5(1):45. https://doi.org/10.1186/s41235-020-00244-3.
33. Beiderbeck D, Frevel N, von der Gracht HA, Schmidt SL, Schweitzer VM. Preparing, conducting, and analyzing Delphi surveys: cross-disciplinary practices, new directions, and advancements. MethodsX. 2021;8:101401. https://doi.org/10.1016/j.mex.2021.101401.
34. Li S-A, Yousefi-Nooraie R, Guyatt G, Talwar G, Wang Q, Zhu Y, et al. A few panel members dominated guideline development meeting discussions: a social network analysis. J Clin Epidemiol. 2022;141:1–10. https://doi.org/10.1016/j.jclinepi.2021.09.023.
35. Hohmann E, Brand JC, Rossi MJ, Lubowitz JH. Expert opinion is necessary: Delphi panel methodology facilitates a scientific approach to consensus. Arthroscopy. 2018;34(2):349–51. https://doi.org/10.1016/j.arthro.2017.11.022.
36. Humphrey-Murto S, Varpio L, Gonsalves C, Wood TJ. Using consensus group methods such as Delphi and nominal group in medical education research. Med Teach. 2017;39(1):14–9. https://doi.org/10.1080/0142159X.2017.1245856.
37. Grant S, Booth M, Khodyakov D. Lack of preregistered analysis plans allows unacceptable data mining for and selective reporting of consensus in Delphi studies. J Clin Epidemiol. 2018;99:96–105. https://doi.org/10.1016/j.jclinepi.2018.03.007.
38. Jünger S, Payne SA, Brine J, Radbruch L, Brearley SG. Guidance on conducting and REporting DElphi studies (CREDES) in palliative care: recommendations based on a methodological systematic review. Palliat Med. 2017;31(8):684–706. https://doi.org/10.1177/0269216317690685.
39. Hohmann E, Cote MP, Brand JC. Research pearls: expert consensus based evidence using the Delphi method. Arthroscopy. 2018;34(12):3278–82. https://doi.org/10.1016/j.arthro.2018.10.004.
40. Humphrey-Murto S, Varpio L, Wood TJ, Gonsalves C, Ufholz LA, Mascioli K, et al. The use of the Delphi and other consensus group methods in medical education research: a review. Acad Med. 2017;92(10):1491–8. https://doi.org/10.1097/ACM.0000000000001812.
41. Gupta UG, Clarke RE. Theory and applications of the Delphi technique: a bibliography (1975–1994). Technol Forecast Soc Change. 1996;53(2):185–211. https://doi.org/10.1016/S0040-1625(96)00094-7.
42. Veugelers R, Gaakeer MI, Patka P, Huijsman R. Improving design choices in Delphi studies in medicine: the case of an exemplary physician multi-round panel study with 100% response. BMC Med Res Methodol. 2020;20(1):156. https://doi.org/10.1186/s12874-020-01029-4.
43. Diamond IR, Grant RC, Feldman BM, Pencharz PB, Ling SC, Moore AM, et al. Defining consensus: a systematic review recommends methodologic criteria for reporting of Delphi studies. J Clin Epidemiol. 2014;67(4):401–9. https://doi.org/10.1016/j.jclinepi.2013.12.002.
44. Paré G, Cameron A-F, Poba-Nzaou P, Templier M. A systematic assessment of rigor in information systems ranking-type Delphi studies. Inf Manag. 2013;50(5):207–17. https://doi.org/10.1016/j.im.2013.03.003.
45. Gattrell WT, Clements SJ, Sheard D. Quality assessment of guidelines/recommendations developed using Delphi methodology. Curr Med Res Opin. 2019;35(Suppl 2):40. https://doi.org/10.1080/03007995.2019.1583496.
46. Thompson M. Considering the implication of variations within Delphi research. Fam Pract. 2009;26(5):420–4. https://doi.org/10.1093/fampra/cmp051.

For peer review only

47. Schlussel M, Collins G, de Beyer JA, Kirtley S. EQUATOR Library Audit. Available from: https://osf.io/fuv3c. Accessed 21 Jan 2022.
48. Banno M, Tsujimoto Y, Kataoka Y. The majority of reporting guidelines are not developed with the Delphi method: a systematic review of reporting guidelines. J Clin Epidemiol. 2020;124:50–7. https://doi.org/10.1016/j.jclinepi.2020.04.010.
49. Brouwers MC, Kho ME, Browman GP, Burgers JS, Cluzeau F, Feder G, et al. AGREE II: advancing guideline development, reporting and evaluation in health care. CMAJ. 2010;182(18):E839–42. https://doi.org/10.1503/cmaj.090449.
50. EQUATOR Network. Available from: equator-network.org. Accessed 17 Sept 2021.
51. Open Science Framework Registries. Available from: https://osf.io/2rzm9. Accessed 07 Mar 2022.
52. Rethlefsen ML, Kirtley S, Waffenschmidt S, Ayala AP, Moher D, Page MJ, et al. PRISMA-S: an extension to the PRISMA statement for reporting literature searches in systematic reviews. Syst Rev. 2021;10(1):39. https://doi.org/10.1186/s13643-020-01542-z.
53. Ouzzani M, Hammady H, Fedorowicz Z, Elmagarmid A. Rayyan—a web and mobile app for systematic reviews. Syst Rev. 2016;5(1):210. https://doi.org/10.1186/s13643-016-0384-4.
54. Holey EA, Feeley JL, Dixon J, Whittaker VJ. An exploration of the use of simple statistics to measure consensus and stability in Delphi studies. BMC Med Res Methodol. 2007;7(1):52. https://doi.org/10.1186/1471-2288-7-52.
55. Akins RB, Tolson H, Cole BR. Stability of response characteristics of a Delphi panel: application of bootstrap data expansion. BMC Med Res Methodol. 2005;5:37. https://doi.org/10.1186/1471-2288-5-37.
56. Chalmers J, Armour M. The Delphi technique. In handbook of research methods in health social sciences. Singapore: Springer; 2019. p. 715–35. https://doi.org/10.1007/978-981-10-5251-4_99.
57. Birko S, Dove ES, Ozdemir V. Evaluation of nine consensus indices in Delphi foresight research and their dependency on Delphi survey characteristics: a simulation study and debate on Delphi design and interpretation. PLoS One. 2015;10(8):e0135162. https://doi.org/10.1371/journal.pone.0135162.

Publisher's Note

Springer Nature remains neutral with regard to jurisdictional claims in published maps and institutional affiliations.

Ready to submit your research? Choose BMC and benefit from:

- fast, convenient online submission
- thorough peer review by experienced researchers in your field
- rapid publication on acceptance
- support for research data, including large and complex data types
- gold Open Access which fosters wider collaboration and increased citations
- maximum visibility for your research: over 100M website views per year

At BMC, research is always in progress.

Learn more biomedcentral.com/submissions

BMC

For peer review only

ACCORD - January 7th, 2022

Regular references:

Total: 2599 references, sourced from:

- Web of Science - core collection: 1775
- MEDLINE (Web of Science): 1501 - 202 unique
- PubMed: 375 - 219 unique
- MEDLINE (OVID): 641 - 174 unique
- Embase (OVID): 331 - 66 unique
- Cochrane Library: 131 - 77 unique
- Emcare (OVID): 179 - 29 unique
- Academic Search Premier: 280 - 23 unique
- PsycINFO: 173 - 34 unique

Meeting abstract references:

Total: 137 references, sourced from:

- Web of Science: 14
- Embase (OVID): 99 - 90 unique
- Cochrane Library: 36 - 33 unique

Known references:

- PubMed: 27841062 26796090 25587865 26395179 24581294
- MEDLINE (Web of Science): PMID=(27841062 OR 26796090 OR 25587865 OR 26395179 OR 24581294)
- Web of Science Core Collection: UT=(000393885800003 OR 000375153500022 OR 000376181900007 OR 000361506400001 OR 000334256400007 OR 000309802600012 OR 000321232400002 OR 000309802600012 OR 000465105500070)

Databases:

Web of Science Core Collection and MEDLINE (Web of Science)
http://isiknowledge.com/wos

((TI=("Delphi Technique" OR "Delphi Technique" OR "Delphi techniques" OR "Delphi method" OR "Delphi methods" OR "Delphi study" OR "Delphi studies" OR "Delphi survey" OR "Delphi surveys" OR "Delphi consensus" OR "Delphi based consensus" OR "Delphi questionnaire" OR "Delphi questionnaires" OR "Delphi research" OR "Delphi review" OR "Delphi reviews" OR "Delphi process" OR "Delphi processes" OR "Delphi based" OR "Delphi procedure" OR "Delphi procedures" OR "Delphi assessment" OR "Delphi assessments" OR "Delphi approach" OR "Delphi approaches" OR "Delphi panel" OR "Delphi panels" OR "Delphi round" OR "Delphi rounds" OR "Delphi analysis" OR

For peer review only

"Delphi expert" OR "Delphi experts" OR "Delphi consultation" OR "Delphi methodology" OR "nominal group technique" OR "nominal group techniques" OR "nominal group" OR "nominal groups" OR "nominal grouping" OR "consensus recommendation" OR "consensus recommendations" OR "consensus development" OR "consensus activity" OR "consensus activities" OR "Consensus Development Conference" OR "Consensus Development" OR "Consensus methodology" OR "consensus method*" OR "RAND" OR (("Guidelines" OR "guideline") NEAR/2 ("consensus" OR "delphi"))) OR AB=("Delphi Technique" OR "Delphi Technique" OR "Delphi techniques" OR "Delphi method" OR "Delphi methods" OR "Delphi study" OR "Delphi studies" OR "Delphi survey" OR "Delphi surveys" OR "Delphi consensus" OR "Delphi based consensus" OR "Delphi questionnaire" OR "Delphi questionnaires" OR "Delphi research" OR "Delphi review" OR "Delphi reviews" OR "Delphi process" OR "Delphi processes" OR "Delphi based" OR "Delphi procedure" OR "Delphi procedures" OR "Delphi assessment" OR "Delphi assessments" OR "Delphi approach" OR "Delphi approaches" OR "Delphi panel" OR "Delphi panels" OR "Delphi round" OR "Delphi rounds" OR "Delphi analysis" OR "Delphi expert" OR "Delphi experts" OR "Delphi consultation" OR "Delphi methodology" OR "nominal group technique" OR "nominal group techniques" OR "nominal group" OR "nominal groups" OR "nominal grouping" OR "consensus recommendation" OR "consensus recommendations" OR "consensus development" OR "consensus activity" OR "consensus activities" OR "Consensus Development Conference" OR "Consensus Development" OR "Consensus methodology" OR "consensus method*" OR "RAND") OR AK=("Delphi Technique" OR "Delphi Technique" OR "Delphi techniques" OR "Delphi method" OR "Delphi methods" OR "Delphi study" OR "Delphi studies" OR "Delphi survey" OR "Delphi surveys" OR "Delphi consensus" OR "Delphi based consensus" OR "Delphi questionnaire" OR "Delphi questionnaires" OR "Delphi research" OR "Delphi review" OR "Delphi reviews" OR "Delphi process" OR "Delphi processes" OR "Delphi based" OR "Delphi procedure" OR "Delphi procedures" OR "Delphi assessment" OR "Delphi assessments" OR "Delphi approach" OR "Delphi approaches" OR "Delphi panel" OR "Delphi panels" OR "Delphi round" OR "Delphi rounds" OR "Delphi analysis" OR "Delphi expert" OR "Delphi experts" OR "Delphi consultation" OR "Delphi methodology" OR "nominal group technique" OR "nominal group techniques" OR "nominal group" OR "nominal groups" OR "nominal grouping" OR "consensus recommendation" OR "consensus recommendations" OR "consensus development" OR "consensus activity" OR "consensus activities" OR "Consensus Development Conference" OR "Consensus Development" OR "Consensus methodology" OR "consensus method*" OR "RAND" OR (("Guidelines" OR "guideline") NEAR/2 ("consensus" OR "delphi")))) AND (TI=("quality of reporting" OR "reporting quality" OR "reporting qualities" OR "selective reporting" OR "poor reporting" OR "poor reported" OR "reporting guideline" OR "reporting" OR ("reporting" AND ("quality" OR "selective" OR "poor" OR "weak" OR "manner" OR "rigor" OR "improv*")) OR "Data Accuracy" OR "quality assessment" OR "strengths" OR "strength" OR "weaknesses" OR "weakness" OR "research method" OR "research methods" OR "research method*") OR AK=("quality of reporting" OR "reporting quality" OR "reporting qualities" OR "selective reporting" OR "poor reporting" OR "poor reported" OR ("reporting" NEAR/5 ("quality" OR "selective" OR "poor" OR "weak" OR "manner" OR "rigor" OR "improv*")) OR "Data Accuracy" OR

"Research Report standards" OR "quality assessment" OR "strengths" OR "strength" OR "weaknesses" OR "weakness" OR "research method" OR "research methods" OR "research method*") OR AB=("quality of reporting" OR "reporting quality" OR "reporting qualities" OR "selective reporting" OR "poor reporting" OR "poor reported" OR ("reporting" NEAR/5 ("quality" OR "selective" OR "poor" OR "weak" OR "manner" OR "rigor" OR "improv*")) OR "Data Accuracy" OR "Research Report standards" OR "quality assessment" OR "strengths" OR "strength" OR "weaknesses" OR "weakness")))

PubMed
http://www.ncbi.nlm.nih.gov/pubmed?otool=leiden

(("Delphi Technique"[majr] OR "Delphi Technique"[ti] OR "Delphi techniques"[ti] OR "Delphi method"[ti] OR "Delphi methods"[ti] OR "Delphi study"[ti] OR "Delphi studies"[ti] OR "Delphi survey"[ti] OR "Delphi surveys"[ti] OR "Delphi consensus"[ti] OR "Delphi based consensus"[ti] OR "Delphi questionnaire"[ti] OR "Delphi questionnaires"[ti] OR "Delphi research"[ti] OR "Delphi review"[ti] OR "Delphi reviews"[ti] OR "Delphi process"[ti] OR "Delphi processes"[ti] OR "Delphi based"[ti] OR "Delphi procedure"[ti] OR "Delphi procedures"[ti] OR "Delphi assessment"[ti] OR "Delphi assessments"[ti] OR "Delphi approach"[ti] OR "Delphi approaches"[ti] OR "Delphi panel"[ti] OR "Delphi panels"[ti] OR "Delphi round"[ti] OR "Delphi rounds"[ti] OR "Delphi analysis"[ti] OR "Delphi expert"[ti] OR "Delphi experts"[ti] OR "Delphi consultation"[ti] OR "Delphi methodology"[ti] OR "nominal group technique"[ti] OR "nominal group techniques"[ti] OR "nominal group"[ti] OR "nominal groups"[ti] OR "nominal grouping"[ti] OR "consensus recommendation"[ti] OR "consensus recommendations"[ti] OR "consensus development"[ti] OR "consensus activity"[ti] OR "consensus activities"[ti] OR "consensus methodology"[ti] OR "consensus method*"[ti] OR "Consensus Development Conferences as Topic"[majr] OR "RAND"[ti] OR ("Guidelines as Topic"[majr:noexp] AND ("consensus"[tw] OR "delphi"[tw]))) AND ("reporting"[ti] OR "quality of reporting"[tw] OR "reporting quality"[tw] OR "reporting qualities"[tw] OR "selective reporting"[tw] OR "poor reporting"[tw] OR "poor reported"[tw] OR "poorly reported"[tw] OR "Research Report/standards"[majr] OR "Research Design/standards"[mesh] OR "Research Design"[majr:noexp] OR "Writing/standards"[mesh] OR "Writing"[majr] OR "research method"[ti] OR "research methods"[ti] OR "research method*"[ti]))

MEDLINE via OVID
http://gateway.ovid.com/ovidweb.cgi?T=JS&MODE=ovid&NEWS=n&PAGE=main&D=medall

((exp *"Delphi Technique"/ OR "Delphi Technique".ti OR "Delphi techniques".ti OR "Delphi method".ti OR "Delphi methods".ti OR "Delphi study".ti OR "Delphi studies".ti OR "Delphi survey".ti OR "Delphi surveys".ti OR "Delphi consensus".ti OR "Delphi based consensus".ti OR "Delphi questionnaire".ti OR "Delphi questionnaires".ti OR "Delphi research".ti OR "Delphi review".ti OR "Delphi reviews".ti OR "Delphi

process".ti OR "Delphi processes".ti OR "Delphi based".ti OR "Delphi procedure".ti OR "Delphi procedures".ti OR "Delphi assessment".ti OR "Delphi assessments".ti OR "Delphi approach".ti OR "Delphi approaches".ti OR "Delphi panel".ti OR "Delphi panels".ti OR "Delphi round".ti OR "Delphi rounds".ti OR "Delphi analysis".ti OR "Delphi expert".ti OR "Delphi experts".ti OR "Delphi consultation".ti OR "Delphi methodology".ti OR "nominal group technique".ti OR "nominal group techniques".ti OR "nominal group".ti OR "nominal groups".ti OR "nominal grouping".ti OR "consensus recommendation".ti OR "consensus recommendations".ti OR "consensus development".ti OR "consensus activity".ti OR "consensus activities".ti OR "consensus methodology".ti OR "consensus method*".ti OR exp *"Consensus Development Conferences as Topic"/ OR "RAND".ti OR ("Guidelines as Topic"/ AND ("consensus".mp OR "delphi".mp)) OR (("Guidelines".mp OR "guideline".mp) ADJ2 ("consensus".mp OR "delphi".mp))) AND ("reporting".ti OR "quality of reporting".mp OR "reporting quality".mp OR "reporting qualities".mp OR "selective reporting".mp OR "poor reporting".mp OR "poor reported".mp OR "poorly reported".mp OR "Research Report/standards"/ OR exp "Research Design"/st OR *"Research Design"/ OR exp "Writing"/st OR exp *"Writing"/ OR "research method".ti OR "research methods".ti OR "research method*".ti OR ("reporting" ADJ8 ("quality" OR "selective" OR "poor" OR "weak" OR "manner" OR "rigor" OR "improv*")).mp))

Embase
http://ovidsp.ovid.com/ovidweb.cgi?T=JS&PAGE=main&MODE=ovid&D=oemezd

((exp *"Delphi Study"/ OR "Delphi Technique".ti OR "Delphi techniques".ti OR "Delphi method".ti OR "Delphi methods".ti OR "Delphi study".ti OR "Delphi studies".ti OR "Delphi survey".ti OR "Delphi surveys".ti OR "Delphi consensus".ti OR "Delphi based consensus".ti OR "Delphi questionnaire".ti OR "Delphi questionnaires".ti OR "Delphi research".ti OR "Delphi review".ti OR "Delphi reviews".ti OR "Delphi process".ti OR "Delphi processes".ti OR "Delphi based".ti OR "Delphi procedure".ti OR "Delphi procedures".ti OR "Delphi assessment".ti OR "Delphi assessments".ti OR "Delphi approach".ti OR "Delphi approaches".ti OR "Delphi panel".ti OR "Delphi panels".ti OR "Delphi round".ti OR "Delphi rounds".ti OR "Delphi analysis".ti OR "Delphi expert".ti OR "Delphi experts".ti OR "Delphi consultation".ti OR "Delphi methodology".ti OR "nominal group technique".ti OR "nominal group techniques".ti OR "nominal group".ti OR "nominal groups".ti OR "nominal grouping".ti OR "consensus recommendation".ti OR "consensus recommendations".ti OR "consensus development".ti OR "consensus activity".ti OR "consensus activities".ti OR "consensus methodology".ti OR "consensus method*".ti OR exp *"Consensus Development"/ OR "RAND".ti OR (("Guidelines".ti,ab OR "guideline".ti,ab) ADJ2 ("consensus".ti,ab OR "delphi".ti,ab))) AND ("reporting".ti OR "quality of reporting".ti,ab OR "reporting quality".ti,ab OR "reporting qualities".ti,ab OR "selective reporting".ti,ab OR "poor reporting".ti,ab OR "poor reported".ti,ab OR "poorly reported".ti,ab OR *"Methodology"/ OR *"Writing"/ OR "research method".ti OR "research methods".ti OR "research method*".ti OR ("reporting" ADJ8 ("quality" OR "selective" OR "poor" OR "weak" OR "manner" OR "rigor" OR "improv*")).ti,ab))

Cochrane
https://www.cochranelibrary.com/advanced-search/search-manager

(("Delphi Technique" OR "Delphi Technique" OR "Delphi techniques" OR "Delphi method" OR "Delphi methods" OR "Delphi study" OR "Delphi studies" OR "Delphi survey" OR "Delphi surveys" OR "Delphi consensus" OR "Delphi based consensus" OR "Delphi questionnaire" OR "Delphi questionnaires" OR "Delphi research" OR "Delphi review" OR "Delphi reviews" OR "Delphi process" OR "Delphi processes" OR "Delphi based" OR "Delphi procedure" OR "Delphi procedures" OR "Delphi assessment" OR "Delphi assessments" OR "Delphi approach" OR "Delphi approaches" OR "Delphi panel" OR "Delphi panels" OR "Delphi round" OR "Delphi rounds" OR "Delphi analysis" OR "Delphi expert" OR "Delphi experts" OR "Delphi consultation" OR "Delphi methodology" OR "nominal group technique" OR "nominal group techniques" OR "nominal group" OR "nominal groups" OR "nominal grouping" OR "consensus recommendation" OR "consensus recommendations" OR "consensus development" OR "consensus activity" OR "consensus activities" OR "Consensus Development Conference" OR "Consensus Development" OR "Consensus methodology" OR "consensus method*" OR "RAND" OR (("Guidelines" OR "guideline") NEAR/2 ("consensus" OR "delphi"))) AND ("quality of reporting" OR "reporting quality" OR "reporting qualities" OR "selective reporting" OR "poor reporting" OR "poor reported" OR ("reporting" NEAR/5 ("quality" OR "selective" OR "poor" OR "weak" OR "manner" OR "rigor" OR "improv*")) OR "Data Accuracy" OR "Research Report standards" OR "quality assessment" OR "strengths" OR "strength" OR "weaknesses" OR "weakness" OR "research method" OR "research methods" OR "research method*")):ti,ab,kw

Emcare
http://ovidsp.ovid.com/ovidweb.cgi?T=JS&NEWS=n&CSC=Y&PAGE=main&D=emcr

((exp *"Delphi Study"/ OR "Delphi Technique".ti OR "Delphi techniques".ti OR "Delphi method".ti OR "Delphi methods".ti OR "Delphi study".ti OR "Delphi studies".ti OR "Delphi survey".ti OR "Delphi surveys".ti OR "Delphi consensus".ti OR "Delphi based consensus".ti OR "Delphi questionnaire".ti OR "Delphi questionnaires".ti OR "Delphi research".ti OR "Delphi review".ti OR "Delphi reviews".ti OR "Delphi process".ti OR "Delphi processes".ti OR "Delphi based".ti OR "Delphi procedure".ti OR "Delphi procedures".ti OR "Delphi assessment".ti OR "Delphi assessments".ti OR "Delphi approach".ti OR "Delphi approaches".ti OR "Delphi panel".ti OR "Delphi panels".ti OR "Delphi round".ti OR "Delphi rounds".ti OR "Delphi analysis".ti OR "Delphi expert".ti OR "Delphi experts".ti OR "Delphi consultation".ti OR "Delphi methodology".ti OR "nominal group technique".ti OR "nominal group techniques".ti OR "nominal group".ti OR "nominal groups".ti OR "nominal grouping".ti OR "consensus recommendation".ti OR "consensus recommendations".ti OR "consensus development".ti OR "consensus activity".ti OR "consensus activities".ti OR "consensus methodology".ti OR "consensus method*".ti OR exp *"Consensus Development"/ OR "RAND".ti OR (("Guidelines".ti,ab OR "guideline".ti,ab) ADJ2 ("consensus".ti,ab OR "delphi".ti,ab))) AND ("reporting".ti

OR "quality of reporting".ti,ab OR "reporting quality".ti,ab OR "reporting qualities".ti,ab OR "selective reporting".ti,ab OR "poor reporting".ti,ab OR "poor reported".ti,ab OR "poorly reported".ti,ab OR *"Methodology"/ OR *"Writing"/ OR "research method".ti OR "research methods".ti OR "research method*".ti OR ("reporting" ADJ8 ("quality" OR "selective" OR "poor" OR "weak" OR "manner" OR "rigor" OR "improv*")).ti,ab))

Academic Search Premier
http://search.ebscohost.com/login.aspx?authtype=ip,uid&profile=lumc&defaultdb=aph

((TI("Delphi Technique" OR "Delphi Technique" OR "Delphi techniques" OR "Delphi method" OR "Delphi methods" OR "Delphi study" OR "Delphi studies" OR "Delphi survey" OR "Delphi surveys" OR "Delphi consensus" OR "Delphi based consensus" OR "Delphi questionnaire" OR "Delphi questionnaires" OR "Delphi research" OR "Delphi review" OR "Delphi reviews" OR "Delphi process" OR "Delphi processes" OR "Delphi based" OR "Delphi procedure" OR "Delphi procedures" OR "Delphi assessment" OR "Delphi assessments" OR "Delphi approach" OR "Delphi approaches" OR "Delphi panel" OR "Delphi panels" OR "Delphi round" OR "Delphi rounds" OR "Delphi analysis" OR "Delphi expert" OR "Delphi experts" OR "Delphi consultation" OR "Delphi methodology" OR "nominal group technique" OR "nominal group techniques" OR "nominal group" OR "nominal groups" OR "nominal grouping" OR "consensus recommendation" OR "consensus recommendations" OR "consensus development" OR "consensus activity" OR "consensus activities" OR "Consensus Development Conference" OR "Consensus Development" OR "Consensus methodology" OR "consensus method*" OR "RAND" OR (("Guidelines" OR "guideline") N2 ("consensus" OR "delphi"))) OR KW("Delphi Technique" OR "Delphi Technique" OR "Delphi techniques" OR "Delphi method" OR "Delphi methods" OR "Delphi study" OR "Delphi studies" OR "Delphi survey" OR "Delphi surveys" OR "Delphi consensus" OR "Delphi based consensus" OR "Delphi questionnaire" OR "Delphi questionnaires" OR "Delphi research" OR "Delphi review" OR "Delphi reviews" OR "Delphi process" OR "Delphi processes" OR "Delphi based" OR "Delphi procedure" OR "Delphi procedures" OR "Delphi assessment" OR "Delphi assessments" OR "Delphi approach" OR "Delphi approaches" OR "Delphi panel" OR "Delphi panels" OR "Delphi round" OR "Delphi rounds" OR "Delphi analysis" OR "Delphi expert" OR "Delphi experts" OR "Delphi consultation" OR "Delphi methodology" OR "nominal group technique" OR "nominal group techniques" OR "nominal group" OR "nominal groups" OR "nominal grouping" OR "consensus recommendation" OR "consensus recommendations" OR "consensus development" OR "consensus activity" OR "consensus activities" OR "Consensus Development Conference" OR "Consensus Development" OR "Consensus methodology" OR "consensus method*" OR "RAND" OR (("Guidelines" OR "guideline") N2 ("consensus" OR "delphi")))) AND (TI("quality of reporting" OR "reporting quality" OR "reporting qualities" OR "selective reporting" OR "poor reporting" OR "poor reported" OR "reporting guideline" OR "reporting" OR ("reporting" AND ("quality" OR "selective" OR "poor" OR "weak" OR "manner" OR "rigor" OR "improv*")) OR "Data Accuracy" OR "quality assessment" OR "strengths" OR "strength" OR "weaknesses" OR "weakness" OR "research method" OR "research methods" OR "research method*") OR

For peer review only

KW("quality of reporting" OR "reporting quality" OR "reporting qualities" OR "selective reporting" OR "poor reporting" OR "poor reported" OR ("reporting" N5 ("quality" OR "selective" OR "poor" OR "weak" OR "manner" OR "rigor" OR "improv*")) OR "Data Accuracy" OR "Research Report standards" OR "quality assessment" OR "strengths" OR "strength" OR "weaknesses" OR "weakness" OR "research method" OR "research methods" OR "research method*") OR AB("quality of reporting" OR "reporting quality" OR "reporting qualities" OR "selective reporting" OR "poor reporting" OR "poor reported" OR ("reporting" N5 ("quality" OR "selective" OR "poor" OR "weak" OR "manner" OR "rigor" OR "improv*")) OR "Data Accuracy" OR "Research Report standards" OR "quality assessment" OR "strengths" OR "strength" OR "weaknesses" OR "weakness"))

PsycINFO
http://search.ebscohost.com/login.aspx?authtype=ip,uid&profile=lumc&defaultdb=psyh

((TI("Delphi Technique" OR "Delphi Technique" OR "Delphi techniques" OR "Delphi method" OR "Delphi methods" OR "Delphi study" OR "Delphi studies" OR "Delphi survey" OR "Delphi surveys" OR "Delphi consensus" OR "Delphi based consensus" OR "Delphi questionnaire" OR "Delphi questionnaires" OR "Delphi research" OR "Delphi review" OR "Delphi reviews" OR "Delphi process" OR "Delphi processes" OR "Delphi based" OR "Delphi procedure" OR "Delphi procedures" OR "Delphi assessment" OR "Delphi assessments" OR "Delphi approach" OR "Delphi approaches" OR "Delphi panel" OR "Delphi panels" OR "Delphi round" OR "Delphi rounds" OR "Delphi analysis" OR "Delphi expert" OR "Delphi experts" OR "Delphi consultation" OR "Delphi methodology" OR "nominal group technique" OR "nominal group techniques" OR "nominal group" OR "nominal groups" OR "nominal grouping" OR "consensus recommendation" OR "consensus recommendations" OR "consensus development" OR "consensus activity" OR "consensus activities" OR "Consensus Development Conference" OR "Consensus Development" OR "Consensus methodology" OR "consensus method*" OR "RAND" OR (("Guidelines" OR "guideline") N2 ("consensus" OR "delphi"))) OR AB("Delphi Technique" OR "Delphi Technique" OR "Delphi techniques" OR "Delphi method" OR "Delphi methods" OR "Delphi study" OR "Delphi studies" OR "Delphi survey" OR "Delphi surveys" OR "Delphi consensus" OR "Delphi based consensus" OR "Delphi questionnaire" OR "Delphi questionnaires" OR "Delphi research" OR "Delphi review" OR "Delphi reviews" OR "Delphi process" OR "Delphi processes" OR "Delphi based" OR "Delphi procedure" OR "Delphi procedures" OR "Delphi assessment" OR "Delphi assessments" OR "Delphi approach" OR "Delphi approaches" OR "Delphi panel" OR "Delphi panels" OR "Delphi round" OR "Delphi rounds" OR "Delphi analysis" OR "Delphi expert" OR "Delphi experts" OR "Delphi consultation" OR "Delphi methodology" OR "nominal group technique" OR "nominal group techniques" OR "nominal group" OR "nominal groups" OR "nominal grouping" OR "consensus recommendation" OR "consensus recommendations" OR "consensus development" OR "consensus activity" OR "consensus activities" OR "Consensus Development Conference" OR "Consensus Development" OR "Consensus methodology" OR "consensus method*" OR "RAND") OR KW("Delphi Technique" OR "Delphi

For peer review only

Technique" OR "Delphi techniques" OR "Delphi method" OR "Delphi methods" OR "Delphi study" OR "Delphi studies" OR "Delphi survey" OR "Delphi surveys" OR "Delphi consensus" OR "Delphi based consensus" OR "Delphi questionnaire" OR "Delphi questionnaires" OR "Delphi research" OR "Delphi review" OR "Delphi reviews" OR "Delphi process" OR "Delphi processes" OR "Delphi based" OR "Delphi procedure" OR "Delphi procedures" OR "Delphi assessment" OR "Delphi assessments" OR "Delphi approach" OR "Delphi approaches" OR "Delphi panel" OR "Delphi panels" OR "Delphi round" OR "Delphi rounds" OR "Delphi analysis" OR "Delphi expert" OR "Delphi experts" OR "Delphi consultation" OR "Delphi methodology" OR "nominal group technique" OR "nominal group techniques" OR "nominal group" OR "nominal groups" OR "nominal grouping" OR "consensus recommendation" OR "consensus recommendations" OR "consensus development" OR "consensus activity" OR "consensus activities" OR "Consensus Development Conference" OR "Consensus Development" OR "Consensus methodology" OR "consensus method*" OR "RAND" OR (("Guidelines" OR "guideline") N2 ("consensus" OR "delphi")))) AND (TI("quality of reporting" OR "reporting quality" OR "reporting qualities" OR "selective reporting" OR "poor reporting" OR "poor reported" OR "reporting guideline" OR "reporting" OR ("reporting" AND ("quality" OR "selective" OR "poor" OR "weak" OR "manner" OR "rigor" OR "improv*")) OR "Data Accuracy" OR "quality assessment" OR "strengths" OR "strength" OR "weaknesses" OR "weakness" OR "research method" OR "research methods" OR "research method*") OR KW("quality of reporting" OR "reporting quality" OR "reporting qualities" OR "selective reporting" OR "poor reporting" OR "poor reported" OR ("reporting" N5 ("quality" OR "selective" OR "poor" OR "weak" OR "manner" OR "rigor" OR "improv*")) OR "Data Accuracy" OR "Research Report standards" OR "quality assessment" OR "strengths" OR "strength" OR "weaknesses" OR "weakness" OR "research method" OR "research methods" OR "research method*") OR AB("quality of reporting" OR "reporting quality" OR "reporting qualities" OR "selective reporting" OR "poor reporting" OR "poor reported" OR ("reporting" N5 ("quality" OR "selective" OR "poor" OR "weak" OR "manner" OR "rigor" OR "improv*")) OR "Data Accuracy" OR "Research Report standards" OR "quality assessment" OR "strengths" OR "strength" OR "weaknesses" OR "weakness")))

For peer review only

**Author, year**	
**Assessor**	

**Background**<br>1.1 Does the study suggest anything about how or if consensus papers should report the context or rationale for choosing a consensus method over other methods?	
**Background**<br>1.2 Does the study suggest anything about how/what or if consensus papers should report the objectives of the consensus exercise?	

**Methods**<br>2.1 Does the study suggest anything about how/what or if consensus papers should report regarding:<br>A literature search/strategy?	
**Methods**<br>2.2 Does the study the suggest anything about how/what or if consensus papers should report regarding:<br>Inclusion and exclusion criteria for the literature search?	
**Methods**<br>2.3 Does the study suggest anything of what or if consensus report should report on panel composition, n of participants, expertise, origin? Prespecified?	
**Methods**<br>2.4 Does the study suggest anything of how or if PPI (public patient involvement) activity should be reported?	
**Methods**<br>2.5 Does the study suggest anything about what or if consensus papers should report regarding panel recruitment strategies, invitations? Any level of detail specified?	
**Methods**<br>2.6 Does the study suggest how or if consensus papers should report the consensus criteria/threshold (or the level of agreement considered to reach consensus)?	
**Methods**<br>2.7 Does the study suggest how or if consensus papers should report how decision of approval of an item will be made?	
**Methods**	

2.8 Does the study suggest anything about what level of detail should be reported regarding the number of Delphi rounds or if this should be reported?	
**Methods**<br>2.9 Does the study suggest anything about what level of detail should be reported regarding the criteria used for defining the number of rounds? (why 2-3 or more e.g.) or if this should be reported?	
**Methods**<br>2.10 Does the study suggest anything about the details that should be reported regarding the time between rounds, if it should be prespecified or if this should be reported?	
**Methods**<br>2.11 Does the study suggest anything about details that should be reported of the names of the techniques of non-Delphi methods used to gather participants' inputs and reach consensus?	
**Methods**<br>2.12 Does the study suggest anything of what or in which detail should be reported regarding tool or electronic system used for Delphi? (If Delphi was used)? Or if this should be reported?	
**Methods**<br>2.13 Does the study suggest anything about how or in what level of detail the anonymity of participants (in Delphi or other methods) has to be reported? Or if this should be reported?	
**Methods**<br>2.14 Does the study suggest anything about how to report, and in what level of detail, the feedback for panellists (in Delphi rounds or other methods) process? Or if this should be reported?	
**Methods**<br>2.15 Does the study suggest anything about how or if data synthesis/analysis should be reported (from any consensus method used and how this was calculated statistically) and in what level of detail?	
**Methods**<br>2.16 Does the study suggest anything about how or if piloting should be reported and in what level of detail (e.g. understanding of consensus items, platforms used, tools used)?	
**Methods**	

For peer review only

2.17 Does the study suggest anything about how or if the role of Steering Committee members should be reported?	
**Methods**<br>2.18 Does the study suggest anything on what or if should be described regarding COI or funding?	
**Methods**<br>2.19 Does the study suggest anything on what should be described of how is dealt with COI of panellist (not allowed to vote when there is COI)? Or if this should be described	

**Results**<br>3.1 Does the study suggest anything on how to report the initial evidence search (presentation of results of the literature review)?	
**Results**<br>3.2 Does the study suggest anything on how to report n of studies found?	
**Results**<br>3.3 Does the study recommend which detail should be used when reporting panellists drop-outs (numbers and reasons)? Or if this should be reported?	
**Results**<br>3.4 Does the study suggest how or if approval rates per item shared with respondents for each round should be reported in the Results section?	
**Results**<br>3.5 Does the study suggest anything about in which detail the items that have been dropped should be reported? (reasons e.g.) Or if this should be reported?	
**Results**<br>3.6 Does the study make any recommendation on how to report the collection, synthesis and use of comments from panellists? Or if this should be reported?	
**Results**<br>3.7 Does the study suggest regarding how the final list of items (for clinical guideline or reporting guideline) should be reported? Or if this should be reported?	

**Discussion**<br>4.1 Does the paper suggest anything about reporting the limitations and strengths of the study and how? Or if this should be reported?	
**Discussion**	

For peer review only

4.2 Does the paper suggest anything about what or in which detail the applicability generalisability, and reproducibility of the study should be reported? Or if this should be reported?	

**5.1 Any other item proposed by the paper that is not captured in other columns?**	
**5.2 Any other item not proposed by the paper, but you think that could be added (not fitting the categories above)?**	

**Examples of text with well reported methods/results (for E&E document) - write NA if none was cited or found by you**	
**Additional comments from assessor**	

For peer review only

Data on reporting quality (*recommendations in italics)*

**Study**	**What is stated regarding reporting quality?**
Banno 2019[32]	• "The reporting quality of the Delphi technique in reporting guidelines is unknown even though the use of the Delphi technique was recommended in the guidance for reporting guidelines." (Note: This is a protocol for the systematic review of 2020.)<br>*4 quality score items are summarised of Delphi methods used in reporting guidelines.*
Banno 2020[16]	• "Reproducible criteria of participants, number of rounds, criteria for dropping items, and stopping criteria other than rounds were found for 87%, 97%, 69%, and 13%, respectively of reporting guidelines developed with the Delphi method. The total score of reporting quality was 2 or more in 94% of reporting guidelines using the Delphi method."<br>*4 quality score items are summarised of Delphi methods used in reporting guidelines.*
Boulkedid 2011[17]	• "Study reports did not consistently provide details that are important for interpreting the results. For example, only 39% of studies reported that individual feedback was given between rounds and the method used to define a consensus was specified in only 77% studies. Moreover, response rates for all rounds were reported in only 31% of studies. Information on both points is needed to evaluate the validity and credibility of the results. If the Delphi method is incompletely described this may affect the overall quality of the final consensus and the selected indicators are unlikely to gain the level of credibility needed for adoption I clinical practice."<br>• "The Delphi procedure is valuable for achieving a consensus about issues where none existed previously. However, our findings indicate a need for improving the use and reporting of this technique."<br>*Table 5 provides recommendations for reporting the Delphi procedure.*
Chan 2019[20]	• "This lack of clear definition has led to considerable confusion and substantial variation in the quality of reporting of Delphi studies"<br>• "One-third of medical education Delphi studies failed to report that a literature review on the topic of interest had been conducted , and over half failed to report key aspects such as what background information was provided to participants; the response rate for each round; what formal feedback of group rating was shared between rounds; a statement that anonymity was maintained; and a clear definition of consensus."<br>• "Lack of clarity in the report in the reporting of procedures and methodological choices associated with the modified Delphi studies can prevent readers from effectively appraising and interpreting findings."<br>• "Methodological rigor and transparent reporting are essential to assure readers that the consensus results are applicable to their environment, and to translate expert opinion into practice."<br>*Box 1 provides recommendations to improve reporting.*
Diamond 2014[18]	• "Definitions of consensus vary widely and are poorly reported. Improved criteria for reporting of methods of Delphi studies are required."<br>• "Methodologic criteria are proposed for the reporting of Delphi studies."<br>• "Despite the fact that the most Delphi studies in our cohort had consensus as their aim, in only a minority of the Delphi studies reviewed was consensus defined with a specific criterion. Furthermore, this criterion was the reason for termination of the Delphi process, usually on the basis of an *a priori* definition."<br>• "We believe that there is a need to improve the reporting of Delphi studies, along the lines of a CONSORT-like guideline, as is used for randomized controlled trials."<br>*Methodologic criteria are proposed for the reporting of Delphi studies.*

For peer review only

Gattrell 2019[29]	"At present there are a lack of standard, validated reporting guidelines for publications reporting the results of Delphi panel studies."<br><br>Quality assessment: Methodological quality<br>• The type of Delphi technique used, or the modifications to the method, was not outlined in all publications (included in 62/90 publications; 68.9%).<br>• Just over half of all publications stated that there was some diversity amongst participants and clearly outlined the methods for the selection of panellists.<br>• Agreement and consensus thresholds should be defined prior to study commencement, but in 40% of publications it was unclear, or not stated whether these thresholds were predefined.<br>• Anonymised responses are typically conveyed back to the group after each round, but this was clearly reported in less than half (38.9%) of publications.<br><br>Quality assessment: Reporting quality and transparency (Figure 3b).<br>• The funding source was not clearly disclosed in over a third of publications, and almost twice as many publications did not clearly disclose the funder's role.<br>• Conflicts of interest were clearly described in most publications (included in 79/90 publications; 87.8%).<br>• Clear disclosure of external support was not evident in the majority of the publications.
Grant 2018[24]	• "Specifying the analysis procedure for consensus is therefore a critical consideration when designing consensus-oriented Delphi processes in health research."<br>• "Without prespecifying their analysis procedures in a study registry, health researchers conducting consensus-oriented Delphi processes can mine for and selectively report the most desirable set of items reaching consensus and even present the reported analysis as the only one conducted. Undisclosed flexibility in data collection, analysis, and reporting is a growing concern in empirical research."<br>• "Without preregistering and reporting all of the attempted analysis procedures and when they were attempted, the extent and impact of researchers trying different analysis procedures is nearly impossible for peer reviewers, editors, and consumers of Delphi research to assess."<br>• "To be completely registered, the preanalysis plan should precisely describe the essential elements of the analysis procedure for determining consensus (see Box 2)."<br>• "Researchers should use existing guidance on reporting completed Delphi processes to provide sufficient information for comparing the final article to the registered preanalysis plan [1,12,42], with particular attention in the final article to any changes from the preanalysis plan in the items, rating criteria, analytic procedure (measure and threshold), and data and participants included in the analysis."<br>*Box 2 provides a minimum set of items to include in prospectively registered preanalysis plans for consensus-oriented Delphi processes.*
Hasson 2017[27]	• "Figure 1 Areas for reporting on the Delphi survey technique."<br>• "In Delphi surveys there exists no consistent method for reporting findings (Schmidt 1997) and a review of the literature showed that a number of approaches have been used."<br>• "The following diagram attempts to outline those sections that researchers should report upon when using the Delphi. This will help readers to judge the reliability of the method and the results obtained."<br>*Followed by a checklist of issues, which could be used by researchers.*

For peer review only - http://bmjopen.bmj.com/site/about/guidelines.xhtml

Humphrey-Murto 2017[21]	• "The authors set out to describe the use of consensus methods in medical education research and to assess the reporting quality of these methods and results."<br>• "Improved criteria for reporting are needed."<br>• "Our findings suggest that the reporting quality and standardization of consensus methods in medical education research varies greatly. The following areas appeared particularly problematic and were often left out or poorly described in the articles we reviewed: conducting a literature review to inform the consensus method; providing background information to participants; reporting the number of participants after each round; describing the level of anonymity used in the study; providing participants with feedback of group ratings; and articulating the definition of consensus used in the study."<br>*Recommendations for improvements in these areas are provided in Discussion.*
Humphrey-Murto 2017[28]	• "Consensus group methods are widely used in research to identify and measure areas where incomplete evidence exists for decision-making. Despite their widespread use, these methods are often inconsistently used and reported."<br>• "This paper and associated Guide aim to describe these methods and to highlight common weaknesses in methodology and reporting."<br>• "The AMEE Guide describes these methods to provide a "how to" approach, highlight common weaknesses in methodology and reporting, and outline recommendations for reporting future consensus based studies."<br>• "Four recent reviews using the Delphi in health care and policy-related research have systematically explored deficiencies in the use and reporting of consensus group methods. Collectively, these studies have noted deficiencies regarding: information provided to the participants at the start of Delphi, reporting response rates, feedback to participants, level of anonymity, outcomes after each round and the definition of consensus."<br>*This guide provides recommendations for improvement of reporting.*
Humphrey-Murto 2019[25]	• "Studies using the Delphi for selecting performance indicators for healthcare, for medical and nursing education, or for determining outcomes to measure in clinical trials, often fail to adequately report sufficient methodological detail. Examples include poor reporting of background information provided to participants, response rates for all rounds, level of anonymity, formal feedback between rounds, and the definition of consensus."<br>*OMERACT Delphi consensus checklist is provided in Figure 1.*
Jünger 2017[12]	• "Substantial variation was found concerning the quality of the study conduct and the transparency of reporting of Delphi studies used for the development of best practice guidance in palliative care. Since credibility of the resulting recommendations depends on the rigorous use of the Delphi technique, there is a need for consistency and quality both in the conduct and reporting of studies. To allow a critical appraisal of the methodology and the resulting guidance, a reporting standard for Conducting and Reporting of DElphi Studies (CREDES) is proposed."<br>Study adds in Box 3*"Recommendations for the Conducting and REporting of DElphi Studies (CREDES)."*
Ng 2018[30]	• "Given the variance in the use of Delphi method, reporting guidelines could help improve reporting of this research, and thereby allow readers to be aware of the accuracy of data and conclusions."<br>• "We anticipate the implementation of this will promote transparent and accurate reporting of research using Delphi method for obtaining quantitative data."<br>*A set of reporting guidelines is proposed.*
Niederberger 2020[26]	• "Significant weaknesses exist in the quality of the reporting."

For peer review only - http://bmjopen.bmj.com/site/about/guidelines.xhtml

	• "Criteria for evaluating the quality of their execution and reporting also appear to be necessary."<br>• "A specific definition of the underlying Delphi technique was found in 61% (ID11) and 88.2% (ID4) of the Delphi articles investigated."<br>• "Most of the Delphi studies analyzed in the reviews reported on the number of participating experts. The rates for the initial round were between 84% (ID6) and 100% (ID12). Four of the reviews investigated whether the number of experts was stated for each round (ID4, ID7, ID11, ID12). In one review based on 10 Delphi studies from health sciences (ID7), the authors discovered that the number of experts per round was stated in all articles. A review of 48 studies in a medical context indicated that the number of invited experts was stated less frequently with each round (ID6). Seven of the 12 reviews investigated whether the backgrounds of the experts had been reported, what kind of expertise they possessed, and the criteria according to which they were selected (ID1, ID3, ID4, ID6, ID9, ID11, ID12). One review of Delphi techniques in a health context determined that the criteria for selecting the experts was reproduced in 65 of 100 articles (65%) (ID3) included in that particular review. In other reviews with a more specific focus, such as on health care, palliative medicine, or health promotion, the rates were higher at 69% (ID11), 70% (ID9) and 79% (ID1), respectively. Based on the results of the reviews, the criteria by which the experts were selected and approached was not always clear. In one review of 100 studies from the care sector, the proportion of articles with unclear selection criteria was 11.2% (ID4), while the proportion was 93.3% in a review of 15 studies from the clinical sector (ID12)."<br>• "Seven of the 12 reviews determined whether and when consensus was defined in the Delphi studies (ID1, ID3, ID4, ID6, ID9, ID11, ID12). The number of studies in which consensus was defined in the article was between 73.5% (ID3) and 83.3% (ID9) in the reviews."<br>• "The authors of seven reviews investigated whether the number of Delphi rounds was published (ID1, ID3, ID4, ID6, ID9, ID11, ID12). The number of Delphi rounds was stated in most of the Delphi studies (e.g., ID1 82.5%, ID4 91%, ID6 100%, ID9 49.3%, ID12 93.3%). Six of the reviews included a report of the generation of the questionnaire (ID1, ID4, ID6, ID9, ID11, ID12). They demonstrated that up to 96.3% of the investigated articles reported on how the items for the questionnaire were developed (ID1). In contrast, this rate stood at 33.3% in the review of palliative care articles (ID9). The authors of two reviews investigated the question of how the items were changed during the Delphi process based on the judgments submitted by the experts (ID3, ID12). In one of the reviews, the authors indicated that 59% of the analyzed articles had defined criteria for dropping items (ID3). In another review, the authors stated that all of the investigated Delphi studies included a report of "what was asked in each round" (ID12, p. 2). The authors of the reviews reported about the feedback in most of the Delphi studies (ID11 67.9%, ID12 93.3%). The information provided about the response rate per Delphi round was less (ID1 and ID4 39%). According to the results of the reviews, around half of the studies did not provide information about the feedback design between the Delphi rounds (ID1 40%, ID4 55.1%, ID6 37.7% ID12 40%). According to the authors of the review on health promotion, the process—from formulating the issue being investigated through to the development of the questionnaire—was in general similar to a "black box," and the methodological quality of the survey instrument was almost impossible to evaluate using the published information (ID11, p. 318)."<br>• "Our results also indicate deficits both in carrying out and also reporting Delphi techniques."

For peer review only

	• "The findings in the reviews we analyzed indicated that there is no uniform process for carrying out and reporting Delphi techniques."
Paré 2013[22]	• "Thirty-one percent of the articles in our sample provided a detailed description of the expert recruitment and selection process, 43% provided only limited details, and 26% did not provide any details."<br>• "All of the articles in our database (n = 42) specified the criteria that were used to select the panel of experts. Position is by far the most used criteria (71%), followed by relevant professional experience (57%), geographic location (7%), and education level (5%)."<br>• "38% of the studies provided detailed information about the participating experts [e.g., 44], 40% provided minimal information [e.g., 2], and 22% did not provide any description".<br>• "The anonymity of the experts was reported in virtually all of the studies (95%) in our sample."<br>• "Only 29% of all of the studies reported the response rate to the initial request for participation."<br>• "35 studies (83%)reported the size of the panels. The majority of the studies (n = 21) reported a panel size between 7 and 30, only one study reported a size of 6 or less, and 13 studies reported panel sizes above 30. Nine studies (19%) examined multiple panels of experts."<br>• "Only 17% of these Delphi studies reported that a pretest of the instruments had been conducted."<br>• "24 studies out of 27 (89%) reported the brainstorming instructions that were sent to the experts."<br>• "Only 8 studies (30%) reported the use of this recommendation. (i.e. Have the experts comment and validate the consolidated list)."<br>• "The vast majority of the studies (85%) reported the final number of items at the end of phase 1."<br>• "Among the 25 studies that did not include this phase (i.e. narrowing down phase), 68% explicitly justified this choice (e.g., the number of items at the end of phase 1 are equal or less than 20 as suggested by Schmidt."<br>• "All 17 studies clearly described the narrowing down instructions that were given to the experts."<br>• "65% of the studies clearly specified their item selection rule."<br>• "Most of the studies (82%) reported the final number of items at the end of the second phase."<br>• "All 42 articles described clearly the ranking instructions that were provided to the experts."<br>• "Almost all of the studies (95%) in our sample reported the statistics that were used for data analysis."<br>• "31% of the studies in our database specified a clear stopping rule."<br>• "Only 15 studies (36%) reported the final consensus rate."<br>• "29 of the 42 studies had multiple rounds of ranking. Of these, the feedback that was provided to the experts in between the rounds included the mean ranks of items (69% of studies), an interpretation of the Kendall's W coefficient (3%), the expert's prior responses (59%), and the comments made by the other experts (38%)."<br>*Recommendations regarding what to report are provided throughout the Results section as well as in the Discussion.*
Resemann 2018[31]	• "Reporting of the Delphi method was critiqued against the AGREE Reporting Checklist."<br>• "All studies reported consensus results. The majority (8/11 [73%]) used a two-stage modified Delphi method, while the remainder used a classic three-stage process. Literature searches guided the development of statements for Delphi panel review in the majority of studies, but only 2/11 (18%) conducted

For peer review only

	systematic literature reviews and merely 6/11 (55%) of studies reported the number of statements assessed. Furthermore, 7/11 (64%) did not report collecting panellist feedback to inform subsequent Delphi stages, 5/11 (45%) of studies did not describe the rating scales used, and 2/11 (18%) omitted reporting the level of consensus reached"<br>• "There is a need for improved reporting of Delphi methods".
Waggoner 2016[23]	• "Despite the widespread utility of consensus methods and the variety of approaches available, there is a lack of guidelines for conducting such studies. This lack of stringency in guidelines for conducting consensus studies has led to variability not only in reporting results but in conducting the studies themselves."<br>• "Many studies describe their methods for collecting data and that they did have a benchmark that would point to a consensus, but a lack of a description of the analytical techniques is apparent in many studies."<br>• "In addition to the lack of descriptive techniques in these articles, there is a wide range of criteria that points to consensus. How these particular benchmarks are determined is also not a topic in many of the studies. Given the lack of current research, we believe that the methodology used I subsequent studies should be described more thoroughly in the manuscript."<br>• "We set out to determine best practices for conducting such research as well as reporting on results in the hopes that future studies are more reliable and valid."<br>*This article provides guidance for reporting of various consensus methods.*
Wang 2015[19]	• "Adoption of reporting guidelines is associated with improved reporting quality of research."<br>• "For example, 28 % of the included guidelines reported no information about consensus, and 57 % were silent about how the feedback after consensus was dealt with."<br>• "In addition to the methodology, only 31 % reported formal consensus method."<br>• "Among guidelines developed through consensus, 30 (50 %) reported group member identification and 31 (52 %) reported member recruitment. Of those who identified members, 27 (45 %) reported specialties of experts, 20 (32 %) described information of members, such as names and institutions, and four (7 %) gave the selection criteria. For those who recruited members, even (12 %) described the recruit methods, for instance, through e-mail, study co-chairs, or group decision. In guidelines developed by a working group, 22 (37 %) reported the number of experts participating in guideline development (median 32, range 3–115). Eleven (18 %) guidelines reported the endpoint of consensus process, which were all terminated after a fixed number of rounds (Table 2). In addition, the inclusion criteria of items were given in eight (13 %) guidelines. For example, items meeting the median score of eight or higher in the final round were included."<br>• "11 (18 %) described the pilot methods, seven (12 %) described the feedback information requirement and five (8 %) gave the methods for feedback collection."<br>• "More than 30 % of the reporting guidelines did not report consensus. For those who did, details of consensus methods were poorly reported."<br>• "Consensus methods should be supported by developers, and the reporting of the methods should be improved."<br>*Recommendations for Consensus methods are provided, but more about improvement of applying and reporting using all other reporting guidelines, but some items are applicable for consensus methodology as well (e.g. reporting COI and funding.*

For peer review only - http://bmjopen.bmj.com/site/about/guidelines.xhtml

**Background**<br>1.1 Does the study suggest anything about how or if consensus papers should report the context or rationale for choosing a consensus method over other methods?	1) Research problem clearly defined and topic and method justification should be reported [Hasson 2000, Figure 1 and page 1013]<br><br>2) Selection of one consensus method over another should be evident if the purpose is clearly stated. [Humphrey-Murto 2017 Med Teach page 16]<br><br>3) What is the rationale for selecting the Delphi procedure? [Humphrey-Murto 2019, Figure 1]<br><br>4) The choice of the Delphi technique as a method of systematically collating expert consultation and building consensus needs to be well justified. A rationale for the choice of the Delphi technique as the most suitable method needs to be provided [Jünger 2017, Box 3, items 1 and 8]
**Background**<br>1.2 Does the study suggest anything about how/what or if consensus papers should report the objectives of the consensus exercise?	1) Define the study objective [Boulkedid 2011, Table 5 page 7]<br><br>2) Define the purpose of the study [Chan 2019, Box 1]<br><br>3) Is the objective of the Delphi study to present results (eg, a list or statement) reflecting the consensus of the group, or does the study aim to merely quantify the level of agreement? [Diamond 2014, Table 6 and page 403] If the aim of the Delphi study is to elicit consensus, then a clear definition for what constitutes consensus should be provided a priori together with threshold values that specify when consensus is reached. If the investigators plan to only quantify the degree of consensus, but not have consensus as a criterion to stop the Delphi study, this should also be explicitly stated [Diamond 2014, page 406]<br><br>4) Research problem clearly defined and topic and method justification should be reported [Hasson 2020, Figure 1 and page 1013]<br><br>5) Authors must provide a clear purpose for their study or line of inquiry [Humphrey-Murto 2017 Med Teach, page 16]<br><br>6) The purpose of the study should be clearly defined and demonstrate the appropriateness of the use of the Delphi technique as a method to achieve the research aim. A rationale for the choice of the Delphi technique as the most suitable method needs to be provided [Jünger 2017, item 8]

For peer review only

	The Delphi technique is a flexible method and can be adjusted to the respective research aims and purposes. Any modifications should be justified by a rationale and be applied systematically and rigorously" [Jünger 2017, item 2]

**Methods**<br>2.1 Does the study the suggest anything about how/what or if consensus papers should report regarding:<br>A literature search/strategy?	1) Describe the selection and preparation of the scientific evidence for the participants [Chan 2019, Box 1]<br><br>2) A literature review should be reported [Hasson 2000, Figure 1]<br><br>3) "We suggest that this important step must be described", but they don't say how. [Humphrey-Murto 2017 AMA, page 1493 and 1496 Partially]<br><br>4) Describe the selection and preparation of the scientific evidence for the participants [Humphrey-Murto 2017 Med Teach, page 16]<br><br>5) Only implying it should happen and be reported [Resemann 2018]
**Methods**<br>2.2 Does study the suggest anything about how/what or if consensus papers should report regarding:<br>Inclusion and exclusion criteria for the literature search?	1) Clear definition of the selection criteria and/or the definition used in the Delphi questionnaire; criteria for selection should be reported [Boulkedid 2011, Table 5, Appendix S1 item 2]<br><br>2) Describe how items were selected for inclusion in questionnaire, in sufficient detail [Chan 2019, Box 1]<br><br>3) Clear selection criteria should be prespecified [Paré 2013 page 210]
**Methods**<br>2.3 Does the study suggest anything of what or if consensus report should report on panel composition, n of participants, expertise, origin? Prespecified?	1) The method used to select participants is stated. Number and type of participant subgroups (eg, patients, generalists and experts) are needed [Banno 2019, page 2 item 1]<br><br>2) The method to include and exclude participants was described. The number and type of participant subgroups (e.g., patients, generalists, and experts) were essential to record [Banno 2020, page 52 item 1]<br><br>3) How the experts were chosen (e.g., willingness to participate, expertise, or membership in an organization); Composition and characteristics of the panel, number of participants (diagram of participant flow); number invited, how they were chosen, whether they were described (age, sex, specialty), years of experience, single or from multiple

For peer review only

	specialties, inclusion of multiple stakeholders, types of stakeholders [Boulkedid 2011, page 2, Table 5, Appendix S1 item 9-15]<br><br>4) Describe how participants were selected and their qualifications. Include description of facilitator credentials [Chan 2019, Box 1]<br><br>5) Were criteria for participants reproducible? How will participants be selected or excluded? [Diamond 2014, Table 5 and 6]<br><br>6) Was there heterogeneity in panel membership and is the method for selection of experts clearly defined [Gattrell 2019, Table 1]<br><br>7) Expert selection process and characteristics should be reported in detail [Hasson 2000, page 1009, 1013]<br><br>8) How many participants were involved? We noted that the type of expertise required of participants was usually not clearly described [Humphrey-Murto 2017 AMA, page 1493 and 1494]<br><br>9) Describe how the participants were selected and their qualifications: if the NGT or RAND/UCLA is used, describe facilitator's credentials. Whatever the makeup of the expert panel, the authors must provide a rationale and justify their choices [Humphrey-Murto 2017 Med Teach]<br><br>10) How many stakeholder/participant groups will be involved in each step? Provide a rationale for inclusion or exclusion and define the stakeholder groups [Humphrey-Murto 2019, Fig 4]<br><br>11) Criteria for the selection of experts and transparent information on recruitment of the expert panel, sociodemographic details including information on expertise regarding the topic in question, (non)response and response rates over the ongoing iterations should be reported [Jünger 2017, Box 3 9]<br><br>12) Describing expert panel selection with eligibility criteria and including conflicts of interest [Ng 2018]<br><br>13) The number of experts in each round should be stated. The backgrounds of the experts should be reported, what kind of expertise they possessed, and the criteria according to which they were selected [Niederberger 2020, page 4]

For peer review only

	14) Explicit procedures for expert selection; Clear selection criteria; Clear selection criteria should be prespecified and may include the candidates' years of related experience, or tenure in a position that is relevant to the subject under study Report the response rate to the initial call for participation; provide detailed information about the participating experts (profile) to better allow judgments about their credibility [Paré 2013, page 210, Table 3]<br><br>15) Explain how groups were chosen. Consensus Development Panels: Panel composition: the panel should be made up of experts in the field; the publication should report on how they were chosen and why; [Waggoner 2016, page 665, 667]<br><br>16) Implied by mentioning that detailed information on participants was lacking in some reporting guidelines. Page 5 Report specialties of experts, names and institutions, the selection criteria [Wang 2015]
**Methods**<br>2.4 Does the study suggest anything of how or if PPI (public patient involvement) activity should be reported	No data
**Methods**<br>2.5 Does the study suggest anything about what or if consensus papers should report regarding panel recruitment strategies, invitations? Any level of detail specified?	1) The use of specific methods to encourage the experts to respond (e.g., stamped addressed envelope for returning the questionnaire and financial compensation) [page 2] and recommendation to report whether special techniques were used to invite participants [Boulkedid 2011, Appendix S1 item 21]<br><br>2) Criteria for the selection of experts and transparent information on recruitment of the expert panel, socio- demographic details including information on expertise regarding the topic in question, (non)response and response rates over the ongoing iterations should be reported" [Jünger 2017, Box 3, 9]<br><br>3) provide a detailed description of the expert recruitment and selection process [Paré 2013, page 215 first bullet on the right]<br><br>4) method of obtaining participants should be described [Waggoner 2016, page 667]
**Methods**	1) The method used to define a consensus among panel members; , whether the percentage of agreement was determined; Whether a cut-off (e.g., median value) was used to select indicators [page 2] Consensus definition at each

For peer review only

2.6 Does the study suggest how or if consensus papers should report the consensus criteria/threshold (or the level of agreement considered to reach consensus)?	round [page 7, Appendix item 28] how was consensus obtained [page 7, Appendix item 28] definition of consensus should be reported [Boulkedid 2011, table 5]<br><br>2) Clearly describe how consensus was defined [Chan 2019, Box 1]<br><br>3) Need to define criteria for consensus and to document the degree of agreement together with the results of the Delphi process. Should be defined a priori. [Diamond 2014, page 404 and table 6]<br><br>4) Was the agreement/consensus threshold predefined? [Gattrell 2019, table 1]<br><br>5) Box 2 Specific threshold for the chosen measure (e.g., median of at least 7 on a nine-point scale and an interquartile range of less than 2) [Grant 2018, p 97]<br><br>6) Determine the criteria and the meaning of `consensus' in relation to the studies [Hasson 2020, page 1013]<br><br>7) No. They do state that "articulating the definition of consensus used" was identified as "particularly problematic and were often left out or poorly described", and that "the most concerning issue we identified was that consensus was often not defined a priori. Only 43.2% of the articles we reviewed reported their definition of consensus at the start of the study." But they do not suggest how to report. [Humphrey-Murto 2017 AMA]<br><br>8) Clearly describe how consensus was defined [Humphrey-Murto 2017 Med Teach, page 18]<br><br>9) suggests definition of consensus should be reported [Humphrey-Murto 2019, table 1, also fig 1 and page 1044]<br><br>10) Definition of consensus. Unless not reasonable due to the explorative nature of the study, an a priori criterion for consensus should be defined. This includes a clear and transparent guide for action on (a) how to proceed with certain items or topics in the next survey round, (b) the required threshold to terminate the Delphi process and (c) procedures to be followed when consensus is (not) reached after one or more iterations". Definition and attainment of consensus. It needs to be comprehensible to the reader how consensus was achieved throughout the process, including strategies to deal with non-consensus". "If an a priori definition of consensus is not realistic due to the explorative nature of the study, it should be identified and established by the research team in the course of the process." [Jünger 2017, item 12]

For peer review only

	11) How was consensus defined and measured? What role did the stability of the answers play? [Niederberger 2020, Table 2] Whether and when consensus was defined in the Delphi studies. Was consensus defined a priori in advance of development of the questionnaire. [Niederberger 2020, Table 5] How was consensus measured, e.g. percentage agreement, units of central tendency (especially median) or a combination of percent agreement within a certain range and for a certain threshold. [Niederberger 2020, page 6]<br><br>12) NGT explain criteria used to determine how and when a consensus was met Consensus Development Panels: Explain what constituted consensus and how this was assessed. [Waggoner 2016, page 665] Delphi Explain what constituted consensus and how this was assessed. [Waggoner 2016, page 667]<br><br>13) The endpoint of consensus [Wang 2015, page 5]
**Methods**<br>2.7 Does the study suggest how or if consensus papers should report how decision of approval of an item will be made?	1) Whether the percentage of agreement was determined [page 2] We recorded the method used to define a consensus among panel members, whether the percentage of agreement was determined, and whether a cut-off (e.g., median value) was used to select [Boulkedid 2011, Appendix S1 item 16 (technique method)]<br><br>2) Reporting on each round separately illustrates clearly the array of themes generated in round one and gives an indication of the strength of support for each round. The presentations of findings are important and findings from subsequent rounds should be reported in a summarized format to indicate the relative standing of each of the opinions. [Hasson 2020, page 1013]<br><br>3) (Non)response and response rates over the ongoing iterations should be reported [Jünger 2017, item 9]
**Methods**<br>2.8 Does the study suggest anything about what level of detail should be reported regarding the number of Delphi rounds or if this should be reported?	1) Was the number of rounds to be performed stated (not how it should be reported, but implies it should be) [Banno 2019, page 2 under item 2]<br><br>2) Was the number of rounds to be performed stated? [Banno 2020, 3.4, table 3]<br><br>3) Describe the number of rounds planned [Chan 2019, Box 1]

	4) Specify a maximum number of rounds [page 404] what was the reason to stop the delphi [Diamond 2014, table 3] What criteria will be used to determine to stop the Delphi process or will the Delphi be run for a specific number of rounds only [Diamond 2014, table 6, table 1 item 2]<br><br>5) number and outline per round should be reported also page 1013 [Hasson 2020, fig 1]<br><br>6) Describe the number of rounds planned and/or criteria for terminating the process [Humphrey-Murto 2017 Med Teach, page 17]<br><br>7) Only implying that x number of rounds are necessary [Humphrey-Murto 2017 AMA]<br><br>8) The methods employed need to be comprehensible; information about the number and design of survey rounds, [Jünger 2017, Box 3 item 10]<br><br>9) Not specifically under item 4 in table 2 report of the specific process used? How many rounds were used in the Delphi technique [Niederberger 2020]<br><br>10) If a study goes beyond the agreed number of rounds (review suggests 2 rounds are required), this should be explained [Waggoner 2016, page 667]
**Methods**<br>2.9 Does the study suggest anything about what level of detail should be reported regarding the criteria used for defining the number of rounds? (why 2-3 or more e.g.) or if this should be reported?	1) Implied in Banno 2020 The prespecified criteria for stopping the Delphi process, other than a statement of the number of rounds, were clarified [Banno 2020]<br><br>2) Describe the number of rounds planned and criteria for terminating the process [Chan 2019, Box 1]<br><br>3) Describe the number of rounds planned and/or criteria for terminating the process [Humphrey-Murto 2017 Med Teach, page 17]<br><br>4) They, imply that the number of rounds is an important thing to report -- but they do not state this as a suggestion.[Humphrey-Murto 2017 AMA]<br><br>5) Will the number of rounds be decided a priori? If not determined a priori, what are the criteria for terminating the process? [Humphrey-Murto 2019, Fig 1]

For peer review only

	6) What was the rationale for the number of rounds; when was the number of rounds defined [Niederberger 2020, page 6]<br>7) Table 3 Report the stopping [Paré 2013]<br>8) For delphi: if a study goes beyond two rounds, explain reason for doing so; [Waggoner 2016, page 667]
**Methods**<br>2.10 Does the study suggest anything about the details that should be reported regarding the time between rounds, if this should be prespecified in advance, or if this should be reported?	1) The time taken to complete the Delphi procedure was recorded [Boulkedid 2011, page 2]
**Methods**<br>2.11 Does the study suggest anything about details that should be reported of the names of the techniques of non-Delphi methods used to gather participants' inputs and reach consensus ?	1) Whether the meeting was held before, after, or between Delphi rounds and what the participants did during the meeting [Boulkedid 2011, page 2]
**Methods**<br>2.12 Does the study suggest anything of what or in which detail	1) What software will be used to administer the Delphi? [Humphrey-Murto 2019, fig 1]

For peer review only

should be reported regarding tool or electronic system used for Delphi? (If Delphi was used)? Or if this should be reported?	
**Methods** 2.13 Does the study suggest anything about how or in what level of detail the anonymity of participants (in Delphi or other methods) has to be reported? Or if this should be reported?	1) No, only that it is a limitation of this study that the quality score did not include that. So actually they feel it should be reported how anonymity was maintained [Banno 2020]<br><br>2) Describe how anonymity was defined [Chan 2019, Box 1]<br><br>3) Were responses anonymized [Gattrell 2019, table 1]<br><br>4) It suggests that conducting anonymous iterative mail or e-mail questionnaire rounds is one of the steps [p 1491]. While the authors may have assumed that readers would understand that anonymity was part of their study design, we suggest that they state this, given the variability in approaches that have been labelled as modified consensus methods. [Humphrey-Murto 2017 AMA, page 1497]<br><br>5) Describe how anonymity was maintained. Authors must clearly state how this was accomplished. It is achieved through the use of mail outs in Delphi and RAND/UCLA and private ranking in NGT. [Humphrey-Murto 2017 Med Teach, page 18]<br><br>6) How will anonymity be maintained? [Humphrey-Murto 2019, fig 1]<br><br>7) Ensure the anonymity of the participants. The anonymity of the experts was reported in virtually all of the studies [Paré 2013]
**Methods** 2.14 Does the study suggest anything about how to report, and in what level of detail, the feedback for panellists (in	1) Whether the experts were informed of both the response of the group and their own individual response (individual feedback) to each item. The type of feedback, which was defined as qualitative when a summary of the panel's comments was sent to each participant and quantitative when simple statistical summaries illustrating the collective opinion (e.g., central tendency and variance) were sent to each participant [page 2] After each round, each participant should be given the panel results (median, lowest, and highest ratings), the participant's response, and a summary of all comments received. These data inform each participant of his or her position relative to the rest of the group, thus assisting in decisions about replies during future Delphi rounds. [Boulkedid 2011, page 8] It has been recommended that

Delphi rounds or other methods) process? Or if this should be reported?	feedback should include qualitative comments and statistical measures [citation 51, Murphy 1998]. More specifically, we determined whether the experts were informed of both the response of the group and their own individual response (individual feedback) to each item [Boulkedid 2011]<br><br>2) Describe the type of feedback provided after each round [Chan 2019, Box 1]<br><br>3) Were participants' responses in each round reported back to the group, and were responses anonymized? [Gattrell 2019, Table 1]<br><br>4) Give attention to issues which guide data collection: the discovery of opinions, the process of determining the most important issues referring to the design of the initial round, and the management of opinions [Hasson 2020, page 1013]<br><br>5) Was formal feedback provided? If so, was the feedback described? [page 1493], areas that need to be improved with reporting providing participants with feedback of group ratings [Humphrey-Murto 2017 AMA, page 1494]<br><br>6) Describe the type of feedback provided after each round [page 18]. Feedback to participants can include quantitative and/or qualitative data. It also involves two types of agreement: the extent to which individual participants agree with an issue, and the extent to which participants agree with one another. Quantitative feedback may include summary statistics such as the participants' score, participants' medians, range of scores and the proportion of participants selecting each point on a scale. Participants are provided an opportunity to change their ranking, but it should be made clear that they do not need to conform. Researchers may ask the participants who are outliers to provide written justification for their choices (qualitative data) [Humphrey-Murto 2017 Med Teach]<br><br>7) What type of feedback will participants received after each round? [2019] indicates feedback between rounds should include individuals' scores for each item and the distribution of votes by participant group. Some, however, preferred to view aggregated feedback as well as feedback to individual participants [Humphrey-Murto 2019 Yes page 1042, table 1]<br><br>8) How was the feedback designed? [Niederberger 2020, table 2]<br><br>9) Citation [Schmidt, 54] recommends three relevant pieces of feedback that can be provided to experts in phase 3 in addition to mean ranks, namely, the interpretation of Kendall's W from the previous round, the percentage of experts

For peer review only

	placing each item in the top half of their list and the relevant comments that were made by the other panellists [Paré 2013, page 213]<br><br>10) They imply that it should be reported that panellist feedback was collected to inform subsequent Delphi rounds [Resemann 2018]<br><br>11) not about reporting but they state "57 % were silent about how the feedback after consensus was dealt with." suggesting that they felt it needs to be reported. [page 2] only that some reporting guidelines described the feedback information requirement, or gave the methods for feedback collection [Wang 2015, page 6]
**Methods**<br>2.15 Does the study suggest anything about how or if data synthesis/analysis should be reported (from any consensus method used and how this was calculated statistically) and in what level of detail?	1) It is important that standards and norms for prospectively defining analysis plans are needed to improve the credibility of Delphi processes for informing health research, practice, and policy [Grant 2018, page 97]<br><br>2) The methods employed need to be comprehensible; information about methods of data analysis, processing and synthesis of experts' responses to inform the subsequent survey round [Box 3] {Jünger 2017]<br>Reporting of results for each round separately is highly advisable in order to make the evolving of consensus over the rounds transparent. This includes figures showing the average group response, changes between rounds, as well as any modifications of the survey instrument such as deletion, addition or modification of survey items based on previous rounds." [Jünger 2017, item 13]<br><br>3) Detailing statistical analyses and interpretation in arriving at final agreed values [Ng 2018, item 7]<br><br>4) The statistical analyses should be reported [Paré 2013, page 211]<br><br>5) Consensus Development Panels: Statistical analysis: must be reasonable for the research question, and should be as rigorous as possible [Waggoner 2016, page 665]
**Methods**<br>2.16 Does the study suggest anything about how or if piloting should be reported and in what	1) Pilot testing with a small group of individuals is suggested before implementation [Humphrey-Murto 2017 Med Teach, page 16]<br><br>2) All material provided to the expert panel at the outset of the project and throughout the Delphi process should be carefully reviewed and piloted in advance in order to examine the effect on experts' judgements and to prevent bias. [Box 3] The methods employed need to be comprehensible; this includes information on preparatory steps (How was

For peer review only

level of detail (e.g. understanding of consensus items, platforms used, tools used)?	available evidence on the topic in question synthesised?), piloting of material and survey instruments, design of the survey instrument(s), the number and design of survey rounds, methods of data analysis, processing and synthesis of experts' responses to inform the subsequent survey round and methodological decisions taken by the research team throughout the process [Jünger 2017]<br><br>3) Pre-test task instructions and questionnaire instruments [Paré 2013]
**Methods**<br>2.17 Does the study suggest anything about how or if the role of Steering Committee members should be reported?	No data
**Methods**<br>2.18 Does the study suggest anything on what or if should be described regarding COI or funding?	1) 'Sources of funding (industry, non-industry)'as items associated with reporting quality [Banno 2019, page 2]<br><br>2) Is the funding source clearly disclosed? [table 1] Is the role of the funder clearly disclosed? [table 1] Is the funding of any external support (e.g. with the Delphi panel meeting/questionnaires, or medical writing support for the final manuscript) clearly disclosed? [Gattrell 2019]<br><br>3) "Prevention of bias. Researchers need to take measures to avoid directly or indirectly influencing the experts' judgements. If one or more members of the research team have a conflict of interest, entrusting an independent researcher with the main coordination of the Delphi study is advisable" [Jünger 2017]<br><br>4) Describing expert panel selection with eligibility criteria and including conflicts of interest [Ng 2018]
**Methods**<br>2.19 Does the study suggest anything on what should be described of how is dealt with COI of panellist (not allowed	1) No. It only deals with COI as a planning/methodological procedure, not reporting. "5. Prevention of bias. Researchers need to take measures to avoid directly or indirectly influencing the experts' judgements. If one or more members of the research team have a conflict of interest, entrusting an independent researcher with the main coordination of the Delphi study is advisable"[Jünger 2017]

For peer review only

to vote when there is COI)? Or if this should be described	

**Results**<br>3.1 Does the study suggest anything on how to report the initial evidence search (presentation of results of the literature review)?	1) No, but they suggest it should be reported [Jünger 2017]
**Results**<br>3.2 Does the study suggest anything on how to report n of studies found?	No data
**Results**<br>3.3 Does the study recommend which detail should be used when reporting panellists drop-outs (numbers and reasons)? Or if this should be reported?	1) No but it states that number the response rate for the first round dropped to 170 (66.1%). [page 1494]; areas that need improvement in reporting the number of participants after each round [page 1496] Other analyses of consensus methods research found similar poor reporting of this feature, with 7% to 39% of studies reporting response rates for all rounds of data collection [Humphrey-Murto 2017 AMA]<br><br>2) Fig 1 step 7 How will non-responders be managed, i.e. will they be excluded in subsequent rounds What response rate will be acceptable for each stakeholder group in each round? [Humphrey-Murto 2019]<br><br>3) Reporting of results for each round separately is highly advisable in order to make the evolving of consensus over the rounds transparent. This includes figures showing the average group response, changes between rounds, as well as any modifications of the survey instrument such as deletion, addition or modification of survey items based on previous rounds [Jünger 2017, Box 3]<br><br>4) Outlining participation and attrition rates for each round [Ng 2018]

For peer review only

	5) report the response rate to the initial request for participation, the size of the panel and the retention rate; [Paré 2013, page 215 3rd bullet]
**Results**<br>3.4 Does the study suggest how or if approval rates per item shared with respondents for each round should be reported in the Results section?	1) Response rate for each round [Boulkedid 2011, Table 5 on page 7]<br><br>2) Yes Box 1 report response rates and results after each round [Chan 2019]<br><br>3) Response rates for each round should be reported, presentation of total of issues generated in round 1, and presentation of results in round 2 indicating strength of support [Hasson 2000, figure 1 and page 1013]<br><br>4) Report response rates and results after each round [Humphrey-Murto 2017 Med Teach, page 18]<br><br>5) it should report response rates for all rounds [Humphrey-Murto 2019, page 1042]<br><br>6) Reporting of results for each round separately is highly advisable in order to make the evolving of consensus over the rounds transparent. This includes figures showing the average group response, changes between rounds, as well as any modifications of the survey instrument such as deletion, addition or modification of survey items based on previous rounds." [Jünger 2017, item 13]Criteria for the selection of experts and transparent information on recruitment of the expert panel, socio- demographic details including information on expertise regarding the topic in question, (non)response and response rates over the ongoing iterations should be reported". [Jünger 2017]<br><br>7) Reporting both quantitative results and textual comments for each round of analysis [Ng 2018]<br><br>8) How high was the response rate from the experts both when initially approached and also for the individual rounds [Niederberger 2020, Table 2]<br><br>9) Level of consensus should be reported [Resemann 2018]
**Results**<br>3.5 Does the study suggest anything about in which detail the items that have been dropped should	1) Were the criteria for dropping clear; are stopping criteria, other than rounds, reported [Banno 2019, item 3 and 4]<br><br>2) Were the criteria for dropping items clear? (yes, no, or not applicable) [Banno 2020, 2.6 item 3]<br><br>3) Clear criteria for dropping or combining items should also be specified based on the level of agreement or disagreement with individual items. One of the limitations of a priori specification is that certain items may fall just below the

For peer review only

be reported? (reasons e.g.) Or if this should be reported?	threshold for what is fundamentally an arbitrary cut off. In the event that items, believed to be important fell just below the threshold for inclusion in the study, the authors could consider including these items as posteriori considerations provided that sufficient justification was provided. [page 405] Suggested quality criteria: Were criteria for dropping items clear; Stopping criteria other than rounds specified? [Table 5] Were items dropped? What criteria will be used to determine which items to drop? [Diamond 2014, Table 6]<br><br>4) No, but they state Interpretation and processing of results. Consensus does not necessarily imply the correct answer or judgement; (non)consensus and stable disagreement provide informative insights and highlight differences in perspectives concerning the topic in question and Definition and attainment of consensus. It needs to be comprehensible to the reader how consensus was achieved throughout the process, including strategies to deal with non-consensus [Jünger 2017 in Box 3]<br><br>5) Were criteria defined for dropping items [Niederberger 2020, page 6]
**Results**<br>3.6 Does the study make any recommendation on how to report the collection, synthesis and use of comments from panellists? Or if this should be reported?	1) It has been recommended that feedback should include qualitative comments and statistical measures [Murphy 1998, 51]. After each round, each participant should be given the panel results (median, lowest, and highest ratings), the participant's response, and a summary of all comments received [Boulkedid 2011]<br><br>2) Describe the type of feedback provided after each round. Quantitative feedback may include summary statistics such as the participants' score, participants' medians, range of scores and the proportion of participants selecting each point on a scale. Participants are provided an opportunity to change their ranking, but it should be made clear that they do not need to conform [Humphrey-Murto 2017 Med Teach]<br><br>3) Reporting of results for each round separately is highly advisable in order to make the evolving of consensus over the rounds transparent. This includes figures showing the average group response, changes between rounds, as well as any modifications of the survey instrument such as deletion, addition or modification of survey items based on previous rounds [Jünger 2017, item 13]<br><br>4) Ask experts to justify their rankings. Have experts comment and validate consolidated list [page 210 Table 3]. Did experts consolidate the list of items; Did experts comment on and validate the list of items; Was the final number of items reported.<br>Report whether panel members had the opportunity to justify or clarify their own reasoning and to comment on the responses of the other experts as well as on the progress of the panel as a whole. [Paré 2013, page 213].

For peer review only

	Were panellists able to revise previous statements [Paré 2013]<br><br>5) No, but implied that it should be: did not report collecting panellist feedback to inform subsequent Delphi stages [Resemann 2018]
**Results**<br>3.7 Does the study suggest regarding how the final list of items (for clinical guideline or reporting guideline) should be reported? Or if this should be reported?	1) Partially. It says it should be detailed and disseminated, but it does not suggest how (in what format) it should be reported [Jünger 2017]<br><br>2) Suggests "detailing statistical analyses and interpretation in arriving at final agreed values" [Ng 2018]<br><br>3) Report final number of items [Paré 2013, page 210 Table 3]<br><br>4) No but again imply "reported the number of statements assessed." [Resemann 2018]

**Discussion**<br>4.1 Does the paper suggest anything about reporting the limitations and strengths of the study and how? Or if this should be reported?	1) Address potential methodological issues (e.g lack of consensus) or limitations in the discussion (e.g. low response rate) [Chan 2019, Box 1]<br><br>2) Interpretation of consensus gained/not gained [Hasson 2020, page 1009]<br><br>3) In the discussion the authors should address issues that may have impacted the results such as poor response rates between rounds, lack of participation from a select group or geographic region, or lack of consensus. [Humphrey-Murto 2017 Med Teach, page 18]<br><br>4) Methodological issues should be reported [Humphrey-Murto 2019, figure 1]<br><br>5) Reporting should include a critical reflection of potential limitations and their impact of the resulting guidance". [Jünger 2017]
**Discussion**<br>4.2 Does the paper suggest anything about what or in	1) Page 5: is considered a good measure if it meets criteria including reliability, sensitivity, specificity, and feasibility (or applicability) [20,31]. The common use of these characteristics can facilitate acceptance and implementation of indicators developed [Boulkedid 2011]

For peer review only

which detail the applicability generalisability, and reproducibility of the study should be reported? Or if this should be reported?	2) The conclusions should adequately reflect the outcomes of the Delphi study with a view to the scope and applicability of the resulting practice guidance. [Jünger 2017, item 15]<br><br>3) It is also necessary to discuss the critical and rationalistic criteria for the validity and reliability of the studies and the more constructivist characteristics of credibility, transparency, and transferability. [Niederberger 2020, page 8]

**5.1 Any other item proposed by the paper that is not captured in other columns?**	1) Were criteria for dropping items clear? Are stopping criteria, other than rounds, specified [Banno 2019]<br><br>2) Differences between the protocol and the article [Banno 2020, 2.9]<br><br>3) Geographic scope of the survey [page 2]. Main methods used to send the questionnaires (e.g., mail, E-mail, or fax). [Boulkedid 2011, page 7]<br>The formulation of the questionnaire items (e.g., open questions, rating of quality indicators, or both). [Boulkedid 2011]<br>Whether the quality indicators were rated (in which case, we recorded the minimum and maximum values on the rating scale). [Boulkedid 2011]<br>A flow chart of quality indicators (figure showing the output and input indicators at each round) and/or for a written description of indicator flow. [Boulkedid 2011, page 3]<br>Quality indicators used in the first round versus the end of the last round. [Boulkedid 2011, page 3]<br>Availability of the questionnaires in the article itself or in an appendix [Boulkedid 2011, page 3]<br>Whether selection criteria changed between rounds [Boulkedid 2011, page 5]<br>Whether panelists were able to make comments. [Boulkedid 2011, page 6]<br>Whether there was a meeting; at what stage it took place and how people participated [Boulkedid 2011]<br>Response rate for each round [Boulkedid 2011, page 7]<br>preparation in advance of starting Delphi (outcome indicators, structure indicators, process indicators) [Boulkedid 2011, In appendix S1, item 1]<br>METHODS<br>We evaluated the relationship between the response rate and the use of specific methods to encourage the experts to respond (e.g., stamped addressed envelope for returning the questionnaire and financial compensation). Also on maybe we should add item regarding encouragement of participants [Boulkedid 2011, page 2, page 5 right column]<br>Geographic scope of Delphi consensus procedure [Boulkedid 2011,item 20 of appendix and table 5]<br>Question format (open questions, rating scale?) Also in table 5 how were questions formulated? [Boulkedid 2011, item 24

For peer review only

	appendix]<br>Rating scale [Boulkedid 2011, item 25]<br>Methods used to send questionnaire (email fax, mail) [Boulkedid 2011, table 5]<br>Time to complete questionnaire reporting of differences in response rate in rounds [Boulkedid 2011]<br>Number of rounds necessary to reach consensus [Boulkedid 2011]<br>Duration of the procedure [Boulkedid 2011]<br>Is questionnaire added as appendix? [Boulkedid 2011]<br>For Discussion: Validity [Boulkedid 2011]<br><br>4) Outline each step of the process. If modifications were made, provide a rationale for your choices. [Chan 2019]<br>Describe the selection and preparation of the scientific evidence for the participants. [Chan 2019]<br>Include a description of the facilitator's credentials. [Chan 2019]<br>What background material was provided to participants. [Chan 2019]<br>What formal feedback of group rating was shared between rounds [Chan 2019]<br><br>5) Specify stopping criteria in the absence of consensus [Diamond 2014]<br><br>6) Were the questions formulated or validated by an expert panellist [Gattrell 2019]<br><br>7) Researchers conducting consensus-oriented Delphi processes should prospectively and completely register the intended procedure for identifying which items reach consensus. [Grant 2018]<br>The analysis procedure for determining consensus for Delphi processes should be chosen a priori ideally before starting the first round but at the very latest before completing data collection to improve the validity of findings. [Grant 2018]<br>Health researchers conducting consensus-oriented Delphi processes should commit themselves in advance to an analytic procedure for determining which items reach consensus before they see the actual data (or, ideally, before they even collect the data). [Grant 2018]<br>Registrations should be in a publicly available and independently controlled platform that time-stamps entries [Grant 2018]<br><br>8) "Copy of each round questionnaire illustrated" [Hasson 2020]<br>statistical interpretation for the reader [Hasson 2020]<br>appendices to include the questionnaires [Hasson 2020]<br>For Discussion interpretations of consensus gained/not gained reliability and validity [Hasson 2020]

For peer review only

	9) *Page 1493(2) Was background information provided to the participants? pg 1496 areas appeared particularly problematic and were often left out or poorly described: providing background information to participants<br>AND so a clear description of what information was provided and in what format is important<br>* (3) Was the consensus method used for item generation, ranking, or both?<br>* (11) Was consensus forced?<br>Was mail/e-mail polling or face-to-face questioning used? [Humphrey-Murto 2017 AMA]<br><br>10) Outline each step of the process: if modifications were made, provide a rationale for the choices made. Providing justification for the choices made will also add credibility. [Humphrey-Murto 2017 Med Teach]<br><br>11) Background provided to participants, what is level of detail provided [Humphrey-Murto 2019]<br>Figure 1 clear outline of the overall process involved and where Delphi fits [Humphrey-Murto 2019, figure 1]<br>How sample size is determined of participants [Humphrey-Murto 2019, figure 1]<br><br>12) Any modifications should be justified by a rationale and be applied systematically and rigorously [Jünger 2017, Box 3]<br>All material provided to the expert panel at the outset of the project and throughout the Delphi process should be carefully reviewed and piloted in advance in order to examine the effect on experts' judgements and to prevent bias [Jünger 2017]<br>It is recommended to have the final draft of the resulting guidance on best practice in palliative care reviewed and approved by an external board or authority before publication and dissemination [Jünger 2017, Box 3]<br>information about methodological decisions taken by the research team throughout the process Jünger 2017, Box 3]<br>Flow chart to illustrate the stages of the Delphi process, including a preparatory phase, the actual Delphi rounds, interim steps of data processing and analysis, and concluding steps [Jünger 2017, Box 3]<br>Publication and dissemination [Jünger 2017, Box 3]<br><br>13) Item 2-4 and 9 appending revised questionnaires [Ng 2018]<br><br>14) Specific definition of underlying Delphi technique (or as I thought it is important to define exactly what method is used, especially if a modified method is used this needs to be very clear [Niederberger 2020]<br>What role did the stability of the answers play? [Niederberger 2020, table 2]<br>Questionnaire and scale development How were the questionnaires and the specific items for a Delphi technique

For peer review only

	developed? [Niederberger 2020]<br>Nevertheless, it is important to precisely describe, justify, and methodologically reflect on any modifications [Niederberger 2020]<br>How were the questionnaires and the specific items for a Delphi technique developed? [Niederberger 2020, Table 2]<br>Were items identified from empirical analyses such as qualitative interviews or focus groups that were completed in advance or were taken from existing guidelines. [Niederberger 2020, Complementary AND page 6<br>Was the first (qualitative) round of questions in the Delphi process used to generate the items for a standardized questionnaire. [Niederberger 2020, Complementary AND page 6]<br><br>15) Was the final number of items reported [Paré 2013, Table 3] Were items randomly ordered [Paré 2013, Table 3]<br><br>16) Describe the rating scales used [Resemann 2018] the number of statements assessed should be reported [Resemann 2018]<br><br>17) For nominal group process, the research question used to prompt the panel must be clear and concise to obtain valid suggestions from panel members. [Waggoner 2016, page 665] The heterogeneity should be reported [Waggoner 2016, page 665] Evaluation of reliability [Waggoner 2016, page 665]<br><br>18) Meeting attendance; format (e.g. face-to-face); agenda preparation; materials sent to participants prior to meeting; duration of meeting [Wang 2015, page 5] Flow diagram [Wang 2015, page 3] Should we add something regarding other consensus methods including an item regarding face to face meetings? [Wang 2015, page 5]
**5.2 Any other item not proposed by the paper, but you think that could be added (not fitting the categories above)?**	1) Are stopping criteria, other than rounds, specified? [Banno 2019, page 2]<br><br>2) Information letter explaining the method and the reasons their participation to the whole process would be necessary, as well as a form for collecting their consent to complete the entire Delphi process. [Boulkedid 2011]<br><br>3) "Round 1: presentation of total number of issues generated" [Hasson 2020]<br><br>4) This paper was "pointing fingers", showing what was wrong, without suggesting solutions. However, we can be inspired by the critics to build the following list of items: 1) Purpose of the consensus study<br>Whether a literature review was done to support the selection of items [Humphrey-Murto 2017 AMA]<br><br>5) Length of the background provided [Humphrey-Murto 2019]

For peer review only

	Purpose of study: outcome/diagnosis/intervention? [Humphrey-Murto 2019]

**Examples of text with well reported methods/results (for E&E document) - write NA if none was cited or found by you**	1) Page 7 Table 5 [Boulkedid 2011]<br>2) Box 1 [Chan 2019]<br>3) Might have a look at table 6 [Diamond 2014]<br>4) Table 1 [Gattrell 2019]<br>5) Parts of Fig 1 and checklist page 1013 [Hasson 2020]<br>6) Table 1 lists "exemplary publications" for nominal group process, consensus development panel and Delphi technique Page 667 references studies that were "Very descriptive" of the statistical techniques used. [Waggoner 2016]
**Additional comments from assessor**	1) Limited value; protocol for Banno 2020 [Banno 2019]<br>2) Of limited use. The authors developed a 4-point quality score that they applied to Delphi publications [Banno 2020]<br>3) Excellent resource [Boulkedid 2011]<br>4) Focusses on defining consensus [Diamond 2014]<br>5) Congress poster only [Gattrell 2019]<br>6) Study used RAND's ExpertLens as the Delphi platform [Grant 2018]<br>7) 1497: The lack of consensus on consensus methods makes it imperative that researchers provide clear and detailed reporting of the methods they used and that they justify these choices. [Humphrey-Murto 2017]

For peer review only

	8) Page 1044 A suggestion to improv uniformity is to use a software program that provides structure and help with reporting all relevant outcomes (e.g. DelphiManager, http://comet-initiative.org/delphimanager/) [Humphrey-Murto 2019]<br><br>9) Very informative [Jünger 2017]<br><br>10) The study focusses on information systems. Arguably, this is not within the inclusion criteria for the search [Paré 2013]<br><br>11) Review covers nominal group process, consensus development panel and Delphi technique [Waggoner 2016]<br><br>12) Study looked at the reporting quality of reporting guidelines [Wang 2015]

For peer review only

ACcurate COnsensus Reporting Document (ACCORD): Summary of extracted data from literature search

Table of Contents

1. Background 2
2. Methods 3
3. Results 9
4. Discussion 11
5. Additional topics 12
References 14

For peer review only

Section: Background

1. Background

Data extraction question	Articles	Checklist item(s) with brief explanation
1.1. Does the study suggest anything about how or if consensus papers should report the context or rationale for choosing a consensus method over other methods?	Hasson F, *et al. J Adv Nurs* 2000[1]<br>Humphrey-Murto S, *et al. Med Teach* 2017[2]<br>Humphrey-Murto S, *et al. J Rheumatol* 2019[3]<br>Jünger S, *et al. Palliat Med* 2017[4]	State the rationale for use of consensus method over other options.<br>*Should consider other consensus methods as well as other methodology types.*
1.2. Does the study suggest anything about how/what or if consensus papers should report the objectives of the consensus exercise?	Hasson F, *et al. J Adv Nurs* 2000[1]<br>Humphrey-Murto S, *et al. Med Teach* 2017[2]<br>Jünger S, *et al. Palliat Med* 2017[4]<br>Boulkedid R, *et al. PLoS One* 2011[5]<br>Chan TM, *et al. CJEM* 2019[6]<br>Diamond IR, *et al. J Clin Epidemiol* 2014[7]	Clearly define study objectives.<br>*Could include presentation of group consensus, or just to quantify the level of agreement.*

For peer review only

Section: Methods

2. Methods

Data extraction question	Articles	Checklist item(s) with brief explanation
2.1. Does the study suggest anything about how/what or if consensus papers should report regarding:<br><br>A literature search/strategy?	Hasson F, *et al. J Adv Nurs* 2000[1]<br>Humphrey-Murto S, *et al. Med Teach* 2017[2]<br>Chan TM, *et al. CJEM* 2019[6]<br>Humphrey-Murto S, *et al. Acad Med* 2017[8]<br>Resemann HK, *et al. Curr Med Res Opin* 2018[9]	A) Describe the strategy for reviewing the existing scientific evidence that informed the study.<br>*If no existing literature is available, the extent of the search should be described.*<br>B) Describe how existing scientific evidence will be provided to the participants.<br>*If different participant groups are involved, it should be stated which information will be provided to which group.*
2.2. Does the study suggest anything about how/what or if consensus papers should report regarding:<br><br>Inclusion and exclusion criteria for the literature search?	Boulkedid R, *et al. PLoS One* 2011[5]<br>Chan TM, *et al. CJEM* 2019[6]<br>Paré G, *et al. Inf Manag* 2013[10]	Describe the process of the literature search.<br>*Should include inclusion and exclusion criteria, and state whether these were prespecified.*
2.3. Does the study suggest anything of what or if consensus report should report on panel composition, n of participants, expertise, origin? Prespecified?	Hasson F, *et al. J Adv Nurs* 2000[1]<br>Humphrey-Murto S, *et al. Med Teach* 2017[2]<br>Humphrey-Murto S, *et al. J Rheumatol* 2019[3]<br>Jünger S, *et al. Palliat Med* 2017[4]<br>Boulkedid R, *et al. PLoS One* 2011[5]<br>Chan TM, *et al. CJEM* 2019[6]<br>Diamond IR, *et al. J Clin Epidemiol* 2014[7]<br>Humphrey-Murto S, *et al. Acad Med* 2017[8]<br>Paré G, *et al. Inf Manag* 2013[10]<br>Banno M, *et al. J Clin Epidemiol* 2019[11]<br>Banno M, *et al. J Clin Epidemiol* 2020[12]<br>Gattrell WT, *et al. Curr Med Res Opin* 2019[13]	A) Describe the structure of the study's participants.<br>*Should describe inclusion of a Chair/Co-chairs, steering committee, and subgroups, if applicable.*<br>B) Explain how panel participants were selected.<br>*Should state who was responsible for panellist selection, the selection criteria applied, the justification for choosing panellist numbers and selection criteria, and whether criteria were prespecified.*

For peer review only

Section: Methods

Data extraction question	Articles	Checklist item(s) with brief explanation
	Ng J. *Value Health* 2018[14]<br>Niederberger M, *et al. Front Public Health* 2020[15]<br>Waggoner J, *et al. Acad Med* 2016[16]<br>Wang X, *et al. BMC Med Res Methodol* 2015[17]	*C)* Describe the composition of the panel. *Should include number of participants at all stages of the process, sociodemographics (e.g. age, sex, specialty, type and duration of relevant experience). Should also describe panel subgroups, if relevant.*<br>D) Describe the expertise of the panel. *Should include the definition of "expert" and description of any public or patients involved.*<br>E) Describe the facilitator(s), if used. *Should include type and duration of relevant experience, and the role played in the process.*
2.4. Does the study suggest anything of how or if PPI (public patient involvement) activity should be reported	No data	Describe the role and involvement of any public or patients.<br>*Should detail the stage(s) at which they were involved, and their roles and contributions.*
2.5. Does the study suggest anything about what or if consensus papers should report regarding panel recruitment strategies, invitations? Any level of detail specified?	Jünger S, *et al. Palliat Med* 2017[4]<br>Boulkedid R, *et al. PLoS One* 2011[5]<br>Paré G, *et al. Inf Manag* 2013[10]<br>Waggoner J, *et al. Acad Med* 2016[16]	Describe how the panel members were recruited.<br>*Could include communication/advertisement method(s) and locations.*
2.6. Does the study suggest how or if consensus papers should report the consensus criteria/threshold (or the level of agreement considered to reach consensus)?	Hasson F, *et al. J Adv Nurs* 2000[1]<br>Humphrey-Murto S, *et al. Med Teach* 2017[2]<br>Humphrey-Murto S, *et al. J Rheumatol* 2019[3]<br>Jünger S, *et al. Palliat Med* 2017[4]<br>Boulkedid R, *et al. PLoS One* 2011[5]<br>Chan TM, *et al. CJEM* 2019[6]<br>Diamond IR, *et al. J Clin Epidemiol* 2014[7]<br>Humphrey-Murto S, *et al. Acad Med* 2017[8]<br>Gattrell WT, *et al. Curr Med Res Opin* 2019[13]	A) Define the consensus measure to be used. *Could include percentage agreement, units of central tendency (e.g. median), a categorical rating (e.g. Agree/Strongly agree) or a combination of percent agreement within a certain range.*<br>B) State the threshold for the group achieving consensus. *Should include whether the threshold was*

For peer review only - http://bmjopen.bmj.com/site/about/guidelines.xhtml

Section: Methods

Data extraction question	Articles	Checklist item(s) with brief explanation
	Niederberger M, *et al. Front Public Health* 2020[15]<br>Waggoner J, *et al. Acad Med* 2016[16]<br>Wang X, *et al. BMC Med Res Methodol* 2015[17]<br>Grant S, *et al. J Clin Epidemiol* 2018[18]	*pre-defined and highlight any threshold variation between rounds, with explanation for the change. If the intention is to quantify the degree of consensus but not to use consensus as a stop criterion for the study, this should be stated.*
2.7. Does the study suggest how or if consensus papers should report how decision of approval of an item will be made?	Hasson F, *et al. J Adv Nurs* 2000[1]<br>Jünger S, *et al. Palliat Med* 2017[4]<br>Boulkedid R, *et al. PLoS One* 2011[5]	Explain how final consensus was reached.<br>*Should describe the evolution of themes between voting rounds, if applicable.*
2.8. Does the study suggest anything about what level of detail should be reported regarding the number of Delphi rounds or if this should be reported?	Hasson F, *et al. J Adv Nurs* 2000[1]<br>Humphrey-Murto S, *et al. Med Teach* 2017[2]<br>Jünger S, *et al. Palliat Med* 2017[4]<br>Chan TM, *et al. CJEM* 2019[6]<br>Diamond IR, *et al. J Clin Epidemiol* 2014[7]<br>Humphrey-Murto S, *et al. Acad Med* 2017[8]<br>Banno M, *et al. J Clin Epidemiol* 2019[11]<br>Banno M, *et al. J Clin Epidemiol* 2020[12]<br>Niederberger M, *et al. Front Public Health* 2020[15]<br>Waggoner J, *et al. Acad Med* 2016[16]	State how many voting rounds were conducted.<br>*Should include whether the number of rounds was prespecified, and whether this was an absolute or a maximum. If the maximum was exceeded, should explain the reasoning for doing so.*
2.9. Does the study suggest anything about what level of detail should be reported regarding the criteria used for defining the number of rounds? (why 2-3 or more e.g.) or if this should be reported?	Humphrey-Murto S, *et al. Med Teach* 2017[2]<br>Humphrey-Murto S, *et al. J Rheumatol* 2019[3]<br>Chan TM, *et al. CJEM* 2019[6]<br>Humphrey-Murto S, *et al. Acad Med* 2017[8]<br>Paré G, *et al. Inf Manag* 2013[10]<br>Banno M, *et al. J Clin Epidemiol* 2020[12]<br>Niederberger M, *et al. Front Public Health* 2020[15]<br>Waggoner J, *et al. Acad Med* 2016[16]	Explain the rationale for choosing the number of voting rounds.<br>*Should also describe the stop criteria, if used, and whether these were prespecified.*
2.10. Does the study suggest anything about the details that should be reported regarding the time between rounds, if this should be	Boulkedid R, *et al. PLoS One* 2011[5]	Describe the time period between voting rounds.<br>*Should include whether the period was*

For peer review only - http://bmjopen.bmj.com/site/about/guidelines.xhtml

Section: Methods

Data extraction question	Articles	Checklist item(s) with brief explanation
prespecified in advance, or if this should be reported?		*prespecified and highlight differences between inter-round periods, if applicable.*
2.11. Does the study suggest anything about details that should be reported of the names of the techniques of non-Delphi methods used to gather participants' inputs and reach consensus?	Boulkedid R, *et al. PLoS One* 2011[5]<br>Waggoner J, *et al. Acad Med* 2016[16]	Describe any additional methods used alongside the consensus process.<br>*Should include all that were used, e.g. a self-administered questionnaire combined with a group meeting. Should also explain how the consensus process fitted into the overall study methodology.*
2.12. Does the study suggest anything of what or in which detail should be reported regarding tool or electronic system used for Delphi? (If Delphi was used)? Or if this should be reported?	Humphrey-Murto S, *et al. J Rheumatol* 2019[3]	Describe any tools used to administer the voting.<br>*Could detail electronic platforms, if used.*
2.13. Does the study suggest anything about how or in what level of detail the anonymity of participants (in Delphi or other methods) has to be reported? Or if this should be reported?	Humphrey-Murto S, *et al. Med Teach* 2017[2]<br>Humphrey-Murto S, *et al. J Rheumatol* 2019[3]<br>Chan TM, *et al. CJEM* 2019[6]<br>Humphrey-Murto S, *et al. Acad Med* 2017[8]<br>Paré G, *et al. Inf Manag* 2013[10]<br>Banno M, *et al. J Clin Epidemiol* 2020[12]<br>Gattrell WT, *et al. Curr Med Res Opin* 2019[13]	Detail how anonymity of voters was maintained.<br>*Could involve use of mail-outs in a standard Delphi procedure, blinding on an electronic platform, or private ranking in the NGT.*
2.14. Does the study suggest anything about how to report, and in what level of detail, the feedback for panellists (in Delphi rounds or other methods) process? Or if this should be reported?	Hasson F, *et al. J Adv Nurs* 2000[1]<br>Humphrey-Murto S, *et al. Med Teach* 2017[2]<br>Humphrey-Murto S, *et al. J Rheumatol* 2019[3]<br>Boulkedid R, *et al. PLoS One* 2011[5]<br>Chan TM, *et al. CJEM* 2019[6]<br>Humphrey-Murto S, *et al. Acad Med* 2017[8]<br>Resemann HK, *et al. Curr Med Res Opin* 2018[9]<br>Paré G, *et al. Inf Manag* 2013[10]<br>Gattrell WT, *et al. Curr Med Res Opin* 2019[13]<br>Niederberger M, *et al. Front Public Health* 2020[15]	Explain how voting feedback was provided to panellists at the end of each round.<br>*Could include summaries of group voting and/or their own individual responses. Should state whether feedback will be quantitative and/or qualitative, and whether it will be anonymised. If no feedback was provided, this should be stated.*

For peer review only - http://bmjopen.bmj.com/site/about/guidelines.xhtml

Section: Methods

Data extraction question	Articles	Checklist item(s) with brief explanation
	Wang X, *et al. BMC Med Res Methodol* 2015[17]	
2.15. Does the study suggest anything about how or if data synthesis/analysis should be reported (from any consensus method used and how this was calculated statistically) and in what level of detail?	Jünger S, *et al. Palliat Med* 2017[4]<br>Paré G, *et al. Inf Manag* 2013[10]<br>Ng J. *Value Health* 2018[14]<br>Waggoner J, *et al. Acad Med* 2016[16]<br>Grant S, *et al. J Clin Epidemiol* 2018[18]	Detail methods used to process responses after each voting round.<br>*Could include statistical analysis methods, if used.*
2.16. Does the study suggest anything about how or if piloting should be reported and in what level of detail (e.g. understanding of consensus items, platforms used, tools used)?	Humphrey-Murto S, *et al. Med Teach* 2017[2]<br>Jünger S, *et al. Palliat Med* 2017[4]<br>Paré G, *et al. Inf Manag* 2013[10]	Describe any piloting of the study materials and/or survey instruments.<br>*Should include the number of individuals in the pilot group and the rationale for their selection. Should also explain any changes made as a result of the pilot. If no pilot was conducted, this should be stated.*
2.17. Does the study suggest anything about how or if the role of Steering Committee members should be reported?	No data	Describe the role(s) of the Steering Committee in the process.<br>*Should also detail the involvement of the Chair/Co-chairs, subgroups, or individual members at relevant stages of the process, if different from the group as a whole.*
2.18. Does the study suggest anything on what or if should be described regarding COI or funding?	Jünger S, *et al. Palliat Med* 2017[4]<br>Banno M, *et al. J Clin Epidemiol* 2019[11]<br>Gattrell WT, *et al. Curr Med Res Opin* 2019[13]<br>Ng J. *Value Health* 2018[14]	A) Disclose any COI of the panellists<br>*Should specify COI of each participant in the panel.*<br>B) Disclose any funding received and the role of the funder.<br>*Should specify the role of the funding source(s), e.g. involvement in the study concept/design, participation of the Steering Committee, for conducting the consensus process, medical writing support for its reporting.*

For peer review only

For peer review only - http://bmjopen.bmj.com/site/about/guidelines.xhtml

Section: Methods

Data extraction question	Articles	Checklist item(s) with brief explanation
2.19. Does the study suggest anything on what should be described of how is dealt with COI of panellist (not allowed to vote when there is COI)? Or if this should be described	Jünger S, *et al*. *Palliat Med* 2017[4]	Describe measures taken to avoid influence by any conflicts of interest (COI). *Should include disclosure of COI and how this was accounted for in the methodology, e.g. by limiting voting in case of a specific COI, adjudication by an independent researcher.*

For peer review only

Section: Results

3. Results

Data extraction question	Articles	Checklist item(s) with brief explanation
3.1. Does the study suggest anything on how to report the initial evidence search (presentation of results of the literature review)?	Jünger S, *et al*. *Palliat Med* 2017[4]	Describe how existing scientific evidence was provided to the participants.<br>*Should include relevant specifics of the literature search, e.g. n of studies reported, to provide relevant context for the results. If different participant groups were involved, it should be stated which information was provided to which group.*
3.2. Does the study suggest anything on how to report n of studies found?	No data	Describe the results of the search and number of included studies.
3.3. Does the study recommend which detail should be used when reporting panellists drop-outs (numbers and reasons)? Or if this should be reported?	Humphrey-Murto S, *et al. J Rheumatol* 2019[3]<br>Jünger S, *et al*. *Palliat Med* 2017[4]<br>Humphrey-Murto S, *et al. Acad Med* 2017[8]<br>Paré G, *et al. Inf Manag* 2013[10]<br>Ng J. *Value Health* 2018[14]	A) State the response rates for each voting round.<br>*Should specify n as well as percent, or otherwise indicate attrition/retention rates.*<br>B) State the reasons cited for voter drop-outs at each stage of the process.<br>*Could be provided as an aggregated summary or as individual responses. If this information was not collected, this should be stated.*<br>C) Describe measures undertaken to maintain acceptable response rates.<br>*If threshold rates differ between stakeholder groups, these should be described with explanation.*

For peer review only - http://bmjopen.bmj.com/site/about/guidelines.xhtml

Section: Results

Data extraction question	Articles	Checklist item(s) with brief explanation
3.4. Does the study suggest how or if approval rates per item shared with respondents for each round should be reported in the Results section?	Hasson F, *et al. J Adv Nurs* 2000[1]<br>Humphrey-Murto S, *et al. Med Teach* 2017[2]<br>Humphrey-Murto S, *et al. J Rheumatol* 2019[3]<br>Jünger S, *et al. Palliat Med* 2017[4]<br>Boulkedid R, *et al. PLoS One* 2011[5]<br>Chan TM, *et al. CJEM* 2019[6]<br>Resemann HK, *et al. Curr Med Res Opin* 2018[9]<br>Ng J. *Value Health* 2018[14]<br>Niederberger M, *et al. Front Public Health* 2020[15]	Describe which results that were shared with respondents after each voting round were reported in the final manuscript.<br>*Could include response rates, the type of information presented, summaries of group voting and/or individual responses. If this information is not provided, this should be stated together with the rationale.*
3.5. Does the study suggest anything about in which detail the items that have been dropped should be reported? (reasons e.g.) Or if this should be reported?	Jünger S, *et al. Palliat Med* 2017[4]<br>Diamond IR, *et al. J Clin Epidemiol* 2014[7]<br>Banno M, *et al. J Clin Epidemiol* 2019[11]<br>Banno M, *et al. J Clin Epidemiol* 2020[12]<br>Niederberger M, *et al. Front Public Health* 2020[15]	A) List any voting items that were dropped.<br>B) Explain the rationale for dropping any voting items.<br>*Should state whether the criteria for dropping any items were prespecified.*
3.6. Does the study make any recommendation on how to report the collection, synthesis and use of comments from panellists? Or if this should be reported?	Humphrey-Murto S, *et al. Med Teach* 2017[2]<br>Jünger S, *et al. Palliat Med* 2017[4]<br>Boulkedid R, *et al. PLoS One* 2011[5]<br>Resemann HK, *et al. Curr Med Res Opin* 2018[9]<br>Paré G, *et al. Inf Manag* 2013[10]	Describe how responses were processed prior to reporting.<br>*Should describe methods by which responses were analysed, aggregated or summarised, include whether any statements were revised between voting rounds, and state by whom the information was processed.*
3.7. Does the study suggest regarding how the final list of items (for clinical guideline or reporting guideline) should be reported? Or if this should be reported?	Jünger S, *et al. Palliat Med* 2017[4]<br>Resemann HK, *et al. Curr Med Res Opin* 2018[9]<br>Paré G, *et al. Inf Manag* 2013[10]<br>Ng J. *Value Health* 2018[14]	Report the final outcomes.<br>*Could be quantitative (e.g. summary statistics, score means, medians and/or ranges) and/or qualitative (e.g. aggregated themes from comments). Should be clear, accurately represent the consensus methodology used, and relevant to the field.*

For peer review only

Section: Discussion

4. Discussion

Data extraction question	Articles	Checklist item(s) with brief explanation
4.1. Does the paper suggest anything about reporting the limitations and strengths of the study and how? Or if this should be reported?	Hasson F, *et al. J Adv Nurs* 2000[1]<br>Humphrey-Murto S, *et al. Med Teach* 2017[2]<br>Humphrey-Murto S, *et al. J Rheumatol* 2019[3]<br>Jünger S, *et al. Palliat Med* 2017[4]<br>Chan TM, *et al. CJEM* 2019[6]	Discuss the study's methodological strengths and limitations.<br>*Should address issues that may impact results, e.g. response rates or representation.*
4.2. Does the paper suggest anything about what or in which detail the applicability generalisability, and reproducibility of the study should be reported? Or if this should be reported?	Jünger S, *et al. Palliat Med* 2017[4]<br>Boulkedid R, *et al. PLoS One* 2011[5]<br>Niederberger M, *et al. Front Public Health* 2020[15]	A) Discuss the reliability of the study.<br>B) Discuss the sensitivity of the study.<br>C) Discuss the specificity of the study.<br>D) Discuss the applicability of the study.<br>E) Discuss the validity of the study.

Section: Additional topics

5. Additional topics

Data extraction question: Any other item proposed by the paper that is not captured in previous sections?

Articles	Checklist item(s) with brief explanation
Humphrey-Murto S, *et al. Med Teach* 2017[3]<br>Jünger S, *et al. Palliat Med* 2017[4]<br>Boulkedid R, *et al. PLoS One* 2011[5]<br>Paré G, *et al. Inf Manag* 2013[10]<br>Banno M, *et al. J Clin Epidemiol* 2020[12]	Explain any deviations from the planned protocol.<br>*Should include any affected stages, including but not limited to change in panel number or composition, number of voting rounds, stopping criteria, statistical plan, reporting of outcomes.*
Boulkedid R, *et al. PLoS One* 2011[5]<br>Resemann HK, *et al. Curr Med Res Opin* 2018[9]	Describe the formulation of questions.<br>*Should include the type of questions, e.g. open questions, numerical rating, level of agreement rating. If rating questions were used, the scale range should be stated, and whether respondents were able to leave additional comments after rating items.*
Boulkedid R, *et al. PLoS One* 2011[5]<br>Wang X, *et al. BMC Med Res Methodol* 2015[17]	Describe any group meetings that were held.<br>*Should state at what stage the meeting took place, objectives/purpose, format (e.g. face-to-face or virtual), pre-read materials shared, attendance, location, duration, and how individuals participated.*
Hasson F, *et al. J Adv Nurs* 2000[1]<br>Boulkedid R, *et al. PLoS One* 2011[5]<br>Ng J. *Value Health* 2018[14]	List any items included in the appendix accompanying the main report.<br>*Could include e.g. full voting questions from each round with response rates, or information provided to the panel as pre-reads or to summarise voting rounds.*
Boulkedid R, *et al. PLoS One* 2011[5]	State how the survey was presented to participants.<br>*For example, as hard copy or via digital platform; could include description of email or mailing process. Should describe any randomisation procedures for questions, if used. If questions were not randomised, this should be stated.*
Boulkedid R, *et al. PLoS One* 2011[5]	Describe incentives for encouraging responses.<br>*Should list any specific methods, e.g. paid return postage for the questionnaire or financial compensation.*
Boulkedid R, *et al. PLoS One* 2011[5]	State the period in which the process was conducted.
Grant S, *et al. J Clin Epidemiol* 2018[18]	Describe any prospective registrations for the consensus process.

For peer review only - http://bmjopen.bmj.com/site/about/guidelines.xhtml

Section: Additional topics

Articles	Checklist item(s) with brief explanation
	Should include the platform on which it was registered and a link, if applicable. If the process was not registered, this should be stated.
Jünger S, *et al*. *Palliat Med* 2017[4]	Describe any external peer review prior to publication.<br>*Should name the authority, state the rationale for their review, and describe any modifications made as a result of their review.*
Humphrey-Murto S, *et al. Med Teach* 2017[3]<br>Jünger S, *et al*. *Palliat Med* 2017[4]	Describe the overall process using a flow chart or diagram.
Paré G, *et al. Inf Manag* 2013[10]<br>Niederberger M, *et al. Front Public Health* 2020[15]	Explain how the initial voting items in the consensus were developed.<br>*Could describe e.g. development from empirical analyses, qualitative interviews, advance focus groups, brainstorming, or existing guidelines. Should state who consolidated the information and developed the voting items.*
Boulkedid R, *et al. PLoS One* 2011[5]	Describe the procedure for collecting participants' consent to complete the full consensus process.<br>*Could briefly describe any forms used and how the data were collected and stored.*

For peer review only

Section: References

References

1. Hasson F, Keeney S, McKenna H. Research guidelines for the Delphi survey technique. *J Adv Nurs* 2000;32:1008-15. doi: 10.1046/j.1365-2648.2000.t01-1-01567.x.
2. Humphrey-Murto S, Varpio L, Gonsalves C, et al. Using consensus group methods such as Delphi and Nominal Group in medical education research. *Med Teach* 2017;39:14-9. doi: 10.1080/0142159X.2017.1245856.
3. Humphrey-Murto S, Crew R, Shea B, et al. Consensus Building in OMERACT: Recommendations for Use of the Delphi for Core Outcome Set Development. *J Rheumatol* 2019;46:1041-6. doi: 10.3899/jrheum.181094.
4. Jünger S, Payne SA, Brine J, et al. Guidance on Conducting and REporting DElphi Studies (CREDES) in palliative care: Recommendations based on a methodological systematic review. *Palliat Med* 2017;31(8):684-706. doi: 10.1177/0269216317690685.
5. Boulkedid R, Abdoul H, Loustau M, et al. Using and Reporting the Delphi Method for Selecting Healthcare Quality Indicators: A Systematic Review. *PLoS One* 2011;6:e20476. doi: 10.1371/journal.pone.0020476.
6. Chan TM, Yarris LM, Humphrey-Murto S. Delving into Delphis. *CJEM* 2019;21:167-9. doi: 10.1017/cem.2019.3.
7. Diamond IR, Grant RC, Feldman BM, et al. Defining consensus: A systematic review recommends methodologic criteria for reporting of Delphi studies. *J Clin Epidemiol* 2014;67:401-9. doi: 10.1016/j.jclinepi.2013.12.002.
8. Humphrey-Murto S, Varpio L, Wood TJ, et al. The Use of the Delphi and Other Consensus Group Methods in Medical Education Research: A Review. *Acad Med* 2017;92:1491-8. doi: 10.1097/ACM.0000000000001812.
9. Resemann HK, Clements S, Griffiths A, et al. Reporting of Delphi methods to achieve consensus on guidelines in rare diseases. *Curr Med Res Opin* 2018;34 (Suppl 1):37.
10. Paré G, Cameron AF, Poba-Nzaou P, et al. A systematic assessment of rigor in information systems ranking-type Delphi studies. *Inf Manag* 2013;50:207-17 doi: 10.1016/j.im.2013.03.003.
11. Banno M, Tsujimoto Y, Kataoka Y. Reporting quality of the Delphi technique in reporting guidelines: a protocol for a systematic analysis of the EQUATOR Network Library. *J Clin Epidemiol* 2019;9:e024942.
12. Banno M, Tsujimoto Y, Kataoka Y. The majority of reporting guidelines are not developed with the Delphi method: a systematic review of reporting guidelines. *J Clin Epidemiol* 2020;124;50-7. doi: 10.1016/j.jclinepi.2020.04.010.
13. Gattrell WT, Clements SJ, Sheard D. Quality assessment of guidelines/recommendations developed using Delphi methodology. *Curr Med Res Opin* 2019;35 (Suppl 2):40. doi: 10.1080/03007995.2019.1583496.
14. Ng J. Delphi method: A qualitative approach for quantitative results. *Value Health* 2018;21:S54. Available at https://www.valueinhealthjournal.com/article/S1098-3015(18)30747-2/pdf.
15. Niederberger M, Spranger J. Delphi Technique in Health Sciences: A Map. *Front Public Health* 2020;8:457. doi: 10.3389/fpubh.2020.00457.

For peer review only - http://bmjopen.bmj.com/site/about/guidelines.xhtml

Section: References

16. Waggoner J, Carline JD, Durning SJ. Is There a Consensus on Consensus Methodology? Descriptions and Recommendations for Future Consensus Research. *Acad Med* 2016;91:663-8. doi: 10.1097/ACM.0000000000001092.
17. Wang X, Chen Y, Yang N, et al. Methodology and reporting quality of reporting guidelines: systematic review. *BMC Med Res Methodol* 2015;15:74. doi: 10.1186/s12874-015-0069-z.
18. Grant S, Booth M, Khodyakov D. Lack of preregistered analysis plans allows unacceptable data mining for and selective reporting of consensus in Delphi studies. *J Clin Epidemiol* 2018;99:96-105. doi: 10.1016/j.jclinepi.2018.03.007.

For peer review only

PRISMA 2020 for Abstracts Checklist

Section and Topic	Item #	Checklist item	Reported (Yes/No)
**TITLE**			
Title	1	Identify the report as a systematic review.	Yes
**BACKGROUND**			
Objectives	2	Provide an explicit statement of the main objective(s) or question(s) the review addresses.	Yes
**METHODS**			
Eligibility criteria	3	Specify the inclusion and exclusion criteria for the review.	Yes
Information sources	4	Specify the information sources (e.g. databases, registers) used to identify studies and the date when each was last searched.	Yes
Risk of bias	5	Specify the methods used to assess risk of bias in the included studies.	Not applicable
Synthesis of results	6	Specify the methods used to present and synthesise results.	Not applicable
**RESULTS**			
Included studies	7	Give the total number of included studies and participants and summarise relevant characteristics of studies.	Yes
Synthesis of results	8	Present results for main outcomes, preferably indicating the number of included studies and participants for each. If meta-analysis was done, report the summary estimate and confidence/credible interval. If comparing groups, indicate the direction of the effect (i.e. which group is favoured).	Yes
**DISCUSSION**			
Limitations of evidence	9	Provide a brief summary of the limitations of the evidence included in the review (e.g. study risk of bias, inconsistency and imprecision).	Not applicable
Interpretation	10	Provide a general interpretation of the results and important implications.	Yes
**OTHER**			
Funding	11	Specify the primary source of funding for the review.	Not in abstract, in main document
Registration	12	Provide the register name and registration number.	Yes

From: Page MJ, McKenzie JE, Bossuyt PM, Boutron I, Hoffmann TC, Mulrow CD, et al. The PRISMA 2020 statement: an updated guideline for reporting systematic reviews. BMJ 2021;372:n71. doi: 10.1136/bmj.n71

For peer review only - http://bmjopen.bmj.com/site/about/guidelines.xhtml

PRISMA 2020 for Abstracts Checklist

For more information, visit: http://www.prisma-statement.org/

For peer review only

For peer review only - http://bmjopen.bmj.com/site/about/guidelines.xhtml

PRISMA 2020 Checklist

Section and Topic	Item #	Checklist item	Location where item is reported
**TITLE**			
Title	1	Identify the report as a systematic review.	Page 1
**ABSTRACT**			
Abstract	2	See the PRISMA 2020 for Abstracts checklist.	Page 2
**INTRODUCTION**			
Rationale	3	Describe the rationale for the review in the context of existing knowledge.	Page 4, 5
Objectives	4	Provide an explicit statement of the objective(s) or question(s) the review addresses.	Page 5
**METHODS**			
Eligibility criteria	5	Specify the inclusion and exclusion criteria for the review and how studies were grouped for the syntheses.	Page 5
Information sources	6	Specify all databases, registers, websites, organisations, reference lists and other sources searched or consulted to identify studies. Specify the date when each source was last searched or consulted.	Page 6
Search strategy	7	Present the full search strategies for all databases, registers and websites, including any filters and limits used.	Online supplemental material 2
Selection process	8	Specify the methods used to decide whether a study met the inclusion criteria of the review, including how many reviewers screened each record and each report retrieved, whether they worked independently, and if applicable, details of automation tools used in the process.	Page 6
Data collection process	9	Specify the methods used to collect data from reports, including how many reviewers collected data from each report, whether they worked independently, any processes for obtaining or confirming data from study investigators, and if applicable, details of automation tools used in the process.	Page 6, 7
Data items	10a	List and define all outcomes for which data were sought. Specify whether all results that were compatible with each outcome domain in each study were sought (e.g. for all measures, time points, analyses), and if not, the methods used to decide which results to collect.	Online supplemental material 3
	10b	List and define all other variables for which data were sought (e.g. participant and intervention characteristics, funding sources). Describe any assumptions made about any missing or unclear information.	Online supplemental material 3
Study risk of bias assessment	11	Specify the methods used to assess risk of bias in the included studies, including details of the tool(s) used, how many reviewers assessed each study and whether they worked independently, and if applicable, details of automation tools used in the process.	Not applicable
Effect measures	12	Specify for each outcome the effect measure(s) (e.g. risk ratio, mean difference) used in the synthesis or presentation of results.	Not applicable
Synthesis methods	13a	Describe the processes used to decide which studies were eligible for each synthesis (e.g. tabulating the study intervention characteristics and comparing against the planned groups for each synthesis (item #5)).	Not applicable
	13b	Describe any methods required to prepare the data for presentation or synthesis, such as handling of missing summary statistics, or data conversions.	Not applicable
	13c	Describe any methods used to tabulate or visually display results of individual studies and syntheses.	Not applicable
	13d	Describe any methods used to synthesize results and provide a rationale for the choice(s). If meta-analysis was performed, describe the model(s), method(s) to identify the presence and extent of statistical heterogeneity, and software package(s) used.	Not applicable

For peer review only - http://bmjopen.bmj.com/site/about/guidelines.xhtml

PRISMA 2020 Checklist

Section and Topic	Item #	Checklist item	Location where item is reported
	13e	Describe any methods used to explore possible causes of heterogeneity among study results (e.g. subgroup analysis, meta-regression).	Not applicable
	13f	Describe any sensitivity analyses conducted to assess robustness of the synthesized results.	Not applicable
Reporting bias assessment	14	Describe any methods used to assess risk of bias due to missing results in a synthesis (arising from reporting biases).	Not applicable
Certainty assessment	15	Describe any methods used to assess certainty (or confidence) in the body of evidence for an outcome.	Not applicable
**RESULTS**			
Study selection	16a	Describe the results of the search and selection process, from the number of records identified in the search to the number of studies included in the review, ideally using a flow diagram.	Page 7 Fig 1
	16b	Cite studies that might appear to meet the inclusion criteria, but which were excluded, and explain why they were excluded.	Page 8, Fig 1
Study characteristics	17	Cite each included study and present its characteristics.	Page 8
Risk of bias in studies	18	Present assessments of risk of bias for each included study.	Not applicable
Results of individual studies	19	For all outcomes, present, for each study: (a) summary statistics for each group (where appropriate) and (b) an effect estimate and its precision (e.g. confidence/credible interval), ideally using structured tables or plots.	Table 1 and 2<br>Online supplemental material 4, 5 and 6
Results of syntheses	20a	For each synthesis, briefly summarise the characteristics and risk of bias among contributing studies.	Not applicable
	20b	Present results of all statistical syntheses conducted. If meta-analysis was done, present for each the summary estimate and its precision (e.g. confidence/credible interval) and measures of statistical heterogeneity. If comparing groups, describe the direction of the effect.	Not applicable
	20c	Present results of all investigations of possible causes of heterogeneity among study results.	Not applicable
	20d	Present results of all sensitivity analyses conducted to assess the robustness of the synthesized results.	Not applicable
Reporting biases	21	Present assessments of risk of bias due to missing results (arising from reporting biases) for each synthesis assessed.	Not applicable
Certainty of evidence	22	Present assessments of certainty (or confidence) in the body of evidence for each outcome assessed.	Not applicable
**DISCUSSION**			
Discussion	23a	Provide a general interpretation of the results in the context of other evidence.	Page 11-13
	23b	Discuss any limitations of the evidence included in the review.	Page 3, 11, 12

For peer review only

PRISMA 2020 Checklist

Section and Topic	Item #	Checklist item	Location where item is reported
	23c	Discuss any limitations of the review processes used.	Page 3, 11-13
	23d	Discuss implications of the results for practice, policy, and future research.	Page 13,14
**OTHER INFORMATION**			
Registration and protocol	24a	Provide registration information for the review, including register name and registration number, or state that the review was not registered.	Page 1, 5
	24b	Indicate where the review protocol can be accessed, or state that a protocol was not prepared.	Page 5 Online supplemental material 1 ref 13 and 15
	24c	Describe and explain any amendments to information provided at registration or in the protocol.	Online supplemental material 1
Support	25	Describe sources of financial or non-financial support for the review, and the role of the funders or sponsors in the review.	Page 14
Competing interests	26	Declare any competing interests of review authors.	Page 14
Availability of data, code and other materials	27	Report which of the following are publicly available and where they can be found: template data collection forms; data extracted from included studies; data used for all analyses; analytic code; any other materials used in the review.	Online supplemental material 1-6

From: Page MJ, McKenzie JE, Bossuyt PM, Boutron I, Hoffmann TC, Mulrow CD, et al. The PRISMA 2020 statement: an updated guideline for reporting systematic reviews. BMJ 2021;372:n71. doi: 10.1136/bmj.n71

For more information, visit: http://www.prisma-statement.org/

For peer review only

Existing guidance on reporting of consensus methodology: a systematic review to inform ACCORD guideline development

Journal:	*BMJ Open*
Manuscript ID	bmjopen-2022-065154.R3
Article Type:	Original research
Date Submitted by the Author:	15-Aug-2022

Complete List of Authors:	van Zuuren, Esther; Leiden University Medical Center, Department of Dermatology<br>Logullo, Patricia; University of Oxford, CSM (Centre for Statistics in Medicine)<br>Price, Amy; Stanford University School of Medicine<br>Fedorowicz, Zbys; Veritas Health Sciences Consultancy<br>Hughes, Ellen L; Sciwright Limited<br>Gattrell, William T; Ipsen

**Primary Subject Heading**:	Research methods
Secondary Subject Heading:	Health policy
Keywords:	Protocols & guidelines < HEALTH SERVICES ADMINISTRATION & MANAGEMENT, STATISTICS & RESEARCH METHODS, Health policy < HEALTH SERVICES ADMINISTRATION & MANAGEMENT

SCHOLARONE™
Manuscripts

I, the Submitting Author has the right to grant and does grant on behalf of all authors of the Work (as defined in the below author licence), an exclusive licence and/or a non-exclusive licence for contributions from authors who are: i) UK Crown employees; ii) where BMJ has agreed a CC-BY licence shall apply, and/or iii) in accordance with the terms applicable for US Federal Government officers or employees acting as part of their official duties; on a worldwide, perpetual, irrevocable, royalty-free basis to BMJ Publishing Group Ltd ("BMJ") its licensees and where the relevant Journal is co-owned by BMJ to the co-owners of the Journal, to publish the Work in this journal and any other BMJ products and to exploit all rights, as set out in our licence.

The Submitting Author accepts and understands that any supply made under these terms is made by BMJ to the Submitting Author unless you are acting as an employee on behalf of your employer or a postgraduate student of an affiliated institution which is paying any applicable article publishing charge ("APC") for Open Access articles. Where the Submitting Author wishes to make the Work available on an Open Access basis (and intends to pay the relevant APC), the terms of reuse of such Open Access shall be governed by a Creative Commons licence – details of these licences and which Creative Commons licence will apply to this Work are set out in our licence referred to above.

Other than as permitted in any relevant BMJ Author's Self Archiving Policies, I confirm this Work has not been accepted for publication elsewhere, is not being considered for publication elsewhere and does not duplicate material already published. I confirm all authors consent to publication of this Work and authorise the granting of this licence.

For peer review only

Existing guidance on reporting of consensus methodology: a systematic review to inform ACCORD guideline development

Esther J van Zuuren[1], Patricia Logullo[2], Amy Price[3], Zbys Fedorowicz[4], Ellen L Hughes[5], William T Gattrell[6]

[1]Department of Dermatology, Leiden University Medical Centre, Leiden, Netherlands

[2]Centre for Statistics in Medicine (CSM), University of Oxford, and EQUATOR Network UK Centre, Oxford, UK

[3]Stanford University School of Medicine, Stanford, CA, USA

[4]Veritas Health Sciences Consultancy, Huntingdon, UK

[5]Sciwright Limited, Somerset, UK

[6]Global Medical Affairs, Ipsen, Abingdon, UK

Correspondence to:
Esther J van Zuuren,
Department of Dermatology B1-Q
Leiden University Medical Centre
Albinusdreef 2
2333ZA Leiden
Netherlands
email: e.j.van_zuuren@lumc.nl

Word count: 2832

Keywords: systematic review, consensus, Delphi, reporting guideline

For peer review only

ABSTRACT

Objective: To identify evidence on the reporting quality of consensus methodology, and to select potential checklist items for the ACCORD (ACcurate COnsensus Reporting Document) project to develop a consensus reporting guideline.

Design: Systematic review.

Data sources: Embase, MEDLINE, Web of Science, PubMed, Cochrane Library, Emcare, Academic Search Premier and PsycINFO from inception until 7 January 2022.

Eligibility criteria: Studies, reviews and published guidance addressing the reporting quality of consensus methodology for improvement of health outcomes in biomedicine or clinical practice. Reports of studies using or describing consensus methods but not commenting on their reporting quality were excluded. No language restrictions were applied.

Data extraction and synthesis: Screening and data extraction of eligible studies were carried out independently by two authors. Reporting quality items addressed by the studies were synthesized narratively.

Results: Eighteen studies were included: 5 systematic reviews, 4 narrative reviews, 3 research papers, 3 conference abstracts, 2 research guidance papers and 1 protocol. The majority of studies indicated that the quality of reporting of consensus methodology could be improved. Commonly addressed items were: consensus panel composition; definition of consensus; and the threshold for achieving consensus. Items least addressed were: public patient involvement (PPI); the role of the steering committee, chair, co-chair; conflict of interest of panellists; and funding. Data extracted from included studies revealed additional items that were not captured in the data extraction form such as justification of deviation from the protocol or incentives to encourage panellist response.

Conclusion: The results of this systematic review confirmed the need for a reporting checklist for consensus methodology and provided a range of potential checklist items to report. The next step in the ACCORD project builds on this systematic review and focuses on reaching consensus on these items to develop the reporting guideline.

Protocol registration: The protocol is registered at https://osf.io/2rzm9.

For peer review only - http://bmjopen.bmj.com/site/about/guidelines.xhtml

STRENGTHS AND LIMITATIONS OF THIS STUDY

- This systematic review utilised a comprehensive search of multiple databases without language restriction.
- The included studies ranged from conference abstracts and protocols to guidelines and systematic reviews.
- For full transparency and to promote discussion, all data retrieved are reported.
- The data extraction form used may have missed a few potential reporting topics, but these will be recovered, in the following stages of the ACCORD project, by additional reviews and the Delphi panel experience.
- Conclusions are limited by the paucity of studies that provided substantial useful guidance.

For peer review only

INTRODUCTION

Healthcare providers face continuing challenges in making treatment decisions, particularly where available information on a clinical topic is limited, contradictory, or non-existent. In such situations, alternative and complementary approaches underpinned by collective judgement and based on expert consensus may be used.[1-3]

A variety of approaches with differing methodological rigour can be used to achieve consensus-based decisions. These range from informal "expert consensus meetings" to structured or systematic approaches such as the Delphi method and the Nominal Group Technique (NGT). These methods can be used for generating ideas or determining priorities and aim to achieve consensus through voting on a series of multiple-choice questions.[4-7] The voting process varies according to the method and may take place anonymously (as in Delphi) and/or face to face (in NGT and consensus conferences).[8-10] Key elements in the process include the use of valid and reliable methods to reach consensus and subsequently their transparent reporting; however, these aspects are seldom clearly and explicitly reported.[3, 11]

Reporting guidelines have been developed and are in use for the majority of study designs, e.g. PRISMA, CONSORT and STROBE (for all existing reporting guidelines see: https://www.equator-network.org/). However, no research reporting guideline exists for studies involving consensus methodology other than best practice guidance for Delphi studies in palliative care.[12] Guidelines should include "a checklist, flow diagram, or explicit text to guide authors in reporting a specific type of research, developed using explicit methodology".[3]

Deficiencies in the reporting of consensus methods have been well documented in the literature and are referred to in the protocol for the ACCORD (ACcurate COnsensus Reporting Document) project, which aims to develop a reporting guideline for methods used to reach consensus.[13] In accordance with the EQUATOR Network guidance in the toolkit for the development of reporting guidelines, the

For peer review only - http://bmjopen.bmj.com/site/about/guidelines.xhtml

next step for the ACCORD project was a review of the relevant literature, which would ultimately inform the voting process.[3]

Our objective was to undertake a thorough and comprehensive systematic review that seeks to identify evidence on the quality of reporting of consensus methodology, for subsequent development into a draft checklist of items for the ACCORD guideline. This ACCORD reporting guideline will assist the biomedical research and clinical practice community to describe the methods used to reach consensus in a complete, transparent, and consistent manner.

METHODS

This manuscript conforms to the Preferred Reporting Items for Systematic Reviews and Meta-Analyses (PRISMA) statement,[14] and follows a prespecified protocol.[13] The protocol was registered on 12 October 2021 at the Open Science Framework (OSF).[15]

Inclusion criteria

Eligible studies consisted of reviews and published guidance which addressed the reporting quality of consensus methodology and aimed to improve health outcomes in biomedicine or clinical practice.

Exclusion criteria

Excluded were publications using consensus methods or describing consensus methods, or discussing the advantages or disadvantages of frameworks, procedures, or techniques to reach consensus, without specifically addressing reporting quality. Examples include guidelines developed through the use of consensus methodologies, such as reporting guidelines, clinical practice guidelines or core outcome set development studies. Editorials (usually brief opinion-based comments), letters about individual publications, and commentaries on consensus methods outside the scope of biomedical research (for example, in the social sciences, economy, politics or marketing) were also excluded for this systematic review.

Literature search strategy and data sources

For peer review only - http://bmjopen.bmj.com/site/about/guidelines.xhtml

For peer review only

A systematic literature search was conducted on 7 January 2022 by a biomedical information specialist. The following bibliographical databases were searched: MEDLINE (OVID version), Embase (OVID version), PubMed, Web of Science, MEDLINE (Web of Science), Cochrane Library, Emcare (OVID version), PsycINFO (EbscoHOST version) and Academic Search Premier. The full search strategy is presented in Supplementary Material 1.

We (EJvZ, ZF, PL and WTG) piloted four initial search strategies provided by the information specialist (JWS, see Acknowledgements). The initial search strategy was sensitive and precise, producing the highest number of retrieved references (N = 7951). After several rounds of checking through known relevant references and controlling for the effect of the performance of certain search terms, modifications were made, including the use of the most explicit terms in the most specific search fields. The performance of search terms was investigated from two vantage points: homonymy (same search term, but different meaning), and, particularly, loss-of-context (right meaning of the word, but not in the correct context). This extended search strategy provided extra 'signal', but also reduced the level of 'noise'. We chose to use specific rather than broad terms (for example, not using the singular terms "delphi" and "consensus" instead we included these words with relevant phrases or with other contextual words). In this way, the refined search strategy was better aligned with our inclusion criteria and the objectives of the systematic review.

Selection process

The final search results were uploaded to Rayyan (https://rayyan.ai) in the blind mode for independent screening by four review authors (EJvZ, ZF, PL, WTG) based on titles and abstracts. No language restrictions were applied. Records deemed eligible or without sufficient detail to make a clear judgement, we retrieved as full-text articles (EJvZ). The same four reviewers independently reassessed the eligibility of these full-text papers and any discrepancies were resolved through discussion. The references of the included studies were also checked for additional potentially eligible studies (EJvZ).

For peer review only - http://bmjopen.bmj.com/site/about/guidelines.xhtml

Data extraction, collection of items and synthesis

Study details and outcome data from the included studies were collected independently within Covidence (https://www.covidence.org/) by two authors using a piloted data extraction form (EJvZ, WTG). The data extraction form questions were compiled based on the review authors' own experiences with reporting quality evaluation and literature on consensus methodology. Furthermore, two additional free text fields were created for extractors to present issues addressed by the included studies that were not captured by the other questions, and for others that the extractors felt were not directly addressed by the studies but were rather inferences about topics that could be potential issues in the reporting of consensus methods. Disagreements were discussed and reconciled by consultation with a third and fourth author (ZF and AP).

The following details were extracted: bibliographic details and reporting items including any suggestions and comments regarding reporting items. Reporting items were divided into the component parts of background, methods, results and discussion, each addressing key aspects of consensus methodology. We also included a section for additional items retrieved from the studies and not captured in the data extraction form. The complete data extraction form can be found as Supplementary Material 2.

The topics extracted and the methods used in the studies included are synthetised narratively, in text and tables (and Supplementary Material). No further analyses were carried out but these will follow during the next stage of the ACCORD project as per protocol.[13]

Patient and public involvement

We involved patients, advocates, and members of the lay public in the initial phases of this protocol [13, 15], as collaborators to develop this project and to co-produce the systematic review and co-author the manuscript. They are collaborating with us by offering their experience with the use of consensus methods to develop guidelines and also systematic reviews. These contributors will work with us to disseminate the results.

For peer review only - http://bmjopen.bmj.com/site/about/guidelines.xhtml

RESULTS

Our searches across the databases identified 2599 articles and 137 further references to abstracts totalling 2736 references (after removal of duplicates) (see Figure 1). A total of 2682 records were excluded after examination of titles and abstracts. Full-text copies of 54 studies were obtained for further assessment of eligibility, and finally, just 18 eligible studies were included. Checking of the references of these full-text publications did not yield any additional eligible articles.

Characteristics of included studies

Eighteen studies matched our prespecified eligibility criteria and were finally included in this review. These studies comprised five systematic reviews,[12, 16-19] four reviews,[20-23] three research papers,[24-26] two research guidelines/guidance,[27, 28] three conference abstracts,[29-31] and one protocol.[32] Of the 18 included studies, 4 used Delphi plus other consensus methods [19, 21, 23 and 28] and the remaining 14 were primarily focused on only the Delphi method.[12, 16-19, 20, 22, 24-27, 29, 30]

Characteristics of excluded studies

A total of 36 studies were excluded.[33- 68] The main reasons for their exclusion were: that they discussed (modified) Delphi methodology but did not include aspects of reporting;[33-54] that they covered reporting but not on consensus methodology;[55-58] that various other consensus methodologies were discussed but not their reporting;[59-67] and that only the concept of experts in consensus methodology was discussed.[68]

Data extraction and narrative synthesis

The majority of studies indicated that reporting of consensus methods could be improved overall. The authors of these studies summarised some current limitations in reporting or proposed suggestions for improvement. Often there were common generic comments that noted reporting of consensus methodologies is inconsistent or lacks transparency. The studies provided few examples of areas that could be reported in more detail such as: selection criteria for the participants and information about the participants; background information for panellists; definition of consensus;

For peer review only - http://bmjopen.bmj.com/site/about/guidelines.xhtml

response rates after each round; description of level of anonymity or how anonymity was maintained; and feedback between rounds (see Table 1).

Table 1. Data on reporting quality of consensus methodologies

Items that are not or not adequately reported in sufficient detail	
Selection criteria for participants/information about the participants [16, 19, 23, 26, 32]	Statement that anonymity was maintained or level of anonymity [[20, 21, 25, 28, 29, 32]
Literature review [20, 21, 31]	Type of consensus method used [29]
Background information for participants [20, 21, 25, 28]	Threshold of consensus [29]
Recruitment strategies [19, 22]	How questionnaire was developed [26]
Criteria for number of rounds [16, 26]	Pretesting of instruments [19, 32]
Stopping criteria [16, 32]	Analysis procedure [24, 32]
Feedback after rounds [17, 20, 21, 23, 25, 26, 28, 31, 32]	Changes to registered pre-analysis plan [24]
Rating scales used [31]	Reporting final number of list of items [32]
Criteria for dropping items [26]	Conflict of interest of panellists [29]
Response rates for each round [17 20, 21, 25, 26, 28, 32]	Funding source [29]
Definition of consensus [17-19, 21, 23, 25, 26, 28]	External support [29]
Level of consensus reached [19, 31]	Generic comments that reporting needs improvement [12, 17, 26, 30]

The studies we reviewed did not provide a systematic or standardised evaluation of the quality of reporting, but they did evaluate the literature critically and offered insights into the gaps of information about consensus. Fifteen papers made recommendations sometimes in the form of short lists —based solely on the authors' opinion, rather than using a systematic approach to reporting guidance development.[12, 16-25, 27, 28, 30, 32] Detailed statements regarding quality of reporting are reproduced in Supplementary Material 3.

In Table 2, we summarise the results of the data extraction, which correlates the corresponding aspects of consensus reporting ("items") to the studies that address them. The items in the table are presented in the format used in the data extraction form (see Supplementary Material 2).

For peer review only - http://bmjopen.bmj.com/site/about/guidelines.xhtml

Table 2. Studies providing guidance for reporting items in the extraction form of this systematic review

Reporting Items	Studies that provide guidance	
Background	Number	References
1.1 Rationale for choosing a consensus method over other methods	4	[12, 25, 27, 28]
1.2 Clearly defined objective	6	[12, 17, 18, 20, 27, 28]
Methods		
2.1 Review of existing evidence informing consensus study	5	[20, 21, 27, 28, 31]
2.2 Inclusion and exclusion criteria of the literature search	3	[17, 20, 22]
2.3 Composition of the panel	16	[12, 16-23, 25-30, 32]
2.4 Public patient involvement (PPI)	0	
2.5 Panel recruitment	4	[12, 17, 22, 23]
2.6 Defining consensus and the threshold for achieving consensus	13	[12, 17-21, 23-29]
2.7 Decision of item approval	3	[12, 17, 27]
2.8 Number of voting rounds	10	[12, 16, 18, 20, 21, 23, 26-28, 32]
2.9 Rationale for number of voting rounds	8	[16, 20, 21-23, 25, 26, 28]
2.10 Time between voting rounds	1	[17]
2.11 Additional methods used alongside consensus	2	[17, 23]
2.12 Software or tools used for voting	1	[25]
2.13 Anonymity of panellists and how this was maintained	7	[16, 20-22, 25, 28, 29]
2.14 Feedback to panellists at the end of each round	11	[17, 19-22, 25-29, 31]
2.15 Synthesis/analysis of responses after voting rounds	5	[12, 22-24, 30]
2.16 Pilot testing of study material/instruments	3	[12, 22, 28]
2.17 Role of the steering committee/chair/co-chair/facilitator	0	
2.18 Conflict of interest or funding received	4	[12, 29, 30, 32]
2.19 Measures to avoid influence by conflict of interest	1	[12]
Results		
3.1 Results of the literature search	1	[12]
3.2 Number of studies found as supporting evidence	0	
3.3 Response rates per voting round	5	[12, 21, 22, 25, 30]
3.4 Results shared with respondents	9	[12, 17, 20, 25-28, 30, 31]
3.5 Dropped items	5	[12, 16, 18, 26, 32]
3.6 Collection, synthesis and comments from panellists	5	[12, 17, 22, 28, 31]
3.7 Final list of items (e.g. for guideline or reporting guideline)	4	[12, 22, 30, 31]
Discussion		
4.1 Limitations and strengths of the study	5	[12, 20, 25, 27, 28]
4.2 Applicability, generalizability, reproducibility	3	[12, 17, 26]

For peer review only

The most frequently addressed item in the included studies (16 times) was the composition of and the criteria for selecting the panellists, including their demographics; specifically, age, gender, specialty, years of experience, and sociodemographic background. The aspects of clarity in, and the importance of, defining consensus and the corresponding thresholds to reach that consensus were addressed in 13 studies. The prespecified number of voting rounds and provision of feedback to the panellists at the end of each round were addressed in 10 and 11 of the studies, respectively.

For peer review only - http://bmjopen.bmj.com/site/about/guidelines.xhtml

None of the included studies reported or made reference to public patient involvement (PPI). The roles of the steering committee/chair/co-chair were not defined in any of the included studies. Reporting of the time interval between voting rounds, panel members' conflicts of interest (COI) and funding sources, as well as the measures used to avoid the influence of COI on voting and decision-making, were minimally addressed.

Conversely, three studies addressed between 12 and 19 reporting items of the 30 items present in the data extraction form of this review,[12, 19, 28] whereas two studies covered only two or three items.[19, 24] We identified a considerable number of other aspects of reporting that were proposed in the included studies, but which were not captured in our data extraction form. These included: 'justifications for deviating from the protocol', 'incentives for encouraging panellists to respond', and 'suggestions to add a flow chart of the consensus process'. All extracted data can be found in Supplementary Material 4 and 5.

DISCUSSION

Although consensus methodology is widely used in healthcare and researchers do raise poor reporting as an issue, we were able to identify only 18 studies that commented on reporting quality and/or provided suggestions to improve the quality of reporting of consensus methodology. These included studies ranged from conference abstracts and protocols to guidelines and systematic reviews. Only four studies covered methods other than the Delphi method and thus providing very limited guidance on other consensus methodologies. We carried out a comprehensive search of the most commonly used databases for systematic reviews without language restriction. However, during peer review of the present manuscript, three studies were brought to our attention as potentially eligible for inclusion.[69-71] Two of the studies had been excluded at the screening stage.[69, 70] After full-text evaluation, one of the articles did discuss reporting quality but failed to make that clear in the title or abstract;[69] however, the findings were consistent with our reported results. The second publication did not meet our eligibility criteria because it focussed on studies of

For peer review only - http://bmjopen.bmj.com/site/about/guidelines.xhtml

health economics rather than health outcomes.[70] Interestingly, the study identified similar gaps to the present study, but its scope is outside our protocol and research question. The third was not picked up during screening because the journal is not indexed in the nine pre-defined data sources for the searches.[71]

The data extraction form may have missed a few potential reporting topics — which will be recovered, in the next stages of the ACCORD project, by additional reviews and the Delphi panel experience. Furthermore, one study was published after our search date, showing that the development of reporting guidelines for consensus methodologies is an active area, with more studies being published on the topic continuously, which could inform future stages or updates of ACCORD.[72] Comments regarding deficient reporting from the included studies varied from generic statements such as “reporting could be improved” to rather specific comments of which aspects of consensus methods were inadequately or not reported. Far more detailed data were provided regarding guidance to improve reporting quality or suggestions for items that require reporting. Both composition and characteristics of the panel, and defining consensus and threshold for achieving assessment received, were consistently addressed and appeared to be critical items that should be reported in sufficient detail. Feedback to the panel might be considered an important aspect of ensuring ongoing engagement with the panellists, transparency and replicability of methods; thus, it was somewhat surprising to see just 11 of the 18 studies consider this an element of consensus methodology worth reporting.

Some items were not addressed in any of the studies, specifically PPI, which is currently considered a key element in the shared decision-making process and is a component of guideline development.[73] Just four studies made reference to the COI of panel members and project funding. COI of panellists, as well as of chair, co-chair and steering committee, can directly or indirectly impact and influence decision-making during the various steps of consensus methodology. As such, COI remains underreported and is often inconsistently described.[74] This also raises

For peer review only - http://bmjopen.bmj.com/site/about/guidelines.xhtml

concerns about the measures that can be taken to mitigate the potential influence of COI and to ensure that those panellists who do have relevant interests are, for example, not able to vote on pertinent items. For full transparency and to promote discussion, all data retrieved are reported as supplementary material (Supplementary Material 3–5).

Although conclusions are limited by the paucity of studies, a few were particularly informative. The first was a systematic review on the use and reporting of the Delphi method for selecting healthcare indicators.[17] Specifically, this review not only provided guidance for planning and using the Delphi procedure but additionally formulated general recommendations for reporting. The second study was a guidance report on consensus methods such as Delphi and NGT, which were used in medical education research.[28] The authors reported that there is a lack of "standardization in definitions, methodology and reporting" and proposed items for researchers to consider when using consensus methods to improve methodological rigour as well as the reporting quality. However, it is worth noting that none of these studies followed the EQUATOR Network guidance for the development of a reporting guideline.[3]

The third study we would like to highlight is the Guidance on Conducting and REporting DElphi Studies (CREDES) in palliative care, which was based on a methodological systematic review.[12] This study focused on the development of guidance in palliative care, although it may not be suitable for extrapolation to other biomedical areas. Furthermore, this study only considered the Delphi methodology, whereas we included studies covering consensus processes involving non-Delphi based methods or "modified Delphi" in our review (and in the ACCORD project overall). However, many of the suggestions made regarding the design and conduct of Delphi studies in addition to recommendations for reporting are equally applicable to our ACCORD project. These items will be used and integrated into the next step of the project, which is the development of a reporting checklist on consensus methods.

For peer review only - http://bmjopen.bmj.com/site/about/guidelines.xhtml

Two additional studies proved to be of particular value.[21, 25] One provided a preliminary Delphi checklist to be used for Outcome Measures in Rheumatology (OMERACT).[25] The other concluded, in a scoping review that consensus methods are "poorly standardized and inconsistently used" and exposed reporting flaws in consensus reports.[21]

CONCLUSION

The principal objectives of this systematic review were to conduct a comprehensive search and to identify the existing evidence on the quality of reporting of consensus methodology. As such, we have been able to gather together all relevant studies, summarise the existing research, and highlight key gaps in the current evidence base on consensus methods. This systematic review will ultimately inform the generation of a draft checklist of items for the development steps of the ACCORD reporting guideline.

Acknowledgments

We would like to thank Jan W. Schoones of the Directorate of Research Policy (formerly: Walaeus Library), Leiden University Medical Centre, Leiden, The Netherlands, for his assistance in the development of the search strategy. Furthermore, we would like to thank the other members of the ACCORD project Steering Committee for their contributions to the protocol: Amrit Pali Hungin, Christopher C Winchester, David Tovey, Keith Goldman, Rob Matheis and Niall Harrison. We are grateful for the editorial guidance and reviewer insights from the BMJ Open team. We agree their observations improved the manuscript.

Competing interests

PL is a member of the UK EQUATOR Centre, an organization that promotes the use of reporting guidelines, many of which are developed using consensus methods, and she is personally involved in the development of other reporting guidelines. ELH has worked with Ogilvy Health UK on consensus projects. WTG is a former employee of Ipsen and is now employed by Bristol Myers Squib. AP is an editor at the BMJ, EJvZ and ZF have no conflict of interest.

For peer review only - http://bmjopen.bmj.com/site/about/guidelines.xhtml

Funding

PL contributed to this study using her time funded by CRUK. WTG contributed his time with the agreement of his employers. Apart from these authors, this study was conducted without external funding for the development of the study design; for the collection, analysis and interpretation of the data; or for the writing of the report. As ZF (Veritas Health Sciences Consultancy) acted as an independent consultant for this work, his support for data extraction and tabulation was funded by Oxford PharmaGenesis. Support for Open Access was also provided by Oxford PharmaGenesis. The decision to submit the paper for publication was made solely by the authors.

Contributors

EJvZ, PL, ZF and WTG contributed to the screening and agreed on the inclusion of studies. EJvZ and WTG extracted data from the included studies. AP, ZF and ELH contributed to the discussion of extracted data and interpretation. EJvZ was the major contributor in the review of studies, data extraction, interpretation of findings as well as writing of the manuscript. All authors read the final manuscript, provided feedback and approved the final manuscript. The author EJvZ is the guarantor.

Patient consent for publication

Not applicable.

Ethics approval

No patient-level data were used in this study and no ethical approval was sought.

Provenance and peer review

Not commissioned; externally peer reviewed.

Dissemination

For peer review only - http://bmjopen.bmj.com/site/about/guidelines.xhtml

A link to this research will be made available on the EQUATOR website and the ACCORD research page, shared via social media, shared with future Delphi respondents and stored in local online institutional repositories.

Data availability statement

All key data for this study are included in this article or uploaded as online supplementary information. The ACCORD protocol has been listed on the EQUATOR website (Reporting guidelines under development for other study designs | The EQUATOR Network (equator-network.org)) and registered with the Open Science Framework (https://osf.io/2rzm9).

Open access

This is an open access article distributed in accordance with the terms of the Creative Commons Attribution (CC BY 4.0) license, which permits others to distribute, remix, adapt and build upon this work, for commercial use, provided the original work is properly cited. See: http://creativecommons.org/licenses/by/4.0.

ORCID iDs

EJvZ: https://orcid.org/0000-0002-4780-0182

PL: https://orcid.org/0000-0001-8708-7003

AP: https://orcid.org/0000-0002-5200-7322

ZF: https://orcid.org/0000-0003-3952-965X

ELH: https://orcid.org/0000-0003-2012-0037

WTG: https://orcid.org/0000-0002-9733-6330

REFERENCES

1. Raine R, Sanderson C, Black N. Developing clinical guidelines: a challenge to current methods. *BMJ* 2005;331:631-3. doi: 10.1136/bmj.331.7517.631.

2. Djulbegovic B, Guyatt G. Evidence vs Consensus in Clinical Practice Guidelines. *JAMA* 2019;322:725-6.doi: 10.1001/jama.2019.9751.
3. Moher D, Schulz KF, Simera I, Altman DG. Guidance for developers of health research reporting guidelines. *PLoS Med* 2010;7:e1000217. doi:10.1371/journal.pmed.1000217.
4. Dalkey N, Helmer O. An Experimental Application of the DELPHI Method to the Use of Experts. Manage Sci. 1963;9:458-467. https://doi.org/10.1287/mnsc.9.3.458.
5. Murphy MK, Black NA, Lamping DL, et al. Consensus development methods, and their use in clinical guideline development. *Health Technol Assess* 1998;2:i-iv, 1-88. doi: 10.3310/hta2030.
6. Foth T, Efstathiou N, Vanderspank-Wright B, et al. The use of Delphi and Nominal Group Technique in nursing education: A review. *Int J Nurs Stud* 2016;60:112-20. doi: 10.1016/j.ijnurstu.2016.04.015.
7. Nair R, Aggarwal R, Khanna D. Methods of formal consensus in classification/diagnostic criteria and guideline development. *Semin Arthritis Rheum* 2011;41:95-105. doi: 10.1016/j.semarthrit.2010.12.001.
8. McMillan SS, King M, Tully MP. How to use the nominal group and Delphi techniques. *Int J Clin Pharm* 2016;38:655-62. doi: 10.1007/s11096-016-0257-x.
9. Grant S, Armstrong C, Khodyakov D. Online Modified-Delphi: a Potential Method for Continuous Patient Engagement Across Stages of Clinical Practice Guideline Development. *J Gen Intern Med* 2021;36:1746-50. doi: 10.1007/s11606-020-06514-6.
10. Helmer-Hirschberg O. Analysis of the future: The Delphi Method. Santa Monica, CA: RAND Corporation, 1967. Available at https://www.rand.org/pubs/papers/P3558.htm.
11. Arakawa N, Bader LR Consensus development methods: Considerations for national and global frameworks and policy development. *Res Social Adm Pharm* 2022;18:2222-9. doi: 10.1016/j.sapharm.2021.06.024.
12. Jünger S, Payne SA, Brine J, et al. Guidance on Conducting and REporting DElphi Studies (CREDES) in palliative care: Recommendations based on a methodological systematic review. *Palliat Med* 2017;31(8):684-706. doi: 10.1177/0269216317690685.
13. Gattrell WT, Hungin AP, Price A, et al. ACCORD guideline for reporting consensus-based methods in biomedical research and clinical practice: a study protocol. *Res Integr Peer Rev* 2022;7(1):3. doi: 10.1186/s41073-022-00122-0.
14. Page MJ, McKenzie JE, Bossuyt PM, et al. The PRISMA 2020 statement: an updated guideline for reporting systematic reviews. *BMJ* 2021;372:n71. doi: 10.1136/bmj.n71.
15. Gattrell W, Hungin AP, Arents BWMA, et al. Consensus-based methods in biomedical research and clinical practice: The ACCORD study protocol for establishing a reporting guideline. https://osf.io/e5w9n/ (accessed 19 April 2022).
16. Banno M, Tsujimoto Y, Kataoka Y. The majority of reporting guidelines are not developed with the Delphi method: a systematic review of reporting guidelines. *J Clin Epidemiol* 2020;124;50-7. doi: 10.1016/j.jclinepi.2020.04.010.
17. Boulkedid R, Abdoul H, Loustau M, et al. Using and Reporting the Delphi Method for Selecting Healthcare Quality Indicators: A Systematic Review. *PLoS One* 2011;6:e20476. doi: 10.1371/journal.pone.0020476.
18. Diamond IR, Grant RC, Feldman BM, et al. Defining consensus: A systematic review recommends methodologic criteria for reporting of Delphi studies. *J Clin Epidemiol* 2014;67:401-9. doi: 10.1016/j.jclinepi.2013.12.002.

For peer review only - http://bmjopen.bmj.com/site/about/guidelines.xhtml

19. Wang X, Chen Y, Yang N, et al. Methodology and reporting quality of reporting guidelines: systematic review. *BMC Med Res Methodol* 2015;15:74. doi: 10.1186/s12874-015-0069-z
20. Chan TM, Yarris LM, Humphrey-Murto S. Delving into Delphis. *CJEM* 2019;21:167-9. doi: 10.1017/cem.2019.3.
21. Humphrey-Murto S, Varpio L, Wood TJ, et al. The Use of the Delphi and Other Consensus Group Methods in Medical Education Research: A Review. *Acad Med* 2017;92:1491-8. doi: 10.1097/ACM.0000000000001812.
22. Paré G, Cameron AF, Poba-Nzaou P, et al. A systematic assessment of rigor in information systems ranking-type Delphi studies. *Inf Manag* 2013;50:207-17 doi: 10.1016/j.im.2013.03.003.
23. Waggoner J, Carline JD, Durning SJ. Is There a Consensus on Consensus Methodology? Descriptions and Recommendations for Future Consensus Research. *Acad Med* 2016;91:663-8. doi: 10.1097/ACM.0000000000001092.
24. Grant S, Booth M, Khodyakov D. Lack of preregistered analysis plans allows unacceptable data mining for and selective reporting of consensus in Delphi studies. *J Clin Epidemiol* 2018;99:96-105. doi: 10.1016/j.jclinepi.2018.03.007.
25. Humphrey-Murto S, Crew R, Shea B, et al. Consensus Building in OMERACT: Recommendations for Use of the Delphi for Core Outcome Set Development. *J Rheumatol* 2019;46:1041-6. doi: 10.3899/jrheum.181094.
26. Niederberger M, Spranger J. Delphi Technique in Health Sciences: A Map. *Front Public Health* 2020;8:457. doi: 10.3389/fpubh.2020.00457.
27. Hasson F, Keeney S, McKenna H. Research guidelines for the Delphi survey technique. *J Adv Nurs* 2000;32:1008-15. doi: 10.1046/j.1365-2648.2000.t01-1-01567.x.
28. Humphrey-Murto S, Varpio L, Gonsalves C, et al. Using consensus group methods such as Delphi and Nominal Group in medical education research. *Med Teach* 2017;39:14-9. doi: 10.1080/0142159X.2017.1245856.
29. Gattrell WT, Clements SJ, Sheard D. Quality assessment of guidelines/recommendations developed using Delphi methodology. *Curr Med Res Opin* 2019;35 (Suppl 2):40. doi: 10.1080/03007995.2019.1583496.
30. Ng J. Delphi method: A qualitative approach for quantitative results. *Value Health* 2018;21:S54. Available at https://www.valueinhealthjournal.com/article/S1098-3015(18)30747-2/pdf.
31. Resemann HK, Clements S, Griffiths A, et al. Reporting of Delphi methods to achieve consensus on guidelines in rare diseases. *Curr Med Res Opin* 2018;34 (Suppl 1):37.
32. Banno M, Tsujimoto Y, Kataoka Y. Reporting quality of the Delphi technique in reporting guidelines: a protocol for a systematic analysis of the EQUATOR Network Library. *J Clin Epidemiol* 2019;9:e024942.
33. Bellali T, Karamitri I. The Delphi research methodology and its applications in the healthcare sciences. *Arch Hellen Med* 2011;28:839-48. Available at http://www.mednet.gr/archives/2011-6/pdf/839.pdf.
34. Bijl R. Delphi in a future scenario study on mental health and mental health care. *Futures* 1992;24:232-50. doi: 10.1016/0016-3287(92)90033-C.
35. Boel A, Navarro-Compán V, Landewé R, et al. Two different invitation approaches for consecutive rounds of a Delphi survey led to comparable final outcome. *J Clin Epidemiol* 2021;129:31-9. doi: 10.1016/j.jclinepi.2020.09.034.

For peer review only - http://bmjopen.bmj.com/site/about/guidelines.xhtml

36. Bowles N. The Delphi technique. *Nurs Stand* 1999;13:21-6. doi: 10.7748/ns1999.07.13.45.32.c2650.
37. Cramer CK, Klasser GD, Epstein JB, et al. The Delphi process in dental research. *J Evid Based Dent Pract* 2008;8:211-20. doi: 10.1016/j.jebdp.2008.09.002.
38. de Loë R, Melnychuk N, Murray D, et al. Advancing the State of Policy Delphi Practice: A Systematic Review Evaluating Methodological Evolution, Innovation, and Opportunities. *Technol Forecast Soc Change* 2016;104:78-88. doi: 10.1016/j.techfore.2015.12.009.
39. Drumm S, Bradley C, Moriarty F. 'More of an art than a science'? The development, design and mechanics of the Delphi Technique. *Res Social Adm Pharm* 2022;18:2230-6. doi: 10.1016/j.sapharm.2021.06.027.
40. Fink-Hafner D, Dagan T, Doušak M, et al. Delphi Method: Strengths and Weaknesses. *Metodoloski Zv* 2019;16:1-19. doi: 10.51936/fcfm6982.
41. Franklin KK, Hart JK. Idea Generation and Exploration: Benefits and Limitations of the Police Delphi Research Method. *Innov High Educ* 2006;31:237-46. 10.1007/s10755-006-9022-8.
42. Guzys D, Dickson-Swift V, Kenny A, et al. Gadamerian philosophical hermeneutics as a useful methodological framework for the Delphi technique. *Int J Qual Stud Health Well-being* 2015;10:26291. doi: 10.3402/qhw.v10.26291.
43. Hallowell MR, Gambatese JA. Qualitative Research: Application of the Delphi Method to CEM Research. *J Constr Eng Manag* 2010;136:99-107. doi: 0.1061/(ASCE)CO.1943-7862.0000137.
44. Holloway K. Doing the E-Delphi: using online survey tools. *Comput Inform Nurs* 2012;30:347-50. doi: 10.1097/NXN.0b013e31825e8923.
45. Hung HL, Altschuld JW, Lee YF. Methodological and conceptual issues confronting a cross-country Delphi study of educational program evaluation. *Eval Program Plann* 2008;31:191-8. doi: 10.1016/j.evalprogplan.2008.02.005.
46. Ibiyemi AO, Adnan YM, Daud MN. The validity of the classical Delphi applications for assessing the industrial sustainability-correction factor: an example study. *Foresight* 2016;18:603-24. doi: 10.1108/FS-04-2016-0016.
47. Keeney S, Hasson F, McKenna HP. Consulting the oracle: ten lessons from using the Delphi technique in nursing research. *J Adv Nurs* 2006;53:205-12. doi: 10.1111/j.1365-2648.2006.03716.x.
48. Keeney S, Hasson F, McKenna HP. A critical review of the Delphi technique as a research methodology for nursing. *Int J Nurs Stud* 2001;38:195-200. doi: 10.1016/S0020-7489(00)00044-4.
49. Kennedy HR. Enhancing Delphi research: methods and results. *J Adv Nurs* 2004;45:504-11. doi: 10.1046/j.1365-2648.2003.02933.x.
50. Khodyakov D, Grant S, Denger B, et al. Practical Considerations in Using Online Modified-Delphi Approaches to Engage Patients and Other Stakeholders in Clinical Practice Guideline Development. *Patient* 2020;13:11-21. doi: 10.1007/s40271-019-00389-4.
51. Murry J, Hammons J. Delphi: A versatile methodology for conducting qualitative research. *Rev High Educ* 2017;18:423-36. doi: 10.1353/rhe.1995.0008.
52. Olsen AA, Wolcott MD, Haines ST, et al. How to use the Delphi method to aid in decision making and build consensus in pharmacy education. *Curr Pharm Teach Learn* 2021;13:1376-85. doi: 10.1016/j.cptl.2021.07.018.

For peer review only - http://bmjopen.bmj.com/site/about/guidelines.xhtml

53. Vernon W. The Delphi technique: A review. *Int J Ther Rehabil* 2009;16:69-76. doi: 10.12968/ijtr.2009.16.2.38892.
54. Von der Gracht HA. Consensus measurement in Delphi studies Review and implications for future quality assurance. Technol Forecast Soc Change 2012;79:1525-36. doi: 10.1016/j.techfore.2012.04.013.
55. Bennett C, Khangura S, Brehaut JC, et al. Reporting guidelines for survey research: an analysis of published guidance and reporting practices. *PloS Med* 2010;8:e1001069. doi: 10.1371/journal.pmed.1001069.
56. Han SJ, Oh MC, Sughrue ME, et al. Reporting standard compliance in publications of vestibular schwannoma patients treated with microsurgery. *Otol Neurotol* 2015;2012:33:648-50. doi: 10.1097/MAO.0b013e31824f998b.
57. Moher D, Weeks L, Ocampo M, et al. Describing reporting guidelines for health research: a systematic review. *J Clin Epidemiol* 2011;64:718-42. doi: 10.1016/j.jclinepi.2010.09.013.
58. Murray A, Junge A, Robinson PG, et al. International consensus statement: methods for recording and reporting of epidemiological data on injuries and illnesses in golf. Br J Sports Med 2020;54:1136-41. doi: 10.1136/bjsports-2020-102380.
59. Campbell SM, Cantrill JA. Consensus methods in prescribing research. *J Clin Pharm Ther* 2001;26:5-14. doi: 10.1046/j.1365-2710.2001.00331.x.
60. Durkin J, Usher K, Jackson D. Using consensus from experts to inform a shared understanding of subjective terms. *Nurse Res* 2019;27:46-9. doi: 10.7748/nr.2019.e1622.
61. Fink A, Kosecoff J, Chassin M, et al. Consensus methods: characteristics and guidelines for use. *Am J Public Health* 1984;74:979-83. doi: 0.2105/ajph.74.9.979.
62. Halcomb E, Davidson P, Hardaker L. Using the consensus development conference method in healthcare research. *Nurs Res* 2008;16:56-71. doi: 10.7748/nr2008.10.16.1.56.c6753.
63. James D, Warren-Forward H. Research methods for formal consensus development. *Nurs Res* 2015;22:35-40. doi: 10.7748/nr.22.3.35.e1297.
64. McMillan SS, King M, Tully MP. How to use the nominal group and Delphi techniques. *Int J Clin Pharm* 2016;38:655-62. doi: 10.1007/s11096-016-0257-x.
65. Nair R, Aggarwal R, Khanna D. Methods of Formal Consensus in Classification/Diagnostic Criteria and Guideline Development. *Semin Arthritis Rheum* 2011;41:95-105. doi: 10.1016/j.semarthrit.2010.12.001.
66. Rotondi AJ, Kvetan V, Carlet J, et al. Consensus conferences in critical care medicine - Methodologies and impact. *Crit Care Clin* 1997;13:417-39. doi: 10.1016/s0749-0704(05)70319-5.
67. van Teijlingen E, Pitchforth E, Bishop C, et al. Delphi method and nominal group technique in family planning and reproductive health research. *J Fam Plann Reprod Health Care* 2006;32:249-52. doi: 10.1783/147118906778586598.
68. Baker J, Lovell K, Harris N. How expert are the experts? An exploration of the concept of 'expert' within Delphi panel techniques. *Nurs Res* 2006;14:59-70. doi: 10.7748/nr2006.10.14.1.59.c6010.
69. Sinha IP, Smyth RL, Williamson PR. Using the Delphi technique to determine which outcomes to measure in clinical trials: recommendations for the future based on a systematic review of existing studies. *PLoS Med* 2011;8:e1000393. doi: 10.1371/journal.pmed.1000393.

For peer review only - http://bmjopen.bmj.com/site/about/guidelines.xhtml

70. Iglesias CP, Thompson A, Rogowski WH, Payne K. Reporting Guidelines for the Use of Expert Judgement in Model-Based Economic Evaluations. *Pharmacoeconomics* 2016;34:1161-72. doi: 10.1007/s40273-016-0425-9.
71. Toma C, Picioreanu I. The Delphi Technique: Methodological Considerations and the Need for Reporting Guidelines in Medical Journals. *Int J Public Health Res* 2016;4(6). Available at: http://www.openscienceonline.com/journal/archive2?journalId=718&paperId=3586. (accessed 15 August 2022).
72. Spranger J, Homberg A, Sonnberger M, Niederberger M. Reporting guidelines for Delphi techniques in health sciences: A methodological review. Berichterstattungsleitlinien für Delphi-Verfahren in den Gesundheitswissenschaften: Ein methodologisches Review. *Z Evid Fortbild Qual Gesundh wesen (ZEFQ)* Available online since June 16th 2022. doi: https://doi.org/10.1016/j.zefq.2022.04.025.
73. National Institute for Health and Care Excellence. NICE Guideline [NG197]: Shared decision making. Available at: https://www.nice.org.uk/guidance/ng197.
74. Dunn AG, Coiera E, Mandl KD, et al. Conflict of interest disclosure in biomedical research: A review of current practices, biases, and the role of public registries in improving transparency. *Res Integr Peer Rev* 2016;1:1. doi: 10.1186/s41073-016-0006-7.

Figure titles

Figure 1. PRISMA 2020 flow diagram for new systematic reviews, including searches of databases, registers and other sources

For peer review only

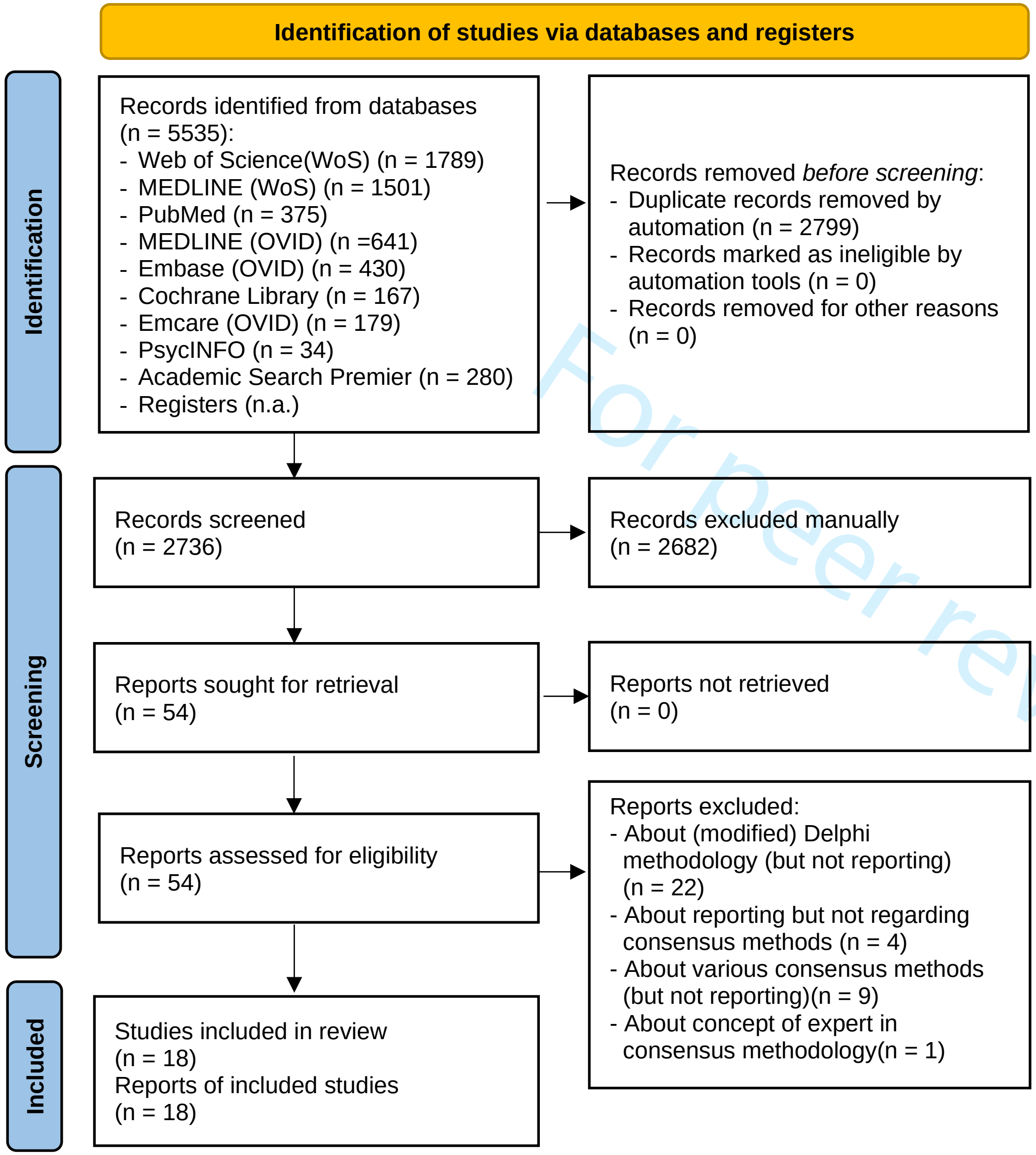

For peer review only - http://bmjopen.bmj.com/site/about/guidelines.xhtml

ACCORD - January 7th, 2022

Regular references:

Total: 2599 references, sourced from:

- Web of Science - core collection: 1775
- MEDLINE (Web of Science): 1501 - 202 unique
- PubMed: 375 - 219 unique
- MEDLINE (OVID): 641 - 174 unique
- Embase (OVID): 331 - 66 unique
- Cochrane Library: 131 - 77 unique
- Emcare (OVID): 179 - 29 unique
- Academic Search Premier: 280 - 23 unique
- PsycINFO: 173 - 34 unique

Meeting abstract references:

Total: 137 references, sourced from:

- Web of Science: 14
- Embase (OVID): 99 - 90 unique
- Cochrane Library: 36 - 33 unique

Known references:

- PubMed: 27841062 26796090 25587865 26395179 24581294
- MEDLINE (Web of Science): PMID=(27841062 OR 26796090 OR 25587865 OR 26395179 OR 24581294)
- Web of Science Core Collection: UT=(000393885800003 OR 000375153500022 OR 000376181900007 OR 000361506400001 OR 000334256400007 OR 000309802600012 OR 000321232400002 OR 000309802600012 OR 000465105500070)

Databases:

Web of Science Core Collection and MEDLINE (Web of Science)
http://isiknowledge.com/wos

((TI=("Delphi Technique" OR "Delphi Technique" OR "Delphi techniques" OR "Delphi method" OR "Delphi methods" OR "Delphi study" OR "Delphi studies" OR "Delphi survey" OR "Delphi surveys" OR "Delphi consensus" OR "Delphi based consensus" OR "Delphi questionnaire" OR "Delphi questionnaires" OR "Delphi research" OR "Delphi review" OR "Delphi reviews" OR "Delphi process" OR "Delphi processes" OR "Delphi based" OR "Delphi procedure" OR "Delphi procedures" OR "Delphi assessment" OR "Delphi assessments" OR "Delphi approach" OR "Delphi approaches" OR "Delphi panel" OR "Delphi panels" OR "Delphi round" OR "Delphi rounds" OR "Delphi analysis" OR

For peer review only

"Delphi expert" OR "Delphi experts" OR "Delphi consultation" OR "Delphi methodology" OR "nominal group technique" OR "nominal group techniques" OR "nominal group" OR "nominal groups" OR "nominal grouping" OR "consensus recommendation" OR "consensus recommendations" OR "consensus development" OR "consensus activity" OR "consensus activities" OR "Consensus Development Conference" OR "Consensus Development" OR "Consensus methodology" OR "consensus method*" OR "RAND" OR (("Guidelines" OR "guideline") NEAR/2 ("consensus" OR "delphi"))) OR AB=("Delphi Technique" OR "Delphi Technique" OR "Delphi techniques" OR "Delphi method" OR "Delphi methods" OR "Delphi study" OR "Delphi studies" OR "Delphi survey" OR "Delphi surveys" OR "Delphi consensus" OR "Delphi based consensus" OR "Delphi questionnaire" OR "Delphi questionnaires" OR "Delphi research" OR "Delphi review" OR "Delphi reviews" OR "Delphi process" OR "Delphi processes" OR "Delphi based" OR "Delphi procedure" OR "Delphi procedures" OR "Delphi assessment" OR "Delphi assessments" OR "Delphi approach" OR "Delphi approaches" OR "Delphi panel" OR "Delphi panels" OR "Delphi round" OR "Delphi rounds" OR "Delphi analysis" OR "Delphi expert" OR "Delphi experts" OR "Delphi consultation" OR "Delphi methodology" OR "nominal group technique" OR "nominal group techniques" OR "nominal group" OR "nominal groups" OR "nominal grouping" OR "consensus recommendation" OR "consensus recommendations" OR "consensus development" OR "consensus activity" OR "consensus activities" OR "Consensus Development Conference" OR "Consensus Development" OR "Consensus methodology" OR "consensus method*" OR "RAND") OR AK=("Delphi Technique" OR "Delphi Technique" OR "Delphi techniques" OR "Delphi method" OR "Delphi methods" OR "Delphi study" OR "Delphi studies" OR "Delphi survey" OR "Delphi surveys" OR "Delphi consensus" OR "Delphi based consensus" OR "Delphi questionnaire" OR "Delphi questionnaires" OR "Delphi research" OR "Delphi review" OR "Delphi reviews" OR "Delphi process" OR "Delphi processes" OR "Delphi based" OR "Delphi procedure" OR "Delphi procedures" OR "Delphi assessment" OR "Delphi assessments" OR "Delphi approach" OR "Delphi approaches" OR "Delphi panel" OR "Delphi panels" OR "Delphi round" OR "Delphi rounds" OR "Delphi analysis" OR "Delphi expert" OR "Delphi experts" OR "Delphi consultation" OR "Delphi methodology" OR "nominal group technique" OR "nominal group techniques" OR "nominal group" OR "nominal groups" OR "nominal grouping" OR "consensus recommendation" OR "consensus recommendations" OR "consensus development" OR "consensus activity" OR "consensus activities" OR "Consensus Development Conference" OR "Consensus Development" OR "Consensus methodology" OR "consensus method*" OR "RAND" OR (("Guidelines" OR "guideline") NEAR/2 ("consensus" OR "delphi")))) AND (TI=("quality of reporting" OR "reporting quality" OR "reporting qualities" OR "selective reporting" OR "poor reporting" OR "poor reported" OR "reporting guideline" OR "reporting" OR ("reporting" AND ("quality" OR "selective" OR "poor" OR "weak" OR "manner" OR "rigor" OR "improv*")) OR "Data Accuracy" OR "quality assessment" OR "strengths" OR "strength" OR "weaknesses" OR "weakness" OR "research method" OR "research methods" OR "research method*") OR AK=("quality of reporting" OR "reporting quality" OR "reporting qualities" OR "selective reporting" OR "poor reporting" OR "poor reported" OR ("reporting" NEAR/5 ("quality" OR "selective" OR "poor" OR "weak" OR "manner" OR "rigor" OR "improv*")) OR "Data Accuracy" OR

"Research Report standards" OR "quality assessment" OR "strengths" OR "strength" OR "weaknesses" OR "weakness" OR "research method" OR "research methods" OR "research method*") OR AB=("quality of reporting" OR "reporting quality" OR "reporting qualities" OR "selective reporting" OR "poor reporting" OR "poor reported" OR ("reporting" NEAR/5 ("quality" OR "selective" OR "poor" OR "weak" OR "manner" OR "rigor" OR "improv*")) OR "Data Accuracy" OR "Research Report standards" OR "quality assessment" OR "strengths" OR "strength" OR "weaknesses" OR "weakness")))

PubMed
http://www.ncbi.nlm.nih.gov/pubmed?otool=leiden

(("Delphi Technique"[majr] OR "Delphi Technique"[ti] OR "Delphi techniques"[ti] OR "Delphi method"[ti] OR "Delphi methods"[ti] OR "Delphi study"[ti] OR "Delphi studies"[ti] OR "Delphi survey"[ti] OR "Delphi surveys"[ti] OR "Delphi consensus"[ti] OR "Delphi based consensus"[ti] OR "Delphi questionnaire"[ti] OR "Delphi questionnaires"[ti] OR "Delphi research"[ti] OR "Delphi review"[ti] OR "Delphi reviews"[ti] OR "Delphi process"[ti] OR "Delphi processes"[ti] OR "Delphi based"[ti] OR "Delphi procedure"[ti] OR "Delphi procedures"[ti] OR "Delphi assessment"[ti] OR "Delphi assessments"[ti] OR "Delphi approach"[ti] OR "Delphi approaches"[ti] OR "Delphi panel"[ti] OR "Delphi panels"[ti] OR "Delphi round"[ti] OR "Delphi rounds"[ti] OR "Delphi analysis"[ti] OR "Delphi expert"[ti] OR "Delphi experts"[ti] OR "Delphi consultation"[ti] OR "Delphi methodology"[ti] OR "nominal group technique"[ti] OR "nominal group techniques"[ti] OR "nominal group"[ti] OR "nominal groups"[ti] OR "nominal grouping"[ti] OR "consensus recommendation"[ti] OR "consensus recommendations"[ti] OR "consensus development"[ti] OR "consensus activity"[ti] OR "consensus activities"[ti] OR "consensus methodology"[ti] OR "consensus method*"[ti] OR "Consensus Development Conferences as Topic"[majr] OR "RAND"[ti] OR ("Guidelines as Topic"[majr:noexp] AND ("consensus"[tw] OR "delphi"[tw]))) AND ("reporting"[ti] OR "quality of reporting"[tw] OR "reporting quality"[tw] OR "reporting qualities"[tw] OR "selective reporting"[tw] OR "poor reporting"[tw] OR "poor reported"[tw] OR "poorly reported"[tw] OR "Research Report/standards"[majr] OR "Research Design/standards"[mesh] OR "Research Design"[majr:noexp] OR "Writing/standards"[mesh] OR "Writing"[majr] OR "research method"[ti] OR "research methods"[ti] OR "research method*"[ti]))

MEDLINE via OVID
http://gateway.ovid.com/ovidweb.cgi?T=JS&MODE=ovid&NEWS=n&PAGE=main&D=medall

((exp *"Delphi Technique"/ OR "Delphi Technique".ti OR "Delphi techniques".ti OR "Delphi method".ti OR "Delphi methods".ti OR "Delphi study".ti OR "Delphi studies".ti OR "Delphi survey".ti OR "Delphi surveys".ti OR "Delphi consensus".ti OR "Delphi based consensus".ti OR "Delphi questionnaire".ti OR "Delphi questionnaires".ti OR "Delphi research".ti OR "Delphi review".ti OR "Delphi reviews".ti OR "Delphi

For peer review only

process".ti OR "Delphi processes".ti OR "Delphi based".ti OR "Delphi procedure".ti OR "Delphi procedures".ti OR "Delphi assessment".ti OR "Delphi assessments".ti OR "Delphi approach".ti OR "Delphi approaches".ti OR "Delphi panel".ti OR "Delphi panels".ti OR "Delphi round".ti OR "Delphi rounds".ti OR "Delphi analysis".ti OR "Delphi expert".ti OR "Delphi experts".ti OR "Delphi consultation".ti OR "Delphi methodology".ti OR "nominal group technique".ti OR "nominal group techniques".ti OR "nominal group".ti OR "nominal groups".ti OR "nominal grouping".ti OR "consensus recommendation".ti OR "consensus recommendations".ti OR "consensus development".ti OR "consensus activity".ti OR "consensus activities".ti OR "consensus methodology".ti OR "consensus method*".ti OR exp *"Consensus Development Conferences as Topic"/ OR "RAND".ti OR ("Guidelines as Topic"/ AND ("consensus".mp OR "delphi".mp)) OR (("Guidelines".mp OR "guideline".mp) ADJ2 ("consensus".mp OR "delphi".mp))) AND ("reporting".ti OR "quality of reporting".mp OR "reporting quality".mp OR "reporting qualities".mp OR "selective reporting".mp OR "poor reporting".mp OR "poor reported".mp OR "poorly reported".mp OR "Research Report/standards"/ OR exp "Research Design"/st OR *"Research Design"/ OR exp "Writing"/st OR exp *"Writing"/ OR "research method".ti OR "research methods".ti OR "research method*".ti OR ("reporting" ADJ8 ("quality" OR "selective" OR "poor" OR "weak" OR "manner" OR "rigor" OR "improv*")).mp))

Embase
http://ovidsp.ovid.com/ovidweb.cgi?T=JS&PAGE=main&MODE=ovid&D=oemezd

((exp *"Delphi Study"/ OR "Delphi Technique".ti OR "Delphi techniques".ti OR "Delphi method".ti OR "Delphi methods".ti OR "Delphi study".ti OR "Delphi studies".ti OR "Delphi survey".ti OR "Delphi surveys".ti OR "Delphi consensus".ti OR "Delphi based consensus".ti OR "Delphi questionnaire".ti OR "Delphi questionnaires".ti OR "Delphi research".ti OR "Delphi review".ti OR "Delphi reviews".ti OR "Delphi process".ti OR "Delphi processes".ti OR "Delphi based".ti OR "Delphi procedure".ti OR "Delphi procedures".ti OR "Delphi assessment".ti OR "Delphi assessments".ti OR "Delphi approach".ti OR "Delphi approaches".ti OR "Delphi panel".ti OR "Delphi panels".ti OR "Delphi round".ti OR "Delphi rounds".ti OR "Delphi analysis".ti OR "Delphi expert".ti OR "Delphi experts".ti OR "Delphi consultation".ti OR "Delphi methodology".ti OR "nominal group technique".ti OR "nominal group techniques".ti OR "nominal group".ti OR "nominal groups".ti OR "nominal grouping".ti OR "consensus recommendation".ti OR "consensus recommendations".ti OR "consensus development".ti OR "consensus activity".ti OR "consensus activities".ti OR "consensus methodology".ti OR "consensus method*".ti OR exp *"Consensus Development"/ OR "RAND".ti OR (("Guidelines".ti,ab OR "guideline".ti,ab) ADJ2 ("consensus".ti,ab OR "delphi".ti,ab))) AND ("reporting".ti OR "quality of reporting".ti,ab OR "reporting quality".ti,ab OR "reporting qualities".ti,ab OR "selective reporting".ti,ab OR "poor reporting".ti,ab OR "poor reported".ti,ab OR "poorly reported".ti,ab OR *"Methodology"/ OR *"Writing"/ OR "research method".ti OR "research methods".ti OR "research method*".ti OR ("reporting" ADJ8 ("quality" OR "selective" OR "poor" OR "weak" OR "manner" OR "rigor" OR "improv*")).ti,ab))

Cochrane
https://www.cochranelibrary.com/advanced-search/search-manager

(("Delphi Technique" OR "Delphi Technique" OR "Delphi techniques" OR "Delphi method" OR "Delphi methods" OR "Delphi study" OR "Delphi studies" OR "Delphi survey" OR "Delphi surveys" OR "Delphi consensus" OR "Delphi based consensus" OR "Delphi questionnaire" OR "Delphi questionnaires" OR "Delphi research" OR "Delphi review" OR "Delphi reviews" OR "Delphi process" OR "Delphi processes" OR "Delphi based" OR "Delphi procedure" OR "Delphi procedures" OR "Delphi assessment" OR "Delphi assessments" OR "Delphi approach" OR "Delphi approaches" OR "Delphi panel" OR "Delphi panels" OR "Delphi round" OR "Delphi rounds" OR "Delphi analysis" OR "Delphi expert" OR "Delphi experts" OR "Delphi consultation" OR "Delphi methodology" OR "nominal group technique" OR "nominal group techniques" OR "nominal group" OR "nominal groups" OR "nominal grouping" OR "consensus recommendation" OR "consensus recommendations" OR "consensus development" OR "consensus activity" OR "consensus activities" OR "Consensus Development Conference" OR "Consensus Development" OR "Consensus methodology" OR "consensus method*" OR "RAND" OR (("Guidelines" OR "guideline") NEAR/2 ("consensus" OR "delphi"))) AND ("quality of reporting" OR "reporting quality" OR "reporting qualities" OR "selective reporting" OR "poor reporting" OR "poor reported" OR ("reporting" NEAR/5 ("quality" OR "selective" OR "poor" OR "weak" OR "manner" OR "rigor" OR "improv*")) OR "Data Accuracy" OR "Research Report standards" OR "quality assessment" OR "strengths" OR "strength" OR "weaknesses" OR "weakness" OR "research method" OR "research methods" OR "research method*")):ti,ab,kw

Emcare
http://ovidsp.ovid.com/ovidweb.cgi?T=JS&NEWS=n&CSC=Y&PAGE=main&D=emcr

((exp *"Delphi Study"/ OR "Delphi Technique".ti OR "Delphi techniques".ti OR "Delphi method".ti OR "Delphi methods".ti OR "Delphi study".ti OR "Delphi studies".ti OR "Delphi survey".ti OR "Delphi surveys".ti OR "Delphi consensus".ti OR "Delphi based consensus".ti OR "Delphi questionnaire".ti OR "Delphi questionnaires".ti OR "Delphi research".ti OR "Delphi review".ti OR "Delphi reviews".ti OR "Delphi process".ti OR "Delphi processes".ti OR "Delphi based".ti OR "Delphi procedure".ti OR "Delphi procedures".ti OR "Delphi assessment".ti OR "Delphi assessments".ti OR "Delphi approach".ti OR "Delphi approaches".ti OR "Delphi panel".ti OR "Delphi panels".ti OR "Delphi round".ti OR "Delphi rounds".ti OR "Delphi analysis".ti OR "Delphi expert".ti OR "Delphi experts".ti OR "Delphi consultation".ti OR "Delphi methodology".ti OR "nominal group technique".ti OR "nominal group techniques".ti OR "nominal group".ti OR "nominal groups".ti OR "nominal grouping".ti OR "consensus recommendation".ti OR "consensus recommendations".ti OR "consensus development".ti OR "consensus activity".ti OR "consensus activities".ti OR "consensus methodology".ti OR "consensus method*".ti OR exp *"Consensus Development"/ OR "RAND".ti OR (("Guidelines".ti,ab OR "guideline".ti,ab) ADJ2 ("consensus".ti,ab OR "delphi".ti,ab))) AND ("reporting".ti

For peer review only - http://bmjopen.bmj.com/site/about/guidelines.xhtml

OR "quality of reporting".ti,ab OR "reporting quality".ti,ab OR "reporting qualities".ti,ab OR "selective reporting".ti,ab OR "poor reporting".ti,ab OR "poor reported".ti,ab OR "poorly reported".ti,ab OR *"Methodology"/ OR *"Writing"/ OR "research method".ti OR "research methods".ti OR "research method*".ti OR ("reporting" ADJ8 ("quality" OR "selective" OR "poor" OR "weak" OR "manner" OR "rigor" OR "improv*")).ti,ab))

Academic Search Premier
http://search.ebscohost.com/login.aspx?authtype=ip,uid&profile=lumc&defaultdb=aph

((TI("Delphi Technique" OR "Delphi Technique" OR "Delphi techniques" OR "Delphi method" OR "Delphi methods" OR "Delphi study" OR "Delphi studies" OR "Delphi survey" OR "Delphi surveys" OR "Delphi consensus" OR "Delphi based consensus" OR "Delphi questionnaire" OR "Delphi questionnaires" OR "Delphi research" OR "Delphi review" OR "Delphi reviews" OR "Delphi process" OR "Delphi processes" OR "Delphi based" OR "Delphi procedure" OR "Delphi procedures" OR "Delphi assessment" OR "Delphi assessments" OR "Delphi approach" OR "Delphi approaches" OR "Delphi panel" OR "Delphi panels" OR "Delphi round" OR "Delphi rounds" OR "Delphi analysis" OR "Delphi expert" OR "Delphi experts" OR "Delphi consultation" OR "Delphi methodology" OR "nominal group technique" OR "nominal group techniques" OR "nominal group" OR "nominal groups" OR "nominal grouping" OR "consensus recommendation" OR "consensus recommendations" OR "consensus development" OR "consensus activity" OR "consensus activities" OR "Consensus Development Conference" OR "Consensus Development" OR "Consensus methodology" OR "consensus method*" OR "RAND" OR (("Guidelines" OR "guideline") N2 ("consensus" OR "delphi"))) OR KW("Delphi Technique" OR "Delphi Technique" OR "Delphi techniques" OR "Delphi method" OR "Delphi methods" OR "Delphi study" OR "Delphi studies" OR "Delphi survey" OR "Delphi surveys" OR "Delphi consensus" OR "Delphi based consensus" OR "Delphi questionnaire" OR "Delphi questionnaires" OR "Delphi research" OR "Delphi review" OR "Delphi reviews" OR "Delphi process" OR "Delphi processes" OR "Delphi based" OR "Delphi procedure" OR "Delphi procedures" OR "Delphi assessment" OR "Delphi assessments" OR "Delphi approach" OR "Delphi approaches" OR "Delphi panel" OR "Delphi panels" OR "Delphi round" OR "Delphi rounds" OR "Delphi analysis" OR "Delphi expert" OR "Delphi experts" OR "Delphi consultation" OR "Delphi methodology" OR "nominal group technique" OR "nominal group techniques" OR "nominal group" OR "nominal groups" OR "nominal grouping" OR "consensus recommendation" OR "consensus recommendations" OR "consensus development" OR "consensus activity" OR "consensus activities" OR "Consensus Development Conference" OR "Consensus Development" OR "Consensus methodology" OR "consensus method*" OR "RAND" OR (("Guidelines" OR "guideline") N2 ("consensus" OR "delphi")))) AND (TI("quality of reporting" OR "reporting quality" OR "reporting qualities" OR "selective reporting" OR "poor reporting" OR "poor reported" OR "reporting guideline" OR "reporting" OR ("reporting" AND ("quality" OR "selective" OR "poor" OR "weak" OR "manner" OR "rigor" OR "improv*")) OR "Data Accuracy" OR "quality assessment" OR "strengths" OR "strength" OR "weaknesses" OR "weakness" OR "research method" OR "research methods" OR "research method*") OR

For peer review only

KW("quality of reporting" OR "reporting quality" OR "reporting qualities" OR "selective reporting" OR "poor reporting" OR "poor reported" OR ("reporting" N5 ("quality" OR "selective" OR "poor" OR "weak" OR "manner" OR "rigor" OR "improv*")) OR "Data Accuracy" OR "Research Report standards" OR "quality assessment" OR "strengths" OR "strength" OR "weaknesses" OR "weakness" OR "research method" OR "research methods" OR "research method*") OR AB("quality of reporting" OR "reporting quality" OR "reporting qualities" OR "selective reporting" OR "poor reporting" OR "poor reported" OR ("reporting" N5 ("quality" OR "selective" OR "poor" OR "weak" OR "manner" OR "rigor" OR "improv*")) OR "Data Accuracy" OR "Research Report standards" OR "quality assessment" OR "strengths" OR "strength" OR "weaknesses" OR "weakness")))

PsycINFO
http://search.ebscohost.com/login.aspx?authtype=ip,uid&profile=lumc&defaultdb=psyh

((TI("Delphi Technique" OR "Delphi Technique" OR "Delphi techniques" OR "Delphi method" OR "Delphi methods" OR "Delphi study" OR "Delphi studies" OR "Delphi survey" OR "Delphi surveys" OR "Delphi consensus" OR "Delphi based consensus" OR "Delphi questionnaire" OR "Delphi questionnaires" OR "Delphi research" OR "Delphi review" OR "Delphi reviews" OR "Delphi process" OR "Delphi processes" OR "Delphi based" OR "Delphi procedure" OR "Delphi procedures" OR "Delphi assessment" OR "Delphi assessments" OR "Delphi approach" OR "Delphi approaches" OR "Delphi panel" OR "Delphi panels" OR "Delphi round" OR "Delphi rounds" OR "Delphi analysis" OR "Delphi expert" OR "Delphi experts" OR "Delphi consultation" OR "Delphi methodology" OR "nominal group technique" OR "nominal group techniques" OR "nominal group" OR "nominal groups" OR "nominal grouping" OR "consensus recommendation" OR "consensus recommendations" OR "consensus development" OR "consensus activity" OR "consensus activities" OR "Consensus Development Conference" OR "Consensus Development" OR "Consensus methodology" OR "consensus method*" OR "RAND" OR (("Guidelines" OR "guideline") N2 ("consensus" OR "delphi"))) OR AB("Delphi Technique" OR "Delphi Technique" OR "Delphi techniques" OR "Delphi method" OR "Delphi methods" OR "Delphi study" OR "Delphi studies" OR "Delphi survey" OR "Delphi surveys" OR "Delphi consensus" OR "Delphi based consensus" OR "Delphi questionnaire" OR "Delphi questionnaires" OR "Delphi research" OR "Delphi review" OR "Delphi reviews" OR "Delphi process" OR "Delphi processes" OR "Delphi based" OR "Delphi procedure" OR "Delphi procedures" OR "Delphi assessment" OR "Delphi assessments" OR "Delphi approach" OR "Delphi approaches" OR "Delphi panel" OR "Delphi panels" OR "Delphi round" OR "Delphi rounds" OR "Delphi analysis" OR "Delphi expert" OR "Delphi experts" OR "Delphi consultation" OR "Delphi methodology" OR "nominal group technique" OR "nominal group techniques" OR "nominal group" OR "nominal groups" OR "nominal grouping" OR "consensus recommendation" OR "consensus recommendations" OR "consensus development" OR "consensus activity" OR "consensus activities" OR "Consensus Development Conference" OR "Consensus Development" OR "Consensus methodology" OR "consensus method*" OR "RAND") OR KW("Delphi Technique" OR "Delphi

For peer review only

Technique" OR "Delphi techniques" OR "Delphi method" OR "Delphi methods" OR "Delphi study" OR "Delphi studies" OR "Delphi survey" OR "Delphi surveys" OR "Delphi consensus" OR "Delphi based consensus" OR "Delphi questionnaire" OR "Delphi questionnaires" OR "Delphi research" OR "Delphi review" OR "Delphi reviews" OR "Delphi process" OR "Delphi processes" OR "Delphi based" OR "Delphi procedure" OR "Delphi procedures" OR "Delphi assessment" OR "Delphi assessments" OR "Delphi approach" OR "Delphi approaches" OR "Delphi panel" OR "Delphi panels" OR "Delphi round" OR "Delphi rounds" OR "Delphi analysis" OR "Delphi expert" OR "Delphi experts" OR "Delphi consultation" OR "Delphi methodology" OR "nominal group technique" OR "nominal group techniques" OR "nominal group" OR "nominal groups" OR "nominal grouping" OR "consensus recommendation" OR "consensus recommendations" OR "consensus development" OR "consensus activity" OR "consensus activities" OR "Consensus Development Conference" OR "Consensus Development" OR "Consensus methodology" OR "consensus method*" OR "RAND" OR (("Guidelines" OR "guideline") N2 ("consensus" OR "delphi")))) AND (TI("quality of reporting" OR "reporting quality" OR "reporting qualities" OR "selective reporting" OR "poor reporting" OR "poor reported" OR "reporting guideline" OR "reporting" OR ("reporting" AND ("quality" OR "selective" OR "poor" OR "weak" OR "manner" OR "rigor" OR "improv*")) OR "Data Accuracy" OR "quality assessment" OR "strengths" OR "strength" OR "weaknesses" OR "weakness" OR "research method" OR "research methods" OR "research method*") OR KW("quality of reporting" OR "reporting quality" OR "reporting qualities" OR "selective reporting" OR "poor reporting" OR "poor reported" OR ("reporting" N5 ("quality" OR "selective" OR "poor" OR "weak" OR "manner" OR "rigor" OR "improv*")) OR "Data Accuracy" OR "Research Report standards" OR "quality assessment" OR "strengths" OR "strength" OR "weaknesses" OR "weakness" OR "research method" OR "research methods" OR "research method*") OR AB("quality of reporting" OR "reporting quality" OR "reporting qualities" OR "selective reporting" OR "poor reporting" OR "poor reported" OR ("reporting" N5 ("quality" OR "selective" OR "poor" OR "weak" OR "manner" OR "rigor" OR "improv*")) OR "Data Accuracy" OR "Research Report standards" OR "quality assessment" OR "strengths" OR "strength" OR "weaknesses" OR "weakness")))

For peer review only

**Author, year**	
**Assessor**	

**Background**<br>1.1 Does the study suggest anything about how or if consensus papers should report the context or rationale for choosing a consensus method over other methods?	
**Background**<br>1.2 Does the study suggest anything about how/what or if consensus papers should report the objectives of the consensus exercise?	

**Methods**<br>2.1 Does the study suggest anything about how/what or if consensus papers should report regarding:<br>A literature search/strategy?	
**Methods**<br>2.2 Does the study the suggest anything about how/what or if consensus papers should report regarding:<br>Inclusion and exclusion criteria for the literature search?	
**Methods**<br>2.3 Does the study suggest anything of what or if consensus report should report on panel composition, n of participants, expertise, origin? Prespecified?	
**Methods**<br>2.4 Does the study suggest anything of how or if PPI (public patient involvement) activity should be reported?	
**Methods**<br>2.5 Does the study suggest anything about what or if consensus papers should report regarding panel recruitment strategies, invitations? Any level of detail specified?	
**Methods**<br>2.6 Does the study suggest how or if consensus papers should report the consensus criteria/threshold (or the level of agreement considered to reach consensus)?	
**Methods**<br>2.7 Does the study suggest how or if consensus papers should report how decision of approval of an item will be made?	
**Methods**	

2.8 Does the study suggest anything about what level of detail should be reported regarding the number of Delphi rounds or if this should be reported?	
**Methods**<br>2.9 Does the study suggest anything about what level of detail should be reported regarding the criteria used for defining the number of rounds? (why 2-3 or more e.g.) or if this should be reported?	
**Methods**<br>2.10 Does the study suggest anything about the details that should be reported regarding the time between rounds, if it should be prespecified or if this should be reported?	
**Methods**<br>2.11 Does the study suggest anything about details that should be reported of the names of the techniques of non-Delphi methods used to gather participants' inputs and reach consensus?	
**Methods**<br>2.12 Does the study suggest anything of what or in which detail should be reported regarding tool or electronic system used for Delphi? (If Delphi was used)? Or if this should be reported?	
**Methods**<br>2.13 Does the study suggest anything about how or in what level of detail the anonymity of participants (in Delphi or other methods) has to be reported? Or if this should be reported?	
**Methods**<br>2.14 Does the study suggest anything about how to report, and in what level of detail, the feedback for panellists (in Delphi rounds or other methods) process? Or if this should be reported?	
**Methods**<br>2.15 Does the study suggest anything about how or if data synthesis/analysis should be reported (from any consensus method used and how this was calculated statistically) and in what level of detail?	
**Methods**<br>2.16 Does the study suggest anything about how or if piloting should be reported and in what level of detail (e.g. understanding of consensus items, platforms used, tools used)?	
**Methods**	

2.17 Does the study suggest anything about how or if the role of Steering Committee members should be reported?	
**Methods**<br>2.18 Does the study suggest anything on what or if should be described regarding COI or funding?	
**Methods**<br>2.19 Does the study suggest anything on what should be described of how is dealt with COI of panellist (not allowed to vote when there is COI)? Or if this should be described	

**Results**<br>3.1 Does the study suggest anything on how to report the initial evidence search (presentation of results of the literature review)?	
**Results**<br>3.2 Does the study suggest anything on how to report n of studies found?	
**Results**<br>3.3 Does the study recommend which detail should be used when reporting panellists drop-outs (numbers and reasons)? Or if this should be reported?	
**Results**<br>3.4 Does the study suggest how or if approval rates per item shared with respondents for each round should be reported in the Results section?	
**Results**<br>3.5 Does the study suggest anything about in which detail the items that have been dropped should be reported? (reasons e.g.) Or if this should be reported?	
**Results**<br>3.6 Does the study make any recommendation on how to report the collection, synthesis and use of comments from panellists? Or if this should be reported?	
**Results**<br>3.7 Does the study suggest regarding how the final list of items (for clinical guideline or reporting guideline) should be reported? Or if this should be reported?	

**Discussion**<br>4.1 Does the paper suggest anything about reporting the limitations and strengths of the study and how? Or if this should be reported?	
**Discussion**	

For peer review only

4.2 Does the paper suggest anything about what or in which detail the applicability generalisability, and reproducibility of the study should be reported? Or if this should be reported?	

**5.1 Any other item proposed by the paper that is not captured in other columns?**	
**5.2 Any other item not proposed by the paper, but you think that could be added (not fitting the categories above)?**	

**Examples of text with well reported methods/results (for E&E document) - write NA if none was cited or found by you**	
**Additional comments from assessor**	

For peer review only

Data on reporting quality (*recommendations in italics)*

Study	What is stated regarding reporting quality?
Banno 2019[32]	• "The reporting quality of the Delphi technique in reporting guidelines is unknown even though the use of the Delphi technique was recommended in the guidance for reporting guidelines." (Note: This is a protocol for the systematic review of 2020.)<br>*4 quality score items are summarised of Delphi methods used in reporting guidelines*.
Banno 2020[16]	• "Reproducible criteria of participants, number of rounds, criteria for dropping items, and stopping criteria other than rounds were found for 87%, 97%, 69%, and 13%, respectively of reporting guidelines developed with the Delphi method. The total score of reporting quality was 2 or more in 94% of reporting guidelines using the Delphi method."<br>*4 quality score items are summarised of Delphi methods used in reporting guidelines*.
Boulkedid 2011[17]	• "Study reports did not consistently provide details that are important for interpreting the results. For example, only 39% of studies reported that individual feedback was given between rounds and the method used to define a consensus was specified in only 77% studies. Moreover, response rates for all rounds were reported in only 31% of studies. Information on both points is needed to evaluate the validity and credibility of the results. If the Delphi method is incompletely described this may affect the overall quality of the final consensus and the selected indicators are unlikely to gain the level of credibility needed for adoption I clinical practice."<br>• "The Delphi procedure is valuable for achieving a consensus about issues where none existed previously. However, our findings indicate a need for improving the use and reporting of this technique."<br>*Table 5 provides recommendations for reporting the Delphi procedure.*
Chan 2019[20]	• "This lack of clear definition has led to considerable confusion and substantial variation in the quality of reporting of Delphi studies"<br>• "One-third of medical education Delphi studies failed to report that a literature review on the topic of interest had been conducted , and over half failed to report key aspects such as what background information was provided to participants; the response rate for each round; what formal feedback of group rating was shared between rounds; a statement that anonymity was maintained; and a clear definition of consensus."<br>• "Lack of clarity in the report in the reporting of procedures and methodological choices associated with the modified Delphi studies can prevent readers from effectively appraising and interpreting findings."<br>• "Methodological rigor and transparent reporting are essential to assure readers that the consensus results are applicable to their environment, and to translate expert opinion into practice."<br>*Box 1 provides recommendations to improve reporting.*
Diamond 2014[18]	• "Definitions of consensus vary widely and are poorly reported. Improved criteria for reporting of methods of Delphi studies are required."<br>• "Methodologic criteria are proposed for the reporting of Delphi studies."<br>• "Despite the fact that the most Delphi studies in our cohort had consensus as their aim, in only a minority of the Delphi studies reviewed was consensus defined with a specific criterion. Furthermore, this criterion was the reason for termination of the Delphi process, usually on the basis of an *a priori* definition."<br>• "We believe that there is a need to improve the reporting of Delphi studies, along the lines of a CONSORT-like guideline, as is used for randomized controlled trials."<br>*Methodologic criteria are proposed for the reporting of Delphi studies.*

For peer review only - http://bmjopen.bmj.com/site/about/guidelines.xhtml

Gattrell 2019[29]	"At present there are a lack of standard, validated reporting guidelines for publications reporting the results of Delphi panel studies."<br><br>Quality assessment: Methodological quality<br>• The type of Delphi technique used, or the modifications to the method, was not outlined in all publications (included in 62/90 publications; 68.9%).<br>• Just over half of all publications stated that there was some diversity amongst participants and clearly outlined the methods for the selection of panellists.<br>• Agreement and consensus thresholds should be defined prior to study commencement, but in 40% of publications it was unclear, or not stated whether these thresholds were predefined.<br>• Anonymised responses are typically conveyed back to the group after each round, but this was clearly reported in less than half (38.9%) of publications.<br><br>Quality assessment: Reporting quality and transparency (Figure 3b).<br>• The funding source was not clearly disclosed in over a third of publications, and almost twice as many publications did not clearly disclose the funder's role.<br>• Conflicts of interest were clearly described in most publications (included in 79/90 publications; 87.8%).<br>• Clear disclosure of external support was not evident in the majority of the publications.
Grant 2018[24]	• "Specifying the analysis procedure for consensus is therefore a critical consideration when designing consensus-oriented Delphi processes in health research."<br>• "Without prespecifying their analysis procedures in a study registry, health researchers conducting consensus-oriented Delphi processes can mine for and selectively report the most desirable set of items reaching consensus and even present the reported analysis as the only one conducted. Undisclosed flexibility in data collection, analysis, and reporting is a growing concern in empirical research."<br>• "Without preregistering and reporting all of the attempted analysis procedures and when they were attempted, the extent and impact of researchers trying different analysis procedures is nearly impossible for peer reviewers, editors, and consumers of Delphi research to assess."<br>• "To be completely registered, the preanalysis plan should precisely describe the essential elements of the analysis procedure for determining consensus (see Box 2)."<br>• "Researchers should use existing guidance on reporting completed Delphi processes to provide sufficient information for comparing the final article to the registered preanalysis plan [1,12,42], with particular attention in the final article to any changes from the preanalysis plan in the items, rating criteria, analytic procedure (measure and threshold), and data and participants included in the analysis."<br>*Box 2 provides a minimum set of items to include in prospectively registered preanalysis plans for consensus-oriented Delphi processes.*
Hasson 2017[27]	• "Figure 1 Areas for reporting on the Delphi survey technique."<br>• "In Delphi surveys there exists no consistent method for reporting findings (Schmidt 1997) and a review of the literature showed that a number of approaches have been used."<br>• "The following diagram attempts to outline those sections that researchers should report upon when using the Delphi. This will help readers to judge the reliability of the method and the results obtained."<br>*Followed by a checklist of issues, which could be used by researchers.*

For peer review only

Humphrey-Murto 2017[21]	• "The authors set out to describe the use of consensus methods in medical education research and to assess the reporting quality of these methods and results."<br>• "Improved criteria for reporting are needed."<br>• "Our findings suggest that the reporting quality and standardization of consensus methods in medical education research varies greatly. The following areas appeared particularly problematic and were often left out or poorly described in the articles we reviewed: conducting a literature review to inform the consensus method; providing background information to participants; reporting the number of participants after each round; describing the level of anonymity used in the study; providing participants with feedback of group ratings; and articulating the definition of consensus used in the study."<br>*Recommendations for improvements in these areas are provided in Discussion.*
Humphrey-Murto 2017[28]	• "Consensus group methods are widely used in research to identify and measure areas where incomplete evidence exists for decision-making. Despite their widespread use, these methods are often inconsistently used and reported."<br>• "This paper and associated Guide aim to describe these methods and to highlight common weaknesses in methodology and reporting."<br>• "The AMEE Guide describes these methods to provide a "how to" approach, highlight common weaknesses in methodology and reporting, and outline recommendations for reporting future consensus based studies."<br>• "Four recent reviews using the Delphi in health care and policy-related research have systematically explored deficiencies in the use and reporting of consensus group methods. Collectively, these studies have noted deficiencies regarding: information provided to the participants at the start of Delphi, reporting response rates, feedback to participants, level of anonymity, outcomes after each round and the definition of consensus."<br>*This guide provides recommendations for improvement of reporting.*
Humphrey-Murto 2019[25]	• "Studies using the Delphi for selecting performance indicators for healthcare, for medical and nursing education, or for determining outcomes to measure in clinical trials, often fail to adequately report sufficient methodological detail. Examples include poor reporting of background information provided to participants, response rates for all rounds, level of anonymity, formal feedback between rounds, and the definition of consensus."<br>*OMERACT Delphi consensus checklist is provided in Figure 1.*
Jünger 2017[12]	• "Substantial variation was found concerning the quality of the study conduct and the transparency of reporting of Delphi studies used for the development of best practice guidance in palliative care. Since credibility of the resulting recommendations depends on the rigorous use of the Delphi technique, there is a need for consistency and quality both in the conduct and reporting of studies. To allow a critical appraisal of the methodology and the resulting guidance, a reporting standard for Conducting and Reporting of DElphi Studies (CREDES) is proposed."<br>Study adds in Box 3*"Recommendations for the Conducting and REporting of DElphi Studies (CREDES)."*
Ng 2018[30]	• "Given the variance in the use of Delphi method, reporting guidelines could help improve reporting of this research, and thereby allow readers to be aware of the accuracy of data and conclusions."<br>• "We anticipate the implementation of this will promote transparent and accurate reporting of research using Delphi method for obtaining quantitative data."<br>*A set of reporting guidelines is proposed.*
Niederberger 2020[26]	• "Significant weaknesses exist in the quality of the reporting."

For peer review only

	• "Criteria for evaluating the quality of their execution and reporting also appear to be necessary."<br>• "A specific definition of the underlying Delphi technique was found in 61% (ID11) and 88.2% (ID4) of the Delphi articles investigated."<br>• "Most of the Delphi studies analyzed in the reviews reported on the number of participating experts. The rates for the initial round were between 84% (ID6) and 100% (ID12). Four of the reviews investigated whether the number of experts was stated for each round (ID4, ID7, ID11, ID12). In one review based on 10 Delphi studies from health sciences (ID7), the authors discovered that the number of experts per round was stated in all articles. A review of 48 studies in a medical context indicated that the number of invited experts was stated less frequently with each round (ID6). Seven of the 12 reviews investigated whether the backgrounds of the experts had been reported, what kind of expertise they possessed, and the criteria according to which they were selected (ID1, ID3, ID4, ID6, ID9, ID11, ID12). One review of Delphi techniques in a health context determined that the criteria for selecting the experts was reproduced in 65 of 100 articles (65%) (ID3) included in that particular review. In other reviews with a more specific focus, such as on health care, palliative medicine, or health promotion, the rates were higher at 69% (ID11), 70% (ID9) and 79% (ID1), respectively. Based on the results of the reviews, the criteria by which the experts were selected and approached was not always clear. In one review of 100 studies from the care sector, the proportion of articles with unclear selection criteria was 11.2% (ID4), while the proportion was 93.3% in a review of 15 studies from the clinical sector (ID12)."<br>• "Seven of the 12 reviews determined whether and when consensus was defined in the Delphi studies (ID1, ID3, ID4, ID6, ID9, ID11, ID12). The number of studies in which consensus was defined in the article was between 73.5% (ID3) and 83.3% (ID9) in the reviews."<br>• "The authors of seven reviews investigated whether the number of Delphi rounds was published (ID1, ID3, ID4, ID6, ID9, ID11, ID12). The number of Delphi rounds was stated in most of the Delphi studies (e.g., ID1 82.5%, ID4 91%, ID6 100%, ID9 49.3%, ID12 93.3%). Six of the reviews included a report of the generation of the questionnaire (ID1, ID4, ID6, ID9, ID11, ID12). They demonstrated that up to 96.3% of the investigated articles reported on how the items for the questionnaire were developed (ID1). In contrast, this rate stood at 33.3% in the review of palliative care articles (ID9). The authors of two reviews investigated the question of how the items were changed during the Delphi process based on the judgments submitted by the experts (ID3, ID12). In one of the reviews, the authors indicated that 59% of the analyzed articles had defined criteria for dropping items (ID3). In another review, the authors stated that all of the investigated Delphi studies included a report of "what was asked in each round" (ID12, p. 2). The authors of the reviews reported about the feedback in most of the Delphi studies (ID11 67.9%, ID12 93.3%). The information provided about the response rate per Delphi round was less (ID1 and ID4 39%). According to the results of the reviews, around half of the studies did not provide information about the feedback design between the Delphi rounds (ID1 40%, ID4 55.1%, ID6 37.7% ID12 40%). According to the authors of the review on health promotion, the process—from formulating the issue being investigated through to the development of the questionnaire—was in general similar to a "black box," and the methodological quality of the survey instrument was almost impossible to evaluate using the published information (ID11, p. 318)."<br>• "Our results also indicate deficits both in carrying out and also reporting Delphi techniques."

For peer review only - http://bmjopen.bmj.com/site/about/guidelines.xhtml

	• "The findings in the reviews we analyzed indicated that there is no uniform process for carrying out and reporting Delphi techniques."
Paré 2013[22]	• "Thirty-one percent of the articles in our sample provided a detailed description of the expert recruitment and selection process, 43% provided only limited details, and 26% did not provide any details."<br>• "All of the articles in our database (n = 42) specified the criteria that were used to select the panel of experts. Position is by far the most used criteria (71%), followed by relevant professional experience (57%), geographic location (7%), and education level (5%)."<br>• "38% of the studies provided detailed information about the participating experts [e.g., 44], 40% provided minimal information [e.g., 2], and 22% did not provide any description".<br>• "The anonymity of the experts was reported in virtually all of the studies (95%) in our sample."<br>• "Only 29% of all of the studies reported the response rate to the initial request for participation."<br>• "35 studies (83%)reported the size of the panels. The majority of the studies (n = 21) reported a panel size between 7 and 30, only one study reported a size of 6 or less, and 13 studies reported panel sizes above 30. Nine studies (19%) examined multiple panels of experts."<br>• "Only 17% of these Delphi studies reported that a pretest of the instruments had been conducted."<br>• "24 studies out of 27 (89%) reported the brainstorming instructions that were sent to the experts."<br>• "Only 8 studies (30%) reported the use of this recommendation. (i.e. Have the experts comment and validate the consolidated list)."<br>• "The vast majority of the studies (85%) reported the final number of items at the end of phase 1."<br>• "Among the 25 studies that did not include this phase (i.e. narrowing down phase), 68% explicitly justified this choice (e.g., the number of items at the end of phase 1 are equal or less than 20 as suggested by Schmidt."<br>• "All 17 studies clearly described the narrowing down instructions that were given to the experts."<br>• "65% of the studies clearly specified their item selection rule."<br>• "Most of the studies (82%) reported the final number of items at the end of the second phase."<br>• "All 42 articles described clearly the ranking instructions that were provided to the experts."<br>• "Almost all of the studies (95%) in our sample reported the statistics that were used for data analysis."<br>• "31% of the studies in our database specified a clear stopping rule."<br>• "Only 15 studies (36%) reported the final consensus rate."<br>• "29 of the 42 studies had multiple rounds of ranking. Of these, the feedback that was provided to the experts in between the rounds included the mean ranks of items (69% of studies), an interpretation of the Kendall's W coefficient (3%), the expert's prior responses (59%), and the comments made by the other experts (38%)."<br>*Recommendations regarding what to report are provided throughout the Results section as well as in the Discussion.*
Resemann 2018[31]	• "Reporting of the Delphi method was critiqued against the AGREE Reporting Checklist."<br>• "All studies reported consensus results. The majority (8/11 [73%]) used a two-stage modified Delphi method, while the remainder used a classic three-stage process. Literature searches guided the development of statements for Delphi panel review in the majority of studies, but only 2/11 (18%) conducted

For peer review only

	systematic literature reviews and merely 6/11 (55%) of studies reported the number of statements assessed. Furthermore, 7/11 (64%) did not report collecting panellist feedback to inform subsequent Delphi stages, 5/11 (45%) of studies did not describe the rating scales used, and 2/11 (18%) omitted reporting the level of consensus reached"<br>• "There is a need for improved reporting of Delphi methods".
Waggoner 2016[23]	• "Despite the widespread utility of consensus methods and the variety of approaches available, there is a lack of guidelines for conducting such studies. This lack of stringency in guidelines for conducting consensus studies has led to variability not only in reporting results but in conducting the studies themselves."<br>• "Many studies describe their methods for collecting data and that they did have a benchmark that would point to a consensus, but a lack of a description of the analytical techniques is apparent in many studies."<br>• "In addition to the lack of descriptive techniques in these articles, there is a wide range of criteria that points to consensus. How these particular benchmarks are determined is also not a topic in many of the studies. Given the lack of current research, we believe that the methodology used I subsequent studies should be described more thoroughly in the manuscript."<br>• "We set out to determine best practices for conducting such research as well as reporting on results in the hopes that future studies are more reliable and valid."<br>*This article provides guidance for reporting of various consensus methods.*
Wang 2015[19]	• "Adoption of reporting guidelines is associated with improved reporting quality of research."<br>• "For example, 28 % of the included guidelines reported no information about consensus, and 57 % were silent about how the feedback after consensus was dealt with."<br>• "In addition to the methodology, only 31 % reported formal consensus method."<br>• "Among guidelines developed through consensus, 30 (50 %) reported group member identification and 31 (52 %) reported member recruitment. Of those who identified members, 27 (45 %) reported specialties of experts, 20 (32 %) described information of members, such as names and institutions, and four (7 %) gave the selection criteria. For those who recruited members, even (12 %) described the recruit methods, for instance, through e-mail, study co-chairs, or group decision. In guidelines developed by a working group, 22 (37 %) reported the number of experts participating in guideline development (median 32, range 3–115). Eleven (18 %) guidelines reported the endpoint of consensus process, which were all terminated after a fixed number of rounds (Table 2). In addition, the inclusion criteria of items were given in eight (13 %) guidelines. For example, items meeting the median score of eight or higher in the final round were included."<br>• "11 (18 %) described the pilot methods, seven (12 %) described the feedback information requirement and five (8 %) gave the methods for feedback collection."<br>• "More than 30 % of the reporting guidelines did not report consensus. For those who did, details of consensus methods were poorly reported."<br>• "Consensus methods should be supported by developers, and the reporting of the methods should be improved."<br>*Recommendations for Consensus methods are provided, but more about improvement of applying and reporting using all other reporting guidelines, but some items are applicable for consensus methodology as well (e.g. reporting COI and funding.*

For peer review only - http://bmjopen.bmj.com/site/about/guidelines.xhtml

**Background**<br>1.1 Does the study suggest anything about how or if consensus papers should report the context or rationale for choosing a consensus method over other methods?	1) Research problem clearly defined and topic and method justification should be reported [Hasson 2000, Figure 1 and page 1013]<br><br>2) Selection of one consensus method over another should be evident if the purpose is clearly stated. [Humphrey-Murto 2017 Med Teach page 16]<br><br>3) What is the rationale for selecting the Delphi procedure? [Humphrey-Murto 2019, Figure 1]<br><br>4) The choice of the Delphi technique as a method of systematically collating expert consultation and building consensus needs to be well justified. A rationale for the choice of the Delphi technique as the most suitable method needs to be provided [Jünger 2017, Box 3, items 1 and 8]
**Background**<br>1.2 Does the study suggest anything about how/what or if consensus papers should report the objectives of the consensus exercise?	1) Define the study objective [Boulkedid 2011, Table 5 page 7]<br><br>2) Define the purpose of the study [Chan 2019, Box 1]<br><br>3) Is the objective of the Delphi study to present results (eg, a list or statement) reflecting the consensus of the group, or does the study aim to merely quantify the level of agreement? [Diamond 2014, Table 6 and page 403] If the aim of the Delphi study is to elicit consensus, then a clear definition for what constitutes consensus should be provided a priori together with threshold values that specify when consensus is reached. If the investigators plan to only quantify the degree of consensus, but not have consensus as a criterion to stop the Delphi study, this should also be explicitly stated [Diamond 2014, page 406]<br><br>4) Research problem clearly defined and topic and method justification should be reported [Hasson 2020, Figure 1 and page 1013]<br><br>5) Authors must provide a clear purpose for their study or line of inquiry [Humphrey-Murto 2017 Med Teach, page 16]<br><br>6) The purpose of the study should be clearly defined and demonstrate the appropriateness of the use of the Delphi technique as a method to achieve the research aim. A rationale for the choice of the Delphi technique as the most suitable method needs to be provided [Jünger 2017, item 8]

For peer review only

	The Delphi technique is a flexible method and can be adjusted to the respective research aims and purposes. Any modifications should be justified by a rationale and be applied systematically and rigorously" [Jünger 2017, item 2]

**Methods**<br>2.1 Does the study the suggest anything about how/what or if consensus papers should report regarding:<br>A literature search/strategy?	1) Describe the selection and preparation of the scientific evidence for the participants [Chan 2019, Box 1]<br><br>2) A literature review should be reported [Hasson 2000, Figure 1]<br><br>3) "We suggest that this important step must be described", but they don't say how. [Humphrey-Murto 2017 AMA, page 1493 and 1496 Partially]<br><br>4) Describe the selection and preparation of the scientific evidence for the participants [Humphrey-Murto 2017 Med Teach, page 16]<br><br>5) Only implying it should happen and be reported [Resemann 2018]
**Methods**<br>2.2 Does study the suggest anything about how/what or if consensus papers should report regarding:<br>Inclusion and exclusion criteria for the literature search?	1) Clear definition of the selection criteria and/or the definition used in the Delphi questionnaire; criteria for selection should be reported [Boulkedid 2011, Table 5, Appendix S1 item 2]<br><br>2) Describe how items were selected for inclusion in questionnaire, in sufficient detail [Chan 2019, Box 1]<br><br>3) Clear selection criteria should be prespecified [Paré 2013 page 210]
**Methods**<br>2.3 Does the study suggest anything of what or if consensus report should report on panel composition, n of participants, expertise, origin? Prespecified?	1) The method used to select participants is stated. Number and type of participant subgroups (eg, patients, generalists and experts) are needed [Banno 2019, page 2 item 1]<br><br>2) The method to include and exclude participants was described. The number and type of participant subgroups (e.g., patients, generalists, and experts) were essential to record [Banno 2020, page 52 item 1]<br><br>3) How the experts were chosen (e.g., willingness to participate, expertise, or membership in an organization); Composition and characteristics of the panel, number of participants (diagram of participant flow); number invited, how they were chosen, whether they were described (age, sex, specialty), years of experience, single or from multiple

For peer review only

	specialties, inclusion of multiple stakeholders, types of stakeholders [Boulkedid 2011, page 2, Table 5, Appendix S1 item 9-15]<br><br>4) Describe how participants were selected and their qualifications. Include description of facilitator credentials [Chan 2019, Box 1]<br><br>5) Were criteria for participants reproducible? How will participants be selected or excluded? [Diamond 2014, Table 5 and 6]<br><br>6) Was there heterogeneity in panel membership and is the method for selection of experts clearly defined [Gattrell 2019, Table 1]<br><br>7) Expert selection process and characteristics should be reported in detail [Hasson 2000, page 1009, 1013]<br><br>8) How many participants were involved? We noted that the type of expertise required of participants was usually not clearly described [Humphrey-Murto 2017 AMA, page 1493 and 1494]<br><br>9) Describe how the participants were selected and their qualifications: if the NGT or RAND/UCLA is used, describe facilitator's credentials. Whatever the makeup of the expert panel, the authors must provide a rationale and justify their choices [Humphrey-Murto 2017 Med Teach]<br><br>10) How many stakeholder/participant groups will be involved in each step? Provide a rationale for inclusion or exclusion and define the stakeholder groups [Humphrey-Murto 2019, Fig 4]<br><br>11) Criteria for the selection of experts and transparent information on recruitment of the expert panel, sociodemographic details including information on expertise regarding the topic in question, (non)response and response rates over the ongoing iterations should be reported [Jünger 2017, Box 3 9]<br><br>12) Describing expert panel selection with eligibility criteria and including conflicts of interest [Ng 2018]<br><br>13) The number of experts in each round should be stated. The backgrounds of the experts should be reported, what kind of expertise they possessed, and the criteria according to which they were selected [Niederberger 2020, page 4]

For peer review only

	14) Explicit procedures for expert selection; Clear selection criteria; Clear selection criteria should be prespecified and may include the candidates' years of related experience, or tenure in a position that is relevant to the subject under study Report the response rate to the initial call for participation; provide detailed information about the participating experts (profile) to better allow judgments about their credibility [Paré 2013, page 210, Table 3]<br><br>15) Explain how groups were chosen. Consensus Development Panels: Panel composition: the panel should be made up of experts in the field; the publication should report on how they were chosen and why; [Waggoner 2016, page 665, 667]<br><br>16) Implied by mentioning that detailed information on participants was lacking in some reporting guidelines. Page 5 Report specialties of experts, names and institutions, the selection criteria [Wang 2015]
**Methods**<br>2.4 Does the study suggest anything of how or if PPI (public patient involvement) activity should be reported	No data
**Methods**<br>2.5 Does the study suggest anything about what or if consensus papers should report regarding panel recruitment strategies, invitations? Any level of detail specified?	1) The use of specific methods to encourage the experts to respond (e.g., stamped addressed envelope for returning the questionnaire and financial compensation) [page 2] and recommendation to report whether special techniques were used to invite participants [Boulkedid 2011, Appendix S1 item 21]<br><br>2) Criteria for the selection of experts and transparent information on recruitment of the expert panel, socio- demographic details including information on expertise regarding the topic in question, (non)response and response rates over the ongoing iterations should be reported" [Jünger 2017, Box 3, 9]<br><br>3) provide a detailed description of the expert recruitment and selection process [Paré 2013, page 215 first bullet on the right]<br><br>4) method of obtaining participants should be described [Waggoner 2016, page 667]
**Methods**	1) The method used to define a consensus among panel members; , whether the percentage of agreement was determined; Whether a cut-off (e.g., median value) was used to select indicators [page 2] Consensus definition at each

For peer review only

2.6 Does the study suggest how or if consensus papers should report the consensus criteria/threshold (or the level of agreement considered to reach consensus)?	round [page 7, Appendix item 28] how was consensus obtained [page 7, Appendix item 28] definition of consensus should be reported [Boulkedid 2011, table 5]<br><br>2) Clearly describe how consensus was defined [Chan 2019, Box 1]<br><br>3) Need to define criteria for consensus and to document the degree of agreement together with the results of the Delphi process. Should be defined a priori. [Diamond 2014, page 404 and table 6]<br><br>4) Was the agreement/consensus threshold predefined? [Gattrell 2019, table 1]<br><br>5) Box 2 Specific threshold for the chosen measure (e.g., median of at least 7 on a nine-point scale and an interquartile range of less than 2) [Grant 2018, p 97]<br><br>6) Determine the criteria and the meaning of `consensus' in relation to the studies [Hasson 2020, page 1013]<br><br>7) No. They do state that "articulating the definition of consensus used" was identified as "particularly problematic and were often left out or poorly described", and that "the most concerning issue we identified was that consensus was often not defined a priori. Only 43.2% of the articles we reviewed reported their definition of consensus at the start of the study." But they do not suggest how to report. [Humphrey-Murto 2017 AMA]<br><br>8) Clearly describe how consensus was defined [Humphrey-Murto 2017 Med Teach, page 18]<br><br>9) suggests definition of consensus should be reported [Humphrey-Murto 2019, table 1, also fig 1 and page 1044]<br><br>10) Definition of consensus. Unless not reasonable due to the explorative nature of the study, an a priori criterion for consensus should be defined. This includes a clear and transparent guide for action on (a) how to proceed with certain items or topics in the next survey round, (b) the required threshold to terminate the Delphi process and (c) procedures to be followed when consensus is (not) reached after one or more iterations". Definition and attainment of consensus. It needs to be comprehensible to the reader how consensus was achieved throughout the process, including strategies to deal with non-consensus". "If an a priori definition of consensus is not realistic due to the explorative nature of the study, it should be identified and established by the research team in the course of the process." [Jünger 2017, item 12]

For peer review only

	11) How was consensus defined and measured? What role did the stability of the answers play? [Niederberger 2020, Table 2] Whether and when consensus was defined in the Delphi studies. Was consensus defined a priori in advance of development of the questionnaire. [Niederberger 2020, Table 5] How was consensus measured, e.g. percentage agreement, units of central tendency (especially median) or a combination of percent agreement within a certain range and for a certain threshold. [Niederberger 2020, page 6]<br><br>12) NGT explain criteria used to determine how and when a consensus was met Consensus Development Panels: Explain what constituted consensus and how this was assessed. [Waggoner 2016, page 665] Delphi Explain what constituted consensus and how this was assessed. [Waggoner 2016, page 667]<br><br>13) The endpoint of consensus [Wang 2015, page 5]
**Methods**<br>2.7 Does the study suggest how or if consensus papers should report how decision of approval of an item will be made?	1) Whether the percentage of agreement was determined [page 2] We recorded the method used to define a consensus among panel members, whether the percentage of agreement was determined, and whether a cut-off (e.g., median value) was used to select [Boulkedid 2011, Appendix S1 item 16 (technique method)]<br><br>2) Reporting on each round separately illustrates clearly the array of themes generated in round one and gives an indication of the strength of support for each round. The presentations of findings are important and findings from subsequent rounds should be reported in a summarized format to indicate the relative standing of each of the opinions. [Hasson 2020, page 1013]<br><br>3) (Non)response and response rates over the ongoing iterations should be reported [Jünger 2017, item 9]
**Methods**<br>2.8 Does the study suggest anything about what level of detail should be reported regarding the number of Delphi rounds or if this should be reported?	1) Was the number of rounds to be performed stated (not how it should be reported, but implies it should be) [Banno 2019, page 2 under item 2]<br><br>2) Was the number of rounds to be performed stated? [Banno 2020, 3.4, table 3]<br><br>3) Describe the number of rounds planned [Chan 2019, Box 1]

For peer review only

	4) Specify a maximum number of rounds [page 404] what was the reason to stop the delphi [Diamond 2014, table 3] What criteria will be used to determine to stop the Delphi process or will the Delphi be run for a specific number of rounds only [Diamond 2014, table 6, table 1 item 2]<br><br>5) number and outline per round should be reported also page 1013 [Hasson 2020, fig 1]<br><br>6) Describe the number of rounds planned and/or criteria for terminating the process [Humphrey-Murto 2017 Med Teach, page 17]<br><br>7) Only implying that x number of rounds are necessary [Humphrey-Murto 2017 AMA]<br><br>8) The methods employed need to be comprehensible; information about the number and design of survey rounds, [Jünger 2017, Box 3 item 10]<br><br>9) Not specifically under item 4 in table 2 report of the specific process used? How many rounds were used in the Delphi technique [Niederberger 2020]<br><br>10) If a study goes beyond the agreed number of rounds (review suggests 2 rounds are required), this should be explained [Waggoner 2016, page 667]
**Methods**<br>2.9 Does the study suggest anything about what level of detail should be reported regarding the criteria used for defining the number of rounds? (why 2-3 or more e.g.) or if this should be reported?	1) Implied in Banno 2020 The prespecified criteria for stopping the Delphi process, other than a statement of the number of rounds, were clarified [Banno 2020]<br><br>2) Describe the number of rounds planned and criteria for terminating the process [Chan 2019, Box 1]<br><br>3) Describe the number of rounds planned and/or criteria for terminating the process [Humphrey-Murto 2017 Med Teach, page 17]<br><br>4) They, imply that the number of rounds is an important thing to report -- but they do not state this as a suggestion.[Humphrey-Murto 2017 AMA]<br><br>5) Will the number of rounds be decided a priori? If not determined a priori, what are the criteria for terminating the process? [Humphrey-Murto 2019, Fig 1]

For peer review only

	6) What was the rationale for the number of rounds; when was the number of rounds defined [Niederberger 2020, page 6]<br>7) Table 3 Report the stopping [Paré 2013]<br>8) For delphi: if a study goes beyond two rounds, explain reason for doing so; [Waggoner 2016, page 667]
**Methods**<br>2.10 Does the study suggest anything about the details that should be reported regarding the time between rounds, if this should be prespecified in advance, or if this should be reported?	1) The time taken to complete the Delphi procedure was recorded [Boulkedid 2011, page 2]
**Methods**<br>2.11 Does the study suggest anything about details that should be reported of the names of the techniques of non-Delphi methods used to gather participants' inputs and reach consensus ?	1) Whether the meeting was held before, after, or between Delphi rounds and what the participants did during the meeting [Boulkedid 2011, page 2]
**Methods**<br>2.12 Does the study suggest anything of what or in which detail	1) What software will be used to administer the Delphi? [Humphrey-Murto 2019, fig 1]

For peer review only

should be reported regarding tool or electronic system used for Delphi? (If Delphi was used)? Or if this should be reported?	
**Methods**<br>2.13 Does the study suggest anything about how or in what level of detail the anonymity of participants (in Delphi or other methods) has to be reported? Or if this should be reported?	1) No, only that it is a limitation of this study that the quality score did not include that. So actually they feel it should be reported how anonymity was maintained [Banno 2020]<br><br>2) Describe how anonymity was defined [Chan 2019, Box 1]<br><br>3) Were responses anonymized [Gattrell 2019, table 1]<br><br>4) It suggests that conducting anonymous iterative mail or e-mail questionnaire rounds is one of the steps [p 1491]. While the authors may have assumed that readers would understand that anonymity was part of their study design, we suggest that they state this, given the variability in approaches that have been labelled as modified consensus methods. [Humphrey-Murto 2017 AMA, page 1497]<br><br>5) Describe how anonymity was maintained. Authors must clearly state how this was accomplished. It is achieved through the use of mail outs in Delphi and RAND/UCLA and private ranking in NGT. [Humphrey-Murto 2017 Med Teach, page 18]<br><br>6) How will anonymity be maintained? [Humphrey-Murto 2019, fig 1]<br><br>7) Ensure the anonymity of the participants. The anonymity of the experts was reported in virtually all of the studies [Paré 2013]
**Methods**<br>2.14 Does the study suggest anything about how to report, and in what level of detail, the feedback for panellists (in	1) Whether the experts were informed of both the response of the group and their own individual response (individual feedback) to each item. The type of feedback, which was defined as qualitative when a summary of the panel's comments was sent to each participant and quantitative when simple statistical summaries illustrating the collective opinion (e.g., central tendency and variance) were sent to each participant [page 2] After each round, each participant should be given the panel results (median, lowest, and highest ratings), the participant's response, and a summary of all comments received. These data inform each participant of his or her position relative to the rest of the group, thus assisting in decisions about replies during future Delphi rounds. [Boulkedid 2011, page 8] It has been recommended that

Delphi rounds or other methods) process? Or if this should be reported?	feedback should include qualitative comments and statistical measures [citation 51, Murphy 1998]. More specifically, we determined whether the experts were informed of both the response of the group and their own individual response (individual feedback) to each item [Boulkedid 2011]<br><br>2) Describe the type of feedback provided after each round [Chan 2019, Box 1]<br><br>3) Were participants' responses in each round reported back to the group, and were responses anonymized? [Gattrell 2019, Table 1]<br><br>4) Give attention to issues which guide data collection: the discovery of opinions, the process of determining the most important issues referring to the design of the initial round, and the management of opinions [Hasson 2020, page 1013]<br><br>5) Was formal feedback provided? If so, was the feedback described? [page 1493], areas that need to be improved with reporting providing participants with feedback of group ratings [Humphrey-Murto 2017 AMA, page 1494]<br><br>6) Describe the type of feedback provided after each round [page 18]. Feedback to participants can include quantitative and/or qualitative data. It also involves two types of agreement: the extent to which individual participants agree with an issue, and the extent to which participants agree with one another. Quantitative feedback may include summary statistics such as the participants' score, participants' medians, range of scores and the proportion of participants selecting each point on a scale. Participants are provided an opportunity to change their ranking, but it should be made clear that they do not need to conform. Researchers may ask the participants who are outliers to provide written justification for their choices (qualitative data) [Humphrey-Murto 2017 Med Teach]<br><br>7) What type of feedback will participants received after each round? [2019] indicates feedback between rounds should include individuals' scores for each item and the distribution of votes by participant group. Some, however, preferred to view aggregated feedback as well as feedback to individual participants [Humphrey-Murto 2019 Yes page 1042, table 1]<br><br>8) How was the feedback designed? [Niederberger 2020, table 2]<br><br>9) Citation [Schmidt, 54] recommends three relevant pieces of feedback that can be provided to experts in phase 3 in addition to mean ranks, namely, the interpretation of Kendall's W from the previous round, the percentage of experts

For peer review only

	placing each item in the top half of their list and the relevant comments that were made by the other panellists [Paré 2013, page 213]<br><br>10) They imply that it should be reported that panellist feedback was collected to inform subsequent Delphi rounds [Resemann 2018]<br><br>11) not about reporting but they state "57 % were silent about how the feedback after consensus was dealt with." suggesting that they felt it needs to be reported. [page 2] only that some reporting guidelines described the feedback information requirement, or gave the methods for feedback collection [Wang 2015, page 6]
**Methods**<br>2.15 Does the study suggest anything about how or if data synthesis/analysis should be reported (from any consensus method used and how this was calculated statistically) and in what level of detail?	1) It is important that standards and norms for prospectively defining analysis plans are needed to improve the credibility of Delphi processes for informing health research, practice, and policy [Grant 2018, page 97]<br><br>2) The methods employed need to be comprehensible; information about methods of data analysis, processing and synthesis of experts' responses to inform the subsequent survey round [Box 3] {Jünger 2017]<br>Reporting of results for each round separately is highly advisable in order to make the evolving of consensus over the rounds transparent. This includes figures showing the average group response, changes between rounds, as well as any modifications of the survey instrument such as deletion, addition or modification of survey items based on previous rounds." [Jünger 2017, item 13]<br><br>3) Detailing statistical analyses and interpretation in arriving at final agreed values [Ng 2018, item 7]<br><br>4) The statistical analyses should be reported [Paré 2013, page 211]<br><br>5) Consensus Development Panels: Statistical analysis: must be reasonable for the research question, and should be as rigorous as possible [Waggoner 2016, page 665]
**Methods**<br>2.16 Does the study suggest anything about how or if piloting should be reported and in what	1) Pilot testing with a small group of individuals is suggested before implementation [Humphrey-Murto 2017 Med Teach, page 16]<br><br>2) All material provided to the expert panel at the outset of the project and throughout the Delphi process should be carefully reviewed and piloted in advance in order to examine the effect on experts' judgements and to prevent bias. [Box 3] The methods employed need to be comprehensible; this includes information on preparatory steps (How was

For peer review only

level of detail (e.g. understanding of consensus items, platforms used, tools used)?	available evidence on the topic in question synthesised?), piloting of material and survey instruments, design of the survey instrument(s), the number and design of survey rounds, methods of data analysis, processing and synthesis of experts' responses to inform the subsequent survey round and methodological decisions taken by the research team throughout the process [Jünger 2017]<br><br>3) Pre-test task instructions and questionnaire instruments [Paré 2013]
**Methods**<br>2.17 Does the study suggest anything about how or if the role of Steering Committee members should be reported?	No data
**Methods**<br>2.18 Does the study suggest anything on what or if should be described regarding COI or funding?	1) 'Sources of funding (industry, non-industry)'as items associated with reporting quality [Banno 2019, page 2]<br><br>2) Is the funding source clearly disclosed? [table 1] Is the role of the funder clearly disclosed? [table 1] Is the funding of any external support (e.g. with the Delphi panel meeting/questionnaires, or medical writing support for the final manuscript) clearly disclosed? [Gattrell 2019]<br><br>3) "Prevention of bias. Researchers need to take measures to avoid directly or indirectly influencing the experts' judgements. If one or more members of the research team have a conflict of interest, entrusting an independent researcher with the main coordination of the Delphi study is advisable" [Jünger 2017]<br><br>4) Describing expert panel selection with eligibility criteria and including conflicts of interest [Ng 2018]
**Methods**<br>2.19 Does the study suggest anything on what should be described of how is dealt with COI of panellist (not allowed	1) No. It only deals with COI as a planning/methodological procedure, not reporting. "5. Prevention of bias. Researchers need to take measures to avoid directly or indirectly influencing the experts' judgements. If one or more members of the research team have a conflict of interest, entrusting an independent researcher with the main coordination of the Delphi study is advisable"[Jünger 2017]

For peer review only

to vote when there is COI)? Or if this should be described	

**Results**<br>3.1 Does the study suggest anything on how to report the initial evidence search (presentation of results of the literature review)?	1) No, but they suggest it should be reported [Jünger 2017]
**Results**<br>3.2 Does the study suggest anything on how to report n of studies found?	No data
**Results**<br>3.3 Does the study recommend which detail should be used when reporting panellists drop-outs (numbers and reasons)? Or if this should be reported?	1) No but it states that number the response rate for the first round dropped to 170 (66.1%). [page 1494]; areas that need improvement in reporting the number of participants after each round [page 1496] Other analyses of consensus methods research found similar poor reporting of this feature, with 7% to 39% of studies reporting response rates for all rounds of data collection [Humphrey-Murto 2017 AMA]<br><br>2) Fig 1 step 7 How will non-responders be managed, i.e. will they be excluded in subsequent rounds What response rate will be acceptable for each stakeholder group in each round? [Humphrey-Murto 2019]<br><br>3) Reporting of results for each round separately is highly advisable in order to make the evolving of consensus over the rounds transparent. This includes figures showing the average group response, changes between rounds, as well as any modifications of the survey instrument such as deletion, addition or modification of survey items based on previous rounds [Jünger 2017, Box 3]<br><br>4) Outlining participation and attrition rates for each round [Ng 2018]

For peer review only

	5) report the response rate to the initial request for participation, the size of the panel and the retention rate; [Paré 2013, page 215 3rd bullet]
**Results**<br>3.4 Does the study suggest how or if approval rates per item shared with respondents for each round should be reported in the Results section?	1) Response rate for each round [Boulkedid 2011, Table 5 on page 7]<br><br>2) Yes Box 1 report response rates and results after each round [Chan 2019]<br><br>3) Response rates for each round should be reported, presentation of total of issues generated in round 1, and presentation of results in round 2 indicating strength of support [Hasson 2000, figure 1 and page 1013]<br><br>4) Report response rates and results after each round [Humphrey-Murto 2017 Med Teach, page 18]<br><br>5) it should report response rates for all rounds [Humphrey-Murto 2019, page 1042]<br><br>6) Reporting of results for each round separately is highly advisable in order to make the evolving of consensus over the rounds transparent. This includes figures showing the average group response, changes between rounds, as well as any modifications of the survey instrument such as deletion, addition or modification of survey items based on previous rounds." [Jünger 2017, item 13]Criteria for the selection of experts and transparent information on recruitment of the expert panel, socio- demographic details including information on expertise regarding the topic in question, (non)response and response rates over the ongoing iterations should be reported". [Jünger 2017]<br><br>7) Reporting both quantitative results and textual comments for each round of analysis [Ng 2018]<br><br>8) How high was the response rate from the experts both when initially approached and also for the individual rounds [Niederberger 2020, Table 2]<br><br>9) Level of consensus should be reported [Resemann 2018]
**Results**<br>3.5 Does the study suggest anything about in which detail the items that have been dropped should	1) Were the criteria for dropping clear; are stopping criteria, other than rounds, reported [Banno 2019, item 3 and 4]<br><br>2) Were the criteria for dropping items clear? (yes, no, or not applicable) [Banno 2020, 2.6 item 3]<br><br>3) Clear criteria for dropping or combining items should also be specified based on the level of agreement or disagreement with individual items. One of the limitations of a priori specification is that certain items may fall just below the

For peer review only

be reported? (reasons e.g.) Or if this should be reported?	threshold for what is fundamentally an arbitrary cut off. In the event that items, believed to be important fell just below the threshold for inclusion in the study, the authors could consider including these items as posteriori considerations provided that sufficient justification was provided. [page 405] Suggested quality criteria: Were criteria for dropping items clear; Stopping criteria other than rounds specified? [Table 5] Were items dropped? What criteria will be used to determine which items to drop? [Diamond 2014, Table 6]<br><br>4) No, but they state Interpretation and processing of results. Consensus does not necessarily imply the correct answer or judgement; (non)consensus and stable disagreement provide informative insights and highlight differences in perspectives concerning the topic in question and Definition and attainment of consensus. It needs to be comprehensible to the reader how consensus was achieved throughout the process, including strategies to deal with non-consensus [Jünger 2017 in Box 3]<br><br>5) Were criteria defined for dropping items [Niederberger 2020, page 6]
**Results**<br>3.6 Does the study make any recommendation on how to report the collection, synthesis and use of comments from panellists? Or if this should be reported?	1) It has been recommended that feedback should include qualitative comments and statistical measures [Murphy 1998, 51]. After each round, each participant should be given the panel results (median, lowest, and highest ratings), the participant's response, and a summary of all comments received [Boulkedid 2011]<br><br>2) Describe the type of feedback provided after each round. Quantitative feedback may include summary statistics such as the participants' score, participants' medians, range of scores and the proportion of participants selecting each point on a scale. Participants are provided an opportunity to change their ranking, but it should be made clear that they do not need to conform [Humphrey-Murto 2017 Med Teach]<br><br>3) Reporting of results for each round separately is highly advisable in order to make the evolving of consensus over the rounds transparent. This includes figures showing the average group response, changes between rounds, as well as any modifications of the survey instrument such as deletion, addition or modification of survey items based on previous rounds [Jünger 2017, item 13]<br><br>4) Ask experts to justify their rankings. Have experts comment and validate consolidated list [page 210 Table 3]. Did experts consolidate the list of items; Did experts comment on and validate the list of items; Was the final number of items reported.<br>Report whether panel members had the opportunity to justify or clarify their own reasoning and to comment on the responses of the other experts as well as on the progress of the panel as a whole. [Paré 2013, page 213].

	Were panellists able to revise previous statements [Paré 2013]<br><br>5) No, but implied that it should be: did not report collecting panellist feedback to inform subsequent Delphi stages [Resemann 2018]
**Results**<br>3.7 Does the study suggest regarding how the final list of items (for clinical guideline or reporting guideline) should be reported? Or if this should be reported?	1) Partially. It says it should be detailed and disseminated, but it does not suggest how (in what format) it should be reported [Jünger 2017]<br><br>2) Suggests "detailing statistical analyses and interpretation in arriving at final agreed values" [Ng 2018]<br><br>3) Report final number of items [Paré 2013, page 210 Table 3]<br><br>4) No but again imply "reported the number of statements assessed." [Resemann 2018]

**Discussion**<br>4.1 Does the paper suggest anything about reporting the limitations and strengths of the study and how? Or if this should be reported?	1) Address potential methodological issues (e.g lack of consensus) or limitations in the discussion (e.g. low response rate) [Chan 2019, Box 1]<br><br>2) Interpretation of consensus gained/not gained [Hasson 2020, page 1009]<br><br>3) In the discussion the authors should address issues that may have impacted the results such as poor response rates between rounds, lack of participation from a select group or geographic region, or lack of consensus. [Humphrey-Murto 2017 Med Teach, page 18]<br><br>4) Methodological issues should be reported [Humphrey-Murto 2019, figure 1]<br><br>5) Reporting should include a critical reflection of potential limitations and their impact of the resulting guidance". [Jünger 2017]
**Discussion**<br>4.2 Does the paper suggest anything about what or in	1) Page 5: is considered a good measure if it meets criteria including reliability, sensitivity, specificity, and feasibility (or applicability) [20,31]. The common use of these characteristics can facilitate acceptance and implementation of indicators developed [Boulkedid 2011]

For peer review only

which detail the applicability generalisability, and reproducibility of the study should be reported? Or if this should be reported?	2) The conclusions should adequately reflect the outcomes of the Delphi study with a view to the scope and applicability of the resulting practice guidance. [Jünger 2017, item 15]<br><br>3) It is also necessary to discuss the critical and rationalistic criteria for the validity and reliability of the studies and the more constructivist characteristics of credibility, transparency, and transferability. [Niederberger 2020, page 8]

**5.1 Any other item proposed by the paper that is not captured in other columns?**	1) Were criteria for dropping items clear? Are stopping criteria, other than rounds, specified [Banno 2019]<br><br>2) Differences between the protocol and the article [Banno 2020, 2.9]<br><br>3) Geographic scope of the survey [page 2]. Main methods used to send the questionnaires (e.g., mail, E-mail, or fax). [Boulkedid 2011, page 7]<br>The formulation of the questionnaire items (e.g., open questions, rating of quality indicators, or both). [Boulkedid 2011]<br>Whether the quality indicators were rated (in which case, we recorded the minimum and maximum values on the rating scale). [Boulkedid 2011]<br>A flow chart of quality indicators (figure showing the output and input indicators at each round) and/or for a written description of indicator flow. [Boulkedid 2011, page 3]<br>Quality indicators used in the first round versus the end of the last round. [Boulkedid 2011, page 3]<br>Availability of the questionnaires in the article itself or in an appendix [Boulkedid 2011, page 3]<br>Whether selection criteria changed between rounds [Boulkedid 2011, page 5]<br>Whether panelists were able to make comments. [Boulkedid 2011, page 6]<br>Whether there was a meeting; at what stage it took place and how people participated [Boulkedid 2011]<br>Response rate for each round [Boulkedid 2011, page 7]<br>preparation in advance of starting Delphi (outcome indicators, structure indicators, process indicators) [Boulkedid 2011, In appendix S1, item 1]<br>METHODS<br>We evaluated the relationship between the response rate and the use of specific methods to encourage the experts to respond (e.g., stamped addressed envelope for returning the questionnaire and financial compensation). Also on maybe we should add item regarding encouragement of participants [Boulkedid 2011, page 2, page 5 right column]<br>Geographic scope of Delphi consensus procedure [Boulkedid 2011,item 20 of appendix and table 5]<br>Question format (open questions, rating scale?) Also in table 5 how were questions formulated? [Boulkedid 2011, item 24

For peer review only

	appendix]<br>Rating scale [Boulkedid 2011, item 25]<br>Methods used to send questionnaire (email fax, mail) [Boulkedid 2011, table 5]<br>Time to complete questionnaire reporting of differences in response rate in rounds [Boulkedid 2011]<br>Number of rounds necessary to reach consensus [Boulkedid 2011]<br>Duration of the procedure [Boulkedid 2011]<br>Is questionnaire added as appendix? [Boulkedid 2011]<br>For Discussion: Validity [Boulkedid 2011]<br><br>4) Outline each step of the process. If modifications were made, provide a rationale for your choices. [Chan 2019]<br>Describe the selection and preparation of the scientific evidence for the participants. [Chan 2019]<br>Include a description of the facilitator's credentials. [Chan 2019]<br>What background material was provided to participants. [Chan 2019]<br>What formal feedback of group rating was shared between rounds [Chan 2019]<br><br>5) Specify stopping criteria in the absence of consensus [Diamond 2014]<br><br>6) Were the questions formulated or validated by an expert panellist [Gattrell 2019]<br><br>7) Researchers conducting consensus-oriented Delphi processes should prospectively and completely register the intended procedure for identifying which items reach consensus. [Grant 2018]<br>The analysis procedure for determining consensus for Delphi processes should be chosen a priori ideally before starting the first round but at the very latest before completing data collection to improve the validity of findings. [Grant 2018]<br>Health researchers conducting consensus-oriented Delphi processes should commit themselves in advance to an analytic procedure for determining which items reach consensus before they see the actual data (or, ideally, before they even collect the data). [Grant 2018]<br>Registrations should be in a publicly available and independently controlled platform that time-stamps entries [Grant 2018]<br><br>8) "Copy of each round questionnaire illustrated" [Hasson 2020]<br>statistical interpretation for the reader [Hasson 2020]<br>appendices to include the questionnaires [Hasson 2020]<br>For Discussion interpretations of consensus gained/not gained reliability and validity [Hasson 2020]

For peer review only

	9) *Page 1493(2) Was background information provided to the participants? pg 1496 areas appeared particularly problematic and were often left out or poorly described: providing background information to participants<br>AND so a clear description of what information was provided and in what format is important<br>* (3) Was the consensus method used for item generation, ranking, or both?<br>* (11) Was consensus forced?<br>Was mail/e-mail polling or face-to-face questioning used? [Humphrey-Murto 2017 AMA]<br><br>10) Outline each step of the process: if modifications were made, provide a rationale for the choices made. Providing justification for the choices made will also add credibility. [Humphrey-Murto 2017 Med Teach]<br><br>11) Background provided to participants, what is level of detail provided [Humphrey-Murto 2019]<br>Figure 1 clear outline of the overall process involved and where Delphi fits [Humphrey-Murto 2019, figure 1]<br>How sample size is determined of participants [Humphrey-Murto 2019, figure 1]<br><br>12) Any modifications should be justified by a rationale and be applied systematically and rigorously [Jünger 2017, Box 3]<br>All material provided to the expert panel at the outset of the project and throughout the Delphi process should be carefully reviewed and piloted in advance in order to examine the effect on experts' judgements and to prevent bias [Jünger 2017]<br>It is recommended to have the final draft of the resulting guidance on best practice in palliative care reviewed and approved by an external board or authority before publication and dissemination [Jünger 2017, Box 3]<br>information about methodological decisions taken by the research team throughout the process Jünger 2017, Box 3]<br>Flow chart to illustrate the stages of the Delphi process, including a preparatory phase, the actual Delphi rounds, interim steps of data processing and analysis, and concluding steps [Jünger 2017, Box 3]<br>Publication and dissemination [Jünger 2017, Box 3]<br><br>13) Item 2-4 and 9 appending revised questionnaires [Ng 2018]<br><br>14) Specific definition of underlying Delphi technique (or as I thought it is important to define exactly what method is used, especially if a modified method is used this needs to be very clear [Niederberger 2020]<br>What role did the stability of the answers play? [Niederberger 2020, table 2]<br>Questionnaire and scale development How were the questionnaires and the specific items for a Delphi technique

For peer review only

	developed? [Niederberger 2020]<br>Nevertheless, it is important to precisely describe, justify, and methodologically reflect on any modifications [Niederberger 2020]<br>How were the questionnaires and the specific items for a Delphi technique developed? [Niederberger 2020, Table 2]<br>Were items identified from empirical analyses such as qualitative interviews or focus groups that were completed in advance or were taken from existing guidelines. [Niederberger 2020, Complementary AND page 6<br>Was the first (qualitative) round of questions in the Delphi process used to generate the items for a standardized questionnaire. [Niederberger 2020, Complementary AND page 6]<br><br>15) Was the final number of items reported [Paré 2013, Table 3] Were items randomly ordered [Paré 2013, Table 3]<br><br>16) Describe the rating scales used [Resemann 2018] the number of statements assessed should be reported [Resemann 2018]<br><br>17) For nominal group process, the research question used to prompt the panel must be clear and concise to obtain valid suggestions from panel members. [Waggoner 2016, page 665] The heterogeneity should be reported [Waggoner 2016, page 665] Evaluation of reliability [Waggoner 2016, page 665]<br><br>18) Meeting attendance; format (e.g. face-to-face); agenda preparation; materials sent to participants prior to meeting; duration of meeting [Wang 2015, page 5] Flow diagram [Wang 2015, page 3] Should we add something regarding other consensus methods including an item regarding face to face meetings? [Wang 2015, page 5]
**5.2 Any other item not proposed by the paper, but you think that could be added (not fitting the categories above)?**	1) Are stopping criteria, other than rounds, specified? [Banno 2019, page 2]<br><br>2) Information letter explaining the method and the reasons their participation to the whole process would be necessary, as well as a form for collecting their consent to complete the entire Delphi process. [Boulkedid 2011]<br><br>3) "Round 1: presentation of total number of issues generated" [Hasson 2020]<br><br>4) This paper was "pointing fingers", showing what was wrong, without suggesting solutions. However, we can be inspired by the critics to build the following list of items: 1) Purpose of the consensus study<br>Whether a literature review was done to support the selection of items [Humphrey-Murto 2017 AMA]<br><br>5) Length of the background provided [Humphrey-Murto 2019]

For peer review only

	Purpose of study: outcome/diagnosis/intervention? [Humphrey-Murto 2019]

**Examples of text with well reported methods/results (for E&E document) - write NA if none was cited or found by you**	1) Page 7 Table 5 [Boulkedid 2011]<br>2) Box 1 [Chan 2019]<br>3) Might have a look at table 6 [Diamond 2014]<br>4) Table 1 [Gattrell 2019]<br>5) Parts of Fig 1 and checklist page 1013 [Hasson 2020]<br>6) Table 1 lists "exemplary publications" for nominal group process, consensus development panel and Delphi technique Page 667 references studies that were "Very descriptive" of the statistical techniques used. [Waggoner 2016]
**Additional comments from assessor**	1) Limited value; protocol for Banno 2020 [Banno 2019]<br>2) Of limited use. The authors developed a 4-point quality score that they applied to Delphi publications [Banno 2020]<br>3) Excellent resource [Boulkedid 2011]<br>4) Focusses on defining consensus [Diamond 2014]<br>5) Congress poster only [Gattrell 2019]<br>6) Study used RAND's ExpertLens as the Delphi platform [Grant 2018]<br>7) 1497: The lack of consensus on consensus methods makes it imperative that researchers provide clear and detailed reporting of the methods they used and that they justify these choices. [Humphrey-Murto 2017]

For peer review only

	8) Page 1044 A suggestion to improv uniformity is to use a software program that provides structure and help with reporting all relevant outcomes (e.g. DelphiManager, http://comet-initiative.org/delphimanager/) [Humphrey-Murto 2019]<br><br>9) Very informative [Jünger 2017]<br><br>10) The study focusses on information systems. Arguably, this is not within the inclusion criteria for the search [Paré 2013]<br><br>11) Review covers nominal group process, consensus development panel and Delphi technique [Waggoner 2016]<br><br>12) Study looked at the reporting quality of reporting guidelines [Wang 2015]

For peer review only

ACcurate COnsensus Reporting Document (ACCORD): Summary of extracted data from literature search

Table of Contents

1. Background ... 2

2. Methods ... 3

3. Results ... 9

4. Discussion ... 11

5. Additional topics ... 12

References ... 14

For peer review only

For peer review only - http://bmjopen.bmj.com/site/about/guidelines.xhtml

Section: Background

1. Background

Data extraction question	Articles	Checklist item(s) with brief explanation
1.1. Does the study suggest anything about how or if consensus papers should report the context or rationale for choosing a consensus method over other methods?	Hasson F, *et al. J Adv Nurs* 2000[1]<br>Humphrey-Murto S, *et al. Med Teach* 2017[2]<br>Humphrey-Murto S, *et al. J Rheumatol* 2019[3]<br>Jünger S, *et al. Palliat Med* 2017[4]	State the rationale for use of consensus method over other options.<br>*Should consider other consensus methods as well as other methodology types.*
1.2. Does the study suggest anything about how/what or if consensus papers should report the objectives of the consensus exercise?	Hasson F, *et al. J Adv Nurs* 2000[1]<br>Humphrey-Murto S, *et al. Med Teach* 2017[2]<br>Jünger S, *et al. Palliat Med* 2017[4]<br>Boulkedid R, *et al. PLoS One* 2011[5]<br>Chan TM, *et al. CJEM* 2019[6]<br>Diamond IR, *et al. J Clin Epidemiol* 2014[7]	Clearly define study objectives.<br>*Could include presentation of group consensus, or just to quantify the level of agreement.*

For peer review only

Section: Methods

2. Methods

Data extraction question	Articles	Checklist item(s) with brief explanation
2.1. Does the study suggest anything about how/what or if consensus papers should report regarding:<br><br>A literature search/strategy?	Hasson F, *et al. J Adv Nurs* 2000[1]<br>Humphrey-Murto S, *et al. Med Teach* 2017[2]<br>Chan TM, *et al. CJEM* 2019[6]<br>Humphrey-Murto S, *et al. Acad Med* 2017[8]<br>Resemann HK, *et al. Curr Med Res Opin* 2018[9]	A) Describe the strategy for reviewing the existing scientific evidence that informed the study.<br>*If no existing literature is available, the extent of the search should be described.*<br>B) Describe how existing scientific evidence will be provided to the participants.<br>*If different participant groups are involved, it should be stated which information will be provided to which group.*
2.2. Does the study suggest anything about how/what or if consensus papers should report regarding:<br><br>Inclusion and exclusion criteria for the literature search?	Boulkedid R, *et al. PLoS One* 2011[5]<br>Chan TM, *et al. CJEM* 2019[6]<br>Paré G, *et al. Inf Manag* 2013[10]	Describe the process of the literature search.<br>*Should include inclusion and exclusion criteria, and state whether these were prespecified.*
2.3. Does the study suggest anything of what or if consensus report should report on panel composition, n of participants, expertise, origin? Prespecified?	Hasson F, *et al. J Adv Nurs* 2000[1]<br>Humphrey-Murto S, *et al. Med Teach* 2017[2]<br>Humphrey-Murto S, *et al. J Rheumatol* 2019[3]<br>Jünger S, *et al. Palliat Med* 2017[4]<br>Boulkedid R, *et al. PLoS One* 2011[5]<br>Chan TM, *et al. CJEM* 2019[6]<br>Diamond IR, *et al. J Clin Epidemiol* 2014[7]<br>Humphrey-Murto S, *et al. Acad Med* 2017[8]<br>Paré G, *et al. Inf Manag* 2013[10]<br>Banno M, *et al. J Clin Epidemiol* 2019[11]<br>Banno M, *et al. J Clin Epidemiol* 2020[12]<br>Gattrell WT, *et al. Curr Med Res Opin* 2019[13]	A) Describe the structure of the study's participants.<br>*Should describe inclusion of a Chair/Co-chairs, steering committee, and subgroups, if applicable.*<br>B) Explain how panel participants were selected.<br>*Should state who was responsible for panellist selection, the selection criteria applied, the justification for choosing panellist numbers and selection criteria, and whether criteria were prespecified.*

For peer review only - http://bmjopen.bmj.com/site/about/guidelines.xhtml

Section: Methods

Data extraction question	Articles	Checklist item(s) with brief explanation
	Ng J. *Value Health* 2018[14]<br>Niederberger M, *et al. Front Public Health* 2020[15]<br>Waggoner J, *et al. Acad Med* 2016[16]<br>Wang X, *et al. BMC Med Res Methodol* 2015[17]	*C)* Describe the composition of the panel. *Should include number of participants at all stages of the process, sociodemographics (e.g. age, sex, specialty, type and duration of relevant experience). Should also describe panel subgroups, if relevant.*<br>D) Describe the expertise of the panel. *Should include the definition of "expert" and description of any public or patients involved.*<br>E) Describe the facilitator(s), if used. *Should include type and duration of relevant experience, and the role played in the process.*
2.4. Does the study suggest anything of how or if PPI (public patient involvement) activity should be reported	No data	Describe the role and involvement of any public or patients. *Should detail the stage(s) at which they were involved, and their roles and contributions.*
2.5. Does the study suggest anything about what or if consensus papers should report regarding panel recruitment strategies, invitations? Any level of detail specified?	Jünger S, *et al. Palliat Med* 2017[4]<br>Boulkedid R, *et al. PLoS One* 2011[5]<br>Paré G, *et al. Inf Manag* 2013[10]<br>Waggoner J, *et al. Acad Med* 2016[16]	Describe how the panel members were recruited. *Could include communication/advertisement method(s) and locations.*
2.6. Does the study suggest how or if consensus papers should report the consensus criteria/threshold (or the level of agreement considered to reach consensus)?	Hasson F, *et al. J Adv Nurs* 2000[1]<br>Humphrey-Murto S, *et al. Med Teach* 2017[2]<br>Humphrey-Murto S, *et al. J Rheumatol* 2019[3]<br>Jünger S, *et al. Palliat Med* 2017[4]<br>Boulkedid R, *et al. PLoS One* 2011[5]<br>Chan TM, *et al. CJEM* 2019[6]<br>Diamond IR, *et al. J Clin Epidemiol* 2014[7]<br>Humphrey-Murto S, *et al. Acad Med* 2017[8]<br>Gattrell WT, *et al. Curr Med Res Opin* 2019[13]	A) Define the consensus measure to be used. *Could include percentage agreement, units of central tendency (e.g. median), a categorical rating (e.g. Agree/Strongly agree) or a combination of percent agreement within a certain range.*<br>B) State the threshold for the group achieving consensus. *Should include whether the threshold was*

For peer review only - http://bmjopen.bmj.com/site/about/guidelines.xhtml

Section: Methods

Data extraction question	Articles	Checklist item(s) with brief explanation
	Niederberger M, *et al. Front Public Health* 2020[15]<br>Waggoner J, *et al. Acad Med* 2016[16]<br>Wang X, *et al. BMC Med Res Methodol* 2015[17]<br>Grant S, *et al. J Clin Epidemiol* 2018[18]	*pre-defined and highlight any threshold variation between rounds, with explanation for the change. If the intention is to quantify the degree of consensus but not to use consensus as a stop criterion for the study, this should be stated.*
2.7. Does the study suggest how or if consensus papers should report how decision of approval of an item will be made?	Hasson F, *et al. J Adv Nurs* 2000[1]<br>Jünger S, *et al. Palliat Med* 2017[4]<br>Boulkedid R, *et al. PLoS One* 2011[5]	Explain how final consensus was reached.<br>*Should describe the evolution of themes between voting rounds, if applicable.*
2.8. Does the study suggest anything about what level of detail should be reported regarding the number of Delphi rounds or if this should be reported?	Hasson F, *et al. J Adv Nurs* 2000[1]<br>Humphrey-Murto S, *et al. Med Teach* 2017[2]<br>Jünger S, *et al. Palliat Med* 2017[4]<br>Chan TM, *et al. CJEM* 2019[6]<br>Diamond IR, *et al. J Clin Epidemiol* 2014[7]<br>Humphrey-Murto S, *et al. Acad Med* 2017[8]<br>Banno M, *et al. J Clin Epidemiol* 2019[11]<br>Banno M, *et al. J Clin Epidemiol* 2020[12]<br>Niederberger M, *et al. Front Public Health* 2020[15]<br>Waggoner J, *et al. Acad Med* 2016[16]	State how many voting rounds were conducted.<br>*Should include whether the number of rounds was prespecified, and whether this was an absolute or a maximum. If the maximum was exceeded, should explain the reasoning for doing so.*
2.9. Does the study suggest anything about what level of detail should be reported regarding the criteria used for defining the number of rounds? (why 2-3 or more e.g.) or if this should be reported?	Humphrey-Murto S, *et al. Med Teach* 2017[2]<br>Humphrey-Murto S, *et al. J Rheumatol* 2019[3]<br>Chan TM, *et al. CJEM* 2019[6]<br>Humphrey-Murto S, *et al. Acad Med* 2017[8]<br>Paré G, *et al. Inf Manag* 2013[10]<br>Banno M, *et al. J Clin Epidemiol* 2020[12]<br>Niederberger M, *et al. Front Public Health* 2020[15]<br>Waggoner J, *et al. Acad Med* 2016[16]	Explain the rationale for choosing the number of voting rounds.<br>*Should also describe the stop criteria, if used, and whether these were prespecified.*
2.10. Does the study suggest anything about the details that should be reported regarding the time between rounds, if this should be	Boulkedid R, *et al. PLoS One* 2011[5]	Describe the time period between voting rounds.<br>*Should include whether the period was*

For peer review only - http://bmjopen.bmj.com/site/about/guidelines.xhtml

Section: Methods

Data extraction question	Articles	Checklist item(s) with brief explanation
prespecified in advance, or if this should be reported?		*prespecified and highlight differences between inter-round periods, if applicable.*
2.11. Does the study suggest anything about details that should be reported of the names of the techniques of non-Delphi methods used to gather participants' inputs and reach consensus?	Boulkedid R, *et al. PLoS One* 2011[5]<br>Waggoner J, *et al. Acad Med* 2016[16]	Describe any additional methods used alongside the consensus process.<br>*Should include all that were used, e.g. a self-administered questionnaire combined with a group meeting. Should also explain how the consensus process fitted into the overall study methodology.*
2.12. Does the study suggest anything of what or in which detail should be reported regarding tool or electronic system used for Delphi? (If Delphi was used)? Or if this should be reported?	Humphrey-Murto S, *et al. J Rheumatol* 2019[3]	Describe any tools used to administer the voting.<br>*Could detail electronic platforms, if used.*
2.13. Does the study suggest anything about how or in what level of detail the anonymity of participants (in Delphi or other methods) has to be reported? Or if this should be reported?	Humphrey-Murto S, *et al. Med Teach* 2017[2]<br>Humphrey-Murto S, *et al. J Rheumatol* 2019[3]<br>Chan TM, *et al. CJEM* 2019[6]<br>Humphrey-Murto S, *et al. Acad Med* 2017[8]<br>Paré G, *et al. Inf Manag* 2013[10]<br>Banno M, *et al. J Clin Epidemiol* 2020[12]<br>Gattrell WT, *et al. Curr Med Res Opin* 2019[13]	Detail how anonymity of voters was maintained.<br>*Could involve use of mail-outs in a standard Delphi procedure, blinding on an electronic platform, or private ranking in the NGT.*
2.14. Does the study suggest anything about how to report, and in what level of detail, the feedback for panellists (in Delphi rounds or other methods) process? Or if this should be reported?	Hasson F, *et al. J Adv Nurs* 2000[1]<br>Humphrey-Murto S, *et al. Med Teach* 2017[2]<br>Humphrey-Murto S, *et al. J Rheumatol* 2019[3]<br>Boulkedid R, *et al. PLoS One* 2011[5]<br>Chan TM, *et al. CJEM* 2019[6]<br>Humphrey-Murto S, *et al. Acad Med* 2017[8]<br>Resemann HK, *et al. Curr Med Res Opin* 2018[9]<br>Paré G, *et al. Inf Manag* 2013[10]<br>Gattrell WT, *et al. Curr Med Res Opin* 2019[13]<br>Niederberger M, *et al. Front Public Health* 2020[15]	Explain how voting feedback was provided to panellists at the end of each round.<br>*Could include summaries of group voting and/or their own individual responses. Should state whether feedback will be quantitative and/or qualitative, and whether it will be anonymised. If no feedback was provided, this should be stated.*

For peer review only - http://bmjopen.bmj.com/site/about/guidelines.xhtml

Section: Methods

Data extraction question	Articles	Checklist item(s) with brief explanation
	Wang X, *et al. BMC Med Res Methodol* 2015[17]	
2.15. Does the study suggest anything about how or if data synthesis/analysis should be reported (from any consensus method used and how this was calculated statistically) and in what level of detail?	Jünger S, *et al. Palliat Med* 2017[4]<br>Paré G, *et al. Inf Manag* 2013[10]<br>Ng J. *Value Health* 2018[14]<br>Waggoner J, *et al. Acad Med* 2016[16]<br>Grant S, *et al. J Clin Epidemiol* 2018[18]	Detail methods used to process responses after each voting round.<br>*Could include statistical analysis methods, if used.*
2.16. Does the study suggest anything about how or if piloting should be reported and in what level of detail (e.g. understanding of consensus items, platforms used, tools used)?	Humphrey-Murto S, *et al. Med Teach* 2017[2]<br>Jünger S, *et al. Palliat Med* 2017[4]<br>Paré G, *et al. Inf Manag* 2013[10]	Describe any piloting of the study materials and/or survey instruments.<br>*Should include the number of individuals in the pilot group and the rationale for their selection. Should also explain any changes made as a result of the pilot. If no pilot was conducted, this should be stated.*
2.17. Does the study suggest anything about how or if the role of Steering Committee members should be reported?	No data	Describe the role(s) of the Steering Committee in the process.<br>*Should also detail the involvement of the Chair/Co-chairs, subgroups, or individual members at relevant stages of the process, if different from the group as a whole.*
2.18. Does the study suggest anything on what or if should be described regarding COI or funding?	Jünger S, *et al. Palliat Med* 2017[4]<br>Banno M, *et al. J Clin Epidemiol* 2019[11]<br>Gattrell WT, *et al. Curr Med Res Opin* 2019[13]<br>Ng J. *Value Health* 2018[14]	A) Disclose any COI of the panellists<br>*Should specify COI of each participant in the panel.*<br>B) Disclose any funding received and the role of the funder.<br>*Should specify the role of the funding source(s), e.g. involvement in the study concept/design, participation of the Steering Committee, for conducting the consensus process, medical writing support for its reporting.*

For peer review only

Section: Methods

Data extraction question	Articles	Checklist item(s) with brief explanation
2.19. Does the study suggest anything on what should be described of how is dealt with COI of panellist (not allowed to vote when there is COI)? Or if this should be described	Jünger S, *et al*. *Palliat Med* 2017[4]	Describe measures taken to avoid influence by any conflicts of interest (COI). *Should include disclosure of COI and how this was accounted for in the methodology, e.g. by limiting voting in case of a specific COI, adjudication by an independent researcher.*

For peer review only

Section: Results

3. Results

Data extraction question	Articles	Checklist item(s) with brief explanation
3.1. Does the study suggest anything on how to report the initial evidence search (presentation of results of the literature review)?	Jünger S, *et al*. *Palliat Med* 2017[4]	Describe how existing scientific evidence was provided to the participants.<br>*Should include relevant specifics of the literature search, e.g. n of studies reported, to provide relevant context for the results. If different participant groups were involved, it should be stated which information was provided to which group.*
3.2. Does the study suggest anything on how to report n of studies found?	No data	Describe the results of the search and number of included studies.
3.3. Does the study recommend which detail should be used when reporting panellists drop-outs (numbers and reasons)? Or if this should be reported?	Humphrey-Murto S, *et al. J Rheumatol* 2019[3]<br>Jünger S, *et al*. *Palliat Med* 2017[4]<br>Humphrey-Murto S, *et al*. *Acad Med* 2017[8]<br>Paré G, *et al*. *Inf Manag* 2013[10]<br>Ng J. *Value Health* 2018[14]	A) State the response rates for each voting round.<br>*Should specify n as well as percent, or otherwise indicate attrition/retention rates.*<br>B) State the reasons cited for voter drop-outs at each stage of the process.<br>*Could be provided as an aggregated summary or as individual responses. If this information was not collected, this should be stated.*<br>C) Describe measures undertaken to maintain acceptable response rates.<br>*If threshold rates differ between stakeholder groups, these should be described with explanation.*

For peer review only - http://bmjopen.bmj.com/site/about/guidelines.xhtml

Section: Results

Data extraction question	Articles	Checklist item(s) with brief explanation
3.4. Does the study suggest how or if approval rates per item shared with respondents for each round should be reported in the Results section?	Hasson F, *et al. J Adv Nurs* 2000[1]<br>Humphrey-Murto S, *et al. Med Teach* 2017[2]<br>Humphrey-Murto S, *et al. J Rheumatol* 2019[3]<br>Jünger S, *et al. Palliat Med* 2017[4]<br>Boulkedid R, *et al. PLoS One* 2011[5]<br>Chan TM, *et al. CJEM* 2019[6]<br>Resemann HK, *et al. Curr Med Res Opin* 2018[9]<br>Ng J. *Value Health* 2018[14]<br>Niederberger M, *et al. Front Public Health* 2020[15]	Describe which results that were shared with respondents after each voting round were reported in the final manuscript.<br>*Could include response rates, the type of information presented, summaries of group voting and/or individual responses. If this information is not provided, this should be stated together with the rationale.*
3.5. Does the study suggest anything about in which detail the items that have been dropped should be reported? (reasons e.g.) Or if this should be reported?	Jünger S, *et al. Palliat Med* 2017[4]<br>Diamond IR, *et al. J Clin Epidemiol* 2014[7]<br>Banno M, *et al. J Clin Epidemiol* 2019[11]<br>Banno M, *et al. J Clin Epidemiol* 2020[12]<br>Niederberger M, *et al. Front Public Health* 2020[15]	A) List any voting items that were dropped.<br>B) Explain the rationale for dropping any voting items.<br>*Should state whether the criteria for dropping any items were prespecified.*
3.6. Does the study make any recommendation on how to report the collection, synthesis and use of comments from panellists? Or if this should be reported?	Humphrey-Murto S, *et al. Med Teach* 2017[2]<br>Jünger S, *et al. Palliat Med* 2017[4]<br>Boulkedid R, *et al. PLoS One* 2011[5]<br>Resemann HK, *et al. Curr Med Res Opin* 2018[9]<br>Paré G, *et al. Inf Manag* 2013[10]	Describe how responses were processed prior to reporting.<br>*Should describe methods by which responses were analysed, aggregated or summarised, include whether any statements were revised between voting rounds, and state by whom the information was processed.*
3.7. Does the study suggest regarding how the final list of items (for clinical guideline or reporting guideline) should be reported? Or if this should be reported?	Jünger S, *et al. Palliat Med* 2017[4]<br>Resemann HK, *et al. Curr Med Res Opin* 2018[9]<br>Paré G, *et al. Inf Manag* 2013[10]<br>Ng J. *Value Health* 2018[14]	Report the final outcomes.<br>*Could be quantitative (e.g. summary statistics, score means, medians and/or ranges) and/or qualitative (e.g. aggregated themes from comments). Should be clear, accurately represent the consensus methodology used, and relevant to the field.*

For peer review only - http://bmjopen.bmj.com/site/about/guidelines.xhtml

Section: Discussion

4. Discussion

Data extraction question	Articles	Checklist item(s) with brief explanation
4.1. Does the paper suggest anything about reporting the limitations and strengths of the study and how? Or if this should be reported?	Hasson F, *et al. J Adv Nurs* 2000[1]<br>Humphrey-Murto S, *et al. Med Teach* 2017[2]<br>Humphrey-Murto S, *et al. J Rheumatol* 2019[3]<br>Jünger S, *et al. Palliat Med* 2017[4]<br>Chan TM, *et al. CJEM* 2019[6]	Discuss the study's methodological strengths and limitations.<br>*Should address issues that may impact results, e.g. response rates or representation.*
4.2. Does the paper suggest anything about what or in which detail the applicability generalisability, and reproducibility of the study should be reported? Or if this should be reported?	Jünger S, *et al. Palliat Med* 2017[4]<br>Boulkedid R, *et al. PLoS One* 2011[5]<br>Niederberger M, *et al. Front Public Health* 2020[15]	A) Discuss the reliability of the study.<br>B) Discuss the sensitivity of the study.<br>C) Discuss the specificity of the study.<br>D) Discuss the applicability of the study.<br>E) Discuss the validity of the study.

For peer review only

Section: Additional topics

5. Additional topics

Data extraction question: Any other item proposed by the paper that is not captured in previous sections?

Articles	Checklist item(s) with brief explanation
Humphrey-Murto S, *et al. Med Teach* 2017[3]<br>Jünger S, *et al. Palliat Med* 2017[4]<br>Boulkedid R, *et al. PLoS One* 2011[5]<br>Paré G, *et al. Inf Manag* 2013[10]<br>Banno M, *et al. J Clin Epidemiol* 2020[12]	Explain any deviations from the planned protocol.<br>*Should include any affected stages, including but not limited to change in panel number or composition, number of voting rounds, stopping criteria, statistical plan, reporting of outcomes.*
Boulkedid R, *et al. PLoS One* 2011[5]<br>Resemann HK, *et al. Curr Med Res Opin* 2018[9]	Describe the formulation of questions.<br>*Should include the type of questions, e.g. open questions, numerical rating, level of agreement rating. If rating questions were used, the scale range should be stated, and whether respondents were able to leave additional comments after rating items.*
Boulkedid R, *et al. PLoS One* 2011[5]<br>Wang X, *et al. BMC Med Res Methodol* 2015[17]	Describe any group meetings that were held.<br>*Should state at what stage the meeting took place, objectives/purpose, format (e.g. face-to-face or virtual), pre-read materials shared, attendance, location, duration, and how individuals participated.*
Hasson F, *et al. J Adv Nurs* 2000[1]<br>Boulkedid R, *et al. PLoS One* 2011[5]<br>Ng J. *Value Health* 2018[14]	List any items included in the appendix accompanying the main report.<br>*Could include e.g. full voting questions from each round with response rates, or information provided to the panel as pre-reads or to summarise voting rounds.*
Boulkedid R, *et al. PLoS One* 2011[5]	State how the survey was presented to participants.<br>*For example, as hard copy or via digital platform; could include description of email or mailing process. Should describe any randomisation procedures for questions, if used. If questions were not randomised, this should be stated.*
Boulkedid R, *et al. PLoS One* 2011[5]	Describe incentives for encouraging responses.<br>*Should list any specific methods, e.g. paid return postage for the questionnaire or financial compensation.*
Boulkedid R, *et al. PLoS One* 2011[5]	State the period in which the process was conducted.
Grant S, *et al. J Clin Epidemiol* 2018[18]	Describe any prospective registrations for the consensus process.

For peer review only - http://bmjopen.bmj.com/site/about/guidelines.xhtml

Section: Additional topics

Articles	Checklist item(s) with brief explanation
	Should include the platform on which it was registered and a link, if applicable. If the process was not registered, this should be stated.
Jünger S, *et al*. *Palliat Med* 2017[4]	Describe any external peer review prior to publication.<br>*Should name the authority, state the rationale for their review, and describe any modifications made as a result of their review.*
Humphrey-Murto S, *et al. Med Teach* 2017[3]<br>Jünger S, *et al*. *Palliat Med* 2017[4]	Describe the overall process using a flow chart or diagram.
Paré G, *et al. Inf Manag* 2013[10]<br>Niederberger M, *et al. Front Public Health* 2020[15]	Explain how the initial voting items in the consensus were developed.<br>*Could describe e.g. development from empirical analyses, qualitative interviews, advance focus groups, brainstorming, or existing guidelines. Should state who consolidated the information and developed the voting items.*
Boulkedid R, *et al. PLoS One* 2011[5]	Describe the procedure for collecting participants' consent to complete the full consensus process.<br>*Could briefly describe any forms used and how the data were collected and stored.*

For peer review only

Section: References

References

1. Hasson F, Keeney S, McKenna H. Research guidelines for the Delphi survey technique. *J Adv Nurs* 2000;32:1008-15. doi: 10.1046/j.1365-2648.2000.t01-1-01567.x.
2. Humphrey-Murto S, Varpio L, Gonsalves C, et al. Using consensus group methods such as Delphi and Nominal Group in medical education research. *Med Teach* 2017;39:14-9. doi: 10.1080/0142159X.2017.1245856.
3. Humphrey-Murto S, Crew R, Shea B, et al. Consensus Building in OMERACT: Recommendations for Use of the Delphi for Core Outcome Set Development. *J Rheumatol* 2019;46:1041-6. doi: 10.3899/jrheum.181094.
4. Jünger S, Payne SA, Brine J, et al. Guidance on Conducting and REporting DElphi Studies (CREDES) in palliative care: Recommendations based on a methodological systematic review. *Palliat Med* 2017;31(8):684-706. doi: 10.1177/0269216317690685.
5. Boulkedid R, Abdoul H, Loustau M, et al. Using and Reporting the Delphi Method for Selecting Healthcare Quality Indicators: A Systematic Review. *PLoS One* 2011;6:e20476. doi: 10.1371/journal.pone.0020476.
6. Chan TM, Yarris LM, Humphrey-Murto S. Delving into Delphis. *CJEM* 2019;21:167-9. doi: 10.1017/cem.2019.3.
7. Diamond IR, Grant RC, Feldman BM, et al. Defining consensus: A systematic review recommends methodologic criteria for reporting of Delphi studies. *J Clin Epidemiol* 2014;67:401-9. doi: 10.1016/j.jclinepi.2013.12.002.
8. Humphrey-Murto S, Varpio L, Wood TJ, et al. The Use of the Delphi and Other Consensus Group Methods in Medical Education Research: A Review. *Acad Med* 2017;92:1491-8. doi: 10.1097/ACM.0000000000001812.
9. Resemann HK, Clements S, Griffiths A, et al. Reporting of Delphi methods to achieve consensus on guidelines in rare diseases. *Curr Med Res Opin* 2018;34 (Suppl 1):37.
10. Paré G, Cameron AF, Poba-Nzaou P, et al. A systematic assessment of rigor in information systems ranking-type Delphi studies. *Inf Manag* 2013;50:207-17 doi: 10.1016/j.im.2013.03.003.
11. Banno M, Tsujimoto Y, Kataoka Y. Reporting quality of the Delphi technique in reporting guidelines: a protocol for a systematic analysis of the EQUATOR Network Library. *J Clin Epidemiol* 2019;9:e024942.
12. Banno M, Tsujimoto Y, Kataoka Y. The majority of reporting guidelines are not developed with the Delphi method: a systematic review of reporting guidelines. *J Clin Epidemiol* 2020;124;50-7. doi: 10.1016/j.jclinepi.2020.04.010.
13. Gattrell WT, Clements SJ, Sheard D. Quality assessment of guidelines/recommendations developed using Delphi methodology. *Curr Med Res Opin* 2019;35 (Suppl 2):40. doi: 10.1080/03007995.2019.1583496.
14. Ng J. Delphi method: A qualitative approach for quantitative results. *Value Health* 2018;21:S54. Available at https://www.valueinhealthjournal.com/article/S1098-3015(18)30747-2/pdf.
15. Niederberger M, Spranger J. Delphi Technique in Health Sciences: A Map. *Front Public Health* 2020;8:457. doi: 10.3389/fpubh.2020.00457.

For peer review only - http://bmjopen.bmj.com/site/about/guidelines.xhtml

Section: References

16. Waggoner J, Carline JD, Durning SJ. Is There a Consensus on Consensus Methodology? Descriptions and Recommendations for Future Consensus Research. *Acad Med* 2016;91:663-8. doi: 10.1097/ACM.0000000000001092.
17. Wang X, Chen Y, Yang N, et al. Methodology and reporting quality of reporting guidelines: systematic review. *BMC Med Res Methodol* 2015;15:74. doi: 10.1186/s12874-015-0069-z.
18. Grant S, Booth M, Khodyakov D. Lack of preregistered analysis plans allows unacceptable data mining for and selective reporting of consensus in Delphi studies. *J Clin Epidemiol* 2018;99:96-105. doi: 10.1016/j.jclinepi.2018.03.007.

For peer review only

PRISMA 2020 Checklist

Section and Topic	Item #	Checklist item	Location where item is reported
**TITLE**			
Title	1	Identify the report as a systematic review.	Page 1
**ABSTRACT**			
Abstract	2	See the PRISMA 2020 for Abstracts checklist.	Page 2
**INTRODUCTION**			
Rationale	3	Describe the rationale for the review in the context of existing knowledge.	Page 4, 5
Objectives	4	Provide an explicit statement of the objective(s) or question(s) the review addresses.	Page 5
**METHODS**			
Eligibility criteria	5	Specify the inclusion and exclusion criteria for the review and how studies were grouped for the syntheses.	Page 5
Information sources	6	Specify all databases, registers, websites, organisations, reference lists and other sources searched or consulted to identify studies. Specify the date when each source was last searched or consulted.	Page 6
Search strategy	7	Present the full search strategies for all databases, registers and websites, including any filters and limits used.	Online supplemental material 2
Selection process	8	Specify the methods used to decide whether a study met the inclusion criteria of the review, including how many reviewers screened each record and each report retrieved, whether they worked independently, and if applicable, details of automation tools used in the process.	Page 6
Data collection process	9	Specify the methods used to collect data from reports, including how many reviewers collected data from each report, whether they worked independently, any processes for obtaining or confirming data from study investigators, and if applicable, details of automation tools used in the process.	Page 6, 7
Data items	10a	List and define all outcomes for which data were sought. Specify whether all results that were compatible with each outcome domain in each study were sought (e.g. for all measures, time points, analyses), and if not, the methods used to decide which results to collect.	Online supplemental material 3
	10b	List and define all other variables for which data were sought (e.g. participant and intervention characteristics, funding sources). Describe any assumptions made about any missing or unclear information.	Online supplemental material 3
Study risk of bias assessment	11	Specify the methods used to assess risk of bias in the included studies, including details of the tool(s) used, how many reviewers assessed each study and whether they worked independently, and if applicable, details of automation tools used in the process.	Not applicable
Effect measures	12	Specify for each outcome the effect measure(s) (e.g. risk ratio, mean difference) used in the synthesis or presentation of results.	Not applicable
Synthesis methods	13a	Describe the processes used to decide which studies were eligible for each synthesis (e.g. tabulating the study intervention characteristics and comparing against the planned groups for each synthesis (item #5)).	Not applicable
	13b	Describe any methods required to prepare the data for presentation or synthesis, such as handling of missing summary statistics, or data conversions.	Not applicable
	13c	Describe any methods used to tabulate or visually display results of individual studies and syntheses.	Not applicable
	13d	Describe any methods used to synthesize results and provide a rationale for the choice(s). If meta-analysis was performed, describe the model(s), method(s) to identify the presence and extent of statistical heterogeneity, and software package(s) used.	Not applicable

For peer review only - http://bmjopen.bmj.com/site/about/guidelines.xhtml

PRISMA 2020 Checklist

Section and Topic	Item #	Checklist item	Location where item is reported
	13e	Describe any methods used to explore possible causes of heterogeneity among study results (e.g. subgroup analysis, meta-regression).	Not applicable
	13f	Describe any sensitivity analyses conducted to assess robustness of the synthesized results.	Not applicable
Reporting bias assessment	14	Describe any methods used to assess risk of bias due to missing results in a synthesis (arising from reporting biases).	Not applicable
Certainty assessment	15	Describe any methods used to assess certainty (or confidence) in the body of evidence for an outcome.	Not applicable
**RESULTS**			
Study selection	16a	Describe the results of the search and selection process, from the number of records identified in the search to the number of studies included in the review, ideally using a flow diagram.	Page 7 Fig 1
	16b	Cite studies that might appear to meet the inclusion criteria, but which were excluded, and explain why they were excluded.	Page 8, Fig 1
Study characteristics	17	Cite each included study and present its characteristics.	Page 8
Risk of bias in studies	18	Present assessments of risk of bias for each included study.	Not applicable
Results of individual studies	19	For all outcomes, present, for each study: (a) summary statistics for each group (where appropriate) and (b) an effect estimate and its precision (e.g. confidence/credible interval), ideally using structured tables or plots.	Table 1 and 2<br>Online supplemental material 4, 5 and 6
Results of syntheses	20a	For each synthesis, briefly summarise the characteristics and risk of bias among contributing studies.	Not applicable
	20b	Present results of all statistical syntheses conducted. If meta-analysis was done, present for each the summary estimate and its precision (e.g. confidence/credible interval) and measures of statistical heterogeneity. If comparing groups, describe the direction of the effect.	Not applicable
	20c	Present results of all investigations of possible causes of heterogeneity among study results.	Not applicable
	20d	Present results of all sensitivity analyses conducted to assess the robustness of the synthesized results.	Not applicable
Reporting biases	21	Present assessments of risk of bias due to missing results (arising from reporting biases) for each synthesis assessed.	Not applicable
Certainty of evidence	22	Present assessments of certainty (or confidence) in the body of evidence for each outcome assessed.	Not applicable
**DISCUSSION**			
Discussion	23a	Provide a general interpretation of the results in the context of other evidence.	Page 11-13
	23b	Discuss any limitations of the evidence included in the review.	Page 3, 11, 12

For peer review only

PRISMA 2020 Checklist

Section and Topic	Item #	Checklist item	Location where item is reported
	23c	Discuss any limitations of the review processes used.	Page 3, 11-13
	23d	Discuss implications of the results for practice, policy, and future research.	Page 13,14
**OTHER INFORMATION**			
Registration and protocol	24a	Provide registration information for the review, including register name and registration number, or state that the review was not registered.	Page 1, 5
	24b	Indicate where the review protocol can be accessed, or state that a protocol was not prepared.	Page 5 Online supplemental material 1 ref 13 and 15
	24c	Describe and explain any amendments to information provided at registration or in the protocol.	Online supplemental material 1
Support	25	Describe sources of financial or non-financial support for the review, and the role of the funders or sponsors in the review.	Page 14
Competing interests	26	Declare any competing interests of review authors.	Page 14
Availability of data, code and other materials	27	Report which of the following are publicly available and where they can be found: template data collection forms; data extracted from included studies; data used for all analyses; analytic code; any other materials used in the review.	Online supplemental material 1-6

From: Page MJ, McKenzie JE, Bossuyt PM, Boutron I, Hoffmann TC, Mulrow CD, et al. The PRISMA 2020 statement: an updated guideline for reporting systematic reviews. BMJ 2021;372:n71. doi: 10.1136/bmj.n71

For more information, visit: http://www.prisma-statement.org/

For peer review only

PRISMA 2020 for Abstracts Checklist

Section and Topic	Item #	Checklist item	Reported (Yes/No)
**TITLE**			
Title	1	Identify the report as a systematic review.	Yes
**BACKGROUND**			
Objectives	2	Provide an explicit statement of the main objective(s) or question(s) the review addresses.	Yes
**METHODS**			
Eligibility criteria	3	Specify the inclusion and exclusion criteria for the review.	Yes
Information sources	4	Specify the information sources (e.g. databases, registers) used to identify studies and the date when each was last searched.	Yes
Risk of bias	5	Specify the methods used to assess risk of bias in the included studies.	Not applicable
Synthesis of results	6	Specify the methods used to present and synthesise results.	Not applicable
**RESULTS**			
Included studies	7	Give the total number of included studies and participants and summarise relevant characteristics of studies.	Yes
Synthesis of results	8	Present results for main outcomes, preferably indicating the number of included studies and participants for each. If meta-analysis was done, report the summary estimate and confidence/credible interval. If comparing groups, indicate the direction of the effect (i.e. which group is favoured).	Yes
**DISCUSSION**			
Limitations of evidence	9	Provide a brief summary of the limitations of the evidence included in the review (e.g. study risk of bias, inconsistency and imprecision).	Not applicable
Interpretation	10	Provide a general interpretation of the results and important implications.	Yes
**OTHER**			
Funding	11	Specify the primary source of funding for the review.	Not in abstract, in main document
Registration	12	Provide the register name and registration number.	Yes

From: Page MJ, McKenzie JE, Bossuyt PM, Boutron I, Hoffmann TC, Mulrow CD, et al. The PRISMA 2020 statement: an updated guideline for reporting systematic reviews. BMJ 2021;372:n71. doi: 10.1136/bmj.n71

For peer review only

PRISMA 2020 for Abstracts Checklist

For more information, visit: http://www.prisma-statement.org/

For peer review only
